# Discovery and Early Optimization of 1*H*-Indole-2-carboxamides with Anti-*Trypanosoma cruzi* Activity

**DOI:** 10.1021/acs.jmedchem.4c02942

**Published:** 2025-03-31

**Authors:** Ramon G. de Oliveira, Luiza R. Cruz, Marco A. Dessoy, Paul J. Koovits, Deborah A. dos Santos, Luiz F. N. de Oliveira, Rafael A. Ferreira, María C. Mollo, Eun Lee, Simone M. Duarte, Renata Krogh, Leonardo L. G. Ferreira, Rafael C. Chelucci, Maria Dichiara, Quillon J. Simpson, Clarissa Feltrin, Adriana C. da Silva, Benedito M. dos Santos, Milena F. Broering, Michael P. Pollastri, Lori Ferrins, Carolina B. Moraes, Adriano D. Andricopulo, Jadel M. Kratz, Peter Sjö, Charles E. Mowbray, Luiz C. Dias

**Affiliations:** † Institute of Chemistry, State University of Campinas, Campinas 13083-862, Brazil; ‡ São Carlos Institute of Physics, University of São Paulo, São Carlos 13563-120, Brazil; ^§^Chemistry and Chemical Biology and ^∥^Pharmaceutical Sciences, Northeastern University, Boston, Massachusetts 02115, United States; ^⊥^Institute of Biomedical Sciences and ^#^School of Pharmaceutical Sciences, University of São Paulo, São Paulo 05508-000, Brazil; ∇ Drugs for Neglected Diseases initiative, Rio de Janeiro 20010-020, Brazil; ○ Drugs for Neglected Diseases initiative, Geneva 1202, Switzerland

## Abstract

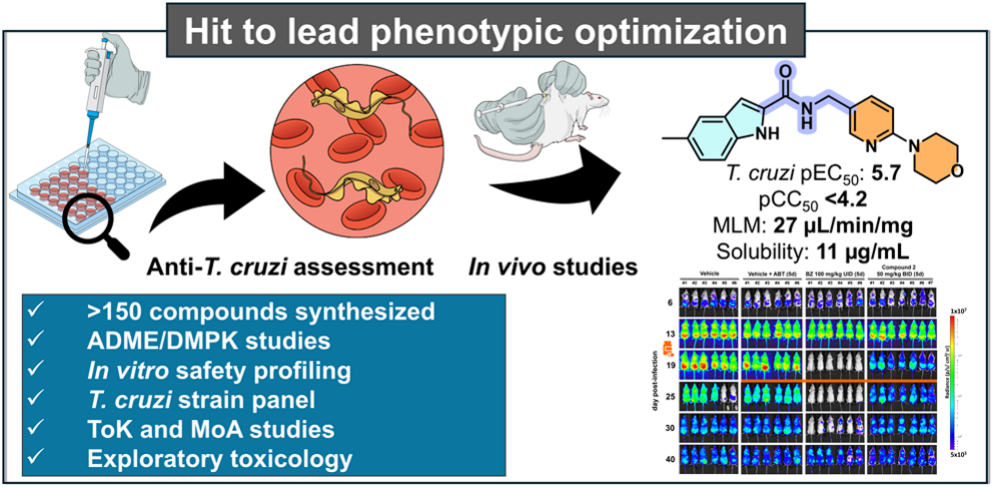

Chagas disease (CD), caused by the flagellate protozoan *Trypanosoma cruzi*, is a neglected tropical disease endemic in 21 countries. The only two antiparasitic drugs approved for its treatment, benznidazole and nifurtimox, have significant drawbacks. We present herein the optimization of a series of substituted indoles that were identified through phenotypic screening against *T. cruzi*. Early lead compounds with balanced potency and physicochemical properties were advanced to animal studies but showed limited plasma exposure. Medicinal chemistry strategies were used to improve metabolic stability and solubility, but unfortunately, this effort failed to yield compounds with improvements in both exposure and potency. Still, the best compound was progressed for a proof-of-concept efficacy study using acute and chronic mice models of Chagas disease. Despite showing antiparasitic activity in these in vivo studies, the optimization work with this series was stopped due to unfavorable drug metabolism and pharmacokinetic (DMPK) properties and a deprioritized mechanism of action (CYP51 inhibition).

## Introduction

Chagas disease (CD) is a potentially fatal parasitic infection caused by protozoan *Trypanosoma cruzi*. CD is endemic to the Americas and belongs to a group of diseases which have been classed as neglected tropical diseases (NTDs) by the World Health Organization (WHO).^1^ NTDs are a leading cause of morbidity and mortality in developing countries and receive little attention and investment compared to other areas of pharmaceutical research and development (R&D). CD is a significant health problem, with an estimated 160,000 new cases reported in 2021,^2^ and, due to changes in climate and population migration, it has crossed international borders and become a global concern.^3^

The acute phase of CD starts days after infection and spontaneously resolves in most patients. Antitrypanosomal treatment is highly recommended at this stage and has a success rate of up to 80–90%. Lack of treatment can lead to progression to a chronic phase in either the indeterminate (asymptomatic) or determinate (characterized by patients with cardiac or digestive disease) form, with symptoms appearing 10–15 years after the initial infection. Antitrypanosomal treatment during the chronic stage has a variable success rate, but it is strongly recommended for reactivated infections and for all children and patients up to 18 years of age with chronic disease.^4^

The only antiparasitic drugs available, benznidazole (BZ) and nifurtimox, have limited efficacy, require long treatment periods,^5^ and a proportion of patients suffer severe side effects.^6^ As such, new treatments that meet the target product profiles (TPPs) described by the WHO and the Drugs for Neglected Diseases initiative (DNDi) are urgently needed.^7^ However, the global R&D pipeline for CD is rather limited, and recent clinical trials with new chemical entities have produced poor results; posaconazole and E1224 (the prodrug of ravuconazole), both *T. cruzi* sterol 14α-demethylase (*TcCYP51*) inhibitors, and fexinidazole, a nitroimidazole, failed to show clinical benefit and development programs were stopped.^8−10^

NTDs generally suffer from a lack of R&D investment and coordination, and progression of compounds from the early discovery stage into preclinical and clinical studies is very rare.^11,12^ We believe this gap is best addressed by integrating academic drug discovery efforts with industry and public-private partnerships. The work presented herein was led by the Drugs for Neglected Diseases initiative’s Lead Optimization Latin America (LOLA) consortium, created in 2013, with the aim of identifying and developing preclinical candidates for the treatment of neglected diseases while enhancing existing R&D potential in endemic regions. The consortium operates through an organized network of academic and research institutions coordinated by DNDi, leveraging expertise and resources from its partners. Since its inception, LOLA has made significant progress in advancing drug discovery and development in the region, training researchers and developing the region’s technical and infrastructure capabilities.

**Figure 1:**
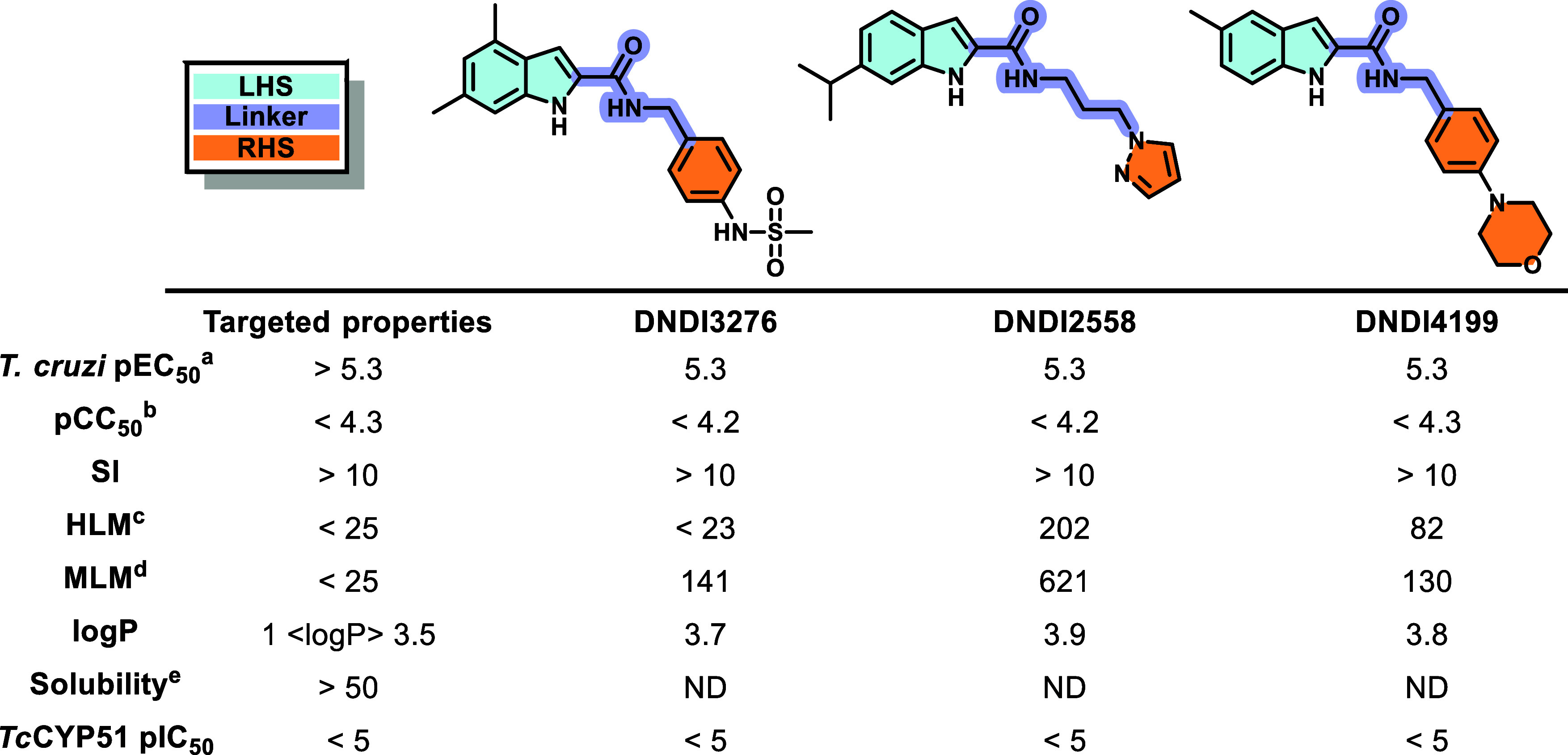
Initial hits of indole series. ^a^*T. cruzi* intracellular amastigote potency, strain: X10/7 A1; ^b^Cytotoxicity was measured in Vero cells. These experiments were conducted in one biological replicate; ^c^Human liver microsome intrinsic clearance (Clint—μL/min/mg); ^d^Mouse liver microsome intrinsic clearance (Clint—μL/min/mg); ^e^Kinetic solubility in phosphate-buffered saline (PBS) (μg/mL).

Hit identification was conducted through cell-based high-content screening (HCS) of a commercial library of small drug-like molecules.^13^ In this campaign, three hits containing an indole core were identified as active against the intracellular amastigote forms of *T. cruzi*. These hits had moderate *in vitro* potency and good selectivity over the host cells (Figure 1). For CD, a good hit is classified by a pEC_50_ > 5.5 (ideally pEC_50_ > 6.0) against intracellular *T. cruzi* amastigotes combined with at least 10-fold selectivity.^14,15^ Additionally, as part of our initial hit assessment campaign, resynthesized hits had their *in vitro* potency confirmed and were screened against recombinant *Tc*CYP51 using a direct biochemical assay. This is an important step, given that this mechanism of action (MoA) has been associated with clinical failure and is currently deprioritized.^16^ Results revealed that *in vitro* potency against the parasite was not correlated with the inhibition of *Tc*CYP51 *in vitro* (all compounds were inactive at the maximum concentration tested of 10 μM).

We found out early on that the phenyl of DNDI4199 could be replaced with a pyridine (matched pair with **2**, Table 1), which conferred improved metabolic stability in MLM (though still high) and garnered a slight boost in potency. The lower lipophilicity conferred by the pyridyl ring was also deemed positive for the series development in general. With these preliminary results in hand, a hit-to-lead medicinal chemistry program was initiated with a focus on the improvement of the aqueous solubility, metabolic stability and to explore the structure–activity relationship (SAR), with the ultimate goal of identifying a preclinical candidate for CD. Two subseries were identified, bearing either a 4-phenylsulfonamide (typified by DNDI3276, Figure 1) or a 4-(2-pyridyl)morpholine substituent in the blue region. In the first tier of the screening cascade that supported the medicinal chemistry efforts, compounds were tested against *T. cruzi* Tulahuen LacZ intracellular amastigotes, cytotoxicity against HFF-1 and HepG2 cell lines, and experimental physicochemical (lipophilicity and kinetic solubility) and ADME (permeability and microsomal stability) properties were also measured.

**Table 1:**
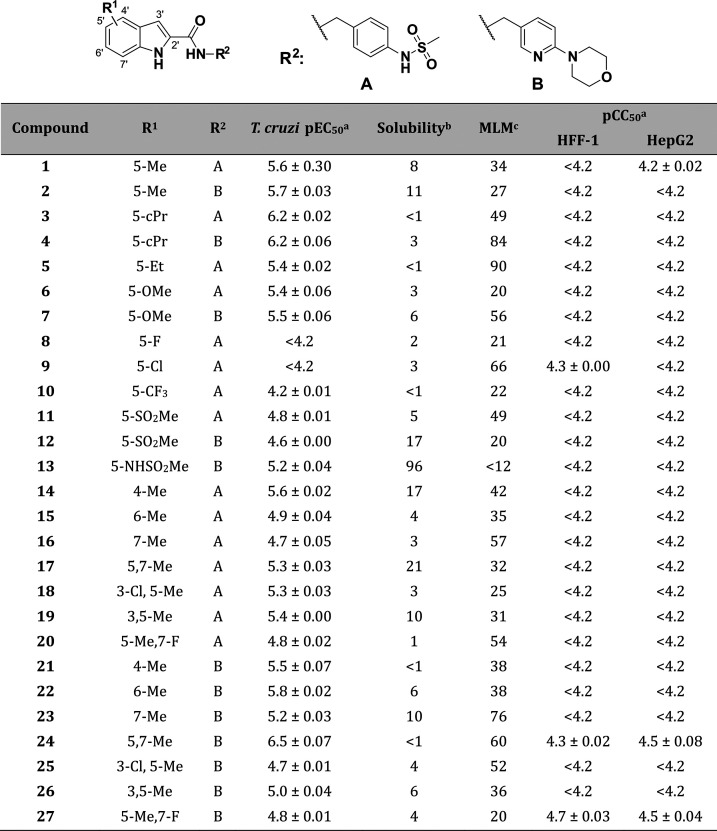
Modifications Exploring Different Substituents and Substitution Patterns on the Indole Core

a Values are shown as the average values from two separate experiments ± standard error of the mean (SEM).

b Kinetic solubility in PBS pH 7.4 (μg/mL).

c Mouse liver microsomes intrinsic clearance (Clint—μL/min/mg).

## Results and Discussion

### SAR Exploration and *In Vitro* Multiparametric Evaluation

First, different substituents in the 5′ position of the indole core of both subseries were synthesized (Table 1). Small, aliphatic, electron donating groups (EDG) were favored in this position—compounds bearing a methyl (**1** and **2**), cyclopropyl (**3** and **4**), ethyl (**5**), or methoxy (**6** and **7**) group showed moderate to good potency (5.4 < pEC_50_ < 6.2). Analogues containing electron-withdrawing groups (EWG) such as halogens (**8** and **9**) and the trifluoromethyl group (**10**) were inactive (pEC_50_ < 4.2). Other groups such as sulfone (**11** and **12**) and a sulfonamide (**13**) restored some of the potency (pEC_50_ 4.5–5.2); interestingly, **13** also had increased solubility (96 μg/mL) and improved metabolic stability (<12 μL/min/mg). Different substitution patterns were also probed for their tolerance of EDGs and EWGs. Within the 4-phenylsulfonamide subseries, the 4′ and 5′ positions (**14** and **1**) were favored over the 6′ and 7′ positions (**15** and **16**). Double substitution, as seen in **17**–**19** and, to a lesser extent, in **20**, was also tolerated. In the 4-(2-pyridyl)morpholine subseries, there was no preference for position: **2** and **21**–**23** had similar activity against the intracellular parasite. On the other hand, **24** was 1-log unit more potent than the initial hits, with a pEC_50_ of 6.5, though some cytotoxicity was introduced there was still a good selectivity window. The other compounds with two substituents on the indole (**25**–**27**) did not have the same tendency. Although the potency of some of the analogues was improved, most compounds of the series had low solubility after this initial analysis. The maintenance of an overall good cytotoxicity profile and controlled microsomal stability was encouraging.

Next, replacement of the indole scaffold was explored to identify the structural requirements driving potency and to identify different chemical space for further modifications (Table 2). Isoquinolines **28** and **29**, azaindole **30**, benzofuran **31**, and benzimidazole **32** were inactive. Regioisomers of **1** and **2**, moving the substituents from 2′ to 1′ (**33** and **34**) and 3′ positions (**35**), also had reduced potency. Analogues **33** and **34** had an improved solubility profile, but this was accompanied by an increase in intrinsic clearance. None of the indole replacements tested seemed to have better overall properties, so this was retained for further SAR exploration and optimization.

**Table 2:**
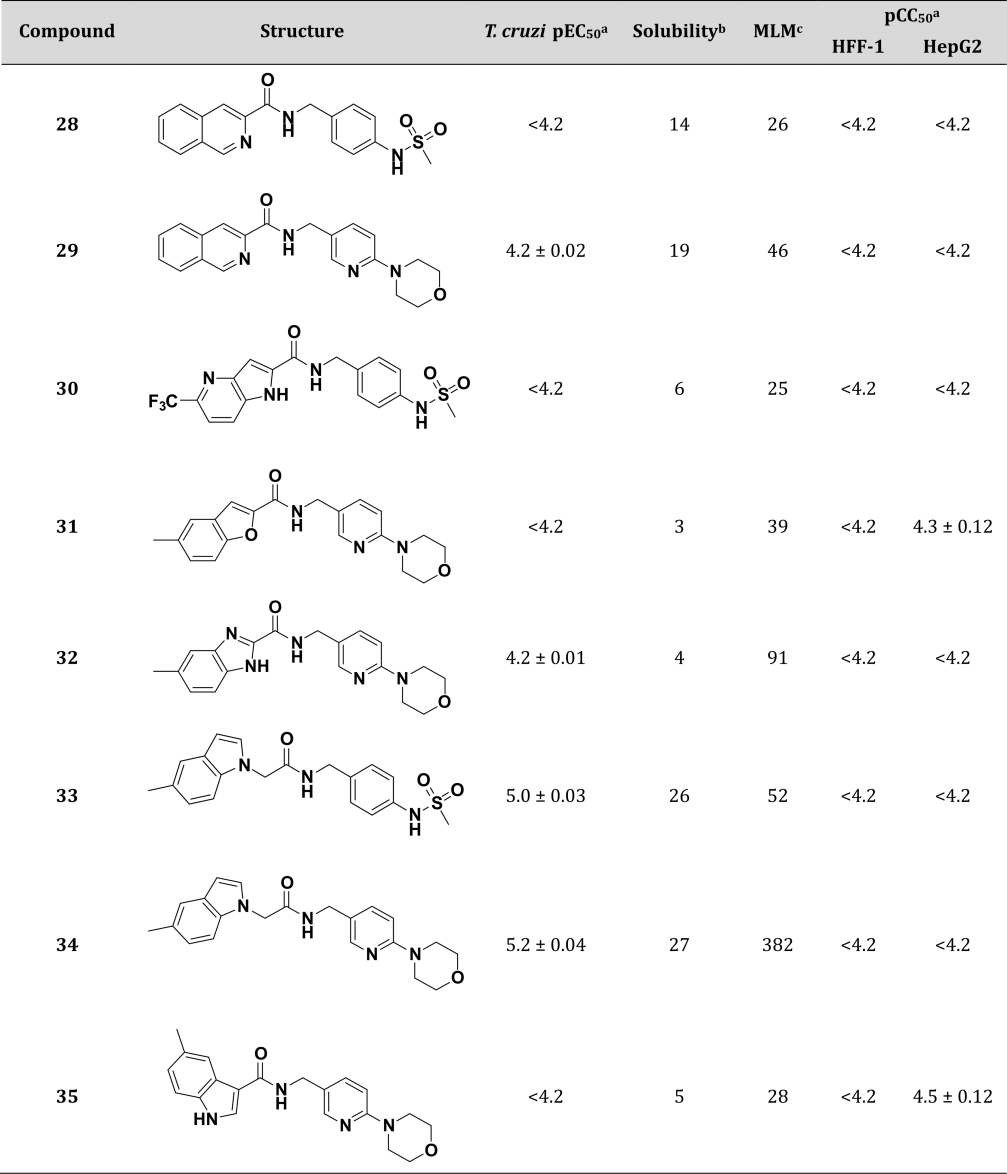
Application of a Scaffold Hopping Strategy to Replace the Indole

a Values are shown as the average values from two separate experiments ± SEM.

b Kinetic solubility in PBS pH 7.4 (μg/mL).

c Mouse liver microsomes intrinsic clearance (Clint—μL/min/mg).

We then focused on modifications to the sulfonamide and morpholine groups (Tables 3 and 4, respectively) to improve potency and modulate drug metabolism and pharmacokinetic (DMPK) properties. We probed analogues with both methyl and cyclopropyl substituents in the 5′ position, since early studies conducted to identify metabolic soft spots (human and mouse S9 fractions) showed hydroxylation at these regions were the main routes of metabolism (Supporting information (SI)—Figures S1–S6).

**Table 3:**
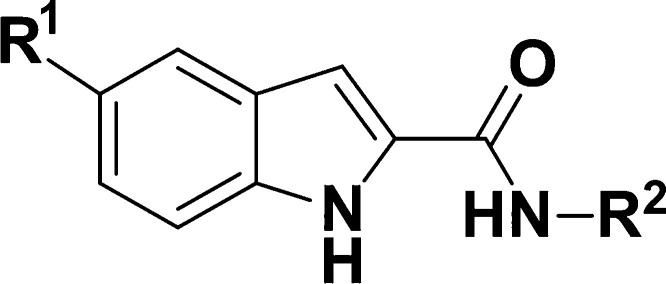

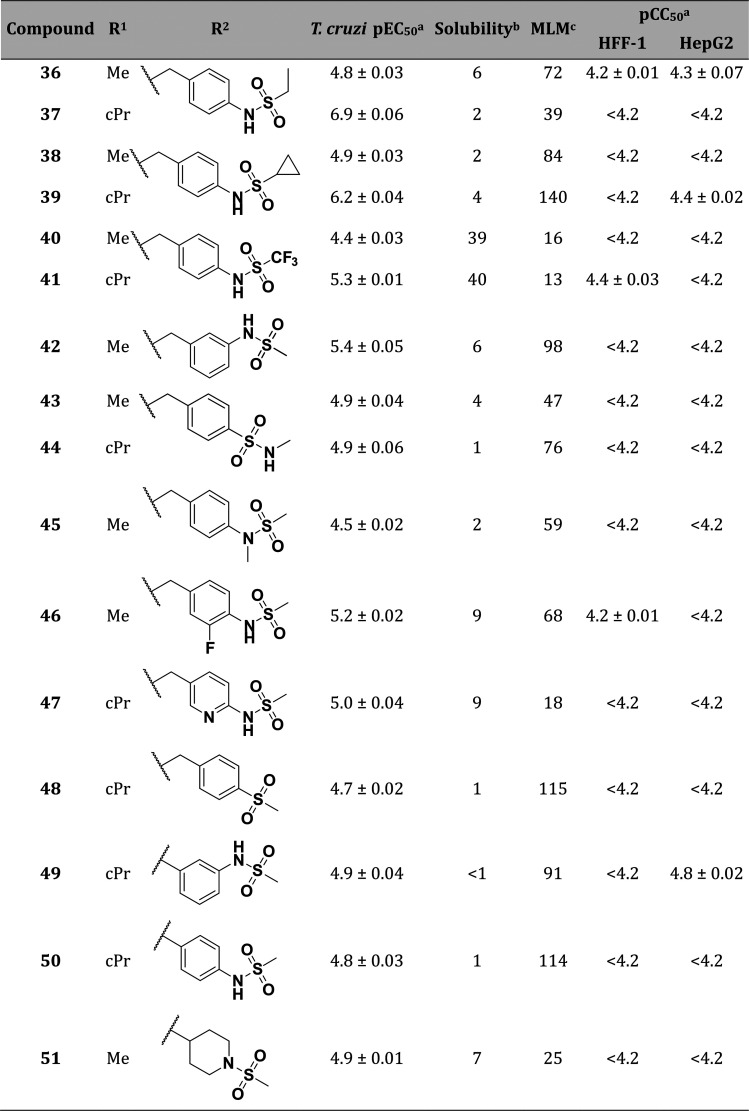
Modifications Exploring the RHS—Sulfonamide Derivatives

a Values are shown as the average values from two separate experiments ± SEM.

b Kinetic solubility in PBS pH 7.4 (μg/mL).

c Mouse liver microsomes intrinsic clearance (Clint—μL/min/mg).

**Table 4:**
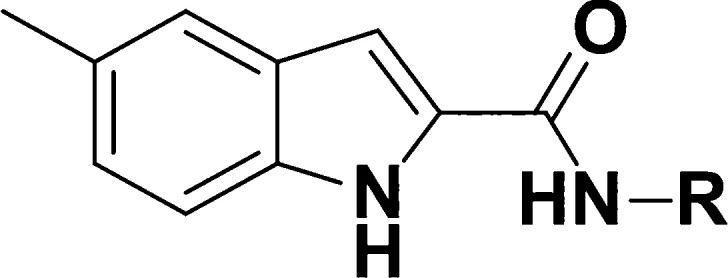

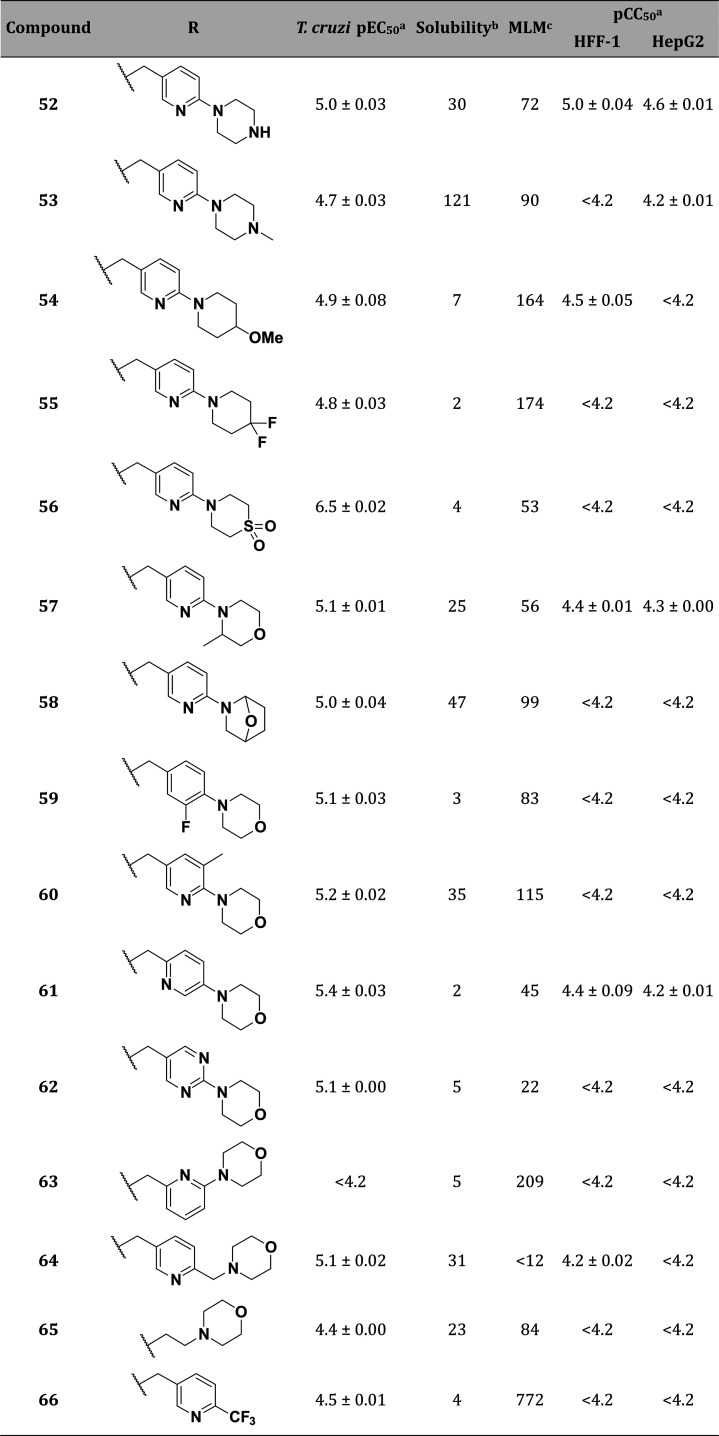
Modifications Exploring the RHS—Pyridylmorpholine Derivatives

a Values are shown as the average values from two separate experiments ± SEM.

b Kinetic solubility in PBS pH 7.4 (μg/mL).

c Mouse liver microsomes intrinsic clearance (Clint—μL/min/mg).

Analogues of **1** and **3** were generally less active than their parent compounds. Two notable exceptions were the ethyl (**37**) and cyclopropyl (**39**) analogues, with a cyclopropyl substituent in the 5′ position and a pEC_50_ of 6.9 and 6.2, respectively. Their 5-Me counterparts (**36** and **38**) were less active (pEC_50_ < 5.0). Adding a trifluoromethyl in the sulfonamide (**40** and **41**) gave compounds with lower potency but increased solubility (39 and 40 μg/mL, respectively) and good metabolic profiles (16 and 13 μL/min/mg, respectively). Moving the sulfonamide to the meta position as in **42** did not affect potency or solubility. Reversing the sulfonamide (**43** and **44**) or methylating it (**45**) resulted in less potent compounds (pEC_50_ < 5.0), making the secondary amide crucial to activity. Adding a fluorine (**46**) or changing to a pyridyl ring (**47**) ortho to the sulfonamide gave equipotent compounds (pEC_50_ 5.2 and 5.0, respectively). Replacing the sulfonamide for a sulfone (**48**) yielded a less potent compound (pEC_50_ < 5), also reflecting the need for the nitrogen in that position. Removing the methylene in the spacer (**49**, **50**, and **51**), and for the case of **51** replacing the phenyl ring for an aliphatic ring, led to a loss of potency (pEC_50_ < 5), possibly due to changes in the spatial configuration of the sulfonamide. With a few exceptions, changes in the sulfonamide RHS were not tolerated, giving compounds with worse potency and/or absorption, distribution, metabolism, and excretion (ADME) profiles.

Modifications of the 4-(2-pyridyl)morpholine moiety are shown in Table 4. Replacement of the morpholine ring for a piperazine (**52**) or a methyl piperazine (**53**) improved solubility but resulted in less potent compounds (pEC_50_ 5.0 and 4.7, respectively) and the free amine showed signs of cytotoxicity (pCC_50_ 5.0). The 4-substituted piperidines, 4-methoxy **54** and 4,4-difluoro **55**, yielded compounds with a pEC_50_ < 5. Surprisingly, replacing the morpholine for a thiomorpholine 1,1-dioxide (**56**) resulted in one of the most potent compounds of the series, with a pEC_50_ of 6.5, but with slightly less favorable microsomal intrinsic clearance. Adding a methyl group to the 3′ position of the morpholine ring (**57**) did not improve potency, nor did replacing it with a bridged morpholine (**58**), giving equipotent compounds (pEC_50_∼5)—compound **58** did, however, have better solubility (47 μg/mL), in accordance with previous literature showing that adding one-carbon tethers to morpholine can reduce lipophilicity, hence modulating ADME properties.^17^ Replacing the pyridyl moiety with an ortho fluorophenyl (**59**) or adding a methyl on the meta position of the pyridyl moiety (**60**) also rendered less active compounds (pEC_50_ 5.1 and 5.2, respectively). Replacing the 2-pyridyl moiety for a 3-pyridyl gave **61** with similar potency to **2** (pEC_50_ 5.4), whereas introduction of a pyridimidin-5-yl (**62**) diminished the potency (pEC_50_ 5.1), albeit with a gain in metabolic stability (22 μL/min/mg). The analogue bearing the 6-morpholinopyridin-2-yl moiety (**63**) was inactive (pEC_50_ < 4.19). Bridging the morpholine and pyridine rings with a methylene group as in **64** partially abolished the potency (pEC_50_ 5.1), but with an improvement in solubility (31 μg/mL) and metabolic stability (<12 μL/min/mg). Finally, removal of the 3-pyridyl moiety and directly linking the morpholine to the carboxamide with an ethyl linker (**65**), or replacement of the morpholine for a trifluoromethyl group (**66**), resulted in inactive compounds (pEC_50_ 4.4 and 4.5, respectively). As for the sulfonamide group, the chemical space on the morpholine RHS seems to be a limiting factor in the exploration of SAR.

Continuing our exploratory SAR, changes in the linker between the indole core and the RHS moiety are shown in Table 5. Shifting the position of the methylene (**67**) or homologating the side chain (**68**) led to inactive compounds (pEC_50_ < 4.3). Potency was restored by reversing the amide as in **69**, with a similar potency to **2** (pEC_50_ 5.7), with improved solubility but higher metabolic instability. Replacing the carboxamide with the nonclassical isostere sulfonamide (**70**) also resulted in complete loss of potency (pEC_50_ 4.3). Branching the side chain with a methyl group (**71**) or *N*-methylating the amide (**72**) resulted in equipotent compounds (pEC_50_ ∼ 5). Interestingly, methylating both the amide and the indole −NHs (**73**) restored potency (pEC_50_ 5.8), with a similar potency to **2** and a better solubility profile (17 μg/mL). This could be partially due to returning the spatial orientation of the compound to its bioactive state.

**Table 5:**
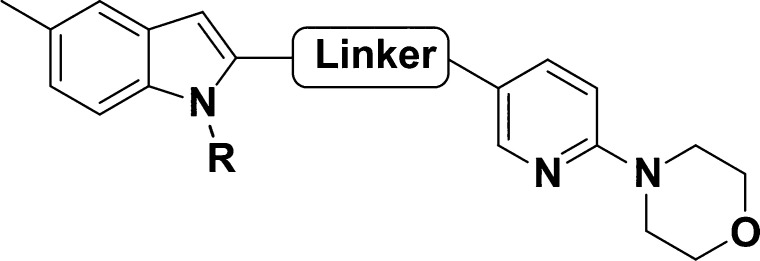

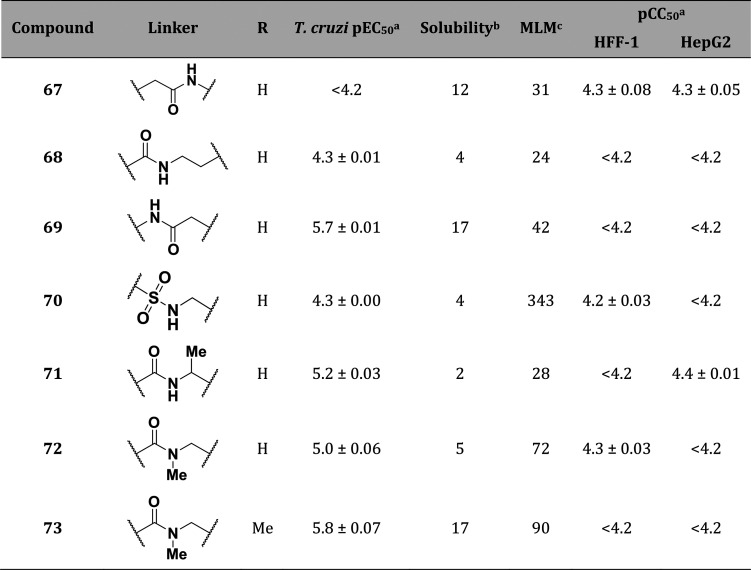
Modifications Exploring the Linker Region

a Values are shown as the average values from two separate experiments ± SEM.

b Kinetic solubility in PBS pH 7.4 (μg/mL).

c Mouse liver microsomes intrinsic clearance (Clint—μL/min/mg).

**Figure 2:**
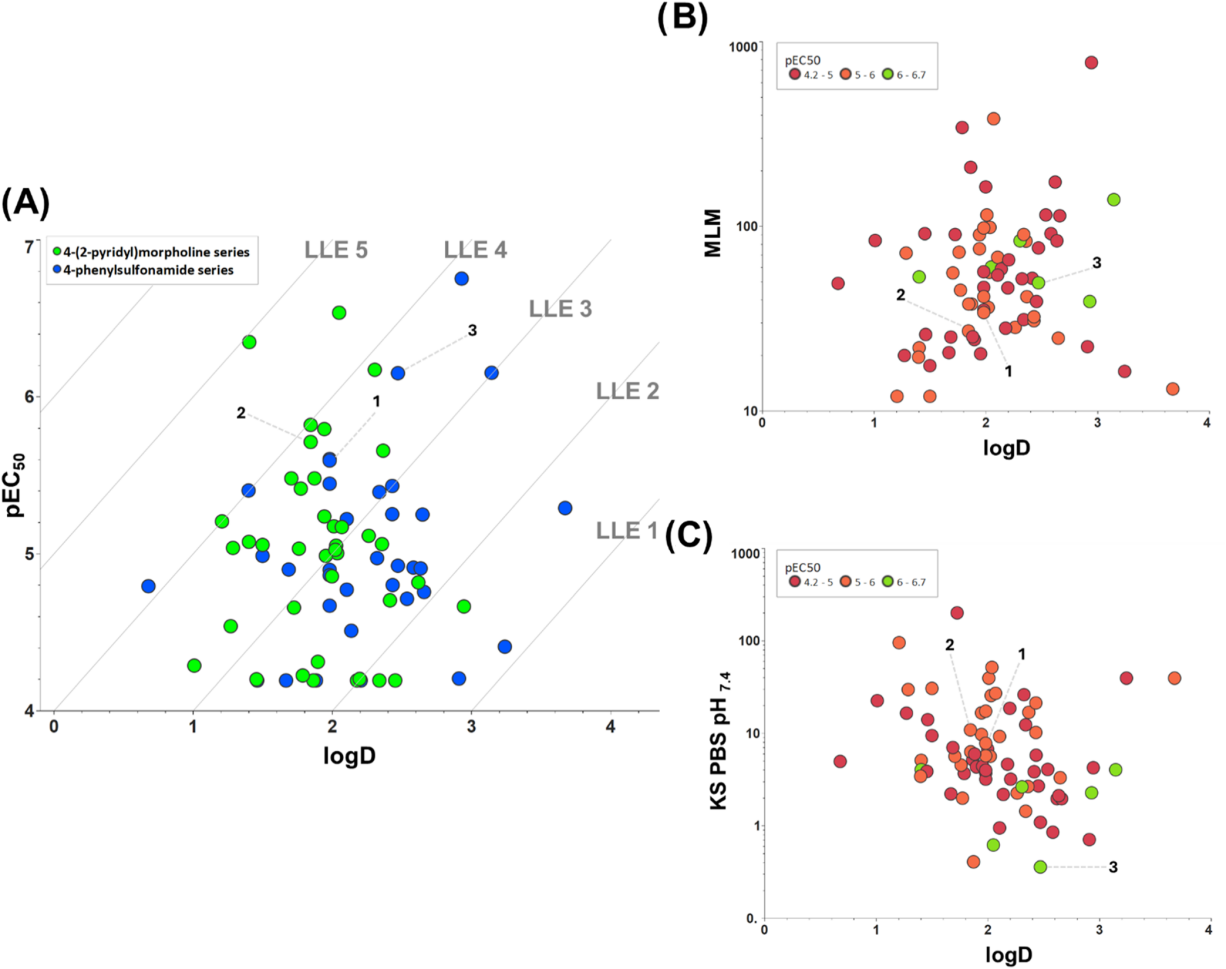
(A) LLE 2D-plot: pEC_50_ versus log *D* of compounds included in Tables 1–5 colored by RHS substituents. (B) 2-D plot of mouse microsomal stability (Clint—μL/min/mg) versus log *D* of compounds from Tables 1–5 colored by anti-*T. cruzi* activity. (C) 2-D plot of kinetic solubility (μg/mL) versus log *D* of compounds from Tables 1–5 colored by anti-*T. cruzi* activity.

When examining this first set of analogues, comprising 73 compounds with a range of diverse chemical features, it is noticeable that increasing potency and keeping the balance of properties is difficult, and that microsomal stability, and especially solubility, are the DMPK limiting factors for this series, hampering their progression to exploratory *in vivo* studies. Poor cellular permeability was not related to difficulties in increasing potency, since most compounds had high *in vitro* passive permeability, and selected compounds were not identified as possible Pgp substrates when assessed in the MDR1-MDCK assay (SI—Table S1). Moreover, a limited chemical space can be seen within the set of most active compounds (pEC_50_ > 5.5): only methyl and cyclopropyl substituents in the indole moiety, as well as little diversity in the RHS, are allowed. The lipophilic ligand efficiency^18^ (LLE = pEC_50_ – cLogP) plot in Figure 2A underpins the issue related to the series, with most compounds having LLE < 3 (ideally, LLE > 4); nonetheless, activity does not seem to be driven by lipophilicity alone. Moreover, physicochemical and ADME properties are similar within this set: general low kinetic solubility at pH_7.4_ (<10 μg/mL) and high microsomal clearance (>25 μL/min/mg). Again, there is no clear correlation between microsomal stability (based on MLM data) or solubility and lipophilicity, as shown in plots in Figure 2B,C, respectively.

With these issues in mind, strategies to overcome poor solubility and microsomal stability were put in place. After our initial exploration around the RHS of the sulfonamide subseries (Table 3), we found that trifluoromethyl analogues **40** and **41** improved ADME profiles and reduced lipophilicity. Modulation of the p*K*_a_ of acidic and basic groups by incorporation of vicinal fluorine is a well-known strategy in medicinal chemistry and many successful examples of improvement in potency and properties have been reported.^19^ We sought to use a “p*K*_a_ tuning strategy” to restore potency while maintaining the improved overall profile. With the experimental p*K*_a_ data for **3** and **41** (Table 6), we designed compounds that would have intermediate p*K*_a_ values, ranging from 4.1 to 8.5, to check whether a balance could indeed be found (the Goldilocks Effect). Compounds **74**–**76** were synthesized and tested. Tuning the electron-withdrawing effect did modulate potency and other properties, albeit moderately. Compounds **74** and **76** were less active than **3** (pEC_50_ 5.8), with a slightly better solubility profile; compound **75** was equipotent to **41** (pEC_50_ 5.3), with only a marginal gain in solubility. Interestingly, compound **76** had a much higher microsomal instability than **3** and the average for the series (62 versus 20 μL/min/mg). This could be due to its higher lipophilicty (experimental logD 4 versus 3.4, available in the SI).

**Table 6:**
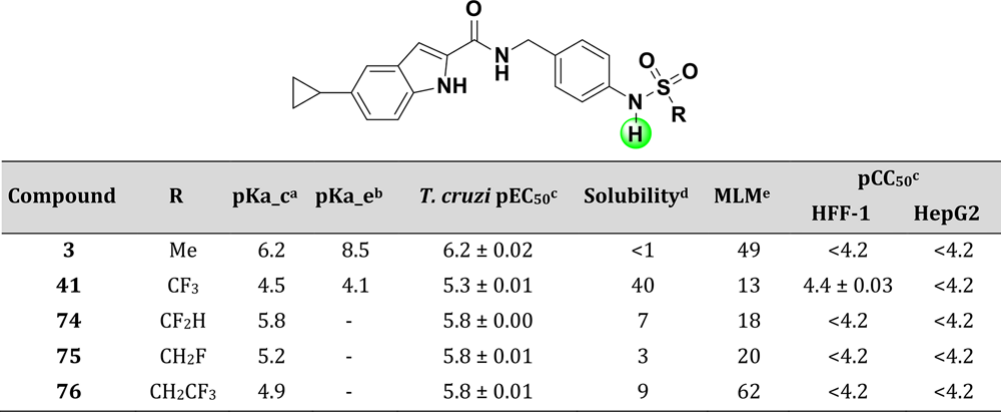
Modifications Using a p*K*_a_ Fine Tuning Strategy to Increase Solubility

a pka_c: calculated p*K*_a_.

b pKa_e: experimental p*K*_a_.

c Values are shown as the average values from two separate experiments ± SEM.

d Kinetic solubility in PBS pH 7.4 (μg/mL).

e Mouse liver microsomes intrinsic clearance (Clint—μL/min/mg).

At this point, it was clear that the planarity of these compounds was linked with their poor solubility. The high melting points measured for some representatives suggested high crystal lattice energies, and thermodynamic solubility determinations confirmed the poor aqueous solubility of the compounds (SI—Table S2). Interestingly, **73** had slightly increased solubility (2-fold) and a lower melting point (172.5 °C, triplicate) than its parent compound **2** (218.1 °C, triplicate). This could be attributed to the disruption of planarity and crystal packing—in fact, **73** presents itself in crystalline sheets, whereas **2** is an amorphous powder (experimental observation). We therefore designed and synthesized analogues bearing *N*-substitutions, aiming to modulate solubility (Table 7). Moreover, while designing these new sets of analogues, we also incorporated polar functional groups, expecting to decrease lipophilicity and further increase in solubility. As with **73**, **77** had similar potency to the parent compound **1** (pEC_50_ 5.6), and much higher kinetic solubility (158 versus 8 μg/mL—20-fold), but with a clear negative effect on microsomal stability (94 versus 34 μL/min/mg). The introduction of a hydroxyl group (**78**–**81**) did not drastically change potency overall, but lowered lipophilicity compared to their doubly methylated analogues, with clear solubility gains and retaining high clearance. Finally, despite the improvement of solubility, substituting the amide NH with a *N,N*-dimethylethylamine residue (**82**) or *N*-methylene nitrile (**83**) gave particularly metabolically unstable and equipotent compounds.

**Table 7:**
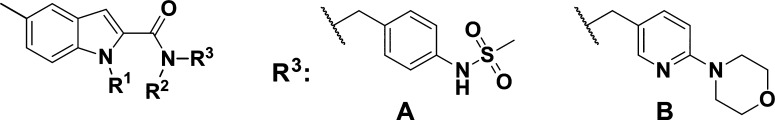

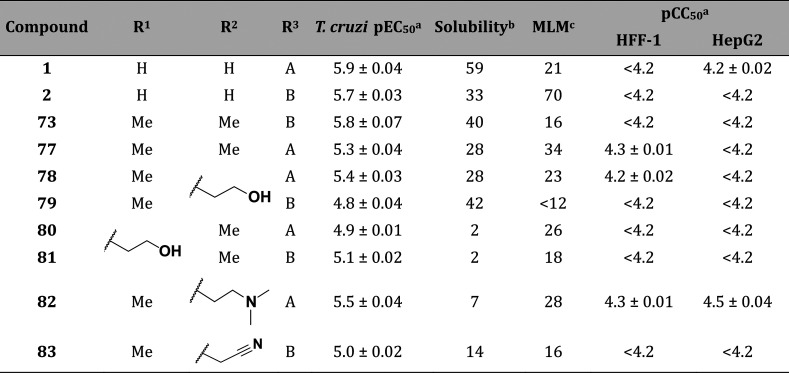
SAR of *N,N*-Substitution Strategy

a Values are shown as the average values from two separate experiments ± SEM.

b Kinetic solubility in PBS pH 7.4 (μg/mL).

c Mouse liver microsomes intrinsic clearance (Clint—μL/min/mg).

Our last strategy was to explore simplified 5-membered rings to mimic the indole scaffold, aimed at reduce lipophilicity and increasing the topological polar surface area (TPSA) via the addition of different heteroatoms (Table 8). These new, simpler monocycles could also provide new vectors and possibilities for SAR exploration. Initially, a set of pyrazole, oxazole, and isoxazole derivatives was synthesized and evaluated. Not surprisingly, the strategy succeeded in improving physicochemical and ADME properties: increased solubility, lower lipophilicity, and good microsomal stability. Pyrazole analogues **84** and **85** kept the average potency seen in this series (pEC_50_ 5.3 and 5.4, respectively) while oxazole derivatives **86** and **87** lost potency (pEC_50_ 4.5 and 5.0, respectively). The 3-cyclopropyl isoxazole derivatives **88** and **89** had their potency partially restored, with interesting solubility and clearance profiles, and a decrease in lipophilicity that enabled further exploration. We designed and synthesized a small set of analogues (**90**–**93**) to occupy the phenyl region of the indole. Unfortunately, installing a phenyl (**90** and **91**) or a 2-pyridyl (**92** and **93**) led to compounds with a pEC_50_ < 5.0, lower solubility and a similar metabolic profile to the cyclopropyl analogues.

**Table 8:**
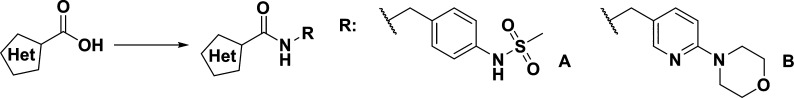

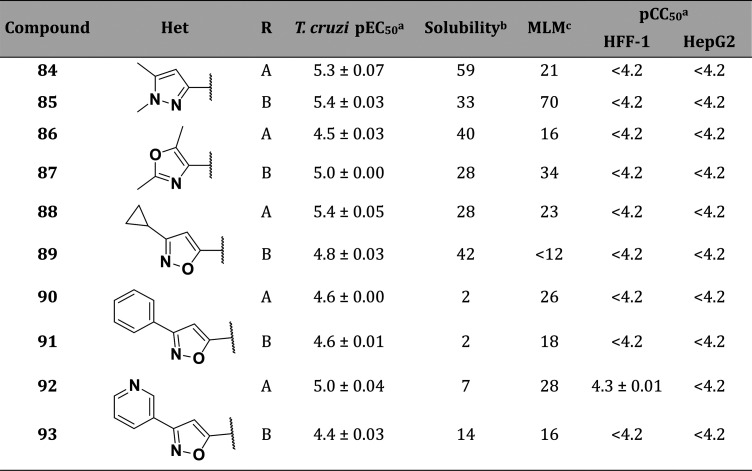
Simplifying the Indole Core

a Values are shown as the average values from two separate experiments ± SEM.

b Kinetic solubility in PBS pH 7.4 (μg/mL).

c Mouse liver microsomes intrinsic clearance (Clint—μL/min/mg).

In total, 153 compounds were designed, synthesized, and tested during this hit-to-lead campaign. The full list of compounds and data set is available in csv format (SI). In summary, moderately potent and balanced compounds can only be found within limited chemical space. A methyl or a cyclopropyl in the 5′ position of the indole seems to be ideal for potency. Unfortunately, all compounds with 1-log increase in potency (pEC_50_ > 6: **3**, **24**, **37**, and **56**) had limited solubility and poorer microsomal stability than **1** and **2**. On the other hand, compounds **13**, **41**, and **64**, had improved solubility and/or metabolic stability than **1** and **2**, but they were also less active (pEC_50_ < 5.3). With these results in hand, it was decided to progress **1, 2**, and **3** to exploratory pharmacokinetics (PK) studies *in vivo*.

### Secondary *In Vitro* and *In Vivo* Profiling of Frontrunners

After a single oral dose (50 mg/kg) in BALB/c mice, prioritized compounds **1**, **2**, and **3** did not reach free plasma exposure levels above the respective *in vitroT. cruzi* EC_50_ (media and mouse plasma protein binding, mouse plasma stability and full pharmacokinetics data are available in the SI—Table S3 and Figures S7–S9). Compound **3** had inferior *C*_max_ and AUC_inf_ values when compared with **1** and **2**, likely due to solubility-limited absorption—with 3-fold lower thermodynamic solubility in fasted state simulated intestinal fluid (FaSSIF) when compared with **2** (SI—Table S4). Compounds had moderate to high *in vivo* clearance, within the 24–57 mL/min/kg range. Using 1-aminobenzotriazole (1-ABT), a pan-CYP inhibitor, a pretreatment strategy increased the exposure 2.5–3-fold for **2** and **3**, showing that hepatic clearance is a relevant component of limiting exposure over time. Mouse liver microsome data was already pointing to possible rapid hepatic clearance *in vivo*, and studies with hepatocytes (human and mouse) showed moderate clearance, in good correlation with *in vivo* data (SI—Table S4). Mouse plasma instability was not related to the rapid clearance (Table S3).

Despite limited exposure, we aimed to investigate at least one compound in the acute Chagas model as a proof-of-concept (PoC) for the series. Due to the limited exposure of **3**, this compound was deprioritized. Considering the lower *in vivo* clearance of **2**, and its increased AUC with 1-ABT treatment, we hypothesized that a 50 mg/kg twice a day (BID) treatment with 1-ABT pretreatment could lead to exposure levels over the *in vitro* EC_50_.

First, we conducted an exploratory 5-day tolerability study in BALB/c mice at three oral dose levels of 50, 100, and 200 mg/kg once daily (QD) corresponding to exposure levels similar to those observed in CYP-inhibited mice. The results indicated that the compound was well tolerated at all dose levels, with no mortality or other noticeable signs of organ toxicity, and only a slight reduction in body weight (<10%) in the 100 and 200 mg/kg groups. Additionally, bioprofiling of **2** against a panel of known off-targets showed it was mostly clean, with just moderate inhibition of the human serotonin 5-HT_2_A receptor (SI—Table S5). hERG channel blocking was not an issue for compounds with the pyridylmorpholine, such as **2**, but **1** and other representatives of the sulfonamide subgroup showed moderate inhibition (IC_50_ ∼ 5 μM) (SI—Table S6).

**Figure 3:**
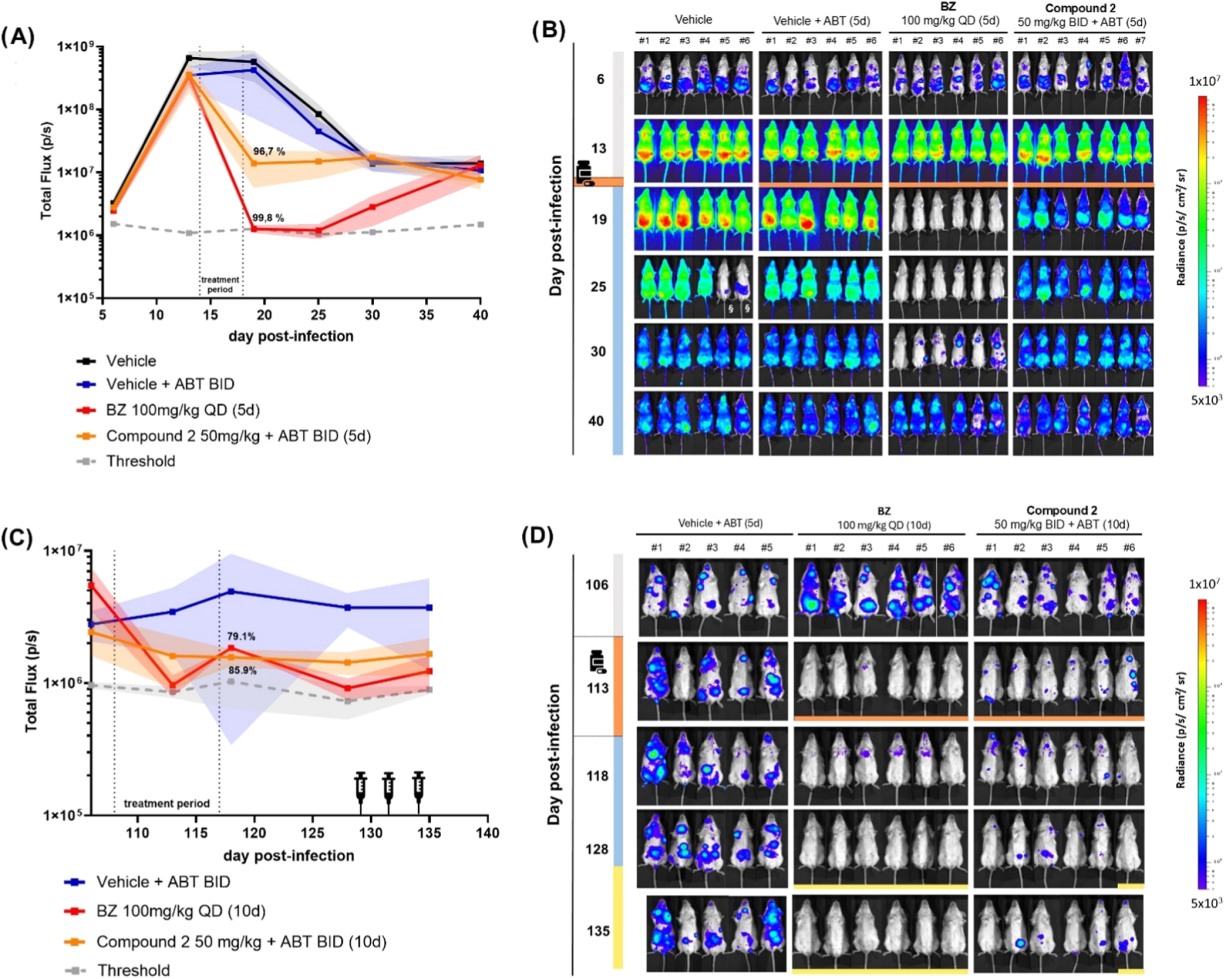
Compound **2** reduces parasite load in acute and chronic mice models of Chagas disease. Mice infected with *Trypanosoma cruzi* (*n* = 6–7/group) treated from 14 to 18 (acute stage) or 108 to 117 (chronic stage) days post-infection (dpi) with **2** at 50 mg/kg BID (twice daily) and co-treated with 1-aminobenzotriazole (1-ABT) at 50 mg/kg, compared to the standard treatment with BZ (100 mg/kg QD), vehicle only (HPMC-SV formulation) or vehicle with 1-ABT. Whole mice bioluminescence quantification of the acute (A) and chronic (C) PoC studies expressed as means and standard deviations (lines and shaded regions, respectively). Percentage reduction obtained by comparing the difference between the treated group and the paired nontreated vehicle group at the end of treatment. Bioluminescent images of infected mice from the acute (B) and chronic (D) PoC studies are shown on a log_10_ scale of signal intensity (low to high levels ranging from blue, to red). At 135 dpi, for the vehicle + ABT group, a representative image was included, and the calculations were based on the average vehicle values from the same experiment. Medicine flask indicates start of treatment (14 or 108 dpi) and orange bar shows end of treatment (18 or 117 dpi). Syringe icon and yellow bars indicate immunosuppression by cyclophosphamide (125 mg/kg i.p.).

Based on these results, we progressed **2** to acute and chronic *in vivo* efficacy studies. Female BALB/c mice (8-weeks old) were infected with bioluminescent *T. cruzi* (CL Brener Luc:Neon—DTU VI) parasites and treatment started either on the 14th day (acute) or on the 108th (chronic) day postinfection (Ethics Committee—CEUA ICB/USP Protocol nos. 7609141119 and 5787250522, respectively).^20^ In this model, parasitic load is determined by quantitatively assessing bioluminescence in the whole mouse, allowing spatial and longitudinal evaluation of drug efficacy against *T. cruzi*.^21^ The dose regimen chosen for **2** was 50 mg/kg BID orally for 5 days (acute) or 10 days (chronic) with pretreatment with 1-ABT 30 min before each dose at 50 mg/kg, aiming to increase exposure (*n* = 7 or 6/group). Blood samples were collected on day 1 and day 5 (acute) or day 10 (chronic) of treatment and compound concentration was measured (*n* = 3). In the acute study, **2** reduced 96.7% of the peak parasitemia at the end of treatment, compared with 99.8% reduction of the CD standard treatment BZ at 100 mg/kg QD (Figure 3A,B). After washout, both treatment groups had infection relapse. This is in line with previous reports showing that it is more difficult to achieve sterile cure in the acute rather than in the chronic stage of infection in this mouse model (for example, at this dose, BZ treatment usually requires 20 days of treatment to avoid parasitemia relapse).^21^ Given the antiparasitic activity observed, these results were considered a positive PoC for **2** and for the indole series. When evaluating the PK data, exposure on Day 1 up to 8 h was similar to the exposure seen in noninfected BALB/c mice, and free plasma exposure above EC_50_ was achieved for most of the treatment period. PK data also showed some compound accumulation, with higher free exposure on Day 5 (SI—Figure S10).

In the chronic infection study, **2** reduced parasitemia by 85.9% at the end of treatment, compared with 79.1% reduction by 100 mg/kg QD BZ (Figure 3C). At the end of the “washout” period, none of the mice from the BZ group and mouse #6 from the group that received compound **2** had detectable bioluminescence and were submitted to four cycles of immunosuppression with cyclophosphamide (CTX) at 125 mg/kg i.p. After immunosuppression, at 135 dpi, mice #6 showed recrudescence of infection (Figure 3D). All mice from the BZ group remained negative with no detectable bioluminescence. Free whole blood exposure of **2** was above EC_50_ throughout the treatment period (SI—Figure S10).

After analyzing the *in vivo* efficacy and exposure data, with the clear recrudescence of infection in both acute and chronic settings, we investigated further the *in vitro* antiparasitic activity of selected compounds to uncover the reason behind this. **1**, **2**, and **3** were profiled against a panel of *T. cruzi* strains from different lineages (TcI–TcIV) using an HCS-based assay.^16,22^ BZ was used as a control, and infected cells were exposed to compounds *in vitro* for 96 h. BZ activity was similar to previously published reports,^16,23^ with low micromolar potency and >85% maximum activity (maximum reduction of infection observed in comparison to controls) against all strains. Compounds **2** and **3** were active against all six strains (Sylvio X10/1—TcI; Y clH10—TcII; ARMA13 cl1—TcIII; ERA cl2—TcIV; 92–10 cl2, TcV and CL Brener, TcVI), albeit with some variability (Table 9). Compound **1** was active against most strains, with the exception of 92–80 cl2, against which its pEC_50_ could not be determined as the maximum activity was only 24%. Notably, all compounds had reduced maximum activity in comparison with BZ and were less active against the 92–80 cl2 strain. This clonal strain belongs to TcV, a group associated with slower *in vitro* growth/longer population doubling times and lower sensitivity to *Tc*CYP51 inhibitors.^16,24^

**Table 9:**
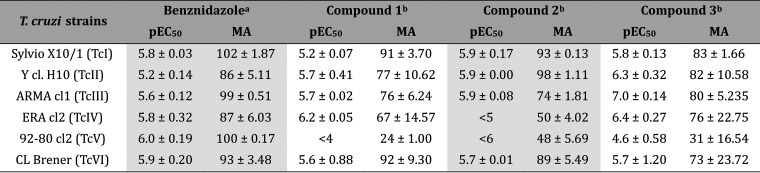
Activity of Compounds **1**, **2**, and **3** against a Panel of *T. cruzi* Strains^a^

a Data shown are the mean value of four (*a*) or two (*b*) independent experiments ± SEM. MA: maximum activity (%) ± SEM.

**Figure 4:**
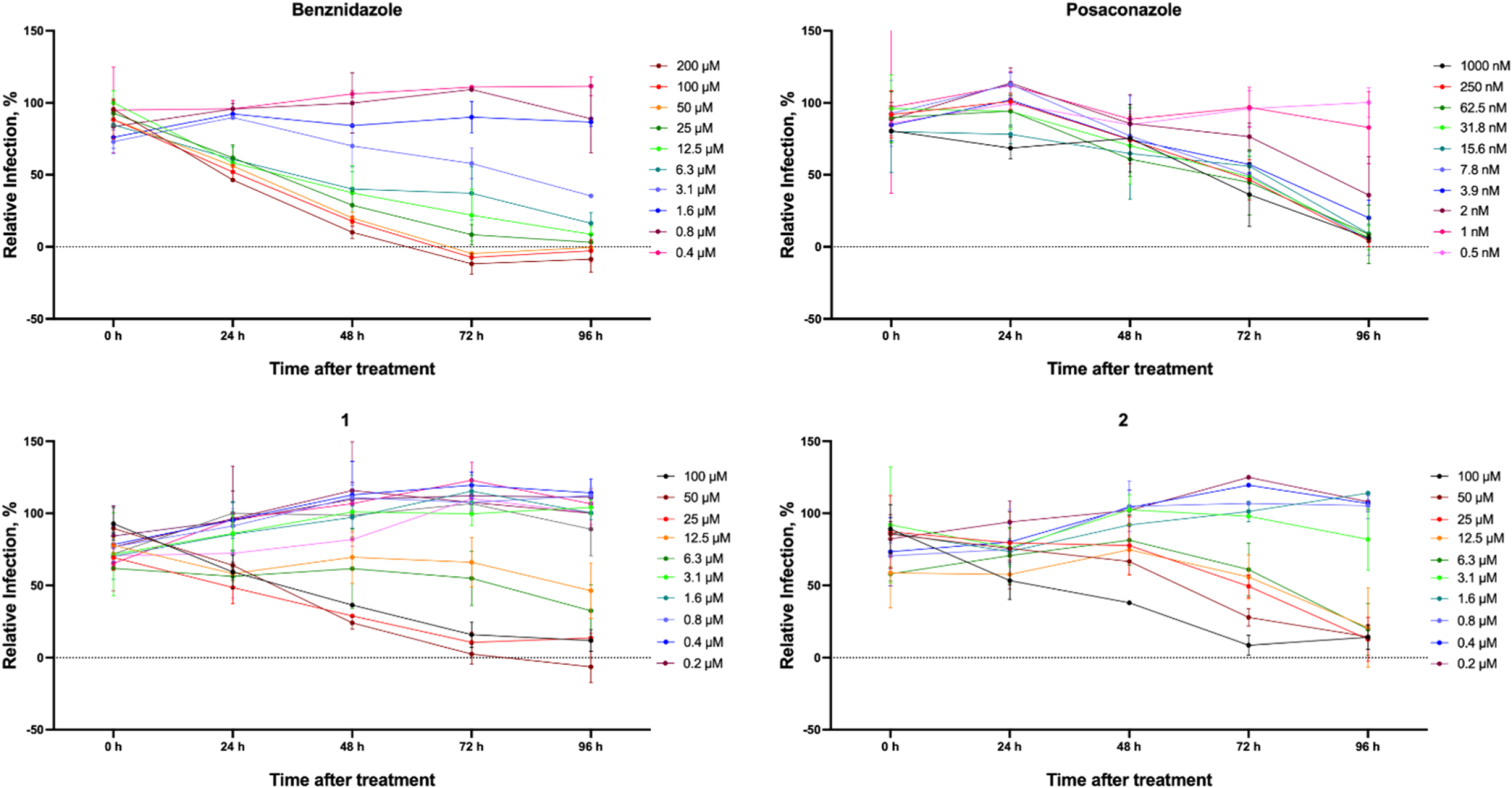
Compounds **1** and **2** had a slow-killing activity profile. BZ, posaconazole, **1** and **2** were tested in time-kill assays, with 14 concentration-points for each compound, for a total of 96 h of drug exposure. Representative drug concentrations are shown. The *X*-axis shows the measured levels of infection at each time point of drug exposure, and the *Y*-axis shows infection levels in relation to intraplate controls.

Compounds **1** and **2** were further profiled in a cell-based time-kill assay (Figure 4).^16^ BZ and posaconazole were used as controls. As expected, BZ had fast-killing, both time- and concentration-dependent, activity profile, with efficacious concentrations reducing infection from 24 h of exposure, and down to nondetectable infection levels by 72 h. Posaconazole, in contrast, had a slow-killing and mostly time-dependent activity profile, with the highest concentration tested greatly reducing infection (but not to undetectable levels) but only at 96 h. Compounds **1** and **2** also had a concentration- and time-dependent activity profile, with highest concentrations reducing infection most significantly from 48 h (**1**) and 72 h (**2**) onward, but they were not able to reduce parasitemia to undetectable levels at 96 h—resembling the subefficacious activity profile of posaconazole.

This profile prompted our team to reinvestigate the possible role of *Tc*CYP51 inhibition in the antiparasitic activity. A group of compounds was screened against the target using the same biochemical assay employed during early hit profiling, and data showed multiple compounds inhibiting *Tc*CYP51 with a similar range of potency to that identified for inhibition of the intracellular amastigotes (SI—Table S7 and Figure S11). In fact, there was a clear correlation between increased potency in the *in vitro T. cruzi* assay and *Tc*CYP51 inhibition for this set of compounds. It was hypothesized that a combination of *Tc*CYP51 and an unknown target(s) play a role in the antiparasitic activity identified for the series.

## Conclusions

In conclusion, based on the *in vitro* and *in vivo* data package, the work on this lead series was stopped despite the progress achieved during the hit-to-lead campaign. Difficulties in reaching sufficient *in vivo* exposure, mostly due to poor solubility and metabolic stability, combined with limited potency and the inability to sterilize chronic infection in animals (and pharmacology at least partially associated with a deprioritized MoA) led to the decision to halt the progression of the series. Since then, we have incorporated routine checks for CYP51 activity, especially when we see a sudden increase in potency within a chemical series. Checking for other deprioritized MoAs is also advisable.

## Chemistry

**Scheme 1:**
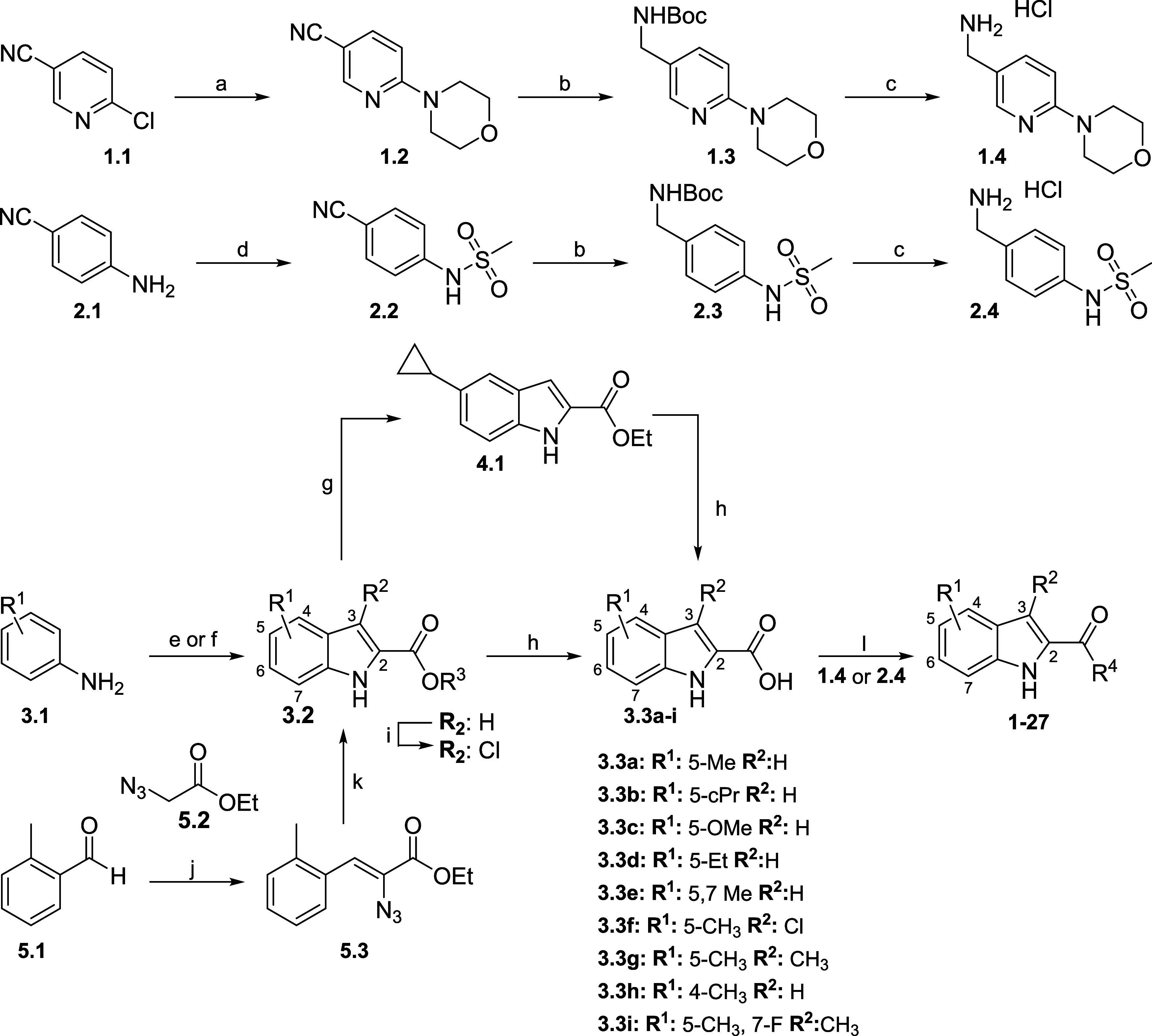
Synthetic Procedures to Assess Functionalized Indole Derivatives Reagents and conditions: (a) morpholine, K_2_CO_3_, MeCN, reflux, 94%; (b) NiCl_2_^.^6H_2_O, NaBH_4_ Boc_2_O, MeOH, 0 °C–r.t., 72–87%; (c) HCl 4 M, dichloromethane (DCM), 0 °C–r.t., 93–97%; (d) MsCl, pyridine, DCM, 0 °C, 98%; (e) Pd(OAc)_2_, dimethyl sulfoxide (DMSO), AcOH, O_2_, pyruvate, 36–82%; (f) NaNO_2_, HCl, ethyl 2-ethyl-3-oxobutanoate, 32%; (g) Pd(OAc)_2_, K_3_PO_4_, cyclopropyl-boronic acid, toluene, 90 °C, 70%; (h) LiOH, H_2_O, EtOH or MeOH; (i) *N*-chlorosuccinimide (NCS), acetone, r.t., 93%; (j) ethyl 2-azidoacetate, EtOH, 0 °C, 61%; (k) toluene, reflux, 24 h, 86%; (l) coupling agent, base, amine (amide coupling conditions are described in the Experimental Section).

**Scheme 2:**
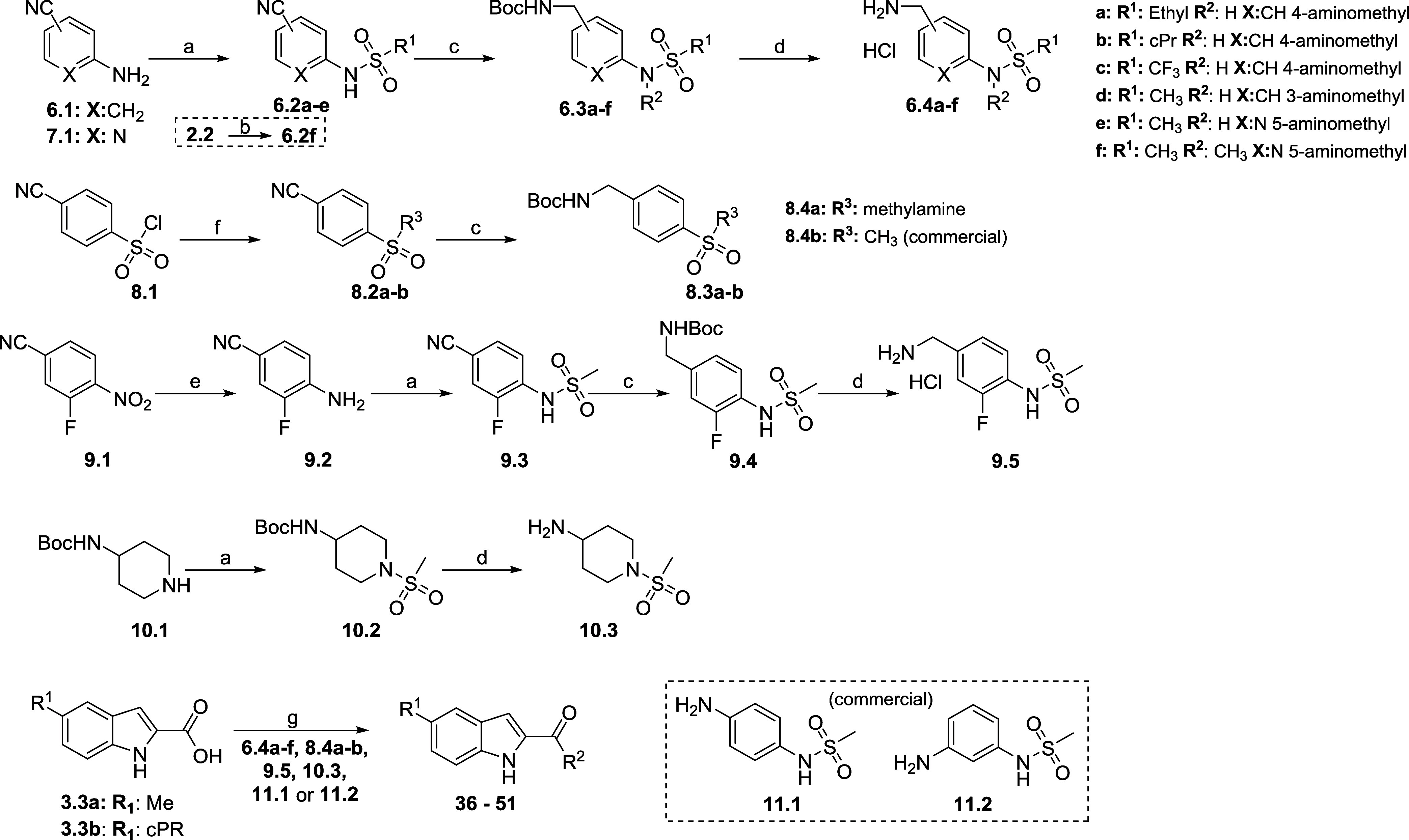
Synthesis of Substituted 4-Phenyl-sulfonamides Reagents and conditions: (a) RSO_2_Cl, pyridine, DCM, 0 °C–r.t., 75–98% or phenyltriflimide, KO*t*Bu, tetrahydrofuran (THF), 0 °C–r.t., 37% (b) MeI, K_2_CO_3_, *N*,*N*-dimethylformamide (DMF), 80 °C, 97% (c) NiCl_2_^.^6H_2_O, NaBH_4_, Boc_2_O, MeOH, 0 °C–r.t., 50–92% (d) HCl 4 M, DCM, 0 °C–r.t., 83–98% (e) H_2_, Pd/C 10%, MeOH, r.t., 76% (f) amine, Et_3_N or *N*,*N*-diisopropylethylamine (DIPEA), 63–90% (g) coupling agent, base, amine (amide coupling conditions are described in the Experimental Section).

**Scheme 3:**
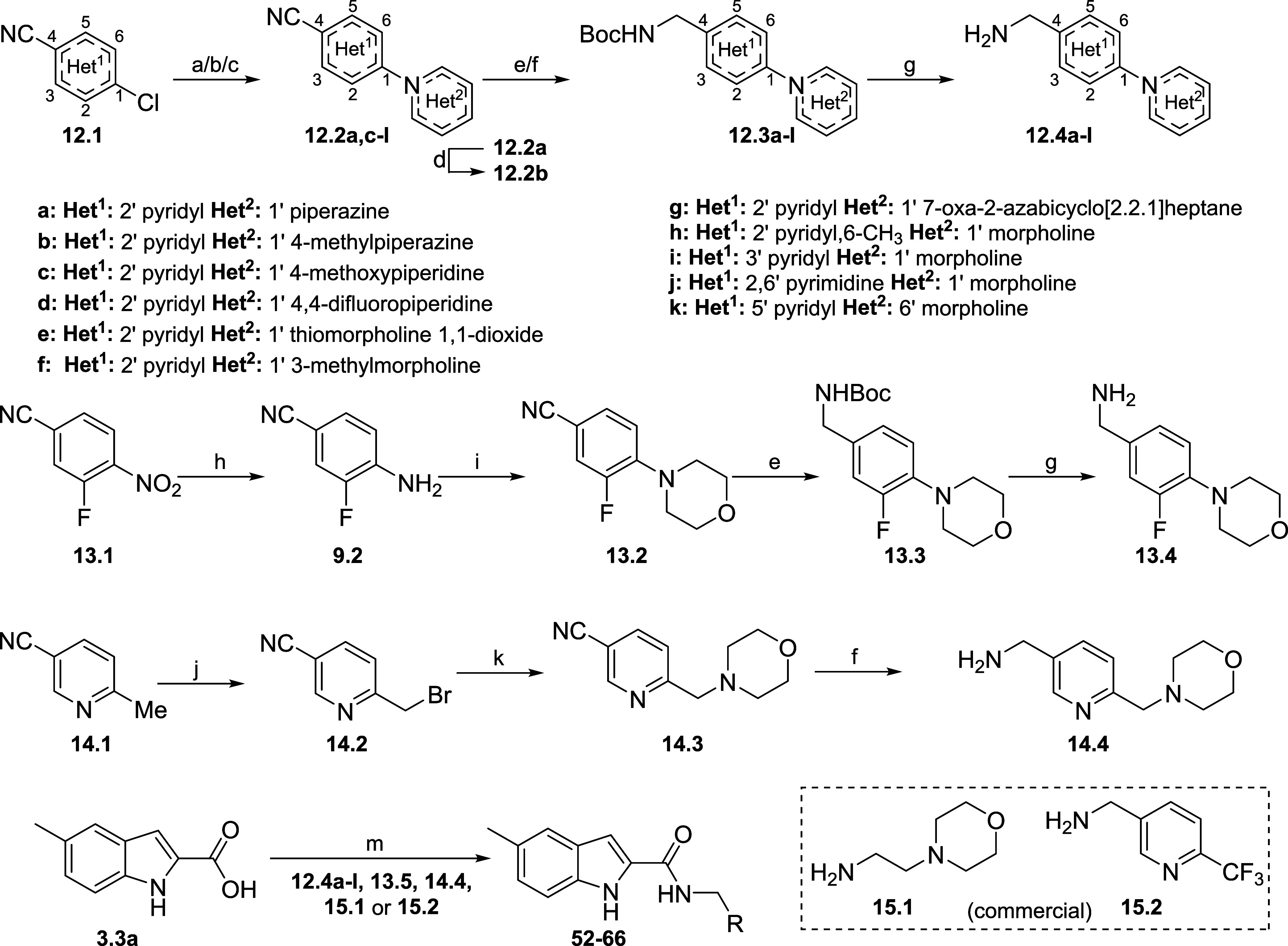
Synthesis of 6-Morpholinopyridin-3-yl Derivatives Reagents and conditions: (a) K_2_CO_3_, MeCN, amine, 16 h, 37–95%; (b) Xphos, K_3_PO_4_, Pd_2_(dba)_3_, toluene, 100 °C, 58%; (c) Xantphos, DMF, toluene, Cs_2_CO_3_, Pd_2_(dba)_3_, 100 °C, 86%; (d) MeI, THF, 0 °C, 97%; (e) NiCl_2^.^_6H_2_O, NaBH_4_, Boc_2_O, MeOH, 0 °C–r.t., 58–86%; (f) Raney-Nickel, NH_4_OH, MeOH, H_2_, r.t., 49–73%; (g) HCl 4 M, DCM, 0 °C–r.t., 85–99%; (h) H_2_, Pd/C 10%, MeOH/EtOAc, 48 h, r.t., 76%; (i) 1-bromo-2-(2-bromoethoxy)ethane, NaH, DMF, 0–80 °C, 98%; (j) NBS, AIBN, CHCl_3_, 80 °C, 48 h 36%; (k) morpholine, K_2_CO_3_, DMF, r.t., 60%; (l) CuI, proline, morpholine, K_2_CO_3_, DMSO, 120 °C, 15 h, 93%; (m) coupling agent, base, amine (amide coupling conditions are described in the Experimental Section).

**Scheme 4:**
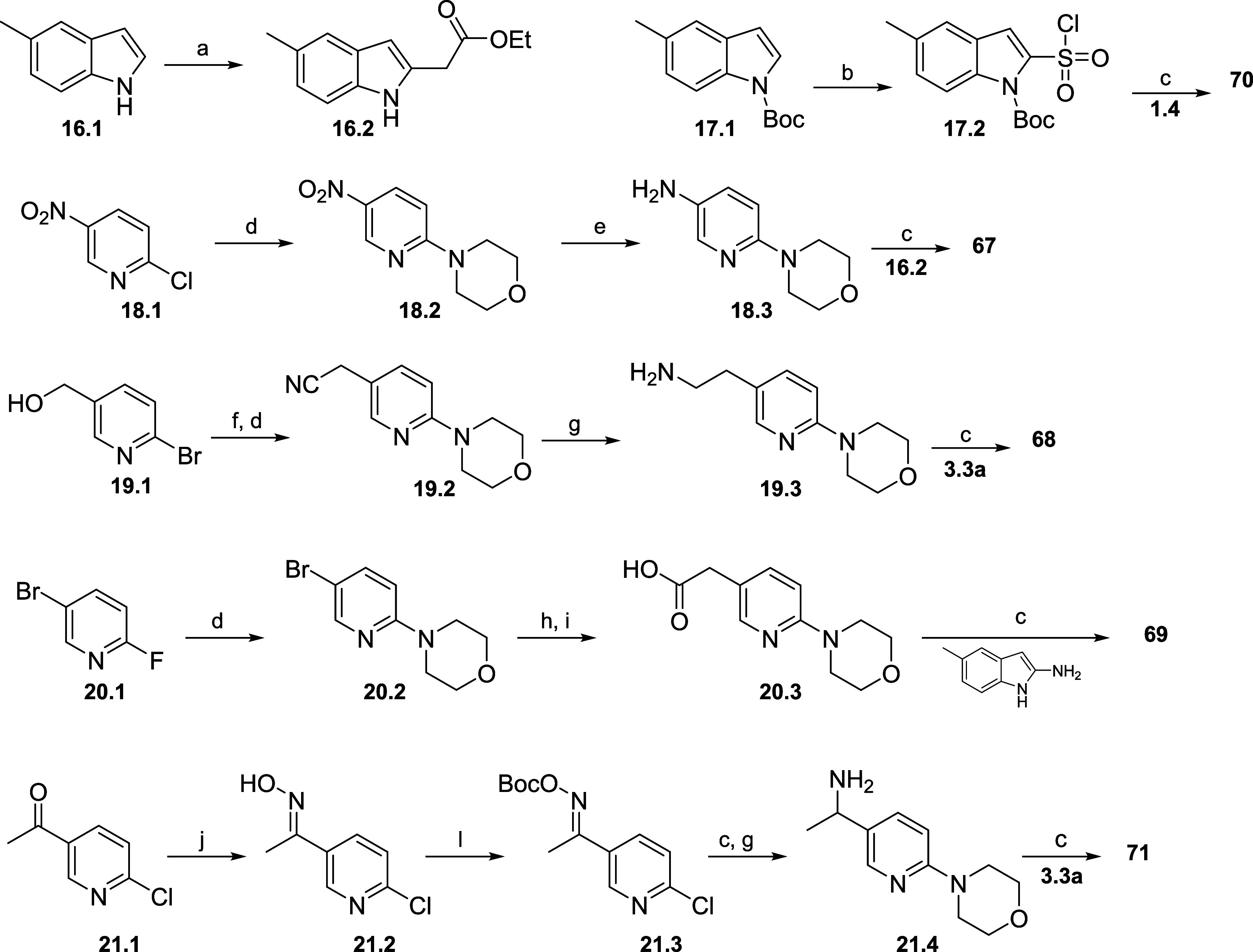
Synthesis of Compounds with Linker Modifications Reagents and conditions: (a) Ethyl bromoacetate, norbornene, Pd(PhCN)_2_Cl_2_, NaHCO_3_, DMF, 70 °C, 25%; (b) *n*-BuLi, THF, −78 °C, SO_2_Cl_2_; (c) coupling agent, base, amine (amide coupling conditions are described in the Experimental Section); (d) K_2_CO_3_, MeCN, morpholine 92% (e) H_2_, Pd/C 10%, EtOAc, r.t., 95% (f) MsCl, DCM, Et_3_N, 1 h, DMSO, NaCN, 6 h, 48% (g) Raney-Nickel, NH_4_OH, MeOH, H_2_, r.t., 10 h; (h) *t*-BuXPhos, K_3_PO_4_, Pd(OAc)_2_, methyl 3-oxobutanoate, 120 °C, 86%; (i) LiOH, EtOH, H_2_O 93%; (j) hydroxylamine, dioxane, 150 °C; (l) Boc_2_O, DCM, Et_3_N, 38%.

**Scheme 5:**
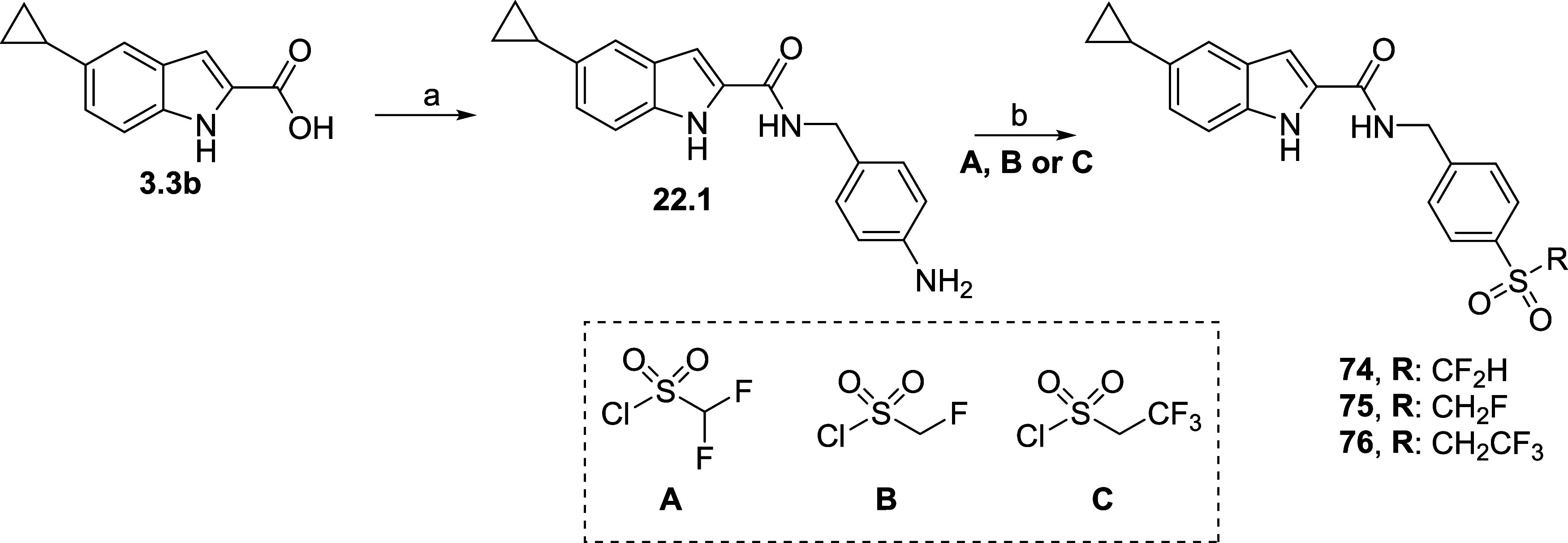
Synthesis of Sulfonamides **74**–**76** Reagents and conditions: (a) HATU, DIPEA, DMF 0 °C–r.t., 4-(aminomethyl)aniline, 16 h, 46%; (b) THF, Et_3_N, 0 °C, (23, 25, or 26), 6 h, 4–30%

**Scheme 6:**
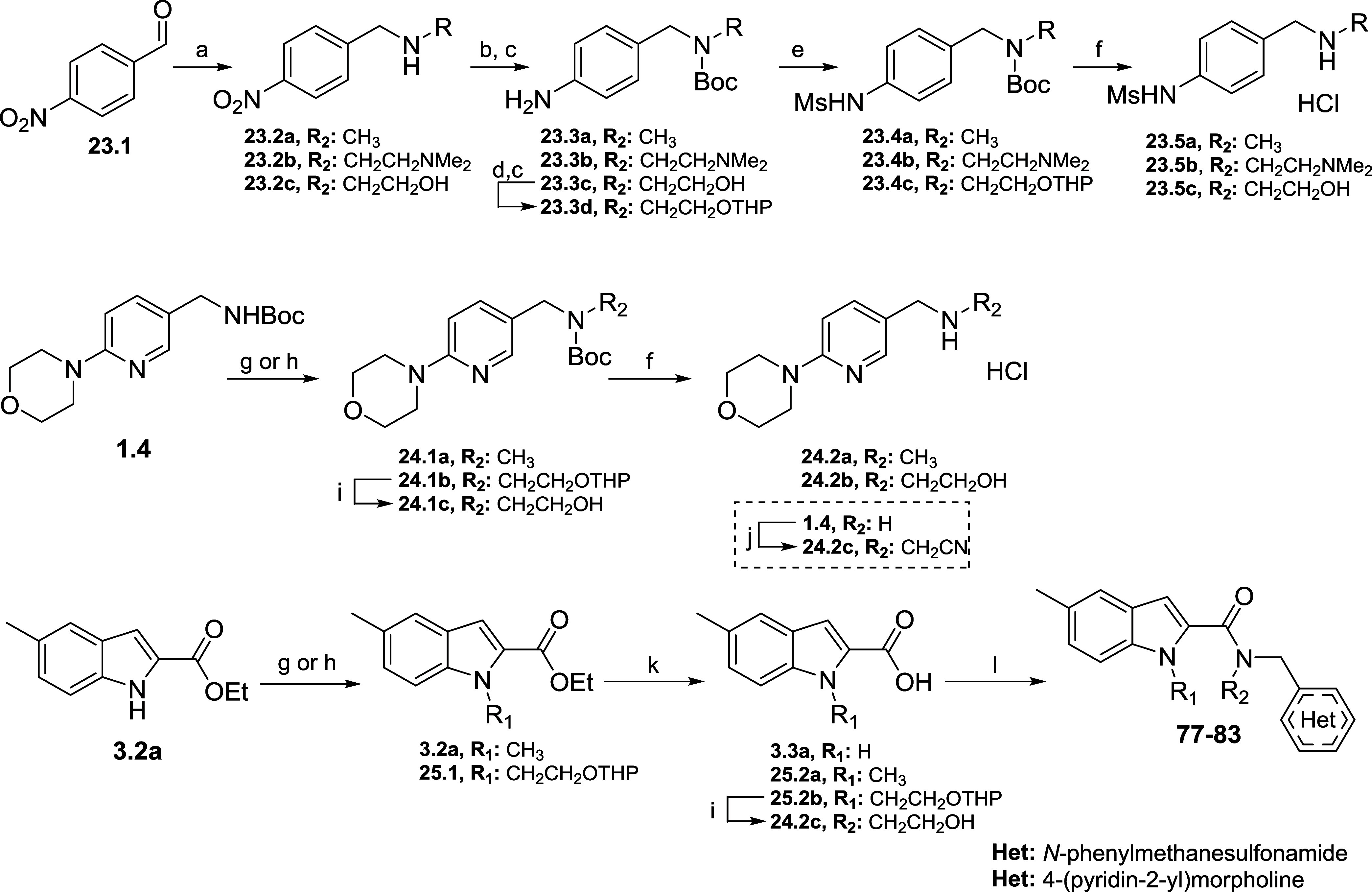
Synthesis of Solubility Improved Analogues Reagents and conditions: (a) Amine, MeOH, NaBH_4_ 0 °C–r.t., 68–96%; (b) Boc_2_O, EtOAc, r.t., 1 h, 51–95%; (c) Pd/C 10%, H_2_ 51%; (d) DHP, DCM, r.t.; (e) MsCl, DCM, pyridine 0 °C–r.t., 95%; (f) HCl 4 M in dioxane, DCM, 0 °C–r.t., 83–93%; (g) MeI, THF, *t*BuOK, 0 °C–r.t., 97%; (h) DMF, NaH, 2-(2-iodoethoxy)tetrahydro-2*H*-pyran, 0 °C–r.t.; (i) MeOH, PTSA, r.t., 48%; (j) DIPEA, MeCN, 2-iodoacetonitrile, r.t., 51%; (k) LiOH, ethanol, H_2_O r.t., 53- 84%; (l) coupling agent, base, amine (amide coupling conditions are described in the Experimental Section).

**Scheme 7:**
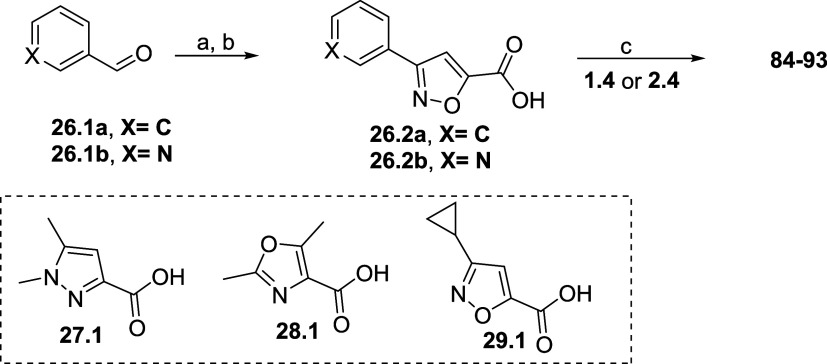
Synthesis of Compounds **84**–**93** Reagents and conditions: (a) HONH_2_·HCl, THF/EtOH/H_2_O 2:5:1, r.t., 30 min, then NCS, THF, r.t., then methyl propiolate, Et_3_N, 60 °C, 16 h; (b) LiOH, ethanol, H_2_O r.t., 53–84%; (c) coupling agent, base, amine (amide coupling conditions are described in the Experimental Section).

All target compounds were prepared by synthetic routes outlined in Schemes 1–7. Experimental details of intermediates are described in the Supporting Information. In general, pyridyl-morpholine compounds (**1.4**) were synthesized via S_N_Ar between 2-chloro-nicotinonitrile and the amine. Nitrile reduction was done using nickel chloride and sodium borohydride^25^ to afford the corresponding *N-*Boc-protected amine and deprotection using HCl solution gave free bases or hydrochloride salts. For the 4-phenyl-sulfonamide compounds (**2.4**), the synthesis started with the sulfonylation of 4-cyano-aniline and followed by nitrile reduction and deprotection, affording phenyl-sulfonamide derivatives. Indole-2-carboxamides were functionalized using different approaches. Modifications at 5′, 6′ or 5′, 7′ positions were accessed by aerobic cross-dehydrogenative coupling of anilines (**3.1**) and ethyl/methyl pyruvates.^26^ Substitutions at the 3′ position were made via the Japp-Klingermann/Fischer-indole synthesis of diazonium salts and substituted β-ketoesters,^27^ and halogenation with *N*-chloro-succinimide.^28^ Suzuki- Miyaura cross-coupling of 5-bromo-indole derivatives decorated the aromatic ring with the cyclopropyl substituent (**4.1**). 4-Substituted indole was synthesized using the Hemetsberger indole synthesis between *o*-tolualdehyde (**5.1**) and ethyl 2-azidoacetate to afford the desired azido acrylate (**5.2**), which would undergo thermolysis to furnish 4-methylindole.^29,30^ Final compounds **1**–**27** were synthesized by amide couplings between indole intermediates **3**.**3a**–**i** and **1**.**4** or **2**.**4** using different coupling agents or by acyl-chloride condensation reactions as listed in the general procedures (Scheme 1).

Final compounds **28**–**35** were synthesized from the correspondent carboxylic acids and amines **1.4** or **2.4** via amide coupling and are described below in the Experimental Procedures. Synthesis of different sulfonamide derivatives were prepared according to Scheme 2. Appropriate sulfonyl chlorides were condensed with 4-cyano-anilines/pyridines **6.1** or **7.1** to afford sulfonamide derivatives **6.2a-e**. Intermediate **2.2** was methylated to give **6.2f**. Nitrile reduction and *N*-Boc deprotection were done using the same conditions as described for Scheme 1, affording amines **6.4a-f**. Reverse sulfonamide **8.4** was obtained from 4-cyanobenzenesulfonyl chloride **8.1** reacting with methylamine to give **8.2**, followed by standard nitrile reduction and *N*-Boc deprotection, giving final amine **8.4a**. Intermediate **8.4b** was obtained from commercial sources. Fluorinated sulfonamide **9.5** was prepared by hydrogenation of 3-fluoro-4-nitrobenzonitrile **9.1** to the corresponding amine **9.2**, followed by sulfonylation, nitrile reduction and *N*-Boc deprotection steps (as described above). Piperidine **10.3** was synthesized using a similar strategy. Final compounds **36**–**51** were synthesized by amide coupling between indole intermediates **3.3a** or **3.3b** and **6.4a-f**, **8.4a-b, 9.5**, **10.3, 11.1**, or **11.2**, using different coupling agents or by acyl-chloride condensation reactions as listed in the general procedures.

Different pyridyl-morpholine derivatives were prepared according to Scheme 3. Analogues **12.4a-l** were synthesized in the same manner as **1.4**: S_N_Ar between **12.1** starting materials and the appropriate amines, followed by nitrile reduction and deprotection, giving free bases or hydrochloride salts. An exception was **12.2a** that was methylated with MeI, generating **12.2b** before nitrile reduction. Intermediate **13.5** was synthesized through a different route: starting with the nitro reduction of 3-fluoro-4-nitrobenzonitrile **13.1** to afford amine **13.2**. Morpholine ring was synthesized *in situ*, using 1-bromo-2-(2-bromoethoxy)ethane in the presence of sodium hydride, followed by standard nitrile reduction and deprotection to give **13.5**. Bromo addition to 6-methylnicotinonitrile **14.1** gave bromide **14.2**, that underwent a displacement reaction with morpholine in the presence of potassium carbonate to give **14.3**, followed by Raney-Nickel promoted nitrile reduction affording final intermediate **14.4**. Final compounds **52**–**66** were synthesized by amide coupling between indole **3.3a** and amines **12.4a-l**, **13.5**, **14.4** or commercial amines **15.1** or **15.2**.

Compounds with different linkers (**67**–**73**) were synthesized according to the syntheses described in Scheme 4. For **67** and **70**, different indole cores **16.2** and **17.2** were synthesized: **16.2** was synthesized from commercially available indole **16.1** via a cross-coupling reaction using ethyl bromoacetate in 25% yield and **17.2** from the Boc-protected indole and sulfuryl chloride. Amine **18.3** was synthesized from commercial nitropyridine **18.1** via our standard S_N_Ar reaction with morpholine (92%) and nitro reduction (92%). Final compound **67** is a product of the amide coupling between **16.2** and **18.3** and final compound **70** was synthesized via the amide coupling between **17.2** and amine **1.4**. For **68**, amine **19.3** was synthesized. Commercial **19.1** was mesylated then reacted with sodium cyanide, followed by the S_N_Ar to give **19.2**. Reduction to the primary amine using Raney-Nickel gave **19.3**. Indole core **3.3a** was then used in the amide coupling reaction to afford **68**. To synthesize reverse sulfonamide **69**, commercial 5-methyl-1*H*-indol-2-amine was coupled with carboxylic acid **20.3**. Pyridine **20.1** was used in our standard S_N_Ar reaction with morpholine to give **20.2**, which was then subjected to a cross-coupling reaction with methyl-3-oxobutanoate, giving **20.3** after ester hydrolysis. Branched final compound **71** was synthesized via amide coupling between **3.3a** and amine **21.5**. Aldehyde **21.1** was first converted in the oxime **21.2**, then *N*-Boc-protected to give **21.2**. Subsequent S_N_Ar and reduction with Raney-Nickel gave amine **21.4**. Methyl-substituted analogues **72** and **73** were synthesized according to the routes shown in Scheme 6. For **72**, amine **24.2a** was synthesized starting from amine **1.4** via methylation with methyl iodide in the presence of potassium *tert*-butoxide followed by Boc-deprotection. Final compound was made via the usual amide coupling. Similarly, for **73**, indole **25.2b** was synthesized and an amide coupling between the carboxylic acid and amine **24.2a** gave the final compound.

Final compounds **74**–**76** were synthesized via the amide coupling between **22.1** and the correspondent sulfonyl chlorides (Scheme 5).

Compounds with different substituents (**77**–**83**) were synthesized according to the syntheses described in Scheme 6. Reductive amination using aldehyde **23.1** and correspondent amines gave secondary amines **23.2a-c**. This was followed by nitro reduction and Boc-protection, giving intermediates **23.3a-c**. Subsequent mesylation and *N*-Boc cleavage afforded final amines **23.4a-c**. Morpholine derivatives **24.2a-d** were synthesized starting from amine **1.4** via substitution with methyl iodide or 2-(2-iodoethoxy)tetrahydro-2*H*-pyran, which was subsequently deprotected using *p*-toluenesulfonic acid. From here, usual Boc-deprotection gave amines **24.2a** and **24.2b**. *N*-Methylene nitrile intermediate **24.2d** was synthesized via substitution of the free amine **24.2c** and 2-iodoacetonitrile. Finally, indole derivatives **25.2a-d** were synthesized using the same strategy: substitution followed by ester hydrolysis and THP deprotection in the case of **25.2d**. Final compounds **77**–**83** were made via the usual amide coupling.

Finally, 5-membered ring analogues **84**–**93** were synthesized according to Scheme 7. Isoxazole-carboxylic acids **26.2a** and **26.2b** were synthesized from the corresponding aldehyde via reaction with hydroxylamine to form the aldoxime, followed by chlorination with *N*-chlorosuccinimide. After consumption of the aldoxime, triethylamine and methyl propiolate were added to form the ester, which was then hydrolyzed to give **26.2a** and **26.2b**. All other 5-membered rings were commercially available. Final compounds were obtained via standard amide coupling with **1.4** or **2.4**.

## Experimental Section

### Chemistry

Reagents purchased were used as received, unless otherwise noted. Dichloromethane (DCM), triethylamine (Et3N), and ethyl formate were distilled from CaH_2_. Tetrahydrofuran (THF) was distilled from sodium/benzophenone. Dimethylformamide (DMF), acetonitrile (MeCN), and 1,4-dioxane were purchased from Aldrich (anhydrous) and used without further purification. Room temperature indicates temperatures in the range of 20–25 °C. Merck silica-aluminum plates were used for thin layer chromatography (TLC), with UV light (254 nm), phosphomolybdic acid, iodine, vanillin, ninhydrin, and potassium permanganate used for visualization. Intermediates and final compounds were purified using silica gel or reverse phase chromatography using the Biotage Isolera, Selekt flash purification systems. Where required, final compounds were purified by preparative reverse phase HPLC (Phenomenex luna C_18_ 100 mm × 40 mm, 3 μm column), with a single wavelength UV–visible detector. LCMS analysis was performed using either a: Waters Alliance reverse phase HPLC (columns Waters SunFire C_18_ 4.6 mm × 50 mm, 3.5 μm, or Waters SunFire C_8_ 4.6 mm × 50 mm, 3.5 μm), using a multiwavelength photodiode array detector from 210 to 600 nm and either a Waters Micromass ZQ detector (electrospray ionization), or Waters Micromass QDA detector; Waters Alliance reverse phase HPLC (2695; Xbridge C_18_ 4.6 mm × 50 mm, 3.5 μm), using a multiwavelength photodiode array detector from 210 to 600 nm and Waters Micromass QDA detector; or Shimadzu LC-20AT, SPD-M20A, column PerkinElmer Brownlee C_18_ (250 mm × 4.6 mm, 5 μm) or (PerkinElmer Brownlee Analytical Phenyl 150 mm × 4.6 mm, 5 μm). All compounds tested had a purity of >95% as measured by LCMS, unless otherwise noted. ^1^H NMR spectra were obtained with Bruker NMR systems, operating at either 250, 400, 500, or 600 MHz at room temperature. Chemical shifts (δ, ppm) are reported relative to the solvent peak (CDCl_3_: 7.26 [1H]; DMSO-*d*_6_: 2.50 [1H]; CD_3_OD: 3.31 [1H]; or Acetone-*d*_6_: 2.05 [1H]). Data for ^1^H NMR spectra are reported as follows: chemical shift (ppm), multiplicity (s for singlet, d for doublet, t for triplet, dd for doublet of doublet, m for multiplet), coupling constant (Hz), and integration. Coupling constants (*J*) are given in Hz and are uncorrected. High-resolution mass spectrometry (HRMS) was measured using electrospray ionization (ESI) (Q-Exactive PlusThermo Fisher Scientific) positive mode from 50 to 750 *m*/*z* and cone tension of 3.5 KV and 50 V SLens. The synthesis of all intermediates and spectral data for final compounds is included in the Supporting Information.

Final compounds are presented below. The synthesis of all intermediates, additional compounds, and spectral data for final compounds are included in the Supporting Information.

### General Procedure A for Amide Coupling

To a solution of appropriate carboxylic acid (1.0 equiv) in DCM (200 mM), 1-ethyl-3-(3-(dimethylamino)propyl)carbodiimide (EDC) (1.2–1.5 equiv), hydroxybenzotriazole (HOBt) (1.2 equiv) and Et_3_N (3.0 equiv) were added. The mixture was stirred at room temperature for 1 h, and then the corresponding free amine or hydrochloride (1.0–1.5 equiv) was added. After the reaction was complete, it was diluted with EtOAc, washed with water, brine, and dried under anhydrous Na_2_SO_4_. The solvent was removed under vacuum and the residue purified by chromatography to provide the desired product.

### General Procedure B for Amide Coupling

To a solution of appropriate carboxylic acid (1.0 equiv) in DMF (200 mM), 2-(1*H*-benzotriazol-1-yl)-1,1,3,3-tetramethyluronium hexafluorophosphate (HBTU) (1.2 equiv), *N*,*N*-diisopropylethylamine (DIPEA) (3.0 equiv) were added. The mixture was stirred at room temperature for 1 h, and then the corresponding free amine or hydrochloride (1.0–1.5 equiv) was added. After the reaction was complete, it was diluted with EtOAc, washed with water, brine, and dried under anhydrous Na_2_SO_4_. The solvent was removed under vacuum and the residue purified by chromatography to provide the desired product.

### General Procedure C for Amide Coupling

To a solution of appropriate carboxylic acid (1.0 equiv) in DCM (200 mM), 1-[bis(dimethylamino)methylene]-1*H*-1,2,3-triazolo[4,5-*b*]pyridinium 3-oxide hexafluorophosphate (HATU) (1.2 equiv), DIPEA (3.0 equiv) were added. The mixture was stirred at room temperature for 1 h, and then the corresponding free amine or hydrochloride (1.2 equiv) was added. After the reaction was complete, it was diluted with EtOAc, washed with water, brine, and dried under anhydrous Na_2_SO_4_. The solvent was removed under vacuum and the residue purified by chromatography to provide the desired product.

### General Procedure D for Amide Coupling

To a solution of appropriate carboxylic acid (1.0 equiv) in thionyl chloride (SOCl_2_) (200 mM), was added cat. DMF. The mixture was stirred at 80 °C for 1 h, the excess thionyl chloride was removed under reduced pressure and the crude acyl chloride was dissolved in dry DCM with Et_3_N (2.0 equiv). The corresponding amine (1.0–1.2 equiv) was added and stirred at 0 °C. After the reaction was complete, it was diluted with EtOAc, washed with sat. NH_4_Cl, brine, and dried under anhydrous Na_2_SO_4_. The solvent was removed under vacuum and the residue purified by chromatography to provide the desired product.

### General Procedure E for Amide Coupling

Step 1: To a cooled solution of boc-amine (1.0 equiv) in DCM (85 mM), HCl (4 M in dioxane, 10.0 equiv) was added dropwise and the reaction left to stir. After the reaction was complete, the solvents were removed *in vacuo*. Step 2: To a solution of appropriate carboxylic acid (1.0 equiv) in DMF (200 mM), DIPEA (3.0 equiv), and HBTU (1.2 equiv) were added. After stirring for 20 min, the previously prepared amine (1.0 equiv) was added and the resulting mixture was stirred at room temperature for 5 h. After the reaction was complete, it was diluted with EtOAc (20 mL), washed with water, brine, and dried under anhydrous Na_2_SO_4_. The solvent was removed under vacuum and the residue purified by chromatography to provide the desired product.

### General Procedure F for Sulfonylation

To a stirred solution of amine derivative (1.0 equiv) in DCM (100 mM) was added Et_3_N (3.0 equiv) at 0 °C. The reaction was stirred for 10 min and the sulfonyl chloride of interest (1.0 equiv) was added at 0 °C, reaction temperature was raised to 25 °C and stirred for 6 h. The reaction mixture was diluted with water and extracted with DCM. The organic layer was dried, concentrated and purified by preparative HPLC or FCC to obtain the desired products.

#### 5-Methyl-*N*-(4-(methylsulfonamido)benzyl)-1*H*-indole-2-carboxamide (**1**)

Compound was synthesized using 5-methyl-1*H*-indole-2-carboxylic acid (190 mg, 1.08 mmol) and *N*-(4-(aminomethyl)phenyl)methanesulfonamide hydrochloride (282 mg, 1.10 mmol) according to General Procedure A. The crude product was purified by FCC (0–5% DCM:MeOH) to yield the title compound as an off-white solid (303 mg, 78%). ^1^H NMR (500 MHz, DMSO-*d*_6_): 11.45 (s, 1H), 9.66 (s, 1H), 8.94 (t, *J* = 6.1 Hz, 1H), 7.38 (sl, 1H), 7.32–7.30 (m, 3H), 7.20–7.16 (m, 2H), 7.07 (d, *J* = 1.3 Hz, 1H), 7.01 (dd, *J* = 8.5, 1.6 Hz, 1H), 4.45 (d, *J* = 6.0 Hz, 2H), 2.95 (s, 3H), 2.36 (s, 3H). ^13^C NMR (126 MHz, DMSO-*d*_6_): 161.1, 137.1, 135.4, 134.9, 131.6, 128.3, 128.3, 127.3, 125.2, 120.8, 120.1, 112.0, 102.1, 41.7, 21.2. ^13^C NMR DEPT-135 (126 MHz, DMSO-*d*_6_): 128.3, 125.2, 120.8, 120.1, 112.0, 102.1, 41.7, 39.1, 21.1. HRMS (ESI): *m*/*z* [M + H]^+^ calcd for C_18_H_19_N_3_O_3_S 358.12199, found 358.12139.

#### 5-Methyl-*N*-((6-morpholinopyridin-3-yl)methyl)-1*H*-indole-2-carboxamide (**2**)

Compound was synthesized using 5-methyl-1*H*-indole-2-carboxylic acid (300 mg, 1.71 mmol) and (6-morpholinopyridin-3-yl)methanamine hydrochloride (433 mg, 1.88 mmol) according to General Procedure A. The crude product was purified by FCC (0–5% DCM:MeOH) to yield the title compound as a white solid (467 mg, 78%). ^1^H NMR (500 MHz, DMSO-*d*_6_): 11.44 (s, 1H), 8.86 (t, *J* = 5.9 Hz, 1H), 8.12 (d, *J* = 2.3 Hz, 1H), 7.55 (dd, *J* = 8.7, 2.5 Hz, 1H), 7.36 (sl, 1H), 7.30 (d, *J* = 8.4 Hz, 1H), 7.03 (d, *J* = 1.3 Hz, 1H), 7.00 (dd, *J* = 8.5, 1.6 Hz, 1H), 6.82 (d, *J* = 8.8 Hz, 1H), 4.36 (d, *J* = 5.9 Hz, 2H), 3.71–3.65 (m, 4H), 3.42–3.36 (m, 4H), 2.35 (s, 3H). ^13^C NMR (126 MHz, DMSO-*d*_6_): 161.1, 158.6, 146.8, 137.5, 134.9, 131.6, 128.2, 127.3, 125.1, 124.5, 120.7, 112.0, 106.9, 102.0, 65.9, 45.4, 21.1. ^13^C NMR DEPT-135 (126 MHz, DMSO-*d*_6_): 146.8, 137.5, 125.1, 120.7, 112.0, 106.9, 102.0, 65.9, 45.4, 39.5, 21.1. HRMS (ESI): *m*/*z* [M + H]^+^ calcd for C_20_H_23_N_4_O_2_ 351.18155, found 351.18121.

#### 5-Cyclopropyl-*N*-(4-(methylsulfonamido)benzyl)-1*H*-indole-2-carboxamide (**3**)

Compound was synthesized using 5-cyclopropyl-1*H*-indole-2-carboxylic acid (72 mg, 0.30 mmol) and *N*-(4-(aminomethyl)phenyl)methanesulfonamide hydrochloride (85 mg, 0.36 mmol) according to General Procedure C. The crude product was purified by FCC (0–5% DCM:MeOH) to yield the title compound as a white solid (64 mg, 56%). ^1^H NMR (400 MHz, DMSO-*d*_6_): 11.43 (s, 1H), 9.66 (s, 1H), 8.94 (t, *J* = 6.0 Hz, 1H), 7.33–7.27 (m, 4H), 7.22–7.14 (m, 2H), 7.06 (dd, *J* = 2.2, 0.9 Hz, 1H), 6.92 (dd, *J* = 8.7, 1.6 Hz, 1H), 4.45 (d, *J* = 6.0 Hz, 2H), 2.95 (s, 3H), 2.02–1.90 (m, 1H), 0.95–0.83 (m, 2H), 0.68–0.60 (m, 2H). ^13^C NMR (101 MHz, DMSO-*d*_6_): 161.1, 137.0, 135.3, 135.0, 134.5, 131.7, 128.2, 127.2, 122.1, 120.1, 117.6, 112.1, 102.1, 41.7, 15.2, 8.7. HRMS (ESI): *m*/*z* [M + H]^+^ calcd for C_20_H_22_N_3_O_3_S 384.13764, found 384.13726.

#### 5-Cyclopropyl-*N*-((6-morpholinopyridin-3-yl)methyl)-1*H*-indole-2-carboxamide (**4**)

Compound was synthesized using 5-cyclopropyl-1*H*-indole-2-carboxylic acid (86 mg, 0.47 mmol) and (6-morpholinopyridin-3-yl)methanamine hydrochloride (108 mg, 0.47 mmol) according to General Procedure C. The crude product was purified by FCC (0–5% DCM:MeOH) to yield the title compound as a white solid (57 mg, 50%). ^1^H NMR (250 MHz, DMSO-*d*_6_): δ 11.43 (s, 1H), 8.87 (t, *J* = 5.9 Hz, 1H), 8.13 (d, *J* = 2.4 Hz, 1H), 7.56 (dd, *J* = 8.7, 2.4 Hz, 1H), 7.36–7.22 (m, 2H), 7.02 (s, 1H), 6.92 (dd, *J* = 8.6, 1.6 Hz, 1H), 6.83 (d, *J* = 8.7 Hz, 1H), 4.37 (d, *J* = 5.8 Hz, 2H), 3.75–3.63 (m, 4H), 3.45–3.36 (m, 4H), 2.0–1.15 (m, 1H). 0.97–0.83 (m, 2H), 0.71–0.57 (m, 2H). HRMS (ESI): *m*/*z* [M + H]^+^ calcd for C_22_H_25_N_4_O_2_ 377.19720, found 377.19696.

#### 5-Ethyl-*N*-(4-(methylsulfonamido)benzyl)-1*H*-indole-2-carboxamide (**5**)

Compound was synthesized using 5-ethyl-1*H*-indole-2-carboxylic acid (140 mg, 0.74 mmol) and *N*-(4-(aminomethyl)phenyl)methanesulfonamide hydrochloride (180 mg, 0.75 mmol) according to General Procedure C. The crude product was purified by FCC (0–5% DCM:MeOH) to yield the title compound as a white solid (169 mg, 62%). ^1^H NMR (500 MHz, DMSO-*d*_6_) d 11.44 (s, 1H), 9.66 (s, 1H), 8.94 (t, *J* = 5.97 Hz, 1H), 7.39 (s, 1H), 7.27–7.36 (m, 3H), 7.18 (d, *J* = 8.49 Hz, 2H), 7.08 (d, *J* = 1.41 Hz, 1H), 7.05 (dd, *J* = 1.41, 8.49 Hz, 1H), 4.46 (d, *J* = 5.97 Hz, 2H), 2.95 (s, 3H), 2.66 (q, *J* = 7.55 Hz, 2H), 1.21 (t, *J* = 7.55 Hz, 3H). HRMS (ESI): *m*/*z* [M + H]^+^ calcd for C_19_H_22_N_3_O_3_S 372.13764, found 372.13739.

#### 5-Methoxy-*N*-(4-(methylsulfonamido)benzyl)-1*H*-indole-2-carboxamide (**6**)

Compound was synthesized using 5-methoxy-1*H*-indole-2-carboxylic acid (70 mg, 0.4 mmol) and *N*-(4-(aminomethyl)phenyl)methanesulfonamide hydrochloride (91 mg, 0.4 mmol) according to General Procedure C. The crude product was purified by FCC (0–5% DCM:MeOH) to yield the title compound as a white solid (100 mg, 67%). ^1^H NMR (500 MHz, DMSO-*d*_6_): δ 11.43 (s, 3H), 9.66 (s, 1H), 8.94 (t, *J* = 6.05 Hz, 1H), 7.27–7.34 (m, 3H), 7.15–7.21 (m, 2H), 7.07–7.06 (m, 2H), 6.83 (dd, *J* = 2.44, 8.88 Hz, 1H), 4.46 (d, *J* = 5.97 Hz, 2H), 3.75 (s, 3H), 2.95 (s, 3H). HRMS (ESI): *m*/*z* [M + H]^+^ calcd for C_18_H_20_N_3_O_4_S 374.11690, found 374.11664.

#### 5-Methoxy-*N*-((6-morpholinopyridin-3-yl)methyl)-1*H*-indole-2-carboxamide (**7**)

Compound was synthesized using 5-methoxy-1*H*-indole-2-carboxylic acid (120 mg, 0.63 mmol) and (6-morpholinopyridin-3-yl)methanamine hydrochloride (150 mg, 0.65 mmol) according to General Procedure C. The crude product was purified by FCC (0–5% DCM:MeOH) to yield the title compound as a white solid (130 mg, 56%). ^1^H NMR (500 MHz, DMSO-*d*_6_) d 11.41 (s, 1H), 8.86 (t, *J* = 5.82 Hz, 1H), 8.13 (d, *J* = 2.04 Hz, 1H), 7.55 (dd, *J* = 2.28, 8.72 Hz, 1H), 7.30 (d, *J* = 8.80 Hz, 1H), 7.05 (dd, *J* = 1.65, 15.33 Hz, 2H), 6.79–6.86 (m, 2H), 4.36 (d, *J* = 5.82 Hz, 2H), 3.64–3.71 (m, 4H), 3.36–3.42 (m, 4H). HRMS (ESI): *m*/*z* [M + H]^+^ calcd for C_20_H_23_N_4_O_3_ 367.17647, found 367.17604.

#### 5-Fluoro-*N*-(4-(methylsulfonamido)benzyl)-1*H*-indole-2-carboxamide (**8**)

Compound was synthesized using 5-fluoro-1*H*-indole-2-carboxylic acid (54 mg, 0.30 mmol) and *N*-(4-(aminomethyl)phenyl)methanesulfonamide hydrochloride (85 mg, 0.36 mmol) according to General Procedure C. The crude product was purified by FCC (0–5% DCM:MeOH) to yield the title compound as a white solid (54 mg, 50%). ^1^H NMR (500 MHz, DMSO-*d*_6_): δ 11.70 (s, 1H), 9.66 (s, 1H), 9.04 (t, *J* = 6.0 Hz, 1H), 7.45–7.36 (m, 2H), 7.33–7.27 (m, 2H), 7.20–7.13 (m, 3H), 7.04 (td, *J* = 9.2, 2.6 Hz, 1H), 4.46 (d, *J* = 6.0 Hz, 2H), 2.95 (s, 3H). HRMS (ESI): *m*/*z* [M + H]^+^ calcd for C_17_H_17_FN_3_O_3_S 362.09692, found 362.654.

#### 5-Chloro-*N*-(4-(methylsulfonamido)benzyl)-1*H*-indole-2-carboxamide (**9**)

Compound was synthesized using 5-chloro-1*H*-indole-2-carboxylic acid (59 mg, 0.30 mmol) and *N*-(4-(aminomethyl)phenyl)methanesulfonamide hydrochloride (85 mg, 0.36 mmol) according to General Procedure C. The crude product was purified by FCC (0–5% DCM:MeOH) to yield the title compound as a white solid (54 mg, 48%). ^1^H NMR (250 MHz, DMSO-*d*_6_): δ 11.80 (s, 1H), 9.67 (s, 1H), 9.08 (t, *J* = 6.0 Hz, 1H), 7.70 (d, *J* = 2.1 Hz, 1H), 7.43 (d, *J* = 8.8 Hz, 1H), 7.30 (d, *J* = 8.4 Hz, 2H), 7.18 (dd, *J* = 8.8, 2.5 Hz, 4H), 4.46 (d, *J* = 5.9 Hz, 2H), 2.94 (s, 3H). HRMS (ESI): *m*/*z* [M + H]^+^ calcd for C_17_H_17_ClN_3_O_3_S 378.06737, found 378.06691.

#### *N*-(4-(Methylsulfonamido)benzyl)-5-(trifluoromethyl)-1*H*-indole-2-carboxamide (**10**)

Compound was synthesized using 5-(trifluoromethyl)-1*H*-indole-2-carboxylic acid (69 mg, 0.30 mmol) and N-(4-(aminomethyl)phenyl)methanesulfonamide hydrochloride (85 mg, 0.36 mmol) according to General Procedure C. The crude product was purified by FCC (0–5% DCM:MeOH) to yield the title compound as a white solid (71 mg, 58%). ^1^H NMR (250 MHz, DMSO-*d*_6_): δ 12.06 (s, 1H), 9.67 (s, 1H), 9.17 (t, *J* = 5.9 Hz, 1H), 8.07 (s, 1H), 7.60 (d, *J* = 8.7 Hz, 1H), 7.46 (dd, *J* = 8.8, 1.8 Hz, 1H), 7.31 (d, *J* = 8.3 Hz, 3H), 7.18 (d, *J* = 8.5 Hz, 2H), 4.47 (d, *J* = 5.8 Hz, 2H), 2.95 (s, 3H). HRMS (ESI): *m*/*z* [M + H]^+^ calcd for C_18_H_17_F_3_N_3_O_3_S 412.09372, found 412.09337.

#### *N*-(4-(Methylsulfonamido)benzyl)-5-(methylsulfonyl)-1*H*-indole-2-carboxamide (**11**)

Compound was synthesized using 5-(methylsulfonyl)-1*H*-indole-2-carboxylic acid (100 mg, 0.41 mmol) and *N*-(4-(aminomethyl)phenyl)methanesulfonamide hydrochloride (109 mg, 1.10 mmol) according to General Procedure A. The crude product was purified by FCC (0–5% DCM:MeOH) to yield the title compound as a white solid (91 mg, 51%). ^1^H NMR (250 MHz, DMSO-*d*_6_): δ 12.18 (s, 1H), 9.68 (s, 1H), 9.21 (t, *J* = 6.0 Hz, 1H), 8.27 (d, *J* = 1.7 Hz, 1H), 7.70 (dd, *J* = 8.7, 1.7 Hz, 1H), 7.62 (d, *J* = 8.7 Hz, 1H), 7.42–7.36 (m, 1H), 7.32 (d, *J* = 8.6 Hz, 2H), 7.28–7.12 (m, 2H), 4.48 (d, *J* = 5.9 Hz, 2H), 3.18 (s, 3H), 2.95 (s, 3H). HRMS (ESI): *m*/*z* [M + H]^+^ calcd for C_18_H_20_N_3_O_5_S_2_ 422.08389, found 422.08351.

#### 5-(Methylsulfonyl)-*N*-((6-morpholinopyridin-3-yl)methyl)-1*H*-indole-2-carboxamide (**12**)

Compound was synthesized using 5-(methylsulfonyl)-1*H*-indole-2-carboxylic acid (100 mg, 0.41 mmol) and (6-morpholinopyridin-3-yl)methanamine hydrochloride (106 mg, 1.10 mmol) according to General Procedure A. The crude product was purified by FCC (0–5% DCM:MeOH) to yield the title compound as a white solid (99 mg, 57%). ^1^H NMR (250 MHz, DMSO-*d*_6_): δ 12.17 (s, 1H), 9.13 (t, *J* = 5.8 Hz, 1H), 8.27 (d, *J* = 1.7 Hz, 1H), 8.14 (d, *J* = 2.4 Hz, 1H), 7.70 (dd, *J* = 8.7, 1.8 Hz, 1H), 7.65–7.52 (m, 2H), 7.35 (d, *J* = 2.1 Hz, 1H), 6.83 (d, *J* = 8.8 Hz, 1H), 4.39 (d, *J* = 5.8 Hz, 2H), 3.75–3.63 (m, 4H), 3.45–3.36 (m, 4H), 3.17 (s, 3H). HRMS (ESI): *m*/*z* [M + H]^+^ calcd for C_20_H_23_N_4_O_4_S 415.14345, found 415.14321.

#### 5-(Methylsulfonamido)-*N*-((6-morpholinopyridin-3-yl)methyl)-1*H*-indole-2-carboxamide (**13**)

Compound was synthesized using 5-(methylsulfonamido)-1*H*-indole-2-carboxylic acid (90 mg, 0.35 mmol) and *N*-(4-(aminomethyl)phenyl)methanesulfonamide hydrochloride (89.5 mg, 0.39 mmol) according to General Procedure A. The crude product was purified by FCC (0–5% DCM:MeOH) to yield the title compound as a white solid (90 mg, 59%). ^1^H NMR (250 MHz, DMSO-*d*_6_): δ 11.62 (s, 1H), 9.32 (s, 1H), 8.13 (d, *J* = 2.4 Hz, 1H), 7.63–7.33 (m, 3H), 7.17–7.02 (m, 2H), 6.82 (d, *J* = 8.7 Hz, 1H), 4.37 (s, 2H), 3.74–3.63 (m, 4H), 3.46–3.36 (m, 4H), 2.87 (s, 3H). HRMS (ESI): *m*/*z* [M + H]^+^ calcd for C_20_H_24_N_5_O_4_S 430.15435, found 430.15417.

#### 4-Methyl-*N*-(4-(methylsulfonamido)benzyl)-1*H*-indole-2-carboxamide (**14**)

Compound was synthesized using 4-methyl-1*H*-indole-2-carboxylic acid (80 mg, 0.45 mmol) and *N*-(4-(aminomethyl)phenyl)methanesulfonamide hydrochloride (141 mg, 0.59 mmol) according to General Procedure C. The crude product was purified by FCC (0–5% DCM:MeOH) to yield the title compound as a white solid (133 mg, 81%). ^1^H NMR (250 MHz, DMSO-*d*_6_) δ: 11.55 (s, 1H), 9.65 (s, 1H), 8.97 (s, 1H), 7.47–6.74 (m, 8H), 4.45 (d, *J* = 6.0 Hz, 2H), 2.95 (s, 3H), 2.47 (s, 3H). HRMS (ESI): *m*/*z* [M + H]^+^ calcd for C_18_H_20_N_3_O_3_S 358.12199, found 358.12138.

#### 6-Methyl-*N*-(4-(methylsulfonamido)benzyl)-1*H*-indole-2-carboxamide (**15**)

Compound was synthesized using 6-methyl-1*H*-indole-2-carboxylic acid (150 mg, 0.86 mmol) and *N*-(4-(aminomethyl)phenyl)methanesulfonamide hydrochloride (230 mg, 1.0 mmol) according to General Procedure C. The crude product was purified by FCC (0–5% DCM:MeOH) to yield the title compound as a white solid (152 mg, 50%). ^1^H NMR (500 MHz, Acetone-*d*_6_): δ 10.64 (br. s., 1H), 8.52 (br. s., 1H), 8.22 (br. s., 1H), 7.48 (d, *J* = 8.02 Hz, 1H), 7.41–7.36 (m, 2H), 7.34 (s, 1H), 7.27–7.32 (m, 2H), 7.08–7.06 (m, 1H), 6.91 (dd, *J* = 0.94, 8.17 Hz, 1H), 4.59 (d, *J* = 6.13 Hz, 2H), 2.96 (s, 3H), 2.41 (s, 3H). HRMS (ESI): *m*/*z* [M + H]^+^ calcd for C_18_H_20_N_3_O_3_S 358.12199, found 358.12174.

#### 7-Methyl-*N*-(4-(methylsulfonamido)benzyl)-1*H*-indole-2-carboxamide (**16**)

Compound was synthesized using 7-methyl-1*H*-indole-2-carboxylic acid (120 mg, 0.68 mmol) and *N*-(4-(aminomethyl)phenyl)methanesulfonamide hydrochloride (160 mg, 0.68 mmol) according to General Procedure C. The crude product was purified by FCC (0–5% DCM:MeOH) to yield the title compound as a white solid (136 mg, 56%). ^1^H NMR (500 MHz, DMSO-*d*_6_): δ 11.34 (s, 1H), 9.66 (s, 1H), 8.93 (t, *J* = 5.97 Hz, 1H), 7.43 (d, *J* = 7.55 Hz, 1H), 7.32 (d, *J* = 8.49 Hz, 2H), 7.17–7.21 (m, 2H), 7.15 (d, *J* = 2.04 Hz, 1H), 6.92–7.00 (m, 2H), 4.48 (d, *J* = 5.97 Hz, 2H), 2.95 (s, 3H), (s, 3H, CH_3_ overlapped with DMSO). HRMS (ESI): *m*/*z* [M + H]^+^ calcd for C_18_H_20_N_3_O_3_S 358.12199, found 358.12178.

#### 5,7-Dimethyl-*N*-(4-(methylsulfonamido)benzyl)-1*H*-indole-2-carboxamide (**17**)

Compound was synthesized using 5,7-dimethyl-1*H*-indole-2-carboxylic acid (100 mg, 0.53 mmol) and *N*-(4-(aminomethyl)phenyl)methanesulfonamide hydrochloride (160 mg, 1.3 mmol) according to General Procedure C. The crude product was purified by FCC (0–5% DCM:MeOH) to yield the title compound as a white solid (64 mg, 32%). ^1^H NMR (250 MHz, DMSO-*d*_6_): δ 11.22 (s, 1H), 9.66 (s, 1H), 8.88 (t, *J* = 6.0 Hz, 1H), 7.33 (s, 1H), 7.23–7.12 (m, 3H), 7.05 (d, *J* = 2.0 Hz, 1H), 6.81 (s, 1H), 4.46 (d, *J* = 5.9 Hz, 2H), 2.95 (s, 3H), 2.46 (s, 3H), 2.32 (s, 3H). HRMS (ESI): *m*/*z* [M + H]^+^ calcd for C_19_H_22_N_3_O_3_S 372.13764, found 372.13732.

#### 3-Chloro-5-methyl-*N*-(4-(methylsulfonamido)benzyl)-1*H*-indole-2-carboxamide (**18**)

Compound was synthesized using 3-chloro-5-methyl-1*H*-indole-2-carboxylic acid (100 mg, 0.47 mmol) and *N*-(4-(aminomethyl)phenyl)methanesulfonamide hydrochloride (111.25 mg, 0.47 mmol) according to General Procedure C. The crude product was purified by FCC (0–5% DCM:MeOH) to yield the title compound as a white solid (17 mg, 11%). ^1^H NMR (250 MHz, DMSO-*d*_6_): δ 11.80 (s, 1H), 9.69 (s, 1H), 8.40 (t, *J* = 6.0 Hz, 1H), 7.40–7.30 (m, 4H), 7.24–7.07 (m, 3H), 4.51 (d, *J* = 5.9 Hz, 2H), 2.96 (s, 3H), 2.41 (s, 3H). HRMS (ESI): *m*/*z* [M + H]^+^ calcd for C_18_H_19_ClN_3_O_3_S 392.08302, found 392.08233.

#### 3,5-Dimethyl-*N*-(4-(methylsulfonamido)benzyl)-1*H*-indole-2-carboxamide (**19**)

Compound was synthesized using 3,5-dimethyl-1*H*-indole-2-carboxylic acid (80 mg, 0.42 mmol) and *N*-(4-(aminomethyl)phenyl)methanesulfonamide hydrochloride (130 mg, 0.55 mmol) according to General Procedure C. The crude product was purified by FCC (0–5% DCM:MeOH) to yield the title compound as a white solid (114 mg, 73%). ^1^H NMR (500 MHz, DMSO-*d*_6_) δ: 11.02 (s, 1H), 9.67 (s, 1H), 8.30 (t, *J* = 5.9 Hz, 1H), 7.38–7.32 (m, 3H), 7.26 (d, *J* = 8.3 Hz, 1H), 7.22–7.17 (m, 2H), 7.03 (dd, *J* = 8.3, 1.6 Hz, 1H), 4.46 (d, *J* = 5.8 Hz, 2H), 2.96 (s, 3H), 2.48 (s, 3H), 2.38 (s, 3H). HRMS (ESI): *m*/*z* [M + H]^+^ calcd for C_19_H_22_N_3_O_3_S 372.13764, found 372.13702.

#### 7-Fluoro-5-methyl-*N*-(4-(methylsulfonamido)benzyl)-1*H*-indole-2-carboxamide (**20**)

Compound was synthesized using 7-fluoro-5-methyl-1*H*-indole-2-carboxylic acid (90 mg, 0.46 mmol) and *N*-(4-(aminomethyl)phenyl)methanesulfonamide hydrochloride (143 mg, 0.60 mmol) according to General Procedure C. The crude product was purified by FCC (0–5% DCM:MeOH) to yield the title compound as a white solid (75 mg, 43%). ^1^H NMR (250 MHz, DMSO-*d*_6_): δ 11.50 (s, 1H), 9.67 (s, 1H), 8.98 (t, *J* = 6.0 Hz, 1H), 7.32 (d, *J* = 8.4 Hz, 2H), 7.26–7.11 (m, 4H), 6.93–6.83 (m, 1H), 4.47 (d, *J* = 6.0 Hz, 2H), 2.96 (s, 3H), (indole CH_3_ overlapped with DMSO). HRMS (ESI): *m*/*z* [M + H]^+^ calcd for C_18_H_19_FN_3_O_3_S 376.11257, found 376.11210.

#### 4-Methyl-*N*-((6-morpholinopyridin-3-yl)methyl)-1*H*-indole-2-carboxamide (**21**)

Compound was synthesized using 4-methyl-1*H*-indole-2-carboxylic acid (40 mg, 0.22 mmol) and (6-morpholinopyridin-3-yl)methanamine hydrochloride (50 mg, 0.22 mmol) according to General Procedure C. The crude product was purified by FCC (0–5% DCM:MeOH) to yield the title compound as a white solid (36 mg, 46%). ^1^H NMR (250 MHz, DMSO-*d*_6_): δ 11.57–11.50 (m, 1H), 8.89 (t, *J* = 5.9 Hz, 1H), 8.13 (d, *J* = 2.4 Hz, 1H), 7.56 (dd, *J* = 8.7, 2.4 Hz, 1H), 7.31–7.15 (m, 2H), 7.06 (t, *J* = 7.6 Hz, 1H), 6.82 (d, *J* = 7.9 Hz, 2H), 4.37 (d, *J* = 5.8 Hz, 2H), 3.68 (t, *J* = 4.9 Hz, 4H), 3.39 (t, *J* = 4.9 Hz, 4H), 2.47 (s, 3H). HRMS (ESI): *m*/*z* [M + H]^+^ calcd for C_20_H_23_N_4_O_2_ 351.18155, found 351.18111.

#### 6-Methyl-*N*-((6-morpholinopyridin-3-yl)methyl)-1*H*-indole-2-carboxamide (**22**)

Compound was synthesized using 6-methyl-1*H*-indole-2-carboxylic acid (150 mg, 0.85 mmol) and (6-morpholinopyridin-3-yl)methanamine hydrochloride (229 mg, 1.0 mmol) according to General Procedure C. The crude product was purified by FCC (0–5% DCM:MeOH) to yield the title compound as a white solid (110 mg, 37%). ^1^H NMR (500 MHz, CDCl_3_): δ 9.20 (br. s., 1H), 8.20 (d, *J* = 1.89 Hz, 1H), 7.57 (dd, *J* = 2.36, 8.80 Hz, 1H), 7.51 (d, *J* = 8.17 Hz, 1H), 7.22 (s, 1H), 6.98 (d, *J* = 8.17 Hz, 1H), 6.77 (d, *J* = 1.26 Hz, 1H), 6.63 (d, *J* = 8.80 Hz, 1H), 6.40 (br. s., 1H), 4.55 (d, *J* = 5.82 Hz, 2H), 3.79–3.85 (m, 4H), 3.47–3.53 (m, 4H), 2.47 (s, 3H). HRMS (ESI): *m*/*z* [M + H]^+^ calcd for C_20_H_23_N_4_O_2_ 351.18155, found 351.18134.

#### 7-Methyl-*N*-((6-morpholinopyridin-3-yl)methyl)-1*H*-indole-2-carboxamide (**23**)

Compound was synthesized using 7-methyl-1*H*-indole-2-carboxylic acid (149 mg, 0.85 mmol) and (6-morpholinopyridin-3-yl)methanamine hydrochloride (195 mg, 0.85 mmol) according to General Procedure C. The crude product was purified by FCC (0–5% CHCl_3_:MeOH) to yield the title compound as a white solid (110 mg, 36%). ^1^H NMR (250 MHz, CDCl_3_): δ 9.18 (br. s., 1H), 8.21 (d, *J* = 2.21 Hz, 1H), 7.57 (dd, *J* = 2.44, 8.77 Hz, 1H), 7.43–7.52 (m, 1H), 7.01–7.14 (m, 2H), 6.82 (d, *J* = 2.14 Hz, 1H), 6.64 (d, *J* = 8.70 Hz, 1H), 6.41 (t, *J* = 5.30 Hz, 1H), 4.56 (d, *J* = 5.72 Hz, 2H), 3.76–3.89 (m, 4H), 3.44–3.56 (m, 4H), 2.52 (s, 3H). HRMS (ESI): *m*/*z* [M + H]^+^ calcd for C_20_H_23_N_4_O_2_ 351.18155, found 351.18134.

#### 5,7-Dimethyl-*N*-((6-morpholinopyridin-3-yl)methyl)-1*H*-indole-2-carboxamide (**24**)

Compound was synthesized using 5,7-dimethyl-1*H*-indole-2-carboxylic acid (100 mg, 0.53 mmol) and (6-morpholinopyridin-3-yl)methanamine hydrochloride (160 mg, 0.70 mmol) according to General Procedure C. The crude product was purified by FCC (0–5% DCM:MeOH) to yield the title compound as a white solid (94 mg, 49%).1H NMR (250 MHz, DMSO-*d*_6_): δ 11.21 (s, 1H), 8.81 (t, *J* = 5.8 Hz, 1H), 8.14 (d, *J* = 2.4 Hz, 1H), 7.56 (dd, *J* = 8.7, 2.5 Hz, 1H), 7.19 (s, 1H), 7.01 (d, *J* = 2.0 Hz, 1H), 6.87–6.77 (m, 2H), 4.37 (d, *J* = 5.7 Hz, 2H), 3.74–3.63 (m, 4H), 3.45–3.34 (m, 4H), 2.45 (s, 3H), 2.32 (s, 3H). HRMS (ESI): *m*/*z* [M + H]^+^ calcd for C_21_H_25_N_4_O_2_ 365.19720, found 365.19695.

#### 3-Chloro-5-methyl-*N*-((6-morpholinopyridin-3-yl)methyl)-1*H*-indole-2-carboxamide (**25**)

Compound was synthesized using 3-chloro-5-methyl-1*H*-indole-2-carboxylic acid (81.7 mg, 0.39 mmol) and (6-morpholinopyridin-3-yl)methanamine hydrochloride (92 mg, 0.39 mmol) according to General Procedure C. The crude product was purified by FCC (0–5% DCM:MeOH) to yield the title compound as a white solid (17 mg, 11%). ^1^H NMR (500 MHz, DMSO-*d*_6_): δ 11.77 (s, 1H), 8.33 (t, *J* = 6.0 Hz, 1H), 8.17 (d, *J* = 2.4 Hz, 1H), 7.61 (dd, *J* = 8.8, 2.5 Hz, 1H), 7.37–7.31 (m, 2H), 7.12 (dd, *J* = 8.4, 1.7 Hz, 1H), 6.83 (d, *J* = 8.8 Hz, 1H), 4.42 (d, *J* = 5.9 Hz, 2H), 3.71–3.66 (m, 4H), 3.43–3.37 (m, 4H), 2.40 (s, 3H). HRMS (ESI): *m*/*z* [M + H]^+^ calcd for C_20_H_22_ClN_4_O_2_ 385.14258, found 385.14210.

#### 3,5-Dimethyl-*N*-((6-morpholinopyridin-3-yl)methyl)-1*H*-indole-2-carboxamide (**26**)

Compound was synthesized using 3,5-dimethyl-1*H*-indole-2-carboxylic acid (80 mg, 0.42 mmol) and (6-morpholinopyridin-3-yl)methanamine hydrochloride (126 mg, 1.3 mmol) according to General Procedure C. The crude product was purified by FCC (0–5% DCM:MeOH) to yield the title compound as a white solid (44 mg, 29%). ^1^H NMR (250 MHz, DMSO-*d*_6_) δ: 10.99 (s, 1H), 8.23 (t, *J* = 5.6 Hz, 1H), 8.15 (d, *J* = 2.3 Hz, 1H), 7.59 (dd, *J* = 8.5, 2.3 Hz, 1H), 7.35 (s, 1H), 7.24 (d, *J* = 8.3 Hz, 1H), 7.02 (d, *J* = 8.3 Hz, 1H), 6.83 (d, *J* = 8.7 Hz, 1H), 4.37 (d, *J* = 5.7 Hz, 2H), 3.69 (t, *J* = 4.8 Hz, 4H), 3.40 (t, *J* = 4.8 Hz, 4H), 2.46 (s, 3H), 2.38 (s, 3H). HRMS (ESI): *m*/*z* [M + H]^+^ calcd for C_21_H_25_N_4_O_2_ 365.19720, found 365.19682.

#### 7-Fluoro-5-methyl-*N*-((6-morpholinopyridin-3-yl)methyl)-1*H*-indole-2-carboxamide (**27**)

Compound was synthesized using 7-fluoro-5-methyl-1*H*-indole-2-carboxylic acid (90 mg, 0.46 mmol) and (6-morpholinopyridin-3-yl)methanamine hydrochloride (139 mg, 1.30 mmol) according to General Procedure C. The crude product was purified by FCC (0–5% DCM:MeOH) to yield the title compound as a white solid (110 mg, 64%). ^1^H NMR (250 MHz, DMSO-*d*_6_): δ 11.48 (s, 1H), 8.90 (t, *J* = 5.8 Hz, 1H), 8.14 (d, *J* = 2.4 Hz, 1H), 7.56 (dd, *J* = 8.7, 2.5 Hz, 1H), 7.19 (dd, *J* = 9.6, 2.5 Hz, 1H), 7.10 (d, *J* = 2.1 Hz, 1H), 6.93–6.77 (m, 2H), 4.38 (d, *J* = 5.8 Hz, 2H), 3.74–3.63 (m, 4H), 3.45–3.34 (m, 4H), (indole CH_3_ overlapped with DMSO). HRMS (ESI): *m*/*z* [M + H]^+^ calcd for C_20_H_22_FN_4_O_2_ 369.17213, found 369.17182.

#### *N*-(4-(Methylsulfonamido)benzyl)isoquinoline-3-carboxamide (**28**)

Compound was synthesized using isoquinoline-3-carboxylic acid (52 mg, 0.30 mmol) and *N*-(4-(aminomethyl)phenyl)methanesulfonamide hydrochloride (85 mg, 0.36 mmol) according to General Procedure C. The crude product was purified by FCC (0–5% DCM:MeOH) to yield the title compound as a white solid (53 mg, 50%). ^1^H NMR (250 MHz, DMSO-*d*_6_): δ 9.64 (s, 1H), 9.48–9.31 (m, 2H), 8.57 (d, *J* = 1.1 Hz, 1H), 8.23 (dd, *J* = 14.6, 7.9 Hz, 2H), 7.93–7.76 (m, 2H), 7.33 (d, *J* = 8.5 Hz, 2H), 7.22–7.10 (m, 2H), 4.51 (d, *J* = 6.4 Hz, 2H), 2.94 (s, 3H). HRMS (ESI): *m*/*z* [M + H]^+^ calcd for C_18_H_18_N_3_O_3_S 356.10634, found 356.10597.

#### *N*-((6-Morpholinopyridin-3-yl)methyl)isoquinoline-3-carboxamide (**29**)

Compound was synthesized using isoquinoline-3-carboxylic acid (52 mg, 0.30 mmol) and (6-morpholinopyridin-3-yl)methanamine hydrochloride (70 mg, 0.36 mmol) according to General Procedure C. The crude product was purified by FCC (0–5% DCM:MeOH) to yield the title compound as a white solid (62 mg, 59%). ^1^H NMR (250 MHz, DMSO-*d*_6_): δ 9.45–9.32 (m, 2H), 8.57 (s, 1H), 8.30–8.12 (m, 3H), 7.94–7.76 (m, 2H), 7.61 (dd, *J* = 8.7, 2.5 Hz, 1H), 6.81 (d, *J* = 8.8 Hz, 1H), 4.43 (d, *J* = 6.3 Hz, 2H), 3.74–3.64 (m, 4H), 3.44–3.35 (m, 4H). HRMS (ESI): *m*/*z* [M + H]^+^ calcd for C_20_H_21_N_4_O_2_ 349.16590, found 349.16559.

#### *N*-(4-(Methylsulfonamido)benzyl)-5-(trifluoromethyl)-1*H*-pyrrolo[3,2-*b*]pyridine-2-carboxamide (**30**)

Compound was synthesized using 5-(trifluoromethyl)-1*H*-pyrrolo[3,2-*b*]pyridine-2-carboxylic acid (69 mg, 0.30 mmol) and *N*-(4-(aminomethyl)phenyl)methanesulfonamide hydrochloride (85 mg, 0.36 mmol) according to General Procedure C. The crude product was purified by FCC (0–5% DCM:MeOH) to yield the title compound as a white solid (42 mg, 33%). ^1^H NMR (250 MHz, DMSO-*d*_6_): δ 12.33 (s, 1H), 9.68 (s, 1H), 9.35 (t, *J* = 6.0 Hz, 1H), 8.02 (d, *J* = 8.5 Hz, 1H), 7.66 (d, *J* = 8.6 Hz, 1H), 7.50–7.41 (m, 1H), 7.33 (d, *J* = 8.5 Hz, 2H), 7.19 (d, *J* = 8.4 Hz, 2H), 4.51 (d, *J* = 5.9 Hz, 2H), 2.95 (s, 3H). HRMS (ESI): *m*/*z* [M + H]^+^ calcd for C_17_H_16_F_3_N4O_3_S 413.08897, found 413.08859.

#### 5-Methyl-*N*-((6-morpholinopyridin-3-yl)methyl)benzofuran-2-carboxamide (**31**)

Compound was synthesized using 5-methylbenzofuran-2-carboxylic acid (200 mg, 1.13 mmol) and (6-morpholinopyridin-3-yl)methanamine (241 mg, 1.25 mmol) according to General Procedure C. The crude product was purified by FCC (0–5% DCM:MeOH) to yield the title compound as a white solid (110 mg, 27%). ^1^H NMR (400 MHz, DMSO-*d*_6_): δ 9.14 (t, *J* = 5.8 Hz, 1H), 8.11 (s, 1H), 7.56–7.46 (m, 4H), 7.26 (d, *J* = 8.6 Hz, 1H), 6.80 (d, *J* = 8.7 Hz, 1H), 4.32 (d, *J* = 5.7 Hz, 2H), 3.68 (t, *J* = 4.5 Hz, 4H), 3.39 (t, *J* = 4.6 Hz, 4H), 2.40 (s, 3H). LC–MS (*m*/*z*): 352.39 [M + H]^+^.

#### 5-Methyl-*N*-((6-morpholinopyridin-3-yl)methyl)-1*H*-benzo[*d*]imidazole-2-carboxamide (**32**)

Compound was synthesized using 5-methyl-1*H*-benzo[*d*]imidazole-2-carboxylic acid (150 mg, 0.82 mmol) and (6-morpholinopyridin-3-yl)methanamine (197.5 mg, 1.0 mmol) according to General Procedure C. The crude product was purified by reverse phase preparative HPLC to yield the title compound as a white solid (45 mg, 15%). ^1^H NMR (400 MHz, DMSO-*d*_6_): δ 12.03 (br.s.,1H), 8.43 (s, 0.5H), 8.18 (s, 1H), 7.92 (t, *J* = 6.4 Hz, 0.5H), 7.82 (d, *J* = 6.5 Hz, 0.5H), 7.63–7.59 (m, 1H), 7.24 (d, *J* = 8.8 Hz, 0.5H), 7.02 (d, *J* = 8.0 Hz, 0.6H), 6.94–6.91 (m, 1.5H), 6.77 (d, *J* = 8.8 Hz, 2H), 4.42 (d, *J* = 6.0 Hz, 2H), 3.66 (t, *J* = 4.8 Hz, 4H), 3.37 (t, *J* = 4.8 Hz, 4H), 2.28 (s, 3H) (rotameric mixture). LC–MS (*m*/*z*): 352.2 [M + H]^+^.

#### 2-(5-Methyl-1*H*-indol-1-yl)-*N*-(4-(methylsulfonamido)benzyl)acetamide (**33**)

Compound was synthesized using 2-(5-methyl-1*H*-indol-1-yl)acetic acid (125 mg, 0.66 mmol) and *N*-(4-(aminomethyl)phenyl)methanesulfonamide hydrochloride (210 mg, 1.34 mmol) according to General Procedure C. The crude product was purified by FCC (0–5% DCM:MeOH) to yield the title compound as a white solid (78 mg, 27%). ^1^H NMR (250 MHz, CDCl_3_): δ 7.47–7.40 (m, 1H), 7.18 (d, *J* = 8.4 Hz, 1H), 7.13–6.99 (m, 6H), 6.52 (d, *J* = 3.2 Hz, 1H), 5.70 (t, *J* = 5.6 Hz, 1H), 4.83 (s, 2H), 4.32 (d, *J* = 6.1 Hz, 2H), 2.96 (s, 3H), 2.45 (s, 3H). HRMS (ESI): *m*/*z* [M + H]^+^ calcd for C_19_H_23_N_3_O_3_S 372.13764, found 372.13719.

#### 2-(5-Methyl-1*H*-indol-1-yl)-*N*-((6-morpholinopyridin-3-yl)methyl)acetamide (**34**)

Compound was synthesized using 2-(5-methyl-1*H*-indol-1-yl)acetic acid (200 mg, 1.06 mmol) and (6-morpholinopyridin-3-yl)methanamine hydrochloride (364 mg, 1.59 mmol) according to General Procedure C. The crude product was purified by FCC (0–5% DCM:MeOH) to yield the title compound as a white solid (106 mg, 27%). ^1^H NMR (250 MHz, DMSO-*d*_6_): δ 8.49 (s, 1H), 8.03 (d, *J* = 2.4 Hz, 1H), 7.45 (dd, *J* = 8.7, 2.5 Hz, 1H), 7.31 (s, 1H), 7.28–7.17 (m, 2H), 6.92 (d, *J* = 8.3 Hz, 1H), 6.80 (d, *J* = 8.8 Hz, 1H), 6.37–6.29 (m, 1H), 4.79 (s, 2H), 4.16 (d, *J* = 5.7 Hz, 2H), 3.74–3.63 (m, 4H), 3.45–3.36 (m, 4H), 2.36 (s, 3H). HRMS (ESI): *m*/*z* [M + H]^+^ calcd for C_21_H_25_N_4_O_2_ 365.19720, found 365.19710.

#### 5-Methyl-*N*-((6-morpholinopyridin-3-yl)methyl)-1*H*-indole-3-carboxamide (**35**)

Compound was synthesized using 5-methyl-1*H*-indole-3-carboxylic acid (150 mg, 0.857 mmol) and (6-morpholinopyridin-3-yl)methanamine (198.5 mg, 1.0 mmol) according to General Procedure C. The crude product was purified by reverse phase preparative HPLC to yield the title compound as a white solid (165 mg, 55%). ^1^H NMR (400 MHz, DMSO-*d*_6_): δ 11.39 (s, 1H), 8.26 (t, *J* = 6.0 Hz, 1H), 8.11 (s, 1H), 7.94 (s, 2H), 7.55 (d, *J* = 6.8 Hz, 1H), 7.28 (d, *J* = 8.2 Hz, 1H), 6.96 (d, *J* = 8.4 Hz, 1H), 6.80 (d, *J* = 8.6 Hz, 1H), 4.32 (d, *J* = 5.5 Hz, 2H), 3.66 (t, *J* = 4.6 Hz, 4H), 3.38 (t, *J* = 4.4 Hz, 4H), 2.33 (s, 3H). LC–MS (*m*/*z*): 351.3 [M + H]^+^.

#### *N*-(4-(Ethylsulfonamido)benzyl)-5-methyl-1*H*-indole-2-carboxamide (**36**)

Compound was synthesized using 5-methyl-1*H*-indole-2-carboxylic acid (100 mg, 0.57 mmol) and *N*-(4-(aminomethyl)phenyl)ethanesulfonamide hydrochloride (143 mg, 0.57 mmol) according to General Procedure A. The crude product was purified by FCC (0–5% DCM:MeOH) to yield the title compound as a white solid (152 mg, 72%). ^1^H NMR (500 MHz, DMSO-*d*_6_): δ 11.46–11.42 (m, 1H), 9.72 (s, 1H), 8.93 (t, *J* = 6.1 Hz, 1H), 7.37 (s, 1H), 7.30 (dd, *J* = 10.9, 8.3 Hz, 3H), 7.18 (d, *J* = 8.2 Hz, 2H), 7.06 (d, *J* = 2.2 Hz, 1H), 7.00 (dd, *J* = 8.3, 1.6 Hz, 1H), 4.44 (d, *J* = 6.0 Hz, 2H), 3.04 (q, *J* = 7.3 Hz, 2H), 2.36 (s, 3H), 1.17 (t, *J* = 7.3 Hz, 3H). HRMS (ESI): *m*/*z* [M + H]^+^ calcd for C_19_H_22_N_3_O_3_S 372.13764, found 372.13722.

#### 5-Cyclopropyl-*N*-(4-(ethylsulfonamido)benzyl)-1*H*-indole-2-carboxamide (**37**)

Compound was synthesized using *N*-(4-aminobenzyl)-5-cyclopropyl-1*H*-indole-2-carboxamide (200 mg, 0.65 mmol) and ethanesulfonyl chloride (83 mg, 0.65 mmol) according to general procedure F to afford a white solid (60 mg, 23%). ^1^H NMR (400 MHz, DMSO-*d*_6_): δ 11.42 (s, 1H), 9.71 (s, 1H), 8.92 (t, *J* = 6.0 Hz, 1H), 7.30–7.27 (m, 4H), 7.17 (d, *J* = 8.4 Hz, 2H), 7.05 (s, 1H), 6.91 (d, *J* = 8.8 Hz, 1H), 4.44 (d, *J* = 5.8 Hz, 2H), 3.04 (q, *J* = 7.2 Hz, 2H), 1.97–1.94 (m, 1H), 1.69 (t, *J* = 7.32 Hz, 3H), 0.91–0.88 (m, 2H), 0.65–0.63 (m, 2H). LC–MS (*m*/*z*): 398.01 [M + H]^+^.

#### *N*-(4-(Cyclopropanesulfonamido)benzyl)-5-methyl-1*H*-indole-2-carboxamide (**38**)

Compound was synthesized using 5-methyl-1*H*-indole-2-carboxylic acid (100 mg, 0.57 mmol) and *N*-(4-(aminomethyl)phenyl)cyclopropanesulfonamide hydrochloride (165 mg, 0.62 mmol) according to General Procedure A. The crude product was purified by FCC (0–5% DCM:MeOH) to yield the title compound as a white solid (141 mg, 64%). ^1^H NMR (250 MHz, CD_3_OD): δ 7.40–7.29 (m, 4H), 7.29–7.21 (m, 2H), 7.06 (dd, *J* = 8.5, 1.7 Hz, 1H), 7.00 (d, *J* = 0.9 Hz, 1H), 4.55 (s, 2H), 2.59–2.42 (m, 1H), 2.40 (s, 3H), 1.07–0.97 (m, 2H), 0.97–0.86 (m, 2H). HRMS (ESI): *m*/*z* [M + H]^+^ calcd for C_20_H_22_N_3_O_3_S 384.13764, found 384.13716.

#### *N*-(4-(Cyclopropanesulfonamido)benzyl)-5-cyclopropyl-1*H*-indole-2-carboxamide (**39**)

Compound was synthesized using 5-cyclopropyl-1*H*-indole-2-carboxylic acid (100 mg, 0.49 mmol) and *N*-(4-(aminomethyl)phenyl)cyclopropanesulfonamide hydrochloride (131 mg, 0.49 mmol) according to General Procedure A. The crude product was purified by FCC (0–5% DCM:MeOH) to yield the title compound as a white solid (152 mg, 75%). ^1^H NMR (400 MHz, CD_3_OD): δ 7.37–7.29 (m, 4H), 7.28–7.23 (m, 2H), 6.99 (dd, *J* = 8.7, 1.4 Hz, 2H), 4.55 (s, 2H), 2.56–2.44 (m, 1H), 2.03–1.91 (m, 1H), 1.05–0.98 (m, 2H), 0.95–0.87 (m, 4H), 0.69–0.61 (m, 2H). HRMS (ESI): *m*/*z* [M + H]^+^ calcd for C_22_H_24_N_3_O_3_S 410.15329, found 410.15295.

#### 5-Methyl-*N*-(4-((trifluoromethyl)sulfonamido)benzyl)-1*H*-indole-2-carboxamide (**40**)

Compound was synthesized using 5-methyl-1*H*-indole-2-carboxylic acid (150 mg, 0.85 mmol) and *N*-(4-(aminomethyl)phenyl)-1,1,1-trifluoromethanesulfonamide hydrochloride (250 mg, 0.85 mmol) according to General Procedure C. The crude product was purified by FCC (0–5% DCM:MeOH) to yield the title compound as a white solid (172 mg, 49%). ^1^H NMR (500 MHz, Acetone-*d*_6_): δ 10.71 (br. s., 1H), 8.31 (t, *J* = 5.66 Hz, 1H), 7.41–7.48 (m, 3H), 7.38 (s, 1H), 7.33–7.37 (m, 2H), 7.04–7.09 (m, 2H), 4.64 (d, *J* = 5.66 Hz, 2H), 2.38 (s, 3H). HRMS (ESI): *m*/*z* [M + H]^+^ calcd for C_18_H_17_F_3_N_3_O_3_S 412.09372, found 412.09324.

#### 5-Cyclopropyl-*N*-(4-((trifluoromethyl)sulfonamido)benzyl)-1*H*-indole-2-carboxamide (**41**)

Compound was synthesized using 5-cyclopropyl-1*H*-indole-2-carboxylic acid (100 mg, 0.50 mmol) and *N*-(4-(aminomethyl)phenyl)-1,1,1-trifluoromethanesulfonamide hydrochloride (160 mg, 0.55 mmol) according to General Procedure C. The crude product was purified by FCC (0–5% DCM:MeOH) to yield the title compound as a white solid (70 mg, 32%). ^1^H NMR (600 MHz, Acetone-*d*_6_): δ 10.68 (br. s., 1H), 8.29 (t, *J* = 5.65 Hz, 1H), 7.41–7.47 (m, 3H), 7.32–7.37 (m, 3H), 7.06 (d, *J* = 1.51 Hz, 1H), 7.00 (dd, *J* = 1.69, 8.47 Hz, 1H), 4.63 (d, *J* = 6.21 Hz, 2H), 1.99 (m, 1H), 0.88–0.92 (m, 2H), 0.63–0.67 (m, 2H). HRMS (ESI): *m*/*z* [M + H]^+^ calcd for C_20_H_18_F_3_N_3_O_3_S 438.10937, found 438.10895.

#### 5-Methyl-*N*-(3-(methylsulfonamido)benzyl)-1*H*-indole-2-carboxamide (**42**)

Compound was synthesized using 5-methyl-1*H*-indole-2-carboxylic acid (150 mg, 0.85 mmol) and *N*-(3-(aminomethyl)phenyl)methanesulfonamide (210 mg, 0.90 mmol) according to General Procedure C. The crude product was purified by FCC (0–5% DCM:MeOH) to yield the title compound as a pale yellow solid (191 mg, 63%). ^1^H NMR (500 MHz, Acetone-*d*_6_): δ 10.78 (br. s., 1H), 8.60 (br. s., 1H), 8.33 (t, *J* = 5.66 Hz, 1H), 7.41 (d, *J* = 8.33 Hz, 1H), 7.38 (d, *J* = 0.63 Hz, 1H), 7.29–7.36 (m, 2H), 7.23–7.26 (m, 1H), 7.18 (d, *J* = 7.55 Hz, 1H), 7.04–7.09 (m, 2H), 4.63 (d, *J* = 6.13 Hz, 2H), 2.96 (s, 3H), 2.38 (s, 3H). HRMS (ESI): *m*/*z* [M + H]^+^ calcd for C_18_H_20_N_3_O_3_S 358.12199, found 358.12158.

#### 5-Methyl-*N*-(4-(*N*-methylsulfamoyl)benzyl)-1*H*-indole-2-carboxamide (**43**)

Compound was synthesized using 5-methyl-1*H*-indole-2-carboxylic acid (75 mg, 0.42 mmol) and *tert*-Butyl (4-(*N*-methylsulfamoyl)benzyl)carbamate (126.5 mg, 0.42 mmol) according to General Procedure E. The crude product was purified by FCC (0–5% DCM:MeOH) to yield the title compound as a white solid (25 mg, 33%). ^1^H NMR (500 MHz, Acetone-*d*_6_): δ 10.69 (br. s., 1H), 8.38 (br. s., 1H), 7.80 (d, *J* = 8.33 Hz, 2H), 7.59 (d, *J* = 8.17 Hz, 2H), 7.43 (d, *J* = 8.33 Hz, 1H), 7.39 (s, 1H), 7.04–7.10 (m, 2H), 6.28 (d, *J* = 4.87 Hz, 1H), 4.71 (d, *J* = 6.13 Hz, 2H), 2.56 (d, *J* = 5.50 Hz, 3H), 2.38 (s, 3H). HRMS (ESI): *m*/*z* [M + H]^+^ calcd for C_18_H_20_N_3_O_3_S 358.12199, found 358.12152.

#### 5-Cyclopropyl-*N*-(4-(*N*-methylsulfamoyl)benzyl)-1*H*-indole-2-carboxamide (**44**)

Compound was synthesized using 5-cyclopropyl-1*H*-indole-2-carboxylic acid (50 mg, 0.25 mmol) and *tert*-Butyl (4-(*N*-methylsulfamoyl)benzyl)carbamate (90 mg, 0.30 mmol) according to General Procedure E. The crude product was purified by reverse phase chromatography (30–100% MeOH:H_2_O) to yield the title compound as a white solid (20 mg, 25%). ^1^H NMR (500 MHz, DMSO-*d*_6_): δ 9.00–9.23 (m, 1H), 7.74 (d, *J* = 8.3 Hz, 2H), 7.53 (d, *J* = 8.3 Hz, 2H), 7.32 (s, 1H), 7.30 (s, 1H), 7.08 (s, 1H), 6.93 (dd, *J* = 1.5, 8.3 Hz, 1H), 4.58 (d, *J* = 5.4 Hz, 2H), 2.38 (s, 3H), 1.88–2.02 (m, 1H), 0.83–0.97 (m, 2H), 0.58–0.71 (m, 2H). LC–MS (*m*/*z*): 384.1 [M + H]^+^.

#### 5-Methyl-*N*-(4-(*N*-methylmethylsulfonamido)benzyl)-1*H*-indole-2-carboxamide (**45**)

Compound was synthesized using 5-methyl-1*H*-indole-2-carboxylic acid (106 mg, 0.60 mmol) and *N*-(4-(aminomethyl)phenyl)-*N*-methylmethanesulfonamide hydrochloride (167 mg, 0.66 mmol) according to General Procedure A. The crude product was purified by FCC (0–5% DCM:MeOH) to yield the title compound as a white solid (158 mg, 70%). ^1^H NMR (250 MHz, DMSO-*d*_6_): δ 11.47 (s, 1H), 9.00 (t, *J* = 6.0 Hz, 1H), 7.42–7.27 (m, 6H), 7.11–6.96 (m, 2H), 4.50 (d, *J* = 5.9 Hz, 2H), 3.21 (s, 3H), 2.92 (s, 3H), 2.36 (s, 3H). HRMS (ESI): *m*/*z* [M + H]^+^ calcd for C_19_H_22_N_3_O_3_S 372.13764, found 372.13736.

#### *N*-(3-Fluoro-4-(methylsulfonamido)benzyl)-5-methyl-1*H*-indole-2-carboxamide (**46**)

Compound was synthesized using 5-methyl-1*H*-indole-2-carboxylic acid (82.5 mg, 0.47 mmol) and *N*-(4-(aminomethyl)-2-fluorophenyl)methanesulfonamide hydrochloride according (100 mg, 0.39 mmol) to General Procedure A. The crude product was purified by FCC (0–5% DCM:MeOH) to yield the title compound as a white solid (115 mg, 78%). ^1^H NMR (250 MHz, DMSO-*d*_6_): δ 11.47 (s, 1H), 9.53 (s, 1H), 9.00 (t, *J* = 6.0 Hz, 1H), 7.41–7.28 (m, 3H), 7.27–7.12 (m, 2H), 7.10–6.97 (m, 2H), 4.48 (d, *J* = 6.0 Hz, 2H), 3.00 (s, 3H), 2.36 (s, 3H). HRMS (ESI): *m*/*z* [M + H]^+^ calcd for C_18_H_19_FN_3_O_3_S 376.11257, found 376.11221.

#### 5-Cyclopropyl-*N*-((6-(methylsulfonamido)pyridin-3-yl)methyl)-1*H*-indole-2-carboxamide (**47**)

Compound was synthesized using 5-cyclopropyl-1*H*-indole-2-carboxylic acid (45 mg, 0.15 mmol) and *N*-(5-(aminomethyl)pyridin-2-yl)methanesulfonamide (45 mg, 0.15 mmol) according to General Procedure C. The crude product was purified by reverse phase chromatography (30–100% MeOH:H_2_O) to yield the title compound as a white solid (12 mg, 21%). ^1^H NMR (500 MHz, CD_3_OD): δ 8.09–8.19 (m, 1H), 7.62–7.69 (m, 1H), 7.31 (s, 3H), 6.98 (s, 3H), 6.90–6.96 (m, 1H), 4.49 (s, 2H), 3.13 (s, 3H), 1.94–2.00 (m, 1H), 0.90–0.92 (m, 2H), 0.65 (dd, *J* = 1.7, 5.1 Hz, 2H). LC–MS (*m*/*z*): 358.2 [M + H]^+^.

#### 5-Cyclopropyl-*N*-(4-(methylsulfonyl)benzyl)-1*H*-indole-2-carboxamide (**48**)

Compound was synthesized using 5-cyclopropyl-1*H*-indole-2-carboxylic acid (80 mg, 0.40 mmol) and *tert*-Butyl (4-(methylsulfonyl)benzyl)carbamate (137 mg, 0.48 mmol) according to general procedure E. The crude product was purified by reverse phase chromatography (30–100% MeOH:H_2_O) to yield the title compound as a white solid (50 mg, 48%).^1^H NMR (500 MHz, DMSO-*d*_6_): δ 9.06–9.12 (m, 1H), 7.75 (d, *J* = 8.3 Hz, 2H), 7.54 (d, *J* = 8.3 Hz, 5H), 7.38–7.43 (m, 1H), 7.32 (s, 2H), 7.08 (s, 1H), 6.90–6.95 (m, 1H), 4.58 (d, *J* = 5.9 Hz, 2H), 2.39 (d, *J* = 3.4 Hz, 3H), 1.90–2.03 (m, 1H), 0.90 (s, 2H), 0.64 (dd, *J* = 1.2, 4.6 Hz, 2H). LC–MS (*m*/*z*): 369.1 [M + H]^+^.

#### 5-Cyclopropyl-*N*-(3-(methylsulfonamido)phenyl)-1*H*-indole-2-carboxamide (**49**)

Compound was synthesized using 5-cyclopropyl-1*H*-indole-2-carboxylic acid (100 mg, 0.50 mmol) and *N*-(3-aminophenyl)methanesulfonamide (93 mg, 0.50 mmol) according to General Procedure B. The crude product was purified by FCC (0–30% EtOAc:PE) to yield the title compound as a white solid (70 mg, 38%). ^1^H NMR (500 MHz, DMSO-*d*_6_): δ 11.60 (s, 1H), 10.20 (s, 1H), 9.71–9.89 (m, 1H), 7.70 (s, 1H), 7.52 (d, *J* = 7.8 Hz, 1H), 7.37 (s, 1H), 7.34 (d, *J* = 6.3 Hz, 2H), 7.27 (t, *J* = 8.1 Hz, 1H), 6.93–6.99 (m, 1H), 6.90 (d, *J* = 7.8 Hz, 1H), 2.99 (s, 3H), 1.95–2.03 (m, 1H), 0.88–0.94 (m, 2H), 0.63–0.69 (m, 2H). LCMS (*m*/*z*): 370.1 [M + H]^+^.

#### 5-Cyclopropyl-*N*-(4-(methylsulfonamido)phenyl)-1*H*-indole-2-carboxamide (**50**)

Compound was synthesized using 5-cyclopropyl-1*H*-indole-2-carboxylic acid (100 mg, 0.50 mmol) and using *N*-(4-aminophenyl)methanesulfonamide (93 mg, 0.50 mmol) according to General Procedure B. The crude product was purified by FCC (0–80% EtOAc:PE) to yield the title compound as a white solid (40 mg, 22%). ^1^H NMR (500 MHz, DMSO-*d*_6_): δ 11.57 (br. s., 1H), 10.12 (s, 1H), 9.53–9.68 (m, 1H), 7.69 (d, *J* = 8.8 Hz, 2H), 7.35–7.38 (m, 1H), 7.33 (d, *J* = 8.3 Hz, 1H), 7.28 (s, 1H), 7.15 (d, *J* = 8.8 Hz, 2H), 6.93–6.98 (m, 1H), 2.90 (s, 3H), 1.99 (s, 1H), 0.91 (dd, *J* = 1.5, 8.3 Hz, 2H), 0.63–0.68 (m, 2H). LC–MS (*m*/*z*): 370.1 [M + H]^+^.

#### 5-Methyl-*N*-(1-(methylsulfonyl)piperidin-4-yl)-1*H*-indole-2-carboxamide (**51**)

Compound was synthesized using 5-methyl-1*H*-indole-2-carboxylic acid (117 mg, 0.66 mmol) and 1-(methylsulfonyl)piperidin-4-amine hydrochloride (130 mg, 0.6 mmol) according to General Procedure A. The crude product was purified by FCC (0–5% DCM:MeOH) to yield the title compound as a beige solid (141 mg, 69%). ^1^H NMR (250 MHz, DMSO-*d*_6_): δ 11.42 (s, 1H), 8.29 (d, *J* = 7.8 Hz, 1H), 7.38 (s, 1H), 7.31 (d, *J* = 8.4 Hz, 1H), 7.10–7.02 (m, 1H), 7.00 (dd, *J* = 8.5, 1.6 Hz, 1H), 4.05–3.84 (m, 1H), 3.67–3.51 (m, 2H), 2.96–2.80 (m, 5H), 2.36 (s, 3H), 2.04–1.84 (m, 2H), 1.72–1.51 (m, 2H). HRMS (ESI): *m*/*z* [M + H]^+^ calcd for C_16_H_22_N_3_O_3_S 336.13764, found 336.13735.

#### 5-Methyl-*N*-((6-(piperazin-1-yl)pyridin-3-yl)methyl)-1*H*-indole-2-carboxamide (**52**)

To a solution of 5-methyl-1*H*-indole-2-carboxylic acid (400 mg, 2.28 mmol) and TBTU (806 mg, 2.51 mmol) in DMF (5 mL) at 0 °C was added diisopropylethylamine (2.0 mL, 11.42 mmol), followed by (6-chloropyridin-3-yl)methanamine dihydrochloride (541 mg, 2.51 mmol). The reaction was stirred at this temperature for a further 15 min and then allowed to warm to room temperature and stirred for 16 h. The reaction was quenched with water (20 mL) and stirred for 30 min. The resultant precipitate was filtered, washed with water, and dried under high vacuum to give *N*-((6-chloropyridin-3-yl)methyl)-5-methyl-1*H*-indole-2-carboxamide (302 mg, 44%) which was used for the next step. To an oven-dried flask containing *N*-((6-chloropyridin-3-yl)methyl)-5-methyl-1*H*-indole-2-carboxamide (100 mg, 0.33 mmol), XPhos Pd G2 (5.2 mg, 0.007 mmol) and XPhos (3.2 mg, 0.007 mmol) was added *tert*-butyl piperazine-1-carboxylate (124.7 mg, 0.67 mmol) followed by lithium bis(trimethylsilyl)amide (1 M in THF, 1.3 mL, 1.33 mmol) and the resultant reaction solution was heated to 65 °C and stirred for 2 h. The reaction was cooled to rt and quenched with water (1 mL). The mixture was concentrated in vacuo and then partitioned between EtOAc (20 mL) and saturated NH_4_Cl (10 mL). The mixture was separated and the organic was dried (MgSO_4_), concentrated in vacuo and purified by flash chromatography (0–100% EtOAc in pet. ether) to give *tert*-butyl 4-(5-((5-methyl-1*H*-indole-2-carboxamido)methyl) pyridine-2-yl)piperazine-1-carboxylate (120 mg, 0.27 mmol, 81%). To a solution of *tert*-butyl 4-(5-((5-methyl-1*H*-indole-2-carboxamido)methyl) pyridine-2-yl)piperazine-1-carboxylate (120 mg, 0.27 mmol) in MeOH (3 mL) was added 2 M HCl in diethyl ether (1.35 mL, 2.70 mmol) and the reaction was stirred at rt until completion. The reaction was concentrated in vacuo and partitioned between DCM (10 mL) and 1 M NaOH (5 mL). The mixture was separated, the organic was dried (MgSO_4_) and concentrated in vacuo. The crude product was purified by flash chromatography (0–20% MeOH in DCM) to give 5-methyl-*N*-((6-(piperazin-1-yl)pyridin-3-yl)methyl)-1*H*-indole-2-carboxamide (89 mg, 94%). ^1^H NMR (250 MHz, CD_3_OD): δ 8.13 (d, *J* = 2.4 Hz, 1H), 7.62 (dd, *J* = 8.8, 2.5 Hz, 1H), 7.40–7.24 (m, 2H), 7.05 (dd, *J* = 8.6, 1.7 Hz, 1H), 6.97 (d, *J* = 0.9 Hz, 1H), 6.81 (d, *J* = 8.8 Hz, 1H), 4.46 (s, 2H), 3.53–3.38 (m, 4H), 2.98–2.84 (m, 4H), 2.39 (s, 3H). HRMS (ESI): *m*/*z* [M + H]^+^ calcd for C_20_H_24_N_5_O 350.19754, found 350.19744.

#### 5-Methyl-*N*-((6-(4-methylpiperazin-1-yl)pyridin-3-yl)methyl)-1*H*-indole-2-carboxamide (**53**)

Compound was synthesized using 5-methyl-1*H*-indole-2-carboxylic acid (55 mg, 0.31 mmol) and (6-(4-methylpiperazin-1-yl)pyridin-3-yl)methanamine (95 mg, 0.31 mmol) according to General Procedure C. The crude product was purified by reverse phase chromatography (30–100% MeOH:H_2_O) to yield the title compound as a white solid (50 mg, 44%). ^1^H NMR (500 MHz, DMSO-*d*_6_): δ 11.39–11.49 (m, 1H), 8.80–8.93 (m, 1H), 8.04–8.16 (m, 1H), 7.48–7.56 (m, 1H), 7.36 (s, 1H), 7.26–7.32 (m, 1H), 7.02 (d, *J* = 1.5 Hz, 2H), 6.75–6.89 (m, 1H), 4.34 (d, *J* = 5.9 Hz, 2H), 3.46 (br. s., 4H), 2.39–2.48 (m, 4H), 2.35 (s, 3H), 2.25 (br. s., 3H). LC–MS (*m*/*z*): 364.2 [M + H]^+^.

#### *N*-((6-(4-Methoxypiperidin-1-yl)pyridin-3-yl)methyl)-5-methyl-1*H*-indole-2-carboxamide (**54**)

Compound was synthesized using 5-methyl-1*H*-indole-2-carboxylic acid (100 mg, 0.57 mmol) and (6-(4-methoxypiperidin-1-yl)pyridin-3-yl)methanamine (126 mg, 0.57 mmol) according to General Procedure C. The crude product was purified by FCC (0–5% CHCl_3_:MeOH) to yield the title compound as a white solid (108 mg, 51%). ^1^H NMR (400 MHz, CDCl_3_): δ 9.57 (br. s., 1H), 8.15 (d, *J* = 2.45 Hz, 1H), 7.51 (dd, *J* = 2.45, 8.80 Hz, 1H), 7.39 (s, 1H), 7.32 (d, *J* = 8.31 Hz, 1H), 7.11 (dd, *J* = 1.47, 8.31 Hz, 1H), 6.76 (d, *J* = 1.47 Hz, 1H), 6.64 (d, *J* = 8.80 Hz, 1H), 6.57 (t, *J* = 5.38 Hz, 1H), 4.52 (d, *J* = 5.38 Hz, 2H), 3.90–4.03 (m, 2H), 3.43 (dt, *J* = 4.16, 8.19 Hz, 1H), 3.38 (s, 3H), 3.20 (ddd, *J* = 3.42, 9.42, 13.08 Hz, 2H), 2.43 (s, 3H), 1.91–2.01 (m, 2H), 1.54–1.68 (m, 2H). HRMS (ESI): *m*/*z* [M + H]+ calcd for C_22_H_27_N_4_O_2_ 379.21285 found 379.21251.

#### *N*-((6-(4,4-Difluoropiperidin-1-yl)pyridin-3-yl)methyl)-5-methyl-1*H*-indole-2-carboxamide (**55**)

Compound was synthesized using 5-methyl-1*H*-indole-2-carboxylic acid (90 mg, 0.51 mmol) and (6-(4,4-difluoropiperidin-1-yl)pyridin-3-yl)methanamine hydrochloride (160.8 mg, 0.61 mmol) according to General Procedure C. The crude product was purified by FCC (0–5% DCM:MeOH) to yield the title compound as a white solid (103 mg, 53%). ^1^H NMR (400 MHz, DMSO-*d*_6_): δ 11.44 (s, 1H), 8.86 (t, *J* = 5.9 Hz, 1H), 8.12 (d, *J* = 2.4 Hz, 1H), 7.56 (dd, *J* = 8.8, 2.5 Hz, 1H), 7.37 (s, 1H), 7.30 (d, *J* = 8.4 Hz, 1H), 7.04–6.97 (m, 2H), 6.94 (d, *J* = 8.7 Hz, 1H), 4.36 (d, *J* = 5.9 Hz, 2H), 3.70–3.62 (m, 4H), 2.35 (s, 3H), 1.95 (tt, *J* = 14.2, 5.7 Hz, 4H). HRMS (ESI): *m*/*z* [M + H]^+^ calcd for C_21_H_23_F_2_N_4_O 385.18344, found 385.18303.

#### *N*-((6-(1,1-Dioxidothiomorpholino)pyridin-3-yl)methyl)-5-methyl-1*H*-indole-2-carboxamide (**56**)

Compound was synthesized using 5-methyl-1*H*-indole-2-carboxylic acid (200 mg, 1.13 mmol) and 4-(5-(aminomethyl)pyridin-2-yl)tetrahydro-2*H*-thiopyran 1,1-dioxide hydrochloride (301 mg, 1.25 mmol) according to general procedure C. Product was purified by reverse phase preparative HPLC to get desired product (50 mg, 11%). ^1^H NMR (400 MHz, DMSO-*d*_6_): δ 11.43 (s, 1H), 8.87 (t, *J* = 6.0 Hz, 1H), 8.16 (d, *J* = 2.1 Hz, 1H), 7.60 (dd, *J* = 8.7 and 2.3 Hz, 1H), 7.37 (s, 1H), 7.30 (d, *J* = 8.2 Hz, 1H), 7.02–6.99 (m, 3H), 4.38 (d, *J* = 5.5 Hz, 2H), 4.03 (t, *J* = 4.7 Hz, 4H), 3.06 (t, *J* = 4.7 Hz, 4H), 2.36 (s, 3H). LC–MS (*m*/*z*): 399.34 [M + H]^+^.

#### 5-Methyl-*N*-((6-(3-methylmorpholino)pyridin-3-yl)methyl)-1*H*-indole-2-carboxamide (**57**)

Compound was synthesized using 5-methyl-1*H*-indole-2-carboxylic acid (149 mg, 0.85 mmol) and (6-(3-methylmorpholino)pyridin-3-yl)methanamine (261 mg, 0.85 mmol) according to General Procedure C. The crude product was purified by reverse phase chromatography (30–100% MeOH:H_2_O) to yield the title compound as a white solid (35 mg, 12%). ^1^H NMR (500 MHz, CDCl_3_): δ 9.58 (br. s., 1H), 8.20 (d, *J* = 1.5 Hz, 1H), 7.55 (dd, *J* = 2.4, 8.8 Hz, 1H), 7.39 (s, 1H), 7.33 (d, *J* = 8.8 Hz, 1H), 7.11 (d, *J* = 8.3 Hz, 1H), 6.75 (s, 1H), 6.56 (d, *J* = 8.8 Hz, 1H), 6.47–6.53 (m, 1H), 4.55 (d, *J* = 5.4 Hz, 2H), 4.28 (d, *J* = 6.3 Hz, 1H), 4.01 (dd, *J* = 3.4, 11.2 Hz, 1H), 3.74–3.85 (m, 3H), 3.61 (dt, *J* = 2.9, 11.7 Hz, 1H), 3.21 (dt, *J* = 3.9, 12.4 Hz, 1H), 2.43 (s, 3H), 1.23 (d, *J* = 6.3 Hz, 3H). LC–MS (*m*/*z*): 365.2 [M + H]^+^.

#### *N*-((6-(7-Oxa-2-azabicyclo[2.2.1]heptan-2-yl)pyridin-3-yl)methyl)-5-methyl-1*H*-indole-2-carboxamide (**58**)

Compound was synthesized using 5-methyl-1*H*-indole-2-carboxylic acid (193 mg, 1.10 mmol) and (6-(7-oxa-2-azabicyclo[2.2.1]heptan-2-yl)pyridin-3-yl)methanamine (336 mg, 1.10 mmol) according to General Procedure C. The crude product was purified by reverse phase chromatography (30–100% MeOH:H_2_O) to yield the title compound as a white solid (37 mg, 10%). ^1^H NMR (500 MHz, CDCl_3_): δ 9.56 (br. s., 1H), 8.12 (s, 1H), 7.52 (dd, *J* = 2.2, 8.5 Hz, 1H), 7.39 (s, 1H), 7.33 (d, *J* = 1.0 Hz, 1H), 7.11 (d, *J* = 8.8 Hz, 1H), 6.76 (s, 1H), 6.56 (br. s., 1H), 6.35 (d, *J* = 8.8 Hz, 1H), 4.88 (s, 1H), 4.69 (s, 1H), 4.53 (d, *J* = 5.9 Hz, 2H), 3.87 (s, 2H), 3.50 (d, *J* = 9.3 Hz, 1H), 3.35 (d, *J* = 9.8 Hz, 1H), 2.44 (s, 3H), 1.96 (s, 2H). LC–MS (*m*/*z*): 363.1 [M + H]^+^.

#### *N*-(3-Fluoro-4-morpholinobenzyl)-5-methyl-1*H*-indole-2-carboxamide (**59**)

Compound was synthesized using 5-methyl-1*H*-indole-2-carboxylic acid (150 mg, 0.85 mmol) and (3-fluoro-4-morpholinophenyl)methanamine hydrochloride according (232 mg, 0.94 mmol) to General Procedure A. The crude product was purified by FCC (0–5% DCM:MeOH) to yield the title compound as a white solid (20 mg, 25%). ^1^H NMR (250 MHz, DMSO-*d*_6_): δ 11.45 (s, 1H), 8.93 (t, *J* = 6.1 Hz, 1H), 7.38 (s, 1H), 7.31 (d, *J* = 8.4 Hz, 1H), 7.17–6.93 (m, 6H), 4.42 (d, *J* = 6.0 Hz, 2H), 3.78–3.67 (m, 4H), 3.01–2.90 (m, 4H), 2.36 (s, 3H). HRMS (ESI): *m*/*z* [M + H]^+^ calcd for C_21_H_23_FN_3_O_2_ 368.17688, found 368.17654.

#### 5-Methyl-*N*-((5-methyl-6-morpholinopyridin-3-yl)methyl)-1*H*-indole-2-carboxamide (**60**)

Compound was synthesized using 5-methyl-1*H*-indole-2-carboxylic acid (85 mg, 0.48 mmol) and (5-methyl-6-morpholinopyridin-3-yl)methanamine (100 mg, 0.48 mmol) according to General Procedure C. The crude product was purified by preparative reverse phase HPLC using 10–40% MeCN:water (TFA) system to yield the title compound as a white solid (25 mg, 14%). ^1^H NMR (500 MHz, DMSO-*d*_6_): δ 11.45 (s, 1H), 8.92 (s, 1H), 8.10 (s, 1H), 7.56 (br. s., 1H), 7.37 (s, 1H), 7.30 (d, *J* = 8.8 Hz, 1H), 7.04 (s, 1H), 7.00 (d, *J* = 8.8 Hz, 1H), 4.41 (d, *J* = 5.9 Hz, 2H), 3.71–3.74 (m, 4H), 3.03–3.08 (m, 4H), 2.35 (s, 3H), 2.25 (s, 3H). LC–MS (*m*/*z*): 365.2 [M + H]^+^.

#### 5-Methyl-*N*-((5-morpholinopyridin-2-yl)methyl)-1*H*-indole-2-carboxamide (**61**)

Compound was synthesized using 5-methyl-1*H*-indole-2-carboxylic acid (200 mg, 1.03 mmol) and (5-morpholinopyridin-2-yl)methanamine (199.48 mg, 1.14 mmol) according to general procedure C. The crude product was purified by reverse phase preparative HPLC to get the desired product as a white solid (90 mg, 25%). ^1^H NMR (400 MHz, DMSO-*d*_6_): δ 11.48 (s, 1H), 8.94 (t, *J* = 6.0 Hz, 1H), 8.20 (d, *J* = 3.12 Hz, 1H), 7.61 (d, *J* = 6.5 Hz, 1H), 7.38 (s, 1H), 7.32 (d, *J* = 8.4 Hz, 1H), 7.08 (s, 1H), 7.06–7.00 (m, 1H), 4.52 (d, *J* = 5.6 Hz, 2H), 3.75 (t, *J* = 4.0 Hz, 4H), 3.00 (t, *J* = 4.0 Hz, 4H), 2.36 (s, 3H). LC–MS (*m*/*z*): 351.12 [M + H]^+^.

#### 5-Methyl-*N*-((2-morpholinopyrimidin-5-yl)methyl)-1*H*-indole-2-carboxamide (**62**)

Compound was synthesized using 5-methyl-1*H*-indole-2-carboxylic acid (297 mg, 1.69 mmol) and (2-morpholinopyrimidin-5-yl)methanamine (300 mg, 1.54 mmol) according to general procedure C. The crude product was purified by reverse phase preparative HPLC to afford the desired product as a white solid (52 mg, 11%). ^1^H NMR (400 MHz, DMSO-*d*_6_): δ 11.43 (s, 1H), 8.85 (t, *J* = 5.9 Hz, 1H), 8.39 (s, 2H), 7.36 (s, 1H), 7.29 (d, *J* = 8.4 Hz, 1H), 7.00–6.98 (m, 2H), 4.30 (d, *J* = 5.6 Hz, 2H), 3.68–3.61 (m, 8H), 2.35 (m, 3H). LC–MS (*m*/*z*): 352.29 [M + H]^+^.

#### 5-Methyl-*N*-((6-morpholinopyridin-2-yl)methyl)-1*H*-indole-2-carboxamide (**63**)

Compound was synthesized using 5-methyl-1*H*-indole-2-carboxylic acid (200 mg, 1.14 mmol) and (6-morpholinopyridin-2-yl)methanamine (243 mg, 1.25 mmol) according to general procedure C. The crude product was purified by reverse phase preparative HPLC to afford the desired product as a white solid (75 mg, 19%). ^1^H NMR (400 MHz, DMSO-*d*_6_): δ 11.45 (s, 1H), 8.92 (t, *J* = 6.0 Hz, 1H), 7.52 (t, *J* = 7.8 Hz, 1H), 7.38 (s, 1H), 7.31 (d, *J* = 8.3 Hz, 1H), 7.10 (s, 1H), 7.01 (d, *J* = 8.2 Hz, 1H), 6.68 (d, *J* = 8.4 Hz, 1H), 6.63 (d, *J* = 7.3 Hz, 1H), 4.42 (d, *J* = 5.9 Hz, 2H), 3.68 (t, *J* = 4.5 Hz, 4H), 3.44 (t, *J* = 4.5 Hz, 4H), 2.36 (s, 3H). LC–MS (*m*/*z*): 351.35 [M + H]^+^.

#### 5-Methyl-*N*-((6-(morpholinomethyl)pyridin-3-yl)methyl)-1*H*-indole-2-carboxamide (**64**)

Compound was synthesized using 5-methyl-1*H*-indole-2-carboxylic acid (318 mg, 1.7 mmol) and (6-(morpholinomethyl)pyridin-3-yl)methanamine (320 mg, 1.5 mmol) according to general procedure C. The crude product was purified by reverse phase preparative HPLC to afford the desired product as a white solid (90 mg, 16%). ^1^H NMR (400 MHz, DMSO-*d*_6_): δ 11.48 (s, 1H), 9.02 (d, *J* = 6.0 Hz, 1H), 8.47 (s, 1H), 7.71 (d, *J* = 6.4 Hz, 1H), 7.40 (m, 2H), 7.37 (s, 1H), 7.30 (d, *J* = 8.0 Hz, 1H), 7.04 (s, 1H), 7.00 (d, *J* = 8.4 Hz, 1H), 4.49 (d, *J* = 5.4 Hz, 2H), 3.55 (m, 6H), 2.38–2.35 (m, 7H). LC–MS (*m*/*z*): 365.1 [M + H]^+^.

#### 5-Methyl-*N*-(2-morpholinoethyl)-1*H*-indole-2-carboxamide (**65**)

Compound was synthesized using of 5-methyl-1*H*-indole-2-carbonyl chloride (50 mg, 0.26 mmol) and 2-morpholinoethan-1-amine (50 μL, 0.39 mmol) according to General Procedure D. The crude product was purified by FCC (0–20% DCM:MeOH) to yield the title compound as a white solid (62 mg, 72%). ^1^H NMR (250 MHz, DMSO-*d*_6_): δ 11.40 (s, 1H), 8.35 (t, *J* = 5.8 Hz, 1H), 7.37 (s, 1H), 7.30 (d, *J* = 8.4 Hz, 1H), 7.05–6.96 (m, 2H), 3.63–3.52 (m, 4H), 3.40 (q, *J* = 6.5 Hz, 2H), 2.48–2.45 (m, 2H), 2.45–2.40 (m, 4H), 2.36 (s, 3H). HRMS (ESI): *m*/*z* [M + H]^+^ calcd for C_16_H_22_N_3_O_2_ 288.17065, found 288.17052.

#### 5-Methyl-*N*-((6-(trifluoromethyl)pyridin-3-yl)methyl)-1*H*-indole-2-carboxamide (**66**)

Compound was synthesized using 5-methyl-1*H*-indole-2-carboxylic acid (53 mg, 0.30 mmol) and (6-(trifluoromethyl)pyridin-3-yl)methanamine (63 mg, 0.36 mmol) according to General Procedure C. The crude product was purified by FCC (0–5% DCM:MeOH) to yield the title compound as a white solid (69 mg, 69%). ^1^H NMR (250 MHz, DMSO-*d*_6_): δ 11.49 (s, 1H), 9.11 (t, *J* = 6.0 Hz, 1H), 8.76 (s, 1H), 8.01 (d, *J* = 8.8 Hz, 1H), 7.95–7.83 (m, 1H), 7.39 (s, 1H), 7.31 (d, *J* = 8.4 Hz, 1H), 7.11–6.94 (m, 2H), 4.62 (d, *J* = 5.9 Hz, 2H), 2.36 (s, 3H). HRMS (ESI): *m*/*z* [M + H]^+^ calcd for C_17_H_15_F_3_N_3_O 334.11617, found 334.11586.

#### 2-(5-Methyl-1*H*-indol-2-yl)-*N*-(6-morpholinopyridin-3-yl)acetamide (**67**)

To a solution of ethyl 2-(5-methyl-1*H*-indol-2-yl)acetate (0.2 g, 0.9 mmol) and 6-morpholinopyridin-3-amine (0.33 g, 1.8 mmol) in toluene (5.0 mL) dropwise added Me_3_Al (0.1 mL) at room temperature. Then, the reaction mixture was heated at 120 °C for 16 h. It was concentrated and purified by purified by reverse phase preparative HPLC to afford the desired product (60 mg, 19%). ^1^H NMR (400 MHz, DMSO-*d*_6_): δ 10.75 (s, 1H), 9.91 (s, 1H), 8.31 (s, 1H), 7.80 (d, *J* = 8.0 Hz, 1H), 7.36 (s, 1H), 7.22 (d, *J* = 7.9 Hz, 1H), 7.18 (s, 1H), 6.89 (d, *J* = 8.4 Hz, 1H), 6.80 (d, *J* = 9.24 Hz, 1H), 3.70–3.65 (m, 6H), 3.35–3.30 (m, 4H), 2.36 (s, 3H). LC–MS (*m*/*z*): 351.40 [M + H]^+^.

#### 5-Methyl-*N*-(2-(6-morpholinopyridin-3-yl)ethyl)-1*H*-indole-2-carboxamide (**68**)

Compound was synthesized using 5-methyl-1*H*-indole-2-carboxylic acid (80 mg, 0.45 mmol) and 2-(6-morpholinopyridin-3-yl)ethan-1-amine (114 mg, 0.54 mmol) according to general procedure C. The crude was purified by Reverse phase preparative HPLC to afford a white solid (10 mg, 6%). ^1^H NMR (400 MHz, DMSO-*d*_6_): δ 11.38 (s, 1H), 8.46 (t, *J* = 5.0 Hz, 1H), 8.01 (s, 1H), 7.46 (d, *J* = 8.6 Hz, 1H), 7.36 (s, 1H), 7.29 (d, *J* = 7.9 Hz, 1H), 6.99 (d, *J* = 8.2 Hz, 1H), 6.98 (s, 1H), 6.77 (d, *J* = 8.8 Hz, 1H), 3.69 (t, *J* = 4.8 Hz, 4H), 3.47–3.42 (m, 2H), 3.36 (t, *J* = 4.7 Hz, 4H), 2.75 (t, *J* = 6.8 Hz, 2H), 2.35 (s, 3H). LC–MS (*m*/*z*): 365.21 [M + H]^+^.

#### *N*-(5-Methyl-1*H*-indol-2-yl)-2-(6-morpholinopyridin-3-yl)acetamide (**69**)

Compound was synthesized using 2-(6-morpholinopyridin-3-yl)acetic acid (40 mg, 0.18 mmol) and 5-methyl-1*H*-indol-2-amine (29 mg, 0.2 mmol) according to general procedure C and purified by reverse phase preparative HPLC to afford a white solid (5 mg, 8%). ^1^H NMR (400 MHz, DMSO-*d*_6_): δ 10.78 (s, 1H), 10.74 (s, 1H), 8.08 (s, 1H), 7.54 (dd, *J* = 8.9 and 1.96 Hz, 1H), 7.23 (d, *J* = 8.1 Hz, 1H), 7.13 (s, 1H), 6.82 (d, *J* = 8.7 Hz, 1H), 6.76 (d, *J* = 8.2 Hz, 1H), 5.92 (s, 1H), 3.68 (t, *J* = 4.68 Hz, 4H), 3.56 (s, 2H), 3.39 (t, *J* = 4.9 Hz, 4H), 2.31 (s, 3H). LC–MS (*m*/*z*): 351.31 [M + H]^+^.

#### 5-Methyl-*N*-((6-morpholinopyridin-3-yl)methyl)-1*H*-indole-2-sulfonamide (**70**)

Compound was synthesized using *tert*-butyl 2-(chlorosulfonyl)-5-methyl-1*H*-indole-1-carboxylate (329 mg, 1 mmol) and (6-morpholinopyridin-3-yl)methanamine (231 mg, 1.2 mmol) according to general procedure F. The corresponding sulfonamide derivative was dissolved in DCM (5 mL) and was deprotected using TFA (0.07 mL), added at 0 °C and stirred for 2h. After completion of the reaction the mixture was poured into an ice cold solution of saturated sodium bicarbonate and extracted with DCM. The organic layer was washed with brine, dried over anhydrous sodium sulfate. It was concentrated and purified by purified by Reverse phase preparative HPLC to afford the desired product as a white solid (25 mg, 6% over 2 steps). ^1^H NMR (400 MHz, DMSO-*d*_6_): δ 11.79 (s, 1H), 8.05 (t, *J* = 6.0 Hz, 1H), 7.96 (s, 1H), 7.45–7.40 (m, 2H), 7.32 (d, *J* = 8.3 Hz, 1H), 7.10 (d, *J* = 8.0 Hz, 1H), 6.82 (s, 1H), 6.70 (d, *J* = 9.0 Hz, 1H), 3.98 (d, *J* = 5.3 Hz, 2H), 3.65 (t, *J* = 4.2 Hz, 4H), 3.35–3.29 (m, 4H), 2.37 (s, 3H). LC–MS (*m*/*z*): 387.20 [M + H]^+^.

#### 5-Methyl-*N*-(1-(6-morpholinopyridin-3-yl)ethyl)-1*H*-indole-2-carboxamide (**71**)

Compound was synthesized using 5-methyl-1*H*-indole-2-carboxylic acid (35 mg, 0.2 mmol) and 1-(6-morpholinopyridin-3-yl)ethan-1-amine (49.69 mg, 0.24 mmol) according to general procedure C and purified by FCC to afford a white solid (18 mg, 25%). ^1^H NMR (400 MHz, DMSO-*d*_6_): δ 11.37 (s, 1H), 8.65 (d, *J* = 6.0 Hz, 1H), 8.15 (s, 1H), 7.65 (d, *J* = 5.5 Hz, 1H), 7.37 (s, 1H), 7.29 (d, *J* = 8.3 Hz, 1H), 7.10 (s, 1H), 7.00 (d, *J* = 7.7 Hz, 1H), 6.90–6.83 (m, 1H), 5.11 (t, *J* = 7.0 Hz, 1H), 3.68 (t, *J* = 4.4 Hz, 4H), 3.39 (t, *J* = 4.7 Hz, 4H), 2.35 (s, 3H), 1.47 (d, *J* = 7.0 Hz, 3H). LC–MS (*m*/*z*): 365.41 [M + H]^+^.

#### *N*,5-Dimethyl-*N*-((6-morpholinopyridin-3-yl)methyl)-1*H*-indole-2-carboxamide (**72**)

Compound was synthesized using 5-methyl-1*H*-indole-2-carboxylic acid (175 mg, 1.0 mmol) and *N*-methyl-1-(6-morpholinopyridin-3-yl)methanamine hydrochloride (240 mg, 1.0 mmol) according to General Procedure C. The crude product was purified by FCC (0–5% CHCl_3_:MeOH) to yield the title compound as a white solid (130 mg, 35%). ^1^H NMR (250 MHz, CDCl_3_): δ 9.72 (br. s., 1H), 8.19 (d, *J* = 2.21 Hz, 1H), 7.54 (d, *J* = 7.40 Hz, 1H), 7.29–7.45 (m, 2H), 7.06–7.17 (m, 1H), 6.75 (br. s., 1H), 6.64 (d, *J* = 8.70 Hz, 1H), 4.78 (br. s., 2H), 3.77–3.90 (m, 4H), 3.46–3.59 (m, 4H), 3.27 (br. s., 3H), 2.43 (s, 3H). HRMS (ESI): *m*/*z* [M + H]^+^ calcd for C_21_H_25_N_4_O_2_ 365.19720, found 365.19688.

#### *N*,1,5-Trimethyl-*N*-((6-morpholinopyridin-3-yl)methyl)-1*H*-indole-2-carboxamide (**73**)

Compound was synthesized using 1,5-dimethyl-1*H*-indole-2-carboxylic acid (110 mg, 0.58 mmol) and *N*-methyl-1-(6-morpholinopyridin-3-yl)methanamine hydrochloride (141.3 mg, 0.58 mmol) according to general procedure C. The crude was purified by FCC and washed with diethyl ether to afford a crystalline white solid (89 mg, 41%). ^1^H NMR (500 MHz, CDCl_3_): δ 8.16 (br. s., 1H), 7.60 (br. s., 1H), 7.39 (s, 1H), 7.24–7.29 (m, 1H), 7.13 (dd, *J* = 1.18, 8.41 Hz, 1H), 6.66 (d, *J* = 8.65 Hz, 1H), 6.57 (s, 1H), 4.67 (s, 2H), 3.79–3.90 (m, 7H), 3.49–3.57 (m, 4H), 3.08 (br. s., 3H), 2.45 (s, 3H). HRMS (ESI): *m*/*z* [M + H]^+^ calcd for C_22_H_27_N_4_O_2_ 379.21285, found 379.21259.

#### 5-Cyclopropyl-*N*-(4-((difluoromethyl)sulfonamido)benzyl)-1*H*-indole-2-carboxamide (**74**)

Compound was synthesized using *N*-(4-aminobenzyl)-5-cyclopropyl-1*H*-indole-2-carboxamide (0.2 g, 0.65 mmol) and difluoromethanesulfonyl chloride (97.8 mg, 0.65 mmol) according to general procedure F to afford a white solid (90 mg, 30%). ^1^H NMR (400 MHz, DMSO-*d*_6_): δ 11.42 (s, 1H), 10.87 (s, 1H), 8.95 (t, *J* = 6.0 Hz, 1H), 7.32–7.28 (m, 4H), 7.24–6.97 (m, 4H), 6.92 (dd, *J* = 8.6 and 1.0 Hz, 1H), 4.46 (d, *J* = 5.9 Hz, 2H), 1.97–1.95 (m, 1H), 0.91–0.87 (m, 2H), 0.65–0.63 (m, 2H). LC–MS (*m*/*z*): 420.06 [M + H]^+^.

#### 5-Cyclopropyl-*N*-(4-((fluoromethyl)sulfonamido)benzyl)-1*H*-indole-2-carboxamide (**75**)

Compound was synthesized using *N*-(4-aminobenzyl)-5-cyclopropyl-1*H*-indole-2-carboxamide (0.2 g, 0.65 mmol) and fluoromethanesulfonyl chloride (86.1 mg, 0.65 mmol) according to general procedure F to afford a white solid (11 mg, 4%). ^1^H NMR (400 MHz, DMSO-*d*_6_): δ 11.43 (s, 1H), 10.33 (s, 1H), 8.94 (t, *J* = 5.0 Hz, 1H), 7.34–7.28 (m, 4H), 7.17 (d, *J* = 8.1 Hz, 2H), 7.05 (s, 1H), 6.92 (d, *J* = 8.5 Hz, 1H), 5.41 (d, *J* = 46.0 Hz, 2H), 4.45 (d, *J* = 5.6 Hz, 2H), 1.99–1.94 (m, 1H), 0.96–0.88 (m, 2H), 0.64–0.63 (m, 2H). LC–MS (*m*/*z*): 402.06 [M + H]^+^.

#### 5-Cyclopropyl-*N*-(4-((2,2,2-trifluoroethyl)sulfonamido)benzyl)-1*H*-indole-2-carboxamide (**76**)

Compound was synthesized using *N*-(4-aminobenzyl)-5-cyclopropyl-1*H*-indole-2-carboxamide (0.2 g, 0.656) and 2,2,2-trifluoroethane-1-sulfonyl chloride (120 mg, 0.65 mmol) according to general procedure F to afford a white solid (40 mg, 14%). ^1^H NMR (400 MHz, DMSO-*d*_6_): δ 11.42 (s, 1H), 10.42 (br.s., 1H), 8.94 (t, *J* = 5.9 Hz, 1H), 7.32–7.29 (m, 4H), 7.18 (d, *J* = 8.4 Hz, 2H), 7.05 (s, 1H), 6.92 (dd, *J* = 8.6 and 1.2 Hz, 1H), 4.49–4.22 (m, 4H), 1.98–1.94 (m, 1H), 0.91–0.88 (m, 2H), 0.65–0.63 (m, 2H). LC–MS (*m*/*z*): 450.1 [M – H]^−^.

#### *N*,1,5-Trimethyl-*N*-(4-(methylsulfonamido)benzyl)-1*H*-indole-2-carboxamide (**77**)

Compound was synthesized using 1,5-dimethyl-1*H*-indole-2-carboxylic acid (120 mg, 0.63 mmol) and *N*-(4-((methylamino)methyl)phenyl)methanesulfonamide hydrochloride (159 mg, 0.63 mmol) according to General Procedure C. The crude product was purified by FCC (0–5% CHCl_3_:MeOH) to yield the title compound as a colorless solid (141 mg, 58%).^1^H NMR (400 MHz, DMSO-*d*_6_): δ 9.47 (br. s., 1H), 7.34–7.39 (m, 2H), 7.21–7.28 (m, 4H), 7.08 (dd, *J* = 1.28, 8.38 Hz, 1H), 6.58 (s, 1H), 4.68 (s, 2H), 3.74 (s, 3H), 3.01 (s, 3H), 2.97 (s, 3H), 2.39 (s, 3H). HRMS (ESI): *m*/*z* [M + H]^+^ calcd for C_20_H_24_N_3_O_3_S 386.15329, found 386.15299.

#### *N*-(2-Hydroxyethyl)-1,5-dimethyl-*N*-(4-(methylsulfonamido)benzyl)-1*H*-indole-2-carboxamide (**78**)

Compound was synthesized using 1,5-dimethyl-1*H*-indole-2-carboxylic acid (120 mg, 0.63 mmol) and *N*-(4-(((2-hydroxyethyl)amino)methyl)phenyl)methanesulfonamide hydrochloride (178 mg, 0.63 mmol) according to General Procedure C. The crude product was purified by FCC (0–5% CHCl_3_:MeOH) to yield the title compound as a colorless solid (141 mg, 58%). ^1^H NMR (400 MHz, DMSO-*d*_6_): δ 9.47 (br. s., 1H), 7.32–7.38 (m, 2H), 7.19–7.30 (m, 4H), 7.06 (dd, *J* = 1.22, 8.56 Hz, 1H), 6.57 (s, 1H), 4.73 (s, 2H), 4.58 (t, *J* = 5.26 Hz, 1H), 3.71 (s, 3H), 3.54–3.61 (m, 2H), 3.48–3.54 (m, 2H), 2.97 (s, 3H), 2.39 (s, 3H). HRMS (ESI): *m*/*z* [M + H]^+^ calcd for C_21_H_25_N_3_O_4_S 416.16385, found 416.16354.

#### *N*-(2-Hydroxyethyl)-1,5-dimethyl-*N*-((6-morpholinopyridin-3-yl)methyl)-1*H*-indole-2-carboxamide (**79**)

Compound was synthesized using 1,5-dimethyl-1*H*-indole-2-carboxylic acid (120 mg, 0.63 mmol) and 2-(((6-morpholinopyridin-3-yl)methyl)amino)ethan-1-ol hydrochloride (174 mg, 0.63 mmol) according to General Procedure C. The crude product was purified by FCC (0–5% CHCl_3_:MeOH) to yield the title compound as a colorless solid (110 mg, 42%). ^1^H NMR (400 MHz, DMSO-*d*_6_): δ 8.08 (br. s., 1H), 7.51 (d, *J* = 8.07 Hz, 1H), 7.31–7.39 (m, 2H), 7.03–7.10 (m, 1H), 6.81 (d, *J* = 8.80 Hz, 1H), 6.56 (s, 1H), 4.65 (s, 2H), 3.65–3.74 (m, 7H), 3.54–3.61 (m, 2H), 3.47–3.54 (m, 2H), 3.40–3.47 (m, 4H), 2.39 (s, 3H). HRMS (ESI): *m*/*z* [M + H]^+^ calcd for C_23_H_29_N_4_O_3_ 409.22342, found 409.22284.

#### 1-(2-Hydroxyethyl)-*N*,5-dimethyl-*N*-(4-(methylsulfonamido)benzyl)-1*H*-indole-2-carboxamide (**80**)

Solid PTSA, monohydrate (13.0 mg, 0.68 mmol) was added to a solution of *N*,5-dimethyl-*N*-(4-(methylsulfonamido)benzyl)-1-(2-((tetrahydro-2*H*-pyran-2-yl)oxy)ethyl)-1*H*-indole-2-carboxamide (171 mg, 0.34 mmol) in methanol. The clear solution was stirred at room temperature for 2 h and then quenched by the addition of solid sodium acetate (8.42 mg, 0.10 mmol). Methanol was evaporated in vacuum and the evaporation residue was partitioned between EtOAc and water. After phase separation, the organic layer was washed with diluted HCl, saturated sodium bicarbonate solution, brine, dried over magnesium sulfate, filtrated, and concentrated to dryness in vacuum. Diethyl ether was added to the crude product and the mixture was kept in the fridge over the weekend. The ethereal phase was removed and the remaining solid was quickly rinsed with fresh diethyl ether. The solid was redissolved in acetone, the solvent was evaporated to dryness in vacuum and the remaining solid was resuspended in diethyl ether. The slurry was kept in a fridge overnight. The ethereal phase was removed, the remaining solid was rinsed with fresh diethyl ether and dried in vacuum to give a white, amorphous solid (125 mg, 88%). ^1^1H NMR (400 MHz, DMSO-*d*_6_) d 9.48 (s, 1H), 7.41 (d, *J* = 8.44 Hz, 1H), 7.35 (s, 1H), 7.27–7.32 (m, 2H), 7.22–7.26 (m, 2H), 7.05 (dd, *J* = 1.28, 8.50 Hz, 1H), 6.57 (s, 1H), 4.69 (s, 2H), 4.33 (t, *J* = 5.87 Hz, 2H), 3.67 (t, *J* = 5.87 Hz, 2H), 3.01 (s, 3H), 2.98 (s, 3H), 2.38 (s, 3H). HRMS (ESI): *m*/*z* [M + H]^+^ calcd for C_21_H_26_N_3_O_4_S 416.16385 found, 416.16338.

#### 1-(2-Hydroxyethyl)-*N*,5-dimethyl-*N*-((6-morpholinopyridin-3-yl)methyl)-1*H*-indole-2-carboxamide (**81**)

Solid para-toluenosulfonic acid monohydrate (65.8 mg, 0.34 mmol) was added to a solution of *N*,5-dimethyl-*N*-((6-morpholinopyridin-3-yl)methyl)-1-(2-((tetrahydro-2*H*-pyran-2-yl)oxy)ethyl)-1*H*-indole-2-carboxamide (155 mg, 0.31 mmol) in methanol. The clear solution was stirred at room temperature for 2 h and then quenched by the addition of solid sodium acetate (31.0 mg, 0.37 mmol). Methanol was evaporated in vacuum and the solid evaporation residue was partitioned between EtOAc and an aqueous sodium carbonate solution. After phase separation, the organic phase was washed with water, brine, dried over magnesium sulfate, filtrated, and concentrated in vacuum to give a white solid (62 mg, 48%). ^1^H NMR (500 MHz, Acetone-*d*_6_): δ 8.19 (br. s., 1H), 7.63 (br. s., 1H), 7.32–7.45 (m, 2H), 7.08 (d, *J* = 8.33 Hz, 1H), 6.81 (d, *J* = 8.80 Hz, 1H), 6.63 (s, 1H), 4.69 (br. s., 2H), 4.40–4.53 (m, 3H), 3.85 (q, *J* = 4.98 Hz, 2H), 3.70–3.76 (m, 4H), 3.45–3.52 (m, 4H), 3.14 (br. s., 3H), 2.39 (s, 3H). HRMS (ESI): *m*/*z* [M + H]^+^ calcd for C_23_H_29_N_4_O_3_ 409.22342, found 409.22294.

#### *N*-(2-(Dimethylamino)ethyl)-1,5-dimethyl-*N*-(4-(methylsulfonamido)benzyl)-1*H*-indole-2-carboxamide (**82**)

Compound was synthesized using *N*-(4-aminobenzyl)-*N*-(2-(dimethylamino)ethyl)-1,5-dimethyl-1*H*-indole-2-carboxamide (147 mg, 0.40 mmol) and methanesulfonyl chloride (46.2 mg, 0.40 mmol) using the same procedure above to yield the title compound as a white solid (120 mg, 67%). ^1^H NMR (250 MHz, CDCl_3_): δ 7.36 (s, 1H), 7.16–7.28 (m, 5H), 7.07–7.15 (m, 1H), 6.55 (s, 1H), 4.81 (s, 2H), 3.83 (s, 3H), 3.62 (t, *J* = 6.56 Hz, 2H), 2.97 (s, 3H), 2.37–2.67 (m, 5H), 2.18 (br. s., 6H). HRMS (ESI): *m*/*z* [M + H]^+^ calcd for C_23_H_31_N_4_O_3_S 443.21114, found 443.21101.

#### *N*-(Cyanomethyl)-1,5-dimethyl-*N*-((6-morpholinopyridin-3-yl)methyl)-1*H*-indole-2-carboxamide (**83**)

Compound was synthesized using 1,5-dimethyl-1*H*-indole-2-carboxylic acid (35.2 mg, 0.18 mmol) and 2-(((6-morpholinopyridin-3-yl)methyl)amino)acetonitrile hydrochloride (50 mg, 0.18 mmol) according to General Procedure A. The crude product was purified by FCC (EtOAc:Hexanes 50%) to yield the title compound as an off-white solid (30 mg, 40%). ^1^H NMR (500 MHz, CDCl_3_): δ 8.17 (s, 1H), 7.48 (d, *J* = 8.02 Hz, 1H), 7.42 (s, 1H), 7.29 (d, *J* = 8.65 Hz, 1H), 7.19 (d, *J* = 8.49 Hz, 1H), 6.76 (s, 1H), 6.67 (d, *J* = 8.80 Hz, 1H), 4.84 (s, 2H), 4.36 (s, 2H), 3.88 (s, 3H), 3.81–3.86 (m, 4H), 3.47–3.61 (m, 4H), 2.45 (s, 3H). HRMS (ESI): *m*/*z* [M + H]^+^ calcd for C_23_H_26_N_5_O_2_ 404.20810, found 404.20786.

#### 1,5-Dimethyl-*N*-(4-(methylsulfonamido)benzyl)-1*H*-pyrazole-3-carboxamide (**84**)

Compound was synthesized using 1,5-dimethyl-1*H*-pyrazole-3-carboxylic acid (80 mg, 0.57 mmol) and *N*-(4-(aminomethyl)phenyl)methanesulfonamide hydrochloride (176 mg, 0.74 mmol) according to General Procedure A. The crude product was purified by FCC (0–5% DCM:MeOH) to yield the title compound as a white solid (54 mg, 29%). ^1^H NMR (500 MHz, DMSO-*d*_6_) δ: 9.61 (s, 1H), 8.48 (t, *J* = 6.3 Hz, 1H), 7.24 (d, *J* = 8.3 Hz, 2H), 7.16–7.11 (m, 2H), 6.41 (d, *J* = 0.9 Hz, 1H), 4.33 (d, *J* = 6.3 Hz, 2H), 3.76 (s, 3H), 2.94 (s, 3H), 2.26 (s, 3H). HRMS (ESI): *m*/*z* [M + H]^+^ calcd for C_14_H_19_N_4_O_3_S 323.11724, found 323.11661.

#### 1,5-Dimethyl-*N*-((6-morpholinopyridin-3-yl)methyl)-1*H*-pyrazole-3-carboxamide (**85**)

Compound was synthesized using 1,5-dimethyl-1*H*-pyrazole-3-carboxylic acid (80 mg, 0.57 mmol) and (6-morpholinopyridin-3-yl)methanamine hydrochloride (157 mg, 0.62 mmol) according to General Procedure A. The crude product was purified by FCC (0–5% DCM:MeOH) to yield the title compound as a white solid (137 mg, 76%). ^1^H NMR (250 MHz, DMSO-*d*_6_) δ: 8.44 (t, *J* = 6.2 Hz, 1H), 8.06 (d, *J* = 2.4 Hz, 1H), 7.51 (dd, *J* = 8.7, 2.4 Hz, 1H), 6.78 (d, *J* = 8.7 Hz, 1H), 6.39 (d, *J* = 0.9 Hz, 1H), 4.24 (d, *J* = 6.2 Hz, 2H), 3.75 (s, 3H), 3.72–3.61 (m, 4H), 3.42–3.35 (m, 4H), 2.25 (s, 3H). HRMS (ESI): *m*/*z* [M + H]^+^ calcd for C_16_H_22_N_5_O_2_ 316.17680, found 316.17646.

#### 2,5-Dimethyl-*N*-(4-(methylsulfonamido)benzyl)oxazole-4-carboxamide (**86**)

Compound was synthesized using 2,5-dimethyloxazole-4-carboxylic acid (100 mg, 0.70 mmol) and *N*-(4-(aminomethyl)phenyl)methanesulfonamide hydrochloride (185 mg, 0.77 mmol) according to General Procedure A. The crude product was purified by FCC (0–5% DCM:MeOH) to yield the title compound as a white solid (164 mg, 72%). ^1^H NMR (250 MHz, CDCl3): δ 7.33 (d, *J* = 8.3 Hz, 2H), 7.18 (d, *J* = 8.5 Hz, 2H), 4.54 (d, *J* = 6.2 Hz, 2H), 2.99 (s, 3H), 2.61 (s, 3H), 2.39 (s, 3H). HRMS (ESI): *m*/*z* [M + H]^+^ calcd for C_14_H_18_N_3_O_4_S 324.10125, found 324.10088.

#### 2,5-Dimethyl-*N*-((6-morpholinopyridin-3-yl)methyl)oxazole-4-carboxamide (**87**)

Compound was synthesized using 2,5-dimethyloxazole-4-carboxylic acid (100 mg, 0.70 mmol) and (6-morpholinopyridin-3-yl)methanamine hydrochloride (179 mg, 0.77 mmol) according to General Procedure A. The crude product was purified by FCC (0–5% DCM:MeOH) to yield the title compound as a white solid (176 mg, 79%). ^1^H NMR (250 MHz, CDCl3): δ 8.15 (s, 1H), 7.51 (dd, *J* = 8.7, 2.4 Hz, 1H), 7.10 (s, 1H), 6.60 (d, *J* = 8.7 Hz, 1H), 4.43 (d, *J* = 6.0 Hz, 2H), 3.87–3.74 (m, 4H), 3.55–3.42 (m, 4H), 2.60 (s, 3H), 2.36 (s, 3H). HRMS (ESI): *m*/*z* [M + H]^+^ calcd for C_16_H_21_N_4_O3 317.16082, found 317.16054.

#### 3-Cyclopropyl-*N*-(4-(methylsulfonamido)benzyl)isoxazole-5-carboxamide (**88**)

Compound was synthesized using 3-cyclopropylisoxazole-5-carboxylic acid (101 mg, 0.66 mmol) and *N*-(4-(aminomethyl)phenyl)methanesulfonamide hydrochloride (203 mg, 0.85 mmol) according to General Procedure A. The crude product was purified by FCC (0–5% DCM:MeOH) to yield the title compound as a white solid (85 mg, 38%). ^1^H NMR (250 MHz, DMSO-*d*_6_): δ 9.67 (s, 1H), 9.36 (t, *J* = 6.1 Hz, 1H), 7.32–7.11 (m, 4H), 6.83 (s, 1H), 4.37 (d, *J* = 6.1 Hz, 2H), 2.95 (s, 3H), 2.15–2.00 (m, 1H), 1.10–0.99 (m, 2H), 0.87–0.74 (m, 2H). HRMS (ESI): *m*/*z* [M + H]^+^ calcd for C_15_H_18_N_3_O_4_S 336.10125, found 336.10075.

#### 3-Cyclopropyl-*N*-((6-morpholinopyridin-3-yl)methyl)isoxazole-5-carboxamide (**89**)

Compound was synthesized using 3-cyclopropylisoxazole-5-carboxylic acid (90 mg, 0.58 mmol) and (6-morpholinopyridin-3-yl)methanamine hydrochloride (162 mg, 0.70 mmol) according to General Procedure A. The crude product was purified by FCC (0–5% DCM:MeOH) to yield the title compound as a white solid (93 mg, 48%). ^1^1H NMR (250 MHz, DMSO-*d*_6_): δ 9.29 (t, *J* = 5.9 Hz, 1H), 8.08 (d, *J* = 2.4 Hz, 1H), 7.51 (dd, *J* = 8.7, 2.5 Hz, 1H), 6.86–6.75 (m, 2H), 4.29 (d, *J* = 5.9 Hz, 2H), 3.73–3.62 (m, 4H), 3.45–3.35 (m, 4H), 2.14–1.97 (m, 1H), 1.11–0.97 (m, 2H), 0.86–0.73 (m, 2H). HRMS (ESI): *m*/*z* [M + H]^+^ calcd for C_17_H_21_N_4_O_3_ 329.16082, found 329.16057.

#### *N*-(4-(Methylsulfonamido)benzyl)-3-phenylisoxazole-5-carboxamide (**90**)

Compound was synthesized using 3-phenylisoxazole-5-carboxylic acid (100 mg, 0.53 mmol) and N-(4-(aminomethyl)phenyl)methanesulfonamide hydrochloride (138 mg, 0.58 mmol) according to General Procedure A. The crude product was purified by FCC (0–5% DCM:MeOH) to yield the title compound as a beige solid (110 mg, 56%). ^1^H NMR (500 MHz, DMSO-*d*_6_): δ 9.68 (s, 1H), 9.54 (t, *J* = 6.0 Hz, 1H), 7.95–7.88 (m, 2H), 7.65 (s, 1H), 7.58–7.50 (m, 3H), 7.31 (d, *J* = 8.5 Hz, 2H), 7.18 (d, *J* = 8.5 Hz, 2H), 4.43 (d, *J* = 6.0 Hz, 2H), 2.96 (s, 3H). HRMS (ESI): *m*/*z* [M + H]^+^ calcd for C_18_H_18_N_3_O_4_S 372.10125, found 372.10081.

#### *N*-((6-Morpholinopyridin-3-yl)methyl)-3-phenylisoxazole-5-carboxamide (**91**)

Compound was synthesized using 3-phenylisoxazole-5-carboxylic acid (100 mg, 0.53 mmol) and (6-morpholinopyridin-3-yl)methanamine hydrochloride (131 mg, 0.57 mmol) according to General Procedure A. The crude product was purified by FCC (0–5% DCM:MeOH) to yield the title compound as a beige solid (100 mg, 52%). ^1^H NMR (500 MHz, DMSO-*d*_6_): δ 9.47 (t, *J* = 5.7 Hz, 1H), 8.12 (d, *J* = 2.2 Hz, 1H), 7.95–7.88 (m, 2H), 7.63 (s, 1H), 7.56 (dd, *J* = 8.8, 2.4 Hz, 2H), 7.54–7.51 (m, 2H), 6.82 (d, *J* = 8.7 Hz, 1H), 4.35 (d, *J* = 5.9 Hz, 2H), 3.71–3.65 (m, 4H), 3.43–3.37 (m, 4H). HRMS (ESI): *m*/*z* [M + H]^+^ calcd for C_20_H_21_N_4_O_3_ 365.16082, found 365.16063.

#### *N*-(4-(Methylsulfonamido)benzyl)-3-(pyridin-3-yl)isoxazole-5-carboxamide (**92**)

Compound was synthesized using 3-(pyridin-3-yl)isoxazole-5-carboxylic acid (50 mg, 0.26 mmol) and *N*-(4-(aminomethyl)phenyl)methanesulfonamide hydrochloride (63 mg, 0.26 mmol) according to General Procedure C. The crude product was purified by FCC (0–5% DCM:MeOH) to yield the title compound as a white solid (68 mg, 61%). ^1^H NMR (500 MHz, DMSO): δ 9.69 (s, 1H), 9.59 (t, *J* = 6.0 Hz, 1H), 9.12 (d, *J* = 1.6 Hz, 1H), 8.73 (dd, *J* = 4.8, 1.6 Hz, 1H), 8.36–8.28 (m, 1H), 7.76 (s, 1H), 7.63–7.54 (m, 1H), 7.31 (d, *J* = 8.5 Hz, 2H), 7.18 (d, *J* = 8.5 Hz, 2H), 4.44 (d, *J* = 6.0 Hz, 2H), 2.96 (s, 3H). HRMS (ESI): *m*/*z* [M + H]^+^ calcd for C_17_H_17_N_4_O_4_S 373.09650, found 373.09623.

#### *N*-((6-Morpholinopyridin-3-yl)methyl)-3-(pyridin-3-yl)isoxazole-5-carboxamide (**93**)

Compound was synthesized using 3-(pyridin-3-yl)isoxazole-5-carboxylic acid (78 mg, 0.41 mmol) and (6-morpholinopyridin-3-yl)methanamine hydrochloride (104 mg, 0.45 mmol) according to General Procedure C. The crude product was purified by FCC (0–5% DCM:MeOH) to yield the title compound as a white solid (100 mg, 67%). ^1^H NMR (250 MHz, DMSO-*d*_6_): δ 9.52 (t, *J* = 5.9 Hz, 1H), 9.11 (d, *J* = 1.6 Hz, 1H), 8.72 (dd, *J* = 4.8, 1.6 Hz, 1H), 8.31 (dt, *J* = 8.0, 1.8 Hz, 1H), 8.12 (d, *J* = 2.1 Hz, 1H), 7.74 (s, 1H), 7.64–7.50 (m, 2H), 6.82 (d, *J* = 8.7 Hz, 1H), 4.35 (d, *J* = 5.9 Hz, 2H), 3.74–3.63 (m, 4H), 3.46–3.35 (m, 4H). HRMS (ESI): *m*/*z* [M + H]^+^ calcd for C_19_H_20_N_5_O_3_ 366.15607, found 366.15566.

## Supporting Information Available

Full description of *in vitro* protocols (parasite and cell cultures, cytotoxicity, primary and secondary parasitology, parallel artificial membrane permeability, kinetic solubility, estimation of log *D*, human and liver microsomal and hepatocytes clearance, hERG, bioprofiling, metabolites identification) and *in vivo* studies (PK and animal models), synthesis of intermediates and additional compounds, NMR and HPLC purity profile (PDF)
Molecular formula strings with associated data (CSV)

## Abbreviations Used

CD: Chagas disease
NTD: neglected tropical diseases
WHO: World Health Organization
R&D: research and development
BZ: benznidazole
TPP: target product profile
DNDi: Drugs for Neglected Diseases initiative
LOLA: Lead Optimization Latin America
HLM: human liver microsomes
MLM: mouse liver microsomes
HFF-1: human foreskin fibroblasts cell line
EDG: electron-donating group
EWG: electron-withdrawing group
MDCK-MDR1: Madin-Darby canine kidney transferred with human MDR1 gene
LLE: lipophilic ligand efficiency
LogD: logarithm of distribution coefficient
TPSA: topological polar surface area
1-ABT: 1-aminobenzotriazole
PoC: proof-of-concept
BID: bis in die
QD: quaque die
DPI: days postinfection
Boc_2_O: *tert*-butoxycarbonyl
DIPEA: *N,N*-diisopropylethylamine
S_N_Ar: nucleophilic aromatic substitution
Het: heterocycle
Xphos: 2-dicyclohexylphosphino-2′,4′,6′-triisopropylbiphenyl
Xantphos: 4,5-bis(diphenylphosphino)-9,9-dimethylxanthene
*t*-BuXPhos: 2-di*tert*-butylphosphino-2′,4′,6′-triisopropylbiphenyl
HATU: 1-[bis(dimethylamino)methylene]-1*H*-1,2,3-triazolo[4,5-*b*]pyridinium 3-oxide hexafluorophosphate
DHP: 3,4-dihydro-2*H*-pyran
PTSA: *p*-toluenesulfonic acid
HBTU: 1-hydroxybenzotriazole hydrate
