# Supplementary material for: Discovery and Early Optimization of 1H-Indole-2-carboxamides with Anti-Trypanosoma cruzi Activity
Source: J Med Chem. 2025 Mar 31;68(7):7313–40. doi: 10.1021/acs.jmedchem.4c02942 (PMC11998015; doi:10.1021/acs.jmedchem.4c02942)
Supplement: Supplementary file 1 — jm4c02942_si_001.pdf [file jm4c02942_si_001.pdf]

Supporting Information

Discovery and early optimization of 1*H*-indole-2-carboxamides with anti-*Trypanosoma cruzi* activity

Ramon G. de Oliveira[1‡], Luiza R. Cruz[1,7‡], Marco A. Dessoy[1], Paul J. Koovits[1], Deborah A. dos Santos[1], Luiz F. N. de Oliveira[1], Rafael A. Ferreira[1], María C. Mollo[1], Eun Lee[1], Simone M. Duarte[2], Renata Krogh[2], Leonardo L. G. Ferreira[2], Rafael C. Chelucci[2], Maria Dichiara[3], Quillon J. Simpson[3], Clarissa Feltrin[5], Adriana C. da Silva[5], Benedito M dos Santos[5], Milena F. Broering[6], Michael P. Pollastri[3], Lori Ferrins[3,4], Carolina B. Moraes[5,6], Adriano D. Andricopulo[2], Jadel M. Kratz[7], Peter Sjö[8], Charles E. Mowbray[8], Luiz C. Dias[1*]

[1]Institute of Chemistry, State University of Campinas, Campinas, 13083-862, Brazil; [2]São Carlos Institute of Physics, University of São Paulo, São Carlos, 13563-120, Brazil; [3]Chemistry and Chemical Biology and [4]Pharmaceutical Sciences, Northeastern University, Boston, 02115, United States; [5]Institute of Biomedical Sciences and [6]School of Pharmaceutical Sciences, University of São Paulo, São Paulo, 05508-000, Brazil; [7]Drugs for Neglected Diseases initiative, Rio de Janeiro, 20010-020, Brazil; [8]Drugs for Neglected Diseases initiative, Geneva, 1202, Switzerland.

Corresponding Author

* Luiz Carlos Dias, ldias@unicamp.br, Institute of Chemistry, State University of Campinas, São Paulo, 13083-862, Brazil.

Table of Contents

Additional data S5
Metabolite Identification (MetID) studies S5
Metabolites of compound 1 in human and mouse S9 fractions S5
Metabolites of compound 2 in human and mouse S9 fractions S7
Experimental protocol S9
MDR1-MDCK permeability assay S11
Results S11
Experimental protocol S11
Solubility studies and melting point determination S12
Results S12
Experimental summary: Thermodynamic Solubility S12
Experimental summary: FaSSIF S12
Pharmacokinetics and Tolerability studies S13
Exposure of compound 1 in non-infected BALB/c mice S13
Exposure of compound 2 in non-infected BALB/c mice S14
Exposure of compound 3 in non-infected BALB/c mice S15
Experimental Protocol – Pharmacokinetics studies S15
Experimental Protocol – 5-day exploratory tolerability study S16
Intrinsic clearance (Clint) in mouse and human hepatocytes and microsomes S17
Results S17
Experimental protocol S17
Bioprofiling panel S19
Results S19
Experimental protocol S19
hERG channel blocking S20
Results S20

Experimental protocol S20
Compound 2 free exposure during acute and chronic efficacy studies in infected BALB/c mice S21
Results S21
*Tc*CYP51 inhibition S21
Results S21
Experimental protocol S22
Primary parasitology and cytotoxicity assays S23
Parasite and cell cultures S23
Compound solutions/dilutions S23
Cytotoxicity assays S23
Routine parasitology assay S23
1st tier ADME assays S25
Parallel artificial membrane permeability assay (PAMPA) S25
Microsomal stability S25
Kinetic solubility assay S25
Estimation of log D at pH 7.4 .... S26
Secondary parasitology and exploratory assays S26
High Content Screening of *T. cruzi* strains DTU-panel and time-kill assay S26
In vivo acute *T. cruzi* infection assay S27
In vivo chronic *T. cruzi* infection assay S28
Organic synthesis procedures S29
General procedures S29
General procedure A for the synthesis of substituted pyridyl nitriles. S30
General Procedure B for the reduction of nitriles to Boc-protected primary amines S30
General procedure C for N-Boc deprotection. S30
General procedure D for Sulfonylation. S30

General procedure E for the synthesis of indole derivatives. S31
General procedure F for ester hydrolysis. S31
General procedure G for the synthesis of reverse sulfonamides. S31
General procedure H for the synthesis of substituted isoxazole carboxylic acids. S31
General procedure I for amide coupling S32
General procedure J for amide coupling S32
General procedure K for urea derivatives. S33
Intermediates characterization S34
Additional compounds (intermediates and final compounds *S1-S54*) S89
NMR spectra of target compounds (1-93) S140
NMR spectra of additional compounds (*S1-S54*) S189
HPLC purity profile of selected compounds S216
References S218

Additional data

Metabolite Identification (MetID) studies

Metabolites of compound **1** in human and mouse S9 fractions

Summary: hydroxylation metabolites (M1, M5) were detected in both mouse and human. GSH (M2, M3) and glucuronide (M4) conjugates were detected in mouse only.

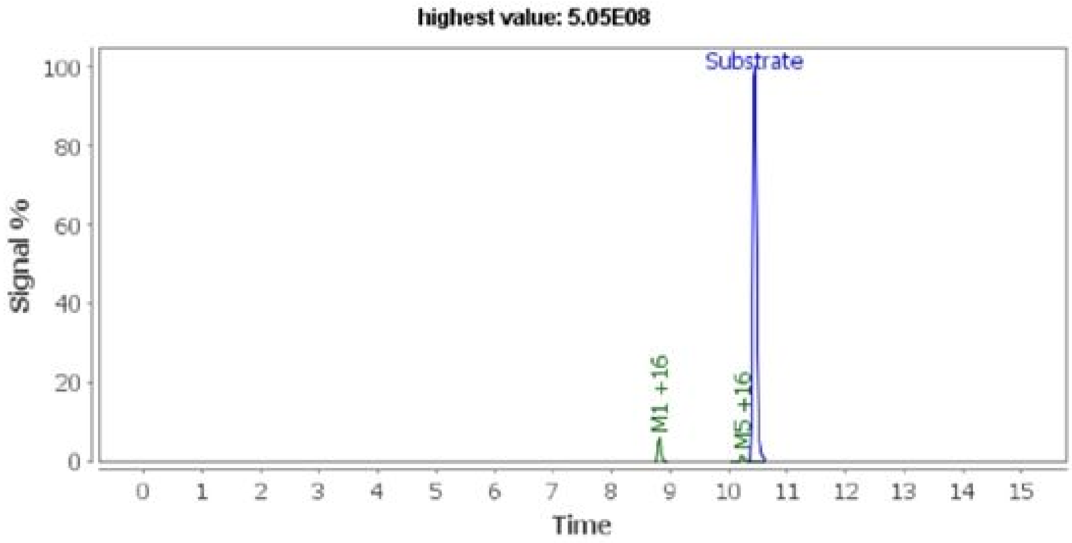

__Figure S1__: Metabolites of compound __1__ in human S9 fractions.

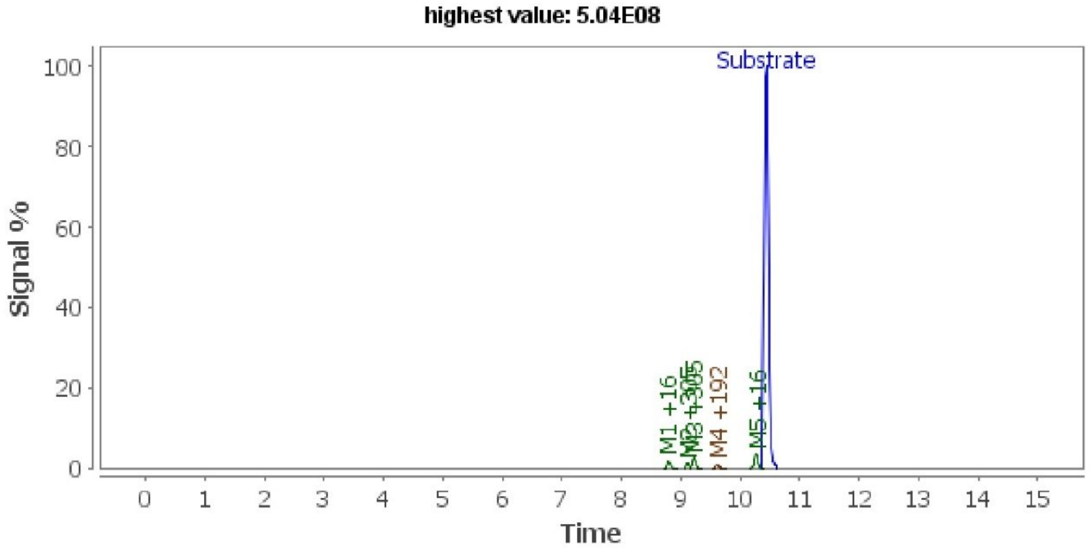

__Figure S2__: Metabolites of compound __1__ in mouse S9 fractions.

Metabolites:

Metabolite						
Peak name	Structure proposal	m/z	ppm	Area %	Conditions	comments
Substrate	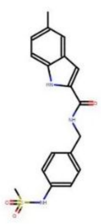	358.1236	-4.5<br>-4.3	91.03<br>93.85	Species=mouse<br>Species=human	
M1 +16<br>RT=8.80	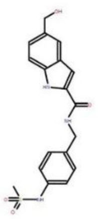	374.1190	-5.4<br>-5.6	1.53<br>4.60	Species=mouse<br>Species=human	
M5 +16<br>RT=10.28	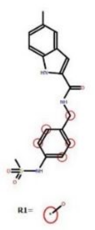	374.1188	-5.0<br>-5.1	1.55<br>3.14	Species=human<br>Species=mouse	
M3 +305<br>RT=9.22	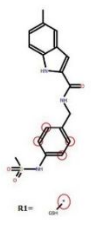	663.1928	-4.1	2.29	Species=mouse	
M2 +305<br>RT=9.13	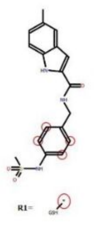	663.1931	-4.4	1.06	Species=mouse	
M4 +192<br>RT=9.61	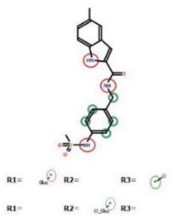	550.1512	-4.1	0.95	Species=mouse	

***Figure S3**: Metabolites of compound **1** in human and mouse S9 fractions.*

Metabolites of compound **2** in human and mouse S9 fractions

Summary: aliphatic hydroxylation (M1) and dehydrogenation (M3, M4) metabolites were detected in both mouse and human. An additional hydroxylation metabolite was detected in human only (M2).

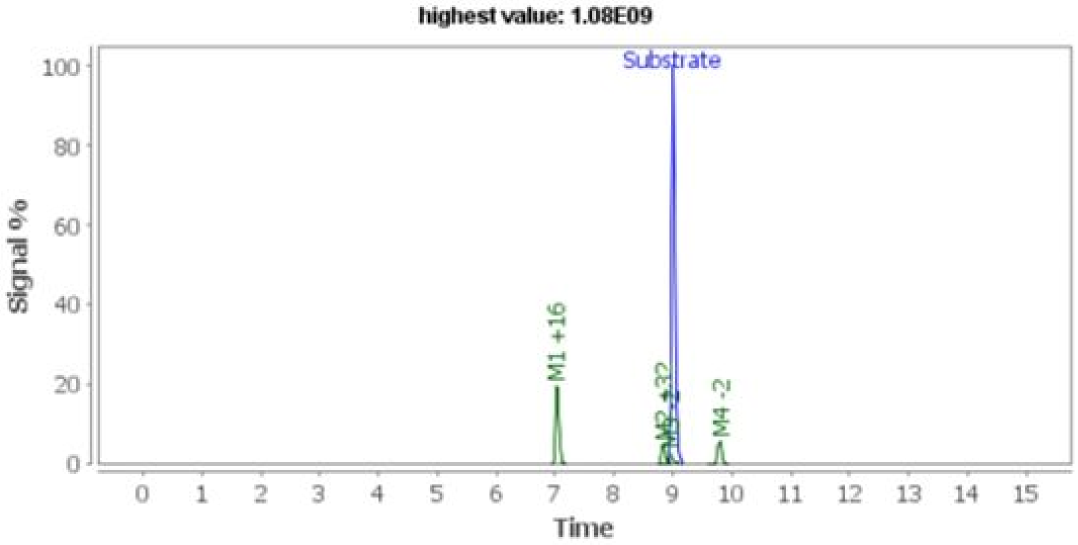

Figure S4: *Metabolites of compound **2** in human S9 fractions.*

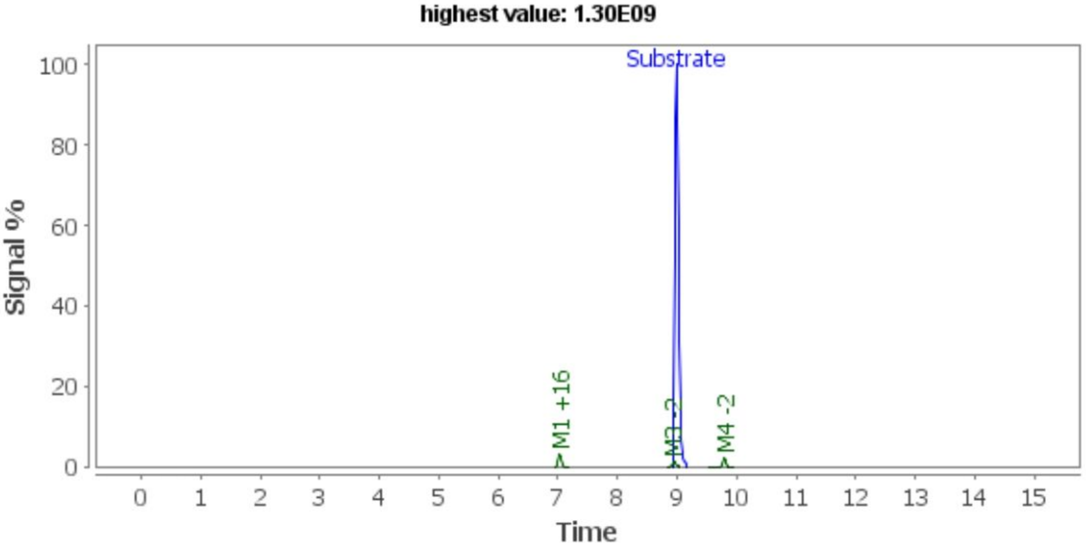

Figure S5: *Metabolites of compound **2** in mouse S9 fractions.*

Metabolites:

Metabolite						
Peak name	Structure proposal	m/z	ppm	Area %	Conditions	comments
Substrate	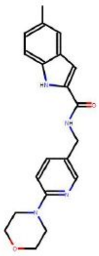	351.1831	-4.4<br>-4.6	93.06<br>74.33	Species=mouse<br>Species=human	
M1 +16<br>RT=7.02	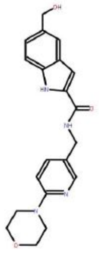	367.1787	-5.7<br>-6.2	14.95<br>3.01	Species=human<br>Species=mouse	
M4 -2<br>RT=9.80	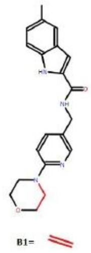	349.1676	-5.0<br>-4.8	2.21<br>4.44	Species=mouse<br>Species=human	
M2 +32<br>RT=8.83	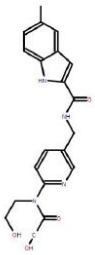	383.1733	-5.1	3.31	Species=human	
M3 -2<br>RT=8.96	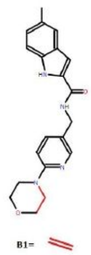	349.1676	-4.6<br>-5.0	1.72<br>2.97	Species=mouse<br>Species=human	

Figure S6*: Metabolites of compound* ***2*** *in human and mouse S9 fractions.*

Experimental protocol

Working solutions:

- 500 mM potassium phosphate buffer

  20 mg/mL liver S9
- 1 mM compound (see dilution below)
- 10 mM NADPH in water
- 50 mM UDPGA in water
- 50 mM GSH in water
- 50 mM $MgCl_2$ in water
- 10 mg/mL Alamethicin in methanol

Compound dilution:

Add 20 µL of 1:2 MeOH/H_2O (*v/v*) to 5 µL of 5 mM substrate in DMSO

Cofactors (fresh from powder each day)

NADPH stock: Add 120 µL water for every 1 mg of NADPH

UDPGA stock: Add 31.6 µL water for every 1 mg of UDPGA

GSH stock: Add 65.5 µL water for every 1mg of GSH

Incubation mixture (per sample):

40 µL 500 mM potassium phosphate buffer pH 7.4

20 µL 20 mg/mL liver S9

13.2 µL 50 mM $MgCl_2$

0.2 µL Alamethicin

20 µL 50 mM UDPGA

20 µL 10 mM NADPH

20 µL 50 mM GSH

2 µL 1 mM compound

64.6 µL H_2O

Incubation Mixture Preparation

Step 1: 20 µL of H_2O/MeOH 2:1 (v/v) was added to 5 µL of 5 mM substrate.
Step 2: 0.2 µL of Alamethicin was added to 20 µL of liver S9 and incubated on ice for 15 minutes.

Step 3: 2 µL of substrate was added to each well that will contain incubation mixture. (0h, 1h)

Step 3: Total KPO_4, $MgCl_2$, S9 mix (from step 2) and H_2O were combined.

Step 4: For 0h controls, 200 µL of MeCN/MeOH 1:1 was added to control wells.

Step 5: 138 µL of incubation mixture was aliquoted to 2 wells for each compound. (0h, 1h)

Step 6: An equal volume of each cofactor (UDPGA, NADPH, and GSH) were pooled.

Step 7: For 0h and 1h incubations, 60 µL of pooled cofactors was added and then incubated at 37 °C for 1h.

Step 8: After 1 hour, the 1h incubations were quenched with 200 µL of MeCN/MeOH 1:1.

Step 9: After quench, samples were centrifuged at 3200 rpm for 30 minutes and supernatant was transferred to plate or HPLC vials.

Step 10: After centrifugation, samples were injected on LC-MS or stored at -20 °C.

MDR1-MDCK permeability assay

Results

Table S1: *Parallel Artificial Membrane Permeability Assay (PAMPA) and MDR1-MDCK permeability assays.*

	PAMPA	MDR1-MDCK		
Entry	P_e (10^{-6} cm/s)	P_{app} (10^{-6} cm/s)		Efflux Ratio
		A to B	B to A	
**2**	18	28.43	17.75	0.62
**3**	0.8	6.69	11.15	1.67

Experimental protocol

MDR1-MDCK cells (obtained from Piet Borst at the Netherlands Cancer Institute) were seeded onto polyethylene membranes (PET) in 96-well insert systems at 2.5 x 10^5 cells/ mL until to 4-7 days for confluent cell monolayer formation. Test compounds were diluted with the transport buffer (HBSS with 10 mM Hepes, pH7.4) from DMSO stock solution to a concentration of 2 µM (DMSO<1%) and applied to the apical or basolateral side of the cell monolayer. Permeation of the test compounds from A to B direction or B to A direction was determined in duplicate. Reference compounds: digoxin was tested at 10 µM from A to B direction or B to A direction as well, while nadolol and metoprolol were tested at 2 µM in A to B direction in duplicate. The plate was incubated for 2.5 hours in CO_2 incubator at 37±1 °C, with 5% CO_2 at saturated humidity without shaking. In addition, the efflux ratio of each compound was also determined. Test and reference compounds were quantified by LC/MS/MS analysis based on the peak area ratio of analyte/IS. After transport assay, Lucifer yellow rejection assay are applied to determine the cell monolayer integrity. Buffers are removed from both apical and basolateral chambers, followed by the addition of 75 µL of 100 µM lucifer yellow in transport buffer and 250 µL transport buffer in apical and basolateral chambers, respectively. The plate is incubated for 30 minutes at 37 °C with 5% CO_2 and saturated humidity without shaking. After 30 minutes incubation, 20 µL of lucifer yellow samples are taken from the apical sides, followed by the addition of 60 µL of Transport Buffer. And then 80 µL of lucifer yellow samples are taken from the basolateral sides. The relative fluorescence unit (RFU) of lucifer yellow is measured at 425/528 nm (excitation/emission) with an Envision plate reader.

PAMPA protocol is detailed below.

Solubility studies and melting point determination

Results

Table S2*: Determination of kinetic and thermodynamic solubilities and melting point for selected compounds.*

	**1**	**2**	**3**	**24**	**41**	**56**	**73**
**Kinetic solubility PBS, pH 7.4** (µg/mL)	5.6	7.2	0.5	0.62	40	4.0	>16.7
**Thermodynamic solubility PBS, pH 7.4** (µg/mL)	1.4	1.3	3.1	ND	19.2	0.8	0.3
**Thermodynamic solubility FaSSIF, pH 6.5** (µg/mL)	9.4	11.1	0.02	ND	34.9	5.9	2.3
**Melting Point** (triplicate, ºC)	ND	218.1	ND	242.2	247.4	134.6	172.5

FaSSIF: fasted-state simulated intestinal fluid. Melting point measured using a M-565 Melting Point apparatus, Buchi.

Experimental summary: Thermodynamic Solubility

Test system	Thermodynamic solubility in PBS pH 7.4
Test concentration	1 mg/mL (n=2)
Incubation	Continuous shaking at 25 °C for 24 h
Detection	LC-MS/MS
Reference standard	Diethylstilbesterol, Haloperidol, Diclofenac sodium (any two)
Data type	Solubility (mg/mL)

Experimental summary: FaSSIF

Test system	Thermodynamic solubility in FaSSIF
Test concentration	1 mg/mL (n=2)
Incubation	Continuous shaking at 25 °C for 24 h
Detection	LC-MS/MS
Reference standard	Diethylstilbesterol, Haloperidol, Diclofenac sodium (any two)
Data type	Solubility (mg/mL)

Pharmacokinetics and Tolerability studies

Table S3: *Single dose (PO and IV) exposure in non-infected BALB/c mice of compounds **1**, **2** and **3**.*

Entry		Dose	AUC all[a]	AUC inf[b]	Tmax[c]	Cmax[d]	$t_{1/2}$[e]	Vss[f]	Clearance[g]	mPPB[h]	PS[i]
**1**	IV	2 mg/kg	504	497	0.08	429	0.60	2.94	57	96.2	91.1
	PO	50 mg/kg	35772	34127	0.25	8184	1.21	-	-		
**2**	IV	2 mg/kg	1146	1460	0.08	1552	0.62	1.1	24	98.0	84.2
	PO	50 mg/kg	30751	30875	1.0	3750	3.2	-	-		
	PO	50 mg/kg + 1-ABT	65059	65062	2.0	4403	1.6	-	-		
**3**	IV	2 mg/kg	710	698	0.08	829	0.58	1.80	35.7	98.2	89.8
	PO	50 mg/kg	4762	4398	0.25	2068	1.78				
	PO	50 mg/kg + 1-ABT	12657	12255	0.25	3778	1.11				

[a]Calculated area under the plasma concentration versus time curve calculated (h.ng/mL); [b]Inferred AUC from zero to time infinity (h.ng/mL); [c]Time of maximum exposure (h); [d]Concentration of maximum exposure (ng/mL); [e]Half-life (h); [f]Volume of distribution at steady state (L/kg); [g]Clearance (mL/min/kg); [h]Mouse plasma protein binding (% bound); [i]Mouse Plasma stability (% remaining after 6 h).

Exposure of compound **1** in non-infected BALB/c mice

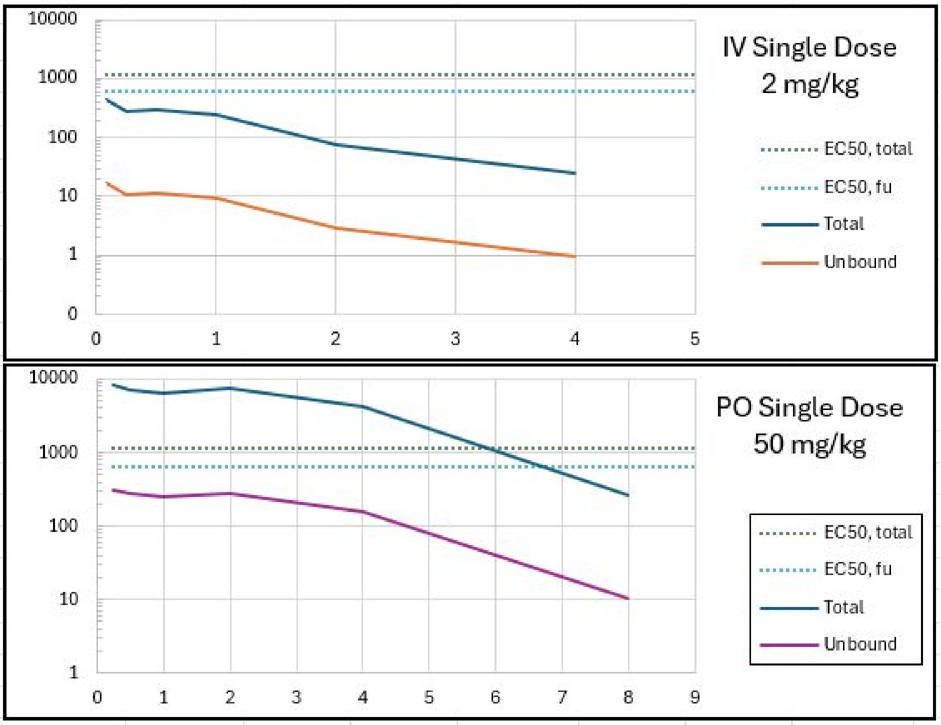

Figure S7: *Plasma exposure of compound **1** in non-infected BALB/c mice after a single dose of 2 mg/kg (IV) and 50 mg/kg (PO) with and without ABT pre-treatment.* EC_{50} *total = 1151 ng/mL;* EC_{50} *free = 626.2 ng/mL (corrected for medium protein binding of 45.6% bound).*

Exposure of compound **2** in non-infected BALB/c mice

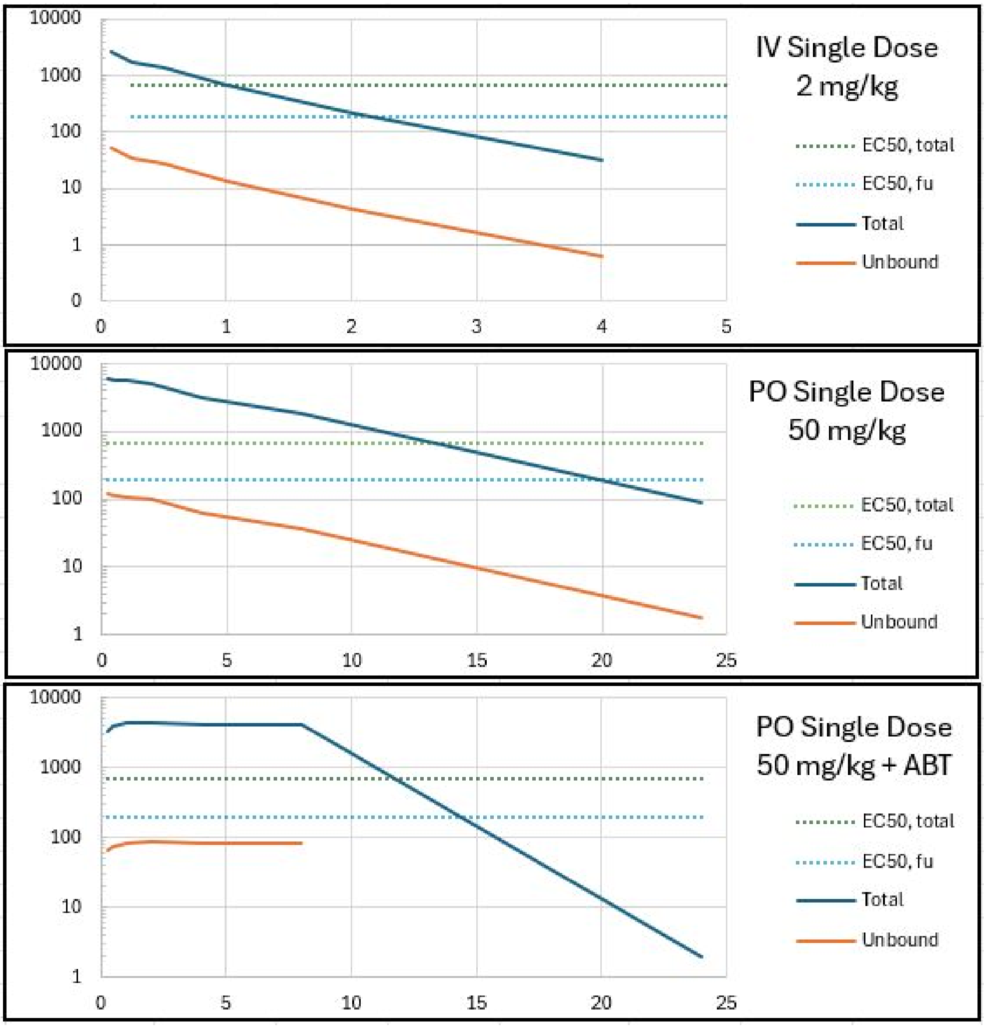

Figure S8: *Plasma exposure of compound* **2** *in non-infected BALB/c mice after a single dose of 2 mg/kg (IV) and 50 mg/kg (PO) with and without ABT pre-treatment.* EC_{50} *total = 680 ng/mL;* EC_{50} *free = 195.1 ng/mL (corrected for medium protein binding of 71.3% bound)*

Exposure of compound **3** in non-infected BALB/c mice

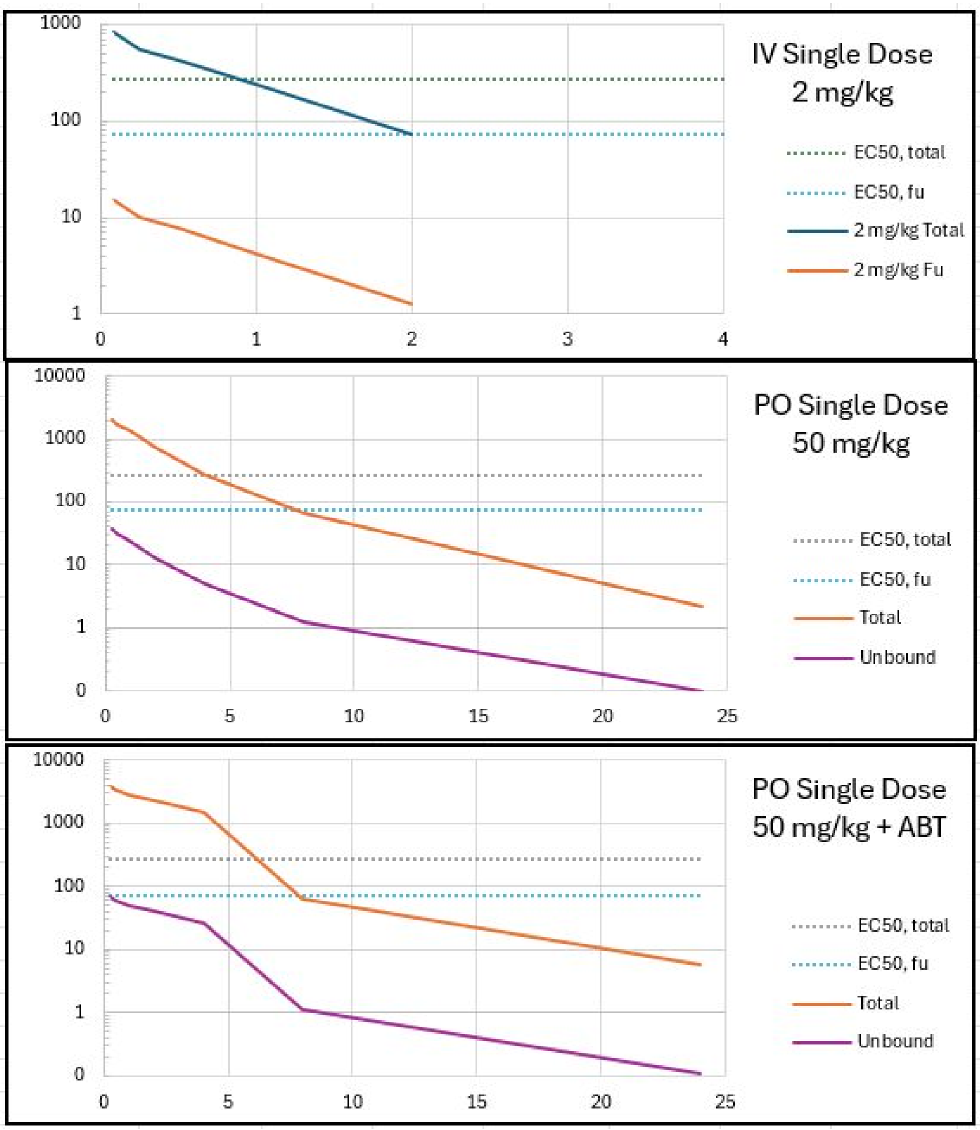

Figure S9*: Plasma exposure of compound **3** in non-infected BALB/c mice after a single dose of 2 mg/kg (IV) and 50 mg/kg (PO) with and without ABT pre-treatment.* ***EC_{50} total = 268 ng/mL. EC_{50} free = 73.8 ng/mL (corrected for medium protein binding of 72.5% bound)***

Experimental Protocol – Pharmacokinetics studies

All animal experiments were performed according to institutional ethical guidelines for animal care. Pharmacokinetic studies (CIEnP, Florianópolis, Brazil) were approved by the internal animal ethical committee (protocol numbers 217/01 and 264/00). All mice used in the experimental procedure were maintained under Specific Pathogen-Free conditions, 12h dark cycle, and ad libitum feeding.

Compounds were administered to groups (n = 6) of female BALB/c mice via single intravenous dosing (at 2 mg/kg) or single oral dosing (at 50 mg/kg) employing a solution/suspension vehicle comprising of 10% ethanol, 40% PEG400 and 0.4% Tween80 in saline buffer (pH adjusted to

9) for the IV dosing and 100% PEG400 for the PO dosing. When relevant, the CYP450 complex inhibitor 1-aminobenzotriazole (ABT) was administered at 100 mg/kg PO (formulated in 0.9% NaCl) at 17h before compounds administration.

Blood samples were collected from mandibular vein or mouse tail snip at 0.25, 1, 2, 4, 8, and 24 h for oral treatments or at 0.083, 0,25, 0,5, 1, 2 e 4 h for intravenous treatments. After processing, plasma samples were obtained and quantified by LC−MS/MS using fit-for-purpose analytical methods. Data analysis and calculation of pharmacokinetic parameters was performed using Phoenix WinNonlin version 7.0.

Experimental Protocol – 5-day exploratory tolerability study

All animal experiments were performed according to institutional ethical guidelines for animal care. Mouse tolerability study (CIEnP, Florianópolis, Brazil) was approved by the internal animal ethical committee (protocol number 241). All mice used in the experimental procedure were maintained under Specific Pathogen-Free conditions, 12h dark cycle, and ad libitum feeding.

Daily doses of compound **2** at 50 mg/kg, 100 mg/kg, and 200 mg/kg (100% PEG400 as vehicle) were administrated for 5 consecutive days in female, 8-12 weeks old, BALB/c mice (n=3/group) by intragastric route (gavage). During the treatment mice's body weight and clinical parameters (vocalization, irritability, piloerection, body and fur appearance, nasal-ocular discharge, tail pinch, lethargy, respiration labored/ decreased, and heartbeat) were assessed. All mice were culled and dissected after four hours of the last treatment dose. The brain, lungs, liver, thymus, adrenal gland, kidney, heart, uterus, ovary, and spleen were evaluated macroscopically, and the weight of each organ was registered and compared with the vehicle group.

Intrinsic clearance (Clint) in mouse and human hepatocytes and microsomes

Results

Table S4: *Hepatocytes and microsomal clearance of compounds **1**, **2**, and **3**.*

	**1**	**2**	**3**
**Hepatocytes Clerance** - µL/min/10^6 cells			
Human Hepatocytes – no inhibitor	11.5	18.2	6.33
Human Hepatocytes – with CYP3A4 inhibitor (azamulin)	11.4	19.6	5.56
Human Hepatocytes – with pan-CYP inhibitor	1.99	3.32	2.23
Mouse Hepatocytes – no inhibitor	4.26	8.49	4.52
**Microsomal Clearance** - µL/min/mg			
Human Liver Microsomes (HLM)	21	43	16
Mouse Liver Microsomes (MLM)	34	27	49

Experimental protocol

Test compounds are incubated with hepatocytes of different species (mouse and human) in 384-well plates. Samples are quenched at 6 time points and analyzed by LC-MSMS to determine the half-life and intrinsic clearance. In the HT-chemical inhibition assay, hepatocytes are pre-incubated with inhibitor for 30 min before the addition of hepatocytes to the compound plate. HT-chemical inhibition should always be run in conjunction with the uninhibited hepatocyte, all prepped simultaneously to ensure they are as similar as possible.

Hepatocytes are prepared by thawing in appropriate media and diluting to 1,000,000 viable cells/mL For the chemical inhibition arms, human hepatocytes are incubated for 30 minutes with inhibitors prior to the beginning of the assay.

- CYP3A4 specific inhibitor - Azamulin is added to 5 µM (2.5 µM when added to time point plates)
- PanCYP inhibitor - 1-ABT and Tienilic acid are added to 1 mM and 3 µM (0.5 mM and 1.5 µM when added to the time point plates)

Compounds are received in 384-well plates (95 nL, 10m M) and solubilized in Incubation Media (supplement Williams E Media with 0.5 mL per 50 mL of total volume of 200 mM GlutaMAX (L-Glutamine can be used if GlutaMAX is unavailable; 2 mM final concentration) and 0.75 mL of 1 M HEPES per 50 mL of total volume (15 mM final concentration) warmed to 37ºC). Through a series of dilutions, a 2X solution of test article of 1 µM is obtained. 12.5 µL of solution is robotically transferred to 6 384-well plates that will be the time point plates. The final test article concentration 0.5 µM

The assay is started for all but the T0 plate by adding 12.5 μL of hepatocytes to each well (500,00 cells/mL in the assay). For the T0, organic is added to the plate prior to hepatocyte addition. The other time points are T15, T30, T60, T120 and T240 minutes incubated for appropriate times in a 37 ^{o}C, 85% humidity, 5% CO_2 incubator with shaking. The assay is stopped by adding 75 μL of quench solution (95:5 acetonitrile: methanol containing 75 nM carbutamide as an internal standard) at the appropriate times. Plates are centrifuged for 15 min at 3000 rpm and analyzed by LC-MSMS to detect test article loss over time.

Bioprofiling panel

Results

***Table S5**:Bioprofiling of compounds **1**, **2**, and **3** against a panel of known off-targets.*

Bioprofiling panel	1	2	3
Bioprofiling 6pt Antag__5-HT2A CHO Human Serotonin (5-HT) - IC_{50}	1.8	8.4	2.9
Bioprofiling 6pt Antag__Cav1.2 (L-type) ND7/23 Mouse/Rat KCl - IC_{50}	> 10	> 10	> 10
2nd Messenger__ADORA1 CHO Calcium Agonist Activation - IC_{50}	> 10	> 10	> 10
2nd Messenger__CHRM2 CHO Calcium Agonist Activation - IC_{50}	> 10	> 10	> 10
2nd Messenger__DRD2 CHO Calcium Agonist Activation - IC_{50}	> 10	> 10	> 10
2nd Messenger__HTR1A CHO Calcium Agonist Activation - IC_{50}	> 10	> 10	> 10
2nd Messenger__HTR2A CHO Calcium Agonist Activation - IC_{50}	> 10	> 10	> 10
2nd Messenger__HTR2B CHO Calcium Agonist Activation - IC_{50}	> 10	> 10	> 10
2nd Messenger__OPRM1 CHO Calcium Agonist Activation - IC_{50}	> 10	> 10	> 10
Bioprofiling 6pt Ag__Alpha1A CHO Human - EC_{50}	> 10	> 10	> 10
Bioprofiling 6pt Ag__Alpha2A CHO Human - EC_{50}	> 10	> 10	> 10
Bioprofiling 6pt Ag__B2 CHO Human - EC_{50}	> 10	> 10	> 10
Bioprofiling 6pt Ag__Beta1 CHO Human - EC_{50}	> 10	> 10	> 10
Bioprofiling 6pt Ag__CB1 CHO Human - EC_{50}	> 10	> 10	> 10
Bioprofiling 6pt Ag__CHO Hamster - EC_{50}	> 10	> 10	> 10
Bioprofiling 6pt Ag__ETA CHO Human - EC_{50}	> 10	> 10	> 10
Bioprofiling 6pt Ag__ETB CHO Human - EC_{50}	> 10	> 10	> 10
Bioprofiling 6pt Ag__H1 CHO Human - EC_{50}	> 10	> 10	> 10
Bioprofiling 6pt Antag__5-HT1A CHO Human 5-Carboxytryptamine (5-CT) - IC_{50}	> 10	> 10	> 10
Bioprofiling 6pt Antag__5-HT2B CHO Human Serotonin (5-HT) - IC_{50}	> 10	> 10	> 10
Bioprofiling 6pt Antag__A1 CHO Human N6-Cyclopentyladenosine (CPA) - IC_{50}	> 10	> 10	> 10
Bioprofiling 6pt Antag__Alpha1A CHO Human A-61603 - IC_{50}	> 10	> 10	> 10
Bioprofiling 6pt Antag__Alpha2A CHO Human UK-14304 - IC_{50}	> 10	> 10	> 10
Bioprofiling 6pt Antag__B2 CHO Human Bradykinin - I IC_{50}	> 10	> 10	> 10
Bioprofiling 6pt Antag__Beta1 CHO Human Norepinephrine - IC_{50}	> 10	> 10	> 10
Bioprofiling 6pt Antag__CB1 CHO Human WIN 55,212-2 - IC_{50}	> 10	> 10	> 10
Bioprofiling 6pt Antag__D2L CHO Human Quinpirole - IC_{50}	> 10	> 10	> 10
Bioprofiling 6pt Antag__ETA CHO Human Endothelin-1 (ET-1) - IC_{50}	> 10	> 10	> 10
Bioprofiling 6pt Antag__ETB CHO Human BQ-3020 - IC_{50}	> 10	> 10	> 10
Bioprofiling 6pt Antag__H1 CHO Human Histamine - IC_{50}	> 10	> 10	> 10
Bioprofiling 6pt Antag__M2 CHO Human Carbachol - IC_{50}	> 10	> 10	> 10
Bioprofiling 6pt Antag__Opioid mu CHO Human DAMGO - IC_{50}	> 10	> 10	> 10
Bioprofiling 6pt Antag__P2Y CHO Hamster ATP - IC_{50}	> 10	> 10	> 10
Bioprofiling 6pt Antag__PDE3A Human cAMP - IC_{50}	> 10	> 10	> 10
Bioprofiling 6pt Antag__PDE4B2 Human cAMP - IC_{50}	> 10	> 10	> 10

Experimental protocol

Compounds were evaluated across a panel of 20 liability targets (37 functional assays) which included functional cell-based GPCRs and ion channels in both agonist and antagonist readout, measuring calcium flux, and biochemical functional assays for nuclear hormone receptors and

phosphodiesterases using a TR-FRET format, in a 6-point,1:3 dilution dose-response (top concentration of 10 μM final).

hERG channel blocking

Results

Table S6: *hERG inhibition of compounds **1**, **2**, and **3**.*

**hERG assay**	**1**	**2**	**3**
Thallium Influx Antag__Kv11.1 (hERG) CHO Human KCl - IC_{50} (μM)	4.56	> 30	>30

Experimental protocol

The method used was a modification of the method described previously[1]. Briefly, hERG functional activity was measured in an inducible hERG T-REx-CHO Cell line (ThermoFisher #K1237) using thallium influx as a surrogate indicator of potassium ion channel activity. Thallium enhances the fluorescent signal of BTC-AM dye (ThermoFisher #B6791). 384-well plates were seeded at 15,000 cells/well with doxycycline hyclate included to induce expression of the hERG channel and grown for 48 hours. Media was removed and cells were loaded with 4 uM dye for 90 mins in a low potassium buffer, then the dye removed, and compounds added to the cells in a high potassium buffer in a 6 points1:3 dilution dose response (top concentration of 30 uM final). After 30 mins of compound incubation, channel activity was recorded upon addition of thallium buffer using a Tetra plate reader. The slope of the kinetic read was used to calculate channel activity.

Compound 2 free exposure during acute and chronic efficacy studies in infected BALB/c mice

Results

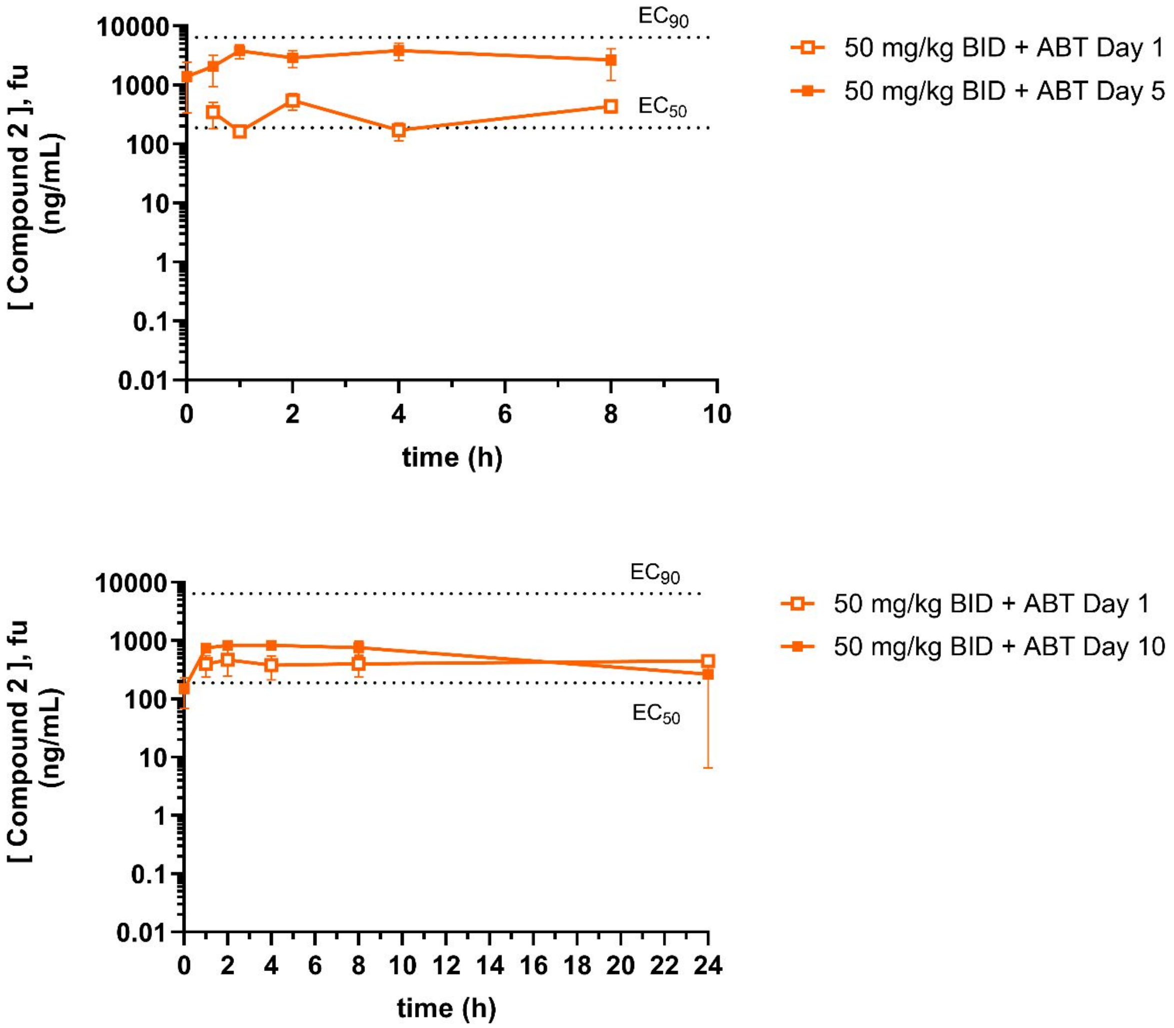

Figure S10*: Free whole blood exposure of compound* ***2*** *during efficacy studies in the acute (top) and chronic (bottom) Chagas BLI mouse models (BALB/c mice infected with T. cruzi). For the compound quantification in whole blood samples the same analytical method developed and validated for the PK study was used.*

*Tc*CYP51 inhibition

Results

Table S7*: TcCYP51 inhibition data for selected compounds.*

Compound	*T. cruzi* pEC50	*Tc*CYP51 pIC50	Compound	*T. cruzi* pEC50	*Tc*CYP51 pIC50
1	5.6	5.3	**24**	6.5	6.4
2	5.7	5.9	**29**	4.2	5.2
3	6.2	5.7	**37**	6.9	6.1

**4**	6.2	5.7	**42**	5.4	4.7
**7**	5.5	4.2	**65**	4.4	<4.0
**10**	4.2	5.2	**73**	5.8	5.0
**21**	5.5	<4.0	**88**	5.4	<4.0
**22**	5.8	5.0	**89**	4.8	<4.0
**23**	5.2	4.3			

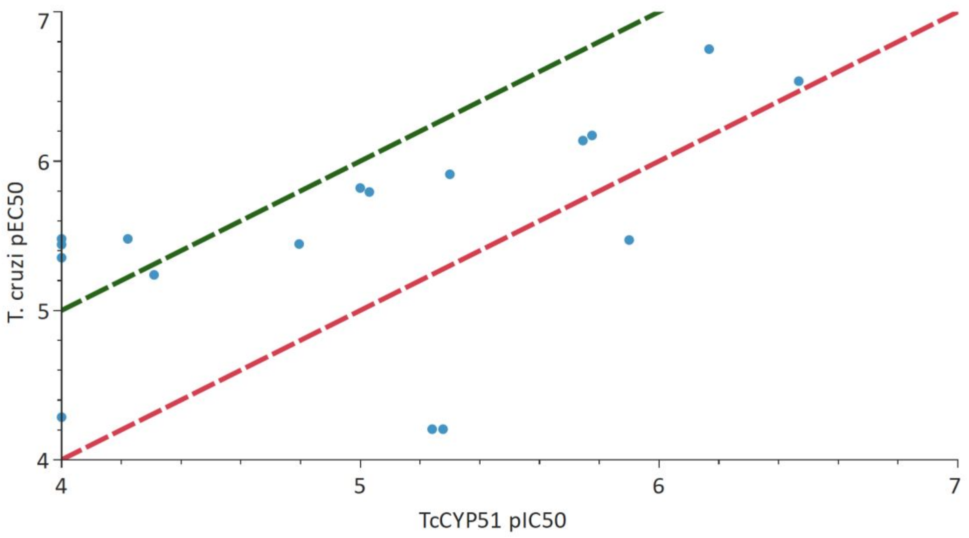

Figure S11: *Scatter plot of T. cruzi intracellular activity (pEC$_{50}$) versus inhibition of T. cruzi CYP51 (pIC$_{50}$) for selected compounds (listed in Table S7). Red line: T. cruzi pEC$_{50}$ = TcCYP51 pIC$_{50}$; green line: 10-fold selectivity.*

Experimental protocol

As previously described.[2]

Primary parasitology and cytotoxicity assays

Parasite and cell cultures

Trypanosoma cruzi, Tulahuen CL2 beta-galactosidase strain was used for the routine antiparasitic assay. The strain was maintained on HFF-1 (human foreskin fibroblast) cells in DMEM medium, supplemented with 200 mM L-glutamine, 16.5 mM sodium bicarbonate ($NaHCO_3$), and 10% inactivated fetal calf serum (FCS). All cultures and assays were conducted at 37 ˚C under an atmosphere of 5% CO_2.

Compound solutions/dilutions

Compound stock solutions were prepared in neat dimethyl sulfoxide (DMSO) at 20 mM. Compounds were serially pre-diluted (2-fold) in DMSO followed by a further (intermediate) dilution in DMEM to assure a final in-test DMSO concentration of <1%. Compounds were tested at 2-fold compound dilutions covering a range of 64 down to 0.00024 µM.

Cytotoxicity assays

HEPG2 and HFF-1 cells were incubated in DMEM medium without phenol red, supplemented with 10% fetal bovine serum and 1% antibiotics (penicillin-streptomycin), at a concentration of $4.0x10^3$ cells/well, in 96-well culture plates. After 24 hours, the compounds were added at concentrations of 0.12-64 µM in duplicate. Doxorubicin was used as a positive control at concentrations of 0.1-20 µM, and 1% DMSO was used as a negative control, the plates were incubated for 72 hours (37 ˚C in 5% CO_2). After the incubation time, 15 µL MTS was added to the wells and incubated for 4 hours at 37 ˚C. The absorbance at 490 nm was measured in a spectrophotometer to evaluate the reduction of MTS by viable cells. All experiments were performed in duplicate.

Routine parasitology assay

Assays were performed in sterile 96-well microtiter plates, each well containing 10 µL of the compound dilutions together with 100 µL of HFF-1 cell/parasite inoculum ($4x10^3$ cells/well + $4x10^4$ parasites/well). Cell growth was compared to untreated-infected controls (100% growth) and non-infected (0% growth) after 4 days of incubation at 37 °C and 5% CO_2. Parasite burdens

were assessed after adding the substrate CPRG (chlorophenol red β-d-galactopyranoside): 50 μL/well of a stock solution containing 15.2 mg CPRG + 250 μL Nonidet in 100 mL PBS. The change in color was measured spectrophotometrically at 570 nm after 4 h incubation at 37 °C. The results were expressed as a % reduction in parasite burdens compared to control wells and an IC_{50} (50% inhibitory concentration) was calculated. Benznidazole was included as the reference drug (EC_{50} ~2-5 μM).

1st tier ADME assays

Parallel artificial membrane permeability assay (PAMPA)

To determine the passive permeability of the compounds, a 96-well plate containing pre-coated membranes was used (Corning Gentest # 353015). The solutions of the compounds were prepared by diluting the stock solutions (10 mM) in phosphate buffered saline (PBS) pH 6.5 at a final concentration of 10 μM. The solutions diluted in PBS pH 6.5 were then added to the donor portion of the plate (300 μL/well), while in the acceptor portion PBS pH 7.4 (200 μL/well) was added. The two portions of the plate were then coupled, and the system was incubated for 5 h at 37 ◦C. Samples of the initial donor solution (T0) were collected and stored at 5 °C. At the end of incubation, samples were collected from the donor and acceptor plates, and then added to plates containing Quench solution [(10% ultrapure water (type 1) and 90% methanol (HPLC grade ≥ 99.9%): acetonitrile (HPLC grade ≥ 99.9%) (1:1) + 50 nM internal standard)], T0 samples were treated similarly. The final concentrations of compounds in the donor, acceptor and T0 wells were quantified by LC-MS/MS. The results were used to calculate an effective permeability (Pe) value. The PAMPA assay was performed in triplicate.

Microsomal stability

For the experimental stability determination of test compounds in human and mouse liver microsomes in the presence of NADPH a clearance rate is determined. Assay conditions were 0.25 mg/mL liver microsomal protein from the species of interest (mouse and human), 0.5 μM test compound, at pH 7.4 and 37 °C. Samples were taken at 0, 5, 10, 20, 30 and 60 mins in triplicate. The reaction was started after the T0 is taken with the addition of NADPH at 0.5 μM. Reaction is stopped by addition acetonitrile (HPLC grade ≥ 99.9%): methanol (HPLC grade ≥ 99.9%) (1:1) + 50 nM internal standard. Time point samples are combined in compound groups of four that have been pre-sorted by mono molecular weight and analyzed by LC/MS/MS. The peak area ratios (analyte/internal standard) were converted into % remaining using the area ratio in Tzero as 100%, from these data obtaining exponential decay curves y=e^(-k) of % remaining vs. incubation time. The decay rate (k) was used to calculate the half-life (T1/2) in minutes (t½ = ln(2)/k) and intrinsic clearance (CLint) in μL/min/mg (Clint = k x 1000/(0.25). Data was qualified if t½>4X the last time point.

Kinetic solubility assay

To determine kinetic solubility, 10 mM samples of each compound were transferred to a 96-well plate (incubation plate) in duplicate; for each sample on the plate, 195 μL of PBS buffer

pH 7.4 and 2.0 (final concentration of 250 μM) was added, and DMSO concentration was 2.5%; the plate was sealed and shaken for 24 ±1 h (200 rpm, r.t.). The precipitates on the incubation plate were removed by centrifugation (15 min, 3000 rpm, r.t.). The supernatant fractions were quantified by LC- MS/MS. Calibration curves were prepared for each compound by diluting 10 mM samples to reach the desired concentration of 50, 40, 20, 2, and 1 μM. The resulting equation for the calibration curve (y =mx + b) was used to calculate the experimental concentration values.

Estimation of log D at pH 7.4

To determine the lipophilicity of the compounds, a methodology based on the retention time of molecules in reverse stationary phase (Supelco Ascentis express RP amide HPLC column 5 cm ×2.1 mm, 2.7 μM). was used. The chromatogram was obtained using liquid chromatography-tandem mass spectrometry (LC-MS/MS). Test compounds were prepared at 1.0 μg/mL by adding the stock solution at (1:1) mobile phases A:B + internal standard at 200 nM (A: 5% methanol in 10 mM ammonium acetate pH 7.4/B: 100% methanol), with a DMSO concentration lower than 2%. The lipophilicity of compounds was assessed by individually injecting the test compounds and a series of eight commercial drugs for which LogD values had already been determined, covering a LogD range of -1.86 to 6.1. The retention time (in minutes) of each of the eight standards was plotted against their LogD values. The resulting equation for the calibration curve (y =mx + b) was used to calculate the LogD values for the test compounds.

Secondary parasitology and exploratory assays

High Content Screening of *T. cruzi* strains DTU-panel and time-kill assay

The assay was performed as described previously, with minor modifications.[3] This assay estimated the compounds' activity against strains of *T. cruzi* DTUs I – VI: Sylvio X10/1 (DTU I), Y clone H10-S (DTU II), ARMA13 cl1 (DTU III), ERA cl2 (DTU IV), 92-80 cl2 (DTU V) and CL Brener (DTU VI). Tissue cultured trypomastigotes (TCTs) forms obtained from infected LLC-MK2 (sourced from the Rio de Janeiro Cell Bank - BCRJ, Brazil), maintained in DMEM media with 10% (v/v) Cosmic Calf Serum (CS – Hyclone, Cytiva),100 U/mL penicillin, and 100 μg/mL streptomycin, in a humidified incubator at 37 °C in 5% (v/v) CO_2.

The compound activity assays were performed in 384-well plates containing 500 U2OS cells/well (BCRJ, Brazil) cultured in DMEM media, supplemented with 10% (v/v) CS, 100 U/mL penicillin, and 100 μg/mL streptomycin, at 37 °C in 5% (v/v) CO_2 in a humidified incubator for 24 h. Host cells were infected with TCTs harvested from culture supernatant and plated according to an adjusted multiplicity of infection (MOI) for the U2OS seeded 24 h earlier, as follows: MOI 20 (Sylvio X10/1 and Y H10-S), MOI 40 (ARMA13 cl1, ERA cl2 and CL Brener) or MOI 60 (92-80 cl2). Plates were incubated at 37 °C and 5% (v/v) CO_2 for additional 24 h, and then compounds were added to plates. Prior to plating, compound solutions were serially diluted by a factor of 2 in neat DMSO, followed by 33.3-fold dilution in PBS and added to assay infected cell-plate (200-fold final dilution). Plates treated with compounds were incubated at 37 °C and 5% (v/v) CO_2 for another 96 h. After treatment, cells were fixed with 4% (w/v) paraformaldehyde in PBS for 15 min at room temperature, washed with PBS pH 7.4, and stained 5 μM Draq5 and 10 μg/mL DAPI (both from Thermo Scientific). The infection readout was performed using Operetta's automated High Content Screening (HCS) imaging system (Perkin Elmer). The number of cells, the number of infected cells, and the total number of intracellular parasites (amastigotes) were determined using the Harmony Software (Perkin Elmer), as described.[4] Data of test and reference compounds were normalized to intraplate controls (infected and non-infected controls treated with 0.5% DMSO, v/v), and the antiparasitic activity was determined based on the inhibition of infection. Concentration-response curves were obtained using GraphPad Prism Software v8.4.0, as described.[4] Time-kill assays were performed with a similar methodology,[3] with small modifications. Briefly, 500 U2OS cells/wells were seeded in 384-well plates, and after 24 h, infected with 10,000 *T. cruzi* trypomastigotes/well (Sylvio X10/1). At 48 h post-infection (hpi), compounds were added to plates as described above, and one replica of each plate was fixed at every 24 h, starting at 0 h and up to 96 h post compound plating. Plates were stained and analyzed as described above.

In vivo acute *T. cruzi* infection assay

Female 8-week old BALB/c mice (n = 6-7/group) were infected with 1×10^3 bloodstream *T. cruzi* trypomastigotes expressing the red-shifted firefly luciferase (strain CL Brener Luc::Neon lineage),[5] obtained from infected BALB/c mice immunosuppressed with cyclophosphamide (66 mg/kg i.p every 72 h for 13 days). The treatment regimen of **2** at 50 mg/kg *bis in die* (BID) orally for 5 days and co-treatment with ABT (Sigma-Aldrich) at 50 mg/kg 30 min before compound dosing. Treatment started at 14 days post-infection (dpi) at the parasitemia peak,

until 18 dpi. A control group was treated with benznidazole 100 mg/kg QD for 5 days. For imaging, mice were injected with 150 mg/kg of D-luciferin (IVIS brite D-Luciferin Potassium Salt, Revvity) i.p. and anesthetized using 2.5% (v/v) isoflurane in oxygen. Images were acquired in the IVIS Spectrum system (Revvity), using the Living Image 4.3. software. The bioluminescence levels of controls and test groups were determined by measuring the ventral and dorsal luminescence values in flux (photons/second).[5–7] Data analysis was performed in Living Image 4.7. software and GraphPad prism v8.4.0. The detection threshold was determined following the same procedure by imaging non-infected control mice of similar weight and age (n=3). The percentage of bioluminescence reduction was calculated by the **Equation S1.**

$$\% \, Reduction = \left\{ \left[\frac{(T - \overline{NT})}{(\bar{t} - \overline{NT})} \right] - 1 \right\} \times 100$$

Equation S1: *Normalized bioluminescence reduction. T stands for treated BLI values for each mouse, NT for non-treated group BLI values, and t for threshold group BLI values. The normalized BLI reduction is expressed in percentage considering the day of the acquisition.*

In vivo chronic *T. cruzi* infection assay

Female 8-week old BALB/c mice (n = 5-6/group) were infected with 1 × 10^3 bloodstream *T. cruzi* trypomastigotes expressing the red-shifted firefly luciferase (strain CL Brener Luc::Neon lineage),[5] obtained from infected BALB/c mice immunosuppressed with cyclophosphamide (66 mg/kg i.p every 72 h for 13 days). The chosen treatment regimen of **2** was 50 mg/kg BID orally for 5 days and co-treatment with ABT (Sigma-Aldrich) at 50 mg/kg, 30 min prior compound dosing. Treatment started at 108 days post-infection (dpi), when infections had reached the chronic stage, until 117 dpi. A control group was treated with benznidazole 100 mg/kg QD for 10 days. At the end of the treatment washout period, mice that did not present detectable bioluminescence were subjected to an immunosuppression regimen with cyclophosphamide (CTX) at 125 mg/kg (i.p.) to confirm the absence of infection and the cure rate. For imaging, mice were injected with 150 mg/kg of D-luciferin (IVIS brite D-Luciferin Potassium Salt, Revvity) i.p. and anesthetized using 2.5% (v/v) isoflurane in oxygen. Images were acquired in the IVIS Spectrum system (Revvity), using the Living Image 4.3. software. The bioluminescence levels of controls and test groups were determined by measuring the ventral and dorsal luminescence values in flux (photons/second).[5–7] Data analysis was performed in Living Image 4.7. software and GraphPad prism v8.4.0. The detection threshold was

determined following the same procedure by imaging non-infected control mice of similar weight and age (n=3). The percentage reduction in bioluminescence was calculated using Equation S1. For the 135 dpi, a representative image was included, and the calculations were based on the average vehicle values from the same experiment.

Organic synthesis procedures

General procedures

Reagents purchased were used as received, unless otherwise noted. Dichloromethane (DCM), and triethylamine (Et_3N) were distilled from CaH_2. Tetrahydrofuran (THF) was distilled from sodium/benzophenone. Dimethyl formamide (DMF), acetonitrile (MeCN), and 1,4-dioxane were purchased from Aldrich (anhydrous) and used without further purification. Room temperature indicates temperatures in the range of 20–25 °C. For the purposes of thin layer chromatography (TLC), Merck silica-aluminum plates were used, with UV light (254 nm), phosphomolybdic acid, iodine, vanillin, ninhydrin, and potassium permanganate used for visualization. Intermediates and final compounds were purified using silica gel or reverse phase chromatography using the Biotage Isolera, or Selekt flash purification systems. Where required, final compounds were purified by preparative reverse phase HPLC (Phenomenex luna C_{18} 100 × 40 mm, 3 μm column), with a single wavelength UV−visible detector. LCMS/HPLC analysis was performed using either: [a]Waters Alliance reverse phase HPLC (columns Waters SunFire C_{18} 4.6 × 50 mm, 3.5 μm, or Waters SunFire C_8 4.6 × 50 mm, 3.5 μm), using a multiwavelength photodiode array detector from 210 to 600 nm and either a Waters Micromass ZQ detector (electrospray ionization), or Waters Micromass QDA detector. [b]Waters Alliance reverse phase HPLC (2695; Xbridge C_{18} 4.6 × 50 mm, 3.5 μm), using a multiwavelength photodiode array detector from 210 nm to 600 nm and Waters Micromass QDA detector. [c]Shimadzu LC-20AT, detector SPD-M20A. All compounds tested had a purity of >95% as measured by LCMS, unless otherwise noted. ^{1}H NMR spectra were obtained with Bruker NMR systems, operating at either 250, 400, 500 or 600 MHz at room temperature. Chemical shifts (δ, ppm) are reported relative to the solvent peak ($CDCl_3$: 7.26 [1H]; DMSO-d_6: 2.50 [1H]; CD_3OD: 3.31 [1H]; or Acetone-d_6: 2.05 [1H]). Data for ^{1}H NMR spectra are reported as follows: chemical shift (ppm),

multiplicity (s for singlet, br.s for broad singled, d for doublet, t for triplet, dd for doublet of doublet, m for multiplet), coupling constant (Hz), and integration. Coupling constants (*J*) are given in Hz and are uncorrected. High-resolution mass spectrometry (HRMS) was measured using electrospray ionization (ESI) (Q-Exactive PlusThermo Fisher Scientific), positive mode from 50-750 *m/z* and cone tension of 3.5KV and 50V SLens.

General procedure A for the synthesis of substituted pyridyl nitriles.

A mixture of (hetero)-aromatic halo-nitriles (1.0 eq.) and K_2CO_3 (2.0 eq.) in MeCN (150 mM) is cooled to 0 °C. Then, amine (1.5 eq.) is added and the reaction stirred at rt on. The solvent was removed and the reaction mixture was diluted with EtOAc and washed with brine (30 mL), dried over anhydrous Na_2SO_4, filtered, and evaporated to dryness. The crude was purified by silica gel chromatography to give the title compound.

General Procedure B for the reduction of nitriles to Boc-protected primary amines.

A solution of (hetero)-aromatic-nitriles (1.0 eq.) in dry MeOH (150 mM) is cooled to 0 °C, then were added Boc_2O (2.0 eq.), $NiCl_2 \cdot 6H_2O$ (0.10 eq.) and $NaBH_4$ (7.0 eq.) portion wise.[8] The resulting reaction mixture containing a finely divided black precipitate was allowed to warm to rt and stirred for a further 1 h. Diethylenetriamine (1.0 eq.) was added and the mixture was allowed to stir for 1 h before solvent evaporation. The purple residue was dissolved in EtOAc and extracted with saturated $NaHCO_3$ solution. The organic layer was dried over Na_2SO_4 and the solvent removed in vacuum.

General procedure C for N-Boc deprotection.

To a cooled solution of Boc-protected derivative (1.0 eq.) in dry DCM (500 mM), a solution of HCl 4M in dioxane (3.0 eq.) is dropwise added and the reaction is left to stir on. After the reaction is complete, the solvents are removed in vacuo and the residual solid washed with cold diethyl ether to afford the corresponding hydrochloride salt or extracted with EtOAc and saturated sodium bicarbonate to give the corresponding free base.

General procedure D for Sulfonylation.

A solution of 4-aminobenzonitrile (1.0 eq.) is cooled to 0 °C, added pyridine (1.0 eq.) in dry DCM (500 mM). After stirring for 10 minutes, was added dropwise a solution of corresponding sulfonyl chloride in DCM left to stir on. After the reaction is complete, solvents are removed

in vacuum and crude is dissolved in EtOAc, washed with HCl 1% and brine. The product was purified by chromatography.

General procedure E for the synthesis of indole derivatives.

Compounds were prepared using the previously described methodology by Ren and coworkers.[9] In a Schlenk tube, was added aniline derivative (1.0 eq.), $Pd(OAc)_2$ (0.05 eq.), and pre-activated 4Å MS equipped with a magnetic stirrer bar. After the air was evacuated and the tube was refilled with O_2 for three times, ethyl, or methyl pyruvate (2.0 eq.), AcOH (4.0 eq.), and DMSO (100 mM) were added via syringe. The mixture was stirred at 70 °C until completion. Upon cooling to room temperature, the reaction mixture was diluted with ethyl acetate and filtered through a pad of Celite using ethyl acetate as an additional eluent. The filtrate was washed with NaCl aqueous solution, dried over Na_2SO_4, filtered, and evaporated under reduced pressure. The residue was purified through flash column chromatography on silica gel furnishing the desired indole derivative.

General procedure F for ester hydrolysis.

The ester (1.0 eq.) was dissolved in the appropriate alcohol (methanol or ethanol) (500mM), and with stirring, lithium hydroxide (LiOH) (5.0 eq,) was added. The reaction mixture was maintained under reflux, and upon completion, the solvent was evaporated, and a 1M hydrochloric acid solution was added until the pH was approximately 3. The carboxylic acid precipitated, was filtered, and dried under vacuum.

General procedure G for the synthesis of reverse sulfonamides.

A mixture of 4-cyanobenzenesulfonyl chloride (1.0 eq.) or 6-aminonicotinonitrile (1.0 eq.) and DIPEA (3.0 eq.) in DCM (150 mM) is cooled to 0 °C. Then, amine (1.5 eq.) is added and the reaction stirred at rt on. The reaction mixture was diluted with DCM and washed with brine (30 mL), dried over anhydrous Na_2SO_4, filtered, and evaporated to dryness. The crude was purified by silica gel chromatography to give the title compound.

General procedure H for the synthesis of substituted isoxazole carboxylic acids.

Aldehydes of interest (1.0 eq.) were added to a stirred solution of hydroxylamine hydrochloride (1.10 eq.) in THF:EtOH:H_2O (2:5:1 v/v, 0.3 M), with the mixture being stirred at room temperature for approximately 30 min. THF and EtOH were then evaporated under reduced pressure. The remaining residue was extracted with diethyl ether, brine, and dried over

anhydrous Na_2SO_4. Solvents were removed under reduced pressure, the crude was washed with cold hexane, the crystals were filtered and dried to yield the desired aldoximes. The aldoxime (1.0 eq.) was dissolved in THF (500 mM), then N-chlorosuccinimide (1.10 eq.) was added. The reaction mixture was stirred at room temperature up to completion consumption of the starting material and then triethylamine (1.20 mmol) and methyl propiolate (1.10 equiv) were added. The reaction mixture was stirred overnight at 60 °C and then concentrated under vacuum. The residue was taken up in DCM and diluted with 1M HCl. The separated organic phase was washed with water. The organic phase was dried over $MgSO_4$, filtered and the solvent was removed under vacuum. The crude was purified by flash chromatography to give methyl esters. Methyl esters were hydrolyzed according to general procedure F, to yield the final isoxazole-carboxylic acids[10,11].

General procedure I for amide coupling

Step 1: to a cooled solution of Boc-amine (1.0 eq.) in DCM (85 mM), HCl (4M in dioxane, 10 equiv.) is dropwise added and the reaction is left to stir on. After the reaction is complete, the solvents are removed in vacuo. Step 2: to a solution of appropriate carboxylic acid (1 equiv.) in DMF (200 mM), DIPEA (3.0 eq.), and HBTU (1.2 eq.) were added. After stirring for 20 min, the previously prepared amine (1 equiv.) has been added and the resulting mixture was stirred at rt for 5 h. After the reaction was complete, it was diluted with EtOAc (20 mL), washed with water (2 x 10 mL), and brine (5 mL), and dried under anhydrous Na_2SO_4. The solvent was removed under vacuum and the residue purified by chromatography to provide the desired product.

General procedure J for amide coupling

To a solution of appropriate carboxylic acid (1.0 eq.) in DCM (200 mM), 1-ethyl-3-(3-dimethylaminopropyl)carbodiimide (EDC) (1.2 – 1.5 eq.), hydroxybenzotriazole (HOBt) (1.2 eq.), and triethylamine (Et_3N) (3.0 eq.) were added. The mixture was stirred at room temperature for 1 hour, and then the corresponding free amine or hydrochloride (1.0 – 1.5 eq.) was added. After the reaction was complete, it was diluted with EtOAc, washed with water, brine, and dried under anhydrous Na_2SO_4. The solvent was removed under vacuum and the residue purified by chromatography to provide the desired product.

General procedure K for urea derivatives.

CDI (1.0 eq.) was dissolved in DCM (200 mM) and cooled to 0 °C, then appropriate amine (1.0 eq.) and DIPEA (1.0 eq.) were added, and the reaction mixture was warmed to rt and stirred for 24 h. After the reaction was complete, the solvent was removed under vacuum. The residue was dissolved in MeCN (200 mM), iodomethane (4.0 eq.) was added and the reaction was stirred for further 24 h. The mixture was concentrated in vacuo. The crude is re-dissolved in DMF (130 mM) and 4-aminomethylbenzonitrile HCl (1.2 eq.) and DIPEA (2.0 eq.) were added and the reaction stirred for 48 h. The solvent was removed under vacuum and the residue purified by reverse phase chromatography.

Intermediates characterization

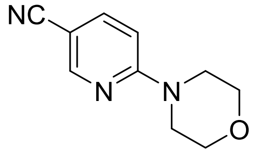

6-Morpholinonicotinonitrile (1.2)

Compound was synthesized using 6-aminonicotinonitrile (5.00 g, 36.1 mmol) and morpholine (6.31 mL, 72.2 mmol) according to General Procedure A. The crude was purified by silica gel chromatography (10% DCM in MeOH) to give the title compound as a white solid (6.43g mg, 94%). ^{1}H NMR (400 MHz, $CDCl_3$): δ 8.42 (br. s., 1H), 7.65 (dd, *J* = 2.1, 8.9 Hz, 1H), 6.60 (d, *J* = 8.9 Hz, 1H), 3.75–3.89 (m, 4H), 3.65 (t, *J* = 4.9 Hz, 4H). ^{1}H NMR (250 MHz, $CDCl_3$): δ 8.41 (d, *J* = 2.3 Hz, 1H), 7.63 (dd, *J* = 9.0, 2.3 Hz, 1H), 6.58 (d, *J* = 9.0 Hz, 1H), 3.84 – 3.74 (m, 4H), 3.68 – 3.60 (m, 4H).

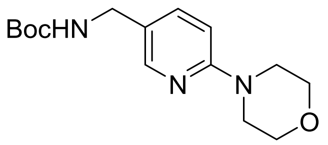

***tert*-Butyl (6-morpholinopyridin-3-yl)methyl)carbamate (1.3)**

Compound was synthesized using 6-morpholinonicotinonitrile (6.40 g, 33.8 mmol) according to General Procedure B and used as it without further purification (7.12 g, 72%). ^{1}H NMR (500 MHz, $CDCl_3$): δ 8.11 (s, 1H), 7.48 (d, *J* = 7.3 Hz, 1H), 6.62 (d, *J* = 8.3 Hz, 1H), 4.20 (d, *J* = 5.4 Hz, 2H), 3.79–3.87 (m, 4H), 3.45–3.53 (m, 4H), 1.44–1.49 (m, 9H).

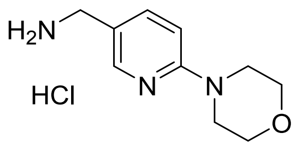

(6-Morpholinopyridin-3-yl)methanamine hydrochloride (1.4)

Compound was synthesized using *tert*-butyl (6-morpholinopyridin-3-yl)methyl)carbamate (6.12 g, 21.0 mmol) according to general procedure C to afford a pale yellow solid (4.68 g, 97%). ^{1}H NMR (500 MHz, CD_3OD): δ 8.19 (d, *J* = 1.9 Hz, 1H), 7.68 (dd, *J* = 1.9, 8.8 Hz, 1H), 6.89 (d, *J* = 8.8 Hz, 1H), 4.03 (s, 2H), 3.75–3.85 (m, 4H), 3.49–3.60 (m, 4H).

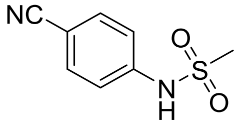

***N*-(4-Cyanophenyl)methanesulfonamide (2.2)**

Compound was synthesized using 4-aminobenzonitrile (2.18 g, 18.5 mmol) and mesyl chloride (1.43 mL, 18.5 mmol) according to General Procedure D to afford a pink solid (3.54 g, 98%). ^{1}H NMR (500 MHz, DMSO-d_6): δ 10.49 (s, 1H), 7.82 – 7.77 (m, 2H), 7.35 – 7.30 (m, 2H), 3.14 (s, 3H).

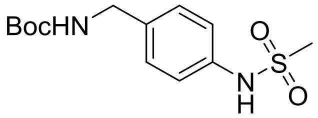

***tert*-Butyl (4-(methylsulfonamido)benzyl)carbamate (2.3)**

Compound was synthesized using *N*-(4-cyanophenyl)methanesulfonamide (2.13 g, 10.8 mmol) according to General Procedure B and used without further purification (2.82 g, 87%). ^{1}H NMR

(250 MHz, DMSO-d_6): δ 9.63 (s, 1H), 7.34 (t, *J* = 6.1 Hz, 1H), 7.23 – 7.09 (m, 4H), 4.07 (d, *J* = 6.2 Hz, 2H), 2.94 (s, 3H), 1.39 (s, 9H).

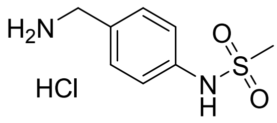

***N*-(4-(Aminomethyl)phenyl)methanesulfonamide hydrochloride (2.4)**

Compound was synthesized using *tert*-butyl (4-(methylsulfonamido)benzyl)carbamate (4.81 g, 16.0 mmol) according to general procedure C to afford a pale yellow solid (3.54 g, 93%). ^{1}H NMR (250 MHz, DMSO-d_6): δ 9.90 (s, 1H), 8.33 (s, 3H), 7.56 – 7.39 (m, 2H), 7.28 – 7.18 (m, 2H), 3.96 (d, *J* = 5.5 Hz, 2H), 3.00 (s, 3H).

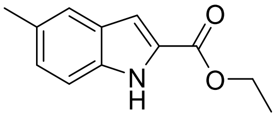

Ethyl 5-methyl-1*H*-indole-2-carboxylate (3.2a)

Compound was synthesized using *p*-toluidine (1.07 g, 10 mmol) and ethyl pyruvate (2.22 mL, 20 mmol) according to General Procedure E and purified by FCC (EtOAc/Hexanes 0-30%) to afford a white solid (1.51 g, 74%). ^{1}H NMR (500 MHz, DMSO-d_6): δ 11.74 (s, 1H), 7.41 (s, 1H), 7.34 (d, *J* = 8.4 Hz, 1H), 7.11 – 7.02 (m, 2H), 4.32 (q, *J* = 7.1 Hz, 2H), 2.36 (s, 3H), 1.33 (t, *J* = 7.1 Hz, 3H).

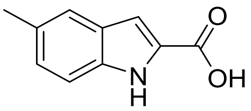

5-Methyl-1*H*-indole-2-carboxylic acid (3.3a)

Compound was synthesized using ethyl 5-methyl-1*H*-indole-2-carboxylate (245 mg, 1.21 mmol) according to general procedure F, furnishing an off-white solid (199 mg, 94%). ^{1}H NMR (250 MHz, DMSO-d_6): δ 11.60 (s, 1H), 7.43 – 7.38 (m, 1H), 7.32 (d, J = 8.4 Hz, 1H), 7.06 (dd, J = 8.5, 1.6 Hz, 1H), 6.98 (dd, J = 2.2, 0.9 Hz, 1H), 2.36 (s, 3H).

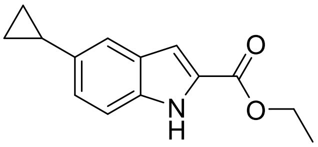

Ethyl 5-cyclopropyl-1*H*-indole-2-carboxylate (4.1)

A solution of ethyl 5-bromo-l*H*-indole-2-carboxylate (100 mg, 0.37 mmol), cyclopropylboronic acid (48 mg, 0.56 mmol), $Pd(OAc)_2$ (42 mg, 0.19 mmol), K_3PO_4 (158 mg, 0.75 mmol) and SPhos (77 mg, 0.19 mmol) in toluene (2.5 mL) and H_2O (0.5 mL) was evacuated and backfilled with N_2. The mixture was stirred at 80 °C. After the reaction was complete, toluene was concentrated under vacuum and the residue diluted with EtOAc (15 mL) and washed with water (10 mL × 2). The combined organic layers were concentrated to dryness to give a dark yellow solid which was purified by silica gel chromatography (15% EtOAc:Hex) to give the title compound as a pale-yellow solid (60 mg, 70%). ^{1}H NMR (500 MHz, $CDCl_3$): δ 8.81 (br. s., 1H), 7.41 (s, 1H), 7.32 (d, J = 8.3 Hz, 1H), 7.15 (d, J = 1.0 Hz, 1H), 7.10 (dd, J = 1.5, 8.8 Hz, 1H), 4.41 (q, J = 7.2 Hz, 2H), 1.97–2.04 (m, 1H), 1.42 (t, J = 7.2 Hz, 3H), 0.93–0.99 (m, 2H), 0.68–0.75 (m, 2H).

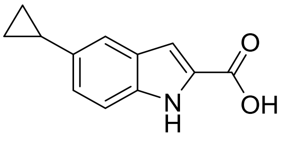

5-Cyclopropyl-1*H*-indole-2-carboxylic acid (3.3b)

Compound was synthesized using ethyl 5-cyclopropyl-1*H*-indole-2-carboxylate (180 mg, 0.79 mmol) according to general procedure F furnishing a white solid (151 mg, 95%). ^{1}H NMR (500 MHz, $CDCl_3$): δ 8.90 (br. s., 1H), 7.43 (s, 1H), 7.35 (d, *J* = 8.8 Hz, 1H), 7.26–7.31 (m, 2H), 7.14 (d, *J* = 8.3 Hz, 1H), 1.99–2.05 (m, 1H), 0.94–1.02 (m, 2H), 0.70–0.76 (m, 2H).

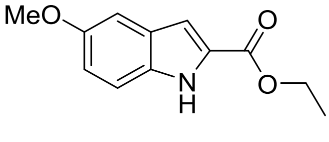

Ethyl 5-methoxy-1H-indole-2-carboxylate (3.2c)

Compound was synthesized using 4-methoxyaniline (610 mg, 5.0 mmol) and ethyl pyruvate (0.90 mL, 8 mmol) according to General Procedure E and purified by FCC (EtOAc/Hexanes 0-10%) to furnish a white solid (400 mg, 36%). ^{1}H NMR (250 MHz, $CDCl_3$) δ 9.07 (br. s., 1H), 7.33 (d, *J* = 8.9 Hz, 1H), 7.16 (dd, *J* = 0.8, 2.1 Hz, 1H), 7.09 (d, *J* = 2.4 Hz, 1H), 7.01 (dd, *J* = 2.4, 8.9 Hz, 1H), 4.43 (q, *J* = 7.1 Hz, 2H), 3.86 (s, 3H), 1.43 (t, *J* = 7.1 Hz, 3H).

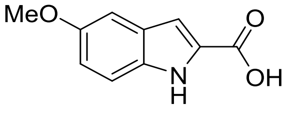

5-Methoxy-1*H*-indole-2-carboxylic acid (3.3b)

Compound was synthesized using ethyl 5-methoxy-1*H*-indole-2-carboxylate (300 mg, 1.4 mmol) according to general procedure F furnishing a white solid (250 mg, 93%). ^{1}H NMR (500

MHz, CD_3OD): δ 7.32 (d, *J* = 9.0 Hz, 1H), 7.07 (dd, *J* = 1.5, 8.7 Hz, 2H), 6.91 (dd, *J* = 2.4, 9.0 Hz, 1H), 3.79 (s, 3H).

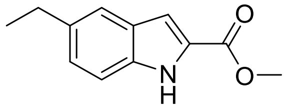

Methyl 5-ethyl-1*H*-indole-2-carboxylate (3.2d)

Compound was synthesized using 4-ethylaniline (1.5 g, 12.4 mmol) and methyl pyruvate (2.32 g, 20.0 mmol) according to General Procedure E and purified by FCC (EtOAc/Hexanes 0-30%) (1.6 g, 63%). ^{1}H NMR (400 MHz, $CDCl_3$): δ 8.98 (br. s., 1H), 7.50 (s, 1H), 7.36 (d, *J* = 8.4 Hz, 1H), 7.16 - 7.24 (m, 2H), 3.96 (s, 3H), 2.76 (q, *J* = 7.6 Hz, 2H), 1.30 (t, *J* = 7.6 Hz, 3H).

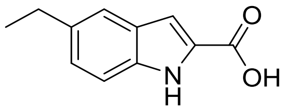

5-Ethyl-1*H*-indole-2-carboxylic acid (3.3d)

Compound was synthesized using methyl 5-ethyl-1*H*-indole-2-carboxylate (710 mg, 3.49 mmol) according to general procedure F furnishing an off-white solid (650 mg, 98%).

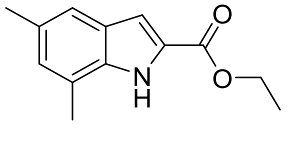

Ethyl 5,7-dimethyl-1*H*-indole-2-carboxylate (3.2e)

Compound was synthesized using 2,4-dimethylaniline (0.5 mL, 4.03 mmol) and ethyl pyruvate (0.90 mL, 8.09 mmol) according to General Procedure E and purified by FCC (EtOAc/Hexanes 0-30%) to afford a white solid (710 mg, 82%). ^{1}H NMR (250 MHz, DMSO-d_6): δ 11.56 (s, 1H),

7.23 (s, 1H), 7.05 (d, J = 2.1 Hz, 1H), 6.88 (s, 1H), 4.33 (q, J = 7.1 Hz, 2H), 2.48 (s, 3H), 2.32 (s, 3H), 1.34 (t, J = 7.1 Hz, 3H).

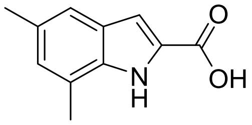

5,7-*di*Methyl-1*H*-indole-2-carboxylic acid (3.3e)

Compound was synthesized using ethyl 5,7-dimethyl-1*H*-indole-2-carboxylate (220 mg, 1.01 mmol) according to general procedure F furnishing a white solid (172 mg, 90%). ^{1}H NMR (250 MHz, $CDCl_3$): δ 8.75 (s, 1H), 7.33 (s, 1H), 7.30 – 7.26 (m, 1H), 7.00 (s, 1H), 2.50 (s, 3H), 2.42 (s, 3H).

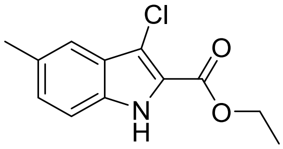

Ethyl 3-chloro-5-methyl-1*H*-indole-2-carboxylate (3.2f)

A solution of ethyl 5-methyl-1*H*-indole-2-carboxylate (255 mg, 1.25 mmol) and *N*-chlorosuccinimide (167 mg, 1.25 mmol) in dry acetone at room temperature. After the reaction was complete, acetone was concentrated under vacuum and the residue diluted with EtOAc (15 mL) and washed with water (10 mL × 2). The combined organic layers were concentrated to dryness to give the title compound as a pale-yellow solid (276 mg, 93%). ^{1}H NMR (500 MHz, DMSO-d_6): δ 12.00 (s, 1H), 7.43 – 7.32 (m, 2H), 7.19 (dd, J = 8.6, 1.6 Hz, 1H), 4.37 (q, J = 7.1 Hz, 2H), 2.41 (s, 3H), 1.36 (t, J = 7.1 Hz, 3H).

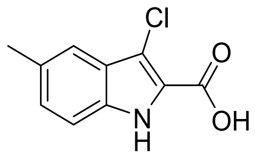

3-Chloro-5-methyl-1*H*-indole-2-carboxylic acid (3.3f)

Compound was synthesized using ethyl 3-chloro-5-methyl-1*H*-indole-2-carboxylate (277 mg, 1.16 mmol) according to general procedure F furnishing a white solid (214 mg, 88%).

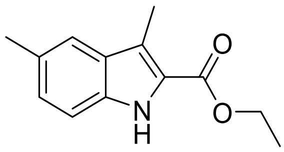

Ethyl 3,5-dimethyl-1*H*-indole-2-carboxylate (3.2g)

Compound was synthesized using the procedure previously described[12]. To a solution of *p*-toluidine (1.0 g, 9.33 mmol) in 2.7 mL of concentrated HCl and 4.5 mL of water was added dropwise a solution of $NaNO_2$ (695 mg, 10.1 mmol) in 1 mL of water at -5 °C. After complete addition, the mixture was stirred at 0 °C for 15 min and brought to pH 3-4 by addition of sodium acetate (893 mg, 6.56 mmol). In a separate flask, a solution of ethyl 2-ethyl-3-oxobutanoate (1.61 g, 10.2 mmol) in 7.5 mL of EtOH was cooled to 0 °C and combined with KOH (623 mg, 11.1 mmol) in 2 mL of water. To this solution was added 13 g of ice followed by addition of the diazonium salt prepared above. The mixture was then adjusted to pH 5-6 and stirred at 0 °C for 15 h. The completed reaction was extracted five times with 50 mL portions of DCM and the combined extracts were washed with brine and dried over Na_2SO_4. Most of the solvent was removed under reduced pressure, and the liquid residue was added dropwise to a solution of 14.5% ethanolic HCl at reflux. After refluxing this mixture for 2 h, the solvent was removed

under reduced pressure and the residue was combined with a mixture of 50 mL of water and 100 mL of DCM. The DCM layer was removed and the aqueous layer was extracted three times with 50-mL portions of DCM. The combined extracts were dried over Na_2SO_4 and concentrated to a residue, which was applied to a silica gel column prepared with DCM. Product fractions were evaporated to afford a white solid (642 mg, 32%). ^{1}H NMR (500 MHz, $CDCl_3$): δ 8.73 (s, 1H), 7.35 – 7.31 (m, 1H), 7.15 (d, *J* = 8.4 Hz, 1H), 7.04 (dd, *J* = 8.3, 1.7 Hz, 1H), 4.32 (q, *J* = 7.1 Hz, 2H), 2.49 (s, 3H), 2.36 (s, 3H), 1.32 (t, *J* = 7.2 Hz, 3H).

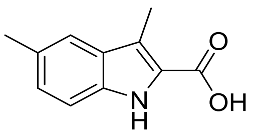

3,5-Dimethyl-1*H*-indole-2-carboxylic acid (3.3g)

Compound was synthesized using ethyl 3,5-dimethyl-1*H*-indole-2-carboxylate (250 mg, 1.15 mmol) according to general procedure F furnishing a white solid (200 mg, 92%). ^{1}H NMR (250 MHz, DMSO-d_6): δ 12.76 (s, 1H), 11.21 (s, 1H), 7.42 – 7.35 (m, 1H), 7.27 (d, *J* = 8.4 Hz, 1H), 7.06 (dd, *J* = 8.5, 1.6 Hz, 1H), 2.49 (s, 3H), 2.38 (s, 3H).

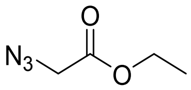

Ethyl 2-azidoacetate (5.2)

Compound synthesized as previously described[13]. To a mixture of 20 mL of acetone and water (1:1) was added sodium azide (8.1 g, 124.3 mmol) and ethyl 2-bromoacetate (7 mL, 65.4 mmol) and stirred overnight at room temperature. The heterogeneous mixture was diluted with water

(70 mL) and washed with ethyl ether (3 x 30 mL). The organic fractions were combined, dried with $MgSO_4$ and the solvent was removed under reduced pressure yielding a colorless liquid (7.21g, 87%). ^{1}H NMR (500 MHz, $CDCl_3$): δ 4.25 (q, J = 7.1 Hz, 2H), 3.85 (s, 2H), 1.30 (t, J = 7.2 Hz, 3H).

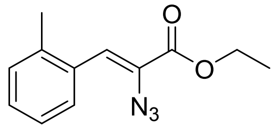

Ethyl (*Z*)-2-azido-3-(*o*-tolyl)acrylate (5.3)

Compound synthesized as previously described[14]. A solution of sodium ethoxide was prepared from metallic sodium (303 mg, 13.2 mmol) in 35 mL of anhydrous ethanol and cooled to -10 °C under an argon atmosphere. To the reaction mixture, a solution containing the corresponding aldehyde (452.9 mg, 3.77 mmol) and ethyl azidoacetate (1.70 g, 13.2 mmol) in 5 mL of ethanol was added dropwise with constant stirring, while maintaining the temperature below -10 °C. The reaction mixture was stirred for 2.5 hours and after completion, 0.5 mL of a saturated solution of NH_4Cl was added, and the solvent was removed under reduced pressure. Extraction was performed with ethyl acetate and water 3 x 50 mL, the organic phases were combined and dried over $MgSO_4$, and the solvent was removed under vacuum. The product was purified by flash column chromatography using silica gel and eluted with acetate:hexanes 0-2%, yielding a yellowish-green oil (533 mg, 61%). ^{1}H NMR (500 MHz, DMSO-d_6): δ 7.91 (d, J = 5.9 Hz, 1H), 7.27 – 7.22 (m, 3H), 7.07 (s, 1H), 4.33 (q, J = 7.2 Hz, 2H), 2.31 (s, 3H), 1.32 (t, J = 7.2 Hz, 3H).

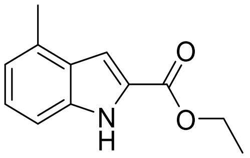

Ethyl 4-methyl-1*H*-indole-2-carboxylate (3.2h)

Compound synthesized as previously described[14]. A solution of ethyl (*Z*)-2-azido-3-(*o*-tolyl)acrylate (100 mg, 0.43 mmol) was dissolved in toluene (10 mL) and refluxed overnight. After the reaction was complete, toluene was concentrated under vacuum and the residue diluted with EtOAc (15 mL) and washed with water (2x10 mL). The combined organic layers were concentrated to dryness to give yellow solid which was purified by silica gel chromatography (15% EtOAc:Hex) to give the title compound as a yellow solid (76 mg, 86%). ^{1}H NMR (250 MHz, $CDCl_3$): δ 8.90 (s, 1H), 7.31 – 7.17 (m, 3H), 6.99 – 6.90 (m, 1H), 4.42 (q, J = 7.1 Hz, 2H), 2.57 (s, 3H), 1.42 (t, J = 7.1 Hz, 3H).

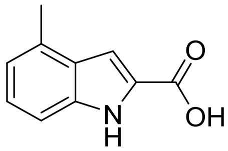

4-Methyl-1*H*-indole-2-carboxylic acid (3.3h)

Compound was synthesized using ethyl 4-methyl-*1*H-indole-2-carboxylate (68 mg, 0.51 mmol) according to general procedure F furnishing a white solid (70 mg, 78%). ^{1}H NMR (500 MHz, CD_3OD): δ 7.16 (d, J = 8.3 Hz, 1H), 7.09 (s, 1H), 7.03 (dd, J = 8.3, 7.1 Hz, 1H), 6.75 (d, J = 7.0 Hz, 1H), 2.42 (s, 3H).

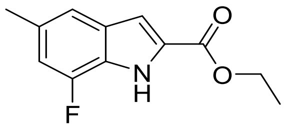

Ethyl 7-fluoro-5-methyl-1*H*-indole-2-carboxylate (3.2i)

Compound was synthesized using 2-fluoro-4-methylaniline (1.01 g, 8.10 mmol) and ethyl pyruvate (1.81 mL, 16.2 mmol) according to General Procedure E and purified by FCC (EtOAc/Hexanes 0-30%) to afford a white solid (1.23 g, 69%). ^{1}H NMR (250 MHz, $CDCl_3$): δ 8.81 (s, 1H), 7.21 – 7.11 (m, 2H), 6.97 – 6.85 (m, 1H), 4.42 (q, *J* = 7.2 Hz, 2H), 2.51 (s, 3H), 1.42 (t, *J* = 7.1 Hz, 3H).

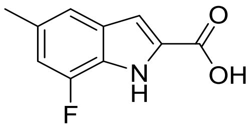

7-fluoro-5-methyl-1*H*-indole-2-carboxylic acid (3.3i)

Compound was synthesized using ethyl 7-fluoro-5-methyl-1*H*-indole-2-carboxylate (340 mg, 1.54 mmol) according to general procedure F furnishing a white solid (206 mg, 70%). ^{1}H NMR (250 MHz, DMSO-d_6): δ 12.96 (s, 1H), 11.73 (s, 1H), 7.21 (dd, *J* = 9.6, 2.5 Hz, 1H), 7.07 (d, *J* = 2.1 Hz, 1H), 6.93 (ddd, *J* = 10.2, 2.5, 1.0 Hz, 1H), (CH_3 overlapped with DMSO).

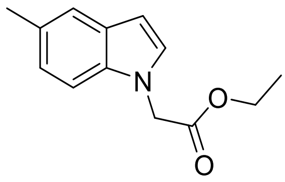

Ethyl 2-(5-methyl-1*H*-indol-1-yl)acetate

To an ice-cold solution of 5-methylindole (2.00 g, 15.2 mmol) and ethyl bromoacetate (2.60 mL, 23.5 mmol) in dry acetone 51 mL (300 mM), solid K_2CO_3 (4.21 g, 30.5 mmol) was added portion wise within ca. 5 minutes. The turbid solution was stirred for 15 minutes, then the ice-bath was removed and stirring continued at room temperature at 50 °C for 2 days. After completion, the reaction was quenched with saturated NH_4Cl solution and acetone evaporated in vacuum. The product was extracted with ethyl acetate. The organic phase was washed with brine, dried over magnesium sulfate, filtrated, and concentrated to dryness in vacuum to furnish the desired ester which was used for the next step.

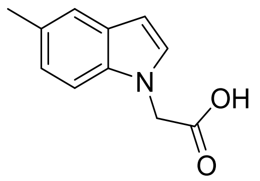

2-(5-Methyl-1*H*-indol-1-yl)acetic acid

Compound was synthesized ethyl 2-(5-methyl-1*H*-indol-1-yl)acetate (153 mg, 0.70 mmol) according to General Procedure F and used as it without further purification (100 mg, 65%). ^{1}H NMR (250 MHz, CD_3OD): δ 7.35 – 7.31 (m, 1H), 7.16 (d, *J* = 8.4 Hz, 1H), 7.11 (d, *J* = 3.2 Hz, 1H), 6.98 (dd, *J* = 8.4, 1.6 Hz, 1H), 6.37 (dd, *J* = 3.2, 0.9 Hz, 1H), (CH_2 overlapped with water peak), 2.40 (s, 3H).

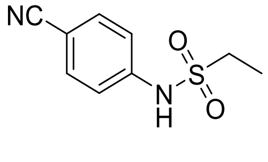

***N*-(4-Cyanophenyl)ethanesulfonamide (6.2a)**

Compound was synthesized using 4-aminobenzonitrile (1.0 g, 8.46 mmol) and ethanesulfonyl chloride (1.33 g, 10.2 mmol) according to general procedure D. The solid was washed with diethyl ether twice to afford a white solid (1.33 g, 75%). ^{1}H NMR (250 MHz, CD_3OD): δ 7.73 – 7.60 (m, 2H), 7.42 – 7.30 (m, 2H), 3.20 (q, J = 7.3 Hz, 2H), 1.31 (t, J = 7.4 Hz, 3H).

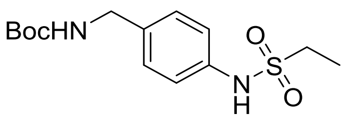

***tert*-butyl (4-(ethylsulfonamido)benzyl)carbamate (6.3a)**

Compound was synthesized using *N*-(4-cyanophenyl)ethanesulfonamide (1.31 g, 6.23 mmol) according to general procedure B. The crude was washed with diethyl ether twice to afford a white solid (1.47 g, 75%). ^{1}H NMR (250 MHz, CD_3OD): δ 7.28 – 7.14 (m, 4H), 4.18 (s, 2H), 3.06 (q, J = 7.4 Hz, 2H), 1.45 (s, 9H), 1.28 (t, J = 7.4 Hz, 3H).

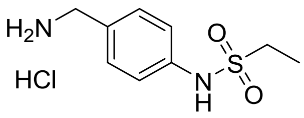

***N*-(4-(Aminomethyl)phenyl)ethanesulfonamide hydrochloride (6.4a)**

Compound was synthesized using *tert*-butyl (4-(ethylsulfonamido)benzyl)carbamate (1.47 g, 4.68 mmol) according to general procedure C. The crude was washed with diethyl ether twice to afford a white solid (1.10 g, 94%). ^{1}H NMR (250 MHz, D_2O): δ 7.53 – 7.43 (m, 2H), 7.41 – 7.29 (m, 2H), 4.18 (s, 2H), 3.29 (q, J = 7.4 Hz, 2H), 1.32 (t, J = 7.4 Hz, 3H).

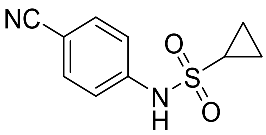

N-(4-Cyanophenyl)cyclopropanesulfonamide (6.2b)

Compound was synthesized using 4-aminobenzonitrile (500 mg, 4.23 mmol) and cyclopropanesulfonyl chloride (736 mg, 5.08 mmol) according to general procedure D, furnishing a white solid (922 mg, 98%). ^{1}H NMR (250 MHz, CD_3OD): δ 7.72 – 7.63 (m, 2H), 7.44 – 7.35 (m, 2H), 2.68 (tt, J = 7.9, 4.9 Hz, 1H), 1.12 (ddd, J = 8.3, 5.4, 3.1 Hz, 2H), 1.07 – 0.93 (m, 2H).

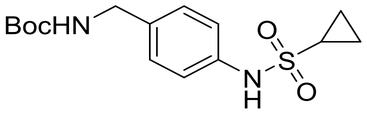

***tert*-Butyl (4-(cyclopropanesulfonamido)benzyl)carbamate (6.3b)**

Compound was synthesized using N-(4-cyanophenyl)cyclopropanesulfonamide (910 mg, 4.09 mmol) according to general procedure B, furnishing a white solid (820 mg, 61%). ^{1}H NMR (250 MHz, CD_3OD): δ 7.25 (br. s, 4H), 4.17 (d, J = 17.9 Hz, 2H), 2.60 – 2.45 (m, 1H), 1.48 (s, 9H), 1.07 – 0.98 (m, 2H), 0.98 – 0.88 (m, 2H).

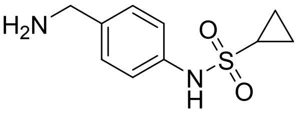

N-(4-(Aminomethyl)phenyl)cyclopropanesulfonamide (6.4b)

Compound was synthesized using *tert*-butyl (4-(cyclopropanesulfonamido)benzyl)carbamate (806 mg, 2.47 mmol) according to general procedure C, furnishing a pale yellow solid (636 mg,

98%). ^{1}H NMR (250 MHz, CD_3OD): δ 7.47 – 7.29 (m, 4H), 4.08 (s, 2H), 2.56 (tt, *J* = 7.9, 4.8 Hz, 1H), 1.09 – 0.88 (m, 4H).

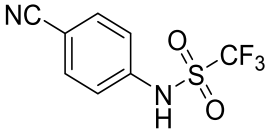

***N*-(4-Cyanophenyl)-1,1,1-trifluoromethanesulfonamide (6.2c)**

4-Aminobenzonitrile (450 mg, 3.80 mmol) was dissolved in dry THF (20 mL), cooled to 0 ˚C and added potassium *tert*-butoxide (850 mg, 7.6 mmol). After stirring for 5 minutes, phenyl triflimide (1.35 g, 3.80 mmol) was added portion wise c.a 10 minutes. After completion, the reaction was acidified with AcOH, and THF removed under vacuum. The crude was dissolved in EtOAc, washed with water and brine, and purified by FCC (hexanes:EtOAc 4:1) to afford the desired solid (350 mg, 37%). ^{1}H NMR (400 MHz, CD_3OD): δ 7.74 (d, *J* = 8.7 Hz, 2H), 7.44 (d, *J* = 8.8 Hz, 2H).

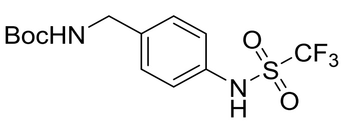

***tert*-Butyl (4-((trifluoromethyl)sulfonamido)benzyl)carbamate (6.3c)**

Compound was synthesized using *N*-(4-cyanophenyl)-1,1,1-trifluoromethanesulfonamide (990 mg, 3.96 mmol) according to general procedure B. The product was purified by FCC to afford the desired solid (1.30g, 92%). 1H NMR (250 MHz, $CDCl_3$): δ 7.19 (br. s, 4H), 5.02 (t, *J* = 6.1 Hz, 1H), 4.28 (d, *J* = 6.1 Hz, 2H), 1.47 (s, 9H).

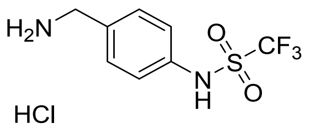

***N*-(4-(Aminomethyl)phenyl)-1,1,1-trifluoromethanesulfonamide hydrochloride (6.4c)**

Compound was synthesized using *tert*-butyl (4-((trifluoromethyl)sulfonamido)benzyl)carbamate (1.3 g, 3.7 mmol) according to general procedure C. The product was precipitated from hexanes and diethyl ether to afford the desired compound (1.05 g, 97%). ^{1}H NMR (400 MHz, DMSO-d_6): δ 8.62 (br. s., 3H), 7.52 - 7.60 (m, 2H), 7.27 - 7.35 (m, 2H), 3.98 (q, J = 5. 7 Hz, 2H).

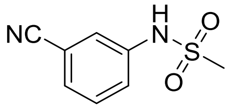

***N*-(3-Cyanophenyl)methanesulfonamide (6.2d)**

Compound was synthesized using 3-aminobenzonitrile (2.36 g, 20 mmol) and mesyl chloride (2.29 g, 20 mmol) according to general procedure D. The residue obtained was suspended in diethyl ether and filtered to afford a white solid (3.35 g, 85%). ^{1}H NMR (500 MHz, DMSO-d_6): δ 10.21 (s, 1H), 7.46 - 7.60 (m, 4H), 3.09 (s, 3H).

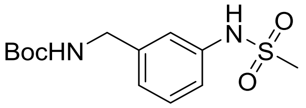

***tert*-Butyl (3-(methylsulfonamido)benzyl)carbamate (6.3d)**

Compound was synthesized using *N*-(3-cyanophenyl)methanesulfonamide (3.20 g, 16.31 mmol) according to general procedure B. The residue was precipitated from diethyl ether and

filtered to afford a white solid (3.25 g, 66%). ^{1}H NMR (400 MHz, DMSO-d_6): δ 9.72 (s, 1H), 7.38 (t, *J* = 6.0 Hz, 1H), 7.22 - 7.30 (m, 1H), 7.02 - 7.15 (m, 2H), 6.96 (d, *J* = 7.6 Hz, 1H), 4.09 (d, *J* = 6.1 Hz, 2H), 2.96 (s, 3H), 1.39 (s, 9H).

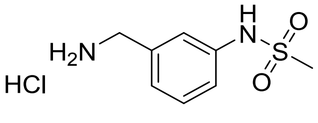

***N*-(3-(Aminomethyl)phenyl)methanesulfonamide hydrochloride (6.4d)**

Compound was synthesized using *tert*-butyl (3-(methylsulfonamido)benzyl)carbamate (3.10 g, 10.33 mmol) according to general procedure C. The product was washed with pentane and diethyl ether to afford the desired product (2.40 g, 98%). ^{1}H NMR (400 MHz, DMSO-d_6): δ 9.95 (s, 1H), 8.57 (br. s., 3H), 7.29 - 7.40 (m, 2H), 7.25 (d, *J* = 7.7 Hz, 1H), 7.14 - 7.21 (m, 1H), 3.96 (q, *J* = 5. 7 Hz, 2H), 3.04 (s, 3H).

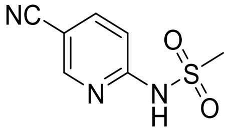

***N*-(5-Cyanopyridin-2-yl)methanesulfonamide (6.2e)**

Compound has been synthesized using 6-aminonicotinonitrile (200 mg, 1.68 mmol) and methane sulfonyl chloride (130 µL, 1.68 mmol) according to General Procedure D. The crude was purified by silica gel chromatography (40% EtOAc:Hex) to give the title compound as a white solid (265 mg, 80%). ^{1}H NMR (400 MHz, DMSO-d_6): δ 8.74–8.77 (m, 1H), 8.15 (d, *J* = 8.8 Hz, 1H), 7.02 (d, *J* = 8.8 Hz, 1H), 3.33 (br. s., 3H).

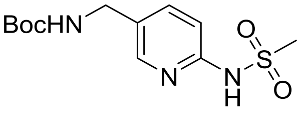

***tert*-Butyl ((6-(methylsulfonamido)pyridin-3-yl)methyl)carbamate (6.3e)**

Compound was synthesized using *N*-(5-cyanopyridin-2-yl)methanesulfonamide (100 mg, 0.51 mmol) according to General Procedure B and obtained as a white solid (100 mg, 65%). ^{1}H NMR (400 MHz, $CDCl_3$): δ 8.15 (br. s., 1H), 7.73 (d, *J* = 7.6 Hz, 1H), 7.39 (d, *J* = 8.1 Hz, 1H), 7.27 (br. s., 1H), 4.09–4.23 (m, 2H), 3.07 (br. s., 3H), 1.42 (d, *J* = 3.4 Hz, 9H).

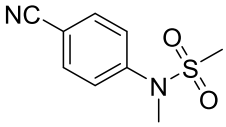

***N*-(4-Cyanophenyl)-N-methylmethanesulfonamide (6.2f)**

To a solution of *N*-(4-cyanophenyl)methanesulfonamide (600 mg, 3.06 mmol) in DMF (5.0 mL, 612 mM), was added potassium carbonate (634 mg, 4.59 mmol), iodomethane (868 mg, 6.12 mmol) and reaction was stirred at 80 °C for 7 h. After completion, was quenched with HCl (1.0 M) extracted with EtOAc, washed with water, brine, organic layers combined and dried using $MgSO_4$. Product was washed with diethyl ether twice to furnish the desired methylated analog (622 mg, 97%). ^{1}H NMR (250 MHz, $CDCl_3$): δ 7.73 – 7.62 (m, 2H), 7.55 – 7.44 (m, 2H), 3.36 (s, 3H), 2.87 (s, 3H).

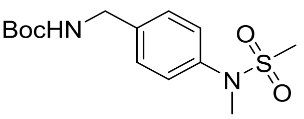

***tert*-Butyl (4-(*N*-methylmethylsulfonamido)benzyl)carbamate (6.3f)**

Compound was synthesized using *N*-(4-cyanophenyl)-N-methylmethanesulfonamide (587 mg, 2.79 mmol) according to general procedure B. The crude was washed with diethyl ether twice to furnish an off white solid (757 mg, 86%). ^{1}H NMR (250 MHz, DMSO-d_6): δ 7.48 – 7.31 (m, 3H), 7.25 (d, *J* = 8.3 Hz, 2H), 4.12 (d, *J* = 6.3 Hz, 2H), 3.21 (s, 3H), 2.92 (s, 3H), 1.39 (s, 9H).

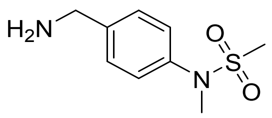

N-(4-(Aminomethyl)phenyl)-N-methylmethanesulfonamide (6.4f)

Compound was synthesized using *tert*-butyl (4-(*N*-methylmethylsulfonamido)benzyl)carbamate (757 mg, 2.41 mmol) according to general procedure C. The residual solid was washed with diethyl ether to furnish a pale yellow solid (514 mg, 85%). ^{1}H NMR (250 MHz, CD_3OD): δ 7.53 (s, 4H), 4.15 (s, 2H), (CH_3 overlapped with water peak), 2.91 (s, 3H).

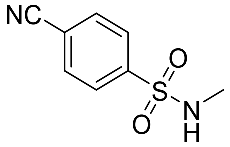

4-Cyano-*N*-methylbenzenesulfonamide (8.2a)

Compound has been synthesized using methylamine (744 µL, 1.49 mmol) according to General Procedure G. The crude was purified by silica gel chromatography (20% EtOAc:Hex) to give the title compound as a white solid (122 mg, 63%). ^{1}H NMR (500 MHz, Acetone-d_6): δ 8.04 (s, 4H), 6.64 (br. s., 1H), 2.63 (d, *J* = 5.4 Hz, 3H).

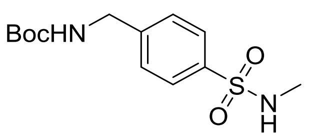

***tert*-Butyl (4-(*N*-methylsulfamoyl)benzyl)carbamate (8.3a)**

Compound was synthesized using 4-cyano-*N*-methylbenzenesulfonamide (120 mg, 0.61 mmol) according to General Procedure B and obtained as a white solid (140 mg, 76%). ^{1}H NMR (500 MHz, Acetone-d_6): δ 7.79 (d, J = 7.8 Hz, 2H), 7.52 (d, J = 7.8 Hz, 2H), 6.60–6.67 (m, 1H), 6.25–6.31 (m, 1H), 4.37 (d, J = 5.9 Hz, 2H), 2.56 (d, J = 5.4 Hz, 3H), 1.42–1.44 (m, 9H).

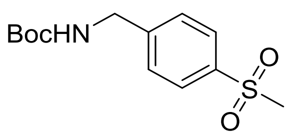

***tert*-Butyl (4-(methylsulfonyl)benzyl)carbamate (8.3b)**

Compound was synthesized using the commercially available 4-(methylsulfonyl)benzonitrile (400 mg, 2.21 mmol) according to General Procedure B and obtained as a white solid (500 mg, 79%). ^{1}H NMR (500 MHz, $CDCl_3$): δ 7.90 (d, J = 7.8 Hz, 2H), 7.48 (d, J = 7.8 Hz, 2H), 5.06 (br. s., 1H), 4.41 (d, J = 4.9 Hz, 2H), 3.04 (s, 3H), 1.47 (br. s., 9H).

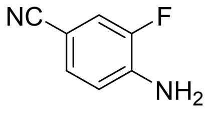

4-Amino-3-fluorobenzonitrile (9.2)

To a round bottom flask, was added 3-fluoro-4-nitrobenzonitrile (1.23g, 7.40 mmol) in MeOH:THF (1/1 v/v 50 mL) and stirred at room temperature. The flask was sealed and backfilled with nitrogen 3 times, and then added Pd/C 10% (788 mg, 7.40 mmol) and the

nitrogen was replaced with hydrogen 3 times. The reaction was stirred at room temperature for 2 days, and then was diluted with EtOAc, filtered through a pad of celite, washed with water, brine and dried using $MgSO_4$. The product was washed with diethyl ether twice to furnish the desired aniline derivative (765 mg, 76%). ^{1}H NMR (250 MHz, $CDCl_3$): δ 7.34 – 7.22 (m, 2H), 6.88 – 6.73 (m, 1H), 4.30 (s, 2H).

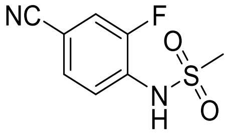

***N*-(4-Cyano-2-fluorophenyl)methanesulfonamide (9.3)**

Compound was synthesized using 4-amino-3-fluorobenzonitrile (300 mg, 2.20 mmol) and mesyl chloride (401 mg, 3.50 mmol) according to general procedure D. Product was washed with diethyl ether twice to furnish a brown solid (367 mg, 78%). ^{1}H NMR (250 MHz, DMSO-d_6): δ 10.3 (s, 1H), 7.92 (dd, J = 10.8, 1.8 Hz, 1H), 7.75 – 7.54 (m, 2H), 3.17 (s, 3H).

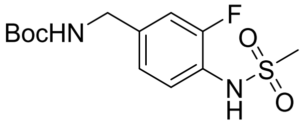

***tert*-Butyl (3-fluoro-4-(methylsulfonamido)benzyl)carbamate (9.4)**

Compound was synthesized using *N*-(4-cyano-2-fluorophenyl)methanesulfonamide (272 mg, 1.27 mmol) according to general procedure B. The crude was washed with diethyl ether twice to afford a white solid (200 mg, 50%). ^{1}H NMR (500 MHz): δ 7.42 (t, J = 8.2 Hz, 1H), 7.13 – 7.06 (m, 2H), 4.21 (s, 2H), 2.97 (s, 3H), 1.45 (s, 9H).

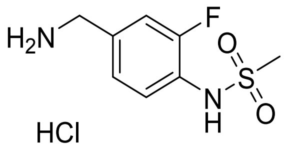

***N*-(4-(Aminomethyl)-2-fluorophenyl)methanesulfonamide hydrochloride (9.5)**

Compound was synthesized using *tert*-butyl (3-fluoro-4-(methylsulfonamido)benzyl)carbamate (186 mg, 0.58 mmol) according to general procedure C. The crude solid was washed with diethyl ether twice to furnish the desired product (148 mg, 99%). ^{1}H NMR (250 MHz, CD_3OD): δ 7.57 (t, *J* = 8.2 Hz, 1H), 7.43 – 7.24 (m, 2H), 4.13 (s, 2H), 3.03 (s, 3H).

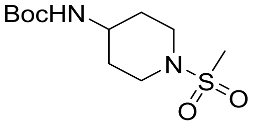

***tert*-Butyl (1-(methylsulfonyl)piperidin-4-yl)carbamate (10.2)**

Compound was synthesized using *tert*-butyl piperidin-4-ylcarbamate (500 mg, 2.50 mmol) and mesyl chloride (444 mg, 3.87 mmol) according to general procedure D. The crude was washed with diethyl ether twice to afford a white solid (661 mg, 95%). ^{1}H NMR (500 MHz, $CDCl_3$): δ 4.47 (br. s., 1H), 3.75 (d, *J* = 11.8 Hz, 2H), 3.57 (br. s., 1H), 2.73 - 2.85 (m, 5H), 2.05 (dd, *J* = 2.52, 12.89 Hz, 2H), 1.40 - 1.56 (m, 11H).

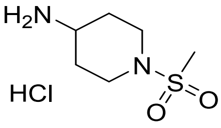

1-(Methylsulfonyl)piperidin-4-amine hydrochloride (10.3)

Compound was synthesized using *tert*-butyl (1-(methylsulfonyl)piperidin-4-yl)carbamate (596 mg, 2.14 mmol) according to general procedure C. The residual solid was washed with diethyl ether twice to furnish a pale yellow solid (429 mg, 93%). ^{1}H NMR (500 MHz, D_2O): δ 3.77 (d, *J* = 12.2 Hz, 2 H), 3.34 (t, *J* = 11.4 Hz, 1 H), 2.98 (s, 3 H), 2.91 (t, *J* = 12.1 Hz, 2 H), 2.13 (d, *J* = 12.1 Hz, 2 H), 1.61 - 1.77 (m, 2 H).

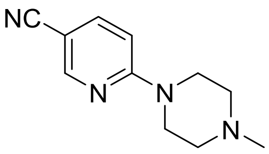

6-(4-Methylpiperazin-1-yl)nicotinonitrile (12.2b)

Compound was synthesized using 6-aminonicotinonitrile (1g, 7.2 mmol) and methyl piperazine (721 mg, 7.2 mmol) according to general procedure A. The product was used for the next step without further purification (1.39 g, 95%).

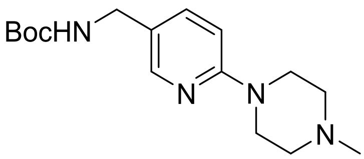

***tert*-Butyl ((6-(4-methylpiperazin-1-yl)pyridin-3-yl)methyl)carbamate (12.3b)**

Compound was synthesized using 6-(4-methylpiperazin-1-yl)nicotinonitrile (687 mg, 3.40 mmol) according to General Procedure B. The crude product was purified by silica gel chromatography (30% EtOAc:Hex) to yield the title compound as a white solid (263 mg, 75%).

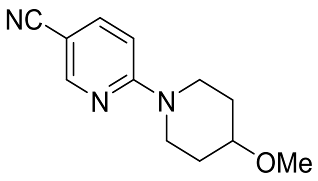

6-(4-Methoxypiperidin-1-yl)nicotinonitrile (12.2c)

A solution of 6-(4-hydroxypiperidin-1-yl)nicotinonitrile (700 mg, 3.44 mmol) in THF (15 mL) was cooled to 0 ˚C. Potassium *tert*-butoxide (410 mg, 3.70 mmol) was added portion wise ca. 10 minutes and methyl iodide (490 mg, 3.50 mmol) was slowly added. After completion, THF was removed under vacuum, crude was dissolved in EtOAc, washed with water and brine to afford a yellowish oil (720 mg, 96%). ^{1}H NMR (400 MHz, $CDCl_3$): δ 8.39 (d, *J* = 2.3 Hz, 1H), 7.58 (dd, *J* = 2.4, 9.11 Hz, 1H), 6.61 (d, *J* = 9.1 Hz, 1H), 3.97 (ddd, *J* = 3.9, 7.3, 13.1 Hz, 2H), 3.40 - 3.55 (m, 3H), 3.39 (s, 3H), 1.88 - 1.98 (m, 2H), 1.65 (dtd, *J* = 3.6, 8.32, 12.6 Hz, 2H).

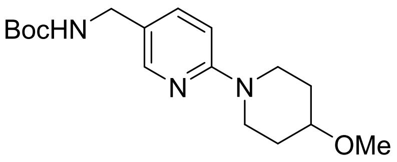

***tert*-Butyl ((6-(4-methoxypiperidin-1-yl)pyridin-3-yl)methyl)carbamate (12.3c)**

Compound was synthesized using 6-(4-methoxypiperidin-1-yl)nicotinonitrile (700 mg, 3.22 mmol) according to general procedure B. The product was purified by FCC (EtOAc:Hexanes 1:2) to afford the desired solid (870 mg, 86%). ^{1}H NMR (500 MHz, $CDCl_3$): δ 8.07 (d, *J* = 2.2 Hz, 1H), 7.43 (d, *J* = 8.0 Hz, 1H), 6.64 (d, *J* = 8.8 Hz, 1H), 4.17 (d, *J* = 4.9 Hz, 2H), 3.91 - 4.01 (m, 2H), 3.35 - 3.46 (m, 4H), 3.19 (ddd, *J* = 3.3, 9.6, 13.1 Hz, 2H), 1.92 - 2.01 (m, 2H), 1.55 - 1.67 (m, 2H), 1.45 (s, 9H).

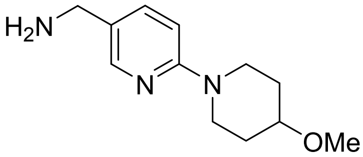

(6-(4-Methoxypiperidin-1-yl)pyridin-3-yl)methanamine (12.4c)

Compound was synthesized using *tert*-butyl ((6-(4-methoxypiperidin-1-yl)pyridin-3-yl)methyl)carbamate (870 mg, 2.70 mmol) according to general procedure C to afford the desired solid (510 mg, 85%). ^{1}H NMR (500 MHz, $CDCl_3$): δ 8.09 (d, *J* = 2.0 Hz, 1H), 7.46 (dd, *J* = 2.4, 8.7 Hz, 1H), 6.67 (d, *J* = 8.7 Hz, 1H), 3.90 - 4.01 (m, 2H), 3.73 (s, 2H), 3.35 - 3.46 (m, 4H), 3.17 (ddd, *J* = 3.2, 9.6, 13.1 Hz, 2H), 1.91 - 2.01 (m, 2H), 1.49 - 1.68 (m, 4H).

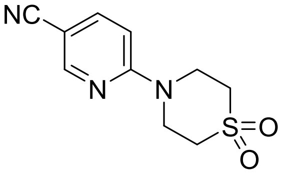

6-(1,1-Dioxidothiomorpholino)nicotinonitrile (12.2e)

Compound was synthesized using 6-chloronicotinonitrile (500 mg, 3.6 mmol) and thiomorpholine 1,1-dioxide (973 mg, 7.2 mmol) according to general procedure A. The product was washed with diethyl ether twice to afford a solid (608 mg, 71%). ^{1}H NMR (500 MHz, $CDCl_3$): δ 8.41 (d, *J* = 2.3 Hz, 1H), 7.68 (dd, *J* = 8.9, 2.3 Hz, 1H), 6.69 (d, *J* = 8.9 Hz, 1H), 4.21 – 4.15 (m, 4H), 3.03 – 2.99 (m, 4H). LC-MS (*m/z*): 238.25 $[M + H]^+$.

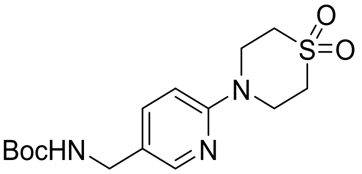

tert-Butyl ((6-(1,1-dioxidothiomorpholino)pyridin-3-yl)methyl)carbamate (12.3e) Compound was synthesized using 6-(1,1-dioxidothiomorpholino)nicotinonitrile (593 mg, 2.50 mmol) according to general procedure B. The product was washed with diethyl ether twice to furnish a solid (854 mg, 63%). ^{1}H NMR (250 MHz, $CDCl_3$): δ 8.12 (s, 1H), 7.53 (d, J = 8.1 Hz, 1H), 6.72 (d, J = 8.1 Hz, 1H), 4.81 (s, 2H), 4.22 – 4.13 (m, 4H), 3.07 – 3.00 (m, 4H), 1.45 (s, 9H).

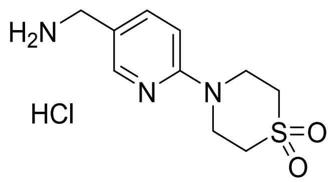

4-(5-(Aminomethyl)pyridin-2-yl)thiomorpholine 1,1-dioxide hydrochloride (12.4e) Compound was synthesized using _tert_-butyl ((6-(1,1-dioxidothiomorpholino)pyridin-3-yl)methyl)carbamate (479 mg, 1.40 mmol) according to general procedure C. Product was washed with diethyl ether twice to furnish a pale yellow solid (386 mg, 99%). ^{1}H NMR (250 MHz, D_2O): δ 8.27 – 8.14 (m, 2H), 7.53 (d, J = 9.3 Hz, 1H), 4.35 – 4.27 (m, 4H), 4.25 (s, 2H), 3.57 – 3.46 (m, 4H).

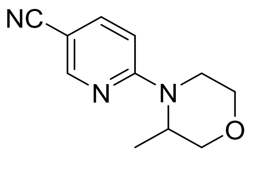

6-(3-Methylmorpholino)nicotinonitrile (12.2f)

Compound has been synthesized using 6-aminonicotinonitrile (250 mg, 1.80 mmol) and 3-methylmorpholine (402 mg, 3.97 mmol) according to General Procedure A and used as it without further purification.

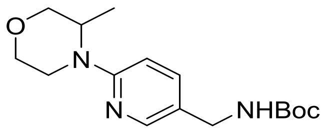

***tert*-Butyl ((6-(3-methylmorpholino)pyridin-3-yl)methyl)carbamate (12.3f)**

Compound was synthesized using 6-(3-methylmorpholino)nicotinonitrile (231 mg, 1.14 mmol) according to General Procedure B. The crude product was purified by silica gel chromatography (30% EtOAc:Hex) to yield the title compound as a white solid (263 mg, 75%). ^{1}H NMR (500 MHz, $CDCl_3$): δ 8.10 (d, *J* = 1.5 Hz, 1H), 7.47 (d, *J* = 8.8 Hz, 1H), 6.56 (d, *J* = 8.8 Hz, 1H), 4.65–4.82 (m, 1H), 4.27 (d, *J* = 6.3 Hz, 1H), 4.19 (d, *J* = 4.9 Hz, 2H), 4.01 (dd, *J* = 3.4, 11.2 Hz, 1H), 3.77–3.85 (m, 3H), 3.57–3.67 (m, 1H), 3.16–3.24 (m, 1H), 1.46 (s, 9H), 1.22 (d, *J* = 6.8 Hz, 3H).

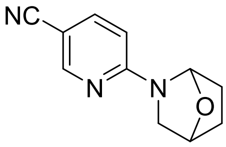

6-(7-Oxa-2-azabicyclo[2.2.1]heptan-2-yl)nicotinonitrile (12.2g)

Compound has been synthesized using 6-aminonicotinonitrile (250 mg, 1.68 mmol) and 7-oxa-2-azabicyclo[2.2.1]heptane hydrochloride (538 mg, 3.97 mmol) according to General Procedure A and used as it without further purification.

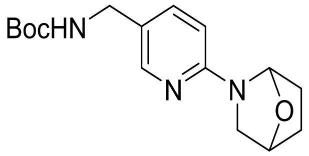

***tert*-Butyl ((6-(7-oxa-2-azabicyclo[2.2.1]heptan-2-yl)pyridin-3-yl)methyl)carbamate (12.3g)**

Compound was synthesized using 6-(7-oxa-2-azabicyclo[2.2.1]heptan-2-yl)nicotinonitrile (334 mg, 1.66 mmol) according to General Procedure B. The crude product was purified by silica gel chromatography (30% EtOAc:Hex) to yield the title compound as a white solid (336 mg, 66%). ^{1}H NMR (500 MHz, $CDCl_3$) δ 8.04 (d, *J* = 1.5 Hz, 1H), 7.43 (d, *J* = 7.8 Hz, 1H), 6.34 (d, *J* = 8.8 Hz, 1H), 4.87 (s, 1H), 4.69 (s, 1H), 4.18 (d, *J* = 4.9 Hz, 2H), 3.83–3.95 (m, 2H), 3.30–3.55 (m, 2H), 1.97 (s, 2H), 1.41–1.53 (m, 9H).

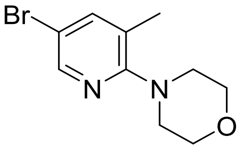

4-(5-Bromo-3-methylpyridin-2-yl)morpholine.

Compound was synthesized using 5-bromo-2-fluoro-3-methylpyridine (2 g, 10.53 mmol), morpholine (2.78 mL, 31.58 mmol) according to general procedure A. The product was washed with diethyl ether to afford a yellow solid (1 g, 37%). ^{1}H NMR (400 MHz, DMSO-d_6): δ 8.28–8.10 (m, 1H), 7.76 (dd, *J* = 0.6, 2.4 Hz, 1H), 3.82–3.62 (m, 4H), 3.08–2.98 (m, 4H), 2.24 (s, 3H).

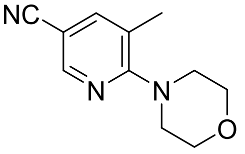

5-Methyl-6-morpholinonicotinonitrile (12.2h)

A mixture of 4-(5-Bromo-3-methylpyridin-2-yl)morpholine (450 mg, 1.75 mmol), Zn (240 mg, 3.67 mmol), $Zn(CN)_2$ (173 µL, 2.73 mmol), $Pd_2(dba)_3$ (96 mg, 0.11 mmol) and DPPF (116 mg, 0.21 mmol) in DMA (3 mL) was degassed and purged with N_2 for 3 times, and then the mixture was stirred at 150 ˚C for 12 h under N_2 atmosphere. The reaction mixture was cooled to 20 ˚C, diluted with EtOAc (15 mL), washed with 2M aqueous ammonium hydroxide (10 mL × 2), brine (10 mL) and dried over Na_2SO_4, filtered, and concentrated under reduced pressure to afford the title compound as a yellow solid (600 mg, crude). ^{1}H NMR (400 MHz, DMSO-d_6): δ 8.52 (d, J = 2.4 Hz, 1H), 7.88 (dd, J = 0.8, 2.2 Hz, 1H), 3.82–3.64 (m, 4H), 3.32–3.26 (m, 4H), 2.26 (s, 3H).

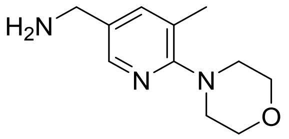

(5-Methyl-6-morpholinopyridin-3-yl)methanamine (12.4h)

To a solution of Raney-Nickel (38 mg, 0.44 mmol) in MeOH (5 mL) was added 5-methyl-6-morpholinonicotinonitrile (450 mg, 2.21 mmol) and ammonium hydroxide (1.71 mL, 11.07 mmol). The mixture was stirred at 30 ˚C for 10 h. The reaction mixture was filtered, and the filtrate concentrated in vacuo. The resultant precipitate was collected by filtration to give the title compound as a brown solid (300 mg, 65%). ^{1}H NMR (400 MHz, DMSO-d_6): δ 8.18 (d, J = 1.8 Hz, 1H), 7.66 (br d, J = 1.6 Hz, 1H), 3.94 (s, 2H), 3.76–3.70 (m, 3H), 3.08–3.04 (m, 3H), 2.94 (s, 2H), 2.26 (s, 3H), 2.00–1.88 (m, 2H).

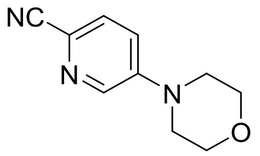

4-(6-Methylpyridin-3-yl)morpholine (12.2i)

In a sealed tube, a solution of 5-bromo-2-methylpyridine (1 g, 5.5 mmol) in toluene (20 mL) was taken. To this solution morpholine (950 mg, 11 mmol) followed by XPhos (262 mg, 0.55 mmol) and K_3PO_4 (2.3 g, 11 mmol) were added. The resulting reaction mixture was degassed by bubbling nitrogen gas through the solution for 15 min. Then $Pd_2(dba)_3$ (0.503 g, 0.55 mmol) was added, and the solution was degassed for an additional 5 min. The tube was then properly capped and the mixture was stirred at 100 °C for 16 h. After completion, the reaction was filtered through a sintered funnel and a pad of celite, concentrated, and purified by column chromatography to get desired product (600 mg, 58%). LC-MS (*m/z*): 190.41 $[M + H]^+$.

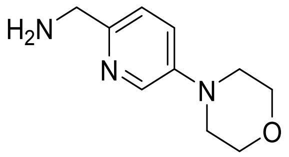

(5-Morpholinopyridin-2-yl)methanamine (12.4i)

A mixture of 4-(6-methylpyridin-3-yl)morpholine (400 mg, 2.1 mmol), Raney-Nickel (440 mg) and ammonium hydroxide (1 mL) in methanol (10 mL) was stirred under 1 atmosphere of hydrogen (balloon pressure) at room temperature for 6 h. The catalyst was removed by filtration through a pad of celite and washed with several portions of methanol. The filtrate and washings were combined and concentrated under vacuum to get desired product (350 mg, crude). LC-MS (*m/z*): 190.35 $[M + H]^+$.

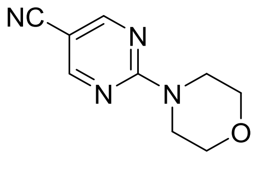

4-(5-Methylpyrimidin-2-yl)morpholine (12.2j)

Compound was synthesized using 2-chloro-5-methylpyrimidine (1 g, 7.16 mmol) morpholine (1.2 mL) according to general procedure A. The residual solid was washed with diethyl ether twice to afford the desired product (1.1 g, 79%). LC-MS (*m/z*): 191.32 $[M + H]^+$.

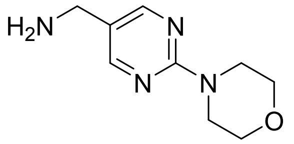

(2-Morpholinopyrimidin-5-yl)methanamine (12.4j)

A mixture of 4-(5-methylpyrimidin-2-yl)morpholine (400 mg, 2.1 mmol), Raney-Nickel (440 mg) and ammonium hydroxide (1 mL) in methanol (10 mL) was stirred under 1 atmosphere of hydrogen (balloon pressure) at room temperature for 1 h. The catalyst was removed by filtration through a pad of celite and washed with several portions of methanol. The filtrate and washings were combined and concentrated under vacuum to get desired product (300 mg, 73%).

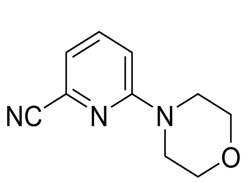

6-Morpholinopicolinonitrile (12.2k)

Compound was synthesized using 6-bromopicolinonitrile (700 mg, 5 mmol) and morpholine (660 mg, 7.6 mmol) according to general procedure A. The crude product was purified by FCC to afford the desired product (600 mg, 62%). LC-MS (*m/z*): 190.2 $[M + H]^+$.

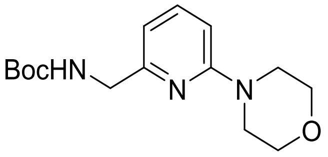

***tert*-Butyl ((6-morpholinopyridin-2-yl)methyl)carbamate (12.3k)**

Compound was synthesized using 6-morpholinopicolinonitrile (600 mg, 3.17 mmol) according to general procedure B. The product was purified by FCC to afford the desired product as a solid (700 mg, 75%). LC-MS (*m/z*): 294.3 $[M + H]^+$.

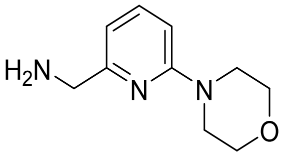

(6-Morpholinopyridin-2-yl)methanamine (12.4k)

Compound was synthesized using *tert*-butyl (6-morpholinopyridin-2-yl)methyl)carbamate (700 mg, 2.4 mmol) according to general procedure C. The crude product was washed with diethyl ether to afford the desired product. (400 mg, crude).

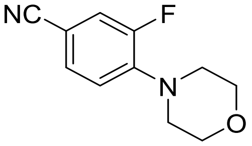

3-Fluoro-4-morpholinobenzonitrile (13.2)

In a round bottom flask, 4-amino-3-fluorobenzonitrile (300 mg, 2.2 mmol) was dissolved in 6 mL of DMF and stirred at 0 ˚C. Sodium hydride 60% dispersion in mineral oil (264 mg, 6.61 mmol) was added portion wise and stirred for 15 minutes allowing the reaction to warm up to room temperature under argon atmosphere, and then 1-bromo-2-(2-bromoethoxy)ethane (767

mg, 3.31 mmol) was added and stirred at 80 °C over 3 h. After completion, the reaction was cooled to room temperature, quenched with isopropanol diluted with EtOAc, washed with water and brine (multiple times). The combined organic layers were combined, dried with $MgSO_4$ and the product obtained was washed with diethyl ether to afford a solid (445 mg, 98%). ^{1}H NMR (250 MHz, $CDCl_3$): δ 7.37 (dd, *J* = 8.6, 2.0 Hz, 1H), 7.28 (dd, *J* = 12.6, 2.0 Hz, 1H), 6.91 (t, *J* = 8.5 Hz, 1H), 3.88 – 3.82 (m, 4H), 3.22 – 3.14 (m, 4H).

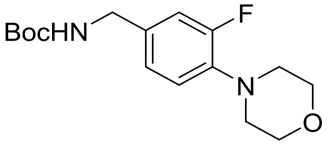

***tert*-Butyl (3-fluoro-4-morpholinobenzyl)carbamate (13.3)**

Compound was prepared from 3-fluoro-4-morpholinobenzonitrile (452 mg, 2.19 mmol) according to general procedure B. The product was washed with diethyl ether twice to afford a solid (560 mg, 82%). ^{1}H NMR (250 MHz, DMSO-d_6): δ 7.35 (t, *J* = 5.9 Hz, 1H), 6.98 (br. s, 3H), 4.05 (d, *J* = 6.1 Hz, 2H), 3.78 – 3.68 (m, 4H), 3.01 – 2.90 (m, 4H), 1.38 (s, 9H).

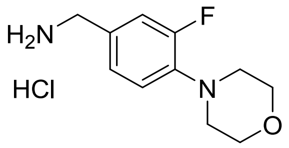

(3-Fluoro-4-morpholinophenyl)methanamine hydrochloride (13.4)

Compound was prepared from *tert*-butyl (3-fluoro-4-morpholinobenzyl)carbamate (488 mg, 1.57 mmol) according to general procedure C. The residual solid was washed with diethyl ether

to furnish the product (385 mg, 99%). ^{1}H NMR (500 MHz, D_2O): δ 7.48 (t, J = 8.5 Hz, 1H), 7.42 – 7.35 (m, 2H), 4.21 (s, 2H), 4.08 – 4.02 (m, 4H), 3.49 – 3.43 (m, 4H).

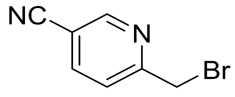

2-(Bromomethyl)-5-methylpyridine (14.2)

6-Methylnicotinonitrile (2.5 g, 21.18 mmol), NBS (5.25 g, 29.66 mmol), AIBN (52 mg, 0.318 mmol) were taken in chloroform (50 mL). The reaction mixture was stirred for 48 h at 80 °C. After completion, reaction mixture quenched by water and extracted by ethyl acetate. The organic layer was dried over Na_2SO_4, concentrated under reduced pressure to get desired product which was used for the next step (1.5 g, 36%).

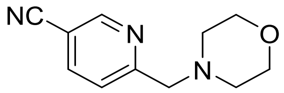

4-((5-Methylpyridin-2-yl)methyl)morpholine (14.3)

2-(Bromomethyl)-5-methylpyridine (570 mg, 2.89 mmol) was dissolved in DMF (6 mL). To this solution, K_2CO_3 (800 mg, 5.79 mmol), morpholine (0.23 mL, 2.6 mmol) were added under nitrogen and reaction mixture was stirred for 20 h at room temperature. Reaction mixture was quenched by water and extracted by ethyl acetate. Organic layer was dried over Na_2SO_4, concentrated under reduced pressure to get desired product (500 mg, 60%).

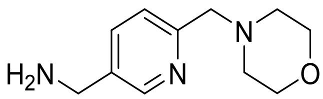

(6-(Morpholinomethyl)pyridin-3-yl)methanamine (14.4)

A mixture of 4-((5-methylpyridin-2-yl)methyl)morpholine (1 g, 4.92 mmol), Raney-Nickel (506 mg, 5.9 mmol) and ammonium hydroxide (1 mL) in methanol (20 mL) was stirred under 1 atmosphere of hydrogen (balloon pressure) at room temperature for 1 h. The catalyst was removed by filtration through a pad of celite and washed with several portions of methanol. The filtrate and washings were combined and concentrated under vacuum to get crude (500 mg, 49%) and used as such for next step. LC-MS (*m/z*): 208.1 $[M + H]^+$.

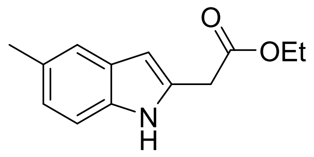

Ethyl 2-(5-methyl-1*H*-indol-2-yl)acetate (16.2)

A vial equipped with a magnetic stir bar and a rubber stopper was charged with $Pd(PhCN)_2Cl_2$ (0.3 g, 0.76 mmol), 5-methyl-1*H*-indole (1 g, 7.6 mmol), norbornene (1.4 g, 15.2 mmol), $NaHCO_3$ (2.6 g, 30.5 mmol) and capped with septa. The vial was evacuated and backfilled with argon and the process was repeated three times. A solution of water in DMF (500 mM) was added via syringe as the solvent. Under argon, ethyl bromoacetate (1.7 mL, 15 mmol) was added via syringe, and then the resulting mixture was stirred at room temperature for 10 minutes. After that, the reaction mixture was then placed in a preheated oil bath at 70 ˚C for 16 h and vigorous stirring was applied. The reaction was monitored by TLC. After completion, the reaction mixture was cooled to room temperature, diluted with ethyl acetate, washed with water (twice) and brine (once), dried over Na_2SO_4, and concentrated. The residue was directly submitted to flash column chromatography to afford desired product (400 mg, 25%).

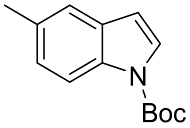

***tert*-Butyl 5-methyl-1*H*-indole-1-carboxylate (17.1)**

To a stirring solution of 5-methyl-1*H*-indole (5.0 g, 38.11 mmol) in MeCN (90 mL) was added DMAP (0.466 g, 3.812 mmol) followed by Boc_2O (9.63 mL, 41.93 mmol) in MeCN (10 mL). Stirring was continued at rt for 6 h. Excess solvent was removed and the reaction mixture was extracted with EtOAc and water. The organic layer was separated and washed with brine, dried over anhydrous sodium sulphate, and concentrated. The crude was purified by column chromatograph to get desired product (8.5 g, 96%).

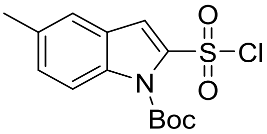

***tert*-Butyl 2-(chlorosulfonyl)-5-methyl-1*H*-indole-1-carboxylate (17.2)**

To a stirring solution of n-BuLi (4.53 mL, 9.51 mmol) in THF (15 mL) was added solution of *tert*-butyl 5-methyl-1*H*-indole-1-carboxylate (2.0 g, 8.65 mmol) in THF (10 mL) at -78 ˚C over 10 minutes and the mixture stirred for a further 25 minutes. The crude mixture was cooled to 0 ˚C and SO_2Cl_2 (0.76 mL, 9.511 mmol) was added dropwise and the mixture was stirred overnight. The solvents were removed under reduced pressure and the solids filtered through a plug of silica gel and washed with 30% DCM in hexanes. The washings were concentrated and the crude product was used as such for the next step (1 g, crude).

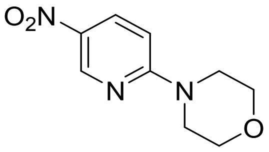

4-(5-Nitropyridin-2-yl)morpholine (18.2)

Compound was synthesized using 2-chloro-5-nitropyridine (500 mg, 3.15 mmol) and morpholine (550 mg, 6.31 mmol) according to general procedure A. Product was washed with diethyl ether twice to afford a yellow solid (628 mg, 92%). ^{1}H NMR (500 MHz, $CDCl_3$): δ ppm 9.06 (d, *J* = 2.6 Hz, 1 H), 8.25 (dd, *J* = 9.5, 2.8 Hz, 1 H), 6.58 (d, *J* = 9.5 Hz, 1 H), 3.71 - 3.87 (m, 8 H).

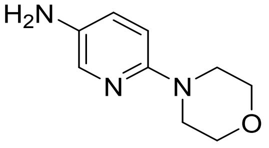

6-Morpholinopyridin-3-amine (18.3)

To a solution of 4-(5-nitropyridin-2-yl)morpholine (353 mg, 1.69 mmol) in EtOAc (20 mL) was added Pd/C 10% (180 mg) portion wise. Then degassed the reaction mixture under nitrogen atmosphere, the atmosphere was replaced with hydrogen after multiple cycles (balloon pressure) and stirred for 2 h. After completion, it was filtered through a pad of celite and washed with EtOAc. The filtrate was concentrated under reduced pressure to get desired product (284 mg, 94%). ^{1}H NMR (500 MHz, $CDCl_3$): δ 7.81 (d, *J* = 2.8 Hz, 1 H), 7.05 (dd, *J* = 8.8, 2.8 Hz, 1 H), 6.59 (d, *J* = 8.9 Hz, 1 H), 3.79 - 3.89 (m, 4 H), 3.33 - 3.40 (m, 4 H).

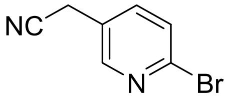

2-(6-Bromopyridin-3-yl)acetonitrile

To a solution of (6-bromopyridin-3-yl)methanol (500 mg, 2.25 mmol) and Et_3N (0.445 mL, 3.191 mmol) in DCM (10 mL) was added mesyl chloride (0.226 mL, 2.926 mmol) at 0 °C under nitrogen. The mixture was stirred at 0 °C for 1 h and at room temperature for 2 h. The reaction mixture was poured into water, extracted with dichloromethane, dried over sodium sulfate, and concentrated in vacuo to give get desired product (600 mg, 85%) and used for the next step. To a stirring solution of (6-bromopyridin-3-yl)methyl methanesulfonate (600 mg, 2.657 mmol) in DMSO (8 mL) was added sodium cyanide (156.22 mg, 3.188 mmol) and the stirring was continued at room temperature for 6 h. After completion of the reaction, the solution was extracted with EtOAc and water. The organic layer was washed with brine, dried over anhydrous sodium sulphate, and concentrated to afford the desired product (250 mg, 48%).

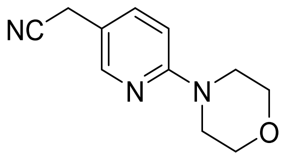

2-(6-Morpholinopyridin-3-yl)acetonitrile (19.2)

Compound was synthesized using 2-(6-bromopyridin-3-yl)acetonitrile (248 mg, 1.25 mmol) according to general procedure A. Product was purified by FCC to get desired product (160 mg, 62%). LC-MS (*m/z*): 203.8 $[M + H]^+$.

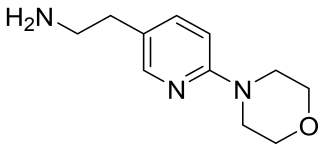

2-(6-Morpholinopyridin-3-yl)ethan-1-amine (19.3)

A mixture of 2-(6-morpholinopyridin-3-yl)acetonitrile (150.0 mg, 0.738 mmol), Raney-Nickel (90.963 mg, 1.55 mmol) and ammonium hydroxide (0.5 mL) in methanol (3.0 mL) were stirred under hydrogen atmosphere (balloon pressure) at room temperature for 16 h. The catalyst was removed by filtration through a pad of celite and washed with methanol. The filtrate was combined and concentrated under vacuum to get desired product (115 mg, crude) which was as such used for next step.

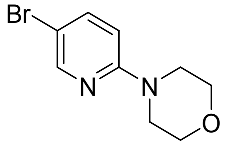

4-(5-Bromopyridin-2-yl)morpholine (20.2)

A solution of 5-bromo-2-fluoropyridine (3 g, 17 mmol) and morpholine (8 mL) was stirred at 100 °C for 16 h. Then, the reaction mixture was concentrated and purified by column chromatography to get desired product (2.9 g, 70%). LC-MS (*m/z*): 242.8 $[M + H]^+$.

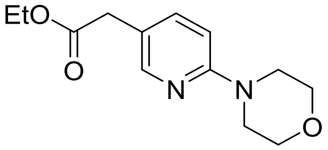

Ethyl 2-(6-morpholinopyridin-3-yl)acetate

In a sealed tube, a solution of 4-(5-bromopyridin-2-yl)morpholine (500.0 mg, 2.057 mmol) in toluene (8 mL) was taken. To this methyl 3-oxobutanoate (0.709 mL, 6.17 mmol) followed by K_3PO_4 (1.74 g, 8.227 mmol) and *t*-BuXPhos (87.33 mg, 0.206 mmol) were added. The resulting reaction mixture was degassed by bubbling nitrogen gas through the solution for 15 min. Then $Pd(OAc)_2$ (23.088 mg, 0.103 mmol) was added and the solution was degassed for an additional

5 min. The tube was then properly capped and the mixture was stirred at 120 ˚C for 20 h. After completion of the reaction it was filtered through celite, concentrated, and purified by column chromatography to get desired product (420 mg, 86%) which was used for the next step.

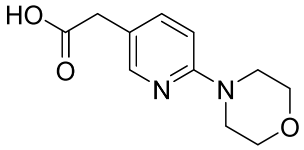

2-(6-Morpholinopyridin-3-yl)acetic acid (20.3)

Compound was synthesized using ethyl 2-(6-morpholinopyridin-3-yl)acetate (80 mg, 0.33 mmol) according to general procedure F to afford the desired compound (70 mg, 93%).

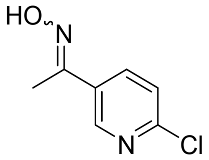

1-(6-Chloropyridin-3-yl)ethan-1-one oxime (21.2)

A solution of 1-(6-chloropyridin-3-yl)ethan-1-one (500 mg, 9.641 mmol), hydroxylamine in water (50%, 2.60 g, 38.565 mmol) and acetic acid (0.331 mL, 5.785 mmol) in dioxane (6.6 mL) was taken in a pressure vessel. The vessel was properly sealed and placed in hot oil bath at 150 ˚C and stirred the solution for 3 h. Then it was cooled down to rt, diluted with chloroform/IPA, washed with water and brine, dried over anhydrous sodium sulfate, and concentrated to get desired product (1.5 g, crude) which was directly used for next step.

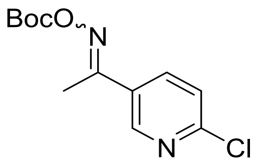

1-(6-Chloropyridin-3-yl)ethan-1-one *O*-(*tert*-butoxycarbonyl) oxime (21.3)

To a stirring solution of 1-(6-chloropyridin-3-yl)ethan-1-one oxime (1.5 g, 8.79 mmol) in DCM (30 mL) was added was added Et_3N (2.45 mL, 17.6 mmol) followed by Boc_2O (2.42 mL, 10.55 mmol) and the stirring was continued for 16 h at rt. After completion of the reaction the crude was concentrated and directly purified by column chromatography to get desired product (900 mg, 38%). LC-MS (*m/z*): 270.8 $[M + H]^+$.

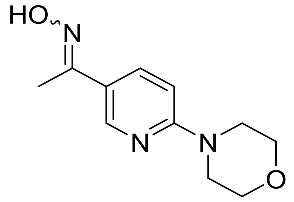

1-(6-Morpholinopyridin-3-yl)ethan-1-one oxime

Compound was synthesized using 1-(6-chloropyridin-3-yl)ethan-1-one *O*-(*tert*-butoxycarbonyl) oxime (900 mg, 3.32 mmol) and morpholine (10 mL) according to general procedure A. The residual solid was purified by FCC to furnish the desired compound (300 mg, 73%). LC-MS (*m/z*): 222.0 $[M + H]^+$.

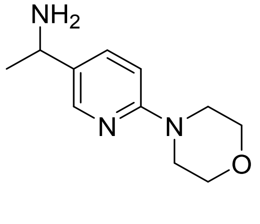

1-(6-Morpholinopyridin-3-yl)ethan-1-amine (21.4)

A mixture of 1-(6-morpholinopyridin-3-yl)ethan-1-one oxime (300 mg, 1.356 mmol), Raney-Nickel (167.117 mg, 2.847 mmol, 2.1 equiv) and ammonium hydroxide (2 mL) in methanol (8 mL) was stirred under 1 atmosphere of hydrogen at room temperature for 1 h. The catalyst was removed by filtration through a pad of celite and washed with several portions of methanol. The filtrate and washings were combined and concentrated under vacuum to get desired product (100 mg, 36%).

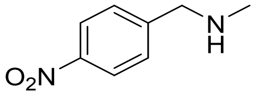

***N*-Methyl-1-(4-nitrophenyl)methanamine (23.2a)**

4-Nitrobenzaldehyde (1.51g, 10 mmol) was dissolved in dry methanol (25 mL) and added methylamine (2 mL, 20 mmol). A red-orange solution formed after 2 h, and the reaction was cooled to 0 °C and added sodium borohydride (1.10g, 30 mmol) portion wise. After completion, the reaction was quenched with AcOH solvent removed and extracted with EtOAc, washed with water and brine to obtain a brownish liquid which was used without further purification (1.6 g, 96%). ^{1}H NMR (250 MHz, $CDCl_3$): δ 8.16 (d, *J* = 8.7 Hz, 2H), 7.49 (d, *J* = 8.8 Hz, 2H), 3.85 (s, 2H), 2.45 (s, 3H).

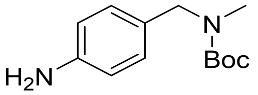

***tert*-Butyl (4-aminobenzyl)(methyl)carbamate (23.3a)**

To a solution of *N*-methyl-1-(4-nitrophenyl)methanamine (1.6 g, 9.63 mmol) in EtOAc (50 mL) was added Boc_2O (2.18 g, 10 mmol) and stirred at room temperature. No starting material was

observed after 1 h, and the atmosphere was replaced with nitrogen multiple times, and then Pd/C 10% was added. After backfilling the flask with hydrogen, the reaction was stirred at room temperature overnight. The reaction was diluted with more EtOAc, filtered through a pad of celite organic layer washed with water and brine. The organic phases were combined, dried with MgSO4, solvent removed and product was rinsed with diethyl ether to afford a white solid (1.18 g, 51%). ^{1}H NMR (500 MHz, $CDCl_3$): δ 7.03 (br. s., 2H), 6.65 (d, J = 8.3 Hz, 2H), 4.30 (br. s., 2H), 2.79 (br. s., 3H), 1.49 (s, 9H).

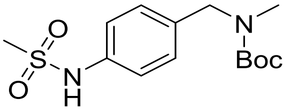

***tert*-Butyl methyl(4-(methylsulfonamido)benzyl)carbamate (23.4a)**

Compound was synthesized using *tert*-butyl (4-aminobenzyl)(methyl)carbamate (1.10g, 4.65 mmol) and mesyl chloride (520 mg, 4.6 mmol) according to general procedure D to furnish the desired product as a yellowish oil (1.40 g, 95%). ^{1}H NMR (500 MHz, $CDCl_3$): δ 7.21 (s, 4H), 6.99 (br. s., 1H), 4.39 (br. s., 2H), 3.01 (s, 3H), 2.82 (br. s., 3H), 1.48 (br. s., 9H).

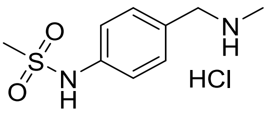

***N*-(4-((Methylamino)methyl)phenyl)methanesulfonamide hydrochloride (23.5a)** Compound was synthesized using *tert*-butyl methyl(4-(methylsulfonamido)benzyl)carbamate (1.40 g, 4.45 mmol) according to general procedure C. The crude product was washed with cold ethanol to afford the desired solid (1.04 g, 93%). ^{1}H NMR (500 MHz, DMSO-d_6): δ 9.98 (s, 1H), 9.36 (br.

s., 2H), 7.50 (d, $J = 8.5$ Hz, 2H), 7.24 (d, $J = 8.7$ Hz, 2H), 4.03 (br. s., 2H), 3.01 (s, 3H), 2.48 (br. s., 3H).

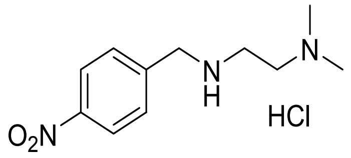

N^1,N^1-Dimethyl-N^2-(4-nitrobenzyl)ethane-1,2-diamine hydrochloride (23.2b)

4-Nitrobenzaldehyde (1.66 g, 11 mmol) was dissolved in dry methanol (30 mL) and added *N*,*N*-dimethylethylenediamine (882 mg, 10 mmol). An orange solution formed after 2 h, and the reaction was cooled to 0 °C and sodium borohydride (200 mg, 5.29 mmol) was added portion wise. After completion, the reaction was quenched with AcOH solvent removed and extracted with EtOAc, washed with water and brine. The crude, brown liquid was dissolved in THF (20.0 mL) and hydrochloric acid (2.50 mL, 10.0 mmol) was carefully added and the slurry thus formed was kept in a fridge overnight. The solid was filtered by suction, rinsed with fresh THF and dried in vacuum to give a white solid (1.76 g, 68%). ^{1}H NMR (400 MHz, D_2O): δ 8.28 (d, $J = 8.7$ Hz, 2H), 7.67 (d, $J = 8.7$ Hz, 2H), 4.34 (s, 2H), 3.42 - 3.54 (m, 4H), 2.93 (s, 6H).

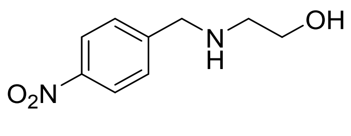

2-((4-Nitrobenzyl)amino)ethan-1-ol (23.2c)

4-Nitrobenzaldehyde (3.02 g, 20 mmol) was dissolved in dry methanol (40 mL) and added ethanolamine (1.34 g, 22 mmol). An orange solution formed after 2 h, and the reaction was cooled to 0 °C and added sodium borohydride (1.43 g, 38 mmol) portion wise. After completion,

the reaction was quenched with AcOH solvent removed and extracted with EtOAc, washed with water and brine to obtain a yellow solid which was used without further purification (3.7 g, 95%). ^{1}H NMR (250 MHz, $CDCl_3$): δ 8.14 - 8.22 (m, 2H), 7.51 (d, *J* = 8.7 Hz, 2H), 3.93 (s, 2H), 3.65 - 3.76 (m, 2H), 2.76 - 2.88 (m, 2H).

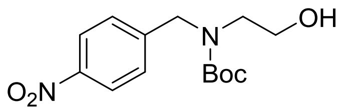

***tert*-Butyl (2-hydroxyethyl)(4-nitrobenzyl)carbamate**

To solution of 2-((4-nitrobenzyl)amino)ethan-1-ol (1.58 g, 8.1 mmol) in dry MeOH (15 mL) was added Boc_2O (1.76 g, 8.1 mmol). After completion the product was obtained as a yellow oil (2.28 g, 95%). ^{1}H NMR (250 MHz, $CDCl_3$): δ 8.20 (d, *J* = 8.5 Hz, 2H), 7.41 (d, *J* = 8.6 Hz, 2H), 4.60 (br. s., 2H), 3.65 - 3.85 (m, 2H), 3.21 - 3.54 (m, 2H), 1.45 (s, 9H).

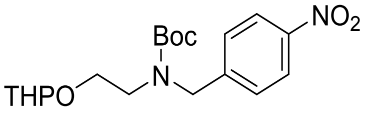

***tert*-Butyl (4-nitrobenzyl)(2-((tetrahydro-2*H*-pyran-2-yl)oxy)ethyl)carbamate**

To a solution of *tert*-butyl (2-hydroxyethyl)(4-nitrobenzyl)carbamate (2.6 g, 8.77 mmol) in dry DCM (20 mL), was added pyridinium a catalytic amount of *p*-toluenesulfonate, 3,4-*d*ihydro-2*H*-pyran (750 mg, 9.0 mmol), and stirred at room temperature. After 48 h was observed total conversion, reaction was added $NaHCO_3$ (300 mg), and solvent removed in vacuum. The crude was dissolved in EtOAc, washed with water, brine, organic layers combined and solvent

removed to furnish a yellow oil (3.70 g) which was used for the next step without further purification.

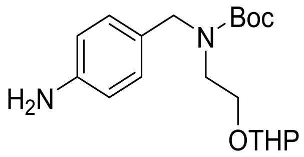

***tert*-Butyl (4-aminobenzyl)(2-((tetrahydro-2H-pyran-2-yl)oxy)ethyl)carbamate (23.3d)**

To a solution of *tert*-butyl (4-nitrobenzyl)(2-((tetrahydro-2*H*-pyran-2-yl)oxy)ethyl)carbamate (3.70 g, 8.85 mmol) in dry THF, was added Pd/C 10% (106 mg, 0.1 mmol) under nitrogen. The reaction was backfilled with hydrogen multiple times and stirred at room temperature. After completion, the crude was dissolved in EtOAc, filtered through a pad of celite and solvent removed in vacuum to furnish a yellow oil which was used for the next step (3.20 g).

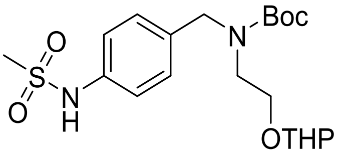

***tert*-Butyl (4-(methylsulfonamido)benzyl)(2-((tetrahydro-2H-pyran-2-yl)oxy)ethyl)carbamate (23.4c)**

Compound was synthesized using *tert*-butyl (4-aminobenzyl)(2-((tetrahydro-2*H*-pyran-2-yl)oxy)ethyl)carbamate (2.81 g, 7.63 mmol) and mesyl chloride (874 mg, 7.63 mmol) according to general procedure D to furnish a yellow oil (3.10 g, 95%).

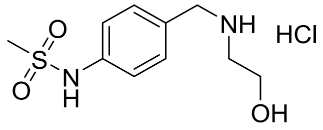

N-(4-(((2-Hydroxyethyl)amino)methyl)phenyl)methanesulfonamide hydrochloride (23.5c)

Compound was synthesized using *tert*-butyl (4-(methylsulfonamido)benzyl)(2-((tetrahydro-2*H*-pyran-2-yl)oxy)ethyl)carbamate (500 mg, 1.45 mmol) according to general procedure C. The crude product was washed with diethyl ether to afford the desired product as a solid (340 mg, 83%). ^{1}H NMR (250 MHz, CD_3OD): δ 7.44 - 7.55 (m, 2H), 7.33 (d, J = 8.5 Hz, 2H), 4.22 (s, 2H), 3.77 - 3.87 (m, 2H), 3.08 - 3.19 (m, 2H), 2.99 (s, 3H).

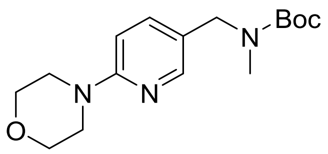

tert-Butyl methyl((6-morpholinopyridin-3-yl)methyl)carbamate (24.1a)

To a solution of *tert*-butyl ((6-morpholinopyridin-3-yl)methyl)carbamate (600 mg, 2 mmol) in dry THF (15 mL) was added potassium *tert*-butoxide (220 mg, 2 mmol) at 0 ˚C. After 10 minutes was added methyl iodide (280 mg, 2 mmol) and allowed to warm up to room temperature. After completion, the reaction was quenched with NH_4Cl solution, and THF removed. The residue was dissolved in EtOAc, washed with water and brine and product purified by FCC to afford the desired compound (596 mg, 97%). ^{1}H NMR (250 MHz, $CDCl_3$): δ 8.08 (d, J = 1.8 Hz, 1H), 7.36 - 7.55 (m, 1H), 6.63 (d, J = 8.7 Hz, 1H), 4.30 (s, 2H), 3.78 - 3.87 (m, 4H), 3.45 - 3.54 (m, 4H), 2.78 (br. s., 3H), 1.49 (s, 9H).

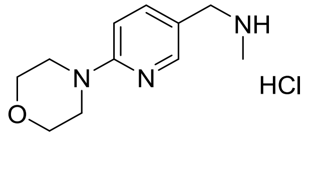

N-Methyl-1-(6-morpholinopyridin-3-yl)methanamine hydrochloride (24.2a)

Compound was synthesized using *tert*-butyl methyl((6-morpholinopyridin-3-yl)methyl)carbamate (510 mg, 1.66 mmol) according to general procedure C. The crude was washed with cold diethyl ether to afford a gray solid (395 mg, 98%). ^{1}H NMR (500 MHz, DMSO-d_6): δ 9.76 (d, J = 4.4 Hz, 2H), 8.22 - 8.31 (m, 2H), 7.46 (d, J = 9.4 Hz, 1H), 4.10 (t, J = 5.7 Hz, 2H), 3.69 - 3.84 (m, 8H), (CH_3 overlapped with DMSO).

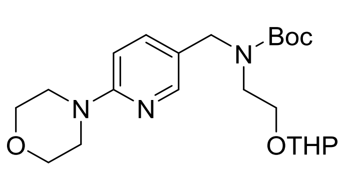

***tert*-Butyl ((6-morpholinopyridin-3-yl)methyl)(2-((tetrahydro-2H-pyran-2-yl)oxy)ethyl)carbamate (24.1b)**

A solution of *tert*-butyl ((6-morpholinopyridin-3-yl)methyl)carbamate (1.70 g, 5.79 mmol) in dry DMF (8.0 mL) was cooled to 0 ˚C and added NaH 60% dispersion in mineral oil (232 mg, 5.79 mmol). After stirring for 1 hour, 2-(2-iodoethoxy)tetrahydro-2*H*-pyran (1.48 g, 5.79 mmol) was added and the reaction stirred for an additional hour. The reaction mixture was cooled down to 0 ˚C and carefully quenched with saturated sodium hydrogen carbonate solution. The slurry was diluted with water (30 mL) and the product was extracted with diethyl ether (3 x 40 mL). The combined ethereal phase was washed with saturated sodium hydrogen carbonate solution, brine, dried over magnesium sulfate, filtrated, and concentrated in vacuum to give a bright yellow viscous liquid which was used for the next step.

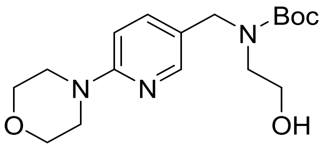

***tert*-Butyl (2-hydroxyethyl)((6-morpholinopyridin-3-yl)methyl)carbamate (24.1c)**

A solution of *tert*-butyl ((6-morpholinopyridin-3-yl)methyl)(2-((tetrahydro-2*H*-pyran-2-yl)oxy)ethyl)carbamate (2.60 g, 6.17 mmol) was dissolved in MeOH (20.0 mL). The turbid solution thus resulted received the addition of solid *p*-toluenosulfonic acid monohydrate (1.35 g, 7.09 mmol) and stirred at room temperature for 1 hour. Solid sodium acetate (0.55 g) was added to the reaction mixture and the methanol was evaporated in vacuum. The evaporation residue was dissolved in water (30 mL), pH value was set to ca. 9.5 by adding solid sodium carbonate, and the product was extracted with EtOAc (3 x 50 mL). The combined organic layers were washed with brine, dried over magnesium sulfate, filtrated and concentrated to dryness in vacuum. The brownish, viscous liquid purified by FCC and rinsed with hexane acetate (2:1, v/v, to remove the mineral oil) and then eluted with neat EtOAc. The eluate was concentrated in vacuum to give (1.0 g, 48%) of a yellow liquid. ^{1}H NMR (250 MHz, $CDCl_3$): δ 8.09 (d, *J* = 2.3 Hz, 1H), 7.38 - 7.54 (m, 1H), 6.63 (d, *J* = 8.8 Hz, 1H), 4.37 (s, 2H), 3.77 - 3.88 (m, 4H), 3.70 (br. s., 2H), 3.45 - 3.55 (m, 4H), 3.29 - 3.42 (m, 2H), 1.50 (s, 9H).

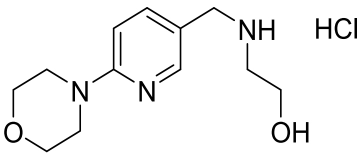

2-(((6-Morpholinopyridin-3-yl)methyl)amino)ethan-1-ol hydrochloride (24.2b)

Compound was synthesized using *tert*-butyl (2-hydroxyethyl)((6-morpholinopyridin-3-yl)methyl)carbamate (1.0 g, 2.96 mmol) according to general procedure C. Product was washed

with diethyl ether twice to afford the desired product (740 mg, 91%). ^{1}H NMR (250 MHz, D_2O): δ 7.99 - 8.10 (m, 2H), 7.26 - 7.37 (m, 1H), 4.22 - 4.29 (m, 2H), 3.80 - 3.94 (m, 6H), 3.65 - 3.75 (m, 4H), 3.17 - 3.28 (m, 2H).

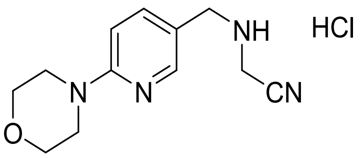

2-(((6-Morpholinopyridin-3-yl)methyl)amino)acetonitrile hydrochloride (24.2c)

Neat DIEA (152 uL, 8.7 mmol) was added to a suspension of (6-morpholinopyridin-3-yl)methanamine hydrochloride (100 mg, 4.35 mmol) in MeCN (5 mL). The suspension was shortly sonicated. The fine particulate suspension thus resulted received the addition of 2-iodoacetonitrile (72.7 mg, 4.35 mmol) and the slurry was stirred at room temperature until starting material was no longer detected by TLC. The acetonitrile was evaporated in vacuum and residue was partitioned between water and EtOAc. The pH-value of the aqueous phase was set to 10 with sodium carbonate and extracted 3 times with EtOAc. The combined organic layer was washed with brine, dried over magnesium sulfate, and concentrated to dryness in vacuum. The brownish, liquid evaporation residue was dissolved in EtOH (3.0 mL), cooled down to 0 °C and neutralized with hydrochloric acid (109 uL, 4.35 mmol). EtOH was evaporated with an air stream and the evaporation residue resuspended in acetone. The brownish supernatant was separated from the off-white precipitated. The solid was dried in vacuum to give the desired product as an off-white solid (60 mg, 51%). ^{1}H NMR (250 MHz, D_2O): δ 7.98 - 8.12 (m, 2H), 7.29 - 7.43 (m, 1H), 4.31 (s, 2H), 4.26 (s, 2H), 3.83 - 3.95 (m, 4H), 3.65 - 3.77 (m, 4H).

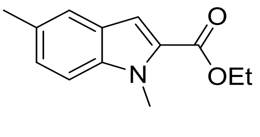

Ethyl 1,5-dimethyl-1*H*-indole-2-carboxylate (25.1b)

Ethyl 5-methyl-1*H*-indole-2-carboxylate (1.00 g, 4.92 mmol) was dissolved in dry THF (10.0 mL). The clear, colorless solution received the addition of solid potassium *tert*-butoxide (552 mg, 4.92 mmol) at room temperature and the mixture was stirred for 10 minutes. To the brownish, a clear solution thus resulted, neat iodomethane (306 uL, 4.92 mmol) was added. After a few minutes, a thick, white precipitated separated and stirring at room temperature continued for 1 hour. After completion, the reaction was quenched with saturated $NaHCO_3$ solution and THF evaporated in vacuum. The product was extracted with ethyl acetate. The organic phase was washed with brine, dried over magnesium sulfate, filtrated, and concentrated to dryness in vacuum. The crude product was dissolved in diethyl ether filtered through a silica pad. The eluate was concentrated in vacuum to yield an off-white solid (1.05 g, 98%). ^1H NMR (250 MHz, $CDCl_3$) δ 7.45 (s, 1H), 7.27 (s, 1H), 7.14 - 7.24 (m, 2H), 4.38 (q, *J* = 7.1 Hz, 2H), 2.45 (s, 3H), 1.42 (t, *J* = 7.1 Hz, 3H).

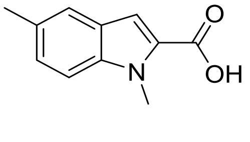

1,5-Dimethyl-1*H*-indole-2-carboxylic acid (25.2a)

Compound was synthesized using ethyl 1,5-dimethyl-1*H*-indole-2-carboxylate (1.05 g, 4.83 mmol) according to general procedure F furnishing an off-white solid (770 mg, 84%). ^1H NMR (250 MHz, DMSO-d_6): δ 7.40 - 7.48 (m, 2H), 7.15 (dd, *J* = 1.7, 8.32 Hz, 1H), 7.11 (s, 1H), 3.99 (s, 3H), 2.38 (s, 3H).

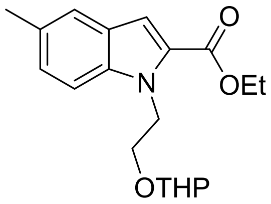

Ethyl 5-methyl-1-(2-((tetrahydro-2*H*-pyran-2-yl)oxy)ethyl)-1*H*-indole-2-carboxylate (25.1b)

To an ice-cold solution of ethyl 5-methyl-1*H*-indole-2-carboxylate (1.00 g, 4.92 mmol) and 2-(2-iodoethoxy)tetrahydro-2*H*-pyran (1.26 g, 4.92 mmol) in dry DMF (8.00 mL), solid sodium hydride (197 mg, 4.92 mmol) was added portion wise within ca. 10 minutes. The slurry was stirred overnight, whilst the temperature was allowed to rise to room temperature. After completion, the reaction mixture was cooled down to 0 °C and poured into an ice-cold, saturated sodium hydrogen carbonate solution (200 mL). The product was extracted with diethyl ether. The organic layer was washed with brine, dried over magnesium sulfate, filtrated, and concentrated in vacuum to dryness to give a yellow liquid which was used for the next step without further purification (1.4 g, 77%).

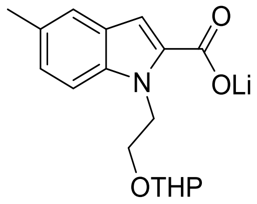

Lithium 5-methyl-1-(2-((tetrahydro-2*H*-pyran-2-yl)oxy)ethyl)-1*H*-indole-2-carboxylate (25.2b)

Ethyl 5-methyl-1-(2-((tetrahydro-2*H*-pyran-2-yl)oxy)ethyl)-1*H*-indole-2-carboxylate (1.40 g, 3.80 mmol) was dissolved in MeOH (10.0 mL), solid lithium hydroxide (182 mg, 7.60 mmol) was added and then water (2.50 mL) was combined, where upon a thick precipitated separated.

THF (10 mL) was then added and the turbid solution thus resulted was stirred at room temperature overnight. After completion, the reaction mixture was concentrated to dryness in vacuum. The dry evaporation residue was suspended in diethyl ether and the slurry thus formed was kept in the fridge for a few days. The supernatant was separated from the white precipitated. The precipitated was rinsed with diethyl ether and dried whilst the supernatant was concentrated and the precipitation process repeated furnishing a white solid (0.62 g, 53%). ^{1}H NMR (250 MHz, CD_3OD): δ 7.36 (d, *J* = 8.6 Hz, 1H), 7.31 (s, 1H), 7.02 (dd, *J* = 1.5, 8.6 Hz, 1H), 6.93 (s, 1H), 4.82 (t, *J* = 5.67 Hz, 2H), 4.40 (t, *J* = 3.2 Hz, 1H), 3.92 - 4.04 (m, 1H), 3.69 - 3.81 (m, 1H), 3.42 - 3.55 (m, 2H), 2.39 (s, 3H), 1.24 - 1.52 (m, 6H).

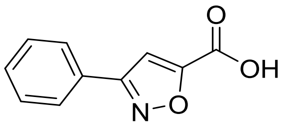

3-Phenylisoxazole-5-carboxylic acid (26.2a)

Compound was synthesized using benzaldehyde (1.50 g, 14.1 mmol) according to general procedure H, as a white solid (1.67g , 72% over 3 steps). 1H NMR (250 MHz, CD_3OD): δ 7.95 – 7.84 (m, 2H), 7.55 – 7.44 (m, 4H).

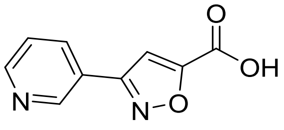

3-(Pyridin-3-yl)isoxazole-5-carboxylic acid (26.2b)

Compound was synthesized using 3-pyridinecarboxaldehyde (1.50 g, 14 mmol) according to general procedure H, as a white solid (82 mg, 12% over 3 steps). ^{1}H NMR (250 MHz, DMSO-

d$_6$): δ 9.16 (d, *J* = 1.6 Hz, 1H), 8.73 (dd, *J* = 4.8, 1.5 Hz, 1H), 8.35 (d, *J* = 8.1 Hz, 1H), 7.90 (s, 1H), 7.58 (dd, *J* = 7.8, 5.0 Hz, 1H).

Additional compounds (intermediates and final compounds *S1-S54*)

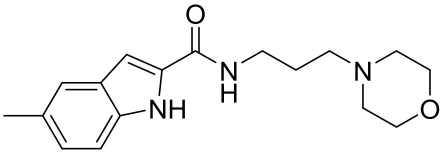

5-Methyl-*N*-(3-morpholinopropyl)-1*H*-indole-2-carboxamide (*S1*)

To a suspension of 5-methyl-1H-indole-2-carbonyl chloride (50 mg, 0.26 mmol) in DCM (2 mL) at 0 °C was added 3-morpholinopropan-1-amine (57 µL, 0.39 mmol) and triethylamine (70 µL, 0.52 mmol). The reaction was stirred at this temperature for 1 h, reaction complete by tlc (100% EtOAc). The reaction was partitioned between sat. NH_4Cl (3 mL) and EtOAc (8 mL) and separated. The organic phase was then dried ($MgSO_4$) and concentrated in vacuo to give crude product. The crude product was purified by flash chromatography (0-20% MeOH in DCM) to give desired product 5-methyl-*N*-(3-morpholinopropyl)-1*H*-indole-2-carboxamide (54 mg, 0.18 mmol, 60%); ^{1}H NMR (250 MHz, DMSO-d_6) δ 11.39 (s, 1H), 8.40 (t, *J* = 5.6 Hz, 1H), 7.37 (s, 1H), 7.30 (d, *J* = 8.4 Hz, 1H), 7.05 – 6.93 (m, 2H), 3.62 – 3.52 (m, 4H), 3.38 – 3.23 (m, 2H), 2.41 – 2.27 (m, 9H), 1.69 (p, *J* = 7.3 Hz, 2H).

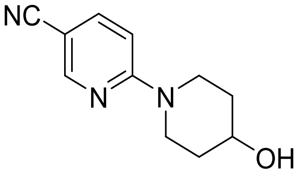

6-(4-Hydroxypiperidin-1-yl)nicotinonitrile

Compound was synthesized using 6-chloronicotinonitrile (1.0 g, 7.23 mmol) and piperidin-4-ol (730 mg, 7.23 mmol) according to general procedure A. Product was washed with diethyl ether to afford a white solid (1.37 g, 93%). ^{1}H NMR (400 MHz, $CDCl_3$): δ 8.39 (dd, *J* = 0.6,

2.32 Hz, 1H), 7.58 (dd, J = 2.3, 9.1 Hz, 1H), 6.62 (dd, J = 0.5, 9.1 Hz, 1H), 4.05 - 4.16 (m, 2H), 4.01 (td, J = 4.1, 8.0 Hz, 1H), 3.37 (ddd, J = 3.4, 9.4, 13.3 Hz, 2H), 1.91 - 2.02 (m, 2H), 1.79 (br. s., 1H), 1.50 - 1.64 (m, 2H).

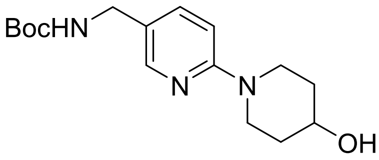

tert-Butyl ((6-(4-hydroxypiperidin-1-yl)pyridin-3-yl)methyl)carbamate

Compound was synthesized using 6-(4-hydroxypiperidin-1-yl)nicotinonitrile (660 mg, 3.24 mmol) according to general procedure B. The product was purified by FCC (EtOAc:Hexanes 1:2) to afford the desired solid (580 mg, 58%). ^{1}H NMR (400 MHz, $CDCl_3$): δ 8.06 (d, J = 2.2 Hz, 1H), 7.36 - 7.48 (m, 1H), 6.64 (d, J = 8.8 Hz, 1H), 4.79 (br. s., 1H), 4.16 (d, J = 5.3 Hz, 2H), 4.03 (td, J = 4.2, 13.4 Hz, 2H), 3.90 (tt, J = 4.2, 8.7 Hz, 1H), 3.13 (ddd, J = 3.1, 10.1, 13.2 Hz, 2H), 1.84 - 2.02 (m, 4H), 1.56 (dtd, J = 3.9, 9.4, 13.0 Hz, 1H), 1.45 (s, 9H).

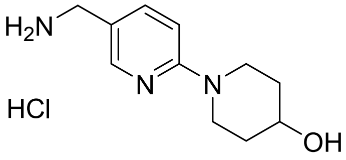

1-(5-(Aminomethyl)pyridin-2-yl)piperidin-4-ol hydrochloride

Compound was synthesized using _tert_-butyl ((6-(4-hydroxypiperidin-1-yl)pyridin-3-yl)methyl)carbamate (570 mg, 1.85 mmol) according to general procedure C. The product was washed with diethyl ether to afford the desired solid (440 mg, 98%). ^{1}H NMR (500 MHz, DMSO-d_6): δ 8.63 (br. s., 3H), 8.12 - 8.19 (m, 2H), 7.47 (d, J = 9.3 Hz, 1H), 3.94 - 4.08 (m,

5H), 3.83 (tt, *J* = 3.7, 7.6 Hz, 1H), 3.49 - 3.63 (m, 2H), 3.16 (s, 1H), 1.84 (ddd, *J* = 3.1, 6.5, 9.7 Hz, 2H), 1.39 - 1.53 (m, 1H).

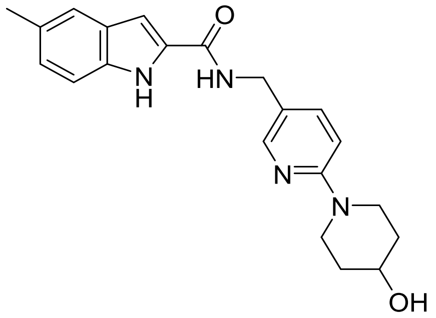

N-((6-(4-Hydroxypiperidin-1-yl)pyridin-3-yl)methyl)-5-methyl-1*H*-indole-2-carboxamide (*S2*)

Compound was synthesized using 5-methyl-1*H*-indole-2-carboxylic acid (78 mg, 0.45 mmol) and 1-(5-(aminomethyl)pyridin-2-yl)piperidin-4-ol hydrochloride (111 mg, 0.45 mmol) according to General Procedure J. The crude product was purified by FCC (0–5% $CHCl_3$:MeOH) to yield the title compound as a white solid (91 mg, 53%). ^{1}H NMR (500 MHz, CD_3OD): δ 8.09 (d, *J* = 1.9 Hz, 1H), 7.57 (dd, *J* = 2.5, 8.8 Hz, 1H), 7.35 (s, 1H), 7.31 (d, *J* = 8.12 Hz, 1H), 7.04 (dd, *J* = 1.3, 8.2 Hz, 1H), 6.97 (s, 1H), 6.79 (d, *J* = 8.8 Hz, 1H), 4.44 (s, 2H), 4.00 (td, *J* = 4.0, 13.4 Hz, 2H), 3.80 (tt, *J* = 4.6, 9.0 Hz, 1H), 3.07 (ddd, *J* = 2.8, 10.2, 13.4 Hz, 2H), 2.38 (s, 3H), 1.89 (qd, *J* = 3.8, 12.6 Hz, 2H), 1.44 - 1.55 (m, 2H). HRMS (ESI): m/z $[M+H]^+$ calcd for $C_{21}H_{25}N_4O_2$ 365.19720, found 365.19711.

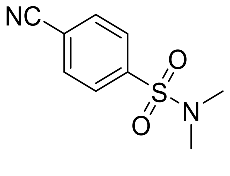

4-Cyano-*N*,*N*-dimethylbenzenesulfonamide

Compound has been synthesized using dimethylamine (415 μL, 2.98 mmol) according to General Procedure G. The crude was purified by silica gel chromatography (30% EtOAc:Hex) to give the title compound as a white solid (260 mg, 83%). ^{1}H NMR (500 MHz, $CDCl_3$): δ 7.89–7.93 (m, 2H), 7.85–7.88 (m, 2H), 2.77 (s, 6H).

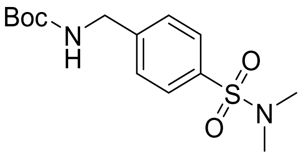

***tert*-Butyl (4-(*N,N*-*d*imethylsulfamoyl)benzyl)carbamate**

Compound was synthesized using 4-cyano-*N,N*-dimethylbenzenesulfonamide (260 mg, 1.24 mmol) according to General Procedure B. The crude product was purified by silica gel chromatography (30% EtOAc:Hex) to yield the title compound as a white solid (200 mg, 51%). ^{1}H NMR (500 MHz, $CDCl_3$): δ 7.74 (d, *J* = 7.8 Hz, 2H), 7.45 (d, *J* = 7.8 Hz, 2H), 4.41 (d, *J* = 4.9 Hz, 2H), 2.70 (s, 6H), 1.48 (br. s., 9H).

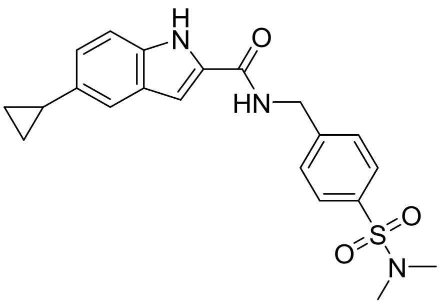

5-Cyclopropyl-*N*-(4-(*N,N*-dimethylsulfamoyl)benzyl)-1*H*-indole-2-carboxamide (*S3*)

Compound was synthesized using *tert*-butyl (4-(*N,N*-*d*imethylsulfamoyl)benzyl)carbamate. (374 mg, 1.19 mmol) and 5-cyclopropyl-1*H*-indole-2-carboxylic acid (200 mg, 1.00 mmol) according to General Procedure I. The crude product was purified by reverse phase

chromatography (30–100% MeOH:H_2O) to yield the title compound as a white solid (85 mg, 21%). ^1H NMR (500 MHz, $CDOD_3$): δ 7.77 (d, *J* = 8.3 Hz, 2H), 7.59–7.64 (m, 3H), 7.45 (d, *J* = 8.3 Hz, 1H), 7.22 (s, 1H), 7.13 (s, 1H), 7.04–7.10 (m, 1H), 4.69 (s, 2H), 2.66 (s, 6H), 1.92–2.01 (m, 1H), 0.86–0.93 (m, 2H), 0.63- 0.64 (m , 2H). LCMS $[M + H]^+$ 398.1 *m/z*.[b]

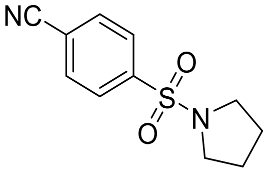

4-(Pyrrolidin-1-ylsulfonyl)benzonitrile

Compound has been synthesized using pyrrolidine (62 μL, 0.74 mmol) according to General Procedure G. The crude was purified by silica gel chromatography (15% EtOAc:Hex) to give the title compound as a white solid (171 mg, 90%). ^1H NMR (400 MHz, $CDCl_3$): δ 7.89 (d, *J* = 8.0 Hz, 2H), 7.80 (d, *J* = 8.0 Hz, 2H), 3.20 (t, *J* = 8.0 Hz, 4H), 3.22–3.19 (m, 4H).

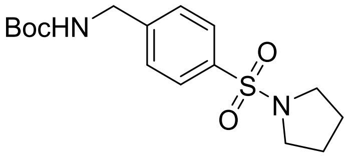

***tert*-Butyl (4-(pyrrolidin-1-ylsulfonyl)benzyl)carbamate**

Compound was synthesized using 4-(pyrrolidin-1-ylsulfonyl)benzonitrile (150 mg, 0.63 mmol) according to General Procedure B. The crude product was purified by silica gel chromatography (20% EtOAc:Hex) to yield the title compound as a white solid (130 mg, 60%). ^1H NMR (500 MHz, Acetone-d_6): δ 7.79 (d, *J* = 8.3 Hz, 2H), 7.54 (d, *J* = 8.3 Hz, 2H), 6.64 (br. s., 1H), 4.39 (d, *J* = 5.9 Hz, 2H), 3.19 (t, *J* = 6.8 Hz, 4H), 1.72 (t, *J* = 6.6 Hz, 4H), 1.43 (s, 9H).

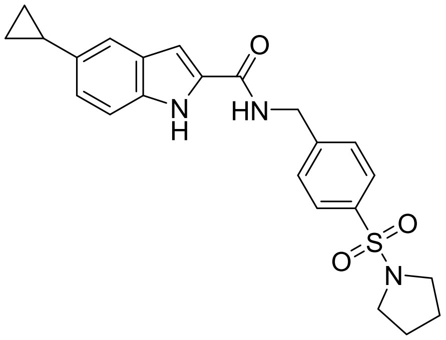

5-Cyclopropyl-*N*-(4-(pyrrolidin-1-ylsulfonyl)benzyl)-1*H*-indole-2-carboxamide (*S4*)

Compound was synthesized using 5-cyclopropyl-1*H*-indole-2-carboxylic acid (50 mg, 0.25 mmol) and (4-(pyrrolidin-1-ylsulfonyl)phenyl)methanamine (102 mg, 0.30 mmol) according to General Procedure I. The crude product was purified by reverse phase chromatography (30–100% MeOH:H_2O) to yield the title compound as a white solid (50 mg, 48%). ^{1}H NMR (500 MHz, DMSO-d_6): δ 11.52 (br. s., 1H), 9.13 (br. s., 1H), 7.78 (d, *J* = 8.3 Hz, 2H), 7.56 (d, *J* = 8.3 Hz, 2H), 7.31 (d, *J* = 10.7 Hz, 2H), 7.08 (s, 1H), 6.93 (dd, *J* = 1.5, 8.8 Hz, 1H), 4.60 (br. s., 2H), 3.12 (t, *J* = 6.6 Hz, 4H), 1.91–2.02 (m, 1H), 1.56–1.72 (m, 4H), 0.84–0.94 (m, 2H), 0.61–0.69 (m, 2H). LC−MS (m/z): 424.1 $[M+H]^+$.

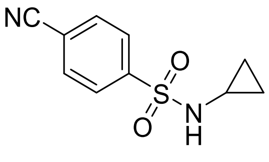

4-Cyano-*N*-cyclopropylbenzenesulfonamide

Compound has been synthesized using cyclopropylamine (52 μL, 0.74 mmol) according to General Procedure G. The crude was purified by silica gel chromatography (20% EtOAc:Hex) to give the title compound as a white solid (81 mg, 72%). ^{1}H NMR (400 MHz, $CDCl_3$): δ 8.03

(d, J = 8.6 Hz, 2H), 7.84 (d, J = 8.6 Hz, 2H), 4.98 (s, 1H), 2.30 (dddd, J = 10.9, 6.4, 3.7, 1.2 Hz, 1H), 0.64 (dddd, J = 10.9, 4.6, 2.3, 1.2 Hz, 4H).

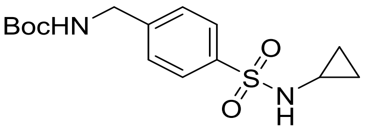

***tert*-Butyl (4-(*N*-cyclopropylsulfamoyl)benzyl)carbamate**

Compound was synthesized 4-cyano-*N*-cyclopropylbenzenesulfonamide (150 mg, 0.67 mmol) according to General Procedure B and obtained as a white solid (150 mg, 70%). ^{1}H NMR (500 MHz, Acetone-d_6): δ 7.83 (d, J = 8.3 Hz, 2H), 7.52 (d, J = 8.3 Hz, 2H), 6.73 (br. s., 1H), 6.60–6.69 (m, 1H), 4.38 (d, J = 5.9 Hz, 2H), 2.15–2.24 (m, 1H), 1.43 (s, 9H), 0.46–0.60 (m, 4H).

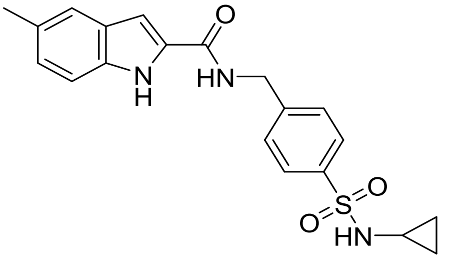

5-Cyclopropyl-*N*-(4-(*N*-cyclopropylsulfamoyl)benzyl)-1*H*-indole-2-carboxamide (S5)

Compound was synthesized using 5-cyclopropyl-1*H*-indole-2-carboxylic acid (50 mg, 0.25 mmol) and *tert*-butyl (4-(*N*-cyclopropylsulfamoyl)benzyl)carbamate (98 mg, 0.30 mmol) according to General Procedure I. The crude product was purified by reverse phase chromatography (30–100% MeOH:H_2O) to yield the title compound as a white solid (30 mg, 29%). ^{1}H NMR (500 MHz, DMSO-d_6): δ 11.50 (br. s., 1H), 9.11 (t, J = 5.9 Hz, 1H), 7.88 (br. s., 1H), 7.78 (d, J = 7.8 Hz, 2H), 7.54 (d, J = 8.3 Hz, 2H), 7.27–7.38 (m, 2H), 7.09 (s, 1H), 6.93

(d, J = 8.3 Hz, 1H), 4.59 (d, J = 5.9 Hz, 2H), 2.06 (td, J = 3.4, 6.8 Hz, 1H), 1.92–2.00 (m, 1H), 0.85–0.93 (m, 2H), 0.64 (q, J = 5.2 Hz, 2H), 0.42–0.50 (m, 2H), 0.31–0.39 (m, 2H). LC−MS (m/z): 410.2 $[M+H]^+$.

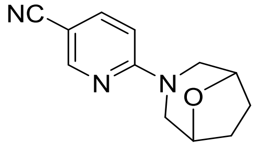

6-(8-Oxa-3-azabicyclo[3.2.1]octan-3-yl)nicotinonitrile

Compound has been synthesized using 6-aminonicotinonitrile (250 mg, 1.80 mmol) and 8-oxa-3-azabicyclo[3.2.1]octane hydrochloride (594 mg, 3.97 mmol) according to General Procedure A and used without further purification.

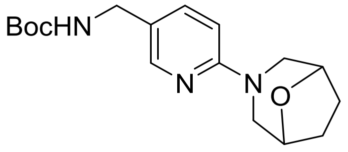

***tert*-Butyl ((6-(8-oxa-3-azabicyclo[3.2.1]octan-3-yl)pyridin-3-yl)methyl)carbamate**

Compound was synthesized using 6-(8-oxa-3-azabicyclo[3.2.1]octan-3-yl)nicotinonitrile (360 mg, 1.67 mmol) according to General Procedure B. The crude product was purified by silica gel chromatography (30% EtOAc:Hex) to yield the title compound as a white solid (376 mg, 71%). ^{1}H NMR (500 MHz, $CDCl_3$) δ 8.08 (d, J = 1.5 Hz, 1H), 7.46 (d, J = 8.3 Hz, 1H), 6.52 (d, J = 8.8 Hz, 1H), 4.63–4.84 (m, 1H), 4.50 (d, J = 2.4 Hz, 2H), 4.19 (d, J = 5.4 Hz, 2H), 3.77 (d, J = 12.2 Hz, 2H), 3.11 (dd, J = 1.9, 12.2 Hz, 2H), 1.94–2.02 (m, 2H), 1.83–1.90 (m, 2H), 1.46 (s, 9H).

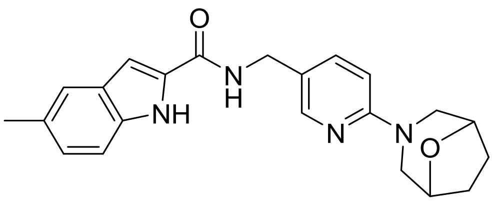

N-((6-(8-Oxa-3-azabicyclo[3.2.1]octan-3-yl)pyridin-3-yl)methyl)-5-methyl-1*H*-indole-2-carboxamide (*S6*)

Compound was synthesized using 5-methyl-1*H*-indole-2-carboxylic acid (207 mg, 1.18 mmol) (6-(8-oxa-3-azabicyclo[3.2.1]octan-3-yl)pyridin-3-yl)methanamine (377 mg, 1.18 mmol) according to General Procedure I. The crude product was purified by reverse phase chromatography (30–100% MeOH:H_2O) to yield the title compound as a white solid (42 mg, 10%). ^1H NMR (500 MHz, $CDCl_3$): δ 8.96–9.12 (m, 1H), 8.12–8.29 (m, 1H), 7.51–7.57 (m, 1H), 7.38–7.44 (m, 1H), 7.30–7.36 (m, 1H), 7.10–7.17 (m, 1H), 6.69–6.72 (m, 1H), 6.51–6.58 (m, 1H), 6.27–6.34 (m, 1H), 4.53 (s, 2H), 4.48–4.52 (m, 2H), 3.75–3.83 (m, 2H), 3.09–3.16 (m, 2H), 2.44 (s, 3H), 1.95–2.02 (m, 2H), 1.84–1.90 (m, 2H). LC−MS (m/z): 377.2 $[M+H]^+$.

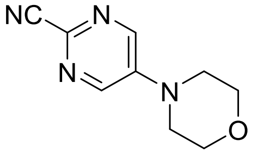

5-Morpholinopyrimidine-2-carbonitrile

In a sealed tube, a solution of 5-bromopyrimidine-2-carbonitrile (1 g, 5.5 mmol) in DMF-toluene (29 mL, 1:1) was taken. To this, morpholine (0.572 mL, 6.56 mmol) followed by Xantphos (1.582 g, 2.7 mmol) and Cs_2CO_3 (4.447 g, 13.6 mmol) were added. The resulting reaction mixture was degassed by bubbling nitrogen gas through the solution for 15 min. Then $Pd_2(dba)_3$ (0.566 g, 0.547 mmol) was added and the solution was degassed for an additional 5

min. The tube was then properly capped and the mixture was stirred at 100 °C for 16 h. After completion, the reaction it was filtered through sintered funnel with a celite pad, concentrated, and purified by column chromatography to get desired product (900 mg, 86%). LC-MS (*m/z*): 191.0 [M + H]$^+$.

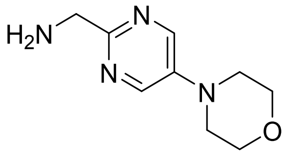

(5-Morpholinopyrimidin-2-yl)methanamine

A mixture of 5-morpholinopyrimidine-2-carbonitrile (684 mg, 3.6 mmol), Raney-Nickel (443.7 mg, 7.56 mmol) and ammonium hydroxide (3.6 mL) in methanol (20 mL) was stirred under 1 atmosphere of hydrogen (balloon pressure) at room temperature for 1 h. The catalyst was removed by filtration through a pad of celite and washed with several portions of methanol. The filtrate and washings were combined and concentrated under vacuum. The crude was purified by column chromatograph to get desired product (510 mg, 73%). LC-MS (*m/z*): 195.0 [M + H]$^+$.

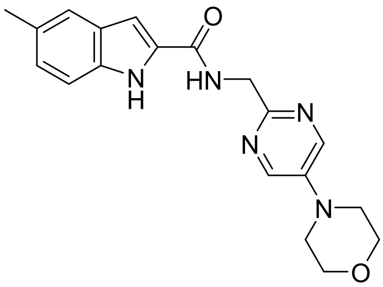

5-Methyl-*N*-((5-morpholinopyrimidin-2-yl)methyl)-1*H*-indole-2-carboxamide (*S7*)

Compound was synthesized using 5-methyl-1*H*-indole-2-carboxylic acid (400 mg, 2.3 mmol) and (5-morpholinopyrimidin-2-yl)methanamine (532.3 mg, 2.7 mmol) according to general procedure J. The crude product was purified by reverse phase preparative HPLC to afford the desired product as a white solid (70 mg, 9%). ^{1}H NMR (400 MHz, DMSO-d_6): δ 11.43 (s, 1H), 8.85 (t, J = 5.7 Hz, 1H), 8.39 (s, 2H), 7.36 (s, 1H), 7.29 (d, J = 8.4 Hz, 1H), 7.00-6.98 (m, 2H), 4.31 (d, J = 5.6 Hz, 2H), 3.68-3.61 (m, 8H), 2.35 (m, 3H). LC−MS (m/z): 352.2 [M+H]$^+$.

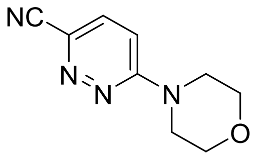

4-(6-Methylpyridazin-3-yl)morpholine

Compound was synthesized using 3-chloro-6-methylpyridazine (1.0 g, 7 mmol) and morpholine (2 mL) according to general procedure A. The product was purified by FCC to afford the desired solid which was used for the next step (700 mg, 51%).

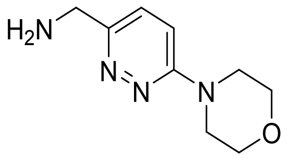

(6-Morpholinopyridazin-3-yl)methanamine

4-(6-methylpyridazin-3-yl)morpholine (350 mg, 1.84 mmol) was dissolved in methanol (10 mL). To it, Raney-Nickel (108 mg, 1.84 mmol) added. After that degassed reaction for 1 min and ammonium hydroxide (1 mL) was added. Then, under hydrogen pressure (ballon pressure), reaction was stirred for 1 h. After completion, the reaction mixture filtered through celite, and

filtrate concentrated under reduced pressure to get a crude which was used without further purification (200 mg, 55%). LC-MS (*m/z*): 195.0 $[M + H]^+$.

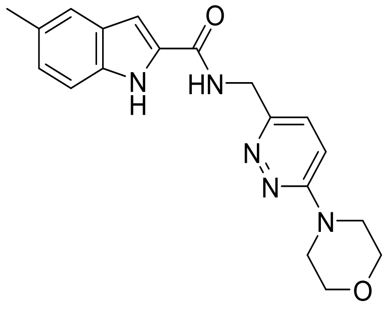

5-Methyl-*N*-((6-morpholinopyridazin-3-yl)methyl)-1*H*-indole-2-carboxamide (*S8*)

Compound was synthesized using 5-methyl-1*H*-indole-2-carboxylic acid (469 mg, 2.4 mmol) and (6-morpholinopyridazin-3-yl)methanamine (465 mg, 2.6 mmol) according to general procedure J. The crude product was purified by reverse phase preparative HPLC to afford the desired product as a white solid (120 mg, 14%). ^{1}H NMR (400 MHz, DMSO-d_6): δ 11.45 (s, 1H), 9.02 (d, J = 7.6 Hz, 1H), 7.41-7.37 (m, 2H), 7.32-7.26 (m, 2H), 7.06 (s, 1H), 7.00 (dd, J = 7.5 and 0.8 Hz, 1H), 4.62 (d, J = 5.8 Hz, 2H), 3.72 (t, J = 5.0 Hz, 4H), 3.50 (t, J = 5.0 Hz, 4H), 2.35 (s, 3H). LC−MS (m/z): 351.9 $[M+H]^+$.

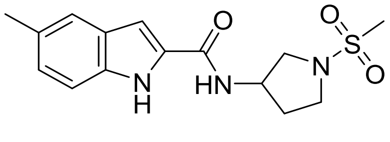

5-Methyl-*N*-((1-(methylsulfonyl)pyrrolidin-3-yl)methyl)-1*H*-indole-2-carboxamide (*S9*)

A mixture of 5-methyl-1*H*-indole-2-carboxylic acid (300 mg, 1.71 mmol) and *tert*-butyl 3-aminopyrrolidine-1-carboxylate (318 mg, 1.71 mmol) were stirred using general procedure A. The crude solid was washed with small portions of diethyl ether (2x 5 mL), the suspention was

filtered and then washed with ethyl acetate (3 mL), filtered, and dried in vacuum to afford *tert*-butyl 3-(5-methyl-1*H*-indole-2-carboxamido)pyrrolidine-1-carboxylate as a beige solid used for the next step without further purification. To the aforementioned amide product (200 mg, 0.58 mmol), DCM (2.5 mL) was added and stirred at 0 °C. A solution of TFA in DCM was added and allowed to warm up to room temperature for 1h. Volatiles were removed and to the corresponding TFA salt (146 mg), dry DCM (4.0 mL) and DIPEA (0.400 mL, 2.30 mmol) were cooled to 0 °C, and added mesyl chloride (0.057 ml, 0.74 mmol). After 3h, volatiles were evaporated in vacuum and the evaporation residue was partitioned between EtOAc and water. After phase separation, the organic layer was washed with saturated sodium bicarbonate, brine, dried over magnesium sulfate, filtrated, and concentrated to dryness in vacuum. The crude solid was washed with small portions of diethyl ether (2x 5 mL), the suspension was filtered and then washed with ethyl acetate (2x 3 mL), filtered, and dried in vacuum to afford 8 (79 mg, 32% over 3 steps) as a beige solid. ^{1}H NMR (500 MHz, DMSO-d_6): δ 11.45 (s, 1H), 8.49 (d, J = 6.5 Hz, 1H), 7.39 (s, 1H), 7.31 (d, J = 8.4 Hz, 1H), 7.11 – 7.07 (m, 2H), 7.01 (dd, J = 8.4, 1.3 Hz, 1H), 4.51 (q, J = 6.4 Hz, 1H), 3.57 (dd, J = 10.3, 6.8 Hz, 1H), 3.46 (ddd, J = 9.9, 7.7, 6.0 Hz, 2H), 3.20 (dd, J = 10.3, 5.4 Hz, 1H), 2.92 (s, 3H), 2.36 (s, 3H), 2.20 (dq, J = 12.9, 6.6 Hz, 1H), 2.08 (s, 1H), 1.99 (dq, J = 14.3, 6.9 Hz, 1H). HRMS (ESI): m/z [M+H]+ calcd for $C_{15}H_{19}N_3O_3S$ 322.12199, found 322.1266.

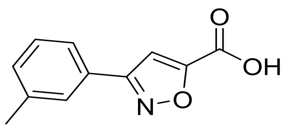

3-(*m*-Tolyl)isoxazole-5-carboxylic acid

Compound was synthesized using *m*-tolualdehyde (1.0 g, 8.32 mmol) according to general procedure H, as a beige solid (1.0 g, 59% over 3 steps). ^{1}H NMR (250 MHz, $CDCl_3$): δ 7.69 – 7.54 (m, 2H), 7.42 – 7.27 (m, 3H), 2.42 (s, 3H).

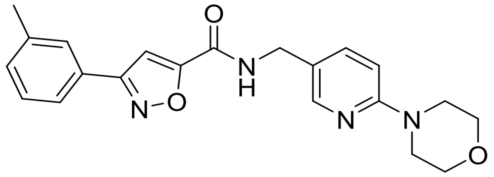

***N*-((6-Morpholinopyridin-3-yl)methyl)-3-(m-tolyl)isoxazole-5-carboxamide (*S10*)**

Compound was synthesized using 3-(m-tolyl)isoxazole-5-carboxylic acid (80 mg, 0.40 mmol) and (6-morpholinopyridin-3-yl)methanamine hydrochloride (100 mg, 0.44 mmol) according to General Procedure J. The crude product was purified by FCC (0–5% DCM:MeOH) to yield the title compound as a white solid (60 mg, 40%). ^{1}H NMR (500 MHz, DMSO-d_6): δ 9.45 (t, J = 5.9 Hz, 1H), 8.12 (d, J = 2.2 Hz, 1H), 7.74 (d, J = 1.7 Hz, 1H), 7.72 – 7.66 (m, 1H), 7.61 (s, 1H), 7.56 (dd, J = 8.7, 2.5 Hz, 1H), 7.42 (t, J = 7.6 Hz, 1H), 7.34 (d, J = 7.6 Hz, 1H), 6.82 (d, J = 8.7 Hz, 1H), 4.34 (d, J = 5.9 Hz, 2H), 3.71 – 3.65 (m, 4H), 3.43 – 3.37 (m, 4H), 2.38 (s, 3H). HRMS (ESI): m/z $[M+H]^+$ calcd for $C_{21}H_{23}N_4O_3$ 379.17647, found 379.17638.

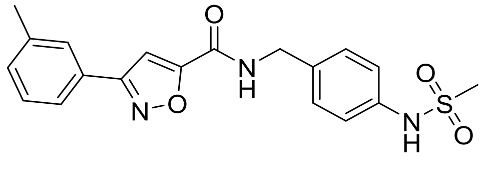

***N*-(4-(Methylsulfonamido)benzyl)-3-(m-tolyl)isoxazole-5-carboxamide (*S11*)**

Compound was synthesized using 3-(m-tolyl)isoxazole-5-carboxylic acid (80 mg, 0.39 mmol) and *N*-(4-(aminomethyl)phenyl)methanesulfonamide hydrochloride (93 mg, 0.39 mmol)

according to General Procedure J. The crude product was purified by FCC (0–5% DCM:MeOH) to yield the title compound as a beige solid (45 mg, 30%). ^{1}H NMR (500 MHz, DMSO-d_6): δ 9.69 (s, 1H), 9.52 (s, 1H), 7.75 (s, 1H), 7.70 (d, *J* = 7.7 Hz, 1H), 7.63 (s, 1H), 7.42 (t, *J* = 7.6 Hz, 1H), 7.35 (d, *J* = 7.6 Hz, 1H), 7.31 (d, *J* = 8.5 Hz, 2H), 7.18 (d, *J* = 8.5 Hz, 2H), 4.43 (d, *J* = 6.0 Hz, 2H), 2.96 (s, 3H), 2.38 (s, 3H). HRMS (ESI): m/z $[M+H]^+$ calcd for $C_{19}H_{20}N_3O_4S$ 386.11690, found 386.11652.

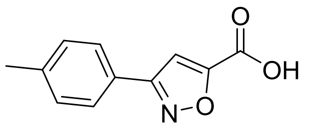

3-(*p*-Tolyl)isoxazole-5-carboxylic acid

Compound was synthesized using *p*-tolualdehyde (2.40 g, 20.0 mmol) according to general procedure H, as a white solid (827 mg, 57% over 3 steps). ^{1}H NMR (500 MHz, CD_3OD): δ 7.74 (d, *J* = 8.2 Hz, 2H), 7.40 (s, 1H), 7.29 (d, *J* = 7.9 Hz, 2H), 2.38 (s, 3H).

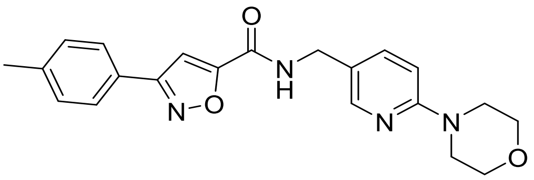

***N*-((6-Morpholinopyridin-3-yl)methyl)-3-(p-tolyl)isoxazole-5-carboxamide (*S12*)**

Compound was synthesized using 3-(p-tolyl)isoxazole-5-carboxylic acid (125 mg, 0.61 mmol) and (6-morpholinopyridin-3-yl)methanamine hydrochloride (141 mg, 0.61 mmol) according to General Procedure J. The crude product was purified by FCC (0–5% DCM:MeOH) to yield the title compound as a white solid (100 mg, 43%). ^{1}H NMR (250 MHz, $CDCl_3$): δ 8.25 (d, *J* = 2.2

Hz, 1H), 7.75 (d, J = 8.1 Hz, 2H), 7.60 (dd, J = 2.4, 8.7 Hz, 1H), 7.33 (d, J = 7.3 Hz, 2H), 7.27 (s, 1H), 6.97 (t, J = 5.1 Hz, 1H), 6.69 (d, J = 8.8 Hz, 1H), 4.58 (d, J = 5.8 Hz, 2H), 3.75 - 3.98 (m, 4H), 3.47 - 3.65 (m, 4H), 2.46 (s, 3H). HRMS (ESI): m/z $[M+H]^+$ calcd for $C_{21}H_{23}N_4O_3$ 379.17647, found 379.17630.

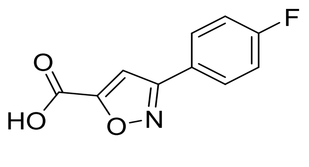

3-(4-Fluorophenyl)isoxazole-5-carboxylic acid

Compound was synthesized using 4-fluor-benzaldehyde (1.86g, 15 mmol) according to general procedure H, as a white solid (1.60 g, 49% over 3 steps). ^{1}H NMR (250 MHz, CD_3OD): δ 7.88 - 7.99 (m, 2H), 7.47 (s, 1H), 7.18 - 7.30 (m, 2H).

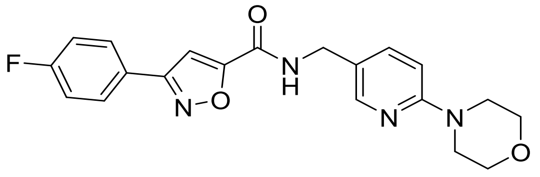

3-(4-Fluorophenyl)-*N*-((6-morpholinopyridin-3-yl)methyl)isoxazole-5-carboxamide (*S13*)

Compound was synthesized using 3-(4-fluorophenyl)isoxazole-5-carboxylic acid (160 mg, 0.77 mmol) and (6-morpholinopyridin-3-yl)methanamine hydrochloride (177 mg, 0.77 mmol) according to General Procedure J. The crude product was purified by FCC (0–5% $CHCl_3$:MeOH) to yield the title compound as a white solid (130 mg, 44%). ^{1}H NMR (500 MHz, DMSO-d_6): δ 9.48 (t, J = 5.9 Hz, 1H), 8.12 (d, J = 2.2 Hz, 1H), 7.94 - 8.01 (m, 2H), 7.64 (s,

1H), 7.55 (dd, *J* = 2.4, 8.80 Hz, 1H), 7.33 - 7.41 (m, 2H), 6.82 (d, *J* = 8.8 Hz, 1H), 4.35 (d, *J* = 6.0 Hz, 2H), 3.63 - 3.72 (m, 4H), 3.36 - 3.43 (m, 4H). HRMS (ESI): m/z $[M+H]^+$ calcd for $C_{20}H_{20}FN_4O_3$ 383.15140, found 383.15117.

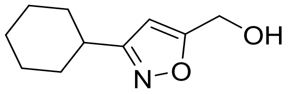

(3-Cyclohexylisoxazol-5-yl)methanol

A mixture of aldehyde (400 mg, 3.57 mmol) and hydroxylamine hydrochloride (310 mg, 4.46 mmol) in THF (2 mL) is cooled to 0 ˚C. Then, a solution of 2M NaOH (3.6 mL, 7.13 mmol) is added dropwise via addition funnel and the reaction is stirred at rt for 16 h. The reaction was then extracted with EtOAc, dried over Na_2SO_4, and concentrated in vacuo to give the imine derivative which was used in the next step without further purification. The imine was dissolved in DMF (3 mL), cooled to 0 ˚C and *N*-chlorosuccinimide (630 mg, 4.72 mmol) was added portion wise. The reaction was allowed to warm to rt and stirred for 16 h. The reaction was then quenched with water (10 mL) and extracted with EtOAc (25 mL × 3), dried over Na_2SO_4, and concentrated in vacuo to give a crude chloride which was used in the next step without further purification. The crude chloride was then dissolved in toluene (12.5 mL) and propargyl alcohol (362 µL, 6.28 mmol) was added. A solution of Na_2CO_3 (20 mL, 6.28 mmol) in water was then added and stirred for 16 h. The reaction was then extracted with EtOAc (15 × 2), dried over Na_2SO_4, and concentrated in vacuo to give product which was purified by column chromatography (0–30% EtOAc:Hex). ^1H NMR (500 MHz, $CDCl_3$) δ 6.11 (s, 1H), 4.72 (s, 2H), 2.68–2.78 (m, 1H), 2.51 (br. s., 1H), 1.95 (d, *J* = 11.2 Hz, 2H), 1.81 (d, *J* = 12.2 Hz, 2H), 1.69–1.77 (m, 1H), 1.33–1.49 (m, 4H), 1.20–1.31 (m, 1H).

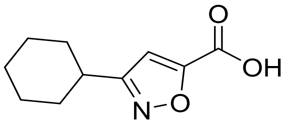

3-Cyclohexylisoxazole-5-carboxylic acid

(3-Cyclohexylisoxazol-5-yl)methanol (210 mg, 1.16 mmol) is stirred with a solution of Na_2CO_3 (29.5 mg, 0.28 mmol) in H_2O (7.5 mL). $KMnO_4$ (366 mg, 2.32 mmol) is added and the reaction stirred at rt for 2 h. Additional $KMnO_4$ (238 mg, 1.51 mmol) is added and stirring continued until complete, as monitored by LCMS. The reaction was filtered, and the filtrate extracted with DCM (10 mL × 2). The aqueous phase was acidified with HCl until pH 2 and extracted twice with diethyl ether (10 mL × 2) and concentrated to give the desired product as a white solid (100 mg, 50%). The compound was used as it without any further purification.

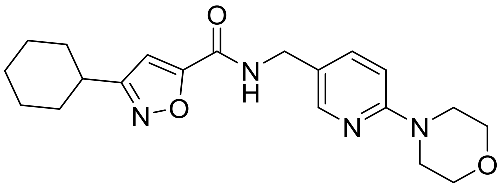

3-Cyclohexyl-*N*-((6-morpholinopyridin-3-yl)methyl)isoxazole-5-carboxamide (*S14*)

Compound was synthesized using 3-cyclohexylisoxazole-5-carboxylic acid (100 mg, 0.51 mmol) and (6-morpholinopyridin-3-yl)methanamine hydrochloride (100 mg, 0.44 mmol) according to General Procedure J. The crude product was purified by FCC (0–5% DCM:MeOH) to yield the title compound as a white solid (50 mg, 31%). ^{1}H NMR (500 MHz, $CDCl_3$): δ 8.17 (d, *J* = 1.9 Hz, 1H), 7.52 (dd, *J* = 2.2, 8.5 Hz, 1H), 6.80 (s, 1H), 6.76 (br. s., 1H), 6.63 (d, *J* = 8.8 Hz, 1H), 4.50 (d, *J* = 5.9 Hz, 2H), 3.79–3.86 (m, 4H), 3.46–3.57 (m, 4H), 2.74–2.83 (m,

1H), 1.97 (d, *J* = 11.2 Hz, 2H), 1.83 (d, *J* = 12.2 Hz, 2H), 1.74 (d, *J* = 12.7 Hz, 1H), 1.34–1.51 (m, 4H), 1.22–1.32 (m, 1H). LC−MS (m/z): 371.2 $[M+H]^+$.

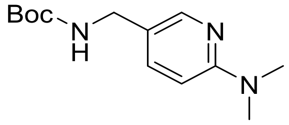

tert-Butyl ((6-(dimethylamino)pyridin-3-yl)methyl)carbamate

Compound was synthesized using 6-(dimethylamino)nicotinonitrile (181 mg, 1.23 mmol) according to General Procedure B and used as it without further purification (197 mg, 64%). ^1H NMR (500 MHz, $CDCl_3$) δ 8.08 (d, *J* = 1.5 Hz, 1H), 7.43 (d, *J* = 8.3 Hz, 1H), 6.51 (d, *J* = 8.8 Hz, 1H), 4.48–4.82 (m, 1H), 4.18 (d, *J* = 5.4 Hz, 2H), 3.09 (s, 6H), 1.47 (s, 9H).

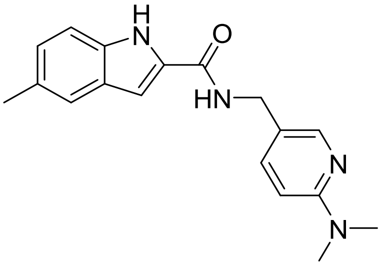

***N*-((6-(Dimethylamino)pyridin-3-yl)methyl)-5-methyl-1*H*-indole-2-carboxamide (*S15*)**

Compound was synthesized using *tert*-butyl ((6-(dimethylamino)pyridin-3-yl)methyl)carbamate (297 mg, 1.18 mmol) and 5-methyl-1*H*-indole-2-carboxylic acid (207 mg, 1.18 mmol) according to General Procedure I. The crude product was purified by reverse phase chromatography (30–100% MeOH:H_2O) to yield the title compound as a white solid (85 mg, 21%). ^1H NMR (500 MHz, $CDCl_3$): δ 9.34 (br. s., 1H), 8.16 (d, *J* = 1.5 Hz, 1H), 7.51 (dd, *J* = 2.4, 8.8 Hz, 1H), 7.40 (s, 1H), 7.33 (d, *J* = 8.3 Hz, 1H), 7.12 (d, *J* = 8.8 Hz, 1H), 6.73 (d, *J* = 1.5

Hz, 1H), 6.51 (d, J = 8.8 Hz, 1H), 6.41 (br. s., 1H), 4.53 (d, J = 5.9 Hz, 2H), 3.09 (s, 6H), 2.44 (s, 3H). LCMS $[M + H]^+$ 309.2 *m/z.*

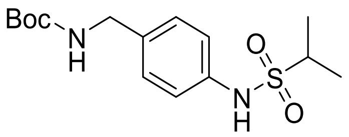

tert-Butyl (4-((1-methylethyl)sulfonamido)benzyl)carbamate

Compound was synthesized using *tert*-butyl (4-aminobenzyl)carbamate (1 g, 4.50 mmol) and propane-2-sulfonyl chloride (601 μL, 5.40 mmol) according to General Procedure D and used as it without further purification (1.12 g, 76%). ^{1}H NMR (400 MHz, DMSO-d_6): δ 8.34 (d, J = 7.8 Hz, 1H), 7.60 (d, J = 4.2 Hz, 1H), 7.27 (d, J = 4.0 Hz, 1H), 3.95–3.86 (m, 2H), 2.97–2.67 (m, 2H), 2.50 (s, 1H), 2.55–2.43 (m, 1H), 1.77 (br dd, J = 2.8, 12.4 Hz, 2H), 1.40 (s, 8H), 1.38 (br d, J = 4.4 Hz, 1H), 1.36–1.28 (m, 1H).

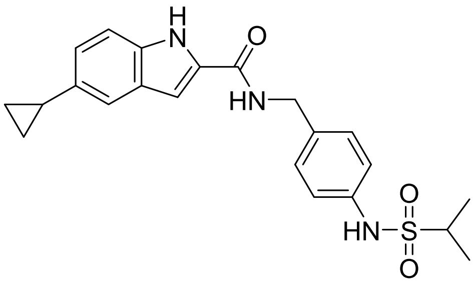

5-Cyclopropyl-*N*-(4-((1-methylethyl)sulfonamido)benzyl)-1*H*-indole-2-carboxamide (*S16*)

Compound was synthesized using *tert*-butyl (4-((1-methylethyl)sulfonamido)benzyl)carbamate (164 mg, 0.50 mmol) and 5-cyclopropyl-1*H*-indole-2-carboxylic acid (100 mg, 0.50 mmol) according to General Procedure J. The crude product was purified by flash silica gel chromatography (0–30% EtOAc:PE) to yield the title compound as a white solid (70 mg, 38%).

^{1}H NMR (500 MHz, DMSO-d_6): δ 11.44 (s, 1H), 9.69 (br. s., 1H), 8.92 (t, *J* = 6.1 Hz, 1H), 7.28–7.32 (m, 2H), 7.24 (d, *J* = 8.3 Hz, 2H), 7.16 (d, *J* = 8.8 Hz, 2H), 7.05 (d, *J* = 1.0 Hz, 1H), 6.89–6.94 (m, 1H), 4.42 (d, *J* = 6.3 Hz, 2H), 3.09–3.20 (m, 1H), 1.92–2.00 (m, 1H), 1.20 (d, *J* = 6.8 Hz, 6H), 0.86–0.92 (m, 2H), 0.62–0.66 (m, 2H). LCMS [M + H]$^+$ 412.1 *m/z*.[b]

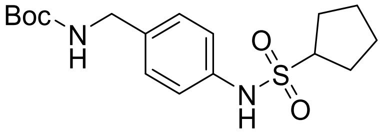

***tert*-Butyl (4-(cyclopentanesulfonamido)benzyl)carbamate**

Compound was synthesized using *tert*-butyl (4-aminobenzyl)carbamate (200 mg, 0.90 mmol) and cyclopentanesulfonyl chloride (601 µL, 1.08 mmol) according to General Procedure D and used as it without further purification (240 mg, 75%).

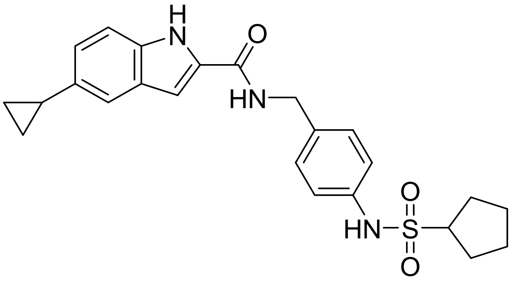

***N*-(4-(Cyclopentanesulfonamido)benzyl)-5-cyclopropyl-1*H*-indole-2-carboxamide 2,2,2-trifluoroacetate (*S17*)**

Compound was synthesized *tert*-butyl (4-(cyclopentanesulfonamido)benzyl)carbamate. (92 mg, 0.26 mmol) and 5-cyclopropyl-1*H*-indole-2-carboxylic acid (40 mg, 0.26 mmol) according to General Procedure J. The crude product was purified by preparative reverse phase HPLC using 20−65% MeCN:water (TFA) system to give the title compound as a white solid (37 mg, 42%). ^{1}H NMR (500 MHz, DMSO-d_6): δ 11.41–11.47 (m, 1H), 8.85–8.95 (m, 1H), 7.29 (d, *J*

= 6.8 Hz, 2H), 7.21 (d, *J* = 8.3 Hz, 2H), 7.11 (d, *J* = 8.3 Hz, 2H), 7.05 (s, 1H), 6.88–6.94 (m, 1H), 4.41 (d, *J* = 6.3 Hz, 2H), 3.38–3.46 (m, 1H), 1.92–2.01 (m, 1H), 1.74–1.91 (m, 4H), 1.57–1.67 (m, 2H), 1.43–1.53 (m, 2H), 0.89 (dd, *J* = 2.0, 8.3 Hz, 2H), 0.61–0.67 (m, 2H). LCMS $[M + H]^+$ 438.2 *m/z.*

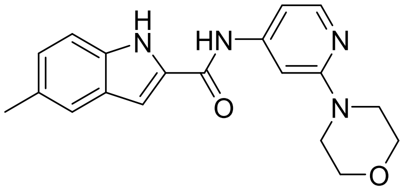

5-Methyl-*N*-(2-morpholinopyridin-4-yl)-1*H*-indole-2-carboxamide (*S18*)

To a solution of 5-methyl-1*H*-indole-2-carboxylic acid (200 mg, 0.73 mmol) in MeCN (0.2 mL) was added chloro-*N*,*N*,*N'*,*N'*-tetramethylformamidinium hexafluorophosphate (TFCH) (408 mg, 1.45 mmol) and NMI (232 µL, 2.91 mmol) at 20 ˚C for 0.5 h. Then, 2-morpholinopyridin-4-amine (130.20 mg, 0.73 mmol) was added. The mixture was stirred at 50 ˚C for 2 h. After cooling to rt, the reaction mixture was concentrated. The residue was diluted with water (20 mL) and extracted with EtOAc (20 mL × 3). The combined organic layers were dried over Na_2SO_4, filtered and dried. The residue was purified by preparative reverse phase HPLC using 1−60% MeCN:water(FA) system to afford the title compound as white solid (12.5 mg, 5%). ^{1}H NMR (500 MHz, CD_3OD): δ 8.00–8.04 (m, 1H), 7.42–7.46 (m, 2H), 7.34–7.39 (m, 1H), 7.26 (s, 1H), 7.14–7.18 (m, 1H), 7.10–7.13 (m, 1H), 3.78–3.90 (m, 4H), 3.42–3.56 (m, 4H), 2.42 (s, 3H). LCMS $[M + H]^+$ 337.1 *m/z.*

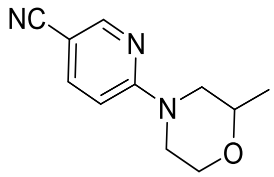

6-(2-Methylmorpholino)nicotinonitrile

Compound has been synthesized using 6-aminonicotinonitrile (250 mg, 1.80 mmol) and 2-methylmorpholine (402 mg, 3.97 mmol) according to General Procedure A. The crude was purified by reverse phase chromatography (10% MeOH:CH_2Cl_2) to give the title compound as a pale-yellow solid (230 mg, 63%). ^{1}H NMR (500 MHz, $CDCl_3$) δ 8.42 (d, *J* = 1.9 Hz, 1H), 7.64 (dd, *J* = 2.4, 8.8 Hz, 1H), 6.60 (d, *J* = 9.3 Hz, 1H), 4.23 (d, *J* = 13.2 Hz, 1H), 4.13 (d, *J* = 13.2 Hz, 1H), 4.03 (td, *J* = 1.7, 11.7 Hz, 1H), 3.59–3.71 (m, 2H), 3.09 (dt, *J* = 3.4, 12.4 Hz, 1H), 2.73 (dd, *J* = 10.5, 12.9 Hz, 1H), 1.27 (d, *J* = 6.3 Hz, 3H).

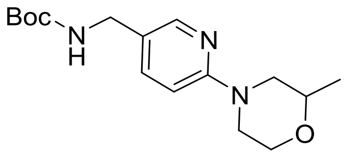

***tert*-Butyl ((6-(2-methylmorpholino)pyridin-3-yl)methyl)carbamate**

Compound was synthesized using 6-(2-Methylmorpholino)nicotinonitrile (350 mg, 1.72 mmol) according to General Procedure B. The crude product was purified by silica gel chromatography (30% EtOAc:Hex) to yield the title compound as a white solid (263 mg, 75%). ^{1}H NMR (400 MHz, DMSO-d_6): δ 7.98 (s, 1H), 7.43 (dd, *J* = 1.9, 8.6 Hz, 1H), 7.31 (t, *J* = 5.6 Hz, 1H), 6.80 (d, *J* = 8.6 Hz, 1H), 4.01–4.11 (m, 1H), 3.97 (d, *J* = 5.6 Hz, 2H), 3.89 (dd, *J* = 2.9, 11.1 Hz, 1H), 3.46–3.60 (m, 2H), 2.73 (dt, *J* = 3.5, 12.3 Hz, 1H), 2.35–2.44 (m, 1H), 1.99 (s, 1H), 1.37 (s, 9H), 1.14 (d, *J* = 6.2 Hz, 3H).

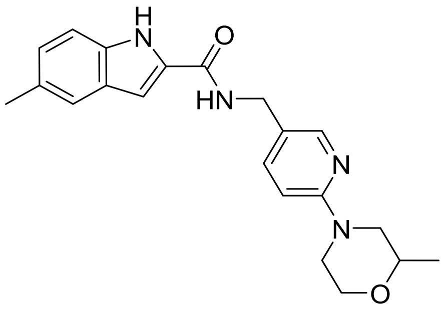

5-Methyl-*N*-((6-(2-methylmorpholino)pyridin-3-yl)methyl)-1*H*-indole-2-carboxamide (*S19*)

Compound was synthesized using *tert*-butyl ((6-(2-methylmorpholino)pyridin-3-yl)methyl)carbamate (209 mg, 0.68 mmol) and 5-methyl-1*H*-indole-2-carboxylic acid (55 mg, 0.31 mmol) according to General Procedure I. The crude product was purified by reverse phase chromatography (30–100% MeOH:H_2O) to yield the title compound as a white solid (50 mg, 24%). ^{1}H NMR (500 MHz, Acetone-d_6): δ 10.64 (br. s., 1H), 8.12–8.20 (m, 2H), 7.59 (dd, *J* = 2.2, 8.6 Hz, 1H), 7.43 (d, *J* = 8.3 Hz, 1H), 7.37 (s, 1H), 7.06 (d, *J* = 8.3 Hz, 1H), 7.00–7.02 (m, 1H), 6.76 (d, *J* = 8.6 Hz, 1H), 4.48 (d, *J* = 5.9 Hz, 2H), 4.13 (d, *J* = 12.3 Hz, 1H), 4.04 (d, *J* = 12.7 Hz, 1H), 3.89–3.94 (m, 1H), 3.54–3.62 (m, 2H), 2.79 (dt, *J* = 3.7, 12.3 Hz, 1H), 2.45 (dd, *J* = 10.5, 12.3 Hz, 1H), 2.38 (s, 3H), 1.17 (d, *J* = 6.3 Hz, 3H). LCMS $[M + H]^+$ 365.2 *m/z.*

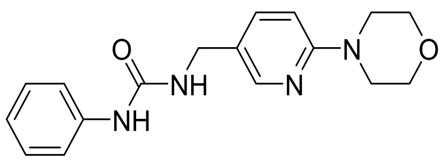

1-((6-Morpholinopyridin-3-yl)methyl)-3-phenylurea (*S20*)

Compound was synthesized using aniline (50 mg, 0.54 mmol) and 6-Morpholinopyridin-3-yl)methanamine (166 mg, 0.86 mmol) according to general procedure K. The resulting mixture was purified by reverse phase chromatography (30–100% MeOH:H_2O) to afford the title compound as a white solid (50 mg, 30%). ^{1}H NMR (500 MHz, Acetone-d_6): δ 8.14 (s, 1H), 7.92

(br. s., 1H), 7.57 (d, *J* = 8.8 Hz, 1H), 7.48 (d, *J* = 7.8 Hz, 2H), 7.21 (t, *J* = 8.1 Hz, 2H), 6.88–6.95 (m, 1H), 6.76 (d, *J* = 8.8 Hz, 1H), 6.12 (br. s., 1H), 4.28 (d, *J* = 5.9 Hz, 2H), 3.69–3.75 (m, 4H), 3.41–3.48 (m, 4H). LCMS $[M + H]^+$ 313.1 *m/z*

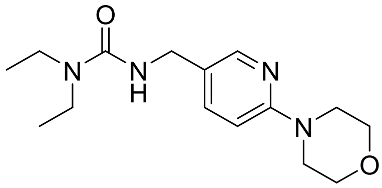

1,1-Diethyl-3-((6-morpholinopyridin-3-yl)methyl)urea (*S21*)

Compound was synthesized using diethylamine (424 µL, 4.10 mmol) according to General Procedure K. The crude product was purified by reverse phase chromatography (30–100% MeOH:H_2O) to yield the title compound as a pale yellow solid (120 mg, 24%). ^{1}H NMR (500 MHz, CD_3OD): δ 8.05 (d, *J* = 1.5 Hz, 1H), 7.55 (dd, *J* = 2.4, 8.8 Hz, 1H), 6.79 (d, *J* = 8.3 Hz, 1H), 4.24 (s, 2H), 3.76–3.80 (m, 4H), 3.40–3.44 (m, 4H), 3.26–3.30 (m, 4H), 1.11 (t, *J* = 7.1 Hz, 6H). LCMS $[M + H]^+$ 293.2 *m/z*.[b]

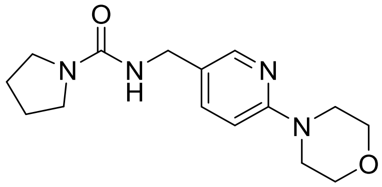

***N*-((6-Morpholinopyridin-3-yl)methyl)pyrrolidine-1-carboxamide (*S22*)**

Compound was synthesized using pyrrolidine (346 µL, 4.22 mmol) according to General Procedure K. The crude product was purified by reverse phase chromatography (30–100% MeOH:H_2O) to yield the title compound as a white solid (50 mg, 26%). ^{1}H NMR (500 MHz, Acetone-d_6): δ 8.10 (s, 1H), 7.54 (dd, *J* = 2.4, 8.8 Hz, 1H), 6.72 (d, *J* = 8.3 Hz, 1H), 5.83 (br. s.,

1H), 4.21 (d, *J* = 5.9 Hz, 2H), 3.69–3.76 (m, 4H), 3.41–3.48 (m, 4H), 3.29 (t, *J* = 6.6 Hz, 4H), 1.81–1.89 (m, 4H). LCMS $[M + H]^+$ 291.1 *m/z*.

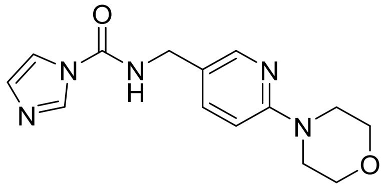

N-((6-Morpholinopyridin-3-yl)methyl)-1H-imidazole-1-carboxamide (*S23*)

To a solution of (6-morpholinopyridin-3-yl)methanamine hydrochloride (153 mg, 0.66 mmol) in water (6 mL) was added CDI (130 mg, 0.79 mmol) and stirred at room temperature overnight. The product precipitated and was filtered as a white solid (76 mg, 40%). ^{1}H NMR (250 MHz, DMSO-d_6): δ 8.95 (t, *J* = 6.1 Hz, 1H), 8.24 (s, 1H), 8.13 (d, *J* = 2.4 Hz, 1H), 7.67 (d, *J* = 1.5 Hz, 1H), 7.56 (dd, *J* = 8.7, 2.4 Hz, 1H), 7.02 (s, 1H), 6.83 (d, *J* = 8.8 Hz, 1H), 4.33 (d, *J* = 5.6 Hz, 2H), 3.74 – 3.63 (m, 4H), 3.46 – 3.36 (m, 4H). HRMS (ESI): m/z [M+H]+ calcd for $C_{14}H_{18}N_5O_2$ 288.14550 found 288.14484.

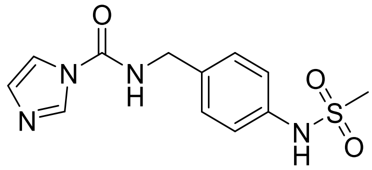

N-(4-(Methylsulfonamido)benzyl)-1H-imidazole-1-carboxamide (*S24*)

To a solution of (N-(4-(aminomethyl)phenyl)methanesulfonamide hydrochloride (150 mg, 0.63 mmol) in water (8 mL) was added CDI (123 mg, 0.76 mmol) and stirred at room temperature overnight. The product precipitated and was filtered as a white solid (126 mg, 68%). ^{1}H NMR (250 MHz, DMSO-d_6): δ 9.71 (s, 1H), 9.04 (t, *J* = 5.8 Hz, 1H), 8.27 (t, *J* = 1.1 Hz, 1H), 7.70 (t,

J = 1.4 Hz, 1H), 7.32 (d, *J* = 8.5 Hz, 2H), 7.24 – 7.12 (m, 2H), 7.04 (s, 1H), 4.41 (d, *J* = 5.7 Hz, 2H), 2.96 (s, 3H). HRMS (ESI): m/z $[M+H]^+$ calcd for $C_{12}H_{15}N_4O_3S$ 295.08594, found 295.08535.

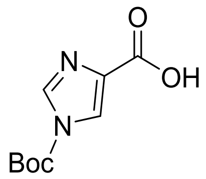

1-(*tert*-Butoxycarbonyl)-1*H*-imidazole-4-carboxylic acid

To a stirring solution of 1*H*-imidazole-4-carboxylic acid (500 mg, 4.46 mmol) in MeOH (15 mL) was added was added DIPEA (0.77 ml, 4.46 mmol) followed by Boc_2O (1.07 g, 4.91 mmol), and the stirring was continued for 16 h at rt. After completion, solvent was removed a d crude, was acidified with 5% H_2SO_4 at pH~5 and extracted with EtOAc twice. The combined organic layers were washed with brine, dried with Na_2SO_4 and the solvent evaporated. The product was isolated as white solid (776 mg, 82%). ^{1}H NMR (250 MHz, $CDCl_3$): δ 9.06 (s, 1H), 8.20 (d, *J* = 1.4 Hz, 1H), 8.10 (d, *J* = 1.4 Hz, 1H), 1.64 (s, 9H).

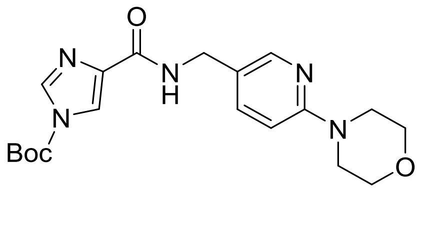

***tert*-Butyl 4-(((6-morpholinopyridin-3-yl)methyl)carbamoyl)-1*H*-imidazole-1-carboxylate**

Compound was synthesized using 1-(tert-butoxycarbonyl)-1*H*-imidazole-4-carboxylic acid (200 mg, 0.94 mmol) and (6-morpholinopyridin-3-yl)methanamine hydrochloride (238 mg, 1.04 mmol) according to general procedure J. The product was washed with diethyl ether to

afford a white solid (150 mg, 41%). ^{1}H NMR (250 MHz, DMSO-d_6): δ 8.70 (t, J = 6.2 Hz, 1H), 8.28 (d, J = 1.4 Hz, 1H), 8.08 (d, J = 2.4 Hz, 1H), 7.88 (d, J = 1.4 Hz, 1H), 7.52 (dd, J = 8.7, 2.4 Hz, 1H), 6.79 (d, J = 8.7 Hz, 1H), 4.28 (d, J = 6.2 Hz, 2H), 3.75 – 3.62 (m, 4H), 3.42 – 3.35 (m, 4H), 1.58 (s, 9H).

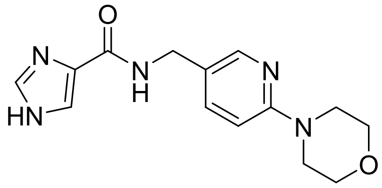

***N*-((6-Morpholinopyridin-3-yl)methyl)-1*H*-imidazole-4-carboxamide (*S25*)**

Compound was synthesized using *tert*-butyl 4-(((6-morpholinopyridin-3-yl)methyl)carbamoyl)-1*H*-imidazole-1-carboxylate (110 mg, 0.28 mmol) according to General Procedure C. The product was washed with diethyl ether twice to yield the title compound as a pale yellow solid (78 mg, 96%). ^{1}H NMR (250 MHz, DMSO-d_6): δ 9.87 (t, J = 5.8 Hz, 1H), 9.11 (d, J = 1.2 Hz, 1H), 8.38 (d, J = 1.2 Hz, 1H), 8.09 – 7.94 (m, 2H), 7.33 (d, J = 9.2 Hz, 1H), 4.41 (d, J = 5.8 Hz, 2H), 3.78 – 3.71 (m, 4H), 3.70 – 3.65 (m, 4H). HRMS (ESI): m/z $[M+H]^+$ calcd for $C_{14}H_{18}N_5O_2$ 288.14550, found 288.14539.

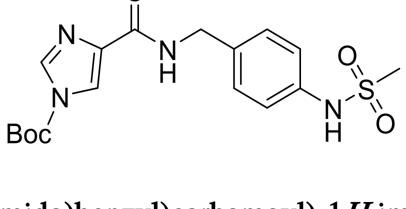

***tert*-Butyl 4-((4-(methylsulfonamido)benzyl)carbamoyl)-1*H*-imidazole-1-carboxylate**

Compound was synthesized using 1-(*tert*-butoxycarbonyl)-1*H*-imidazole-4-carboxylic acid (200 mg, 0.94 mmol) and *N*-(4-(aminomethyl)phenyl)methanesulfonamide hydrochloride (245

mg, 1.04 mmol) according to general procedure J. The product was washed with diethyl ether twice to afford a white solid (265 mg, 71%). ^{1}H NMR (250 MHz, DMSO-d_6): δ 9.64 (s, 1H), 8.75 (t, *J* = 6.3 Hz, 1H), 8.30 (d, *J* = 1.4 Hz, 1H), 7.91 (d, *J* = 1.4 Hz, 1H), 7.27 (d, *J* = 8.4 Hz, 2H), 7.22 – 7.10 (m, 2H), 4.38 (d, *J* = 6.3 Hz, 2H), 2.95 (s, 3H), 1.59 (s, 9H).

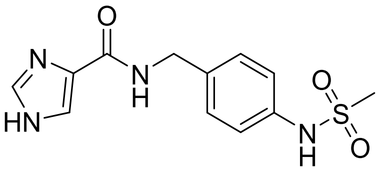

_N_-(4-(Methylsulfonamido)benzyl)-1_H_-imidazole-4-carboxamide (_S26_)

Compound was synthesized using *tert*-butyl 4-((4-(methylsulfonamido)benzyl)carbamoyl)-1*H*-imidazole-1-carboxylate according to General Procedure C. The product was washed with diethyl ether and pentane to yield the title compound as a solid (98 mg, 63%). ^{1}H NMR (400 MHz, DMSO-d_6): δ 9.73 (s, 1H), 9.51 (t, *J* = 5.9 Hz, 1H), 9.07 (s, 1H), 8.27 (s, 1H), 7.30 (d, *J* = 8.4 Hz, 2H), 7.18 (d, *J* = 8.4 Hz, 2H), 4.43 (d, *J* = 5.9 Hz, 2H), 2.95 (s, 3H). HRMS (ESI): m/z [M+H]+ calcd for $C_{12}H_{15}N_4O_3S$ 295.08594, found 295.08575.

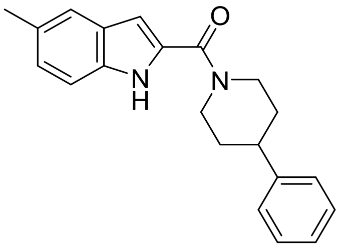

(5-Methyl-1_H_-indol-2-yl)(4-phenylpiperidin-1-yl)methanone (_S27_)

Compound was synthesized using 5-methyl-1*H*-indole-2-carboxylic acid (100 mg, 0.57 mmol) and 4-phenylpiperidine (101 mg, 0.62 mmol) according to general procedure J. The product was purified by FCC (DCM:MeOH 5%) to afford the desired compound as a white solid (140

mg, 77%). ^{1}H NMR (250 MHz, DMSO-d_6): δ 11.43 (s, 1H), 7.41 – 7.13 (m, 7H), 7.01 (dd, J = 8.4, 1.6 Hz, 1H), 6.71 (d, J = 2.1 Hz, 1H), 4.60 (d, J = 13.1 Hz, 2H), 3.23 – 2.75 (m, 3H), 2.37 (s, 3H), 1.87 (d, J = 12.8 Hz, 2H), 1.76 – 1.54 (m, 2H). HRMS (ESI): m/z $[M+H]^+$ calcd for $C_{21}H_{23}N_2O$ 319.18049, found 319.18017.

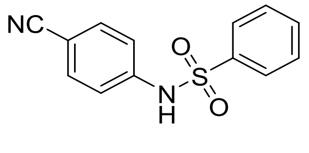

***N*-(4-Cyanophenyl)benzenesulfonamide**

Compound was synthesized using 4-aminobenzonitrile (500 mg, 4.23 mmol) and benzenesulfonyl chloride (748 mg, 4.23 mmol) according to general procedure D. The crude product was washed with diethyl ether to afford the title compound (1.07 g, 98%). ^{1}H NMR (250 MHz, DMSO-d_6): δ 11.06 (s, 1H), 7.90 – 7.79 (m, 2H), 7.76 – 7.51 (m, 5H), 7.34 – 7.19 (m, 2H).

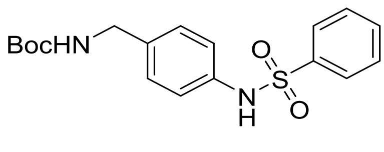

***tert*-Butyl (4-(phenylsulfonamido)benzyl)carbamate**

Compound was synthesized using *N*-(4-cyanophenyl)benzenesulfonamide (1.01 g, 3.92 mmol) according to general procedure B. The product was purified by FCC (DCM: MeOH 0-5%) to afford the desired compound as a white solid (1.35 g, 95%). ^{1}H NMR (250 MHz, $CDCl_3$): δ 7.81 – 7.70 (m, 2H), 7.59 – 7.49 (m, 1H), 7.43 (tt, J = 6.6, 1.8 Hz, 2H), 7.14 (d, J = 8.5 Hz, 2H), 7.06 – 6.94 (m, 2H), 4.81 (t, J = 5.7 Hz, 1H), 4.23 (d, J = 6.1 Hz, 2H), 1.44 (s, 9H).

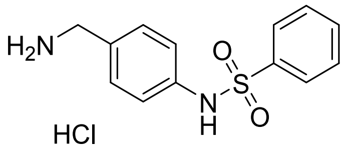

***N*-(4-(Aminomethyl)phenyl)benzenesulfonamide hydrochloride**

Compound was synthesized using *tert*-butyl (4-(phenylsulfonamido)benzyl)carbamate (1.19 g, 3.27 mmol) according to general procedure C. The title compound was washed with diethyl ether to afford the desired compound as a pale yellow solid (941 mg, 96%). ^{1}H NMR (500 MHz, DMSO-d_6): δ 10.53 (s, 1H), 8.31 (s, 3H), 7.82 – 7.77 (m, 2H), 7.63 – 7.58 (m, 1H), 7.58 – 7.51 (m, 2H), 7.37 – 7.31 (m, 2H), 7.15 – 7.09 (m, 2H), 3.87 (q, *J* = 5.7 Hz, 2H).

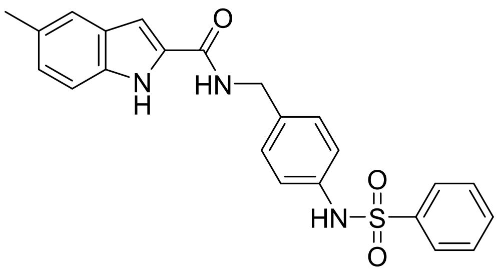

5-Methyl-*N*-(4-(phenylsulfonamido)benzyl)-1*H*-indole-2-carboxamide (*S28*)

Compound was synthesized using 5-methyl-1*H*-indole-2-carboxylic acid (150 mg, 0.85 mmol) and *N*-(4-(aminomethyl)phenyl)benzenesulfonamide hydrochloride (281 mg, 0.94 mmol) according to general procedure J. The product was purified by FCC (DCM:MeOH 5%) to afford the desired compound was a white solid (352 mg, 98%). ^{1}H NMR (250 MHz, DMSO-d_6): δ 11.42 (s, 1H), 8.87 (t, *J* = 6.0 Hz, 1H), 7.83 – 7.45 (m, 6H), 7.36 (s, 1H), 7.30 (d, *J* = 8.4 Hz, 1H), 7.23 – 6.93 (m, 6H), 4.38 (d, *J* = 6.0 Hz, 2H), 2.35 (s, 3H). HRMS (ESI): m/z [M+H]+ calcd for $C_{23}H_{22}N_3O_3S$ 420.13764, found 420.13709.

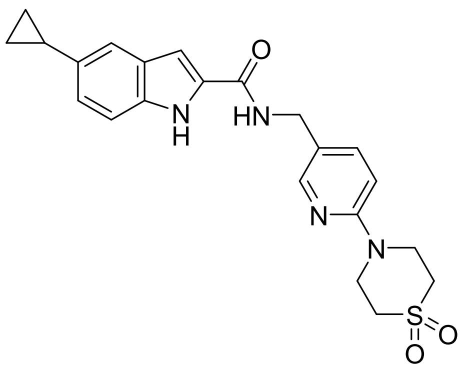

5-Cyclopropyl-*N*-((6-(1,1-dioxidothiomorpholino)pyridin-3-yl)methyl)-1*H*-indole-2-carboxamide (*S29*)

Compound was synthesized using 5-cyclopropyl-1*H*-indole-2-carboxylic acid (86 mg, 0.42 mmol) and 4-(5-(aminomethyl)pyridin-2-yl)thiomorpholine 1,1-dioxide hydrochloride (154 mg, 0.55 mmol) according to general procedure J. The product was washed with diethyl ether to afford a white solid (41 mg, 23%). 1H NMR (250 MHz, DMSO-d_6): δ11.42 (s, 1H), 8.89 (t, *J* = 5.9 Hz, 1H), 8.15 (d, *J* = 2.3 Hz, 1H), 7.60 (dd, *J* = 8.7, 2.4 Hz, 1H), 7.35 – 7.24 (m, 2H), 7.07 – 6.97 (m, 2H), 6.91 (dd, *J* = 8.6, 1.6 Hz, 1H), 4.38 (d, *J* = 5.8 Hz, 2H), 4.07 – 4.00 (m, 4H), 3.12 – 3.03 (m, 4H), 2.05 – 1.87 (m, 1H), 0.97 – 0.82 (m, 2H), 0.70 – 0.57 (m, 2H). HRMS (ESI): m/z $[M+H]^+$ calcd for $C_{22}H_{25}N_4O_3S$ 425.16419, found 425.16348.

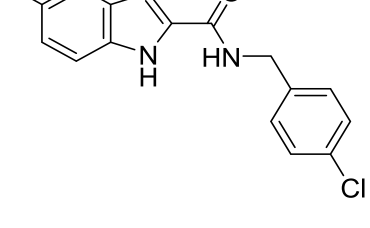

N-(4-Chlorobenzyl)-5-methyl-1*H*-indole-2-carboxamide (*S30*)

Compound was synthesized using 5-methyl-1*H*-indole-2-carboxylic acid (150 mg, 0.85 mmol) and (4-chlorophenyl)methanamine (159 mg, 1.1 mmol) according to general procedure J. The product was purified by FCC (DCM:MeOH 5%) to afford the desired compound was a white solid (198 mg, 77%). ^{1}H NMR (250 MHz, DMSO-d_6): δ 11.46 (s, 1H), 9.00 (s, 1H), 7.45 – 7.26 (m, 6H), 7.11 – 6.96 (m, 2H), 4.48 (d, *J* = 6.0 Hz, 2H), 2.36 (s, 3H). HRMS (ESI): m/z [M+H]$^+$ calcd for $C_{17}H_{16}ClN_2O$ 299.09457, found 299.09446.

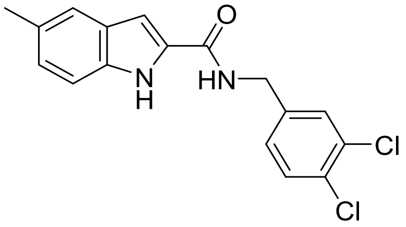

N-(3,4-Dichlorobenzyl)-5-methyl-1H-indole-2-carboxamide (*S31*)

Compound was synthesized using 5-methyl-1*H*-indole-2-carboxylic acid (150 mg, 0.85 mmol) and (3,4-dichlorophenyl)methanamine (196 mg, 1.11 mmol) according to general procedure J. The product was purified by FCC (DCM:MeOH 5%) to afford the desired compound was a white solid (214 mg, 75%). ^{1}H NMR (250 MHz, DMSO-d_6): δ 11.48 (s, 1H), 9.04 (t, *J* = 6.0 Hz, 1H), 7.66 – 7.54 (m, 2H), 7.43 – 7.25 (m, 3H), 7.11 – 6.96 (m, 2H), 4.49 (d, *J* = 6.0 Hz, 2H), 2.36 (s, 3H). HRMS (ESI): m/z [M+H]$^+$ calcd for $C_{17}H_{15}Cl_2N_2O$ 333.05559, found 333.05544.

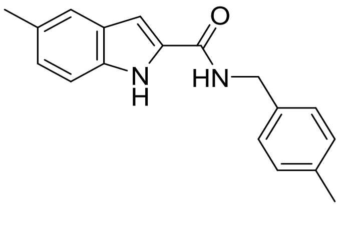

5-Methyl-*N*-(4-methylbenzyl)-1*H*-indole-2-carboxamide (*S32*)

Compound was synthesized using 5-methyl-1*H*-indole-2-carboxylic acid (150 mg, 0.85 mmol) and *p*-tolylmethanamine (114 mg, 0.94 mmol) according to general procedure J. The product was purified by FCC (DCM:MeOH 5%) to afford the desired compound was a white solid (167 mg, 70%). ^{1}H NMR (250 MHz, DMSO-d_6): δ 11.45 (s, 1H), 8.93 (t, J = 6.1 Hz, 1H), 7.38 (s, 1H), 7.32 (d, J = 8.4 Hz, 1H), 7.23 (d, J = 8.1 Hz, 2H), 7.14 (d, J = 7.8 Hz, 2H), 7.07 (d, J = 1.6 Hz, 1H), 7.00 (dd, J = 8.4, 1.6 Hz, 1H), 4.46 (d, J = 6.0 Hz, 2H), 2.36 (s, 3H), 2.27 (s, 3H). HRMS (ESI): m/z $[M+H]^+$ calcd for $C_{18}H_{19}N_2O$ 279.14919, found 279.14910.

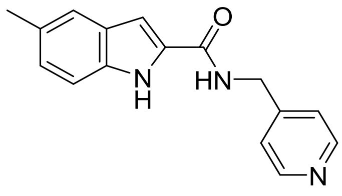

5-Methyl-*N*-(pyridin-4-ylmethyl)-1*H*-indole-2-carboxamide (*S33*)

Compound was synthesized using 5-methyl-1*H*-indole-2-carboxylic acid (150 mg, 0.85 mmol) and pyridin-4-ylmethanamine (111 mg, 1.03 mmol) according to general procedure J. The product was purified by FCC (DCM:MeOH 5%) to afford the desired compound was a white solid (165 mg, 73%). ^{1}H NMR (250 MHz, DMSO-d_6): δ 11.50 (s, 1H), 9.08 (t, J = 6.1 Hz, 1H), 8.58 – 8.46 (m, 2H), 7.40 (s, 1H), 7.36 – 7.29 (m, 3H), 7.11 (d, J = 2.1 Hz, 1H), 7.02 (dd, J = 8.4, 1.7 Hz, 1H), 4.53 (d, J = 6.0 Hz, 2H), 2.37 (s, 3H). HRMS (ESI): m/z $[M+H]^+$ calcd for $C_{16}H_{16}N_3O$ 266.12879, found 266.12919.

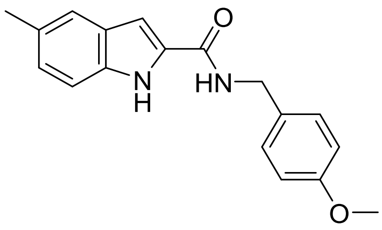

N-(4-Methoxybenzyl)-5-methyl-1H-indole-2-carboxamide (*S34*)

Compound was synthesized using 5-methyl-1H-indole-2-carboxylic acid (150 mg, 0.85 mmol) and (4-methoxyphenyl)methanamine (129 mg, 0.94 mmol) according to general procedure J. The product was purified by FCC (DCM:MeOH 5%) to afford the desired compound was a white solid (148 mg, 59%). ^{1}H NMR (250 MHz, DMSO-d_6): δ 11.44 (s, 1H), 8.90 (t, J = 6.0 Hz, 1H), 7.39 – 7.35 (m, 1H), 7.35 – 7.22 (m, 3H), 7.06 (dd, J = 2.2, 0.9 Hz, 1H), 7.00 (dd, J = 8.4, 1.6 Hz, 1H), 6.93 – 6.85 (m, 2H), 4.43 (d, J = 6.0 Hz, 2H), 3.72 (s, 3H), 2.36 (s, 3H). HRMS (ESI): m/z $[M+H]^+$ calcd for $C_{18}H_{19}N_2O_2$ 295.14410, found 295.14385.

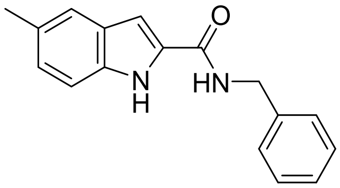

N-Benzyl-5-methyl-1H-indole-2-carboxamide (*S35*)

Compound was synthesized using 5-methyl-1H-indole-2-carboxylic acid (150 mg, 0.85 mmol) and phenylmethanamine (101 mg, 0.94 mmol) according to general procedure J. The product was purified by FCC (DCM:MeOH 5%) to afford the desired compound was a white solid (141 mg, 62%). ^{1}H NMR (250 MHz, DMSO-d_6): δ 11.46 (s, 1H), 8.98 (t, J = 6.1 Hz, 1H), 7.41 – 7.22 (m, 7H), 7.09 (dd, J = 2.2, 0.9 Hz, 1H), 7.01 (dd, J = 8.4, 1.6 Hz, 1H), 4.51 (d, J = 6.0 Hz, 2H), 2.36 (s, 3H). HRMS (ESI): m/z $[M+H]^+$ calcd for $C_{17}H_{17}N_2O$ 265.13354, found 265.13347.

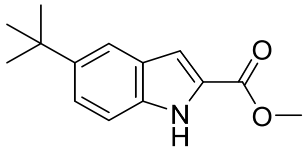

Methyl 5-(*tert*-butyl)-1*H*-indole-2-carboxylate

Compound was synthesized using *tert*-butyl-aniline (149.2 mg, 1 mmol) and methyl pyruvate (204 mg, 2 mmol) according to general procedure E. The crude product was purified by FCC (EtOAc: hexanes 0-30%) to afford the desired solid (61%).^{1}H NMR (250 MHz, $CDCl_3$): δ 8.81 (s, 1H), 7.69 – 7.62 (m, 1H), 7.49 – 7.30 (m, 2H), 7.18 (dd, *J* = 2.0, 1.0 Hz, 1H), 3.94 (s, 3H), 1.38 (s, 9H).

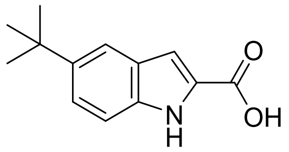

5-(*tert*-Butyl)-1*H*-indole-2-carboxylic acid

Compound was synthesized using methyl 5-(*tert*-butyl)-1*H*-indole-2-carboxylate (60 mg, 0.25 mmol) according to general procedure F to afford the desired product (77%). ^{1}H NMR (250 MHz, DMSO-d_6): δ 12.83 (s, 1H), 11.58 (s, 1H), 7.57 (s, 1H), 7.35 (d, *J* = 1.4 Hz, 2H), 7.04 (d, *J* = 2.1 Hz, 1H), 1.32 (s, 9H).

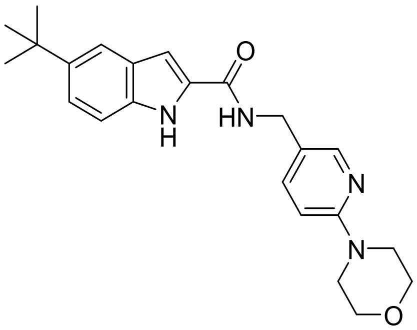

5-(*tert*-Butyl)-*N*-((6-morpholinopyridin-3-yl)methyl)-1*H*-indole-2-carboxamide (*S36*)

Compound was synthesized using 5-(*tert*-butyl)-1*H*-indole-2-carboxylic acid (100 mg, 0.46 mmol) and (6-morpholinopyridin-3-yl)methanamine hydrochloride (126 mg, 0.55 mmol) according to general procedure J. The crude product was washed with ethanol and diethyl ether to afford an off white solid (110 mg, 61%). ^{1}H NMR (500 MHz, DMSO-d_6): δ 11.40 (s, 1H), 8.86 (t, J = 5.9 Hz, 1H), 8.13 (d, J = 2.4 Hz, 1H), 7.58 – 7.51 (m, 2H), 7.34 (d, J = 8.7 Hz, 1H), 7.27 (dd, J = 8.7, 1.9 Hz, 1H), 7.06 (d, J = 2.1 Hz, 1H), 6.82 (d, J = 8.8 Hz, 1H), 4.37 (d, J = 5.9 Hz, 2H), 3.71 – 3.65 (m, 4H), 3.42 – 3.37 (m, 4H), 1.32 (s, 9H). HRMS (ESI): m/z [M+H]$^+$ calcd for $C_{23}H_{29}N_4O_2$ 393.22850, found 393.22802.

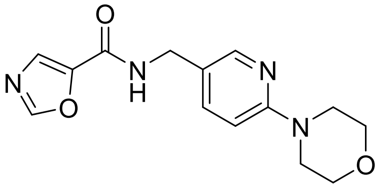

***N*-((6-Morpholinopyridin-3-yl)methyl)oxazole-5-carboxamide (*S37*)**

Compound was synthesized using oxazole-5-carboxylic acid (100 mg, 0.88 mmol) and (6-morpholinopyridin-3-yl)methanamine hydrochloride (223 mg, 0.97 mmol) according to General Procedure J. The crude product was purified by FCC (0–5% DCM:MeOH) to yield the title compound as a solid (174 mg, 68%). ^{1}H NMR (250 MHz, DMSO-d_6): δ 9.07 (t, J = 6.0

Hz, 1H), 8.54 (s, 1H), 8.09 (d, *J* = 2.4 Hz, 1H), 7.77 (s, 1H), 7.52 (dd, *J* = 8.7, 2.5 Hz, 1H), 6.80 (d, *J* = 8.7 Hz, 1H), 4.30 (d, *J* = 5.9 Hz, 2H), 3.73 – 3.61 (m, 4H), 3.42 – 3.36 (m, 4H). HRMS (ESI): m/z $[M+H]^+$ calcd for $C_{14}H_{17}N_4O_3$ 289.12952, found 289.12944.

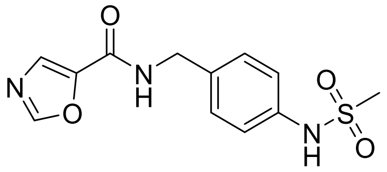

N-(4-(Methylsulfonamido)benzyl)oxazole-5-carboxamide (*S38*)

Compound was synthesized using oxazole-5-carboxylic acid (100 mg, 0.88 mmol) and N-(4-(aminomethyl)phenyl)methanesulfonamide hydrochloride (230 mg, 0.97 mmol) according to General Procedure J. The crude product was purified by FCC (0–5% DCM:MeOH) to yield the title compound as a solid (160 mg, 61%). ^1H NMR (250 MHz, DMSO-d_6): δ 9.66 (s, 1H), 9.14 (t, *J* = 6.0 Hz, 1H), 8.56 (s, 1H), 7.79 (s, 1H), 7.27 (d, *J* = 8.4 Hz, 2H), 7.16 (d, *J* = 8.5 Hz, 2H), 4.38 (d, *J* = 6.1 Hz, 2H), 2.95 (s, 3H). HRMS (ESI): m/z $[M+H]^+$ calcd for $C_{12}H_{14}N_3O_4S$ 296.06995, found 296.06987.

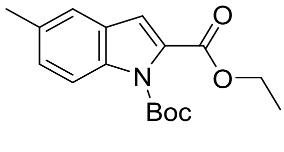

1-(*tert*-Butyl) 2-ethyl 5-methyl-1*H*-indole-1,2-dicarboxylate

To a stirring solution of ethyl 5-methyl-1*H*-indole-2-carboxylate (1.0 g, 4.92 mmol) in DCM (30 mL) was added was added DIPEA (1.3 ml, 7.38 mmol) followed by Boc_2O (1.61 g, 7.38 mmol), DMAP (60 mg, 0.49 mmol) and the stirring was continued for 16 h at rt. After completion of the reaction the crude was concentrated and directly purified by column

chromatography to get desired product (1.42 g, 95%). ^{1}H NMR (250 MHz, $CDCl_3$): δ 7.94 (d, *J* = 8.6 Hz, 1H), 7.37 (s, 1H), 7.22 (dd, *J* = 8.6, 1.7 Hz, 1H), 7.02 (s, 1H), 4.37 (q, *J* = 7.1 Hz, 2H), 2.43 (s, 3H), 1.62 (s, 9H), 1.39 (t, *J* = 7.1 Hz, 3H).

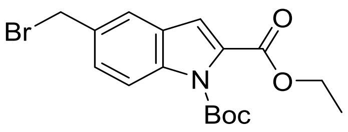

1-(*tert*-Butyl) 2-ethyl 5-(bromomethyl)-1*H*-indole-1,2-dicarboxylate

To a stirring solution of 1-(*tert*-butyl) 2-ethyl 5-methyl-1*H*-indole-1,2-dicarboxylate (500 mg, 1.65 mmol) in CCl_4 (17 mL), was added *N*-bromosuccinimide (293 mg, 1.65 mmol) and benzoyl peroxide (16 mg, 0.06 mmol). After 18h, the solvent was removed under reduced pressure, crude dissolved in EtOAc, washed with water and brine. The product was partially purified by FCC (EtOAc:hexanes 0-5%) however some traces of starting material were still present. The product was used for the next step.

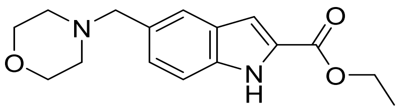

Ethyl 5-(morpholinomethyl)-1*H*-indole-2-carboxylate

To a solution of 1-(*tert*-butyl) 2-ethyl 5-(bromomethyl)-1*H*-indole-1,2-dicarboxylate (573 mg, 1.50 mmol) in MeCN (10 ml), morpholine (303 mg, 3.48 mmol) and potassium carbonate (414 mg, 3.00 mmol) were added and stirred at 80 °C for 48 h. After completion, solvent was removed, crude dissolved in EtOAc, washed with water and brine. The product was purified by FCC (EtOAc:Hexanes 0-80%) to afford the desired product, which during the purification

already lost the boc protecting group (373 mg, 71% over 2 steps). 1H NMR (250 MHz, Acetone-d_6): δ 10.86 (s, 1H), 7.63 – 7.56 (m, 1H), 7.51 – 7.45 (m, 1H), 7.31 (dd, *J* = 8.6, 1.6 Hz, 1H), 7.17 – 7.12 (m, 1H), 4.36 (q, *J* = 7.1 Hz, 2H), 3.64 – 3.57 (m, 4H), 3.54 (s, 2H), 2.45 – 2.34 (m, 4H), 1.36 (t, *J* = 7.1 Hz, 3H).

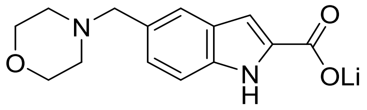

Lithium 5-(morpholinomethyl)-1*H*-indole-2-carboxylate

To a solution of ethyl 5-(morpholinomethyl)-1*H*-indole-2-carboxylate (250 mg, 0.87 mmol) in ethanol and water (2:1 v/v, 5 mL) was added lithium hydroxide (20.8 mg, 0.87 mmol) and stirred at room temperature overnight. After completion, the solvent was removed under reduced pressure to obtain the desired lithium salt (226 mg, 99%). 1H NMR (250 MHz, CD_3OD): δ 7.50 (s, 1H), 7.37 (d, *J* = 8.4 Hz, 1H), 7.16 (dd, *J* = 8.4, 1.6 Hz, 1H), 6.93 (d, *J* = 0.9 Hz, 1H), 3.73 – 3.64 (m, 4H), 3.57 (s, 2H), 2.54 – 2.42 (m, 4H).

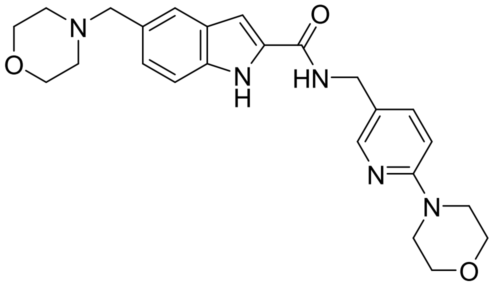

5-(Morpholinomethyl)-*N*-((6-morpholinopyridin-3-yl)methyl)-1*H*-indole-2-carboxamide

(*S39*) Compound was synthesized using lithium 5-(morpholinomethyl)-1*H*-indole-2-carboxylate (110 mg, 0.41 mmol) and (6-morpholinopyridin-3-yl)methanamine hydrochloride

(104 mg, 0.45 mmol) according to general procedure J. The crude product was rinsed with diethyl ether and pentane to afford the desired product as a white solid (81 mg, 45%). ^{1}H NMR (250 MHz, CD_3OD): δ 8.14 (d, *J* = 2.4 Hz, 1H), 7.63 (dd, *J* = 8.8, 2.5 Hz, 1H), 7.54 (s, 1H), 7.41 (d, *J* = 8.5 Hz, 1H), 7.24 (dd, *J* = 8.5, 1.6 Hz, 1H), 7.05 (s, 1H), 6.81 (d, *J* = 8.8 Hz, 1H), 4.47 (s, 2H), 3.82 – 3.74 (m, 4H), 3.73 – 3.64 (m, 4H), 3.59 (s, 2H), 3.48 – 3.39 (m, 4H), 2.55 – 2.41 (m, 4H). HRMS (ESI): m/z $[M+H]^+$ calcd for $C_{24}H_{30}N_5O_3$ 436.23432, found 436.23401.

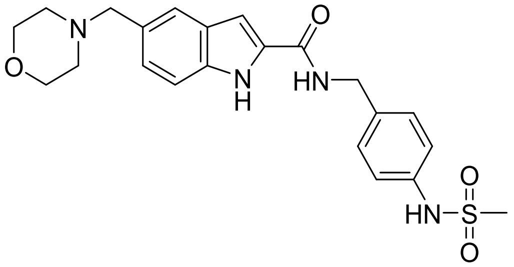

***N*-(4-(Methylsulfonamido)benzyl)-5-(morpholinomethyl)-1*H*-indole-2-carboxamide (*S40*)**

Compound was synthesized using lithium 5-(morpholinomethyl)-1*H*-indole-2-carboxylate (108 mg, 0.41 mmol) and *N*-(4-(aminomethyl)phenyl)methanesulfonamide hydrochloride (108 mg, 0.45 mmol) according to general procedure J. The crude product was rinsed with diethyl ether and pentane to afford the desired product as a white solid (129 mg, 70%). ^{1}H NMR (250 MHz, CD_3OD): δ 7.55 (s, 1H), 7.45 – 7.32 (m, 3H), 7.29 – 7.17 (m, 3H), 7.08 (d, *J* = 0.9 Hz, 1H), 4.56 (s, 2H), 3.73 – 3.64 (m, 4H), 3.59 (s, 2H), 2.92 (s, 3H), 2.55 – 2.39 (m, 4H). HRMS (ESI): m/z $[M+H]^+$ calcd for $C_{22}H_{27}N_4O_4S$ 443.17475, found 443.17453.

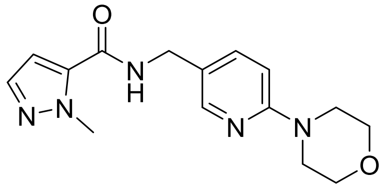

1-Methyl-*N*-((6-morpholinopyridin-3-yl)methyl)-1*H*-pyrazole-5-carboxamide (*S41*)

Compound was synthesized using 1-methyl-1*H*-pyrazole-5-carboxylic acid (90 mg, 0.71 mmol) and (6-morpholinopyridin-3-yl)methanamine hydrochloride (197 mg, 0.85 mmol) according to General Procedure J. The crude product was purified by FCC (0–5% DCM:MeOH) to yield the title compound as a solid (164 mg, 76%). ^{1}H NMR (250 MHz, DMSO-d_6): δ 8.89 (t, *J* = 5.8 Hz, 1H), 8.09 (d, *J* = 2.4 Hz, 1H), 7.52 (dd, *J* = 8.7, 2.5 Hz, 1H), 7.44 (d, *J* = 2.0 Hz, 1H), 6.88 – 6.76 (m, 2H), 4.30 (d, *J* = 5.9 Hz, 2H), 4.05 (s, 3H), 3.74 – 3.63 (m, 4H), 3.45 – 3.34 (m, 4H). HRMS (ESI): m/z [M+H]$^+$ calcd for $C_{15}H_{20}N_5O_2$ 302.16115 found 302.16088.

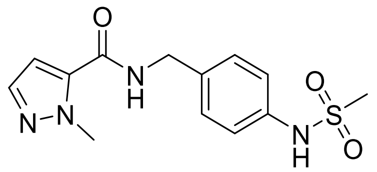

1-Methyl-*N*-(4-(methylsulfonamido)benzyl)-1*H*-pyrazole-5-carboxamide (*S42*)

Compound was synthesized using 1-methyl-1*H*-pyrazole-5-carboxylic acid (90 mg, 0.71 mmol) and *N*-(4-(aminomethyl)phenyl)methanesulfonamide hydrochloride (203 mg, 0.85 mmol) according to General Procedure J. The crude product was purified by FCC (0–5% DCM:MeOH) to yield the title compound as a solid (193 mg, 88%). ^{1}H NMR (250 MHz, DMSO-d_6): δ 9.66 (s, 1H), 8.97 (s, 1H), 7.45 (s, 1H), 7.28 (d, *J* = 8.1 Hz, 2H), 7.17 (d, *J* = 8.1 Hz, 2H), 6.89 (s, 1H), 4.38 (d, *J* = 6.0 Hz, 2H), 4.05 (s, 3H), 2.95 (s, 3H). HRMS (ESI): m/z [M+H]$^+$ calcd for $C_{13}H_{17}N_4O_3S$ 309.10159, found 309.10118.

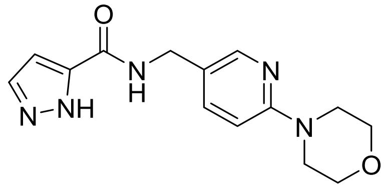

***N*-((6-Morpholinopyridin-3-yl)methyl)-1*H*-pyrazole-3-carboxamide (*S43*)**

Compound was synthesized using 1*H*-pyrazole-5-carboxylic acid (90 mg, 0.80 mmol) and (6-morpholinopyridin-3-yl)methanamine hydrochloride (221 mg, 0.96 mmol) according to General Procedure J. The crude product was purified by FCC (0–5% DCM:MeOH) to yield the title compound as a solid (180 mg, 78%). ^{1}H NMR (250 MHz, DMSO-d_6): δ 13.21 (s, 1H), 8.59 (s, 1H), 8.08 (d, *J* = 2.4 Hz, 1H), 7.80 (s, 1H), 7.53 (dd, *J* = 8.7, 2.5 Hz, 1H), 6.79 (d, *J* = 8.8 Hz, 1H), 6.62 (s, 1H), 4.28 (d, *J* = 6.2 Hz, 2H), 3.73 – 3.62 (m, 4H), 3.42 – 3.34 (m, 4H). HRMS (ESI): m/z $[M+H]^+$ calcd for $C_{14}H_{18}N_5O_2$ 288.14550, found 288.14531.

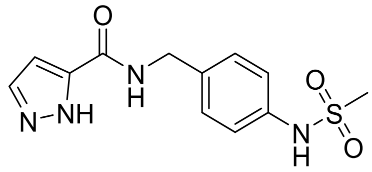

***N*-(4-(Methylsulfonamido)benzyl)-1*H*-pyrazole-3-carboxamide (*S44*)**

Compound was synthesized using 1*H*-pyrazole-5-carboxylic acid (80 mg, 0.71 mmol) and *N*-(4-(aminomethyl)phenyl)methanesulfonamide hydrochloride (203 mg, 0.85 mmol) according to General Procedure J. The crude product was purified by FCC (0–5% DCM:MeOH) to yield the title compound as a solid (132 mg, 63%). ^{1}H NMR (250 MHz, DMSO-d_6): δ 13.23 (s, 1H), 9.63 (s, 1H), 8.63 (t, *J* = 6.3 Hz, 1H), 7.88 – 7.76 (m, 1H), 7.27 (d, *J* = 8.5 Hz, 2H), 7.14 (d, *J* = 8.4 Hz, 2H), 6.64 (t, *J* = 2.1 Hz, 1H), 4.37 (d, *J* = 6.4 Hz, 2H), 2.94 (s, 3H). HRMS (ESI): m/z $[M+H]^+$ calcd for $C_{12}H_{15}N_4O_3S$ 295.08594, found 295.08571.

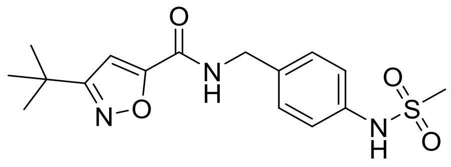

3-(*tert*-Butyl)-*N*-(4-(methylsulfonamido)benzyl)isoxazole-5-carboxamide (*S45*)

Compound was synthesized using 3-(*tert*-butyl)isoxazole-5-carboxylic acid (109 mg, 0.64 mmol) and *N*-(4-(aminomethyl)phenyl)methanesulfonamide hydrochloride (198 mg, 0.83 mmol) according to General Procedure J. The crude product was purified by FCC (0–5% DCM:MeOH) to yield the title compound as a white solid (20 mg, 9%).^{1}H NMR (500 MHz, DMSO-d_6) δ: 9.67 (s, 1H), 9.37 (t, *J* = 6.1 Hz, 1H), 7.28 (d, 1H), 7.17 (d, *J* = 8.5 Hz, 1H), 7.13 (s, 1H), 4.39 (d, *J* = 6.0 Hz, 2H), 2.95 (s, 3H), 1.29 (s, 9H). HRMS (ESI): m/z $[M+H]^+$ calcd for $C_{16}H_{22}N_3O_4S$ 352.13255, found 352.13222.

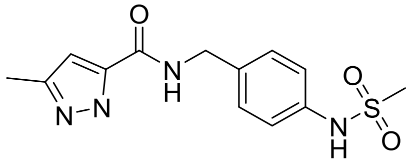

5-Methyl-*N*-(4-(methylsulfonamido)benzyl)-1*H*-pyrazole-3-carboxamide (*S46*)

Compound was synthesized using 5-methyl-1*H*-pyrazole-3-carboxylic acid (80 mg, 0.63 mmol) and *N*-(4-(aminomethyl)phenyl)methanesulfonamide hydrochloride (195 mg, 0.82 mmol) according to General Procedure J. The crude product was purified by FCC (0–5% DCM:MeOH) to yield the title compound as a white solid (52 mg, 27%). ^{1}H NMR (250 MHz, DMSO-d_6) δ 12.89 (s, 1H), 9.62 (s, 1H), 8.52 (t, *J* = 5.7 Hz, 1H), 7.25 (d, *J* = 8.3 Hz, 2H), 7.14 (d, *J* = 8.2

Hz, 2H), 6.36 (s, 1H), 4.34 (d, J = 6.4 Hz, 2H), 2.94 (s, 3H), 2.25 (s, 3H). HRMS (ESI): m/z $[M+H]^+$ calcd for $C_{13}H_{17}N_4O_3S$ 309.10159, found 309.10104.

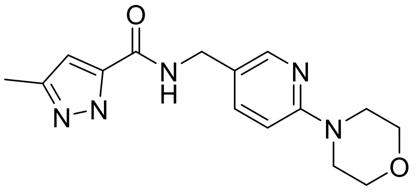

5-Methyl-*N*-((6-morpholinopyridin-3-yl)methyl)-1*H*-pyrazole-3-carboxamide (*S47*)

Compound was synthesized using 5-methyl-1*H*-pyrazole-3-carboxylic acid (80 mg, 0.63 mmol) and (6-morpholinopyridin-3-yl)methanamine hydrochloride (175 mg, 0.76 mmol) according to General Procedure J. The crude product was purified by FCC (0–5% DCM:MeOH) to yield the title compound as a white solid (127 mg, 66%). ^1H NMR (250 MHz, DMSO-d_6) δ 12.88 (s, 1H), 8.48 (t, J = 6.3 Hz, 1H), 8.07 (d, J = 2.4 Hz, 1H), 7.52 (dd, J = 8.7, 2.5 Hz, 1H), 6.78 (d, J = 8.7 Hz, 1H), 6.35 (s, 1H), 4.26 (d, J = 6.3 Hz, 2H), 3.75 – 3.58 (m, 4H), 3.45 – 3.34 (m, 4H), 2.24 (s, 3H). HRMS (ESI): m/z $[M+H]^+$ calcd for $C_{15}H_{20}N_5O_2$ 302.16115, found 302.16090.

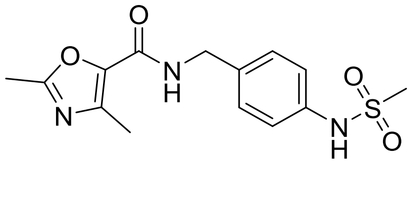

2,4-Dimethyl-*N*-(4-(methylsulfonamido)benzyl)oxazole-5-carboxamide (*S48*)

Compound was synthesized using 2,4-dimethyloxazole-5-carboxylic acid (100 mg, 0.70 mmol) and *N*-(4-(aminomethyl)phenyl)methanesulfonamide hydrochloride (185 mg, 0.77 mmol) according to General Procedure J. The crude product was purified by FCC (0–5% DCM:MeOH)

to yield the title compound as a white solid (191 mg, 83%). ^{1}H NMR (250 MHz, DMSO-d_6): δ 9.64 (s, 1H), 8.78 (t, J = 6.1 Hz, 1H), 7.32 – 7.20 (m, 2H), 7.20 – 7.10 (m, 2H), 4.33 (d, J = 6.2 Hz, 2H), 2.94 (s, 3H), 2.41 (s, 3H), 2.30 (s, 3H). HRMS (ESI): m/z [M+H]$^+$ calcd for $C_{14}H_{18}N_3O_4S$ 324.10125, found 324.10086.

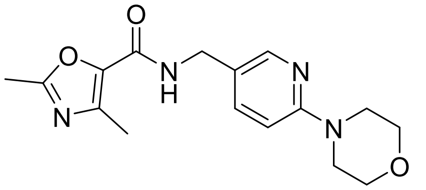

2,4-Dimethyl-*N*-((6-morpholinopyridin-3-yl)methyl)oxazole-5-carboxamide (*S49*)

Compound was synthesized using 2,4-dimethyloxazole-5-carboxylic acid (100 mg, 0.70 mmol) and (6-morpholinopyridin-3-yl)methanamine hydrochloride (179 mg, 0.77 mmol) according to General Procedure J. The crude product was purified by FCC (0–5% DCM:MeOH) to yield the title compound as a white solid (187 mg, 83%). ^{1}H NMR (250 MHz, DMSO-d_6): δ 8.72 (t, J = 6.0 Hz, 1H), 8.07 (d, J = 2.3 Hz, 1H), 7.51 (dd, J = 8.7, 2.4 Hz, 1H), 6.79 (d, J = 8.7 Hz, 1H), 4.24 (d, J = 6.1 Hz, 2H), 3.73 – 3.63 (m, 4H), 3.43 – 3.36 (m, 4H), 2.40 (s, 3H), 2.30 (s, 3H). HRMS (ESI): m/z [M+H]$^+$ calcd for $C_{16}H_{21}N_4O_3$ 317.16082, found 317.16049.

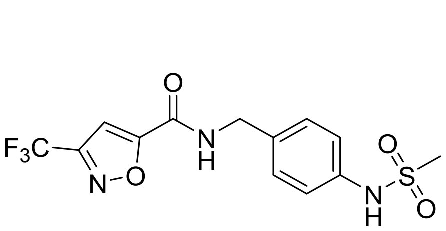

***N*-(4-(Methylsulfonamido)benzyl)-3-(trifluoromethyl)isoxazole-5-carboxamide (*S50*)**

Compound was synthesized using 3-(trifluoromethyl)isoxazole-5-carboxylic acid (63 mg, 0.34 mmol) and *N*-(4-(aminomethyl)phenyl)methanesulfonamide hydrochloride (90.6 mg, 0.38

mmol) according to General Procedure J. The crude product was purified by FCC (0–5% DCM:MeOH) to yield the title compound as a white solid (79 mg, 62%). ^{1}H NMR (500 MHz, DMSO-d_6): δ 9.70 (t, J = 6.0 Hz, 1H), 7.72 (s, 1H), 7.36 – 7.25 (m, 2H), 7.22 – 7.12 (m, 2H), 4.44 (s, 2H), 2.96 (s, 3H). HRMS (ESI): m/z [M+H]$^+$ calcd for $C_{13}H_{13}F_3N_3O_4S$ 364.05734, found 364.05692.

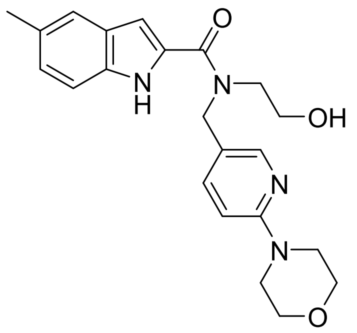

***N*-(2-Hydroxyethyl)-5-methyl-*N*-((6-morpholinopyridin-3-yl)methyl)-1*H*-indole-2-carboxamide (*S51*)**

Solid *para*-toluenosulfonic acid, monohydrate (158 mg, 0.83 mmol) was added to a solution of 5-methyl-*N*-((6-morpholinopyridin-3-yl)methyl)-*N*-(2-((tetrahydro-2*H*-pyran-2-yl)oxy)ethyl)-1*H*-indole-2-carboxamide (362 mg, 0.75 mmol) in methanol. The clear solution was stirred at room temperature for two hours and then quenched by the addition of solid sodium acetate (74.5 mg, 0.90 mmol). After completion, methanol was evaporated in vacuum and the solid evaporation residue was partitioned between EtOAc and an aqueous sodium carbonate solution. After phase separation, the organic phase was washed with water, brine, dried over magnesium sulfate, filtrated, and concentrated in vacuum. The title compound was purified by FCC chloroform/methanol 40:1 as eluent to afford white solid (207 mg, 69%). ^{1}H NMR (400 MHz, Acetone-d_6): δ 10.74 (br. s., 1H), 8.19 (s, 1H), 7.59 (d, J = 8.3 Hz, 1H), 7.33 - 7.48 (m, 2H),

7.06 (dd, *J* = 1.5, 8.3 Hz, 1H), 6.71 - 6.95 (m, 2H), 4.85 (br. s., 2H), 3.89 (br. s., 2H), 3.65 - 3.80 (m, 6H), 3.42 - 3.52 (m, 4H), 2.38 (s, 3H). HRMS (ESI): m/z $[M+H]^+$ calcd for $C_{22}H_{27}N_4O_3$ 395.20777, found 395.20753.

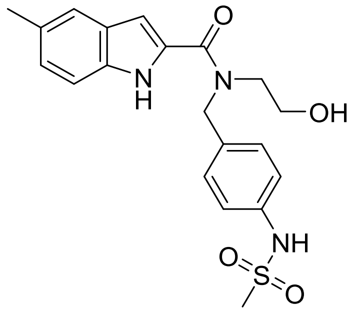

***N*-(2-Hydroxyethyl)-5-methyl-*N*-(4-(methylsulfonamido)benzyl)-1*H*-indole-2-carboxamide (*S52*)**

Compound was synthesized using 5-methyl-1*H*-indole-2-carboxylic acid (42mg , 0.24 mmol) and *N*-(4-(((2-hydroxyethyl)amino)methyl)phenyl)methanesulfonamide hydrochloride (67.3 mg, 0.24 mmol) according to General Procedure J. The crude product was purified by FCC (0–5% $CHCl_3$:MeOH) to yield the title compound as an off-white solid (53 mg, 55%). ^{1}H NMR (400 MHz, DMSO-d_6): δ 11.22 (br. s., 1H), 9.46 (s, 1H), 7.32 - 7.37 (m, 2H), 7.25 - 7.31 (m, 2H), 7.19 - 7.25 (m, 2H), 7.01 (dd, *J* = 1.2, 8.56 Hz, 1H), 6.70 (s, 1H), 4.84 (s, 2H), 3.57 - 3.71 (m, 4H), 2.97 (s, 3H), 2.36 (s, 3H) . HRMS (ESI): m/z $[M+H]^+$ calcd for $C_{20}H_{24}N_3O_4S$ 402.14820, found 402.14794.

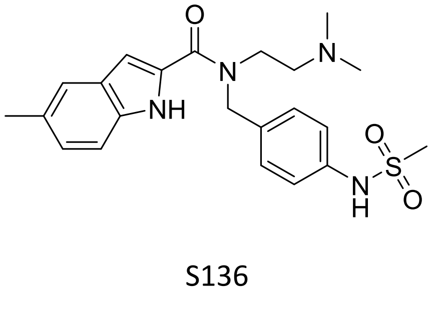

***N*-(2-(Dimethylamino)ethyl)-5-methyl-*N*-(4-(methylsulfonamido)benzyl)-1*H*-indole-2-carboxamide (*S53*)**

A solution of *N*-(4-aminobenzyl)-*N*-(2-(dimethylamino)ethyl)-5-methyl-1*H*-indole-2-carboxamide (100 mg, 0.28 mmol) and pyridinium *p*-toluenesulfonate (71.7 mg, 0.28 mmol) in dry DCM (10 mL) was cooled down to 0 °C. Neat methanesulfonyl chloride (32.7 mg, 0.28 mmol) was added portion wise within ca. 30 minutes. The mixture was stirred for 2 hours and then quenched with a few drops of water. After completion, the reaction mixture was concentrated in vacuum. The evaporation residue was partitioned between water and EtOAc and the pH was set to 10 with solid sodium carbonate. The organic layer was washed with brine, dried over magnesium sulfate, filtrated, and concentrated to dryness in vacuum. Product was washed with diethyl ether and chloroform to afford the desired solid (104 mg, 85%). ^{1}H NMR (500 MHz, DMSO-d_6): δ 11.59 (br. s., 1H), 9.73 (br. s., 1H), 7.10 - 7.46 (m, 6H), 7.01 (d, J = 8.33 Hz, 1H), 6.34 - 6.92 (m, 1H), 4.80 (br. s., 2H), 3.57 (br. s., 2H), 2.98 (s, 3H), 2.34 (s, 3H), 2.06 - 2.24 (m, 6H). HRMS (ESI): m/z [M+H]$^+$ calcd for $C_{22}H_{29}N_4O_3S$ 429.19549, found 429.19528.

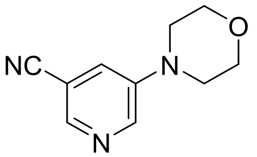

5-Morpholinonicotinonitrile

DMSO (3 mL) taken in a sealed tube and degassed for 5 min. To it, 5-bromonicotinonitrile (250 mg, 1.366 mmol), CuI (52.034 mg, 0.273 mmol), proline (62.91 mg, 0.55 mmol), morpholine (237.98 mg, 2.73 mmol) and K_2CO_3 (566.39 mg, 4.09 mmol) were added sequentially. Reaction

mixture was stirred for 15 h at 120 °C. LCMS showed desired mass. Reaction was quenched by water and extracted by ethyl acetate, dried over Na_2SO_4. Concentrated under reduced pressure to get white crude which was purified by combi flash column to get desired product (240 mg, 93%). LC-MS (*m/z*): 190.0 $[M + H]^+$.

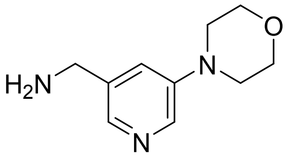

(5-Morpholinopyridin-3-yl)methanamine

A mixture of 5-morpholinonicotinonitrile (50 mg, 0.26 mmol), Raney-Nickel (100 mg) and ammonium hydroxide (0.5 mL) in methanol (5 mL) was stirred under 1 atmosphere of hydrogen (balloon pressure) at room temperature for 1 h. The catalyst was removed by filtration through a pad of celite and washed with several portions of methanol. The filtrate and washings were combined and concentrated under vacuum to get desired product (30 mg, crude). LC-MS (*m/z*): 194.1 $[M + H]^+$.

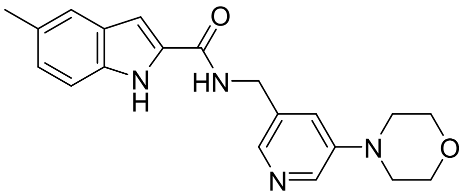

5-Methyl-*N*-((5-morpholinopyridin-3-yl)methyl)-1*H*-indole-2-carboxamide (*S54*)

To a stirring solution of 5-methyl-1*H*-indole-2-carboxylic acid (0.25 g, 1.32 mmol) in DMF (10 ml) was added DIPEA (0.46 ml, 2.6 mmol) and HATU (0.6 g, 1.5 mmol) and stirring was continued for 10 min at rt. Then 5-morpholinonicotinonitrile (278 mg, 1.58 mmol) was added

to it and the reaction was continued for 16 h. LCMS showed the formation of product. Solvent was evaporated and the reaction mixture was extracted with EtOAc and washed by water. The organic layer was concentrated and purified by RP Prep HPLC to get desired product (60 mg, 13%). 1H NMR (400 MHz, DMSO-d_6) δ 11.45 (s, 1H), 8.95 (t, *J* = 5.8 Hz, 1H), 8.20 (d, *J* = 2.6 Hz, 1H), 8.02 (s, 1H), 7.38 (s, 1H), 7.30 (d, *J* = 8.4 Hz, 1H), 7.28 (s, 1H), 7.05 (s, 1H), 7.00 (d, *J* = 8.4 Hz, 1H), 4.47 (d, *J* = 5.9 Hz, 1H), 3.73 (d, *J* = 4.6 Hz, 4H), 3.14 (t, *J* = 4.8 Hz, 4H), 2.37 (s, 3H). LC-MS (*m/z*): 351.3[M + H]$^+$.

NMR spectra of target compounds (1-93)

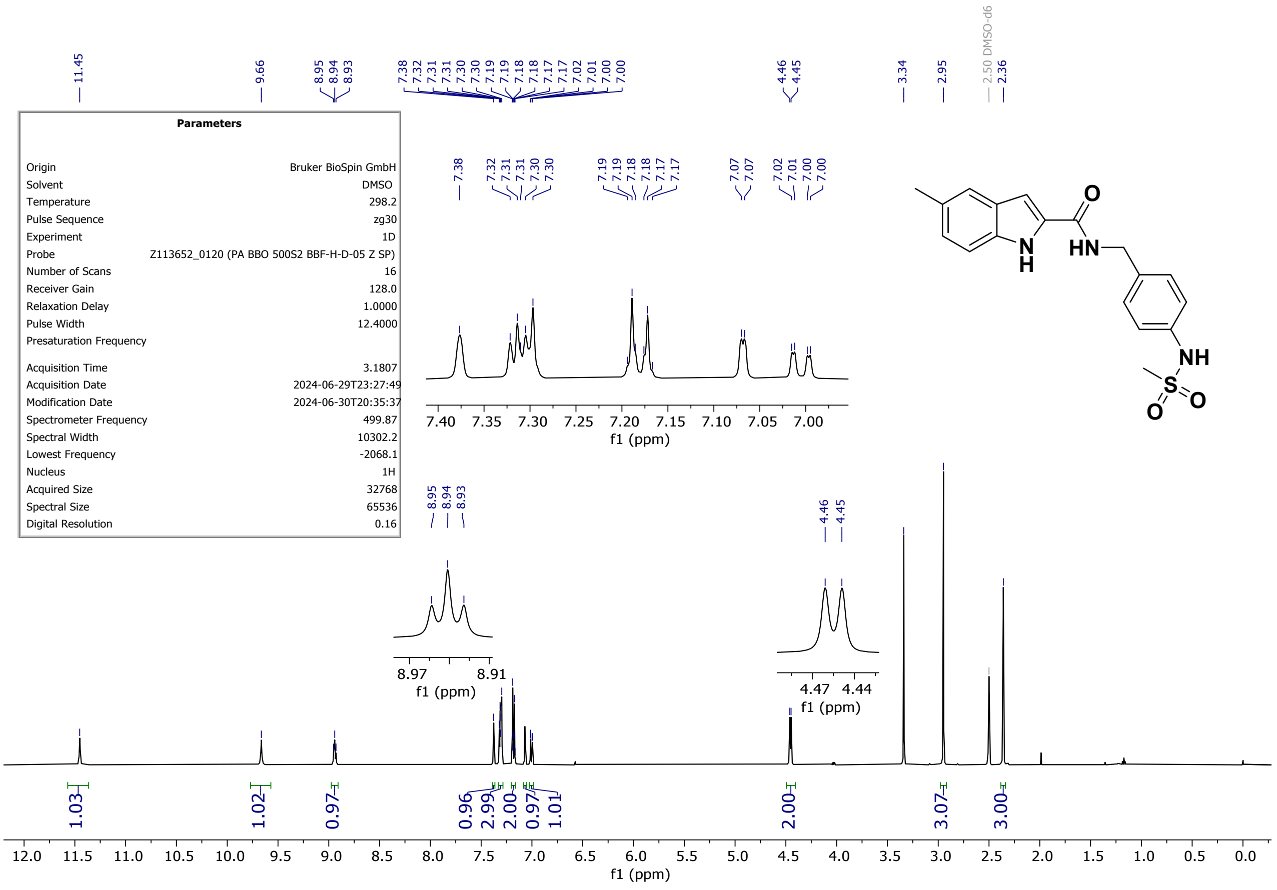

Figure S10: 5-methyl-N-(4-(methylsulfonamido)benzyl)-1H-indole-2-carboxamide (1)

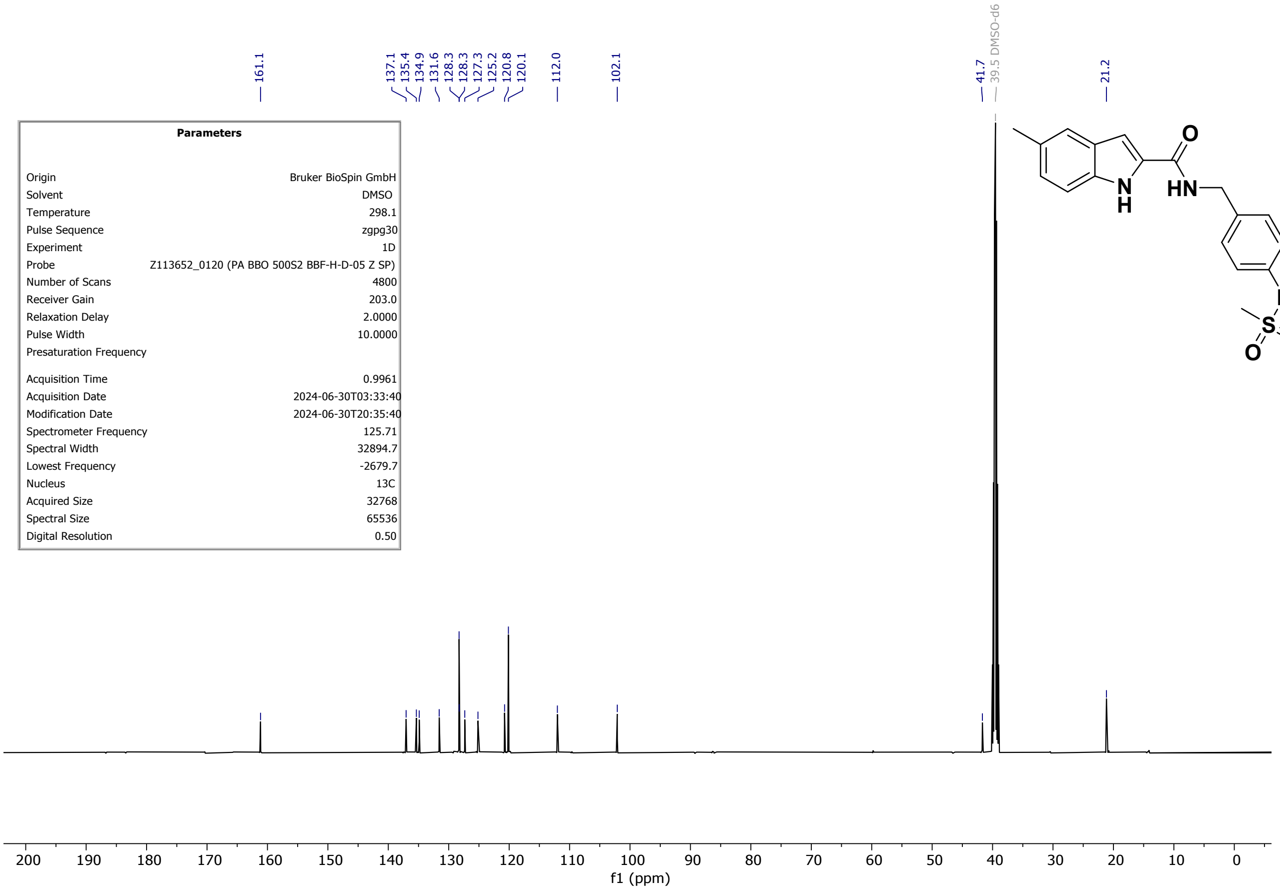

Figure S11: 5-methyl-N-(4-(methylsulfonamido)benzyl)-1H-indole-2-carboxamide (1)

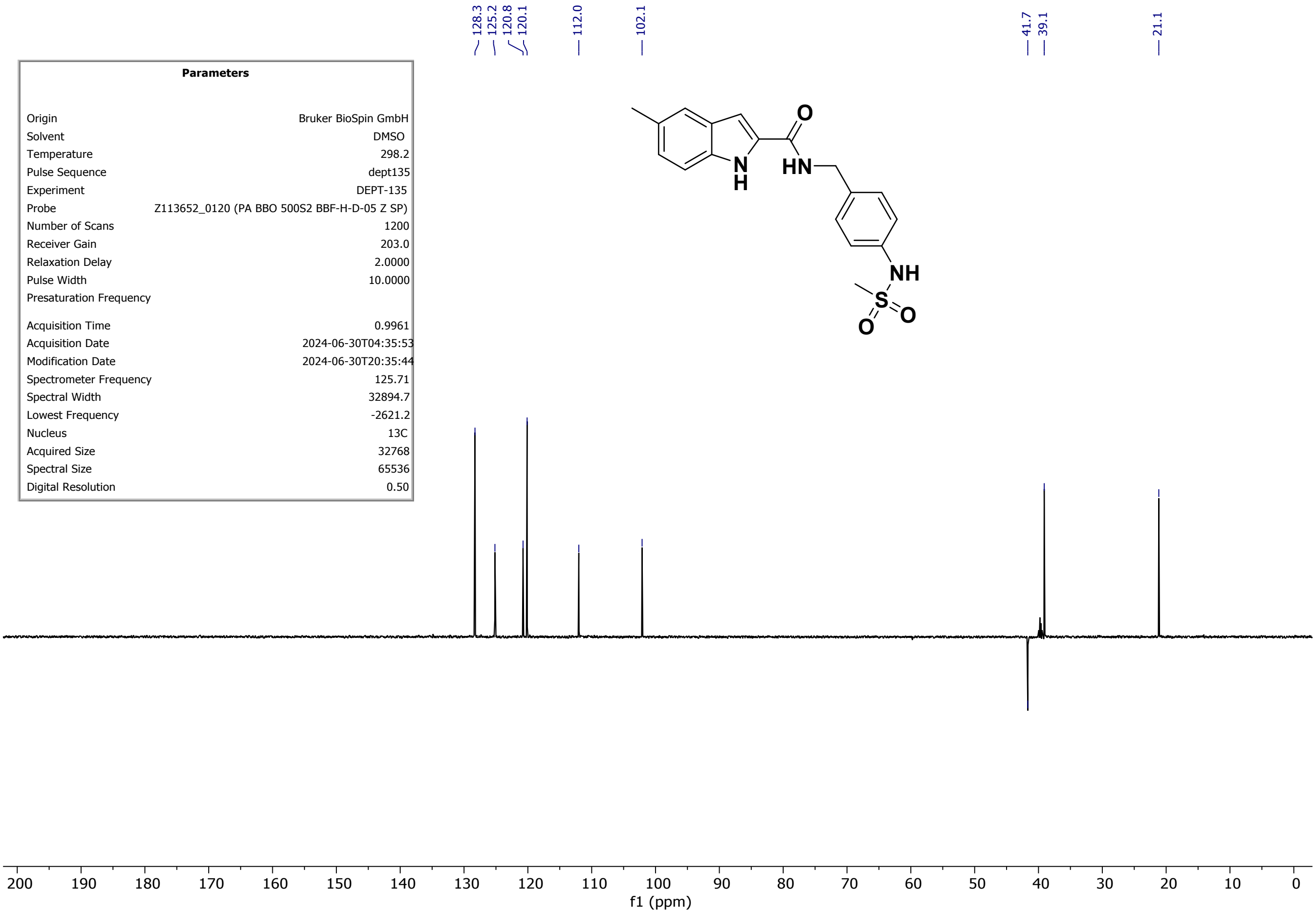

Figure S12: 5-methyl-N-(4-(methylsulfonamido)benzyl)-1H-indole-2-carboxamide (1)

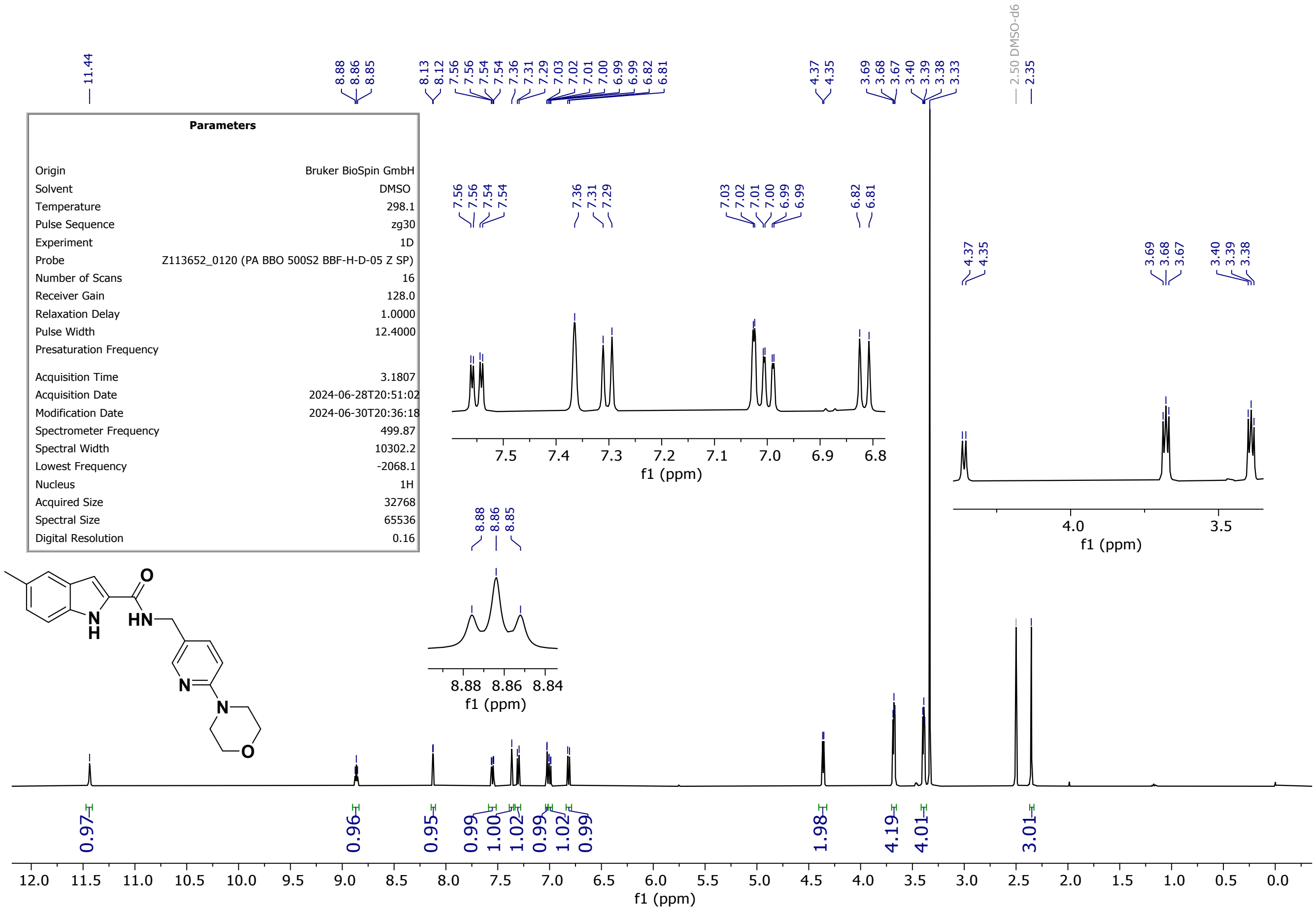

Figure S13: 5-methyl-N-((6-morpholinopyridin-3-yl)methyl)-1H-indole-2-carboxamide (2)

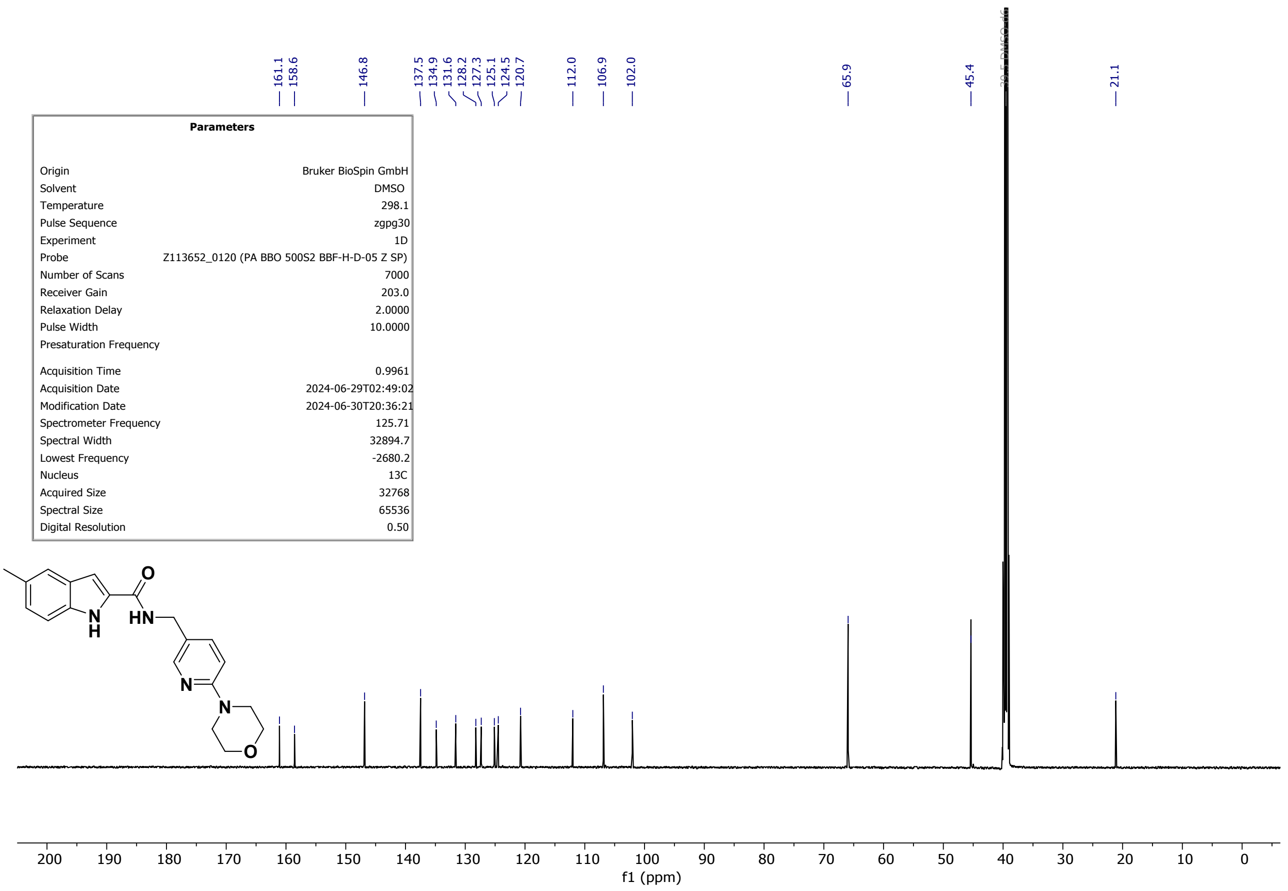

Figure S14: 5-methyl-N-((6-morpholinopyridin-3-yl)methyl)-1H-indole-2-carboxamide (2)

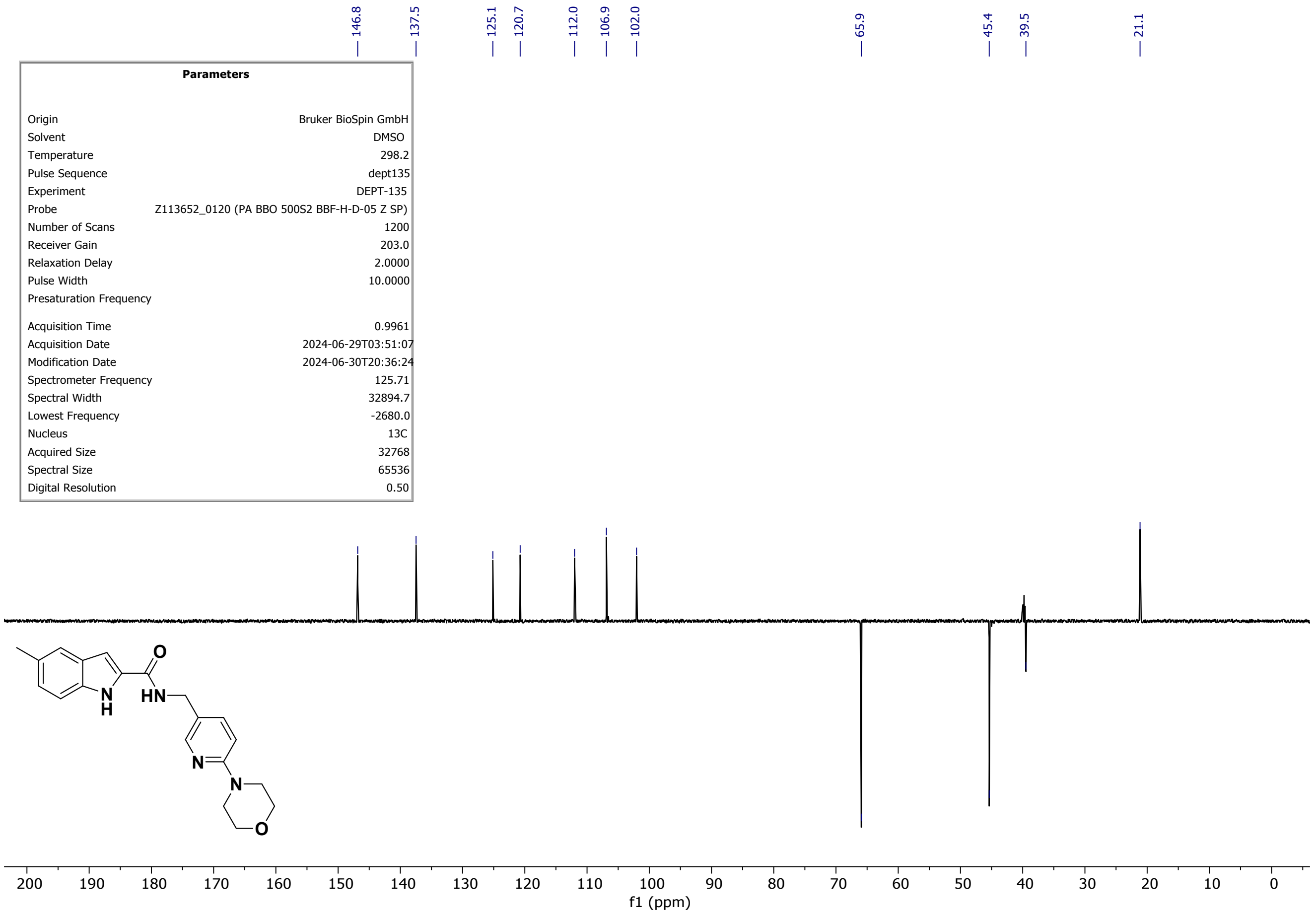

Figure S15: 5-methyl-N-((6-morpholinopyridin-3-yl)methyl)-1H-indole-2-carboxamide (2)

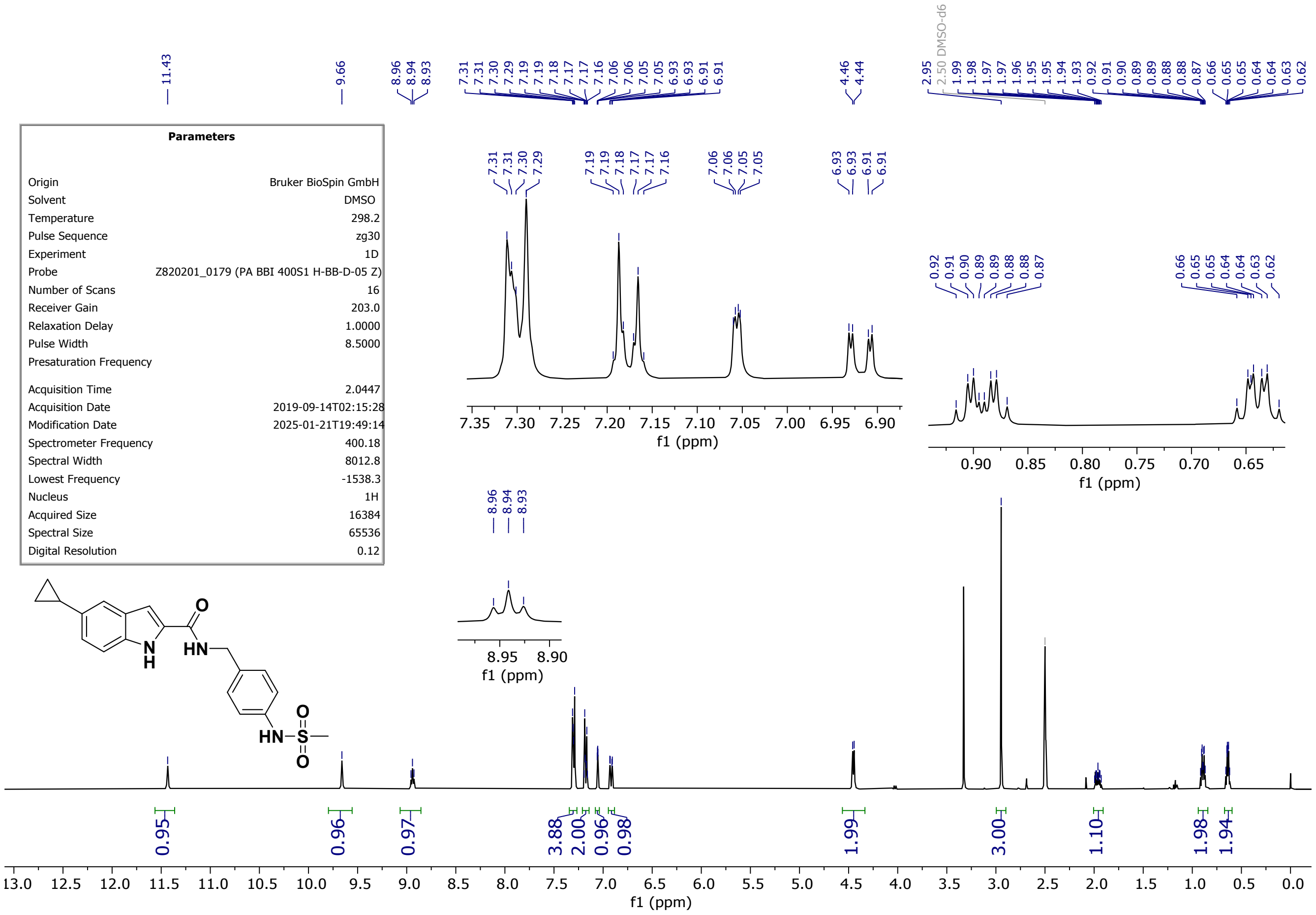

Figure S16: 5-cyclopropyl-N-(4-(methylsulfonamido)benzyl)-1H-indole-2-carboxamide (3)

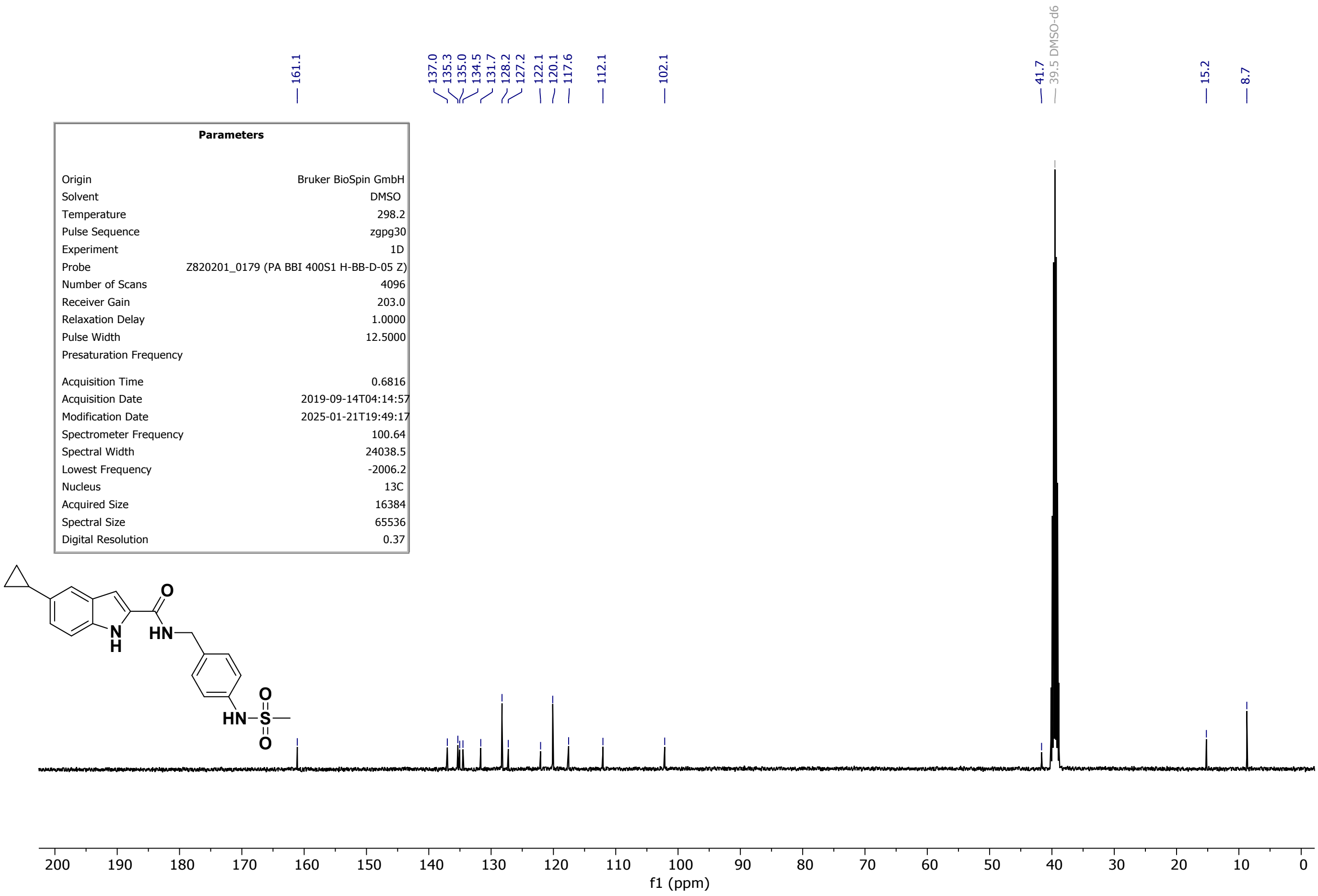

Figure S17: 5-cyclopropyl-N-(4-(methylsulfonamido)benzyl)-1H-indole-2-carboxamide (3).

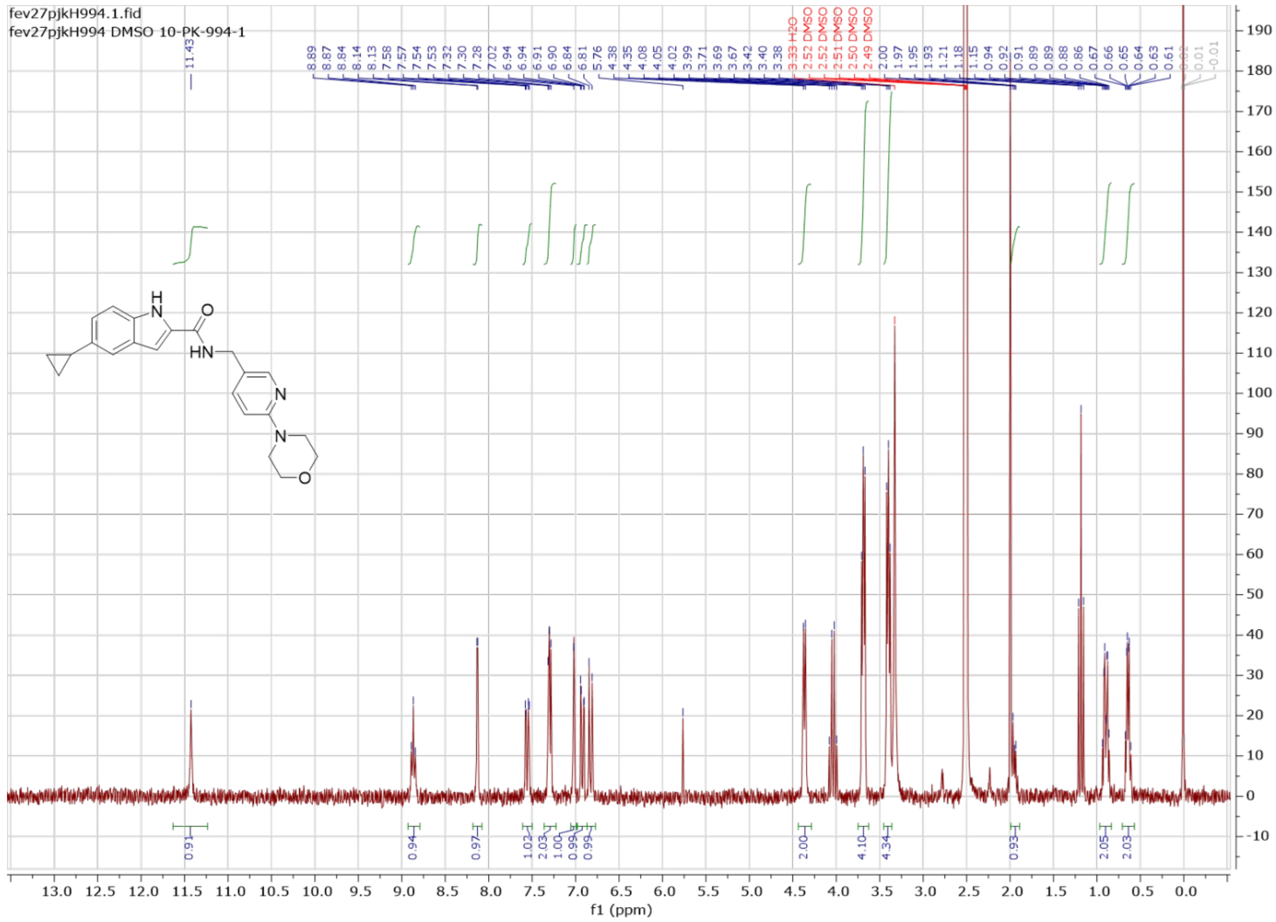

Figure S18: 5-cyclopropyl-N-((6-morpholinopyridin-3-yl)methyl)-1H-indole-2-carboxamide (4)

This report was created by ACD/NMR Processor Academic Edition. For more information go to www.acdlabs.com/nmrproc/

**Acquisition Time (sec)**	1.5903	**Comment**	Dessoy - MAD 1961 - DMSO - Avance 500 MHz - out02madH1 - 1H			**Date**	02 Oct 2019 15:05:44
**File Name**	\\nmrsparc.iqm.unicamp.br\espectros\avance500\2019\out19\Sala\Luiz Carlos\out02madH1_001001r					**Frequency (MHz)**	499.87
**Nucleus**	1H	**Number of Transients**	16	**Original Points Count**	16384	**Points Count**	65536
**Pulse Sequence**	zg30	**Solvent**	DMSO-d6	**Spectrum Offset (Hz)**	3082.7227	**Sweep Width (Hz)**	10302.20
**Temperature (degree C)**	25.152						

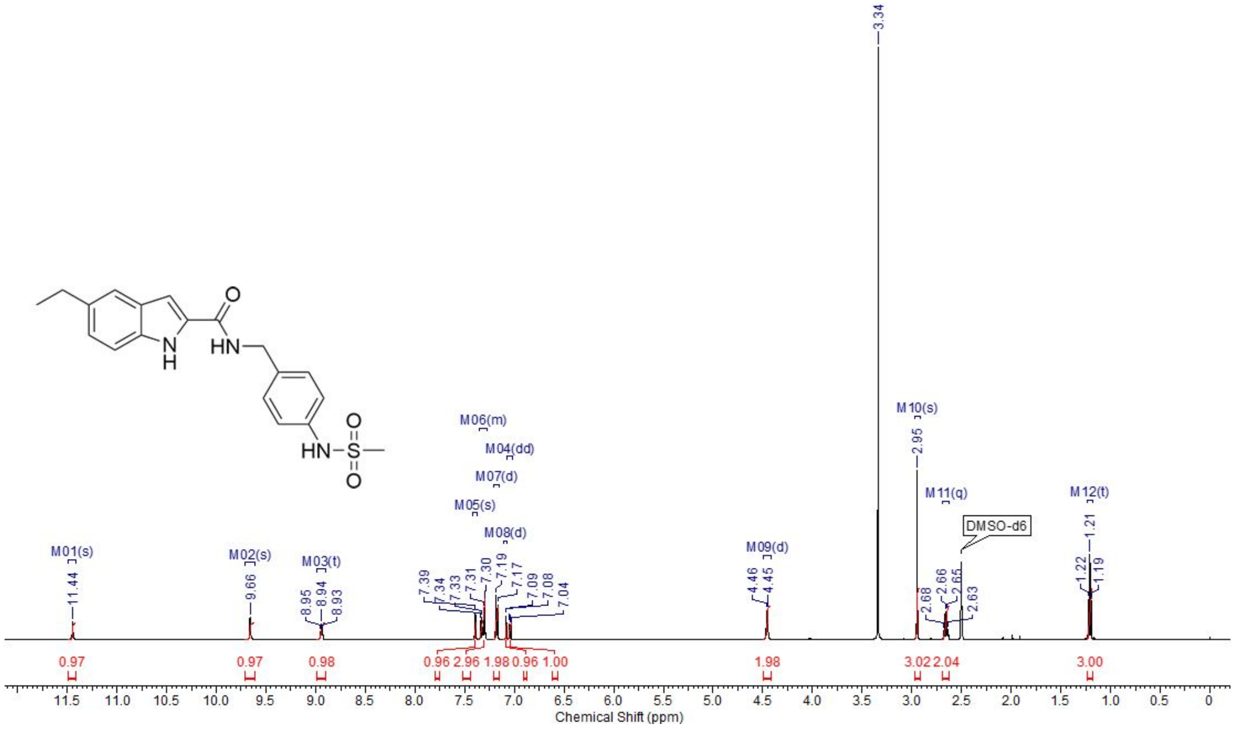

Figure S19: 5-ethyl-N-(4-(methylsulfonamido)benzyl)-1H-indole-2-carboxamide (5)

This report was created by ACD/NMR Processor Academic Edition. For more information go to www.acdlabs.com/nmrproc/

Acquisition Time (sec)	1.5903	***Comment***	Dessoy - MAD 1931 - DMSO - AV 500 MHz - ago05madH1			***Date***	05 Aug 2019 10:22:46
File Name	\\nmrsparc.iqm.unicamp.br\espectros\avance500\2019\ago19\Sala\Luiz Carlos\ago05madH1_001001r					***Frequency (MHz)***	499.87
Nucleus	1H	***Number of Transients***	16	***Original Points Count***	16384	***Points Count***	65536
Pulse Sequence	zg30	***Solvent***	DMSO-d6	***Spectrum Offset (Hz)***	3082.5654	***Sweep Width (Hz)***	10302.20
Temperature (degree C)	25.148						

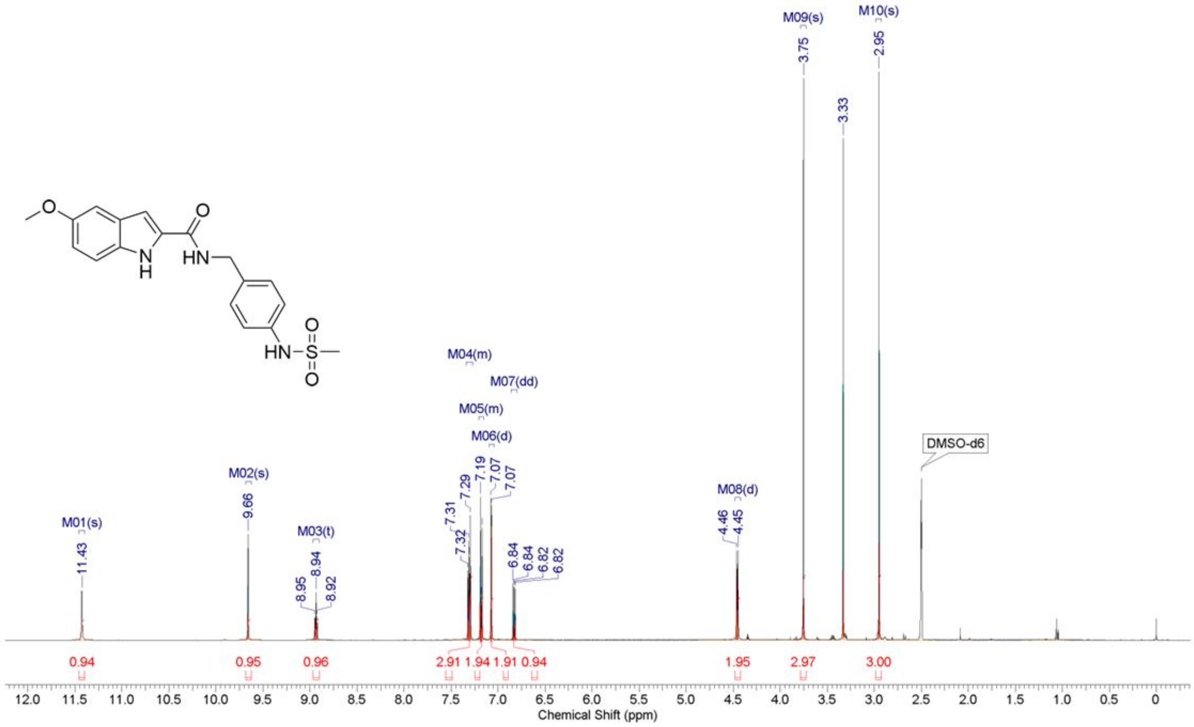

Figure S20: 5-methoxy-N-(4-(methylsulfonamido)benzyl)-1H-indole-2-carboxamide (6)

This report was created by ACD/NMR Processor Academic Edition. For more information go to www.acdlabs.com/nmrproc/

Acquisition Time (sec)	1.5903	***Comment***	Dessoy - MAD1930 - DMSO - Av 500 MHz - ago02madH1			***Date***	02 Aug 2019 14:19:30
File Name	\\nmrsparc.iqm.unicamp.br\espectros\avance500\2019\ago19\Sala\Luiz Carlos\ago02madH1_001001r					***Frequency (MHz)***	499.87
Nucleus	1H	***Number of Transients***	16	***Original Points Count***	16384	***Points Count***	65536
Pulse Sequence	zg30	***Solvent***	DMSO-d6	***Spectrum Offset (Hz)***	3082.7227	***Sweep Width (Hz)***	10302.20
Temperature (degree C)	25.147						

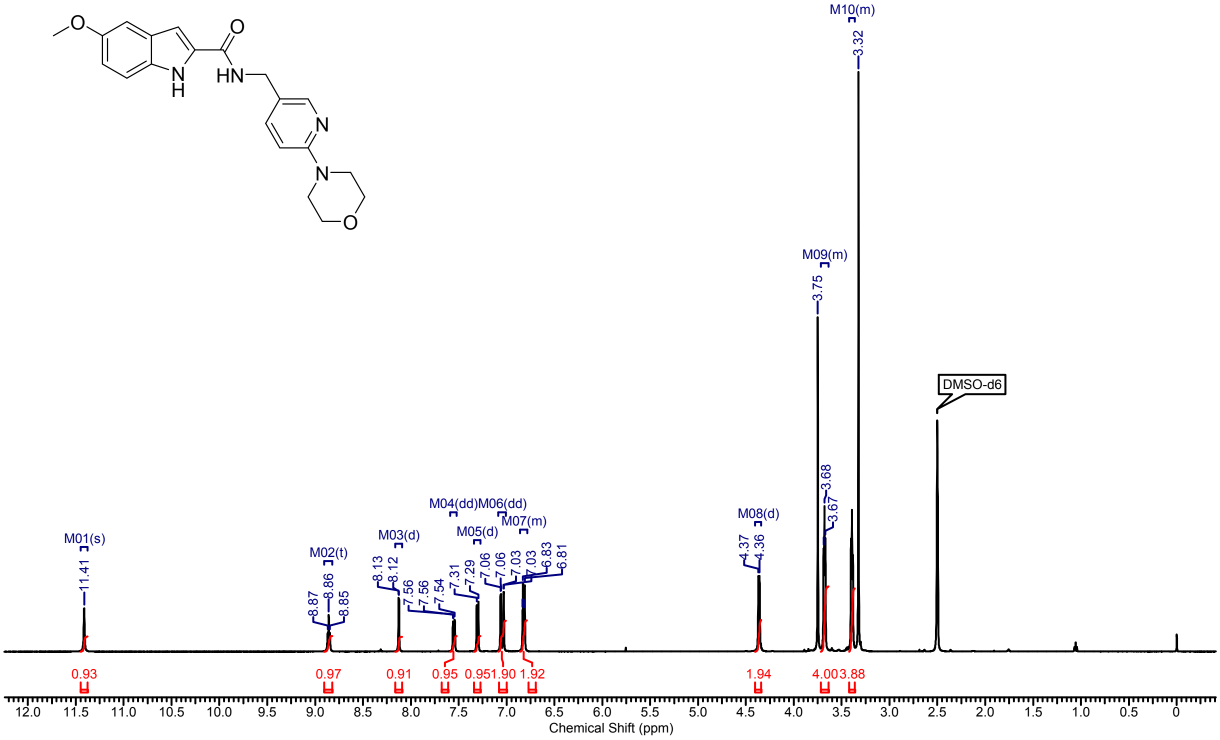

Figure S21: 5-methoxy-N-((6-morpholinopyridin-3-yl)methyl)-1H-indole-2-carboxamide (7)

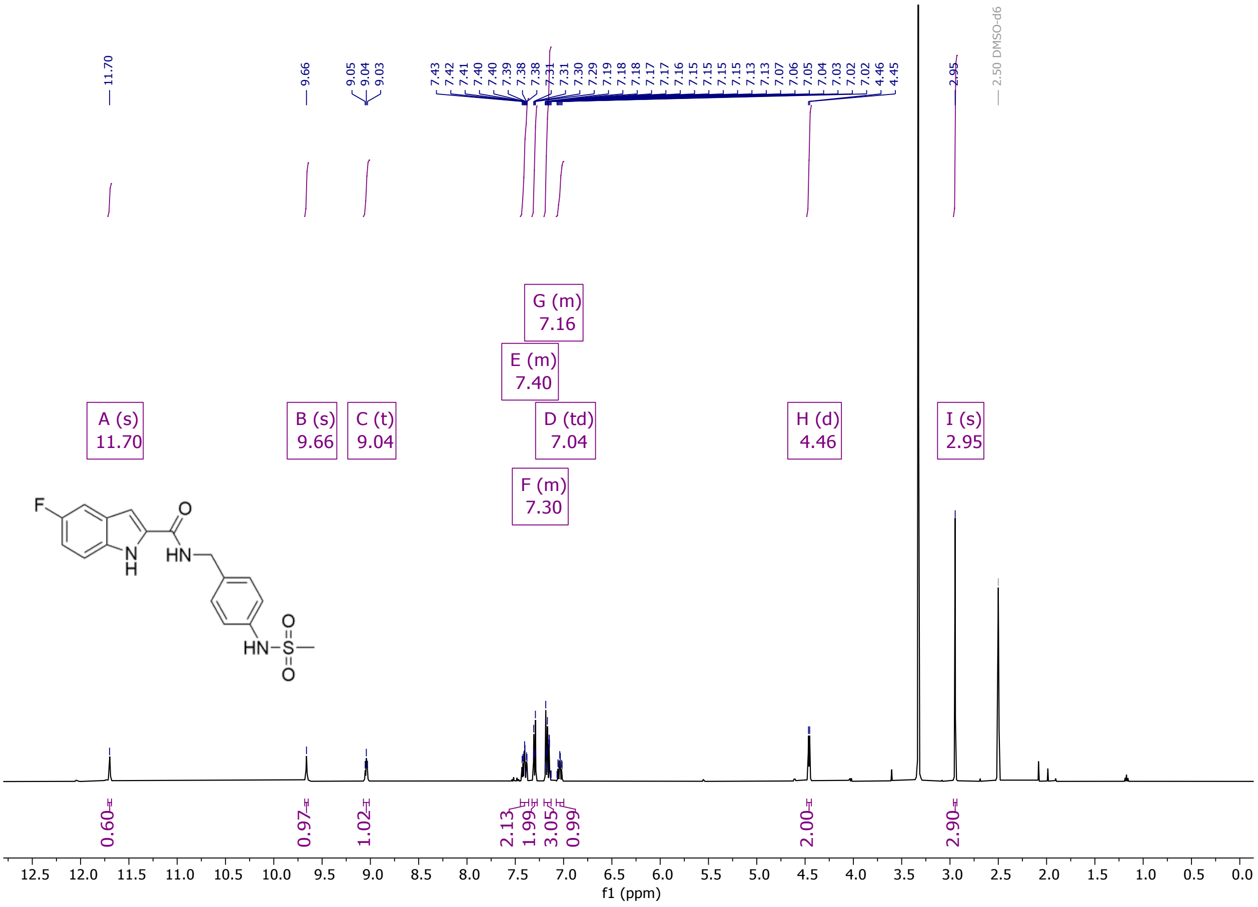

Figure S22: 5-fluoro-N-(4-(methylsulfonamido)benzyl)-1H-indole-2-carboxamide (8)

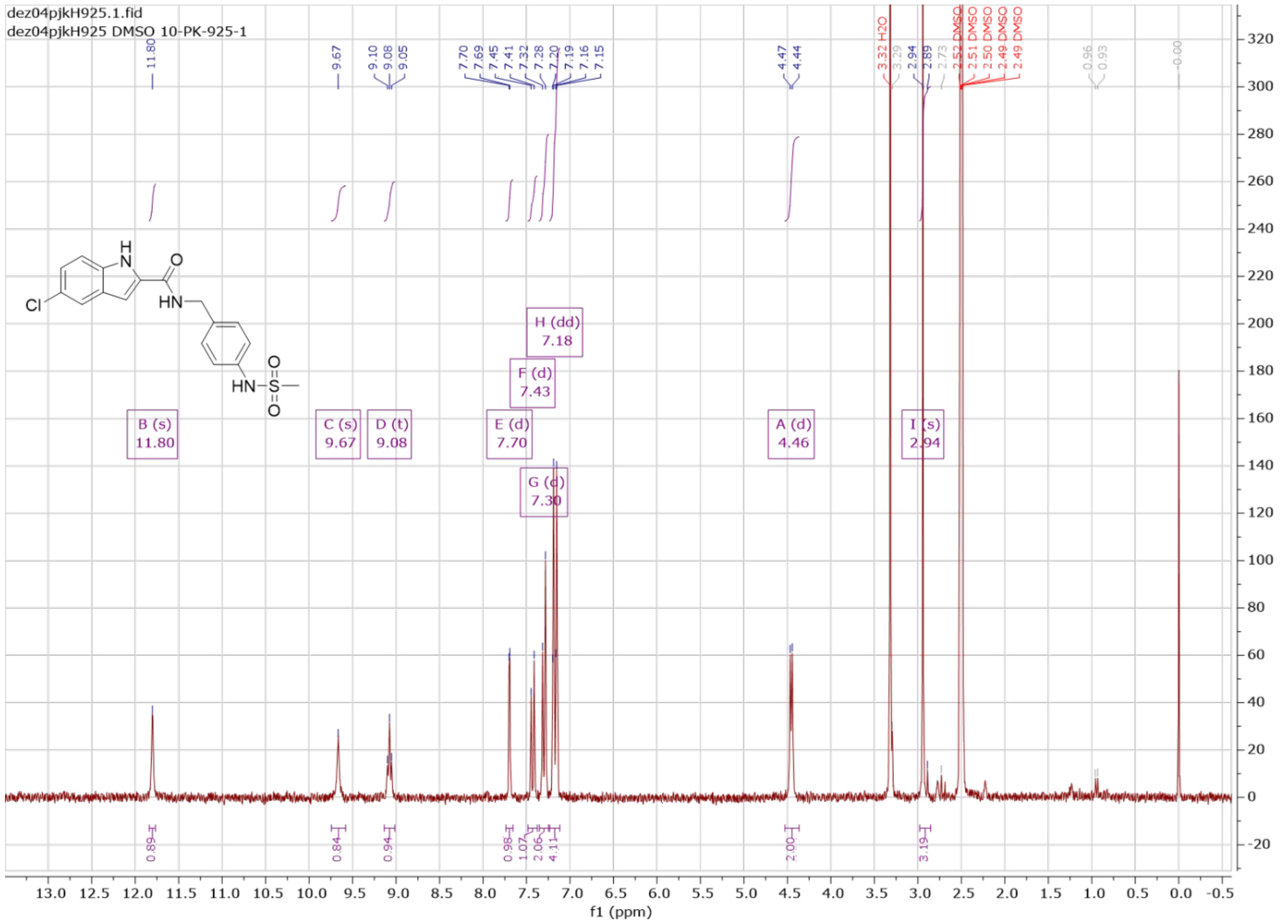

Figure S23: 5-chloro-N-(4-(methylsulfonamido)benzyl)-1H-indole-2-carboxamide (9)

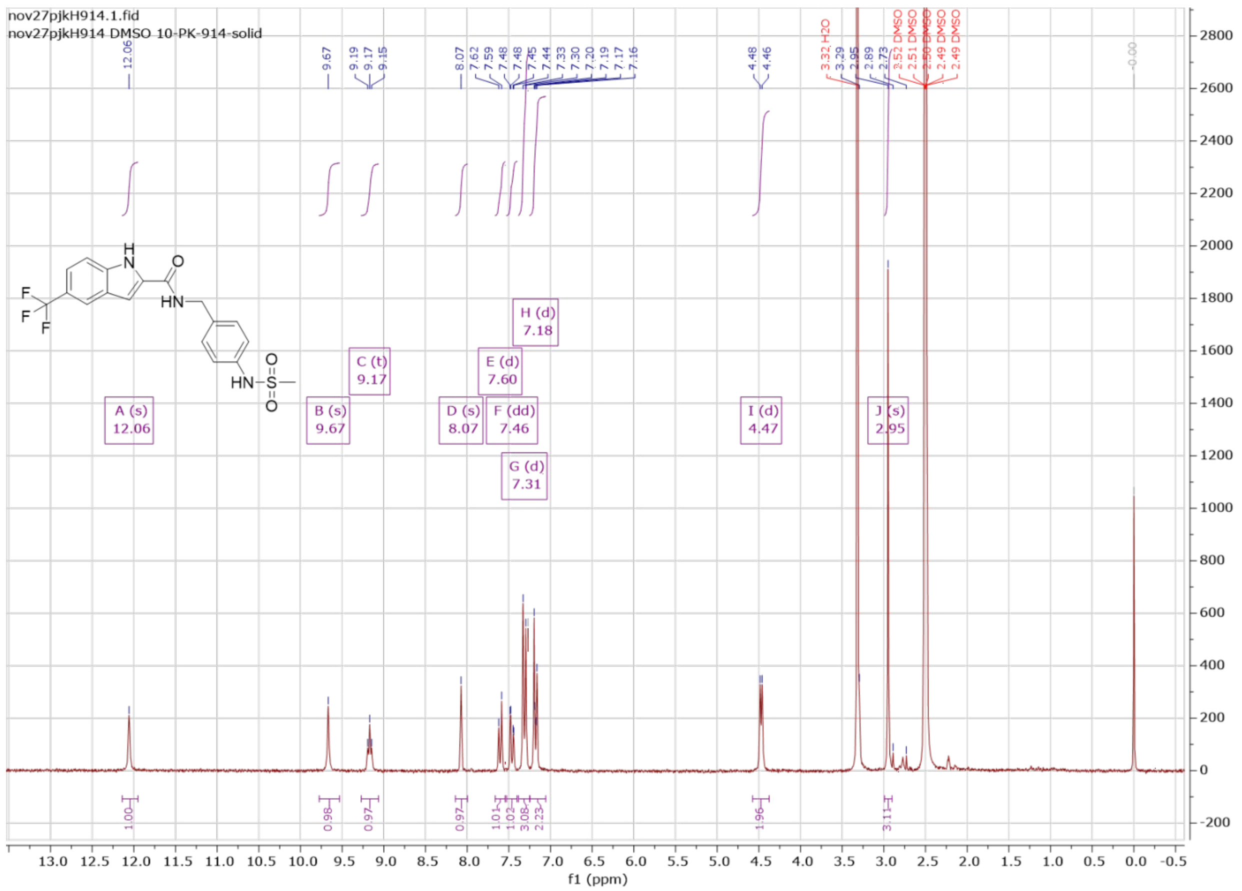

Figure S24: N-(4-(methylsulfonamido)benzyl)-5-(trifluoromethyl)-1H-indole-2-carboxamide (10)

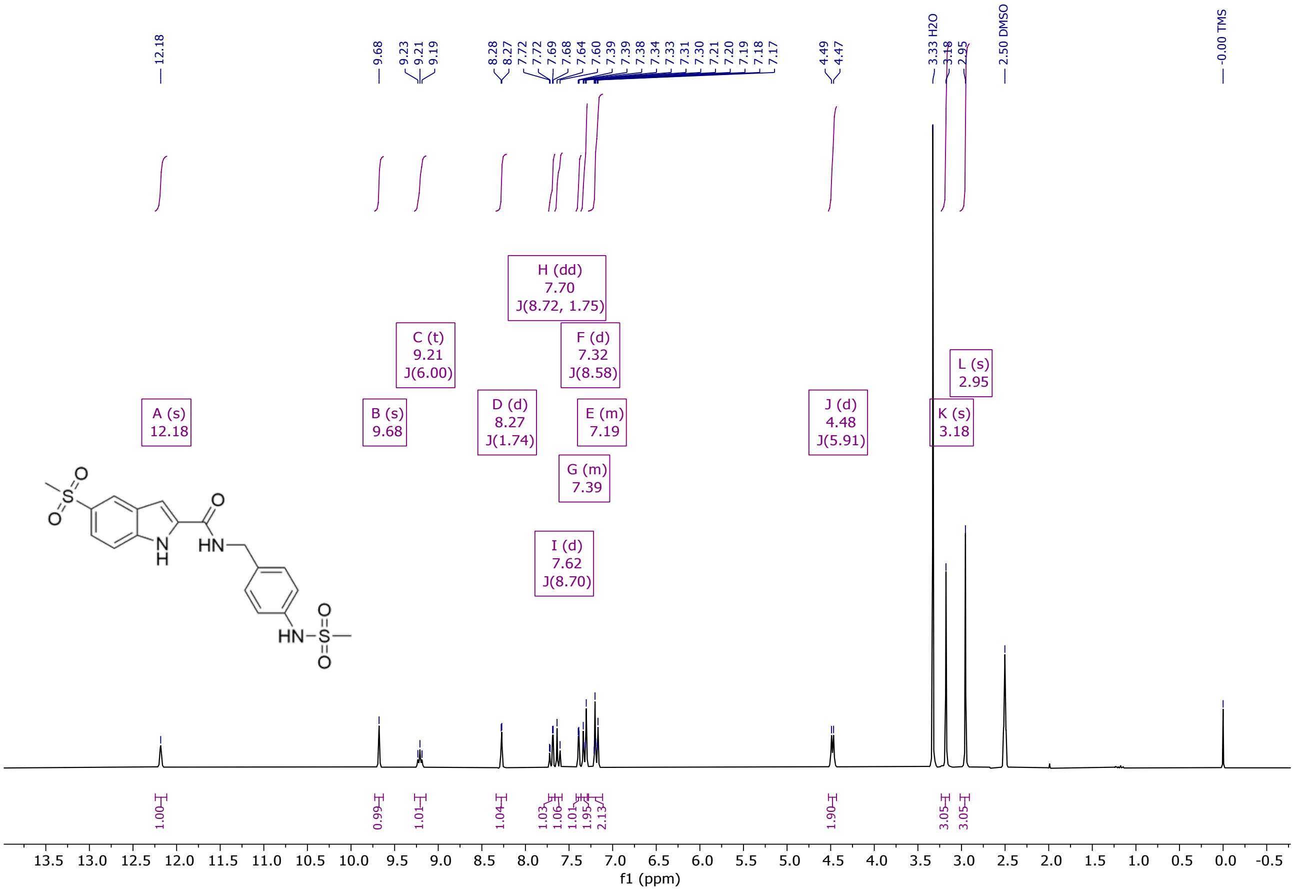

Figure S25: N-(4-(methylsulfonamido)benzyl)-5-(methylsulfonyl)-1H-indole-2-carboxamide (11)

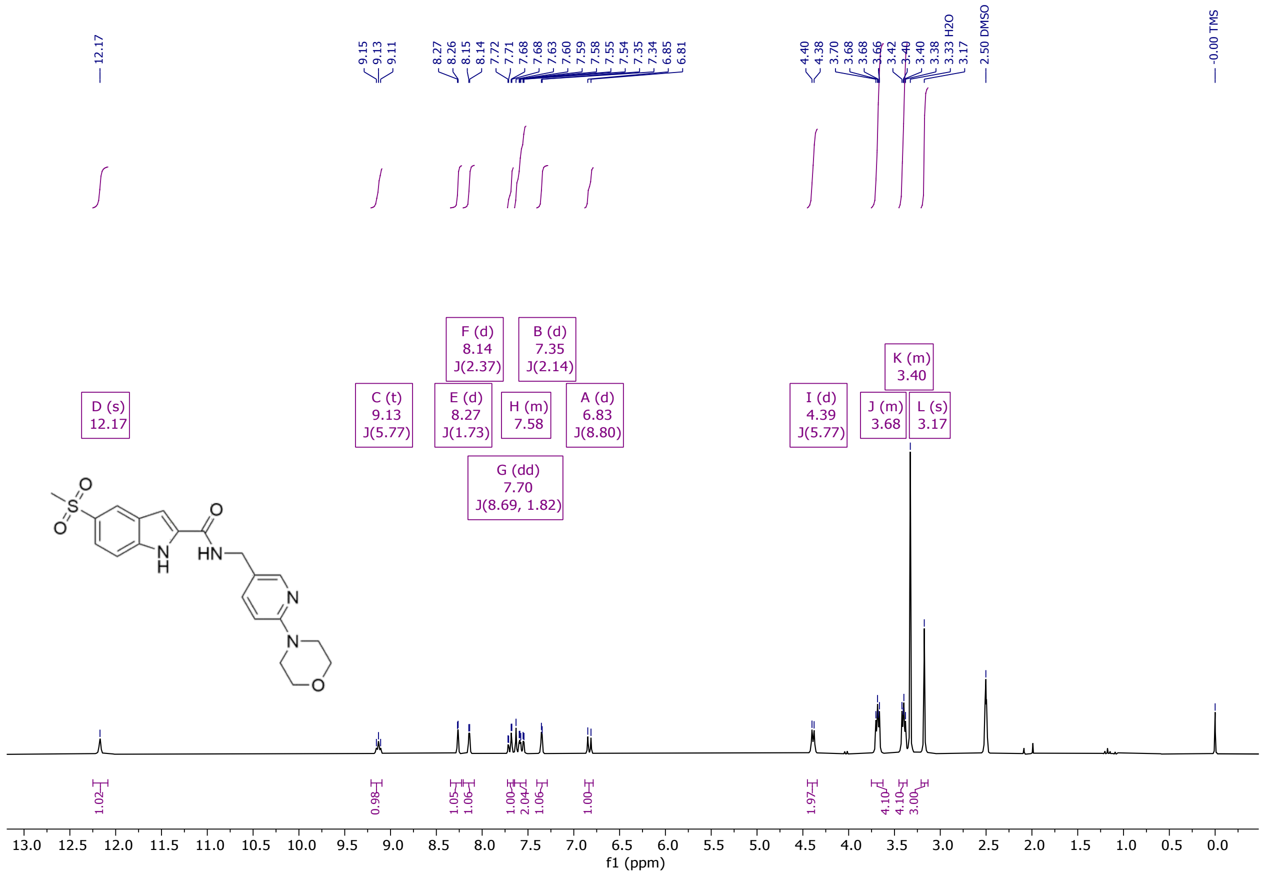

Figure S26: 5-(methylsulfonyl)-N-((6-morpholinopyridin-3-yl)methyl)-1H-indole-2-carboxamide (12)

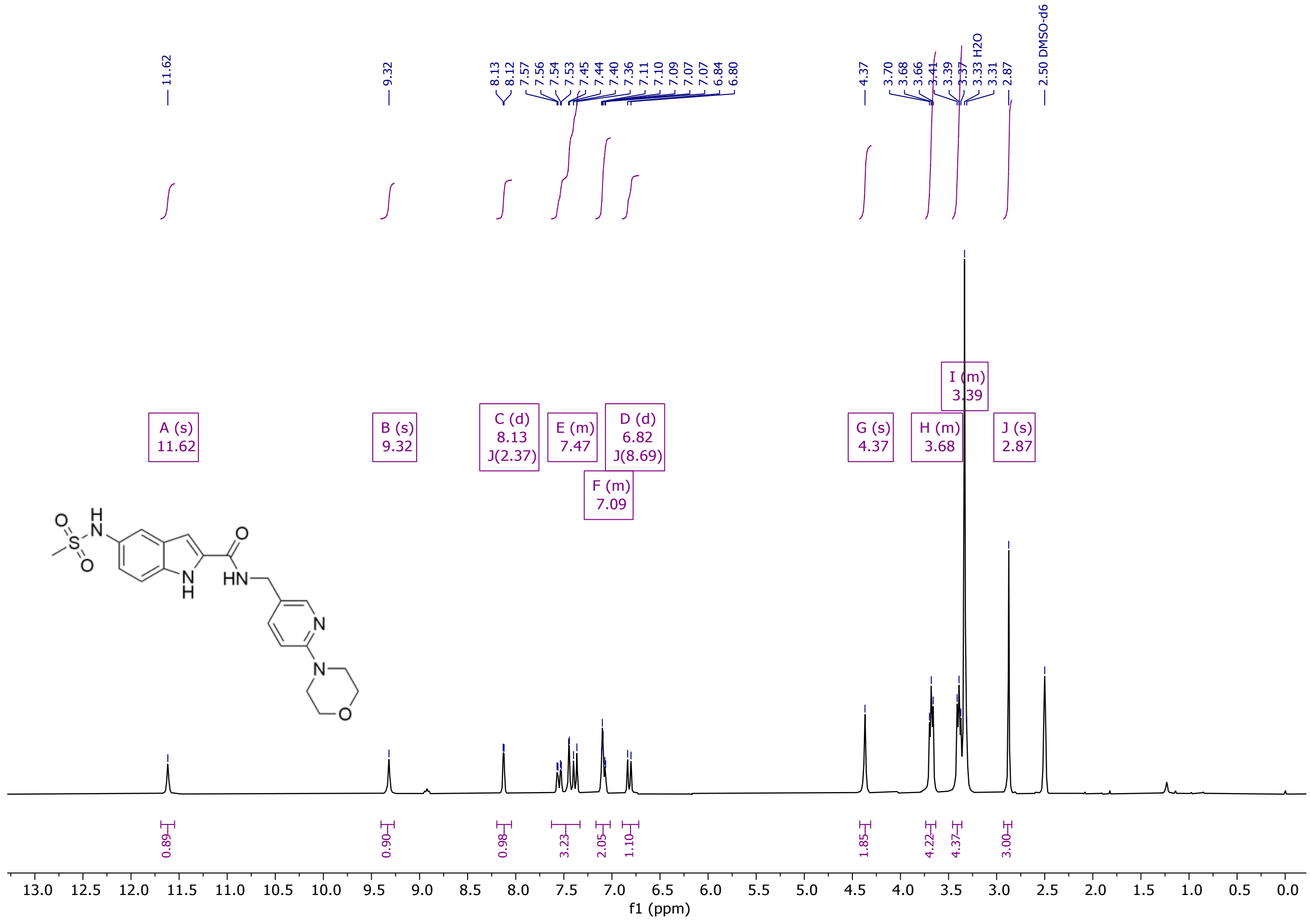

Figure S27: 5-(methylsulfonamido)-N-((6-morpholinopyridin-3-yl)methyl)-1H-indole-2-carboxamide (13)

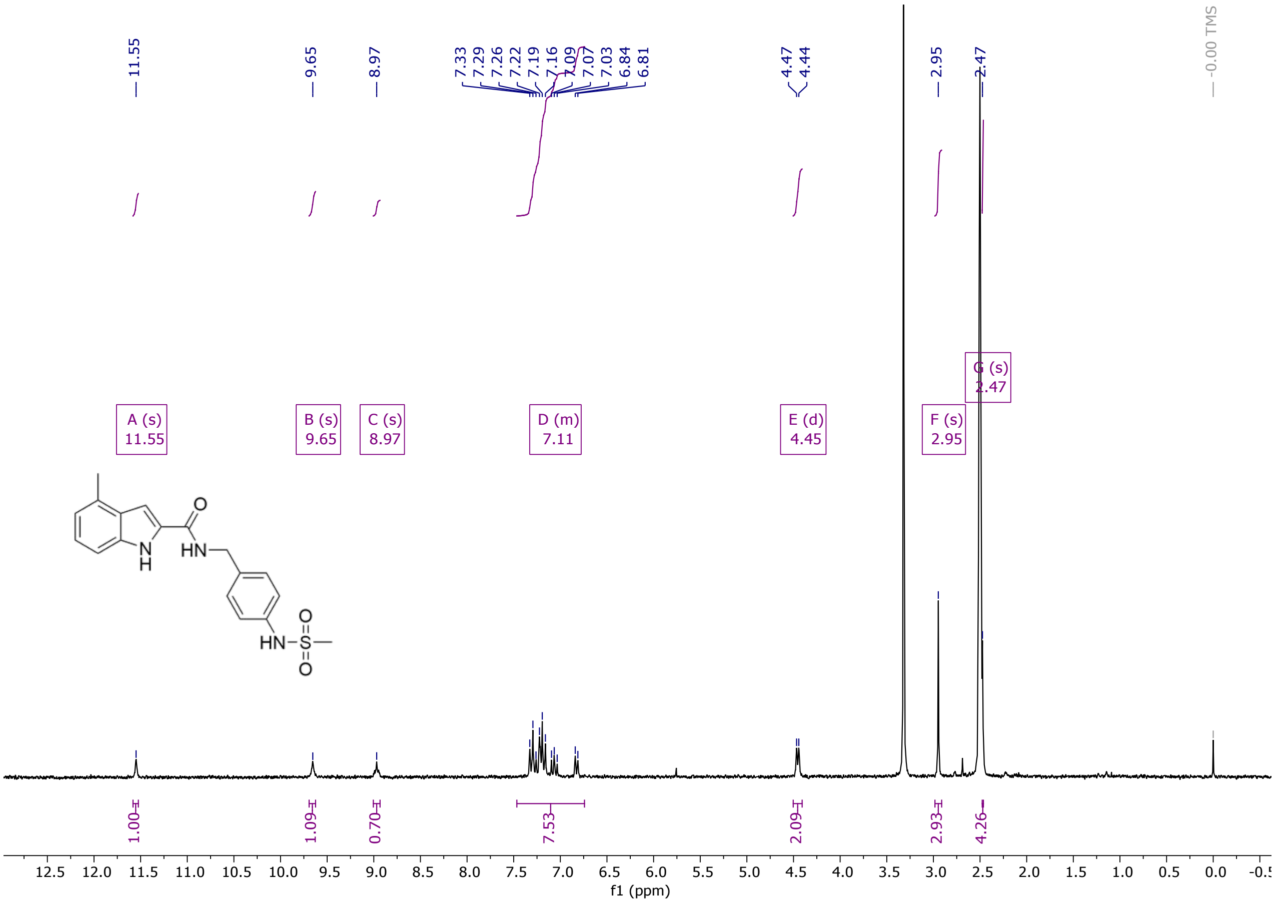

Figure S28: 4-methyl-N-(4-(methylsulfonamido)benzyl)-1H-indole-2-carboxamide (14)

This report was created by ACD/NMR Processor Academic Edition. For more information go to www.acdlabs.com/nmrproc/

Acquisition Time (sec)	1.5903	***Comment***	Dessoy - MAD1946 - Acetona - Av 500 MHz - set11madH2			***Date***	12 Sep 2019 11:41:28
File Name	\\nmrsparc.iqm.unicamp.br\espectros\avance500\2019\set19\Sala\Luiz Carlos\set11madH2_001001r					***Frequency (MHz)***	499.87
Nucleus	1H	***Number of Transients***	16	***Original Points Count***	16384	***Points Count***	65536
Pulse Sequence	zg30	***Solvent***	Acetone	***Spectrum Offset (Hz)***	3078.4922	***Sweep Width (Hz)***	10302.20
Temperature (degree C)	25.149						

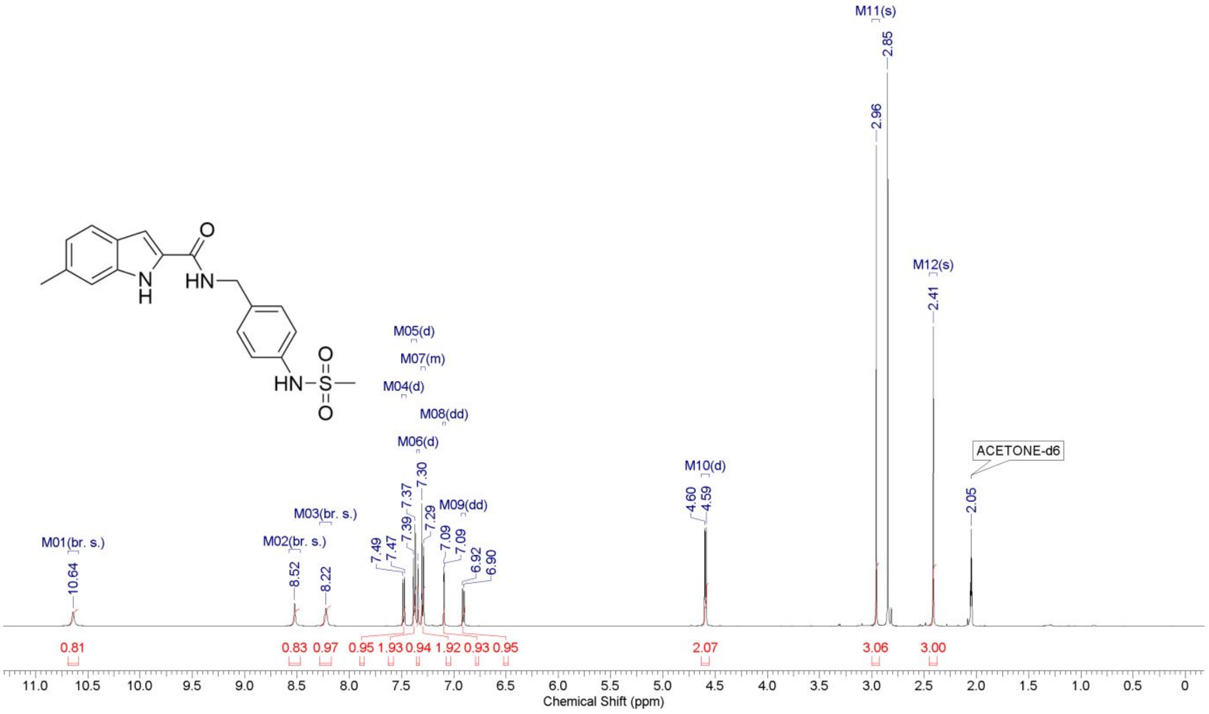

Figure S29: 6-methyl-N-(4-(methylsulfonamido)benzyl)-1H-indole-2-carboxamide (15)

This report was created by ACD/NMR Processor Academic Edition. For more information go to www.acdlabs.com/nmrproc/

**Acquisition Time (sec)**	1.5903	**Comment**	Dessoy - MAD 1948 - DMSO - AV 500 MHz - set23madH1			**Date**	23 Sep 2019 08:42:40
**File Name**	\\nmrsparc.iqm.unicamp.br\espectros\avance500\2019\set19\Sala\Luiz Carlos\set23madH1_001001r					**Frequency (MHz)**	499.87
**Nucleus**	1H	**Number of Transients**	16	**Original Points Count**	16384	**Points Count**	65536
**Pulse Sequence**	zg30	**Solvent**	DMSO-d6	**Spectrum Offset (Hz)**	3083.5854	**Sweep Width (Hz)**	10302.20
**Temperature (degree C)**	25.144						

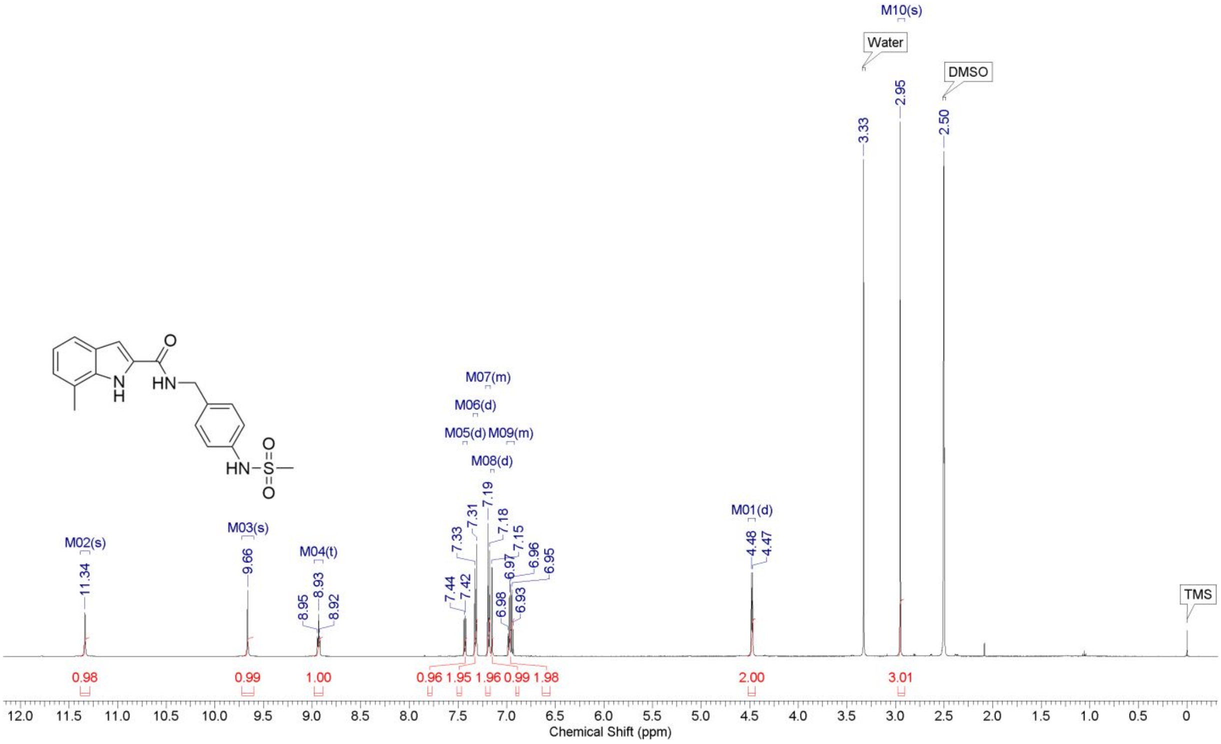

Figure S30: 7-methyl-N-(4-(methylsulfonamido)benzyl)-1H-indole-2-carboxamide (16)

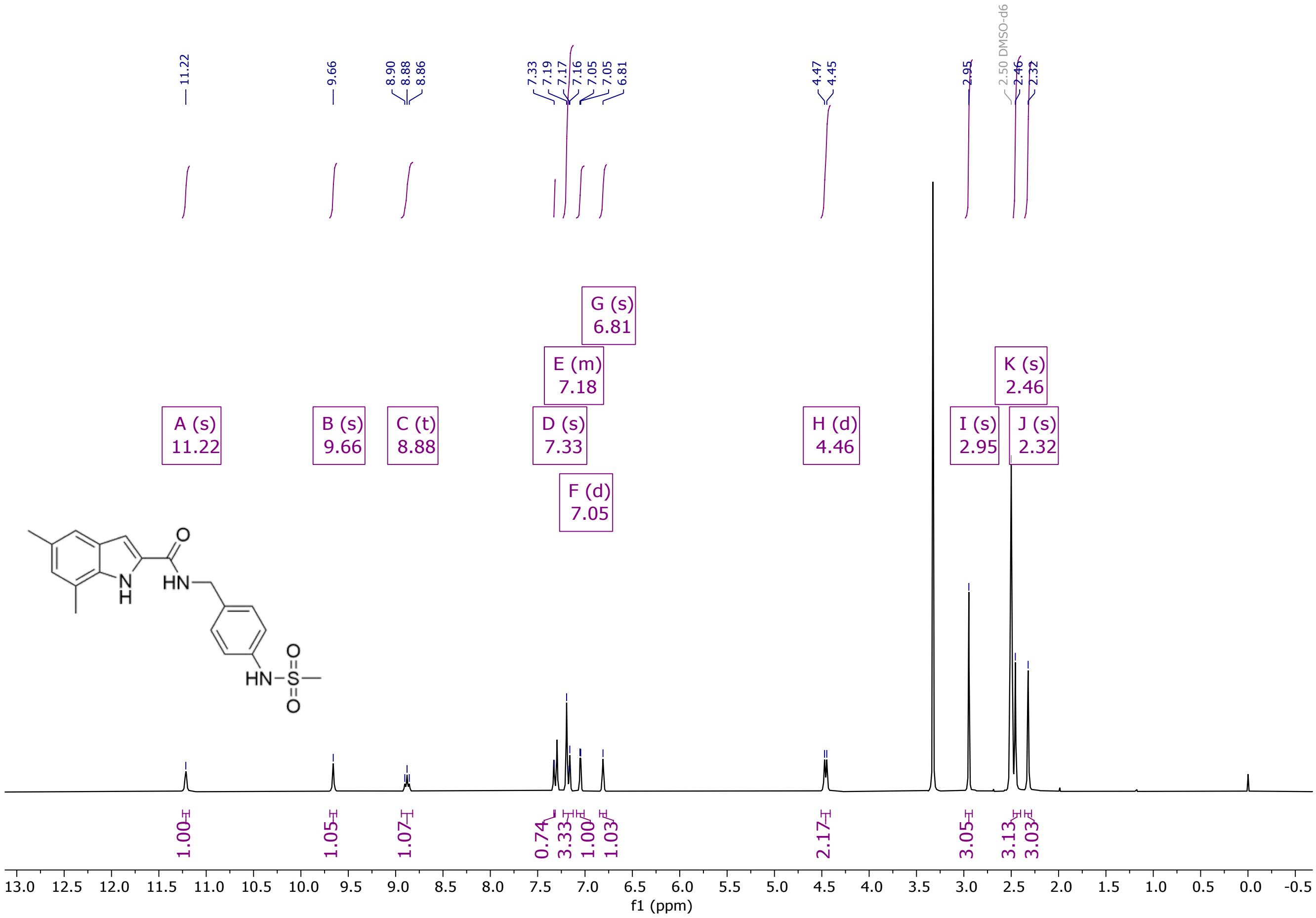

Figure S31: 5,7-dimethyl-N-(4-(methylsulfonamido)benzyl)-1H-indole-2-carboxamide (17)

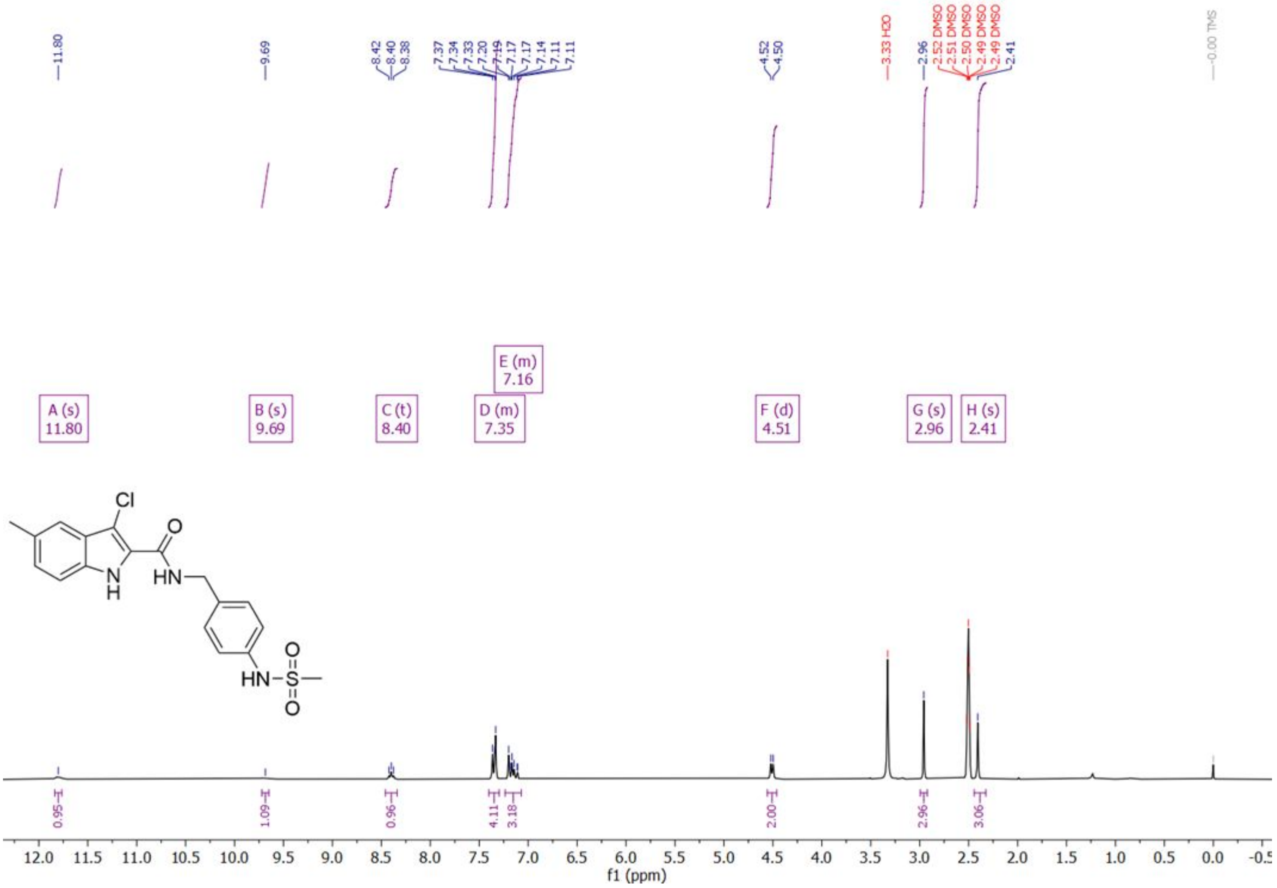

Figure S32: 3-chloro-5-methyl-N-(4-(methylsulfonamido)benzyl)-1H-indole-2-carboxamide (18)

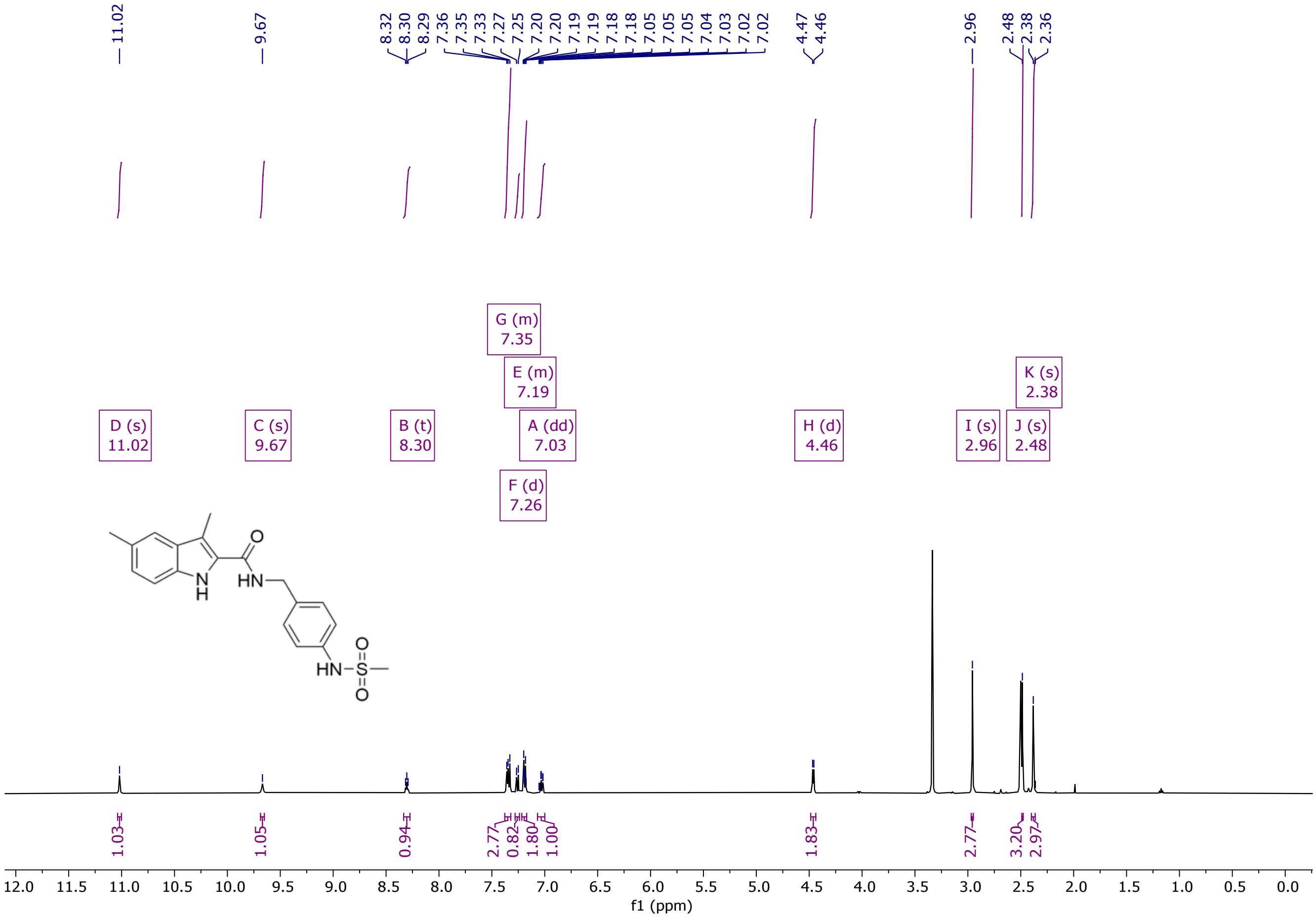

Figure S33: 3,5-dimethyl-N-(4-(methylsulfonamido)benzyl)-1H-indole-2-carboxamide (19)

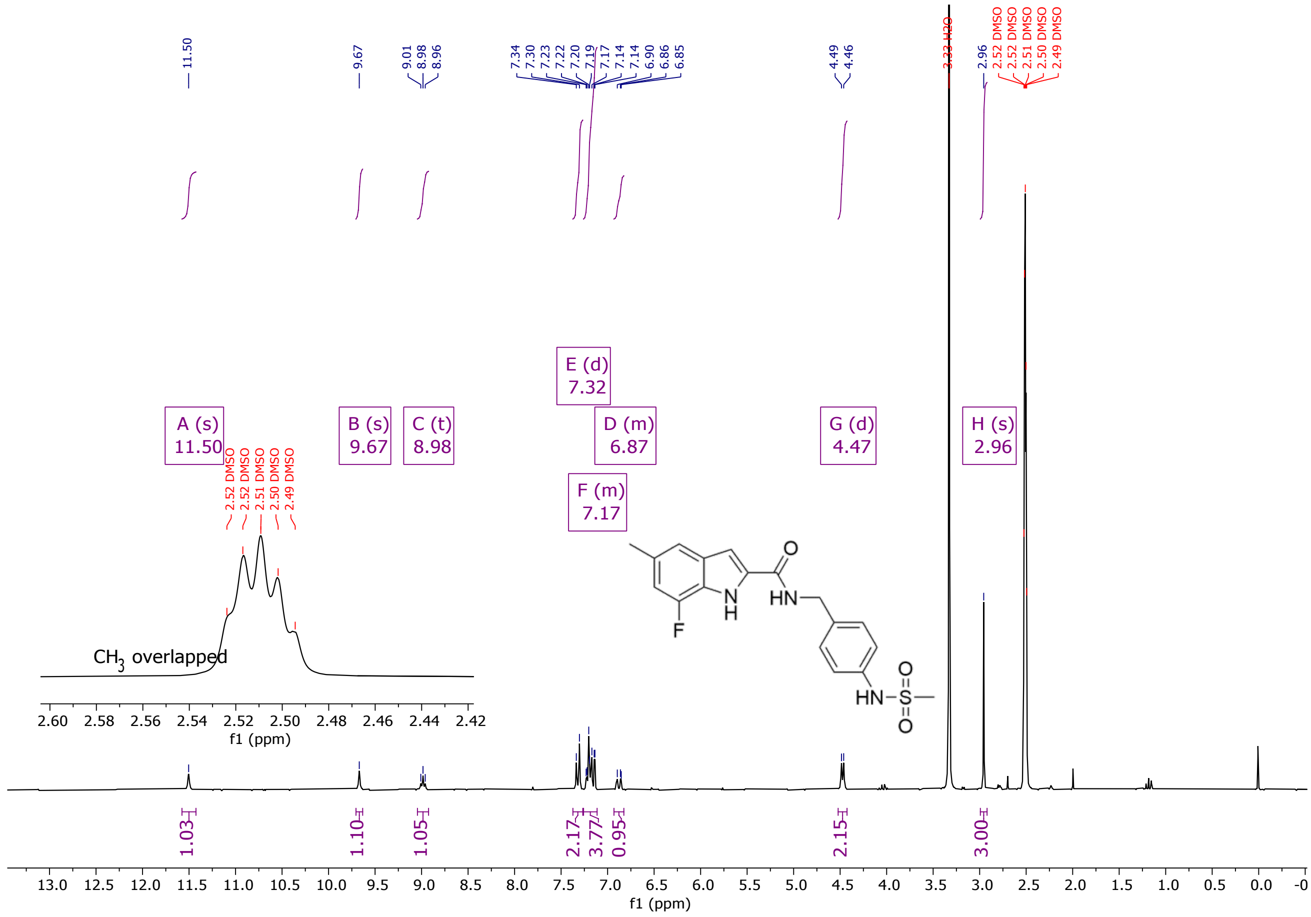

Figure S34: 7-fluoro-5-methyl-N-(4-(methylsulfonamido)benzyl)-1H-indole-2-carboxamide (20)

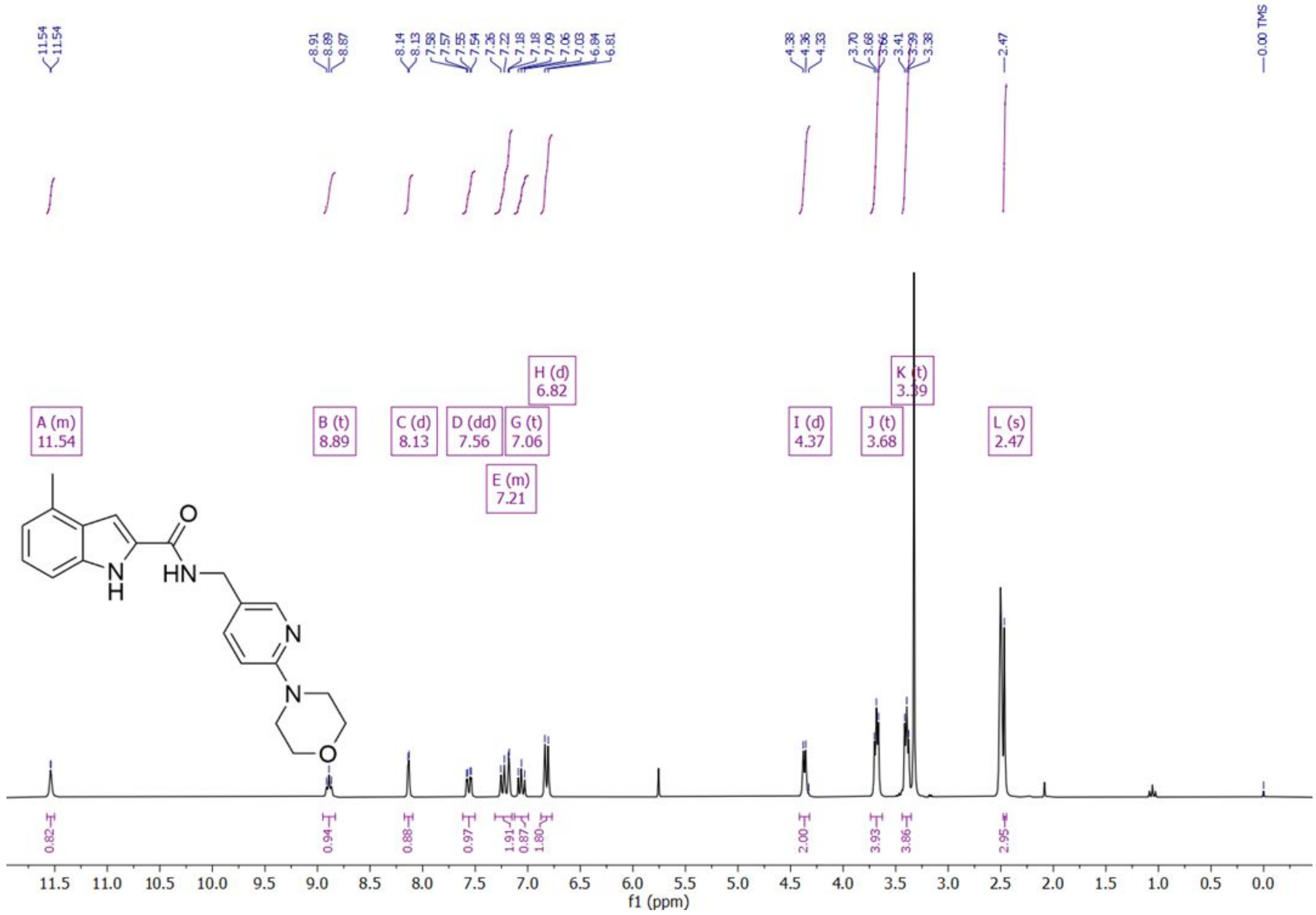

Figure S35: 4-methyl-N-((6-morpholinopyridin-3-yl)methyl)-1H-indole-2-carboxamide (21)

This report was created by ACD/NMR Processor Academic Edition. For more information go to www.acdlabs.com/nmrproc/

Acquisition Time (sec)	1.5903	***Comment***	Dessoy - mad 1938 - CDCl3 - Av 500 MHz - set04madH1			***Date***	04 Sep 2019 12:05:52
File Name	\\nmrsparc.iqm.unicamp.br\espectros\avance500\2019\set19\Sala\Luiz Carlos\set04madH1_001001r					***Frequency (MHz)***	499.87
Nucleus	1H	***Number of Transients***	16	***Original Points Count***	16384	***Points Count***	65536
Pulse Sequence	zg30	***Solvent***	CHLOROFORM-d	***Spectrum Offset (Hz)***	3079.6836	***Sweep Width (Hz)***	10302.20
Temperature (degree C)	25.147						

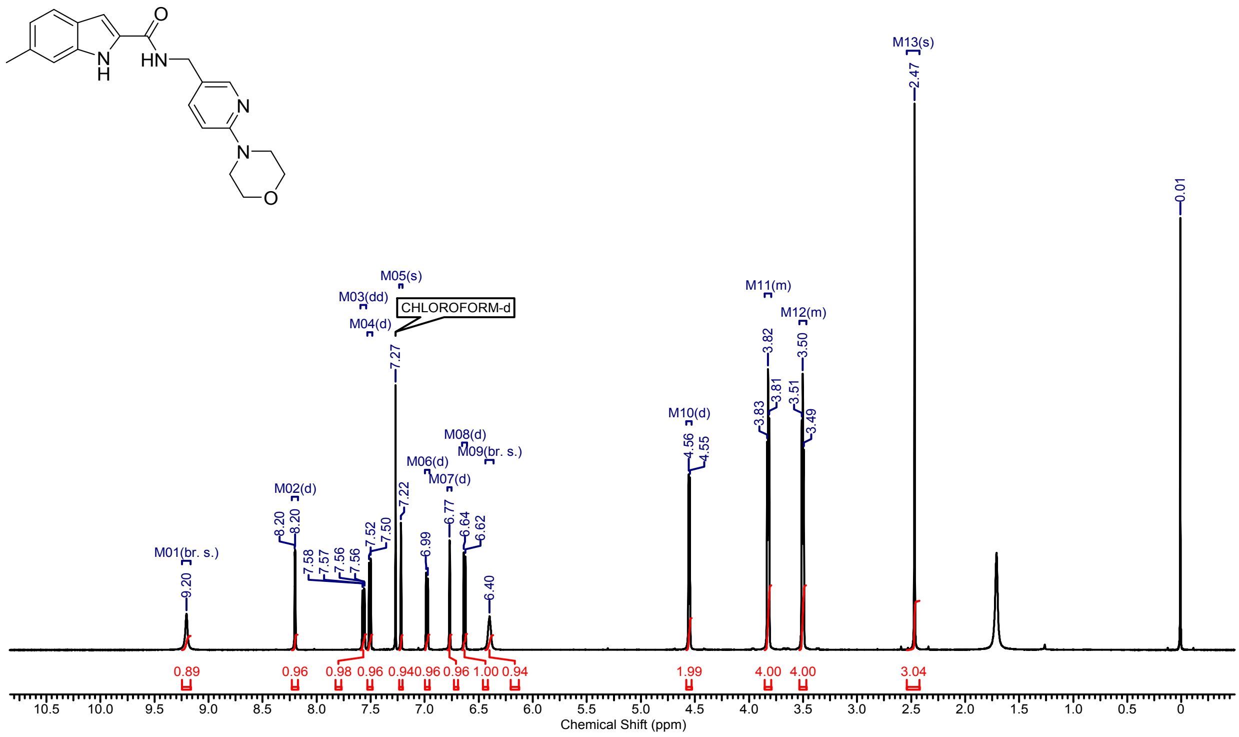

Figure S36: 6-methyl-N-((6-morpholinopyridin-3-yl)methyl)-1H-indole-2-carboxamide (22)

This report was created by ACD/NMR Processor Academic Edition. For more information go to www.acdlabs.com/nmrproc/

Acquisition Time (sec)	3.2768	***Date***	02 Sep 2019 15:27:56				
File Name	\\nmrsparc.iqm.unicamp.br\espectros\bruker250\2019\set19\Reserva\Luiz Carlos\sep02madH2_001001r					***Frequency (MHz)***	250.13
Nucleus	1H	***Number of Transients***	16	***Original Points Count***	16384	***Points Count***	65536
Pulse Sequence	zg30	***Solvent***	CHLOROFORM-d	***Spectrum Offset (Hz)***	1540.9979	***Sweep Width (Hz)***	5000.00
Temperature (degree C)	25.151						

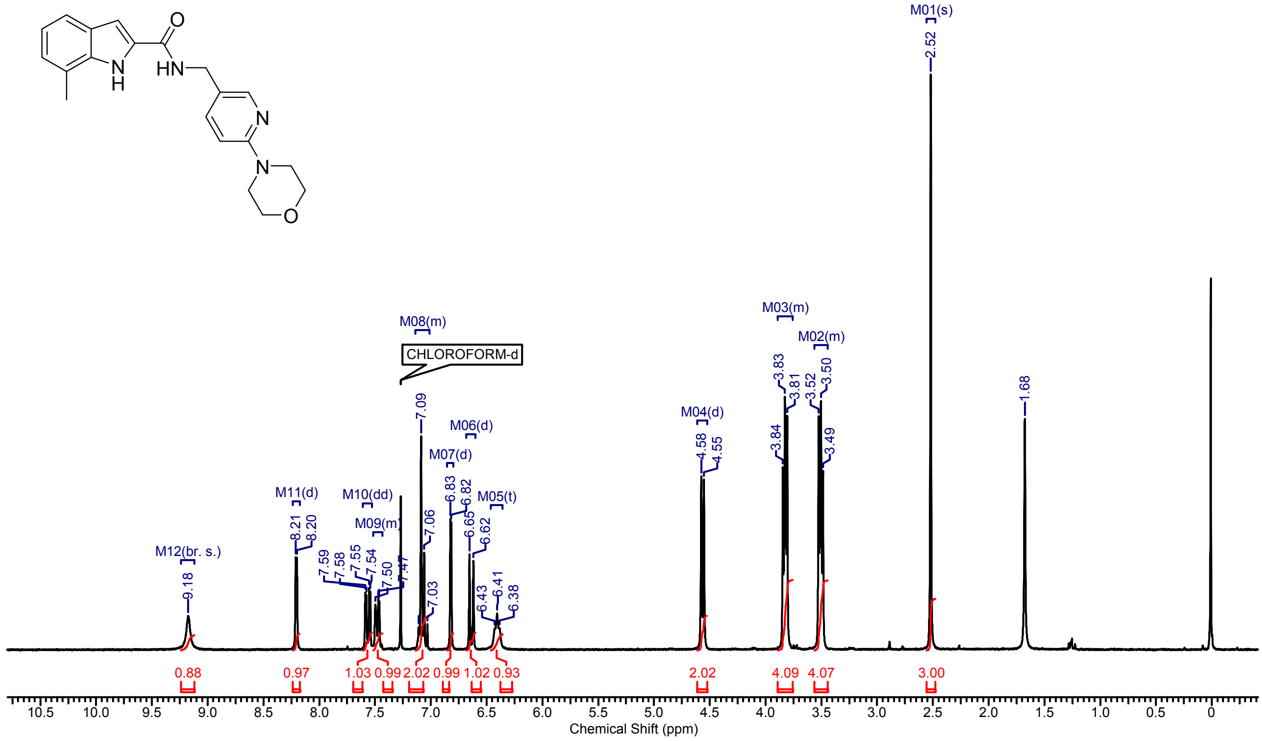

Figure S37: 7-methyl-N-((6-morpholinopyridin-3-yl)methyl)-1H-indole-2-carboxamide (23)

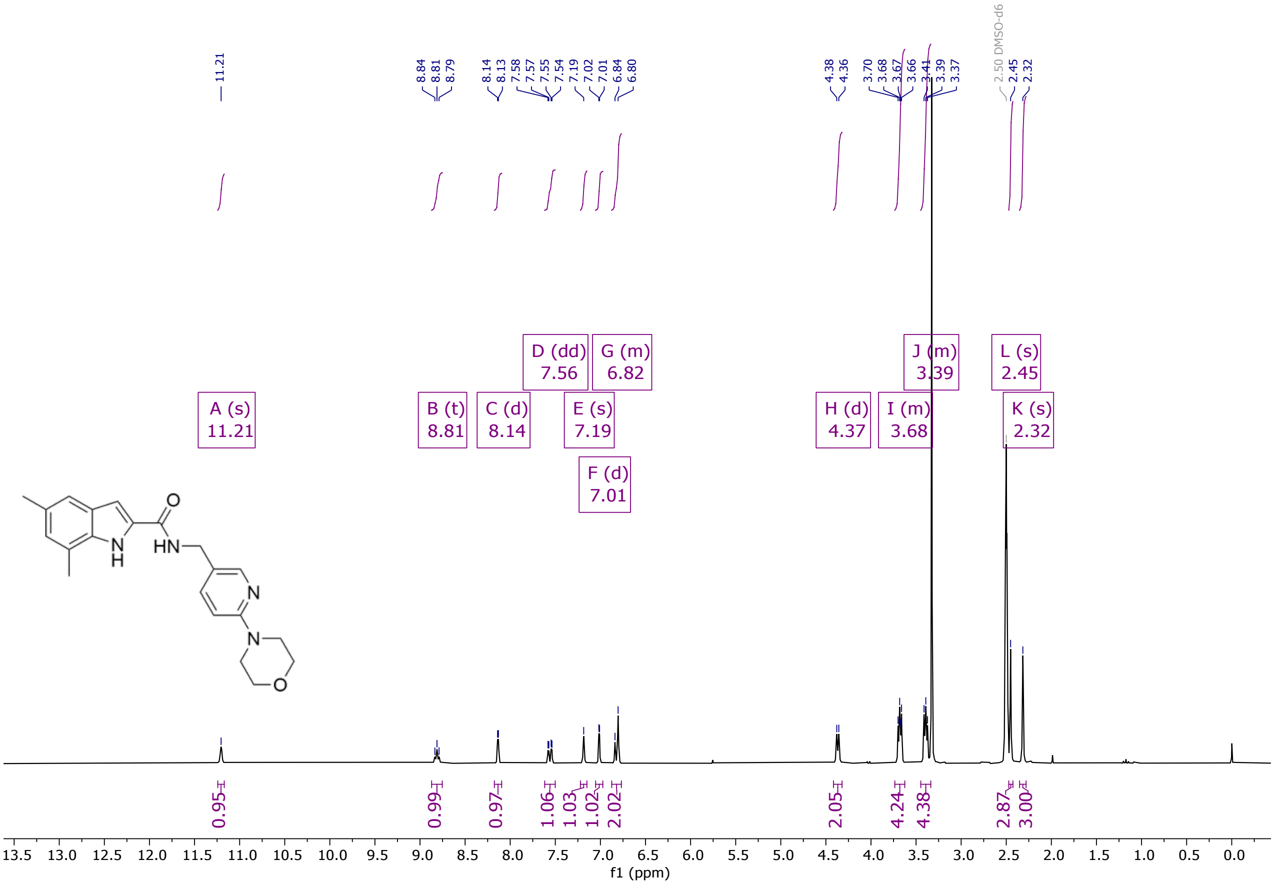

Figure S38: 5,7-dimethyl-N-((6-morpholinopyridin-3-yl)methyl)-1H-indole-2-carboxamide (24)

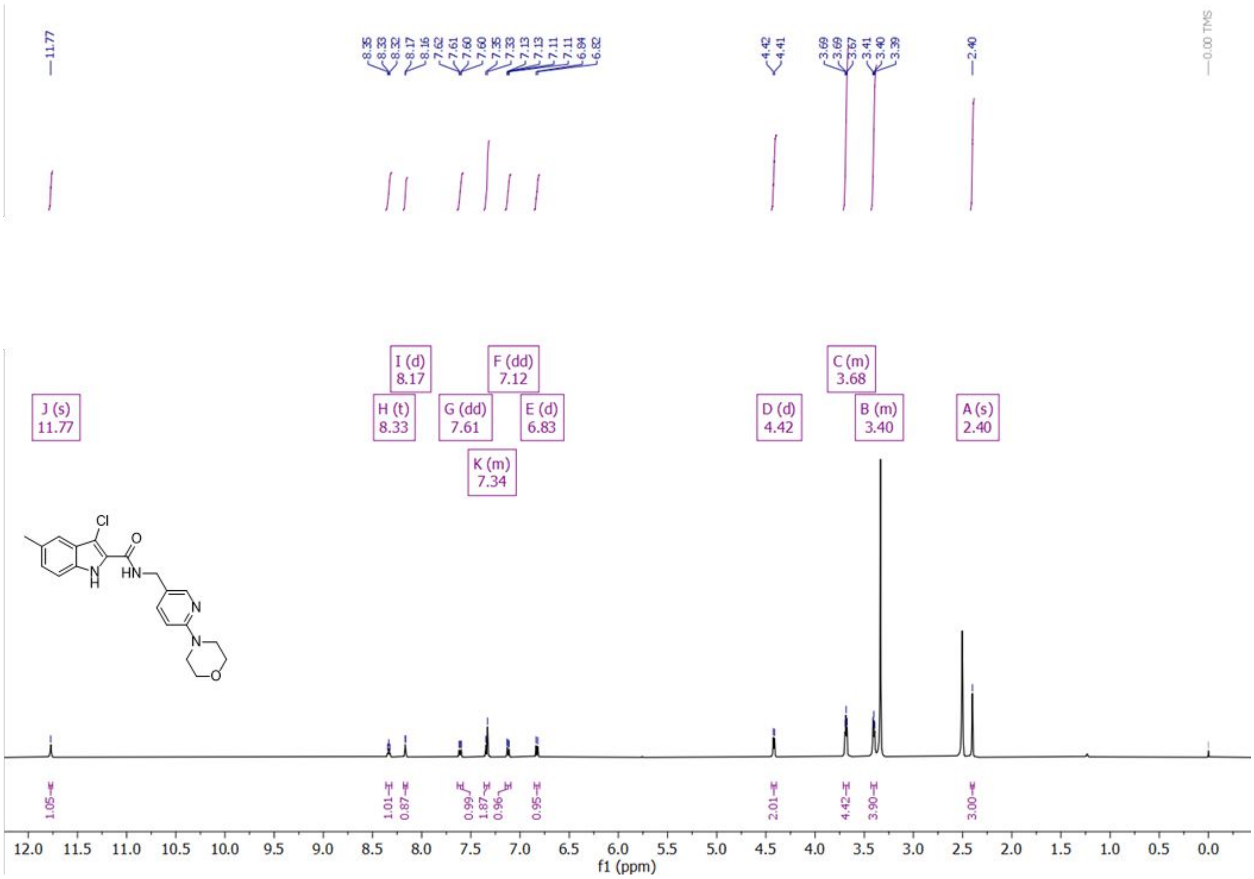

Figure S39: 3-chloro-5-methyl-N-((6-morpholinopyridin-3-yl)methyl)-1H-indole-2-carboxamide (25)

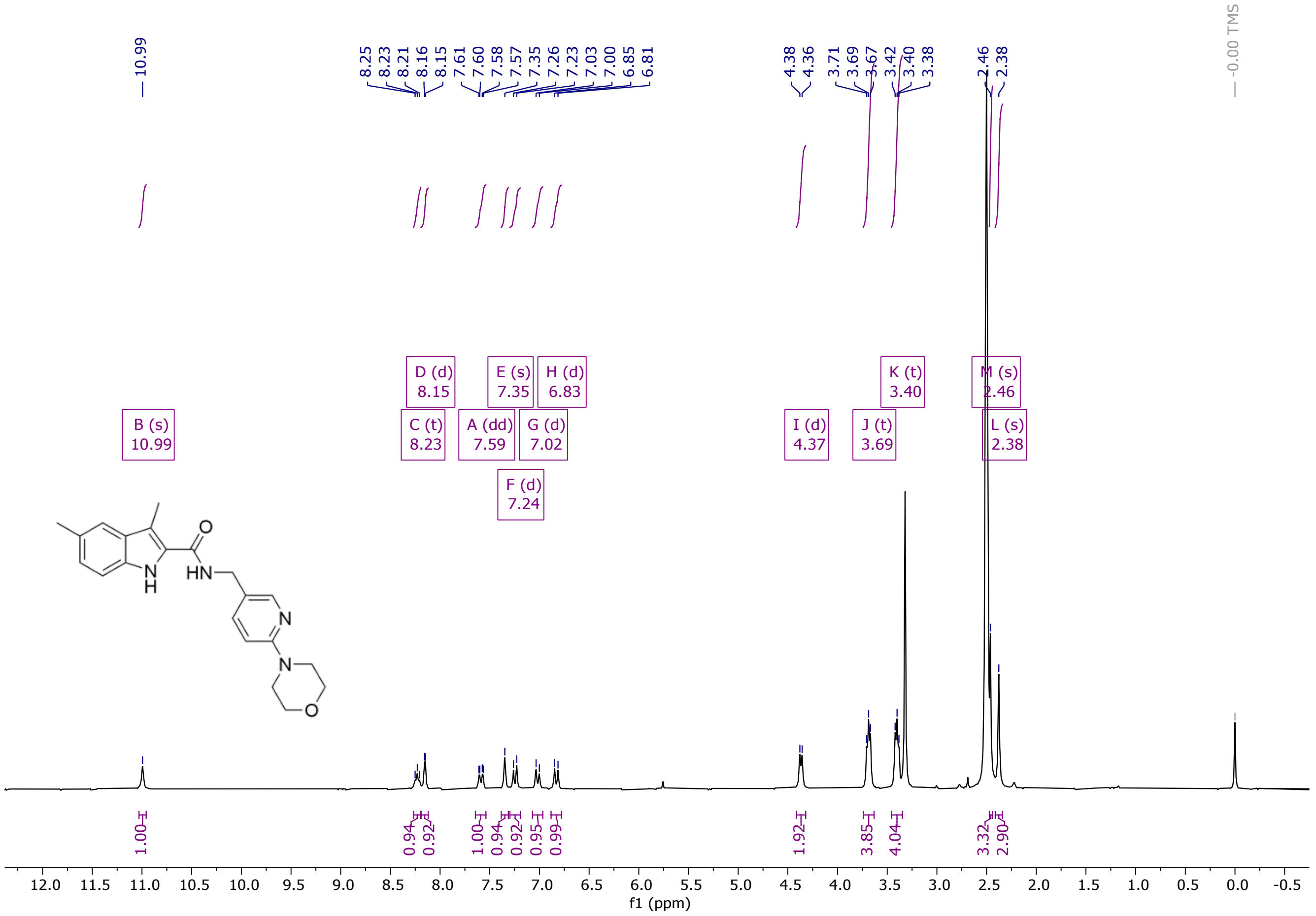

Figure S40: 3,5-dimethyl-N-((6-morpholinopyridin-3-yl)methyl)-1H-indole-2-carboxamide (26)

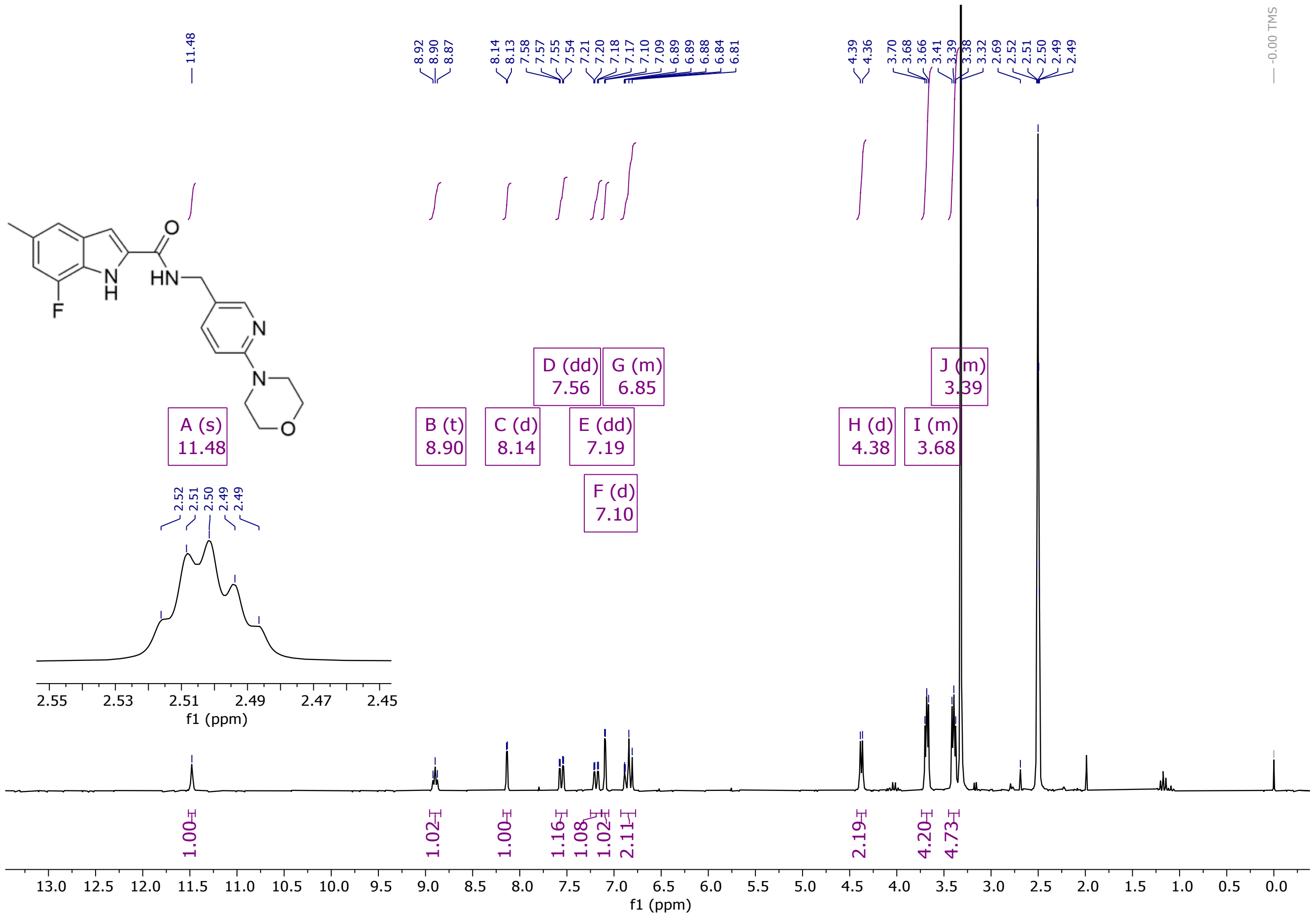

Figure S41: 7-fluoro-5-methyl-N-((6-morpholinopyridin-3-yl)methyl)-1H-indole-2-carboxamide (27)

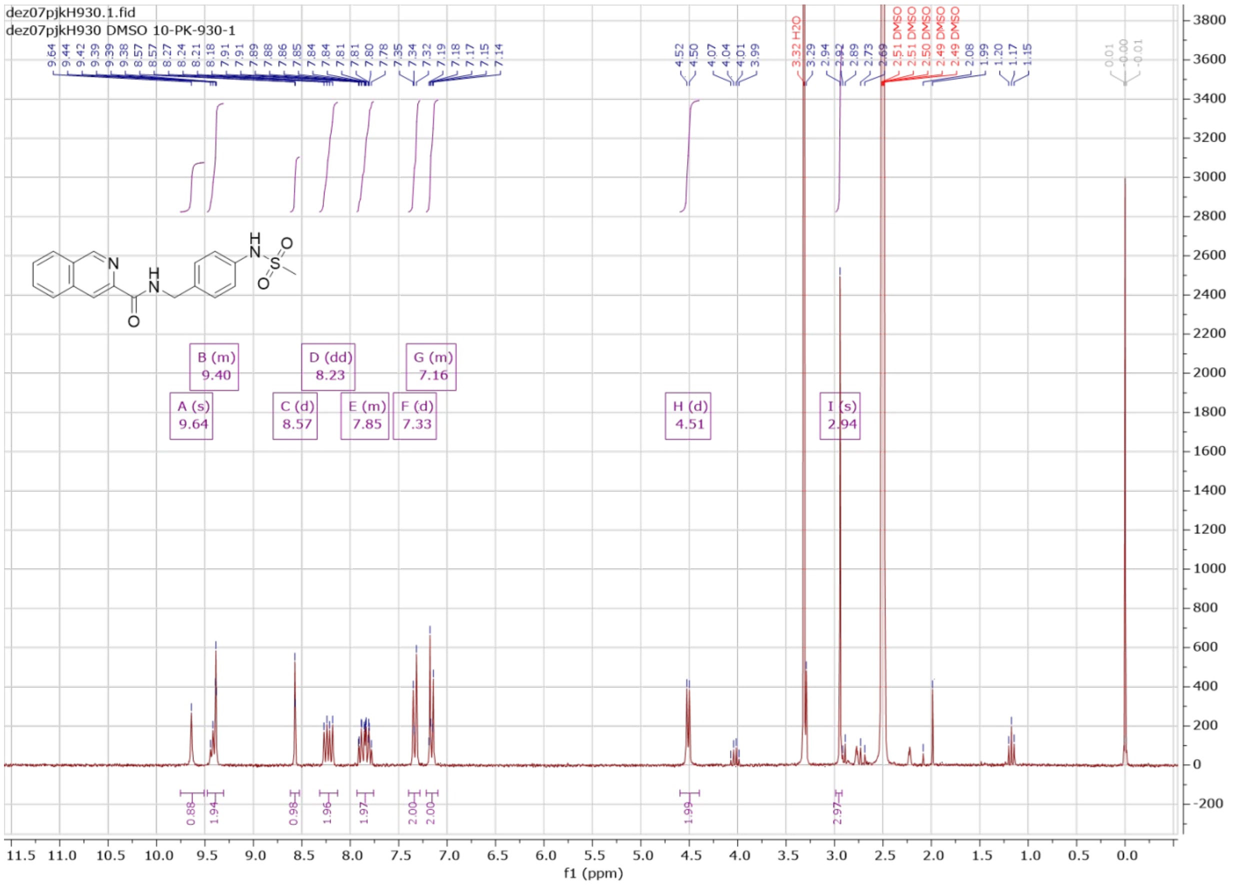

Figure S42: N-(4-(methylsulfonamido)benzyl)isoquinoline-3-carboxamide (28)

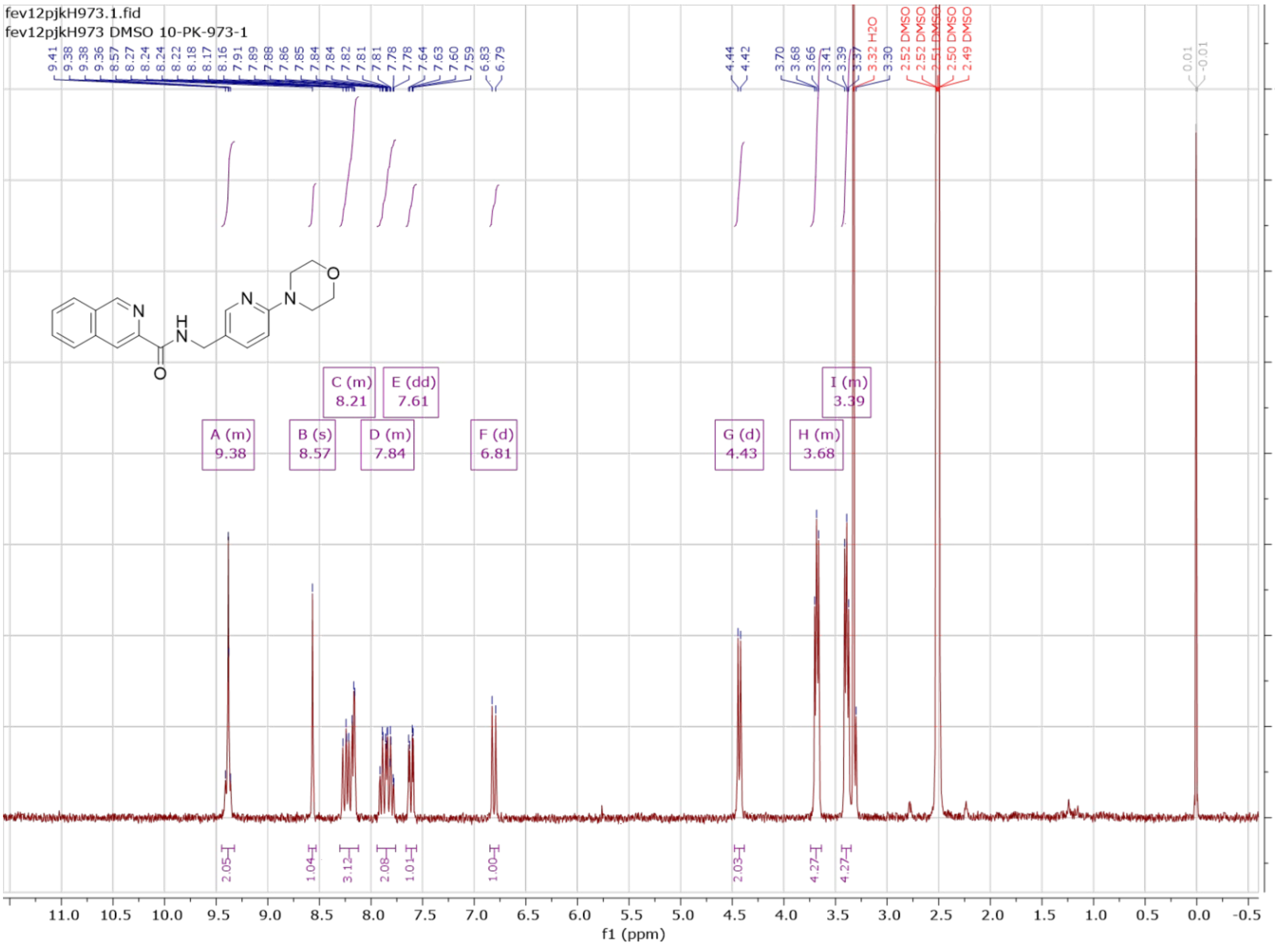

Figure S43: N-((6-morpholinopyridin-3-yl)methyl)isoquinoline-3-carboxamide (29)

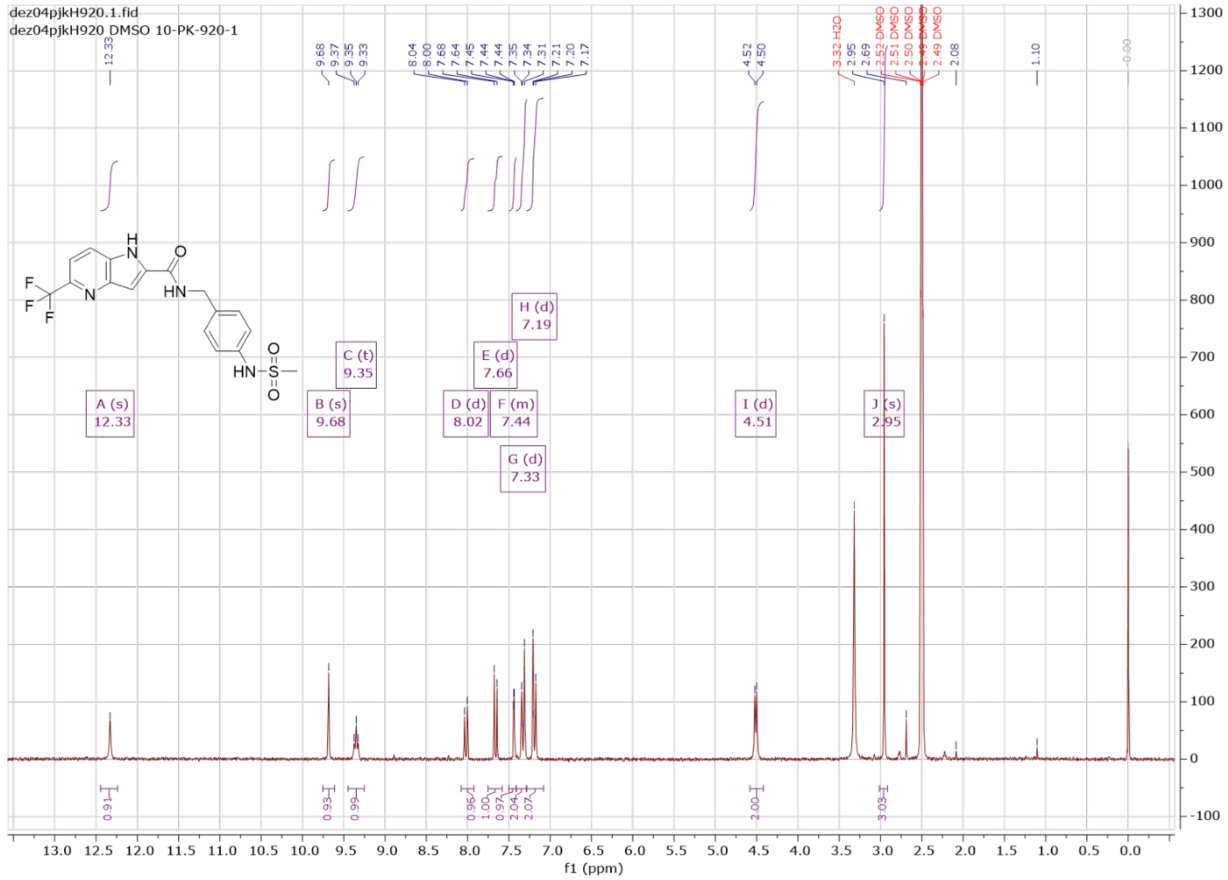

Figure S44: N-(4-(methylsulfonamido)benzyl)-5-(trifluoromethyl)-1H-pyrrolo[3,2-b]pyridine-2-carboxamide (30)

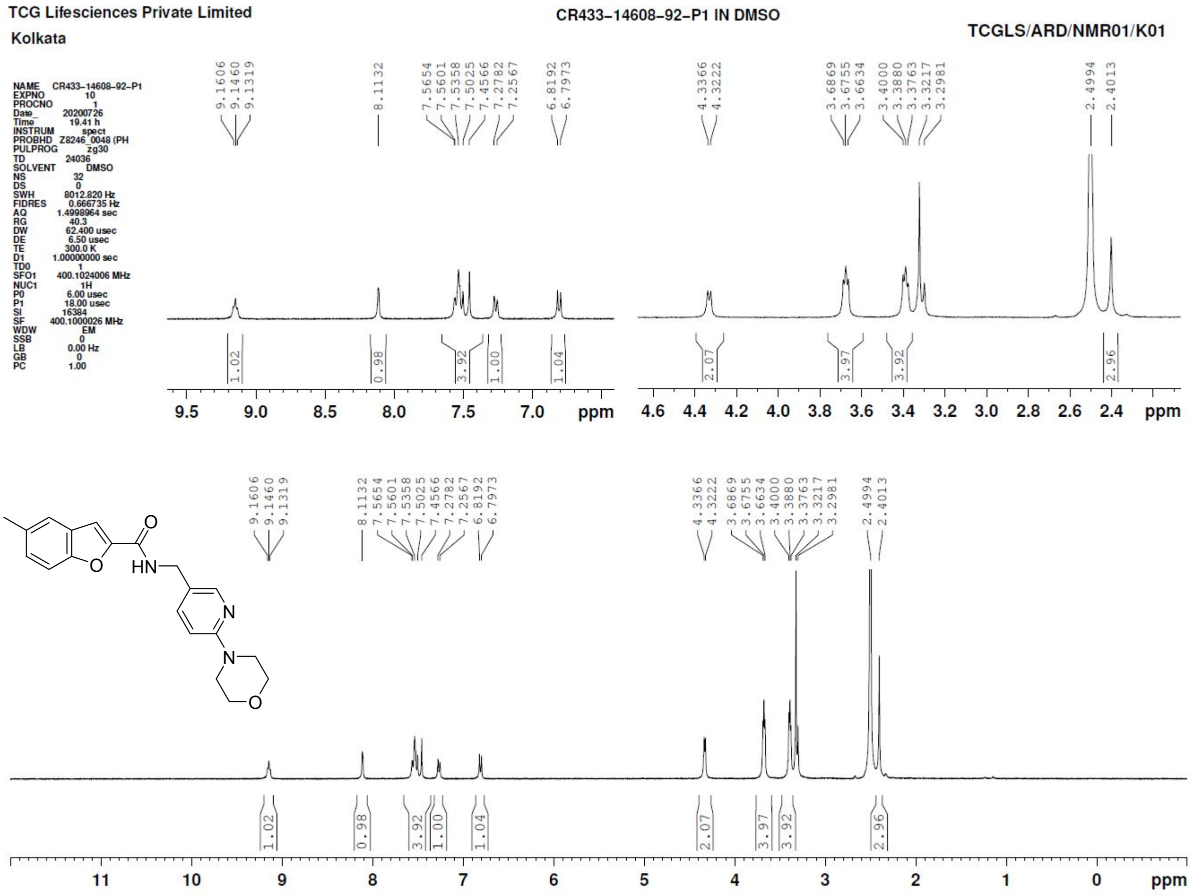

Figure S45: 5-methyl-N-((6-morpholinopyridin-3-yl)methyl)benzofuran-2-carboxamide (31)

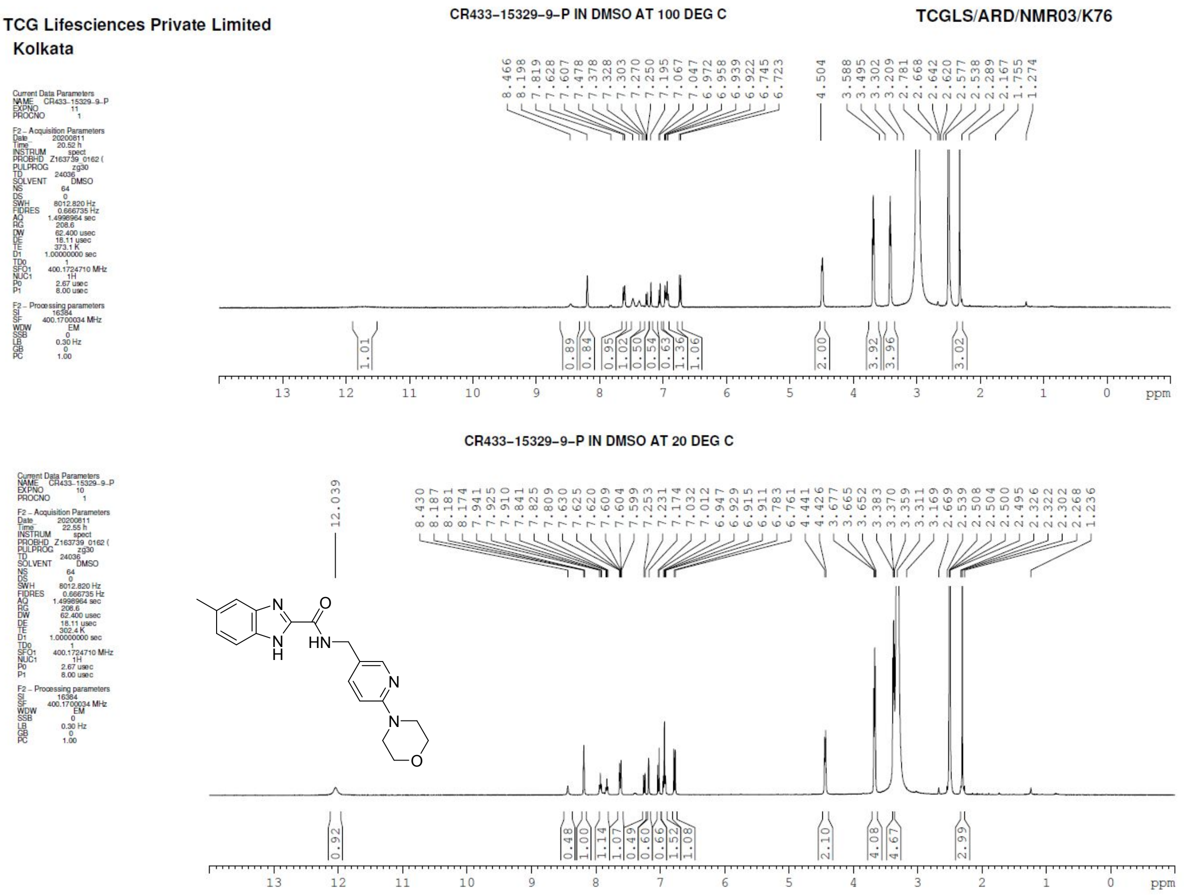

Figure S46: 5-methyl-N-((6-morpholinopyridin-3-yl)methyl)-1H-benzo[d]imidazole-2-carboxamide (32)

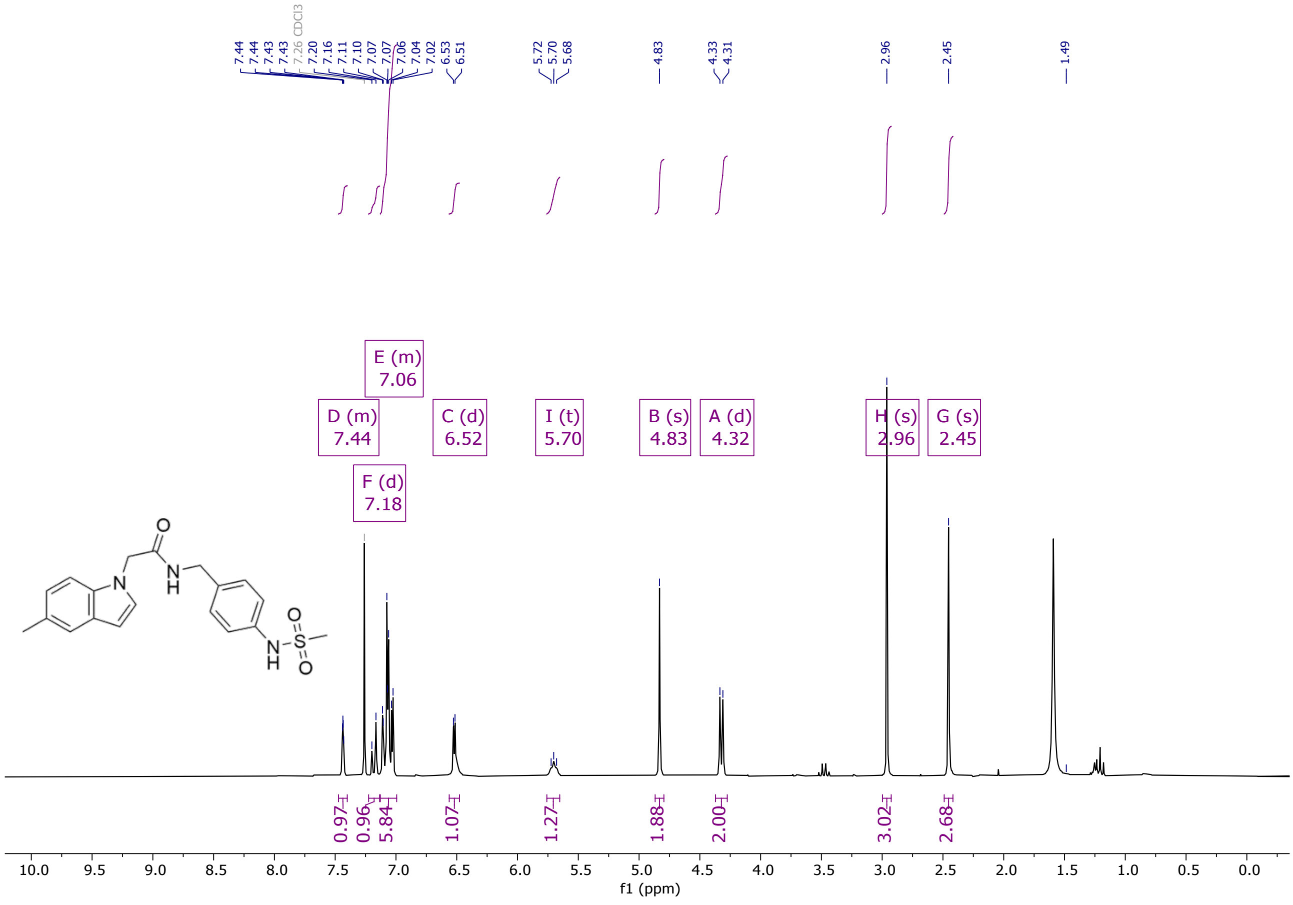

Figure S47: 2-(5-methyl-1H-indol-1-yl)-N-(4-(methylsulfonamido)benzyl)acetamide (33)

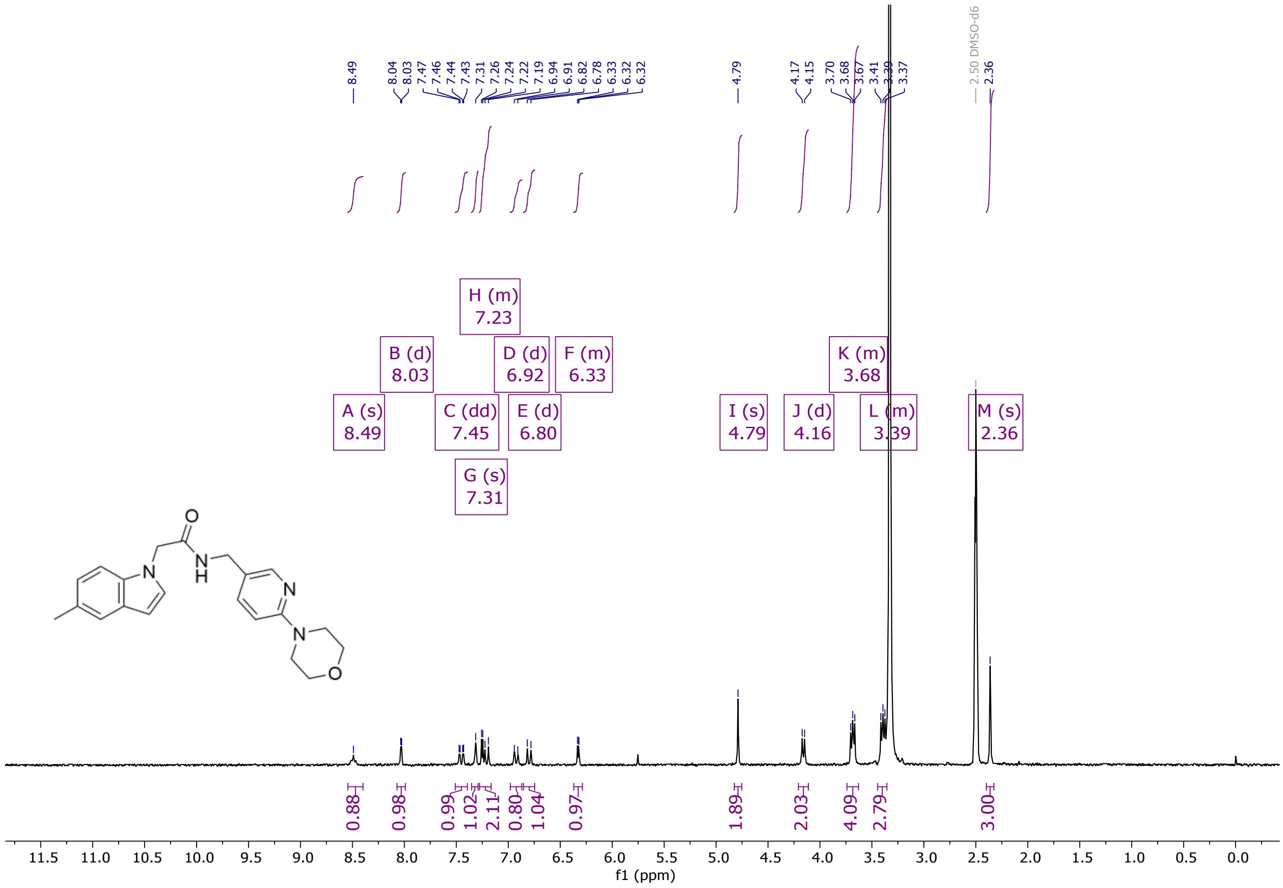

Figure S48: 2-(5-methyl-1H-indol-1-yl)-N-((6-morpholinopyridin-3-yl)methyl)acetamide (34)

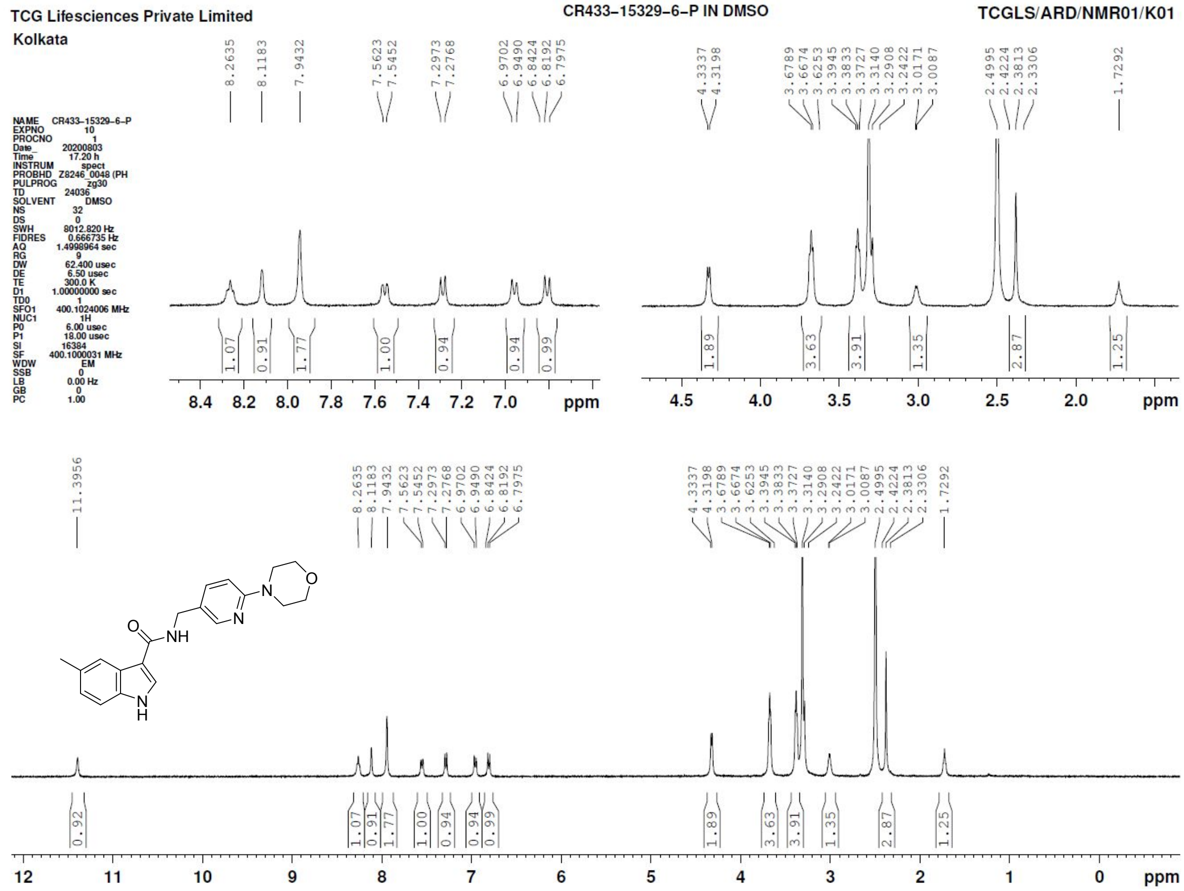

Figure S49: 5-methyl-N-((6-morpholinopyridin-3-yl)methyl)-1H-indole-3-carboxamide (35)

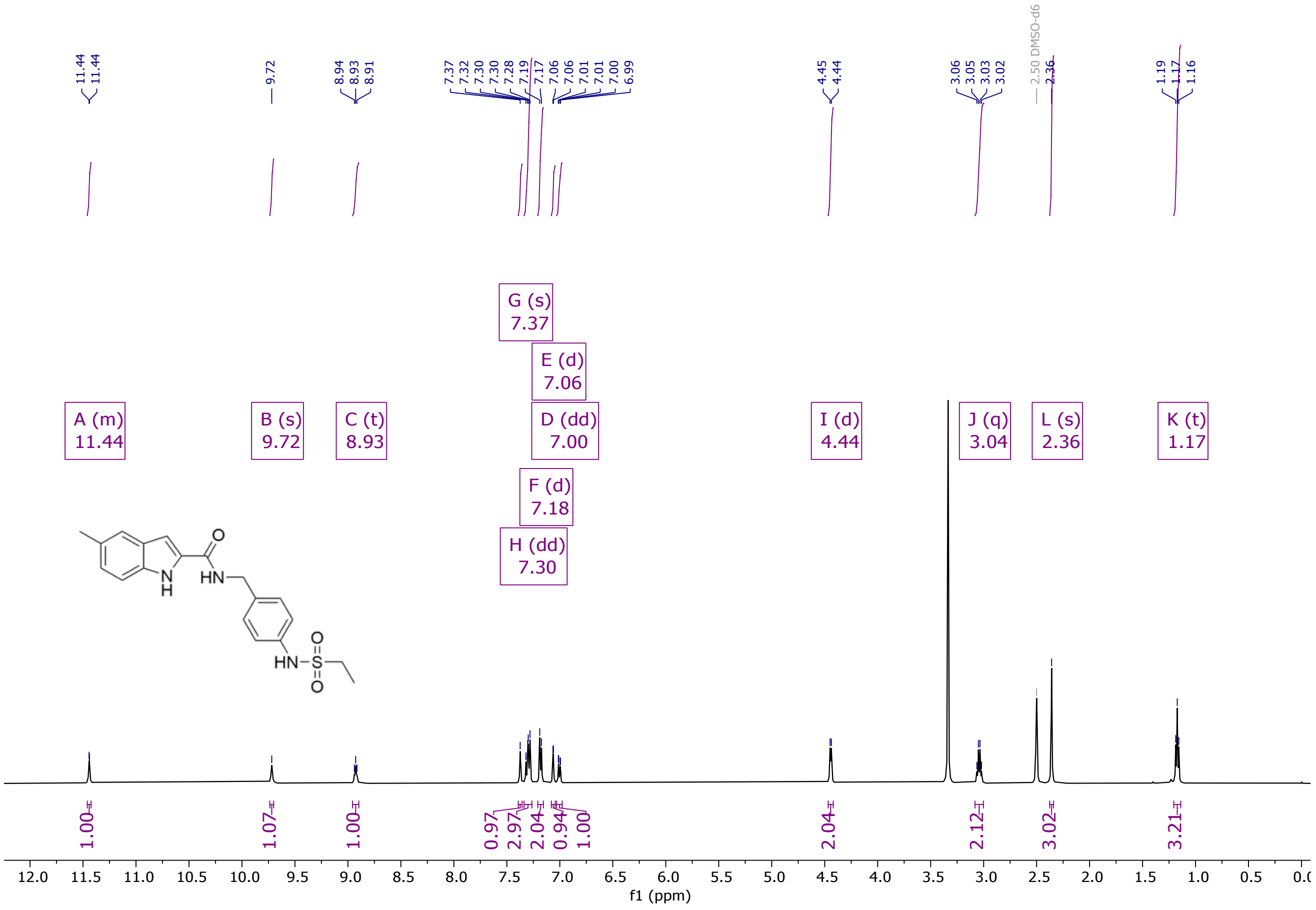

Figure S50: N-(4-(ethylsulfonamido)benzyl)-5-methyl-1H-indole-2-carboxamide (36)

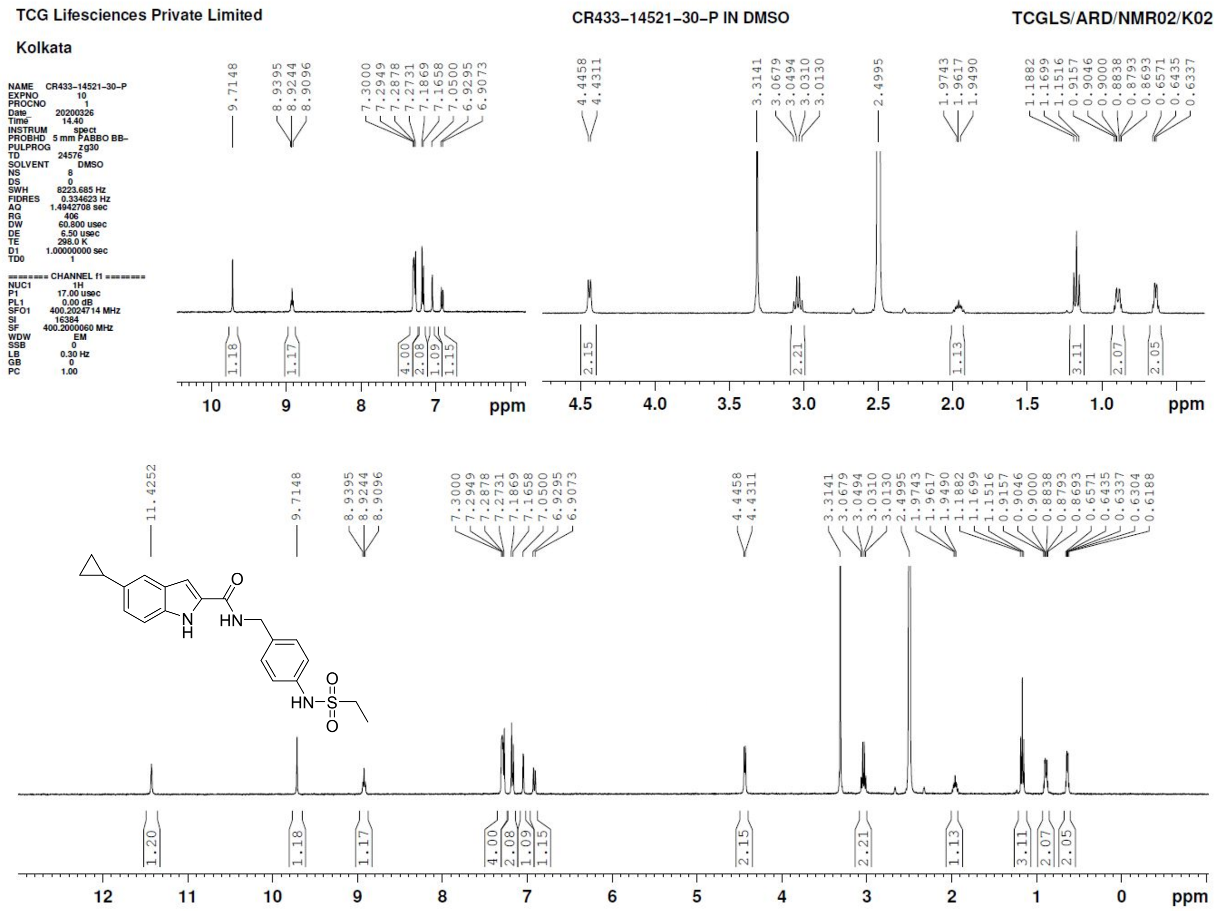

Figure S51: 5-cyclopropyl-N-(4-(ethylsulfonamido)benzyl)-1H-indole-2-carboxamide (37)

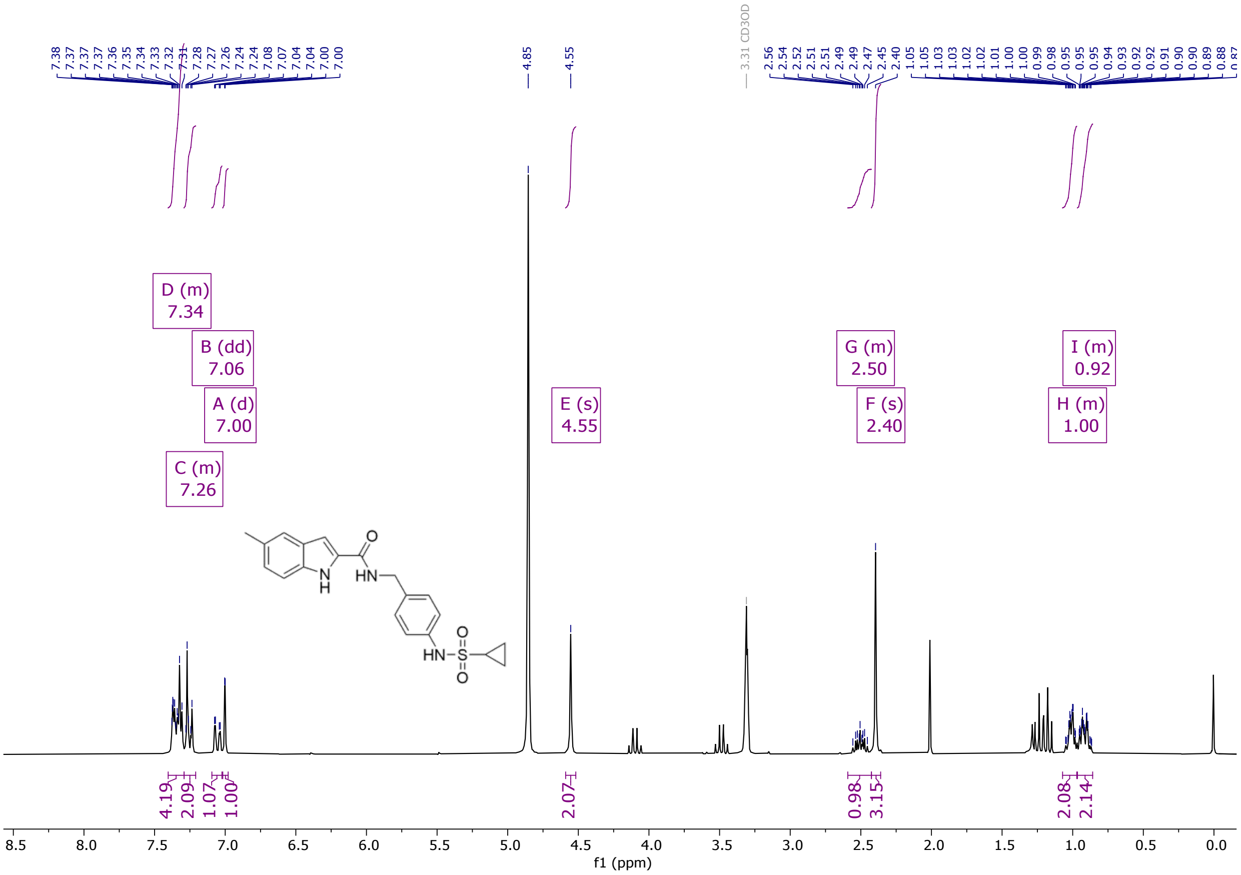

Figure S52: N-(4-(cyclopropanesulfonamido)benzyl)-5-methyl-1H-indole-2-carboxamide (38)

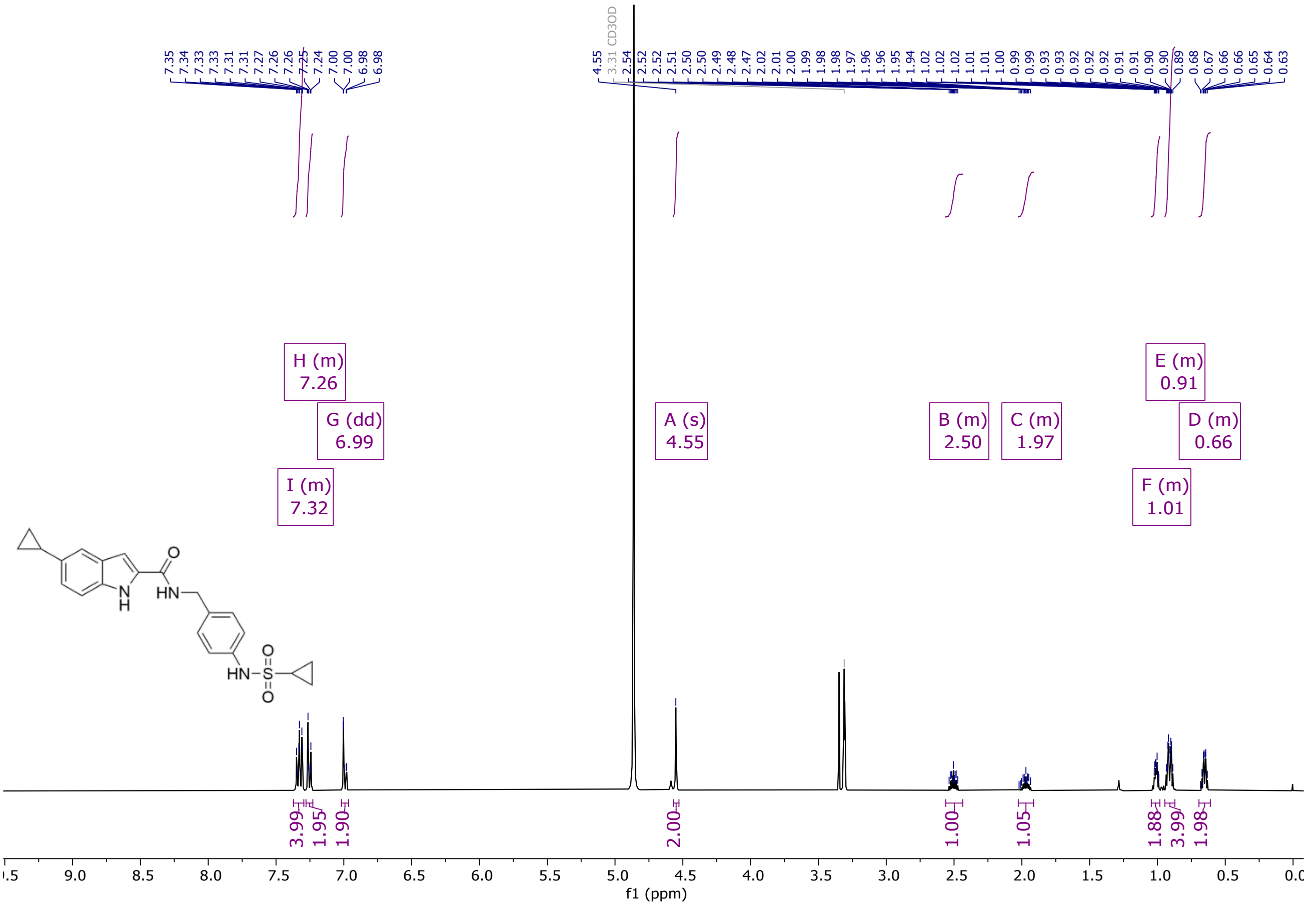

Figure S53:N-(4-(cyclopropanesulfonamido)benzyl)-5-cyclopropyl-1H-indole-2-carboxamide (39)

This report was created by ACD/NMR Processor Academic Edition. For more information go to www.acdlabs.com/nmrproc/

Acquisition Time (sec)	1.5903	*Comment*	Dessoy - MAD 1955 - Acetona - AV 500 MHz - set23madH3			*Date*	23 Sep 2019 12:24:32
File Name	\\nmrsparc.iqm.unicamp.br\espectros\avance500\2019\set19\Sala\Luiz Carlos\set23madH3\set23madH3_001000fid					*Frequency (MHz)*	499.87
Nucleus	1H	*Number of Transients*	16	*Original Points Count*	16384	*Points Count*	16384
Pulse Sequence	zg30	*Solvent*	Acetone	*Spectrum Offset (Hz)*	3078.1995	*Sweep Width (Hz)*	10302.20
Temperature (degree C)	25.147						

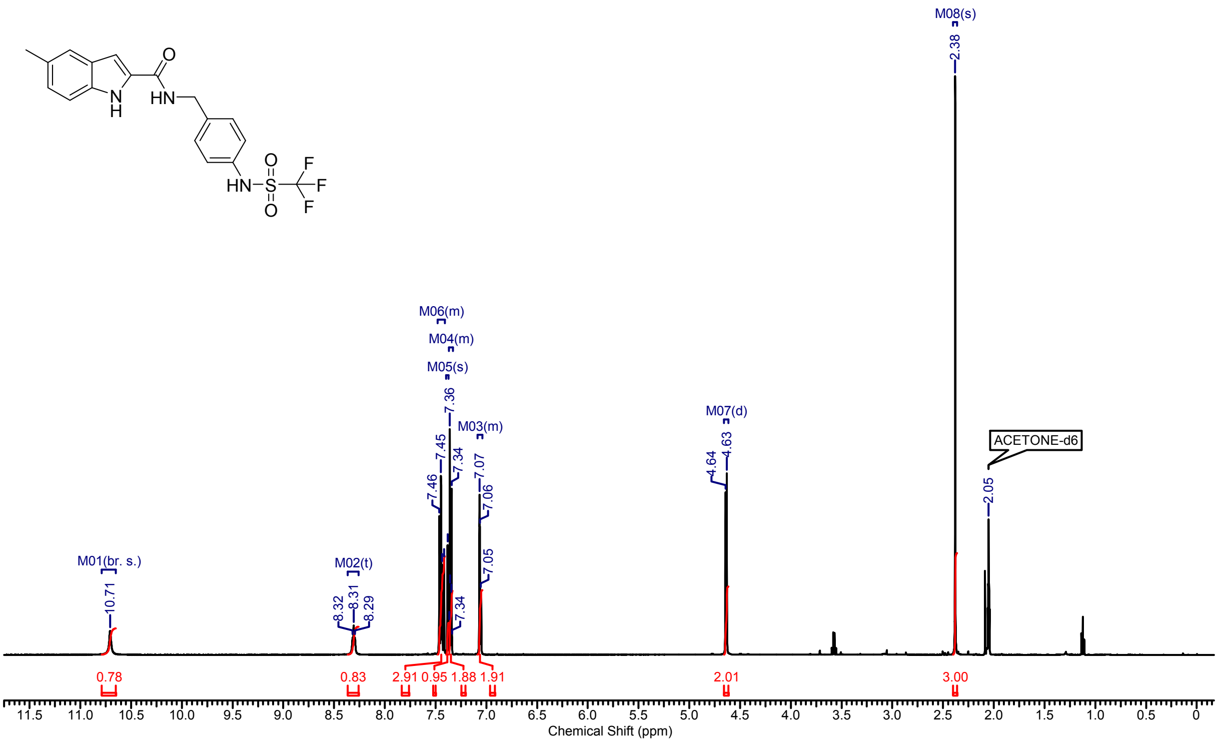

Figure S54: 5-methyl-N-(4-((trifluoromethyl)sulfonamido)benzyl)-1H-indole-2-carboxamide (40)

This report was created by ACD/NMR Processor Academic Edition. For more information go to www.acdlabs.com/nmrproc/

Acquisition Time (sec)	1.3282	*Comment*	Dessoy - MAD1957 - Acetona - Av 600 MHz - out01madH1			*Date*	01 Oct 2019 14:01:48
File Name	\\nmrsparc.iqm.unicamp.br\espectros\avance600\2019\out19\Sala\Luiz Carlos\out01madH1_001001r					*Frequency (MHz)*	600.17
Nucleus	1H	*Number of Transients*	16	*Original Points Count*	16384	*Points Count*	65536
Pulse Sequence	zg30	*Solvent*	Acetone	*Spectrum Offset (Hz)*	3695.8538	*Sweep Width (Hz)*	12335.53
Temperature (degree C)	25.150						

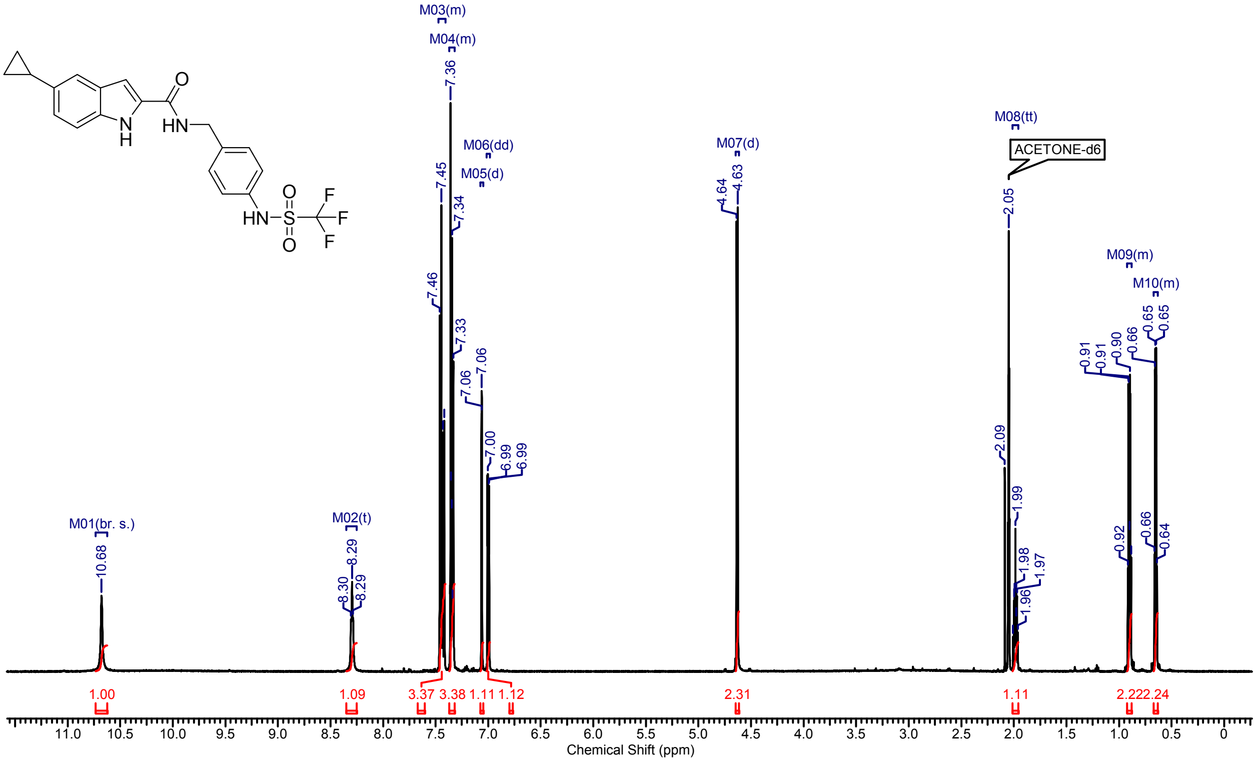

Figure S55: 5-cyclopropyl-N-(4-((trifluoromethyl)sulfonamido)benzyl)-1H-indole-2-carboxamide (41)

This report was created by ACD/NMR Processor Academic Edition. For more information go to www.acdlabs.com/nmrproc/

Acquisition Time (sec)	1.5903	***Comment***	Dessoy - MAD 1967 - Acetona - Avance 500 MHz - out11madH1- 1H			***Date***	11 Oct 2019 12:57:20
File Name	\\nmrsparc.iqm.unicamp.br\espectros\avance500\2019\out19\Sala\Luiz Carlos\out11madH1_001001r					***Frequency (MHz)***	499.87
Nucleus	1H	***Number of Transients***	16	***Original Points Count***	16384	***Points Count***	65536
Pulse Sequence	zg30	***Solvent***	Acetone	***Spectrum Offset (Hz)***	3078.3350	***Sweep Width (Hz)***	10302.20
Temperature (degree C)	25.151						

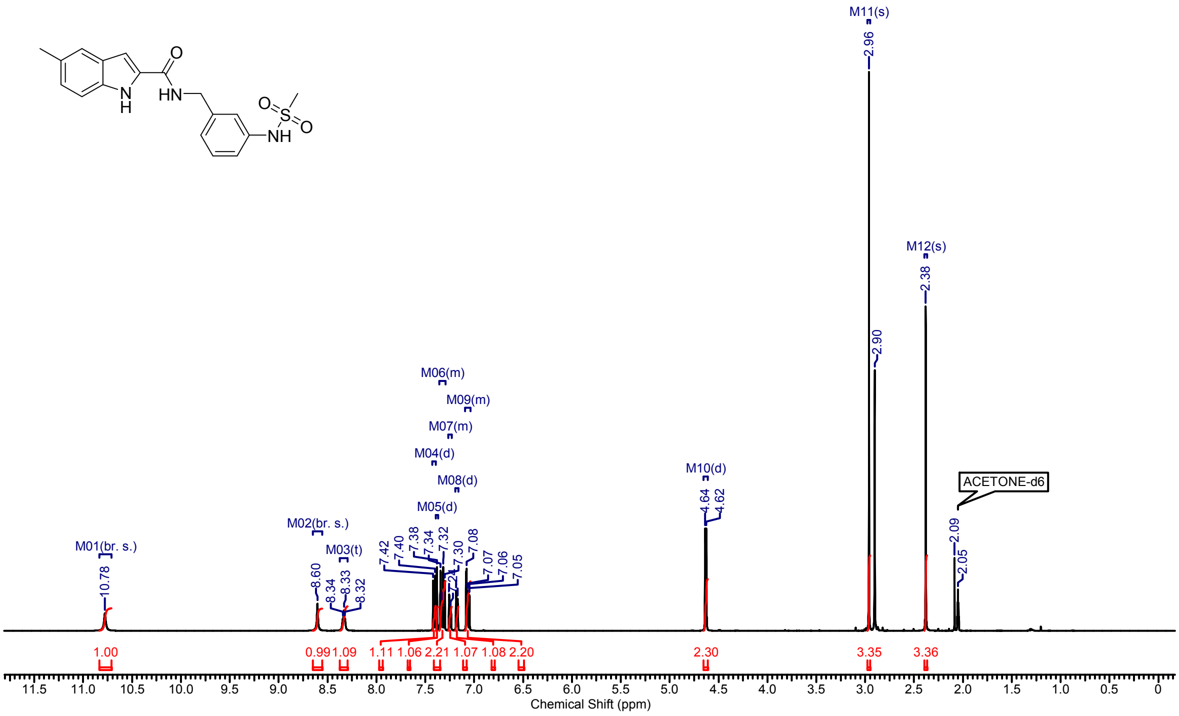

Figure S56: 5-methyl-N-(3-(methylsulfonamido)benzyl)-1H-indole-2-carboxamide (42)

This report was created by ACD/NMR Processor Academic Edition. For more information go to www.acdlabs.com/nmrproc/

Acquisition Time (sec)	1.5903	***Comment***	Dessoy - MAD 2153 - Acetona - Avance 500 MHz - ago23madH2 - 1H			***Date***	23 Aug 2021 18:00:04
File Name	\\nmrsparc.iqm.unicamp.br\espectros\avance500\2021\ago21\Sala\Luiz Carlos\ago23madH2_001001r					***Frequency (MHz)***	499.87
Nucleus	1H	***Number of Transients***	16	***Original Points Count***	16384	***Points Count***	65536
Pulse Sequence	zg30	***Solvent***	Acetone	***Spectrum Offset (Hz)***	3078.4922	***Sweep Width (Hz)***	10302.20
Temperature (degree C)	25.146						

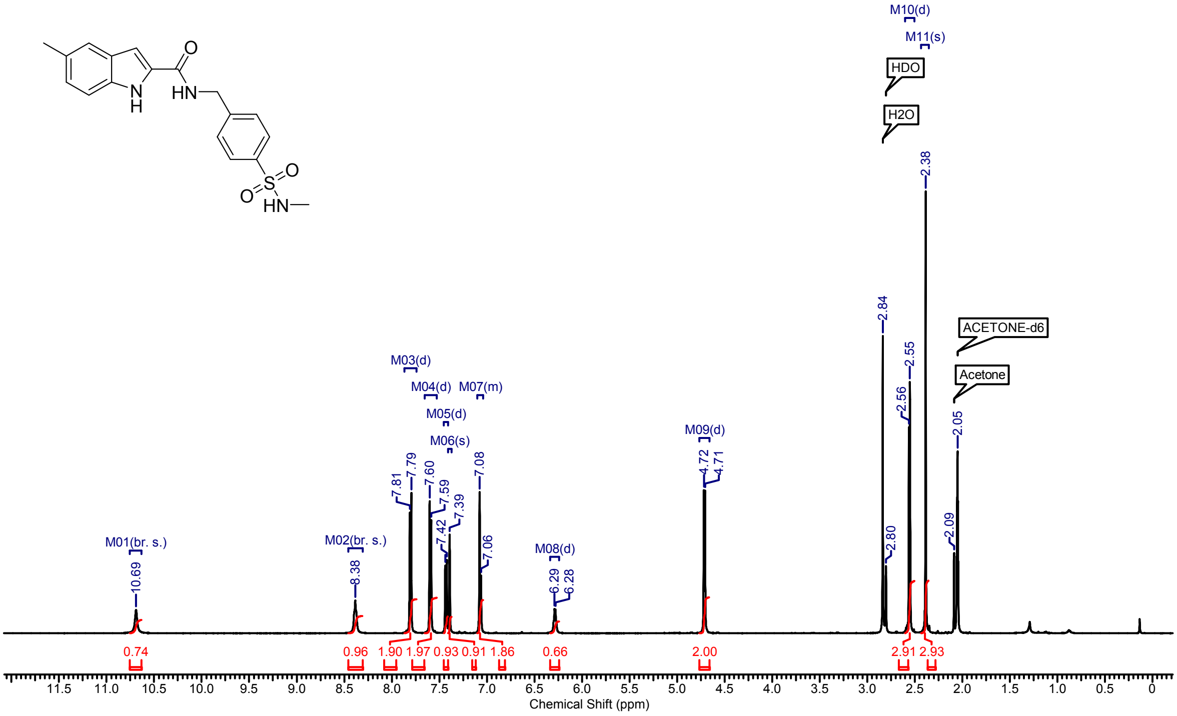

Figure S57: 5-methyl-N-(4-(N-methylsulfamoyl)benzyl)-1H-indole-2-carboxamide (43)

This report was created by ACD/NMR Processor Academic Edition. For more information go to www.acdlabs.com/nmrproc/

MDC-02-051

7/15/2022 7:04:13 PM

Acquisition Time (sec)	2.0494	***Date***	Feb 25 2022	***Date Stamp***	Feb 25 2022		
File Name	C:\Users\w540\Desktop\NMR_BOSTON\MDC-02-051_20220225_01\PROTON_03.fid\fid					***Frequency (MHz)***	499.66
Nucleus	1H	***Number of Transients***	64	***Original Points Count***	16384	***Points Count***	16384
Pulse Sequence	s2pul	***Receiver Gain***	60.00	***Solvent***	DMSO-d6	***Spectrum Offset (Hz)***	2497.1294
Spectrum Type	STANDARD	***Sweep Width (Hz)***	7994.40	***Temperature (degree C)***	25.000		

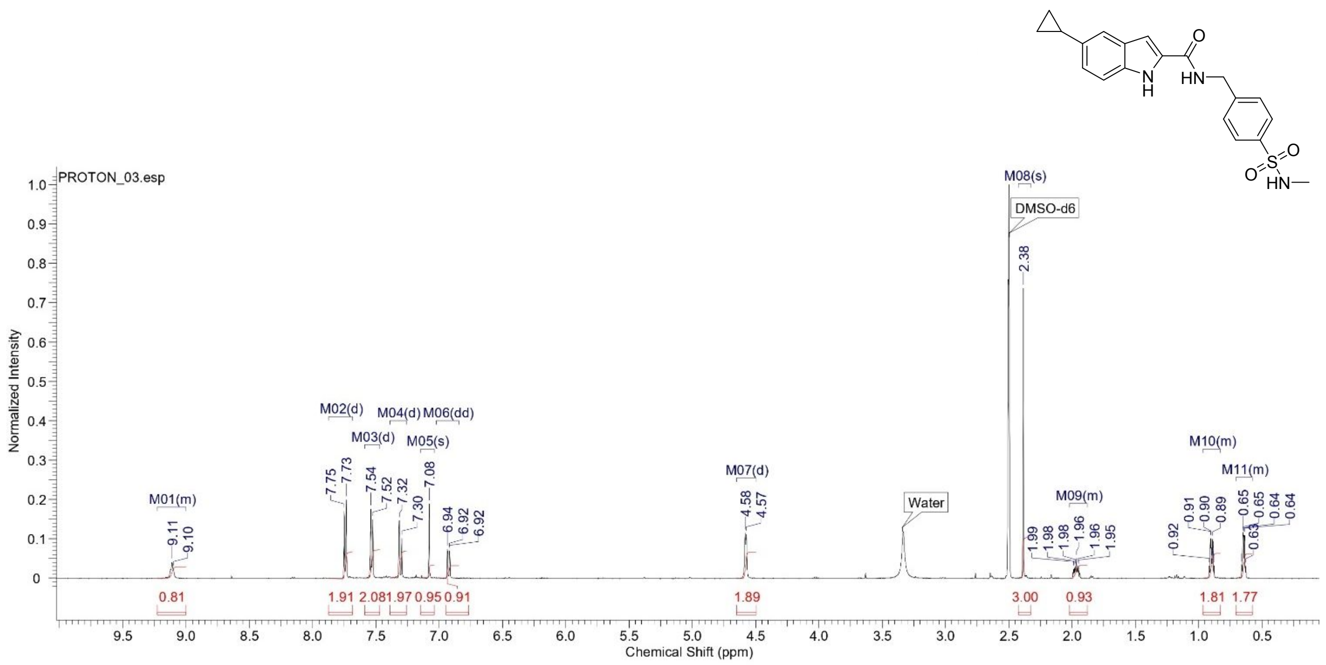

Figure S58: 5-cyclopropyl-N-(4-(N-methylsulfamoyl)benzyl)-1H-indole-2-carboxamide (44)

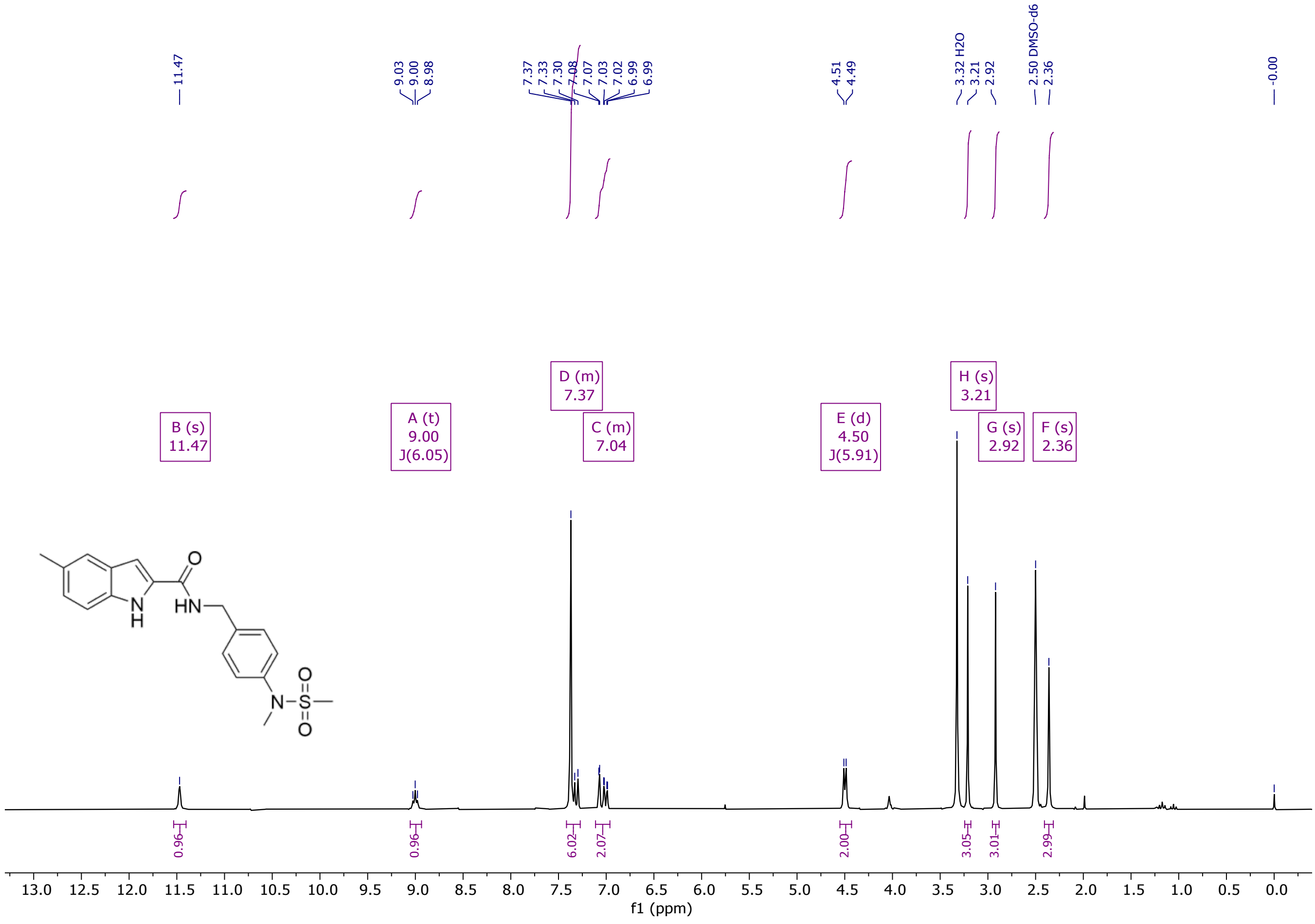

Figure S59: 5-methyl-N-(4-(N-methylmethylsulfonamido)benzyl)-1H-indole-2-carboxamide (45)

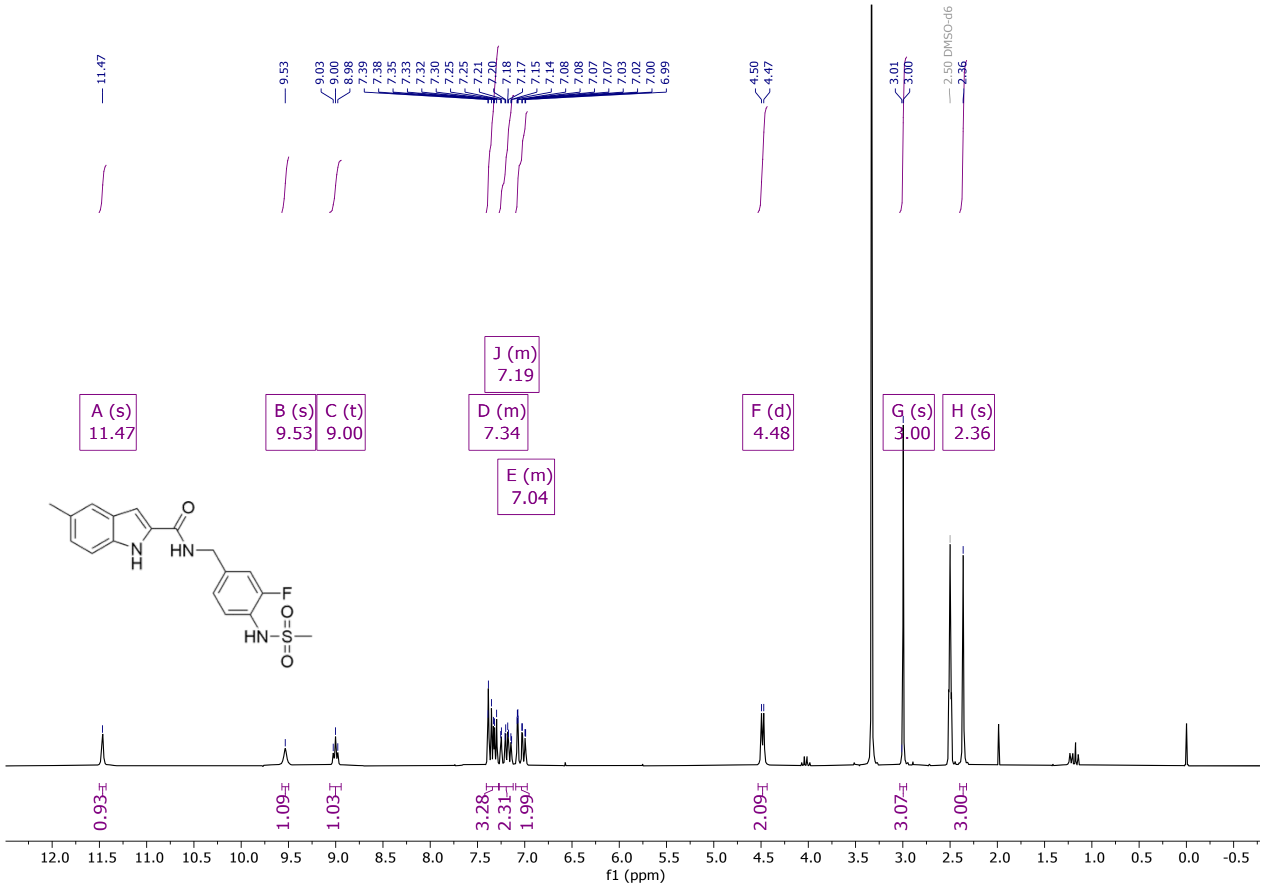

Figure S60: N-(3-fluoro-4-(methylsulfonamido)benzyl)-5-methyl-1H-indole-2-carboxamide (46)

This report was created by ACD/NMR Processor Academic Edition. For more information go to www.acdlabs.com/nmrproc/

MDC-02-015

7/14/2022 6:40:47 PM

Acquisition Time (sec)	2.0494	*Date*	Jan 29 2022	*Date Stamp*	Jan 29 2022		
File Name	C:\Users\w540\Desktop\NMR_BOSTON\MDC-02-015-sec_20220129_01\PROTON_03.fid\fid					*Frequency (MHz)*	499.66
Nucleus	1H	*Number of Transients*	128	*Original Points Count*	16384	*Points Count*	16384
Pulse Sequence	s2pul	*Receiver Gain*	60.00	*Solvent*	METHANOL-d4	*Spectrum Offset (Hz)*	2491.4695
Spectrum Type	STANDARD	*Sweep Width (Hz)*	7994.40	*Temperature (degree C)*	25.000		

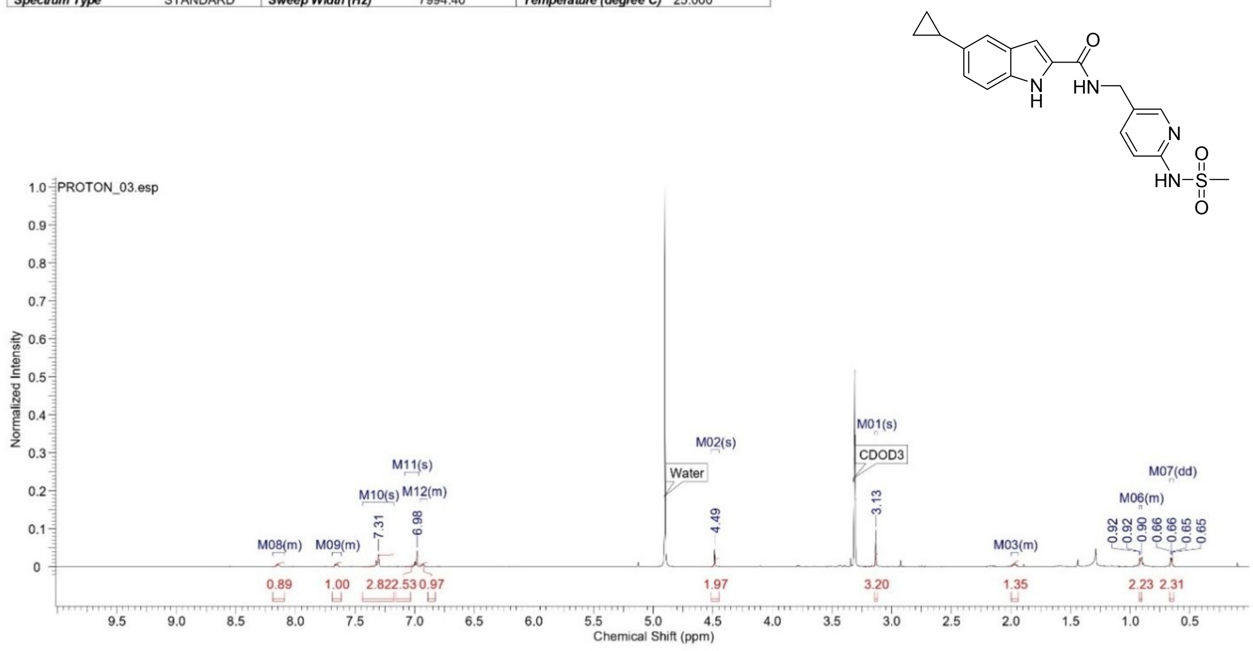

Figure S61: 5-cyclopropyl-N-((6-(methylsulfonamido)pyridin-3-yl)methyl)-1H-indole-2-carboxamide (47)

This report was created by ACD/NMR Processor Academic Edition. For more information go to www.acdlabs.com/nmrproc/

MDC-02-067

Acquisition Time (sec)	2.0494	Date	Apr 2 2022	Date Stamp	Apr 2 2022		
File Name	C:\Users\w540\Desktop\NMR_BOSTON\MDC-02-067-05_20220402_01\PROTON_07.fid\fid					Frequency (MHz)	499.66
Nucleus	1H	Number of Transients	256	Original Points Count	16384	Points Count	16384
Pulse Sequence	s2pul	Receiver Gain	60.00	Solvent	DMSO-d6	Spectrum Offset (Hz)	2500.5452
Spectrum Type	STANDARD	Sweep Width (Hz)	7994.40	Temperature (degree C)	25.000		

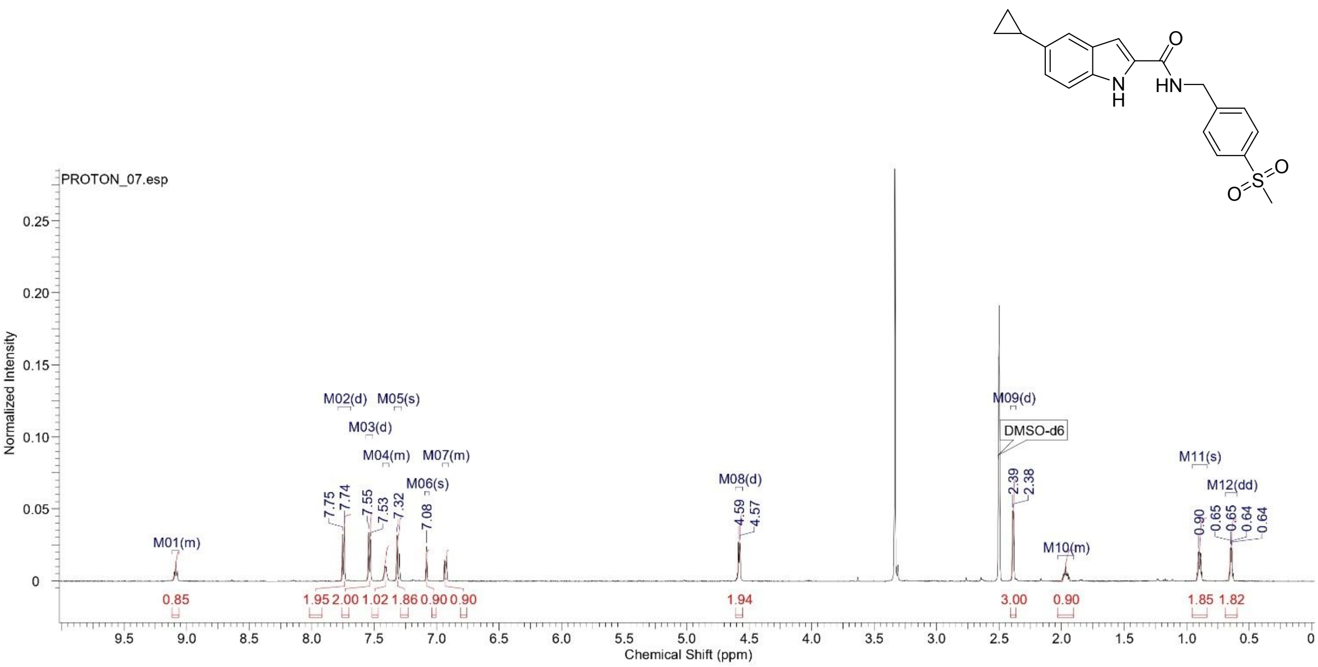

Figure S62: 5-cyclopropyl-N-(4-(methylsulfonyl)benzyl)-1H-indole-2-carboxamide (48)

This report was created by ACD/NMR Processor Academic Edition. For more information go to www.acdlabs.com/nmrproc/

MDC-02-079

7/16/2022 3:01:40 PM

Acquisition Time (sec)	2.0494	Date	Apr 11 2022	Date Stamp	Apr 11 2022				
File Name	C:\Users\w540\Desktop\NMR_BOSTON\NEU-70_20220411_01\PROTON_02.fid\fid					Frequency (MHz)	499.66	Nucleus	1H
Number of Transients	64	Original Points Count	16384	Points Count	16384	Pulse Sequence	s2pul	Receiver Gain	60.00
Solvent	DMSO-d6	Spectrum Offset (Hz)	2500.5452	Spectrum Type	STANDARD	Sweep Width (Hz)	7994.40	Temperature (degree C)	25.000

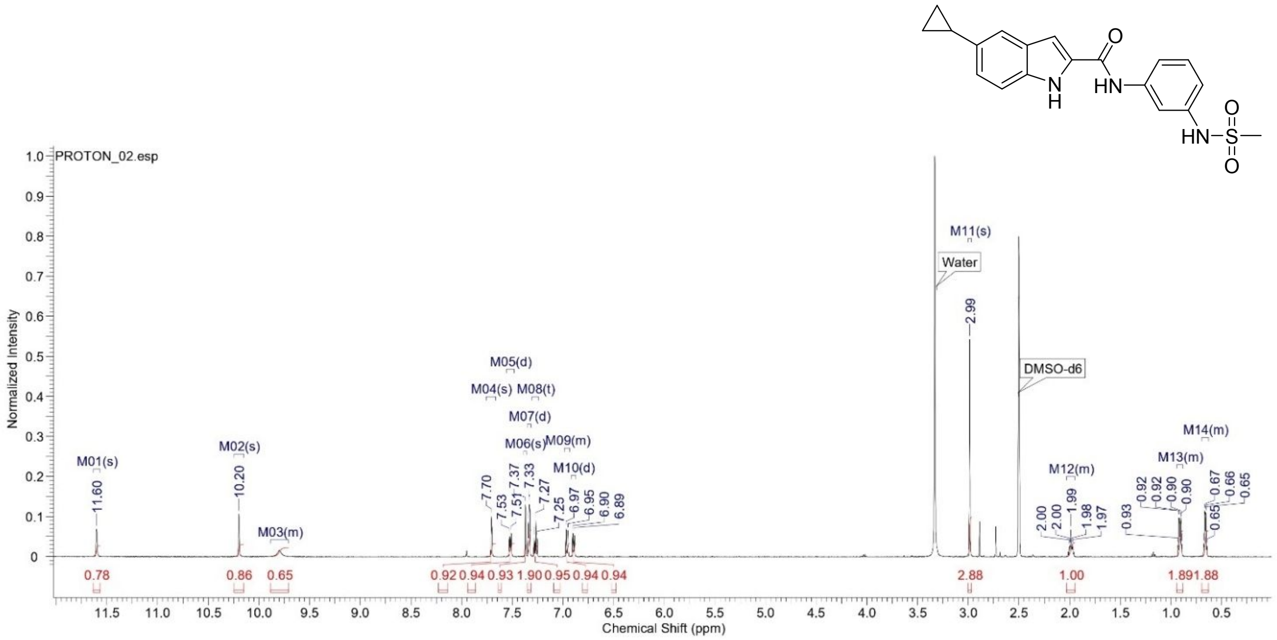

Figure S63: 5-cyclopropyl-N-(3-(methylsulfonamido)phenyl)-1H-indole-2-carboxamide (49)

This report was created by ACD/NMR Processor Academic Edition. For more information go to www.acdlabs.com/nmrproc/

MDC-02-078

7/16/2022 2:40:51 PM

Acquisition Time (sec)	2.0494	*Date*	Apr 15 2022	*Date Stamp*	Apr 15 2022		
File Name	C:\Users\w540\Desktop\NMR_BOSTON\MDC-02-078_20220415_01\PROTON_02.fid\fid					*Frequency (MHz)*	499.66
Nucleus	1H	*Number of Transients*	64	*Original Points Count*	16384	*Points Count*	16384
Pulse Sequence	s2pul	*Receiver Gain*	60.00	*Solvent*	DMSO-d6	*Spectrum Offset (Hz)*	2500.5452
Spectrum Type	STANDARD	*Sweep Width (Hz)*	7994.40	*Temperature (degree C)*	25.000		

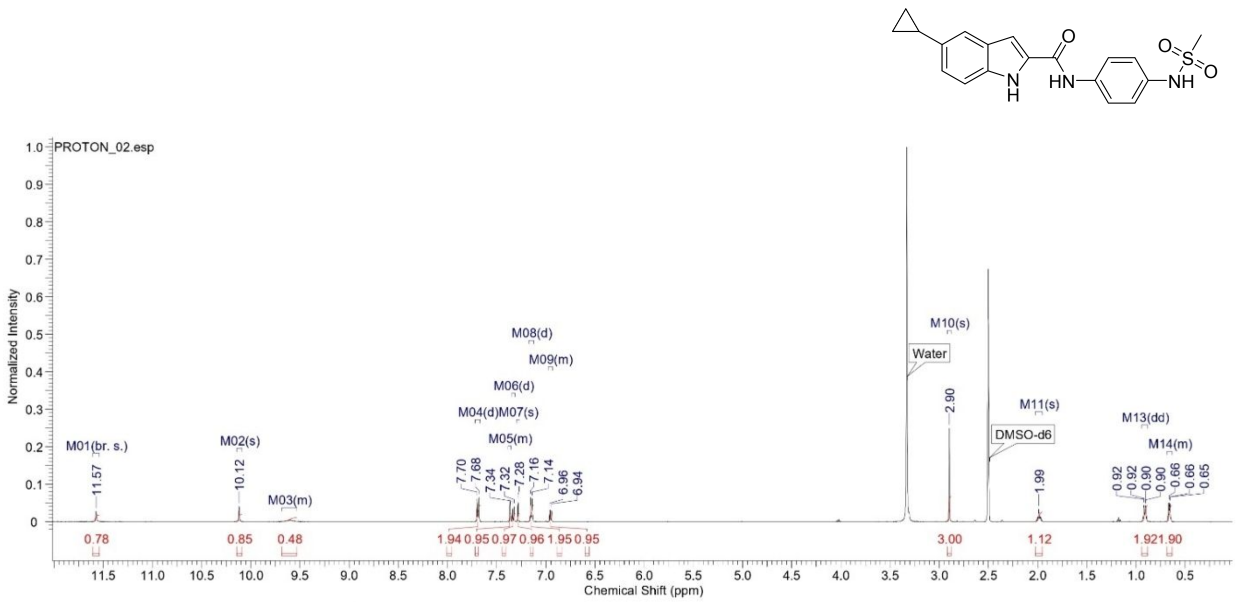

Figure S64: 5-cyclopropyl-N-(4-(methylsulfonamido)phenyl)-1H-indole-2-carboxamide (50)

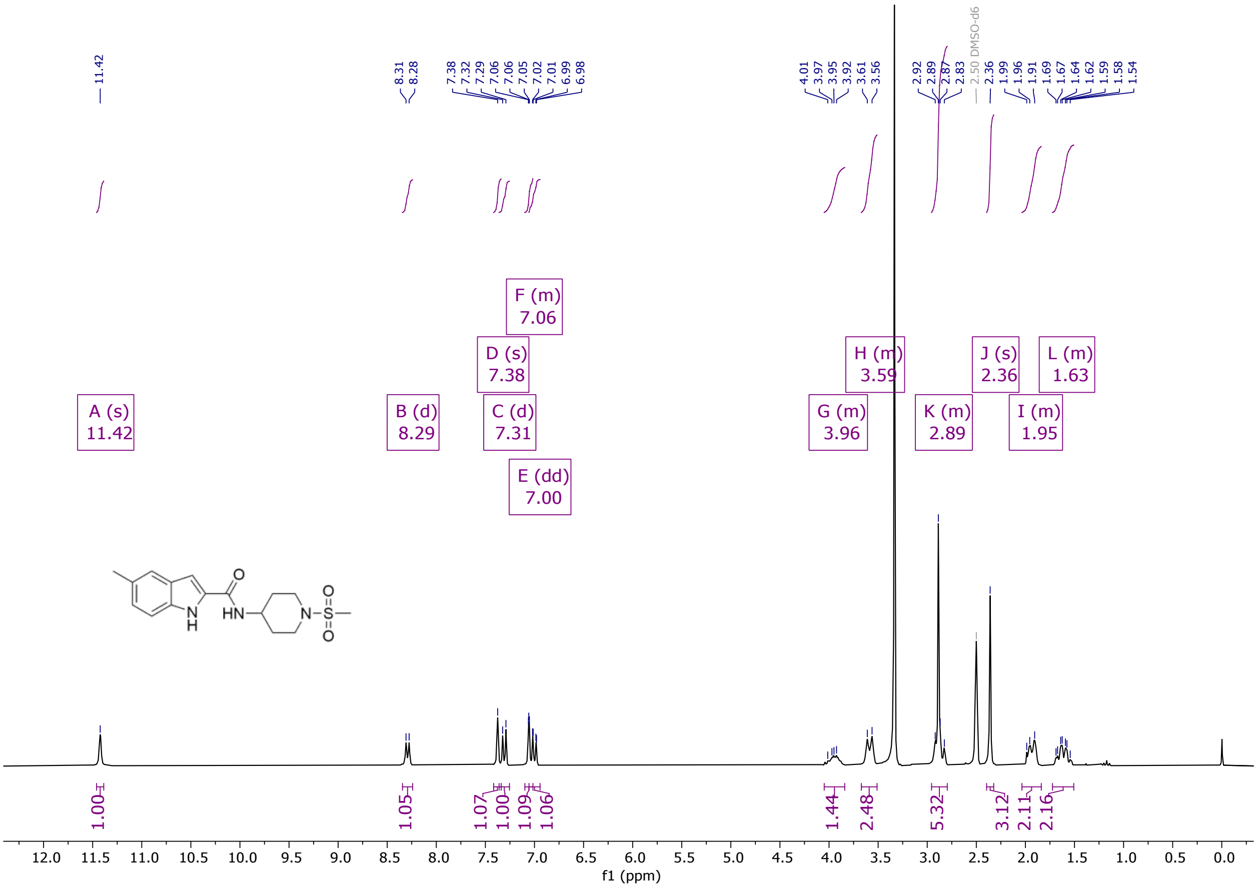

Figure S65: 5-methyl-N-(1-(methylsulfonyl)piperidin-4-yl)-1H-indole-2-carboxamide (51)

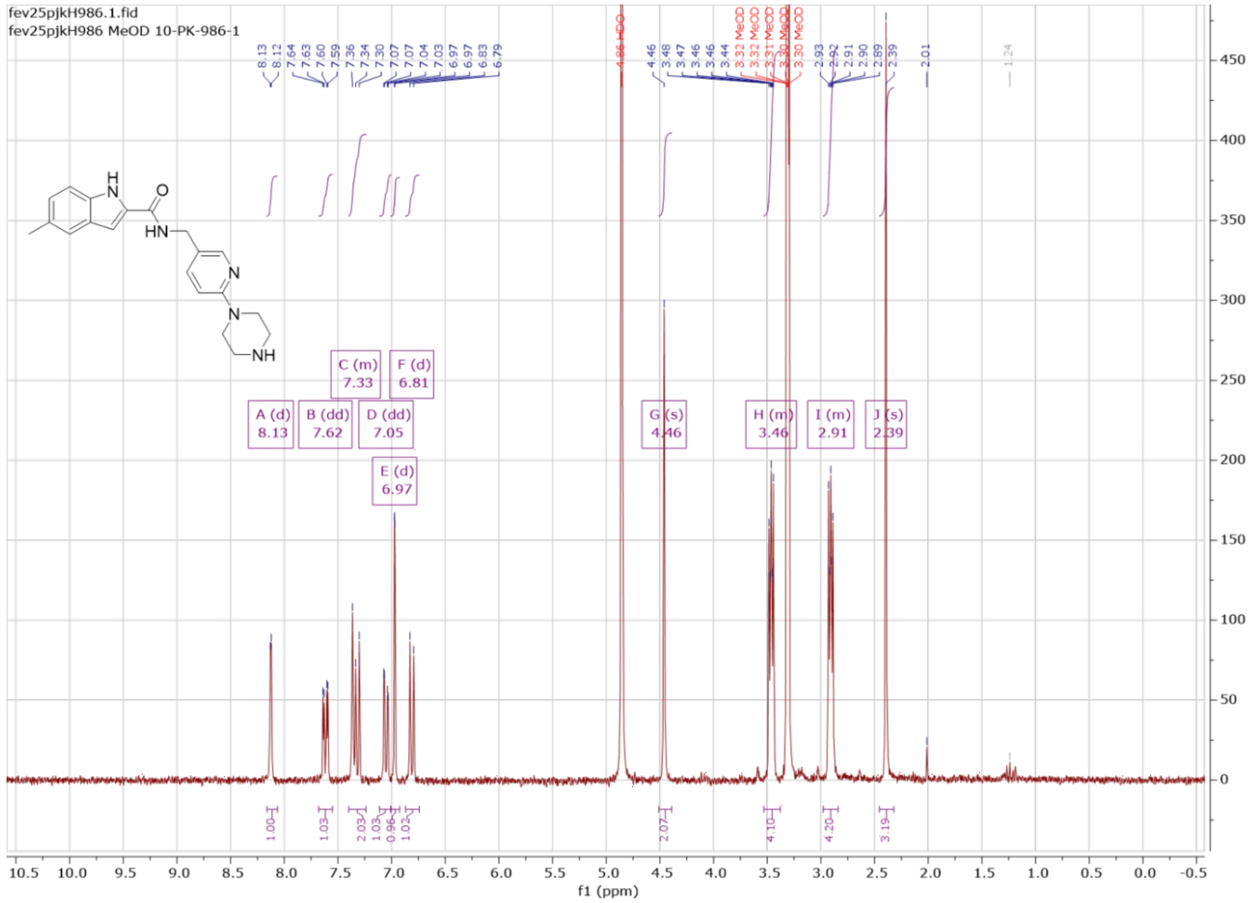

Figure S66: 5-methyl-N-((6-(piperazin-1-yl)pyridin-3-yl)methyl)-1H-indole-2-carboxamide (52)

This report was created by ACD/NMR Processor Academic Edition. For more information go to www.acdlabs.com/nmrproc/

QJS-08-048

7/16/2022 1:10:11 PM

Acquisition Time (sec)	2.0494	***Date***	Jan 25 2022	***Date Stamp***	Jan 25 2022		
File Name	C:\Users\w540\Desktop\NMR_BOSTON\QJS-08-48_20220125_01\PROTON_02.fid\fid					***Frequency (MHz)***	499.66
Nucleus	1H	***Number of Transients***	32	***Original Points Count***	16384	***Points Count***	16384
Pulse Sequence	s2pul	***Receiver Gain***	54.00	***Solvent***	DMSO-d6	***Spectrum Offset (Hz)***	2497.1294
Spectrum Type	STANDARD	***Sweep Width (Hz)***	7994.40	***Temperature (degree C)***	25.000		

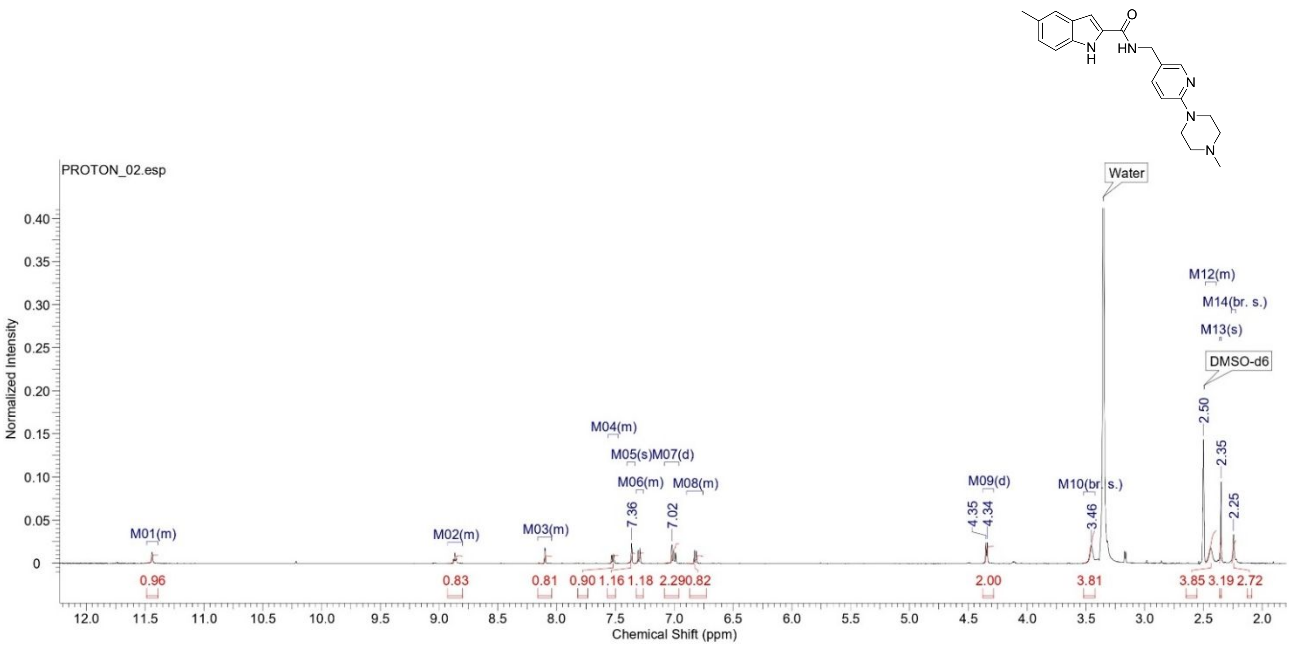

Figure S67: 5-methyl-N-((6-(4-methylpiperazin-1-yl)pyridin-3-yl)methyl)-1H-indole-2-carboxamide (53)

This report was created by ACD/NMR Processor Academic Edition. For more information go to www.acdlabs.com/nmrproc/

Acquisition Time (sec)	2.0447	Comment	Dessoy - MAD 1995 - CDCl3 - AV 400 MHz - jan14madH2		
Date	14 Jan 2020 20:03:12	File Name	\\nmrsparc.iqm.unicamp.br\espectros\avance400\2020\jan20\Sala\Luiz Carlos\jan14madH2\jan14madH2_001000fid		
Frequency (MHz)	400.18	Nucleus	1H	Number of Transients	16
Original Points Count	16384	Points Count	16384	Pulse Sequence	zg30
Solvent	CHLOROFORM-d	Spectrum Offset (Hz)	2465.4741	Sweep Width (Hz)	8012.82
Temperature (degree C)	25.178				

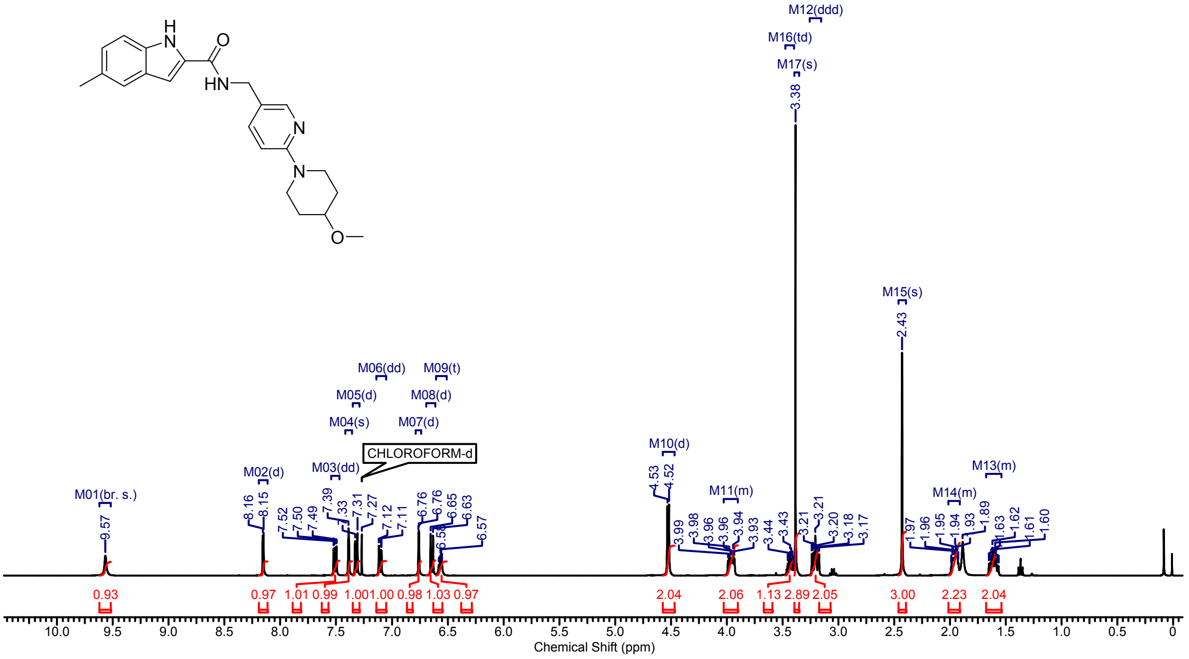

Figure S68: N-((6-(4-methoxypiperidin-1-yl)pyridin-3-yl)methyl)-5-methyl-1H-indole-2-carboxamide (54)

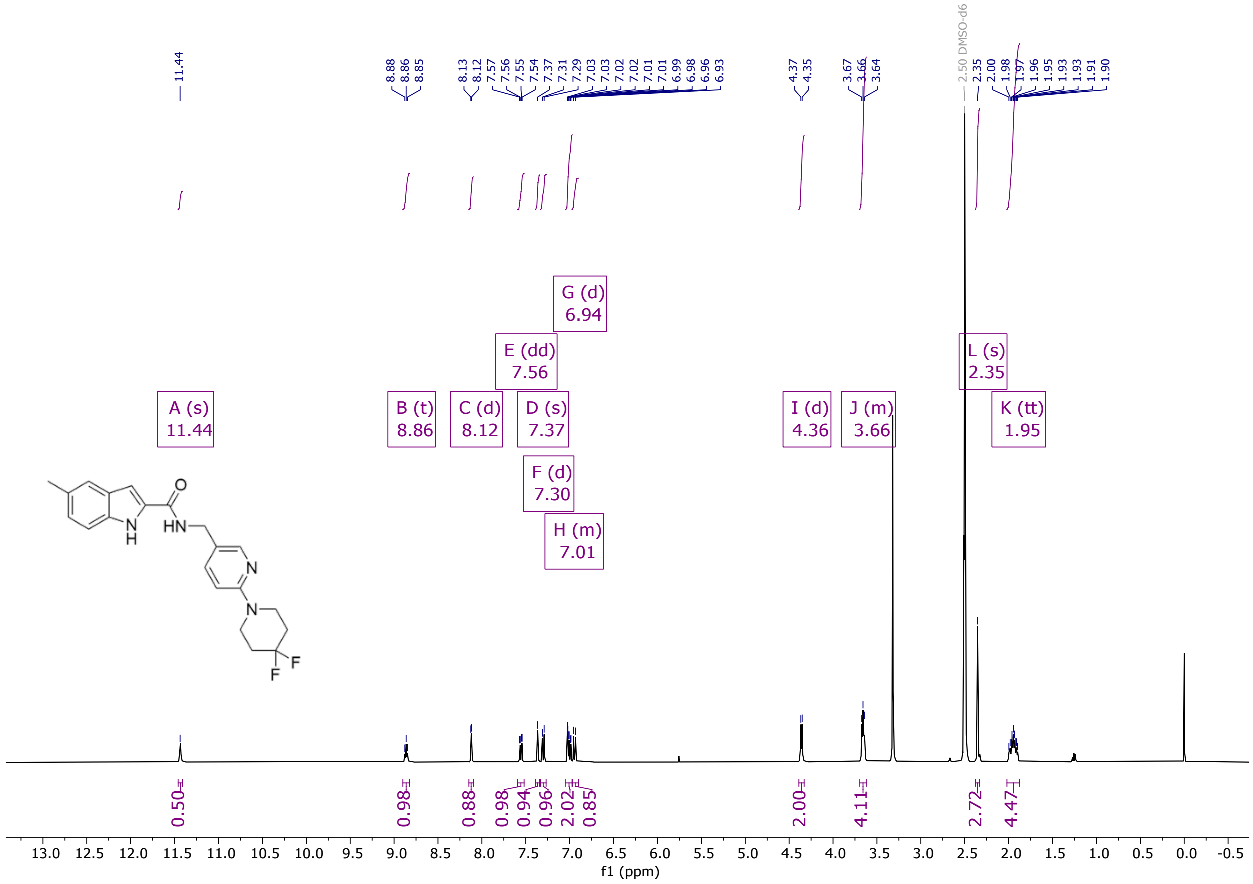

Figure S69: N-((6-(4,4-difluoropiperidin-1-yl)pyridin-3-yl)methyl)-5-methyl-1H-indole-2-carboxamide (55)

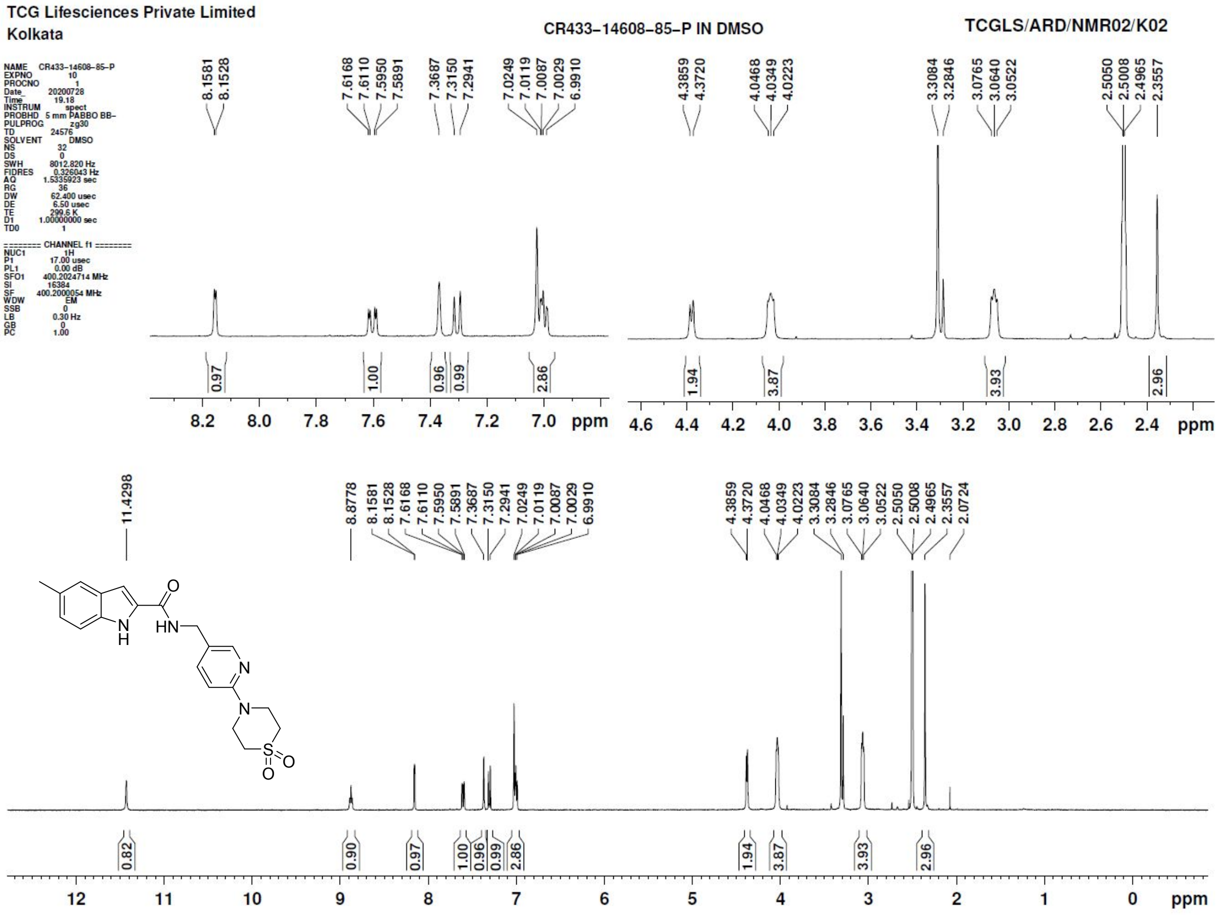

Figure S70: N-((6-(1,1-dioxidothiomorpholino)pyridin-3-yl)methyl)-5-methyl-1H-indole-2-carboxamide (56)

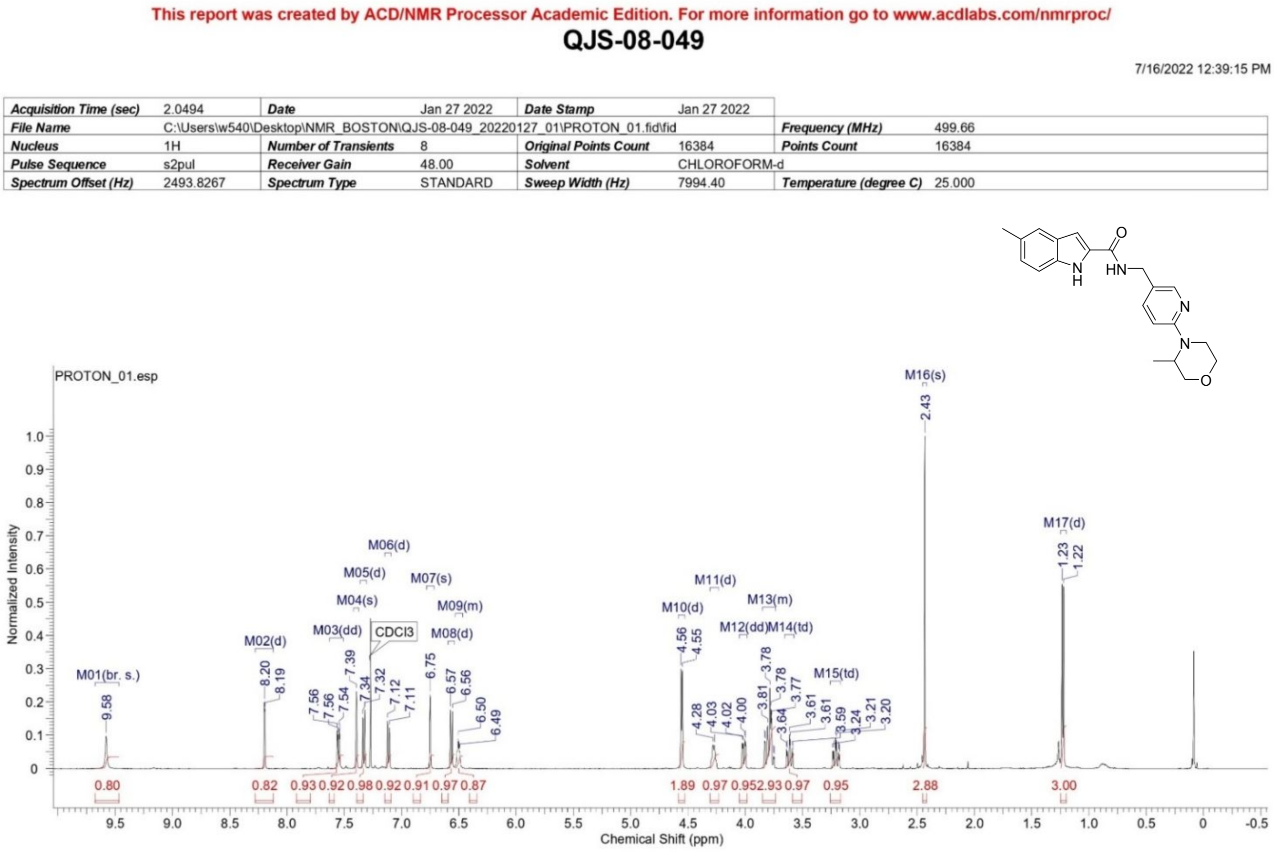

Figure S71: 5-methyl-N-((6-(3-methylmorpholino)pyridin-3-yl)methyl)-1H-indole-2-carboxamide (57)

This report was created by ACD/NMR Processor Academic Edition. For more information go to www.acdlabs.com/nmrproc/

QJS-08-051

7/16/2022 11:13:11 AM

Acquisition Time (sec)	2.0494	Date	Jan 27 2022	Date Stamp	Jan 27 2022		
File Name	C:\Users\w540\Desktop\NMR_BOSTON\QJS-08-051_20220127_01\PROTON_01.fid\fid					Frequency (MHz)	499.66
Nucleus	1H	Number of Transients	8	Original Points Count	16384	Points Count	16384
Pulse Sequence	s2pul	Receiver Gain	42.00	Solvent	CHLOROFORM-d		
Spectrum Offset (Hz)	2493.8267	Spectrum Type	STANDARD	Sweep Width (Hz)	7994.40	Temperature (degree C)	25.000

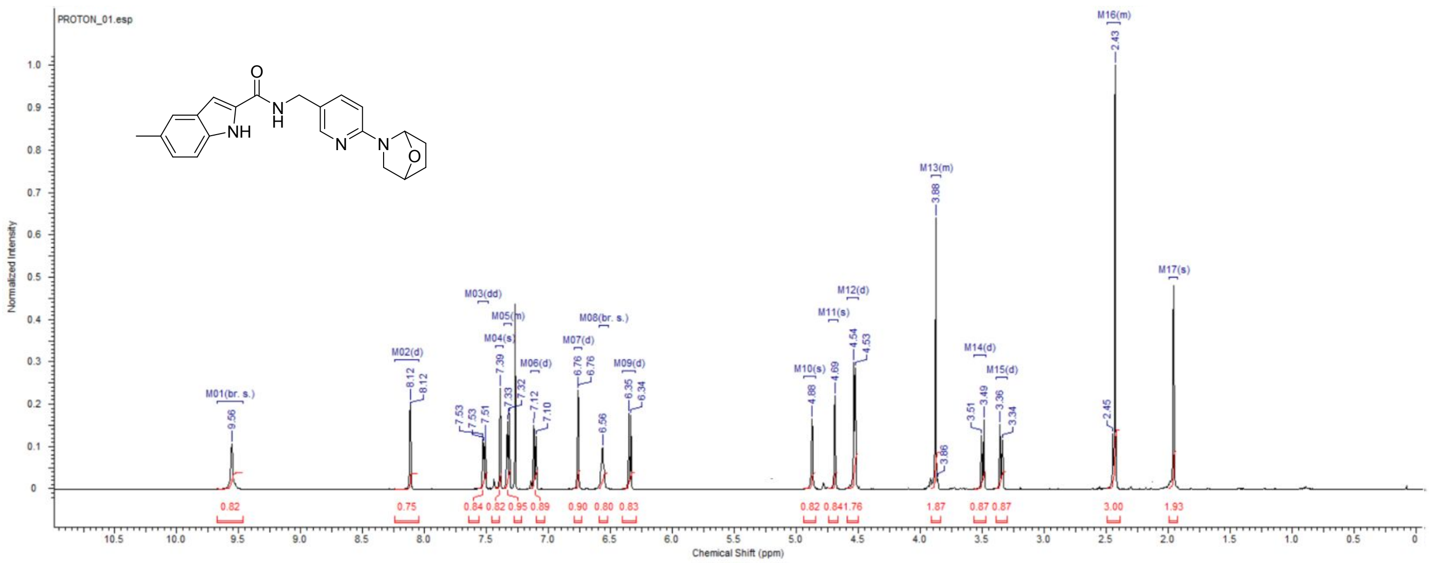

Figure S72: N-((6-(7-oxa-2-azabicyclo[2.2.1]heptan-2-yl)pyridin-3-yl)methyl)-5-methyl-1H-indole-2-carboxamide (58)

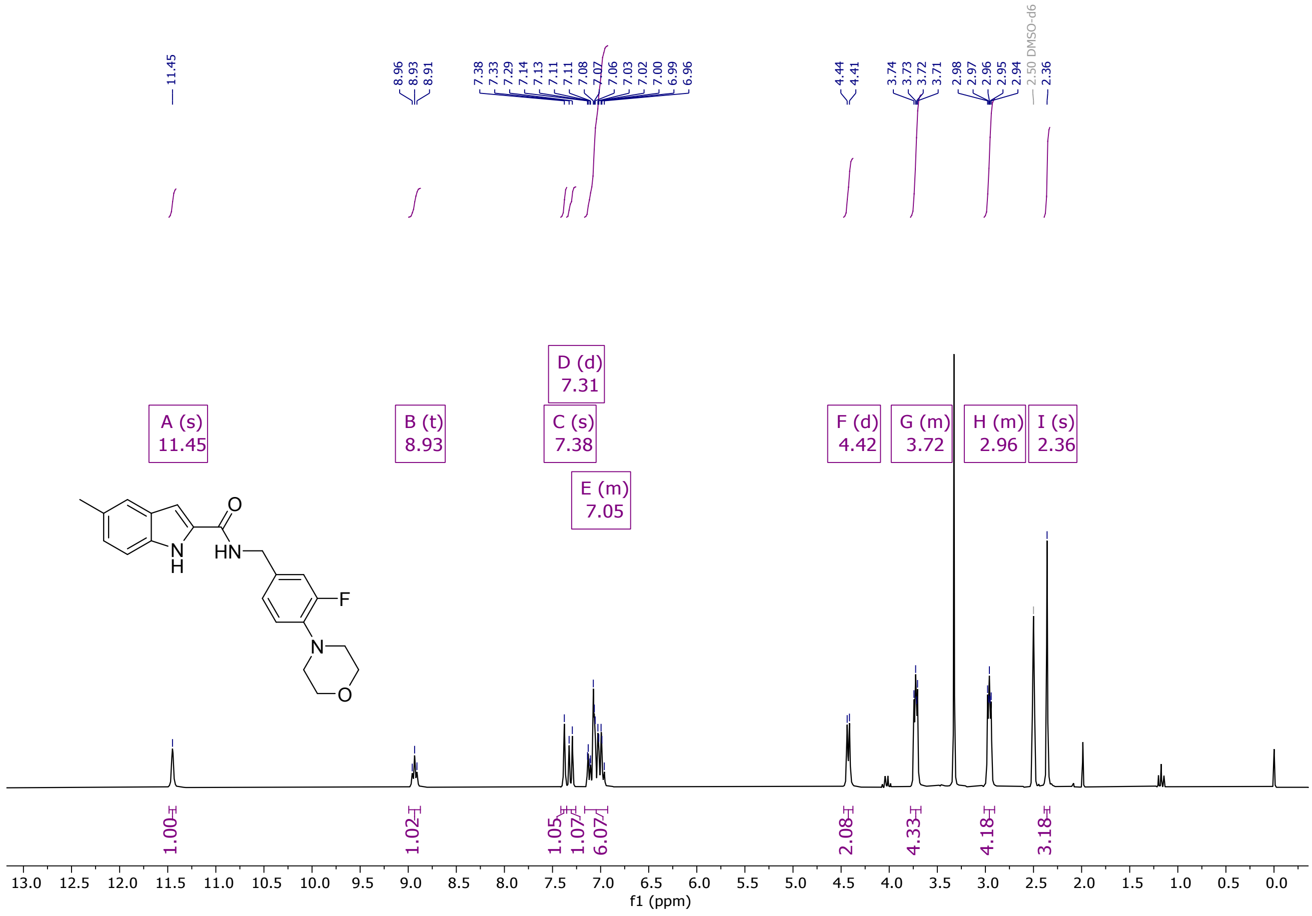

Figure S73: N-(3-fluoro-4-morpholinobenzyl)-5-methyl-1H-indole-2-carboxamide (59)

This report was created by ACD/NMR Processor Academic Edition. For more information go to www.acdlabs.com/nmrproc/

MDC-02-091

7/16/2022 2:32:12 PM

Acquisition Time (sec)	2.0494	Date	May 2 2022	Date Stamp	May 2 2022						
File Name	C:\Users\w540\Desktop\NMR_BOSTON\NEU-65_20220502_01\PROTON_01.fid\fid					Frequency (MHz)	499.66	Nucleus	1H		
Number of Transients	64	Original Points Count	16384	Points Count	16384	Pulse Sequence	s2pul	Receiver Gain	60.00		
Solvent	DMSO-d6	Spectrum Offset (Hz)	2500.5452	Spectrum Type	STANDARD	Sweep Width (Hz)	7994.40	Temperature (degree C)	25.000		

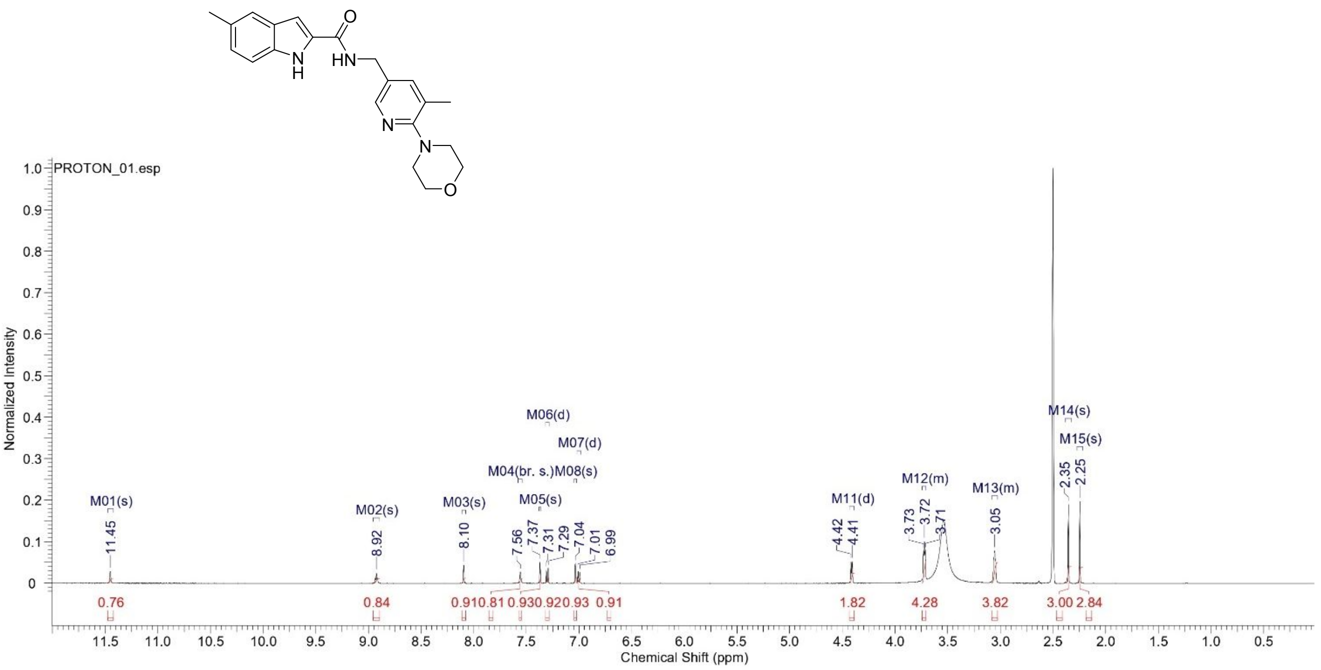

Figure S74: 5-methyl-N-((5-methyl-6-morpholinopyridin-3-yl)methyl)-1H-indole-2-carboxamide (60)

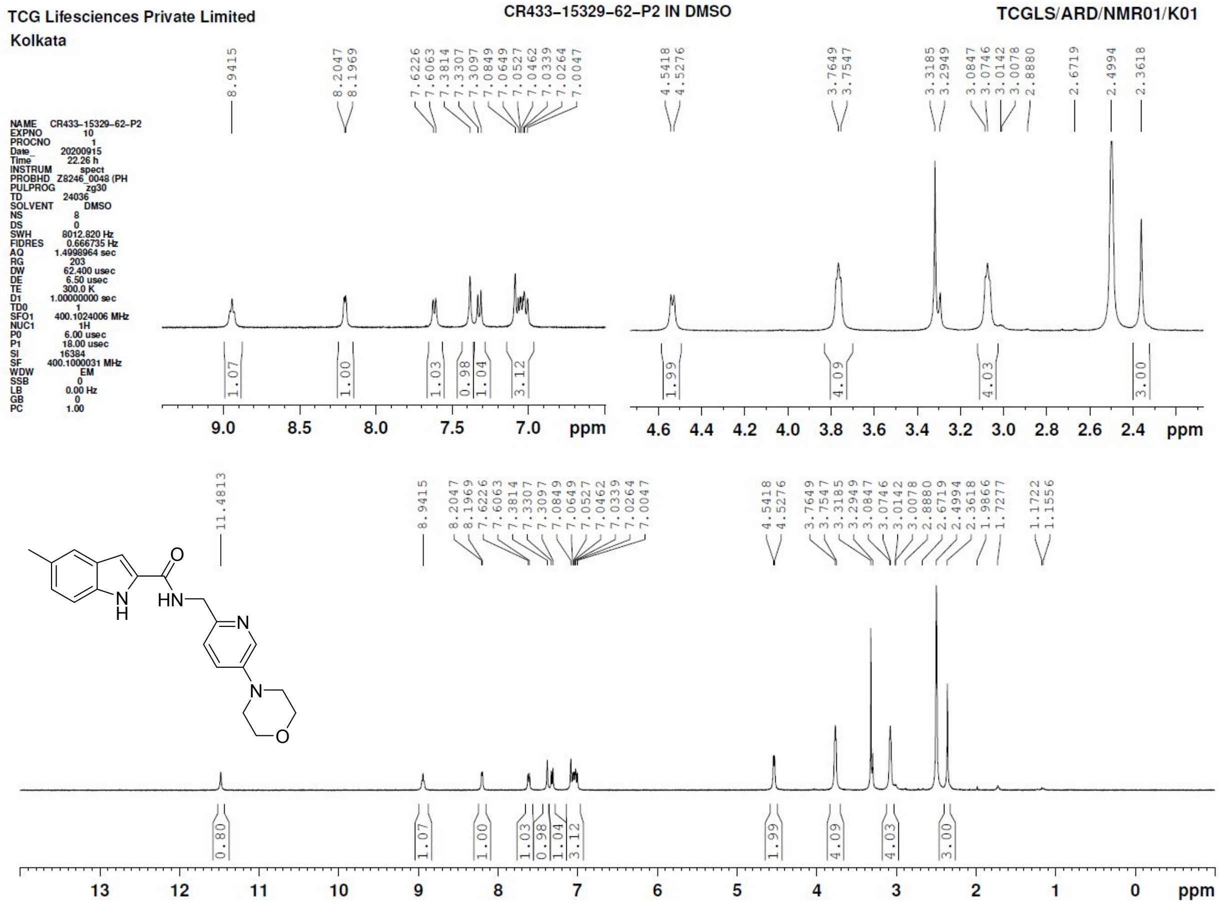

Figure S75: 5-methyl-N-((5-morpholinopyridin-2-yl)methyl)-1H-indole-2-carboxamide (61)

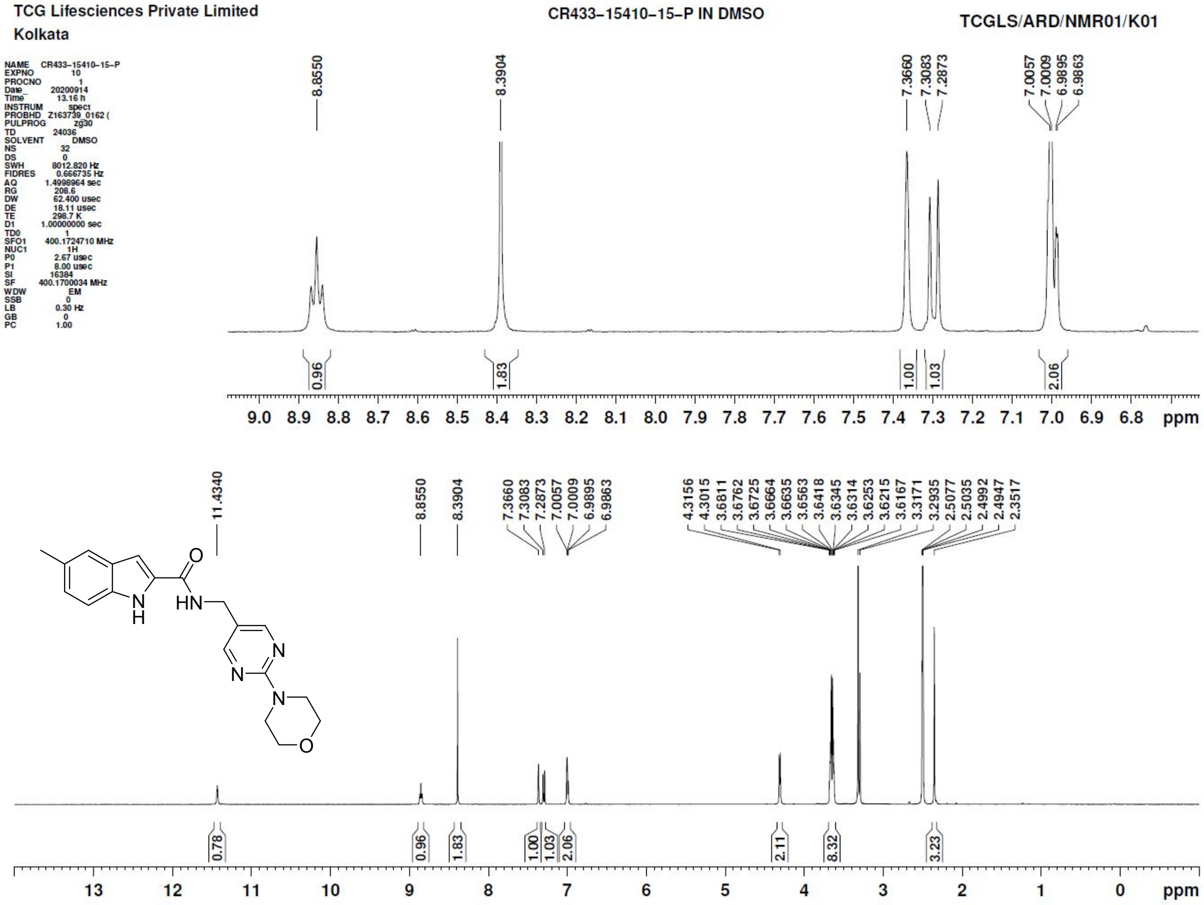

Figure S76: 5-methyl-N-((2-morpholinopyrimidin-5-yl)methyl)-1H-indole-2-carboxamide (62)

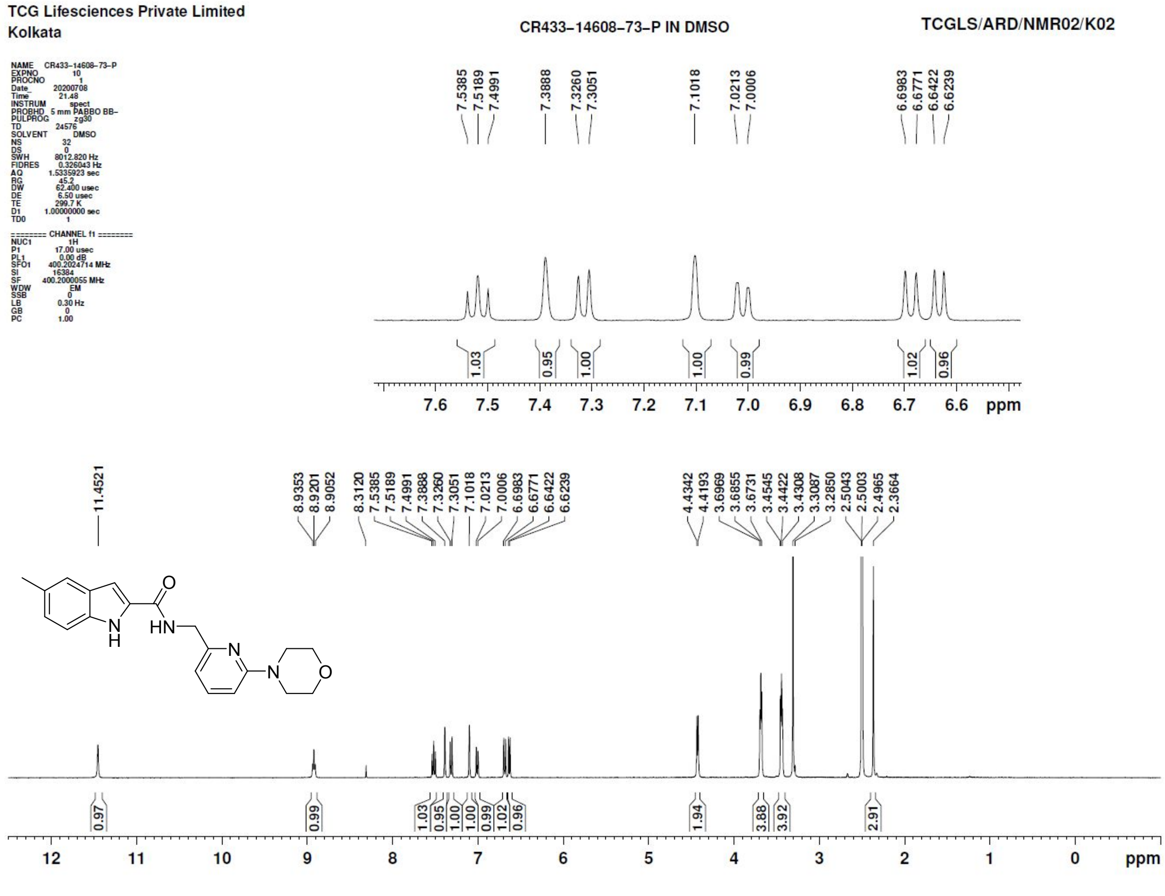

Figure S77: 5-methyl-N-((6-morpholinopyridin-2-yl)methyl)-1H-indole-2-carboxamide (63)

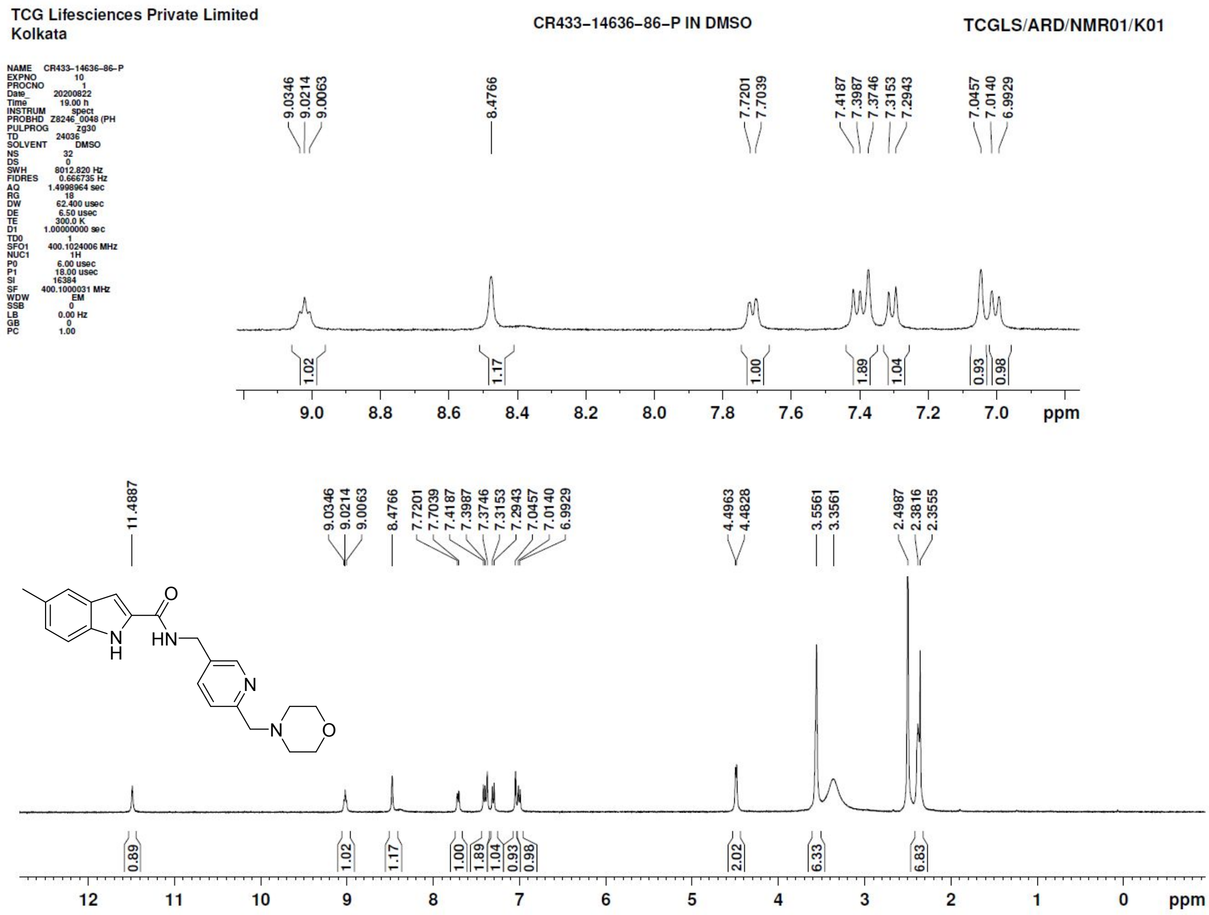

Figure S78: 5-methyl-N-((6-(morpholinomethyl)pyridin-3-yl)methyl)-1H-indole-2-carboxamide (64)

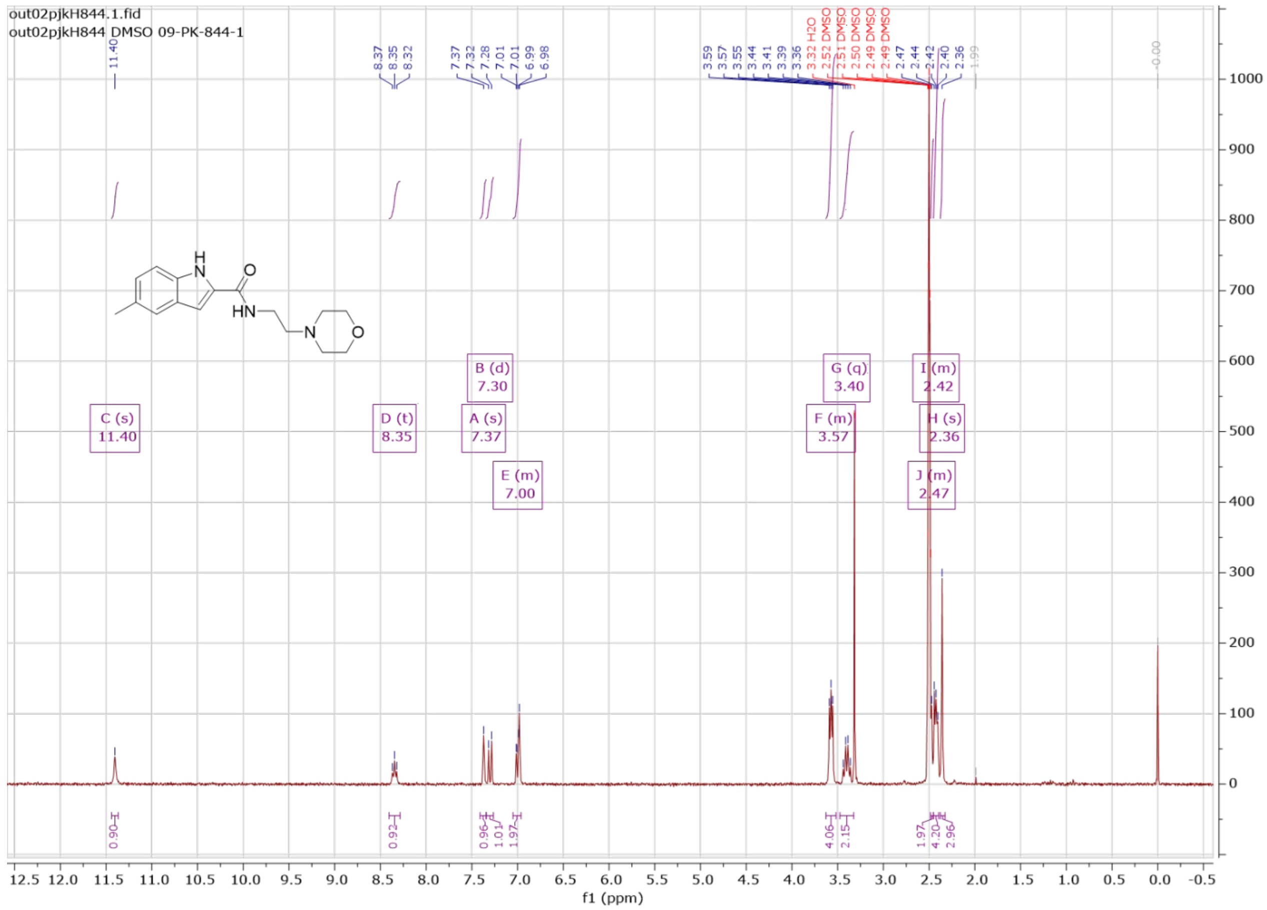

Figure S79: 5-methyl-N-(2-morpholinoethyl)-1H-indole-2-carboxamide (65)

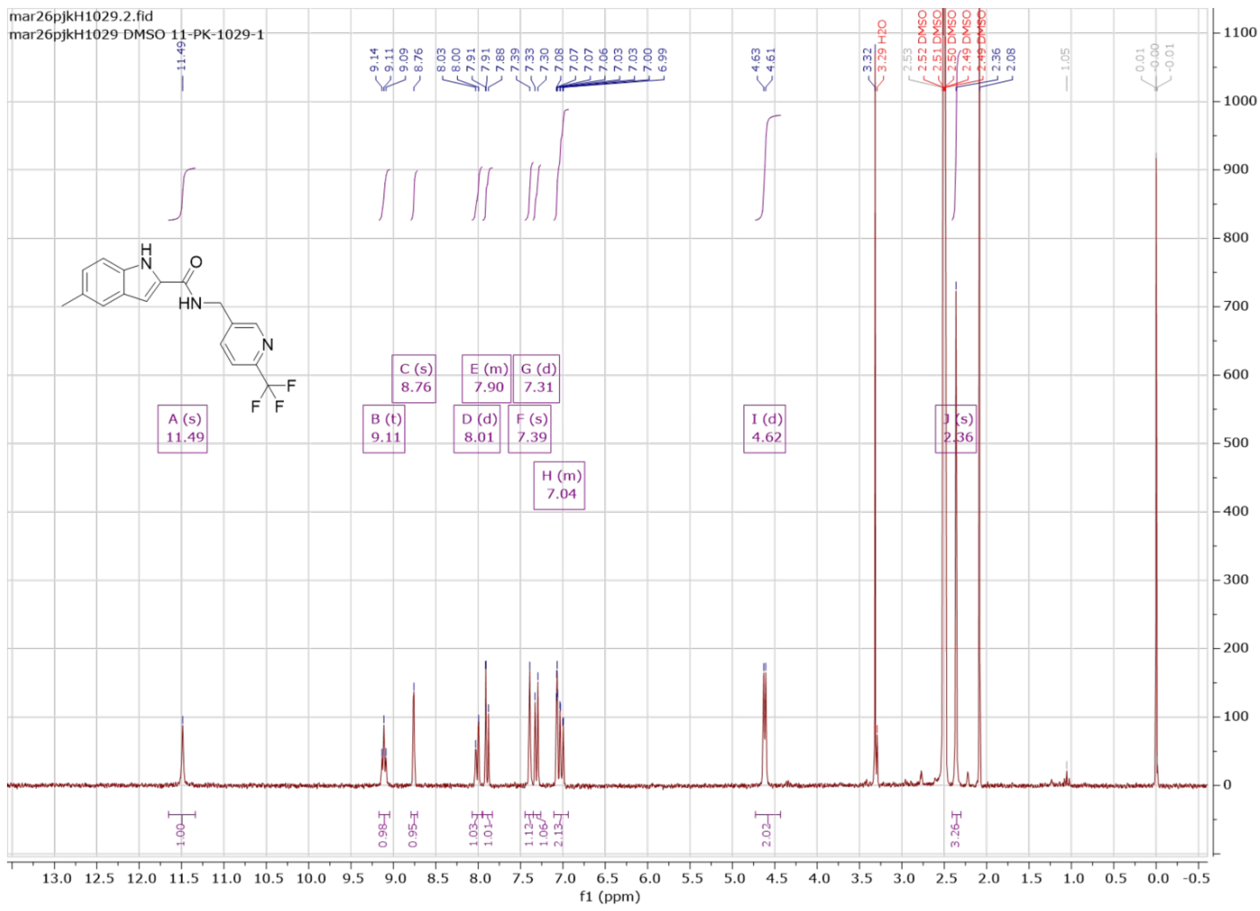

Figure S80: 5-methyl-N-((6-(trifluoromethyl)pyridin-3-yl)methyl)-1H-indole-2-carboxamide (66)

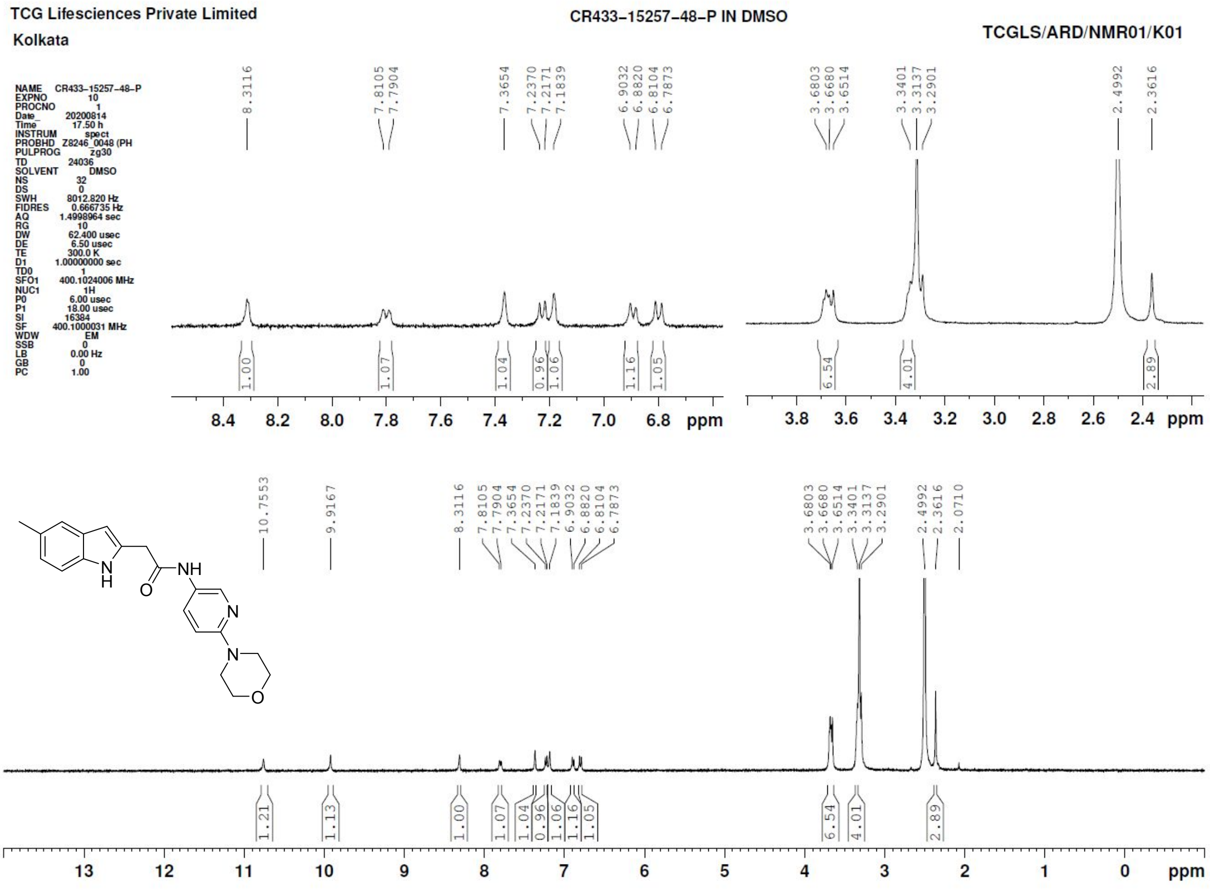

Figure S81: 2-(5-methyl-1H-indol-2-yl)-N-(6-morpholinopyridin-3-yl)acetamide (67)

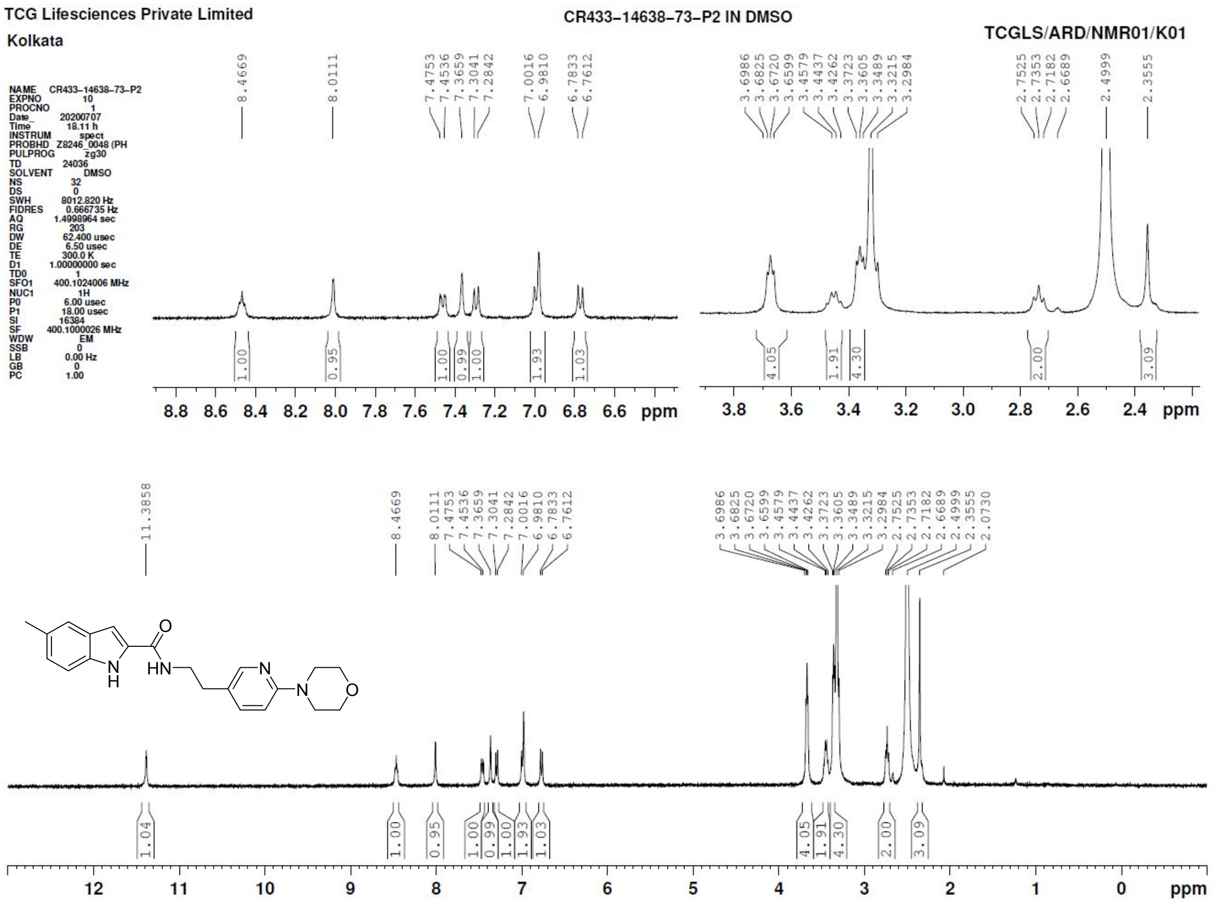

Figure S82: 5-methyl-N-(2-(6-morpholinopyridin-3-yl)ethyl)-1H-indole-2-carboxamide (68)

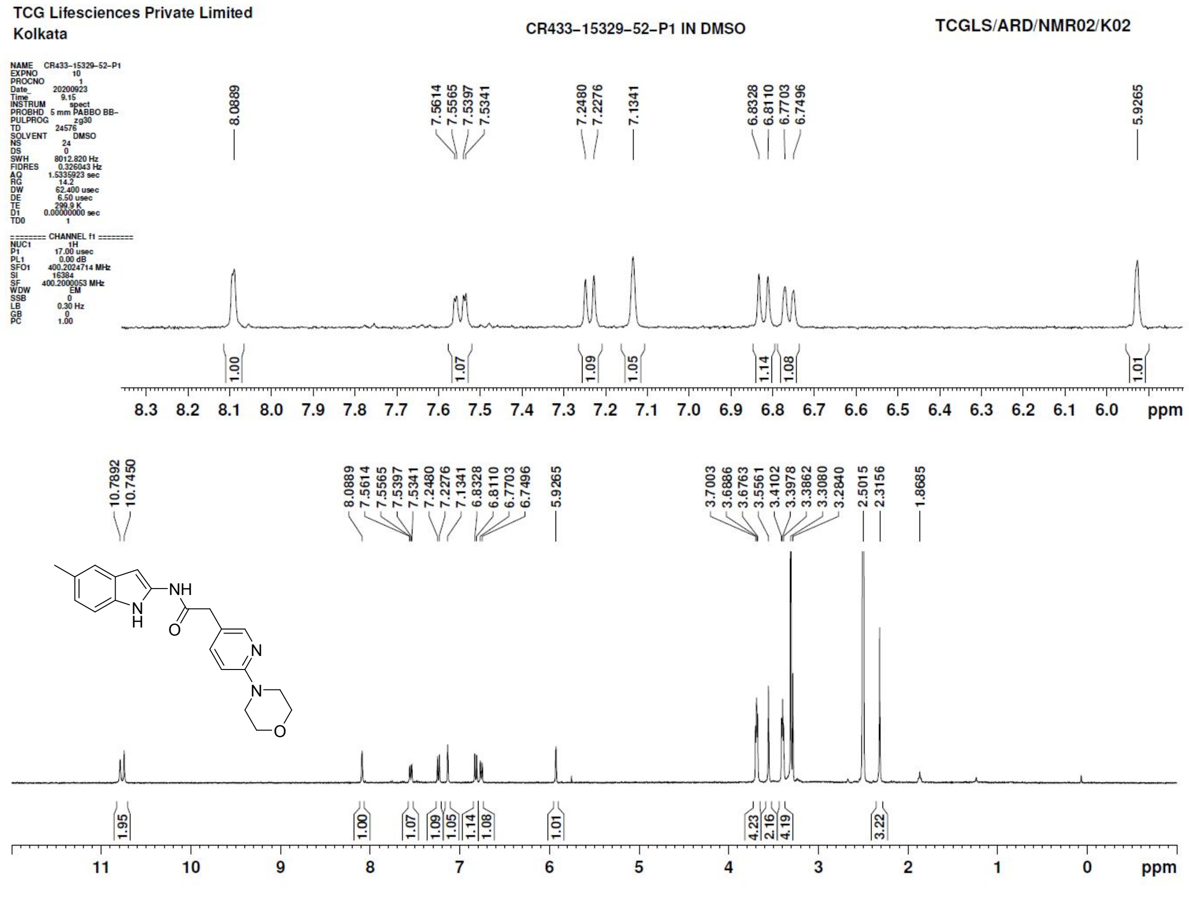

Figure S83: N-(5-methyl-1H-indol-2-yl)-2-(6-morpholinopyridin-3-yl)acetamide (69)

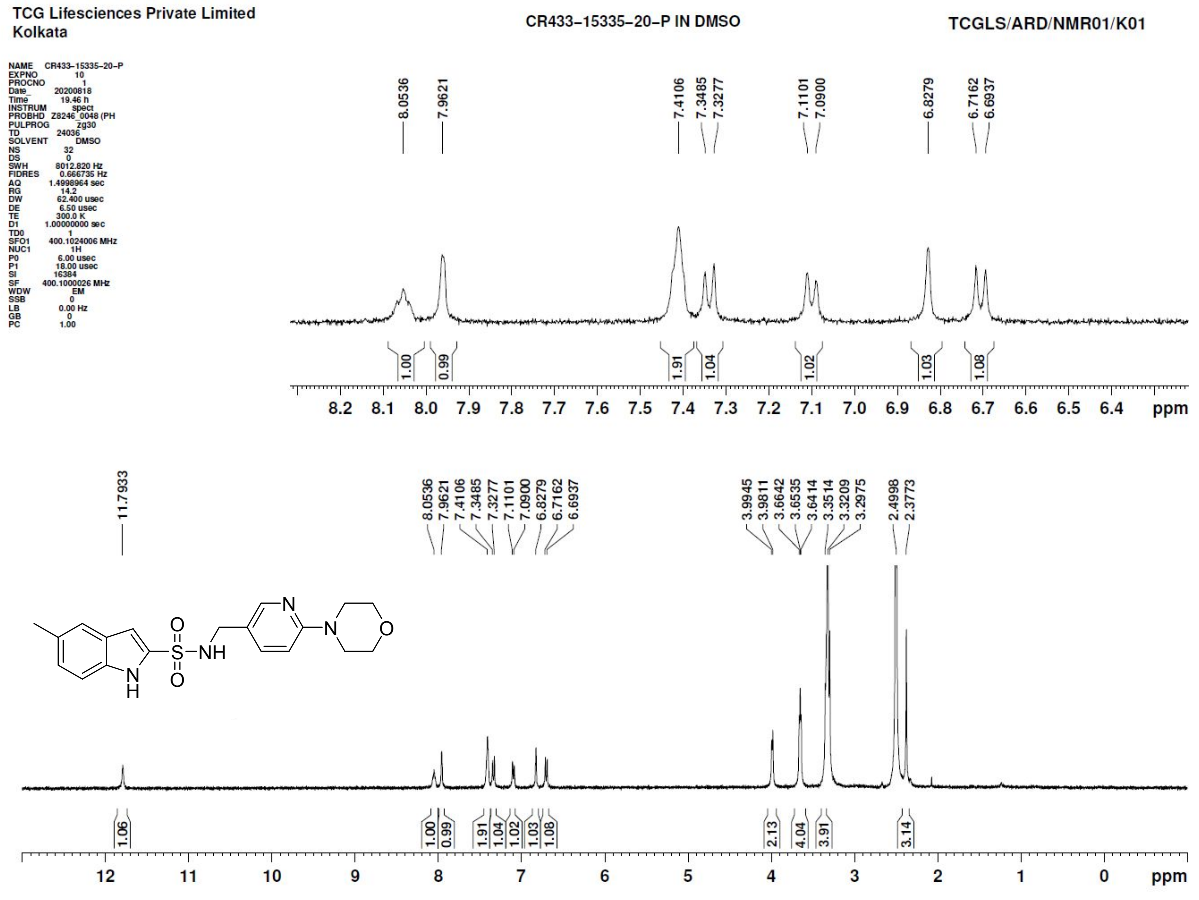

Figure S84: 5-methyl-N-((6-morpholinopyridin-3-yl)methyl)-1H-indole-2-sulfonamide (70)

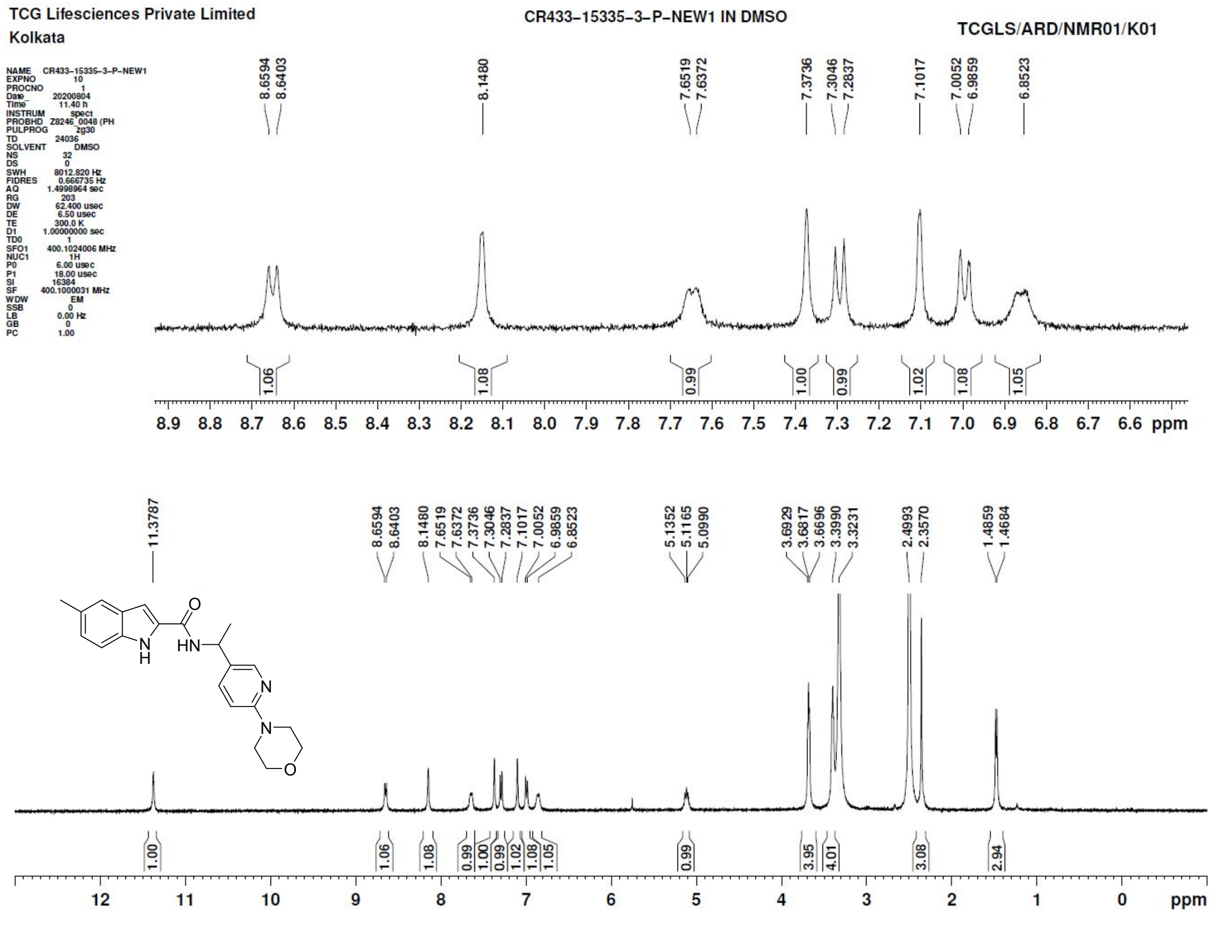

Figure S85: 5-methyl-N-(1-(6-morpholinopyridin-3-yl)ethyl)-1H-indole-2-carboxamide (71)

This report was created by ACD/NMR Processor Academic Edition. For more information go to www.acdlabs.com/nmrproc/

Acquisition Time (sec)	3.2768	***Comment***	DESSOY_MAD2014 CDCl3 250MHz fev14madH3			***Date***	14 Feb 2020 17:34:44
File Name	\\nmrsparc.iqm.unicamp.br\espectros\bruker250\2020\fev20\Reserva\Luiz Carlos\fev14madH3_001001r					***Frequency (MHz)***	250.13
Nucleus	1H	***Number of Transients***	8	***Original Points Count***	16384	***Points Count***	65536
Pulse Sequence	zg30	***Solvent***	CHLOROFORM-d	***Spectrum Offset (Hz)***	1541.0742	***Sweep Width (Hz)***	5000.00
Temperature (degree C)	25.148						

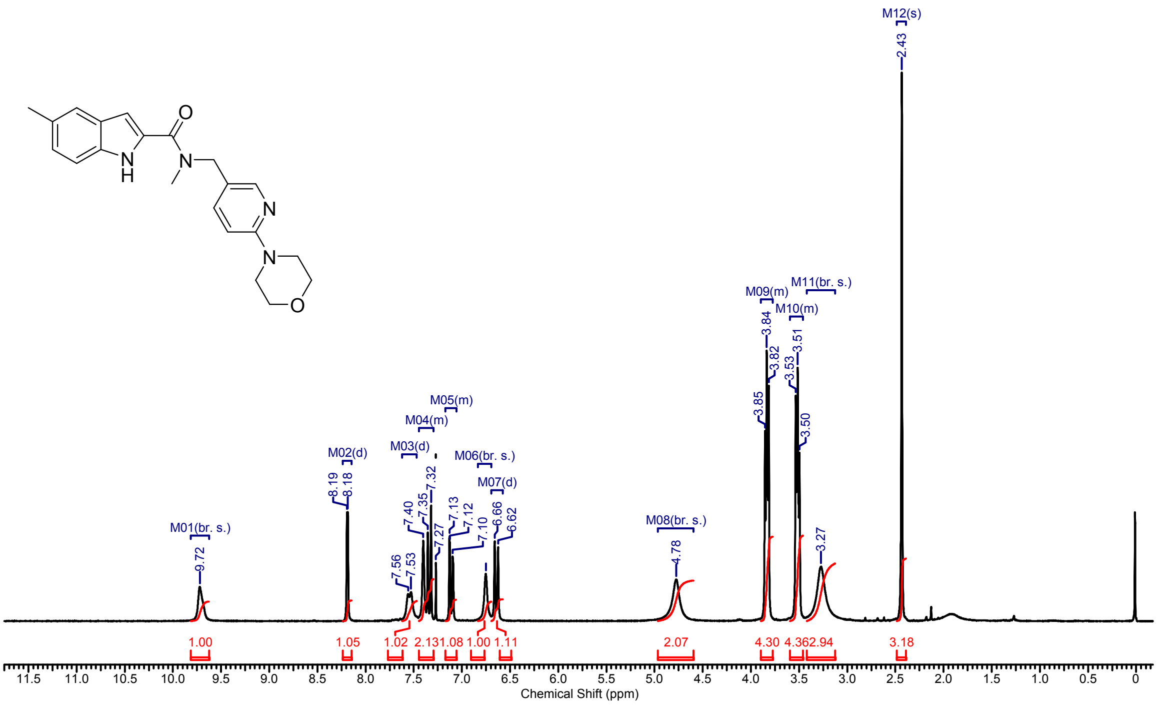

Figure S86: N,5-dimethyl-N-((6-morpholinopyridin-3-yl)methyl)-1H-indole-2-carboxamide (72)

This report was created by ACD/NMR Processor Academic Edition. For more information go to www.acdlabs.com/nmrproc/

Acquisition Time (sec)	1.5903	***Comment***	Dessoy - MAD 2043 - CDCl3 - Avance 500 MHz - nov30madH1 - 1H		
Date	30 Nov 2020 08:24:22	***File Name***	\\nmrsparc.iqm.unicamp.br\espectros\avance500\2020\nov20\Sala\Luiz Carlos\no30madH1_001001r		
Frequency (MHz)	499.87	***Nucleus***	1H	***Number of Transients***	16
Original Points Count	16384	***Points Count***	65536	***Pulse Sequence***	zg30
Solvent	CHLOROFORM-d	***Spectrum Offset (Hz)***	3080.1555	***Sweep Width (Hz)***	10302.20
Temperature (degree C)	25.140				

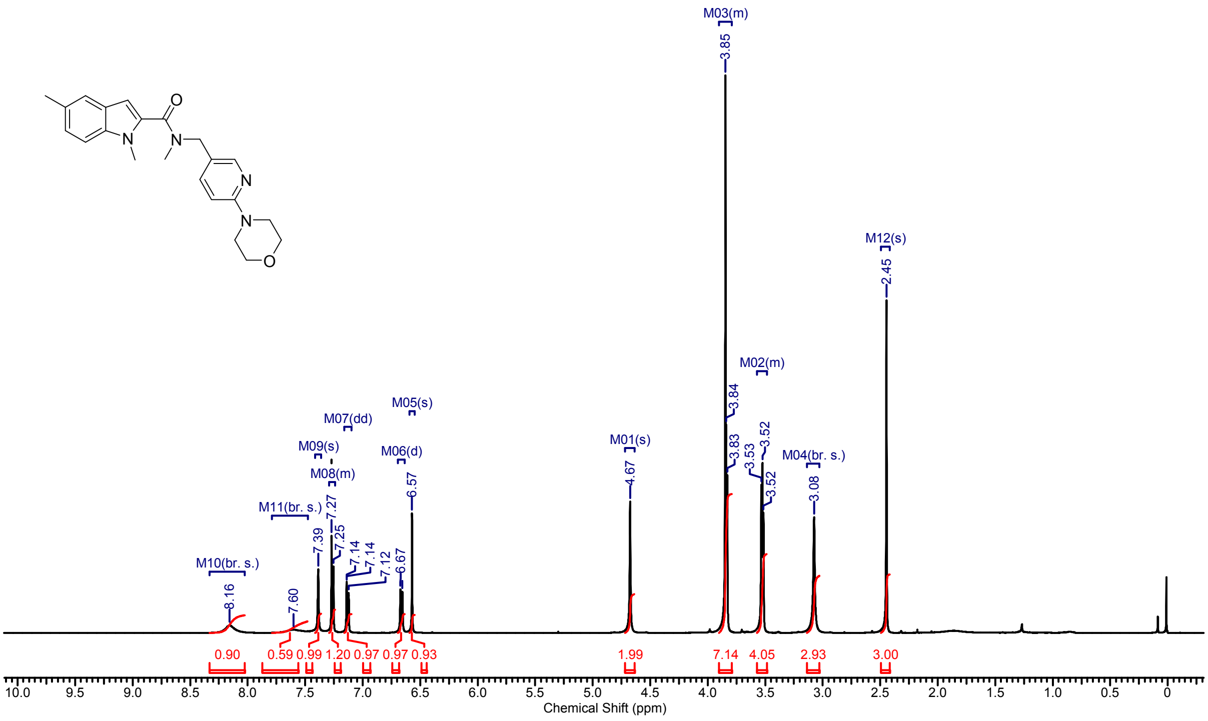

Figure S87: N,1,5-trimethyl-N-((6-morpholinopyridin-3-yl)methyl)-1H-indole-2-carboxamide (73)

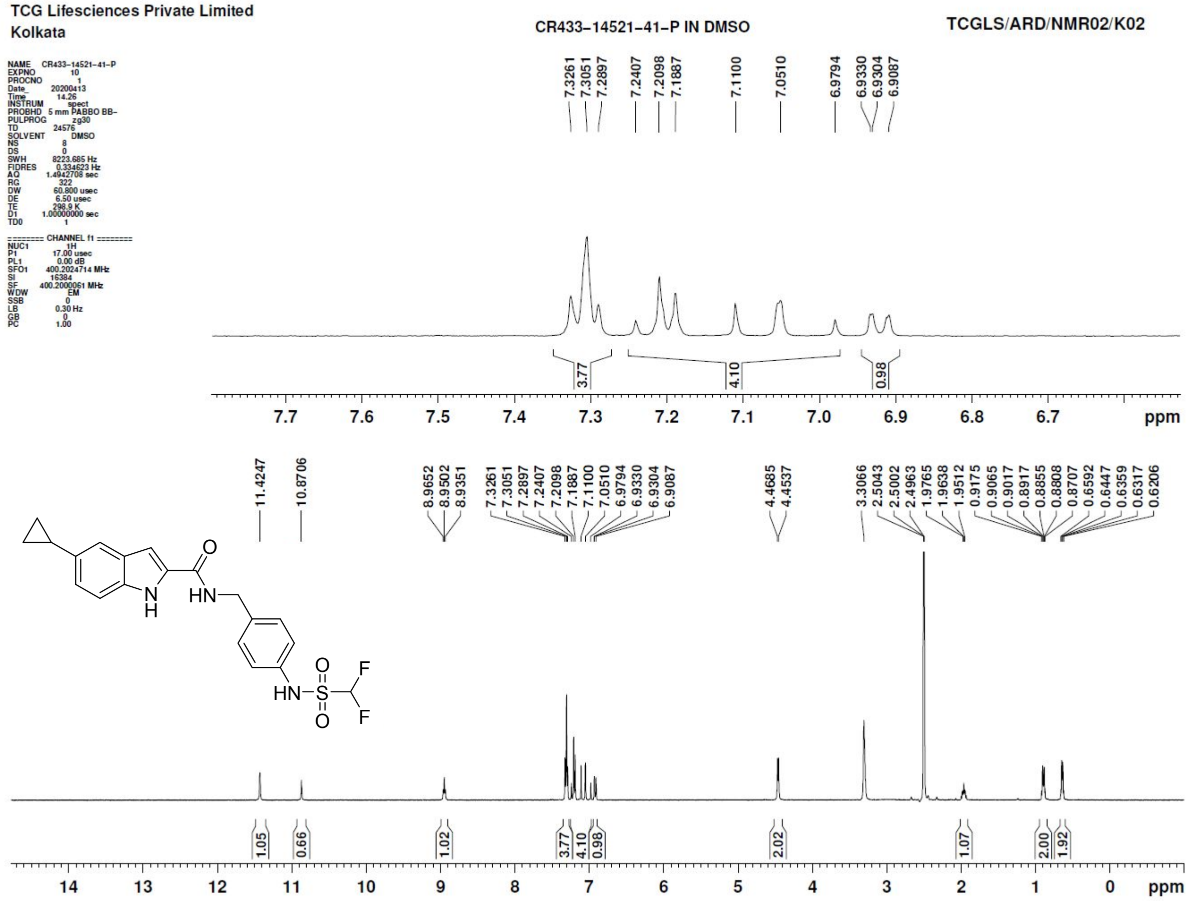

Figure S88: 5-cyclopropyl-N-(4-((difluoromethyl)sulfonamido)benzyl)-1H-indole-2-carboxamide (74)

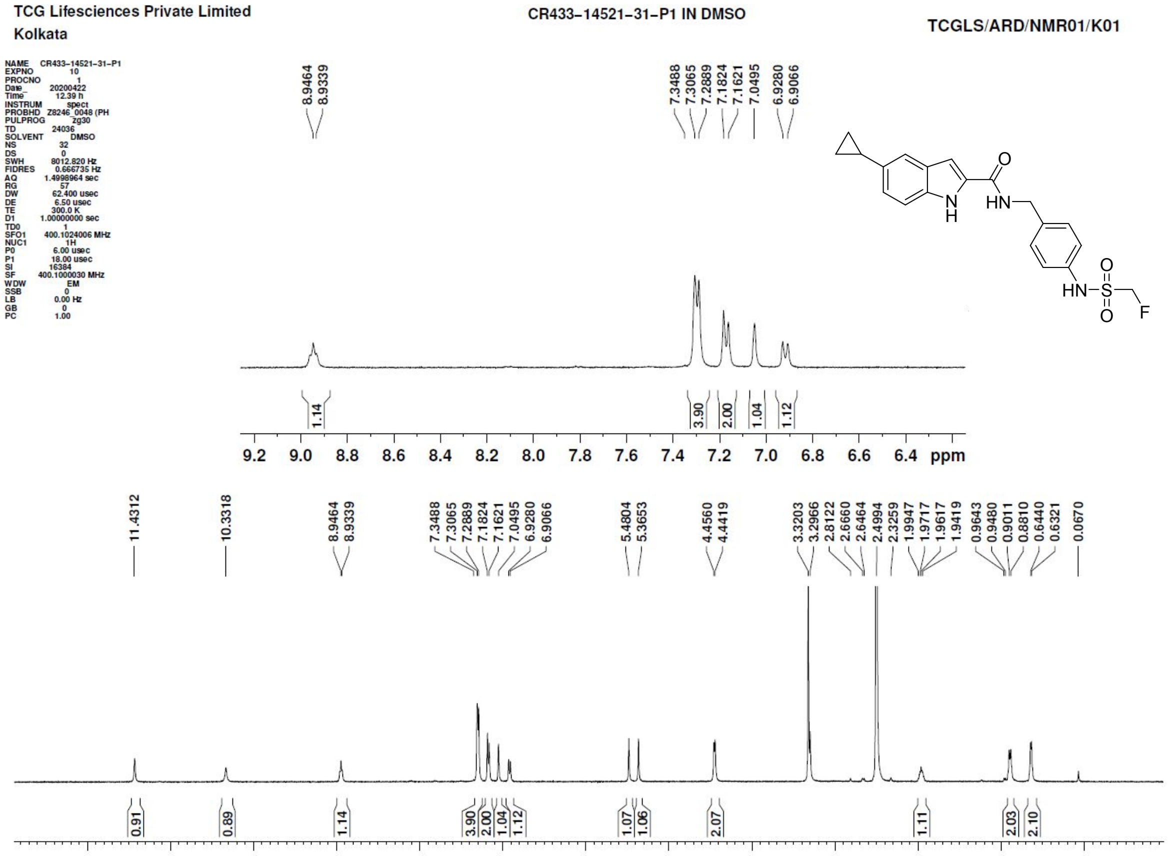

Figure S89: 5-cyclopropyl-N-(4-((fluoromethyl)sulfonamido)benzyl)-1H-indole-2-carboxamide (75)

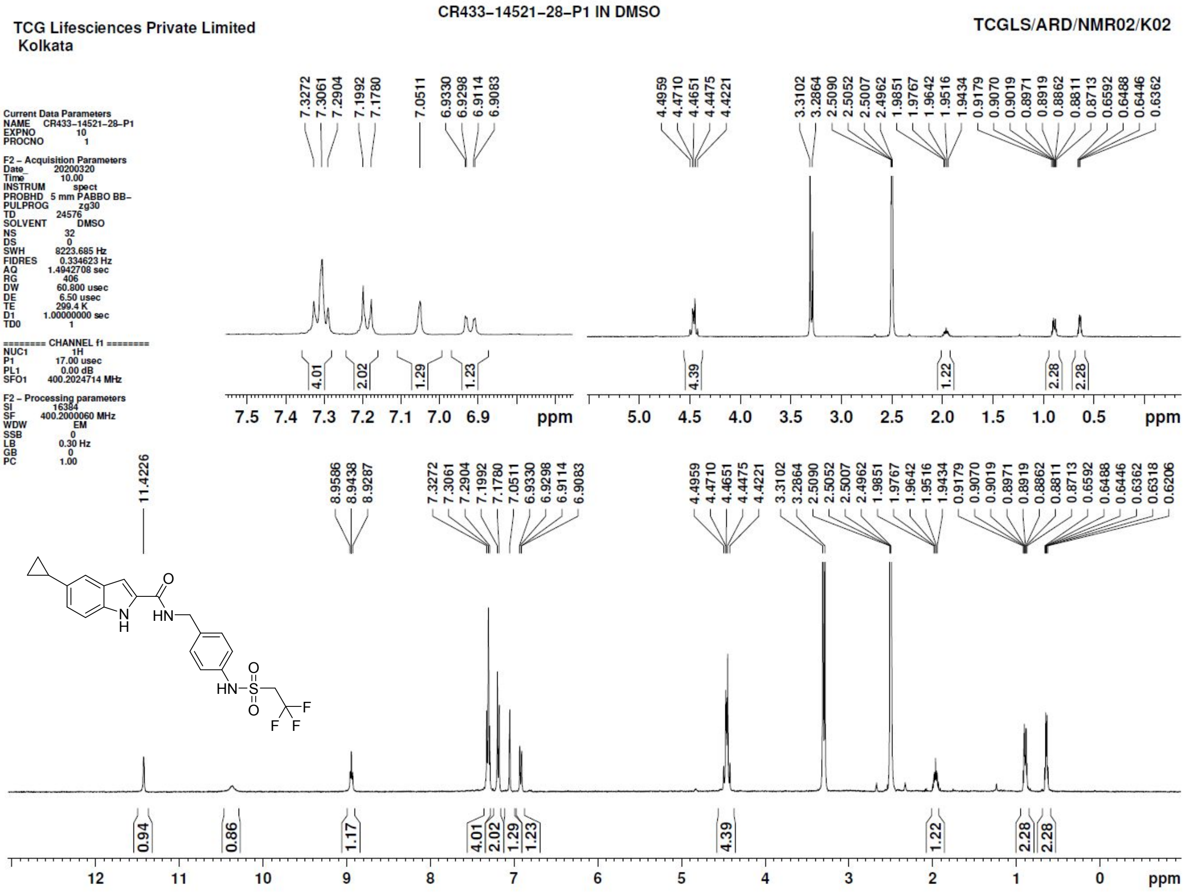

Figure S90: 5-cyclopropyl-N-(4-((2,2,2-trifluoroethyl)sulfonamido)benzyl)-1H-indole-2-carboxamide (76)

This report was created by ACD/NMR Processor Academic Edition. For more information go to www.acdlabs.com/nmrproc/

Acquisition Time (sec)	2.0447	Comment	Dessoy - MAD 2122 - DMSO - Avance 400 MHz - jul05madH2 - 1H	Date	02 Aug 2021 08:12:46
File Name	\\nmrsparc.iqm.unicamp.br\espectros\avance400\2021\ago21\Sala\Luiz Carlos\ago02madH2_001001r			Frequency (MHz)	400.18
Nucleus	1H	Number of Transients	16	Original Points Count 16384	Points Count 65536
Pulse Sequence	zg30	Solvent	DMSO-d6	Spectrum Offset (Hz) 2468.2139	Sweep Width (Hz) 8012.82
Temperature (degree C)	80.119				

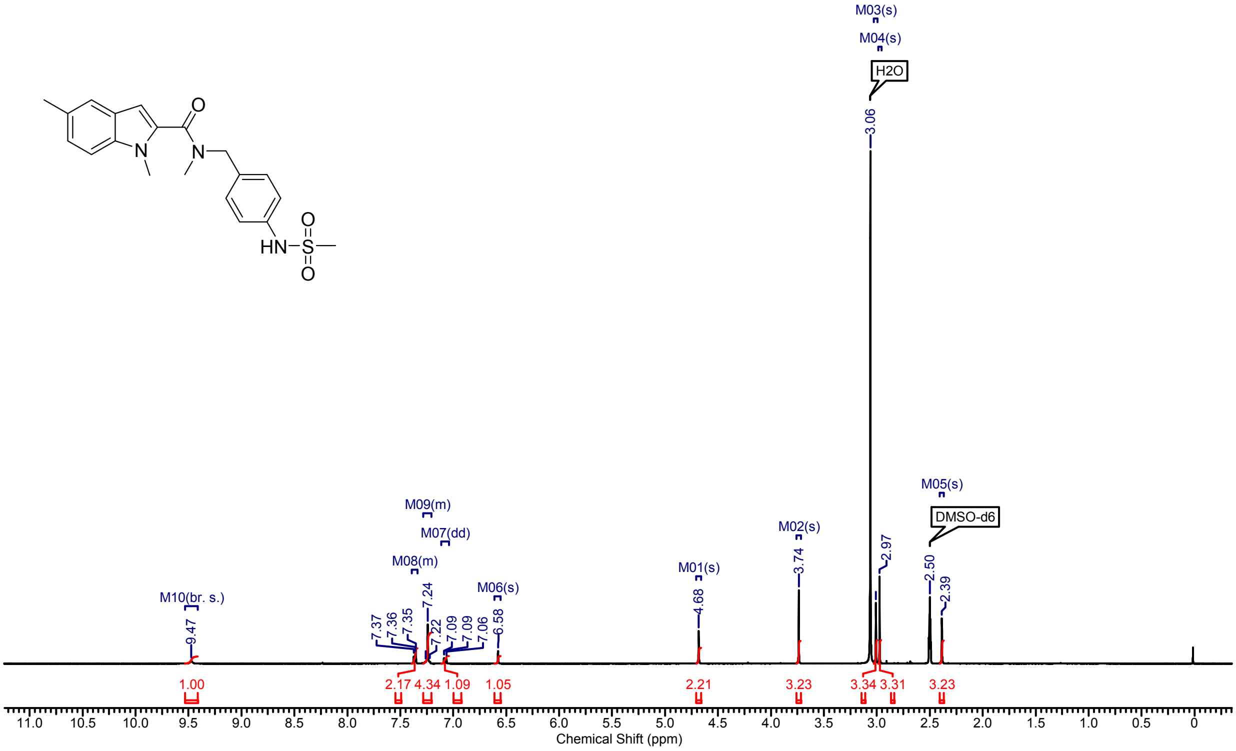

Figure S91: N,1,5-trimethyl-N-(4-(methylsulfonamido)benzyl)-1H-indole-2-carboxamide (77)

This report was created by ACD/NMR Processor Academic Edition. For more information go to www.acdlabs.com/nmrproc/

Acquisition Time (sec)	2.0447	***Comment***	Dessoy - MAD 2128 - DMSO - Avance 400 MHz - jul05madH3 - 1H			***Date***	02 Aug 2021 08:29:16
File Name	\\nmrsparc.iqm.unicamp.br\espectros\avance400\2021\ago21\Sala\Luiz Carlos\ago02madH3_001001r					***Frequency (MHz)***	400.18
Nucleus	1H	***Number of Transients***	16	***Original Points Count***	16384	***Points Count***	65536
Pulse Sequence	zg30	***Solvent***	DMSO-d6	***Spectrum Offset (Hz)***	2468.2139	***Sweep Width (Hz)***	8012.82
Temperature (degree C)	80.119						

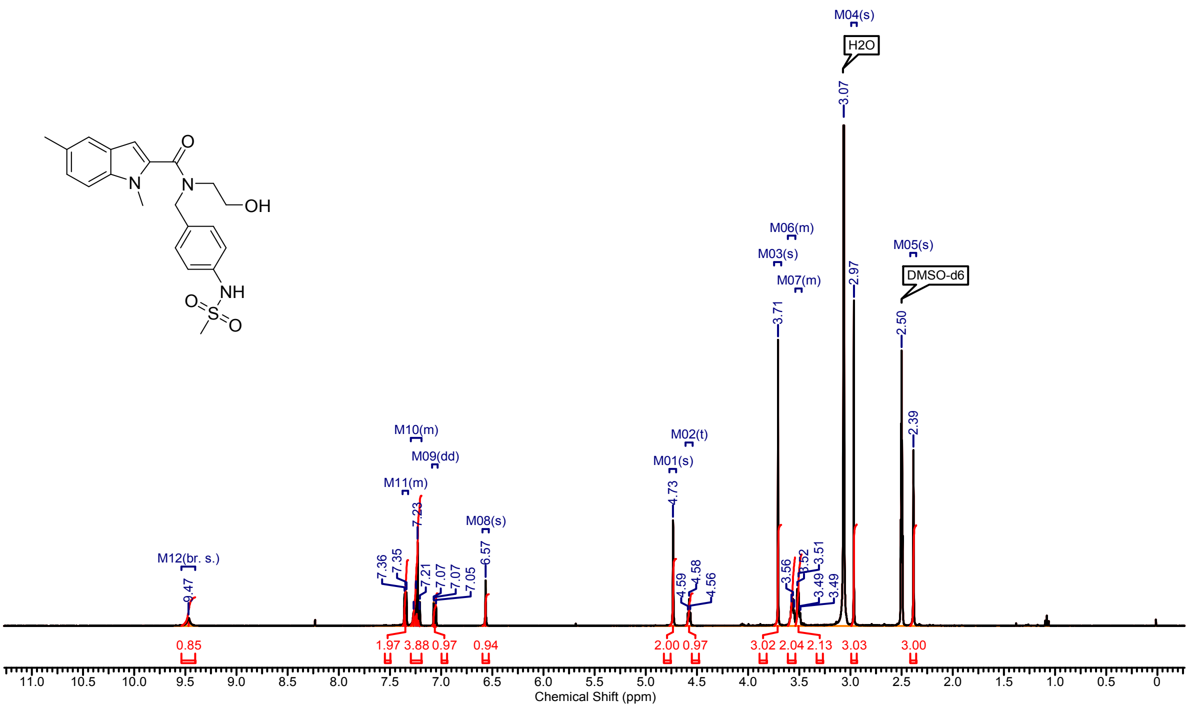

Figure S92: N-(2-hydroxyethyl)-1,5-dimethyl-N-(4-(methylsulfonamido)benzyl)-1H-indole-2-carboxamide (78)

This report was created by ACD/NMR Processor Academic Edition. For more information go to www.acdlabs.com/nmrproc/

Acquisition Time (sec)	2.0447	***Comment***	Dessoy - MAD 2132 - DMSO - Avance 400 MHz - jul05madH1 - 1H			***Date***	02 Aug 2021 07:57:06
File Name	\\nmrsparc.iqm.unicamp.br\espectros\avance400\2021\ago21\Sala\Luiz Carlos\ago02madH1_001001r					***Frequency (MHz)***	400.18
Nucleus	1H	***Number of Transients***	16	***Original Points Count***	16384	***Points Count***	65536
Pulse Sequence	zg30	***Solvent***	DMSO-d6	***Spectrum Offset (Hz)***	2468.0916	***Sweep Width (Hz)***	8012.82
Temperature (degree C)	80.237						

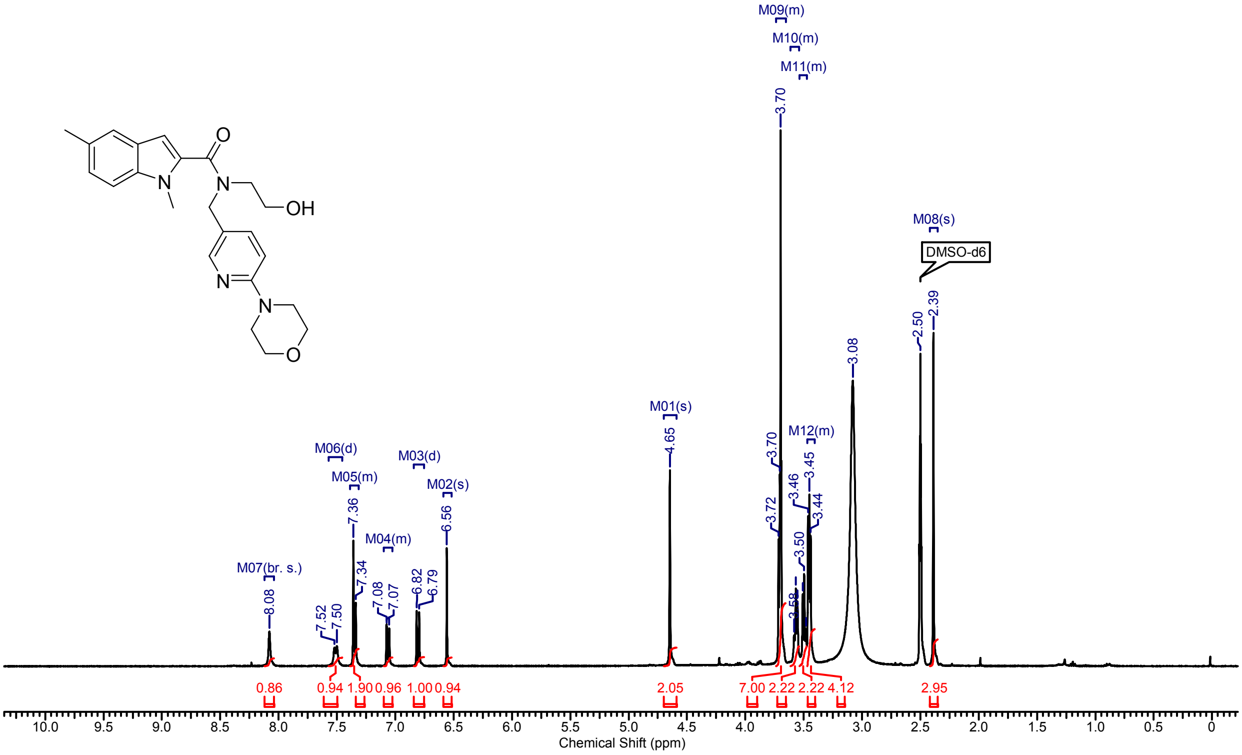

Figure S93: N-(2-hydroxyethyl)-1,5-dimethyl-N-((6-morpholinopyridin-3-yl)methyl)-1H-indole-2-carboxamide (79)

This report was created by ACD/NMR Processor Academic Edition. For more information go to www.acdlabs.com/nmrproc/

Acquisition Time (sec)	2.0447	*Comment*	Dessoy - MAD 2156 - DMSO - Avance 400 MHz - set14madH1 - 1H			*Date*	14 Sep 2021 08:05:34
File Name	\\nmrsparc.iqm.unicamp.br\espectros\avance400\2021\set21\Sala\Luiz Carlos\set14madH1_002001r					*Frequency (MHz)*	400.18
Nucleus	1H	*Number of Transients*	16	*Original Points Count*	16384	*Points Count*	65536
Pulse Sequence	zg30	*Solvent*	DMSO-d6	*Spectrum Offset (Hz)*	2468.4583	*Sweep Width (Hz)*	8012.82
Temperature (degree C)	80.237						

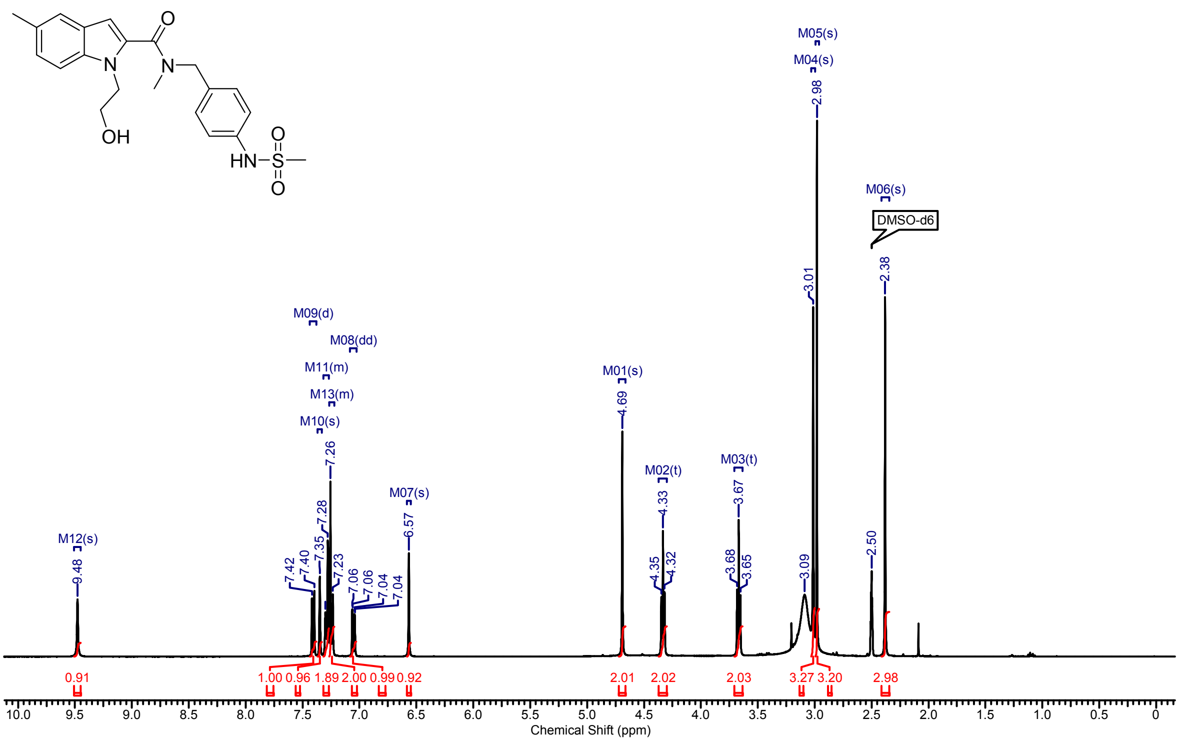

Figure S94: 1-(2-hydroxyethyl)-N,5-dimethyl-N-(4-(methylsulfonamido)benzyl)-1H-indole-2-carboxamide (80)

This report was created by ACD/NMR Processor Academic Edition. For more information go to www.acdlabs.com/nmrproc/

Acquisition Time (sec)	1.5903	*Comment*	Dessoy - MAAD 2158 - Acetona - Avance 500 MHz - set17madH1 - 1H			*Date*	17 Sep 2021 08:28:08
File Name	\\nmrsparc.iqm.unicamp.br\espectros\avance500\2021\set21\Sala\Luiz Carlos\set17madH1_001001r					*Frequency (MHz)*	499.87
Nucleus	1H	*Number of Transients*	16	*Original Points Count*	16384	*Points Count*	65536
Pulse Sequence	zg30	*Solvent*	Acetone	*Spectrum Offset (Hz)*	3078.6492	*Sweep Width (Hz)*	10302.20
Temperature (degree C)	25.151						

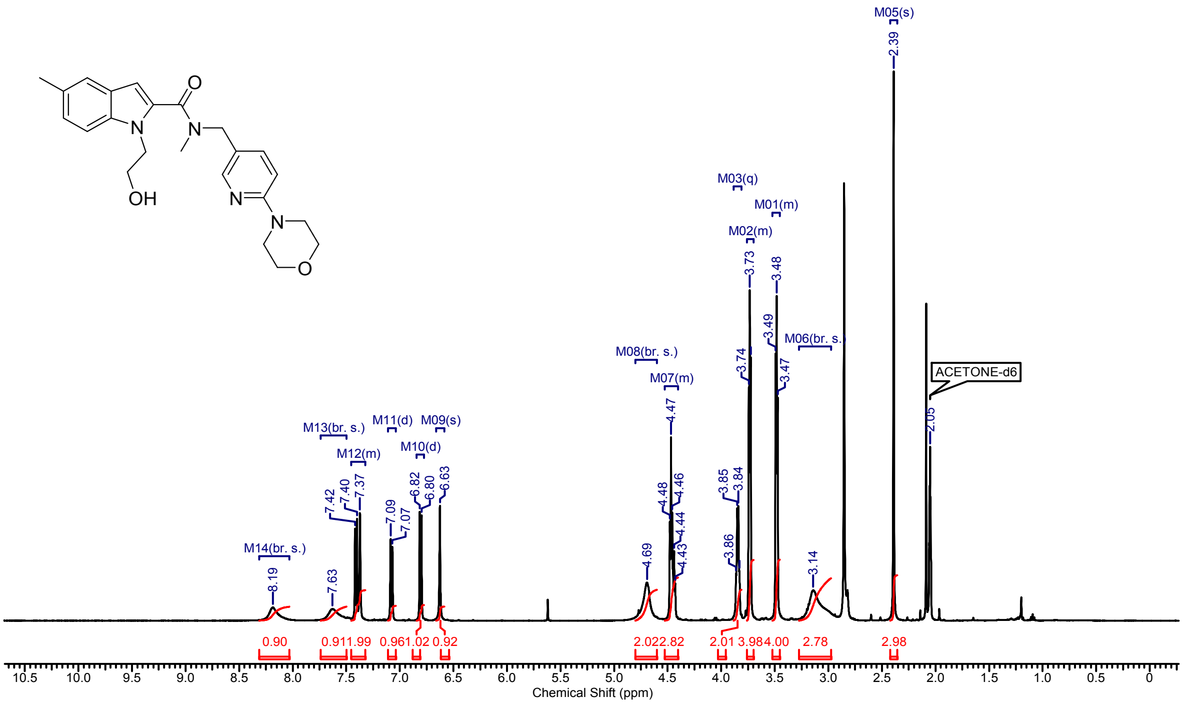

Figure S95: 1-(2-hydroxyethyl)-N,5-dimethyl-N-((6-morpholinopyridin-3-yl)methyl)-1H-indole-2-carboxamide (81)

This report was created by ACD/NMR Processor Academic Edition. For more information go to www.acdlabs.com/nmrproc/

Acquisition Time (sec)	3.2768	*Comment*	MAD2235 CDCl3	*Date*	18 Feb 2022 17:25:42		
File Name	\\nmrsparc.iqm.unicamp.br\espectros\bruker250\2022\fev22\Reserva\Luiz Carlos\Feb18-2022-MAD2235_001001r					*Frequency (MHz)*	250.13
Nucleus	1H	*Number of Transients*	8	*Original Points Count*	16384	*Points Count*	65536
Pulse Sequence	zg30	*Solvent*	CHLOROFORM-d	*Spectrum Offset (Hz)*	1540.9979	*Sweep Width (Hz)*	5000.00
Temperature (degree C)	25.148						

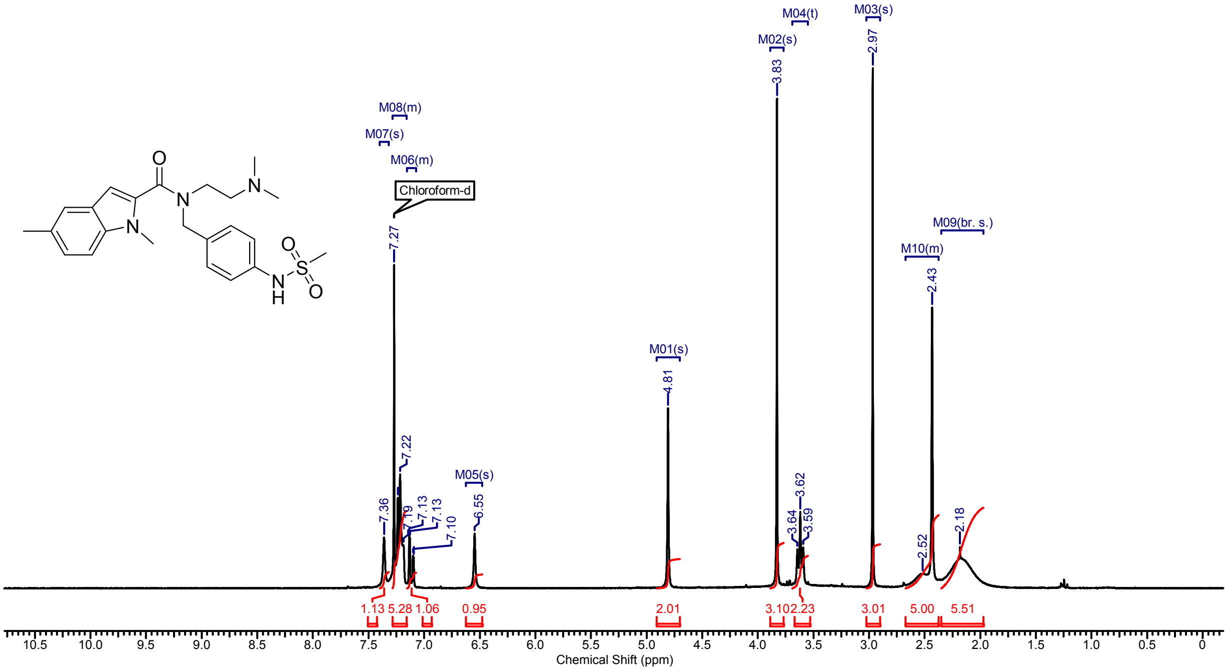

Figure S96: N-(2-(dimethylamino)ethyl)-1,5-dimethyl-N-(4-(methylsulfonamido)benzyl)-1H-indole-2-carboxamide (82)

This report was created by ACD/NMR Processor Academic Edition. For more information go to www.acdlabs.com/nmrproc/

Acquisition Time (sec)	3.1807	*Comment*	Dessoy - MAD 2202 - CDCl3 - Avance 500 MHz - dez07madH1 - 1H		
Date	07 Dec 2021 10:11:24	*File Name*	\\nmrsparc.iqm.unicamp.br\espectros\avance500\2021\dez21\Sala\Luiz Carlos\dez07madH1_001001r		
Frequency (MHz)	499.87	*Nucleus*	1H	*Number of Transients*	16
Original Points Count	32768	*Points Count*	65536	*Pulse Sequence*	zg30
Solvent	CHLOROFORM-d	*Spectrum Offset (Hz)*	3079.9985	*Sweep Width (Hz)*	10302.20
Temperature (degree C)	25.149				

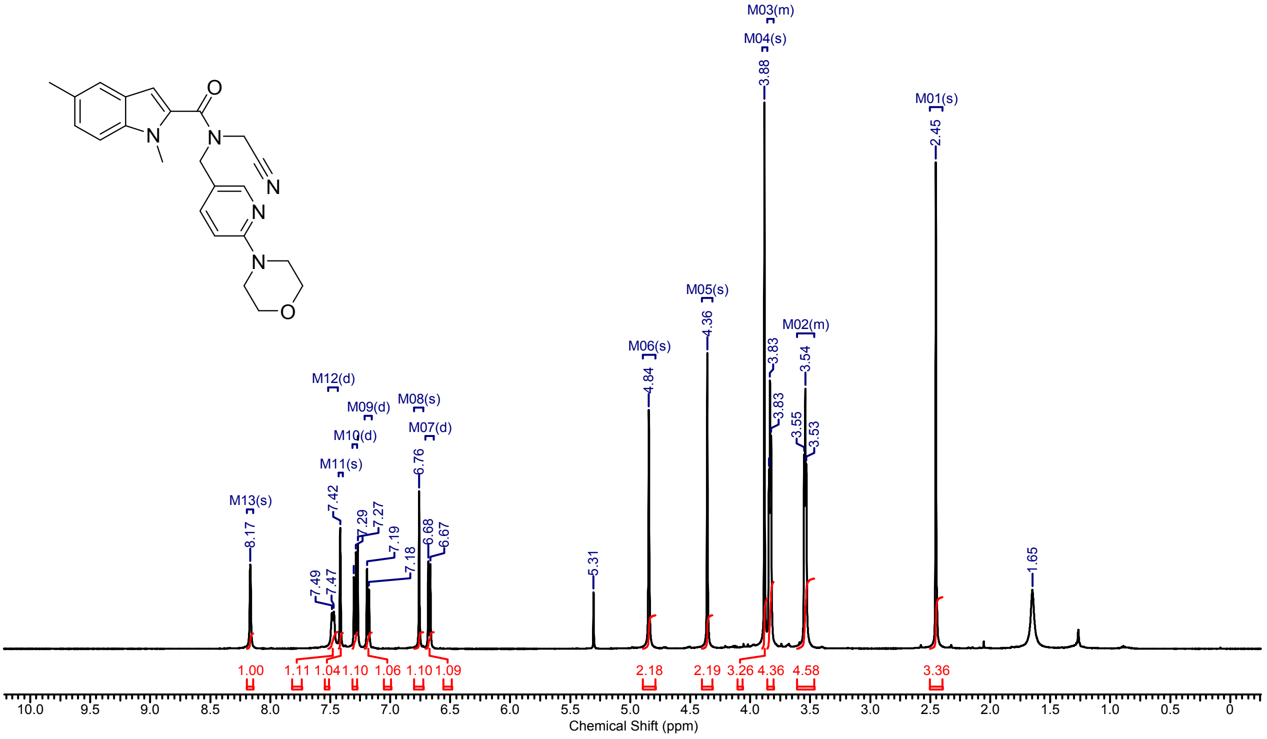

Figure S97: N-(cyanomethyl)-1,5-dimethyl-N-((6-morpholinopyridin-3-yl)methyl)-1H-indole-2-carboxamide (83)

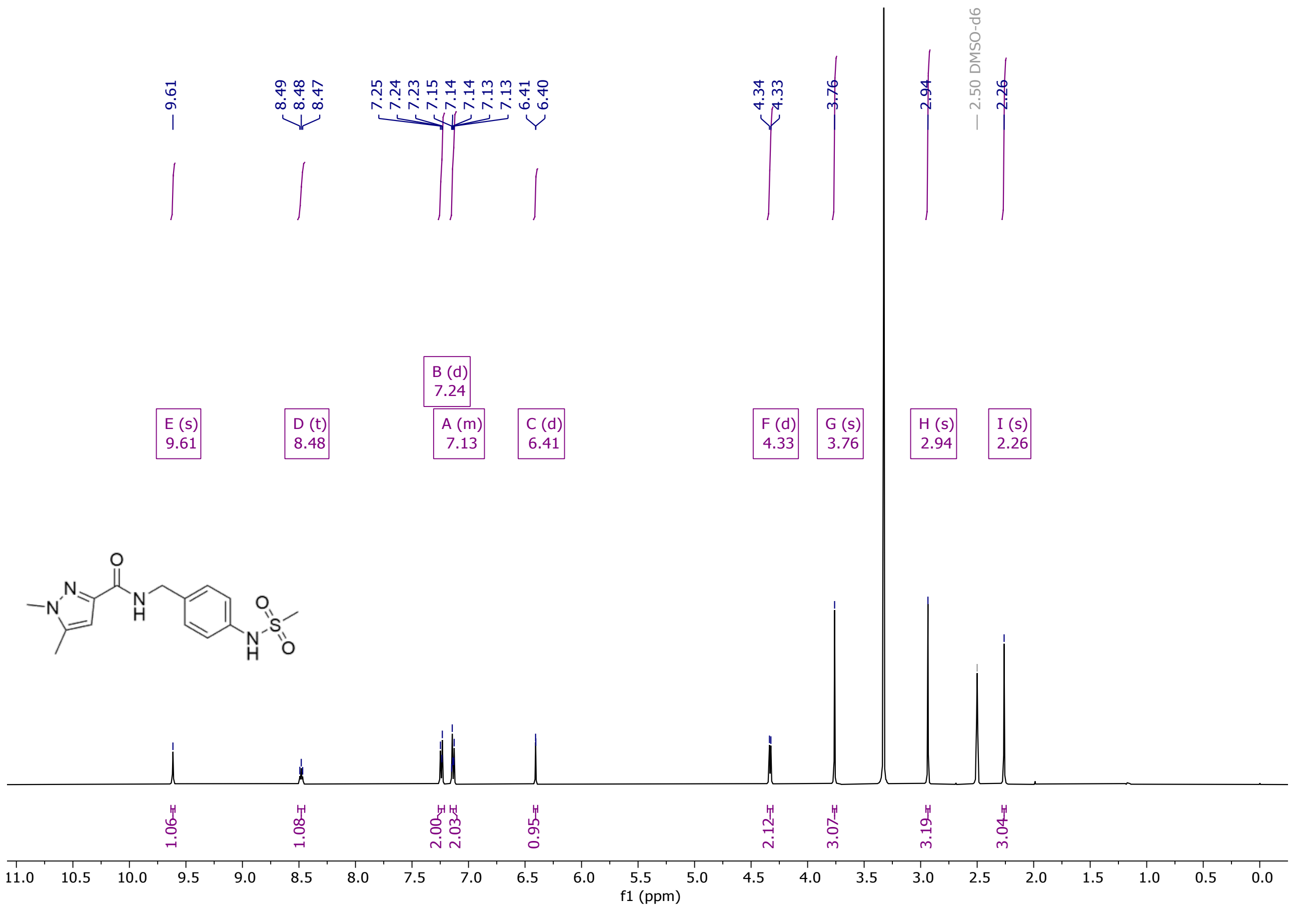

Figure S98: 1,5-dimethyl-N-(4-(methylsulfonamido)benzyl)-1H-pyrazole-3-carboxamide (84)

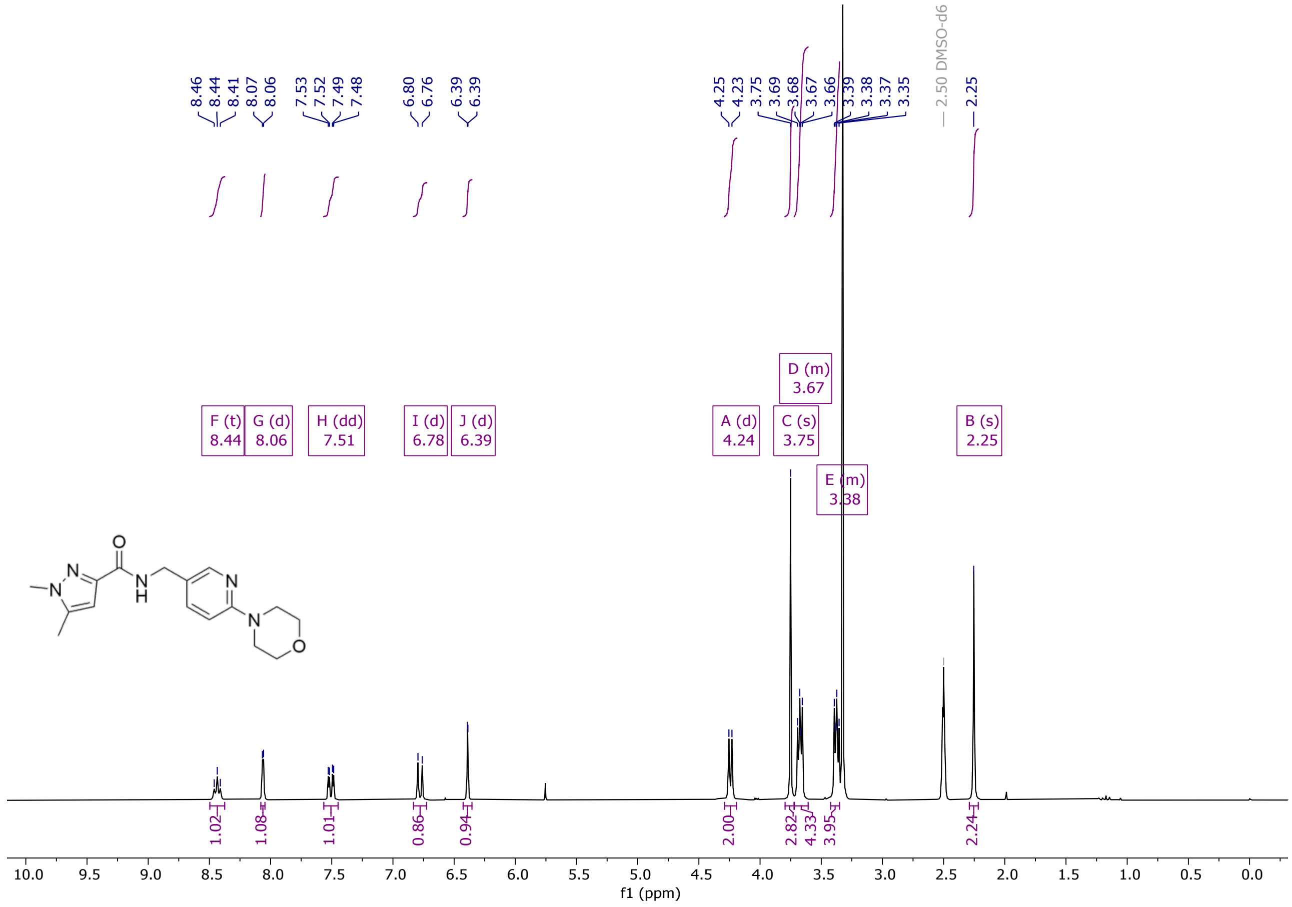

Figure S99: 1,5-dimethyl-N-((6-morpholinopyridin-3-yl)methyl)-1H-pyrazole-3-carboxamide (85)

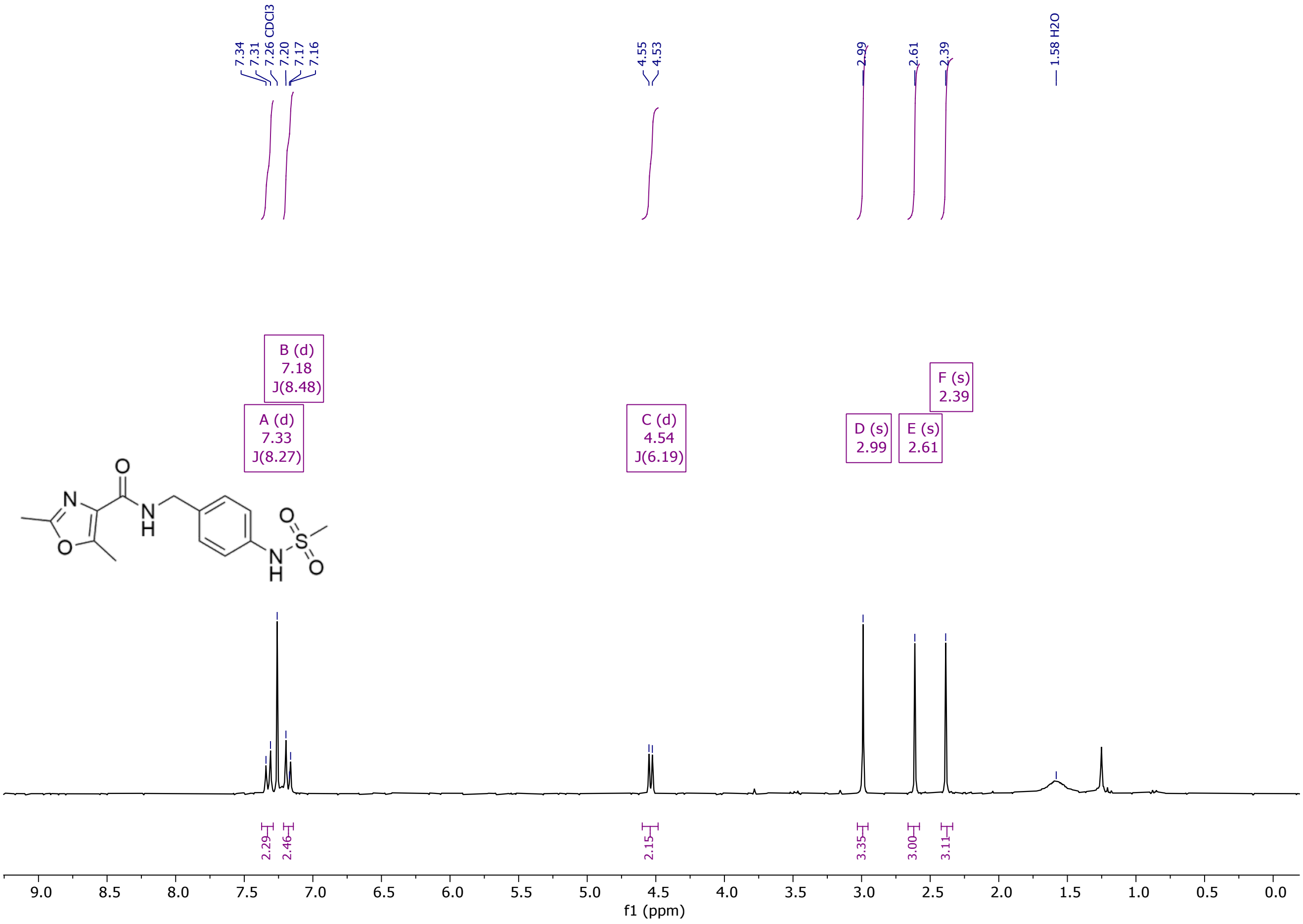

Figure S100: 2,5-dimethyl-N-(4-(methylsulfonamido)benzyl)oxazole-4-carboxamide (86)

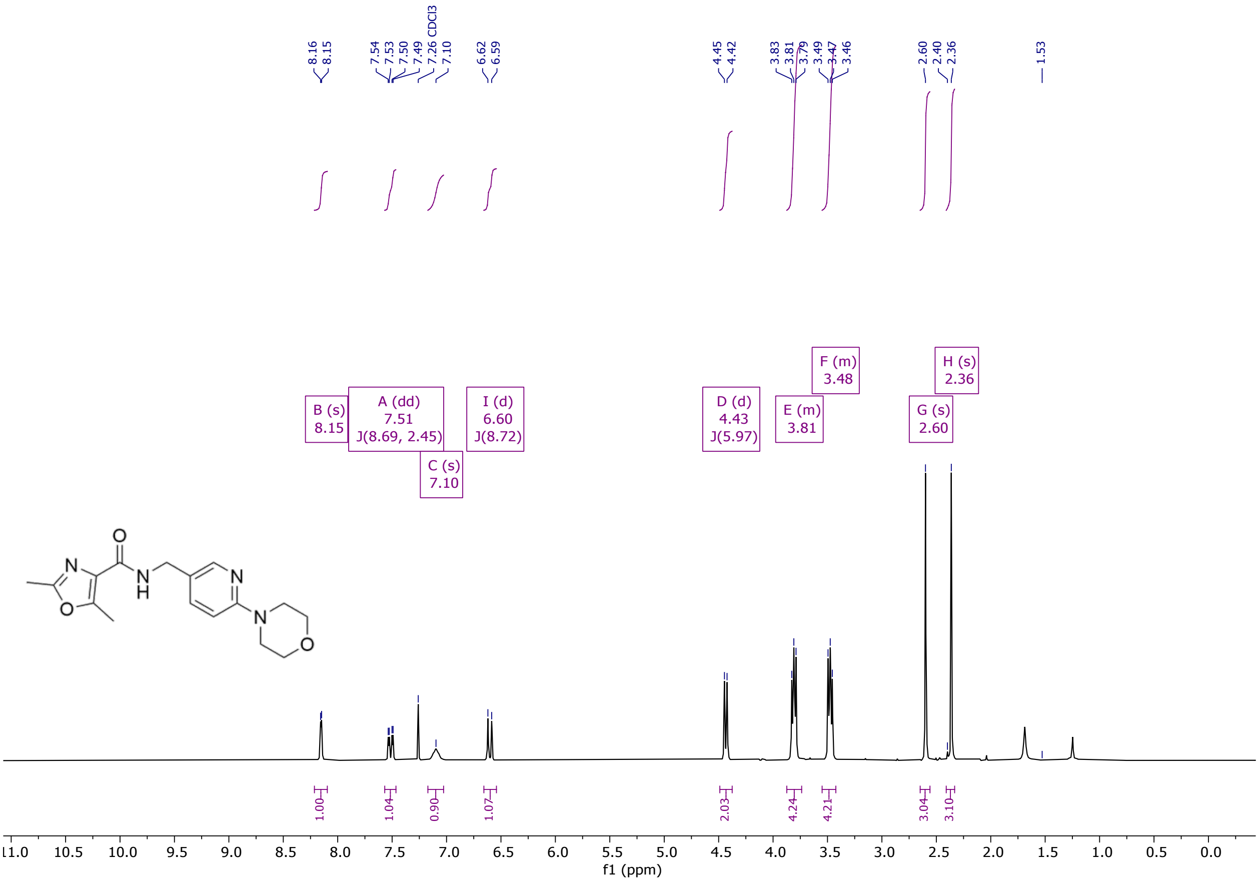

Figure S101: 2,5-dimethyl-N-((6-morpholinopyridin-3-yl)methyl)oxazole-4-carboxamide (87)

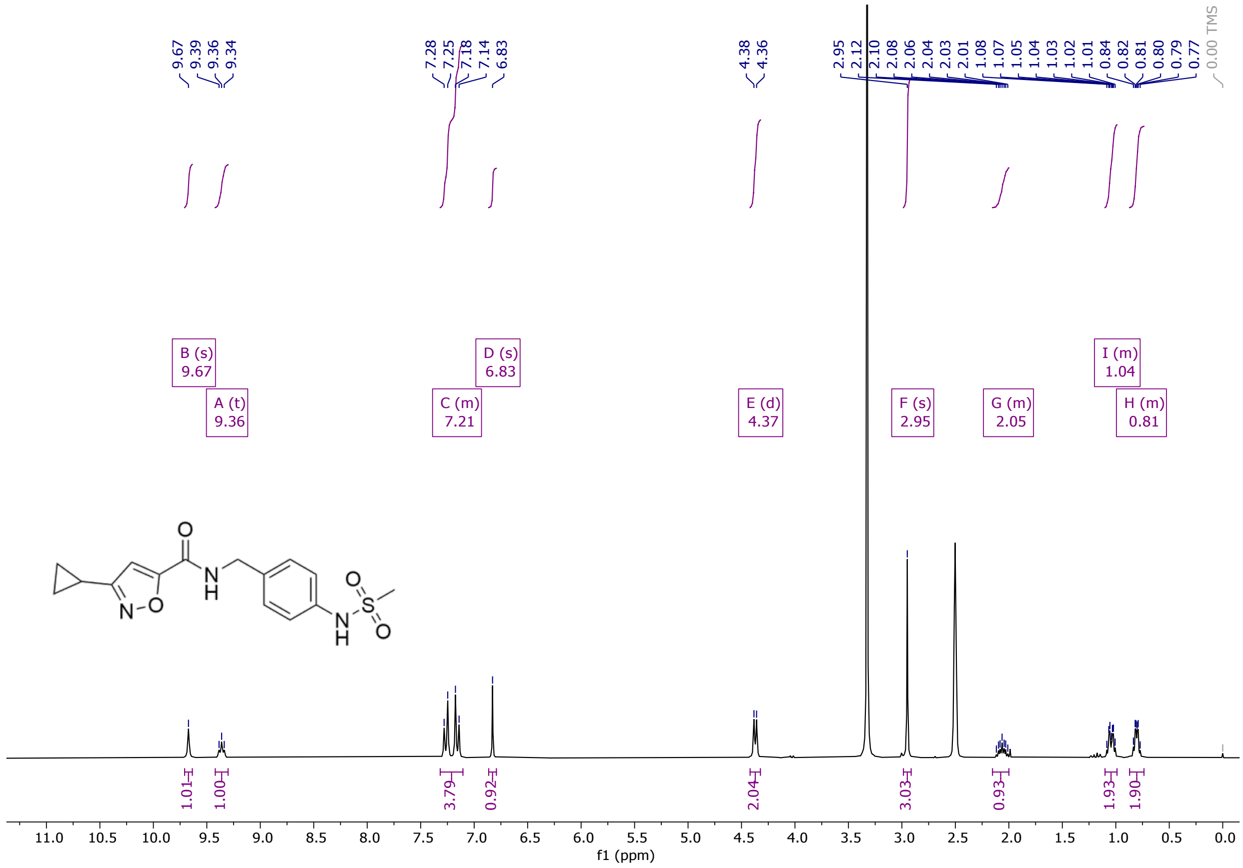

Figure S102: 3-cyclopropyl-N-(4-(methylsulfonamido)benzyl)isoxazole-5-carboxamide (88)

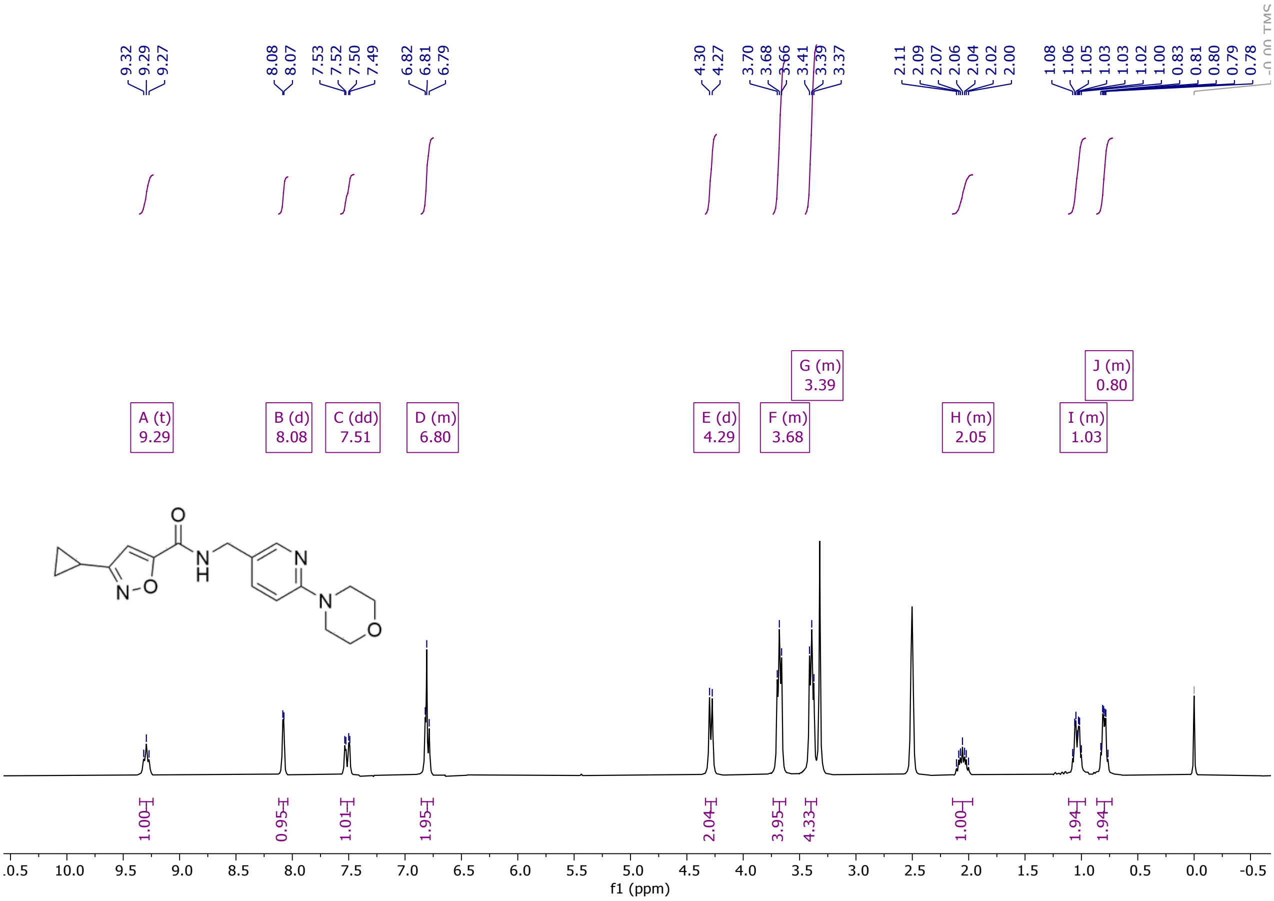

Figure S103: 3-cyclopropyl-N-((6-morpholinopyridin-3-yl)methyl)isoxazole-5-carboxamide (89)

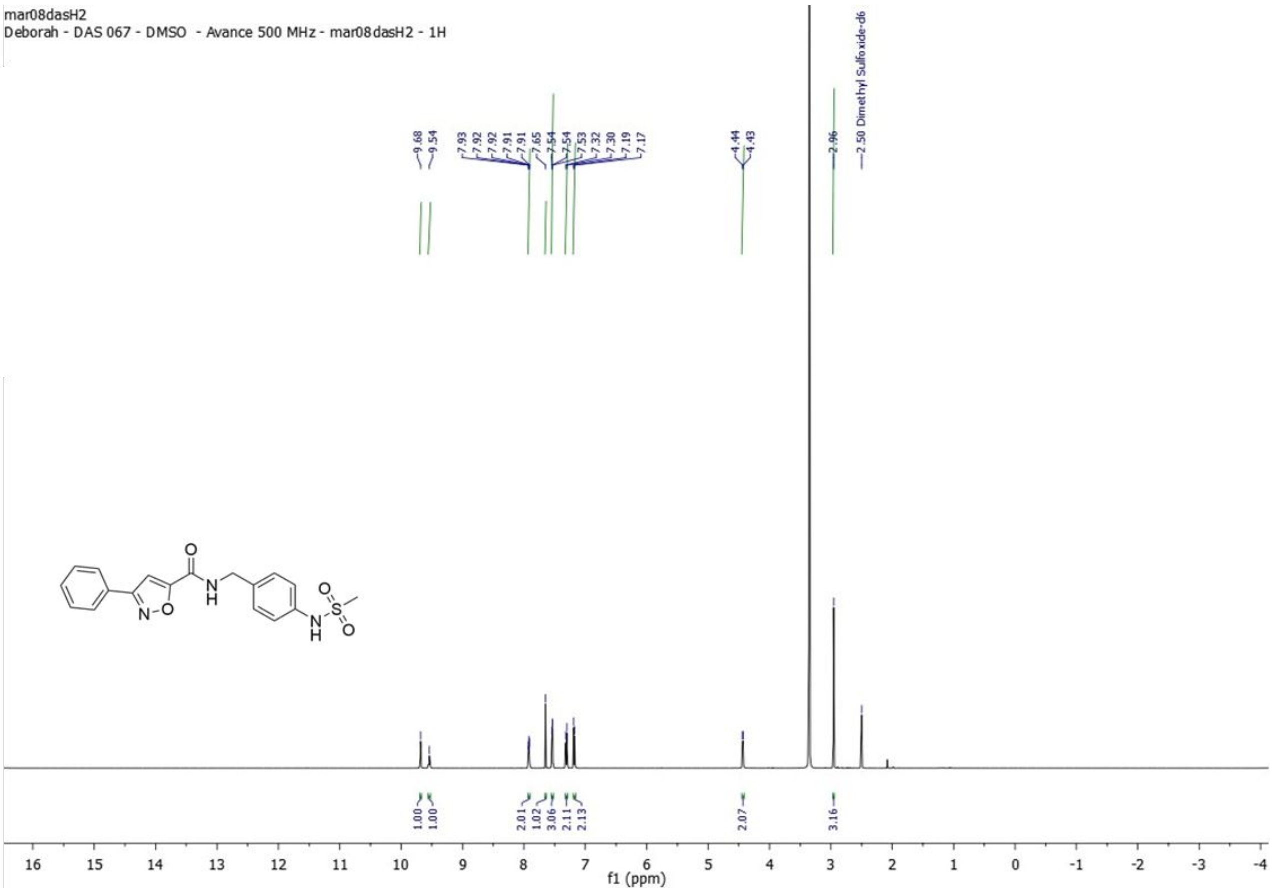

Figure S104: N-(4-(methylsulfonamido)benzyl)-3-phenylisoxazole-5-carboxamide (90)

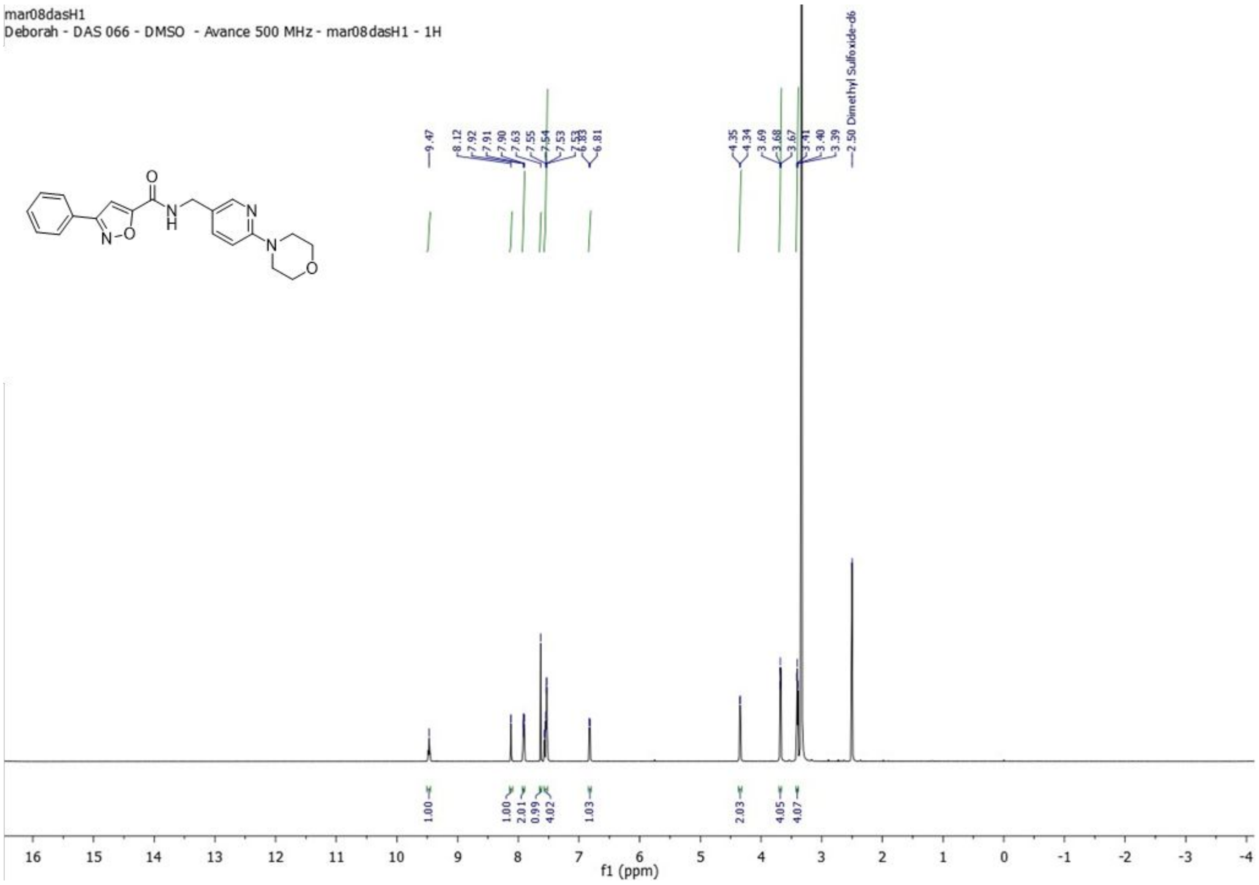

Figure S105: N-((6-morpholinopyridin-3-yl)methyl)-3-phenylisoxazole-5-carboxamide (91)

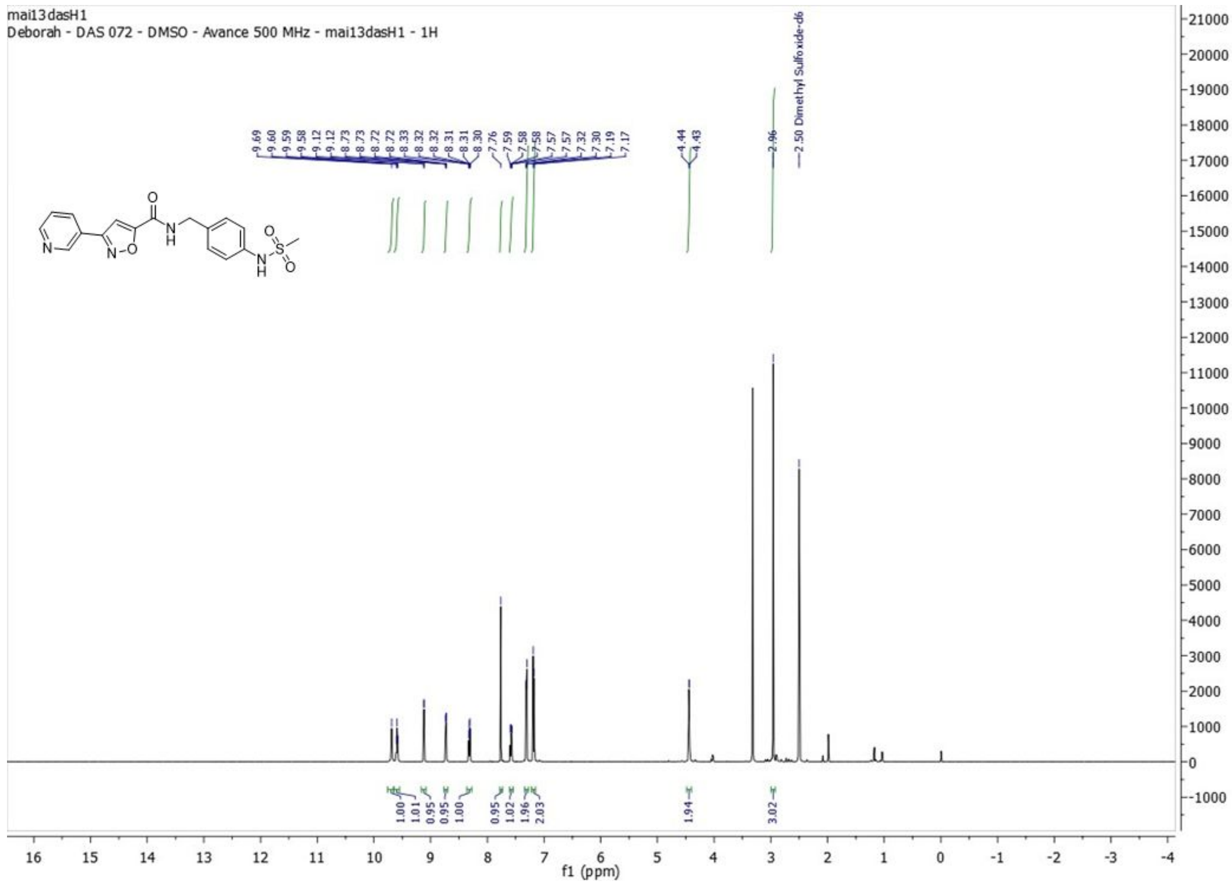

Figure S106: N-(4-(methylsulfonamido)benzyl)-3-(pyridin-3-yl)isoxazole-5-carboxamide (92)

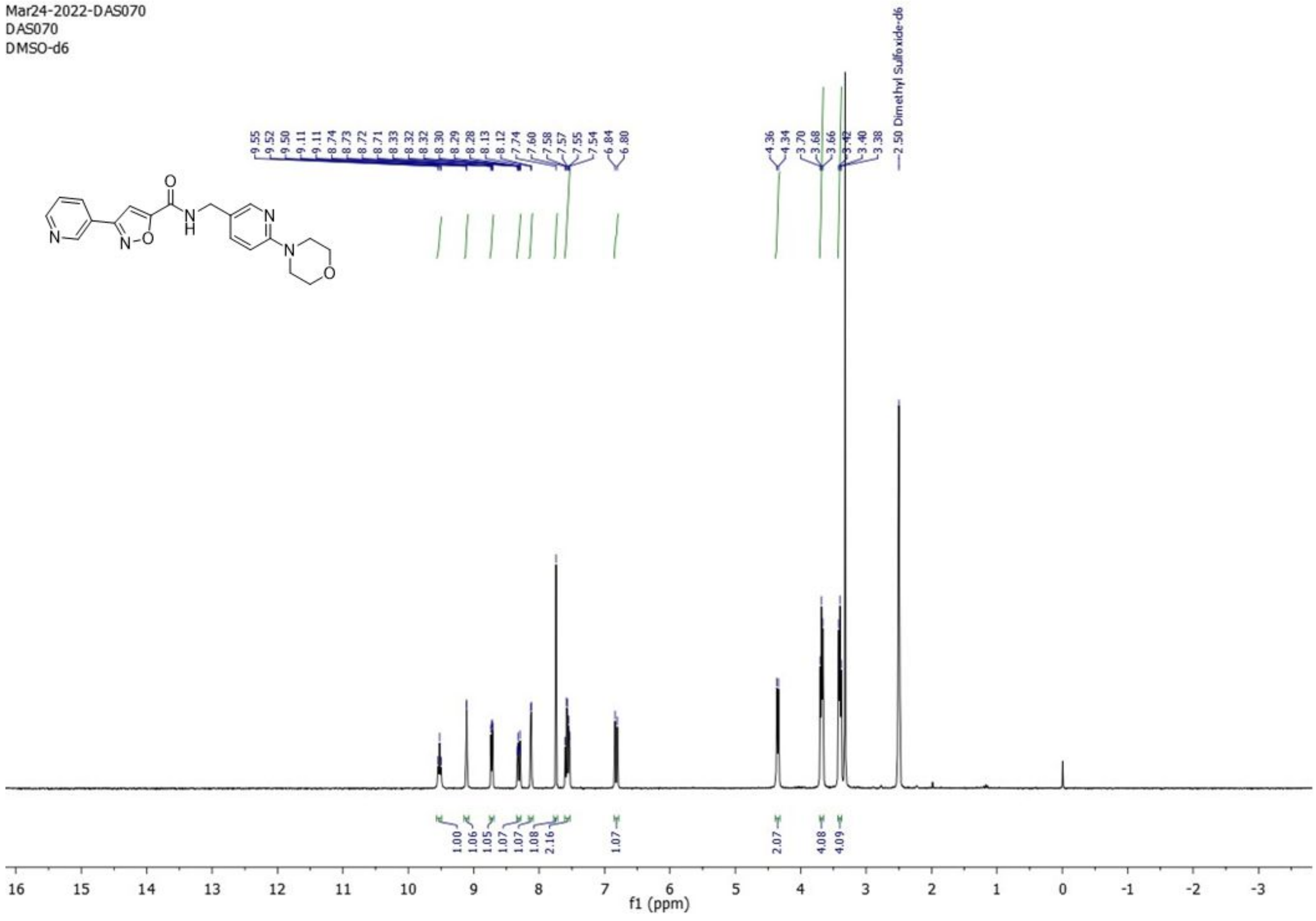

Figure S107: N-((6-morpholinopyridin-3-yl)methyl)-3-(pyridin-3-yl)isoxazole-5-carboxamide (93)

NMR spectra of additional compounds (*S1-S54*)

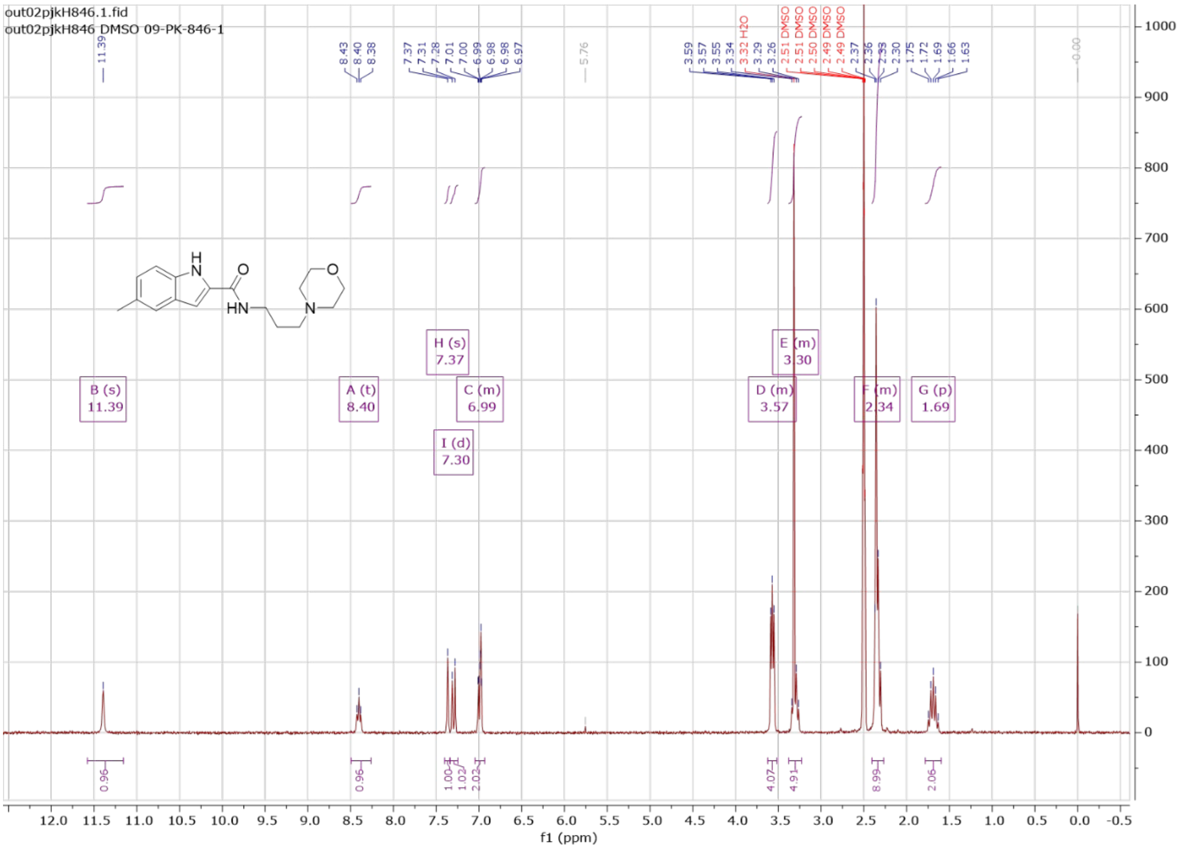

Figure S108: 5-methyl-N-(3-morpholinopropyl)-1H-indole-2-carboxamide (S1)

This report was created by ACD/NMR Processor Academic Edition. For more information go to www.acdlabs.com/nmrproc/

Acquisition Time (sec)	1.5903	Comment	Dessoy - MAD1192 - MeOD - Av 500 MHz - jan17madH1			Date	17 Jan 2020 13:41:20
File Name	\\nmrsparc.iqm.unicamp.br\espectros\avance500\2020\jan20\Sala\Luiz Carlos\jan17madH1\jan17madH1_001000fid					Frequency (MHz)	499.87
Nucleus	1H	Number of Transients	16	Original Points Count	16384	Points Count	16384
Pulse Sequence	zg30	Solvent	CD3OH	Spectrum Offset (Hz)	3072.2876	Sweep Width (Hz)	10302.20
Temperature (degree C)	25.152						

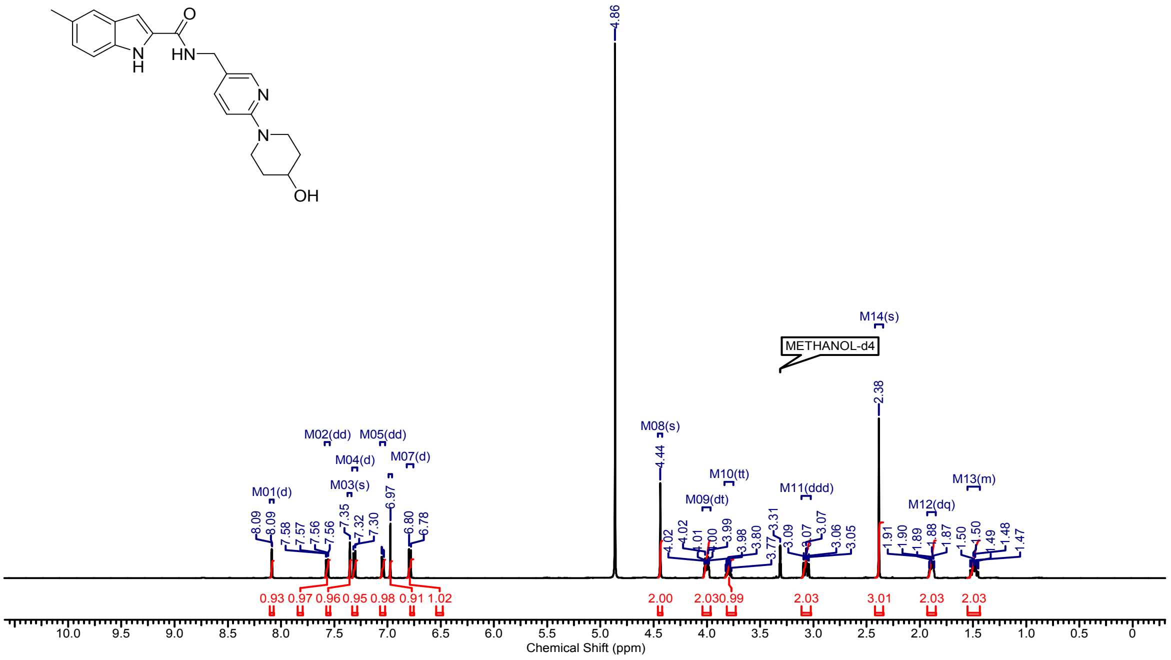

Figure S109: N-((6-(4-hydroxypiperidin-1-yl)pyridin-3-yl)methyl)-5-methyl-1H-indole-2-carboxamide (S2)

This report was created by ACD/NMR Processor Academic Edition. For more information go to www.acdlabs.com/nmrproc/

4/2/2022 7:13:08 PM

Acquisition Time (sec)	2.0494	Date	Apr 2 2022	Date Stamp	Apr 2 2022		
File Name	C:\Users\w540\Desktop\NMR_BOSTON\MDC-02-072-01_20220402_01\PROTON_03.fid\fid					Frequency (MHz)	499.66
Nucleus	1H	Number of Transients	64	Original Points Count	16384	Points Count	16384
Pulse Sequence	s2pul	Receiver Gain	60.00	Solvent	DMSO-d6	Spectrum Offset (Hz)	2498.2732
Spectrum Type	STANDARD	Sweep Width (Hz)	7994.40	Temperature (degree C)	25.000		

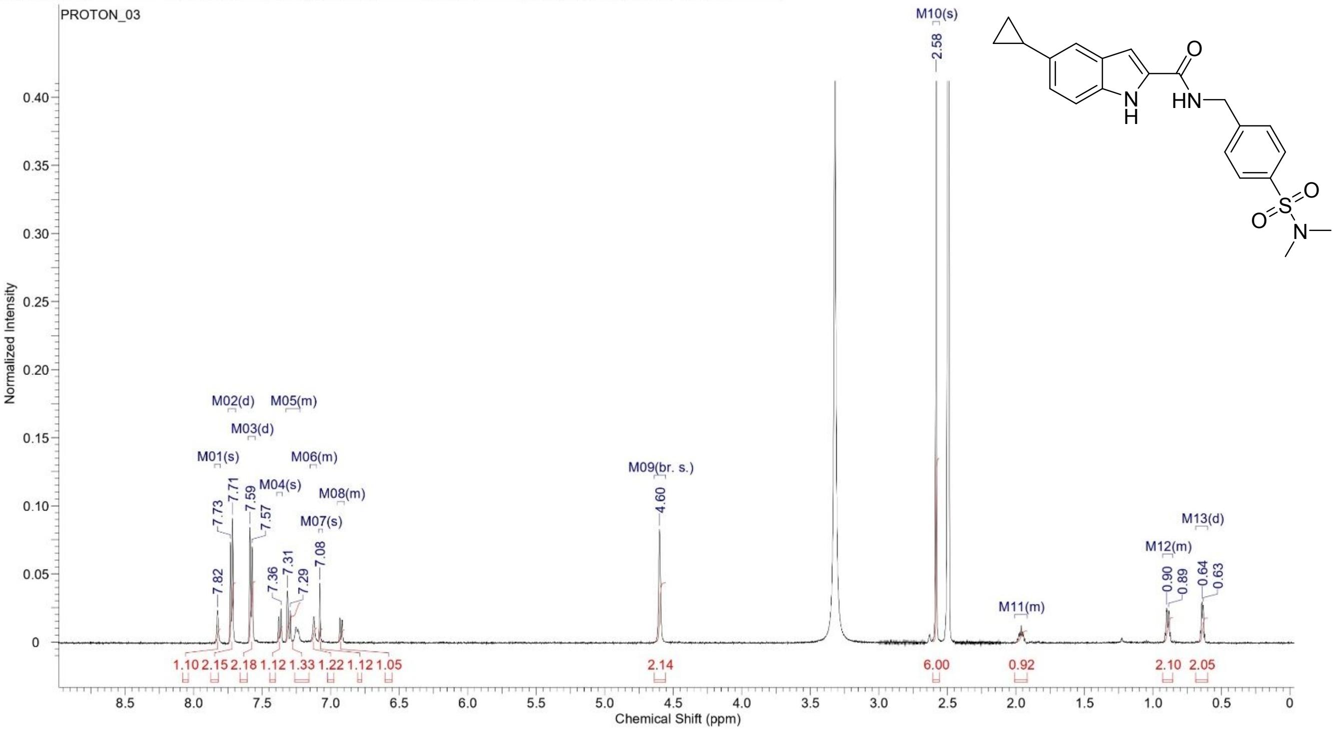

Figure S110: 5-cyclopropyl-N-(4-(N,N-dimethylsulfamoyl)benzyl)-1H-indole-2-carboxamide (S3)

This report was created by ACD/NMR Processor Academic Edition. For more information go to www.acdlabs.com/nmrproc/

2/25/2022 1:42:45 PM

Acquisition Time (sec)	2.0494	Date	Feb 25 2022	Date Stamp	Feb 25 2022		
File Name	C:\Users\w540\Desktop\NMR_BOSTON\MDC-02-049_20220225_01\PROTON_02.fid\fid					Frequency (MHz)	499.66
Nucleus	1H	Number of Transients	16	Original Points Count	16384	Points Count	16384
Pulse Sequence	s2pul	Receiver Gain	60.00	Solvent	DMSO-d6	Spectrum Offset (Hz)	2497.1294
Spectrum Type	STANDARD	Sweep Width (Hz)	7994.40	Temperature (degree C)	25.000		

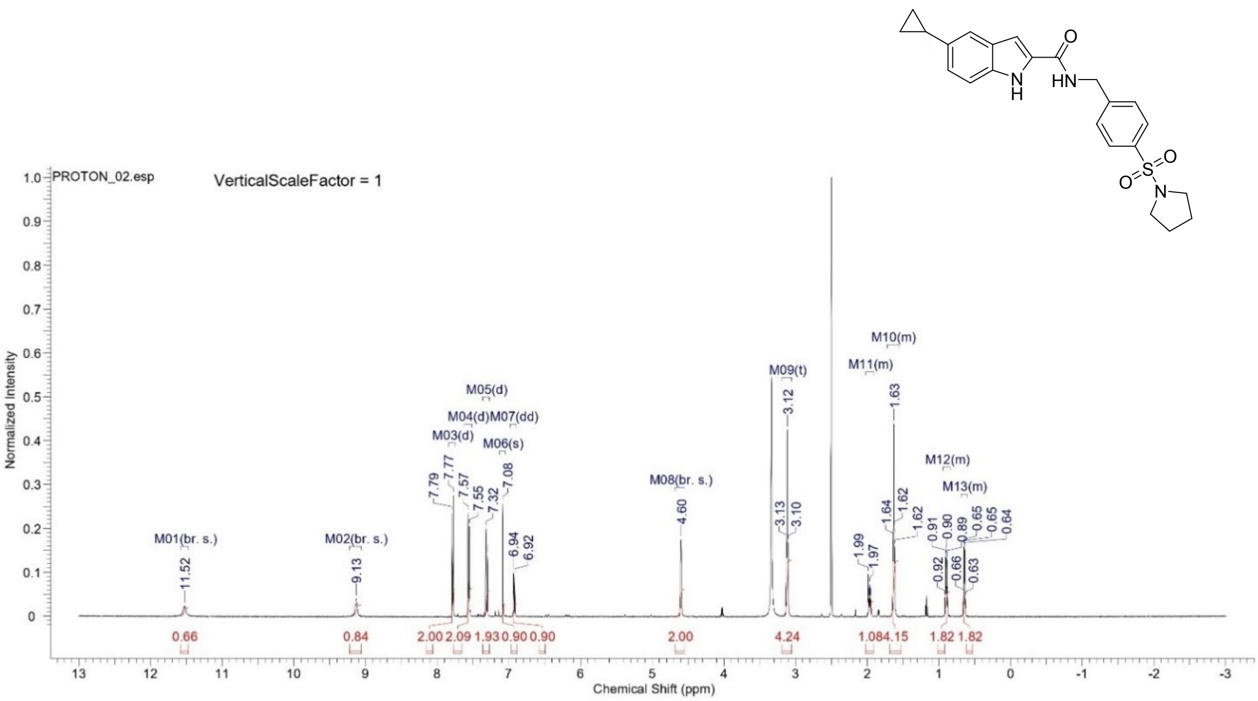

Figure S111: 5-cyclopropyl-N-(4-(pyrrolidin-1-ylsulfonyl)benzyl)-1H-indole-2-carboxamide (S4)

This report was created by ACD/NMR Processor Academic Edition. For more information go to www.acdlabs.com/nmrproc/

2/25/2022 1:43:36 PM

**Acquisition Time (sec)**	2.0494	**Date**	Feb 25 2022	**Date Stamp**	Feb 25 2022		
**File Name**	C:\Users\w540\Desktop\NMR_BOSTON\MDC-02-050_20220225_01\PROTON_02.fid\fid					**Frequency (MHz)**	499.66
**Nucleus**	1H	**Number of Transients**	16	**Original Points Count**	16384	**Points Count**	16384
**Pulse Sequence**	s2pul	**Receiver Gain**	60.00	**Solvent**	DMSO-d6	**Spectrum Offset (Hz)**	2497.1294
**Spectrum Type**	STANDARD	**Sweep Width (Hz)**	7994.40	**Temperature (degree C)**	25.000		

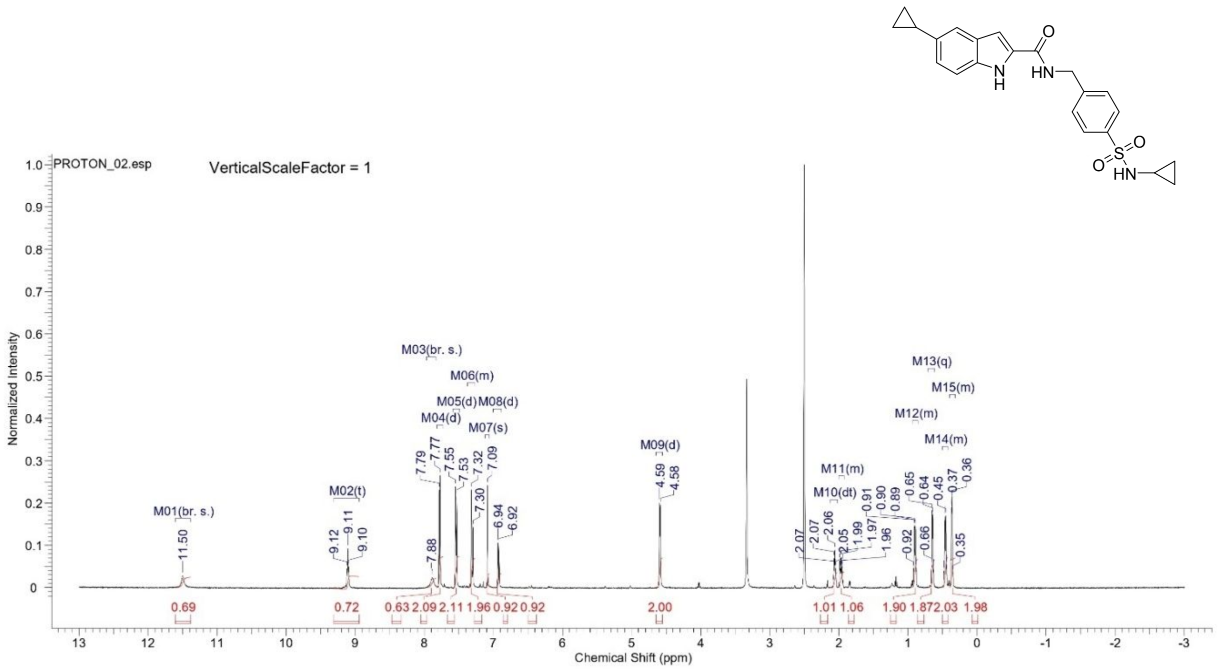

Figure S112: 5-cyclopropyl-N-(4-(N-cyclopropylsulfamoyl)benzyl)-1H-indole-2-carboxamide (S5)

This report was created by ACD/NMR Processor Academic Edition. For more information go to www.acdlabs.com/nmrproc/

QJS-08-050

7/16/2022 12:04:49 PM

**Acquisition Time (sec)**	2.0494	**Date**	Jan 27 2022	**Date Stamp**	Jan 27 2022		
**File Name**	C:\Users\w540\Desktop\NMR_BOSTON\QJS-08-050_20220127_01\PROTON_02.fid\fid					**Frequency (MHz)**	499.66
**Nucleus**	1H	**Number of Transients**	64	**Original Points Count**	16384	**Points Count**	16384
**Pulse Sequence**	s2pul	**Receiver Gain**	60.00	**Solvent**	CHLOROFORM-d		
**Spectrum Offset (Hz)**	2493.8267	**Spectrum Type**	STANDARD	**Sweep Width (Hz)**	7994.40	**Temperature (degree C)**	25.000

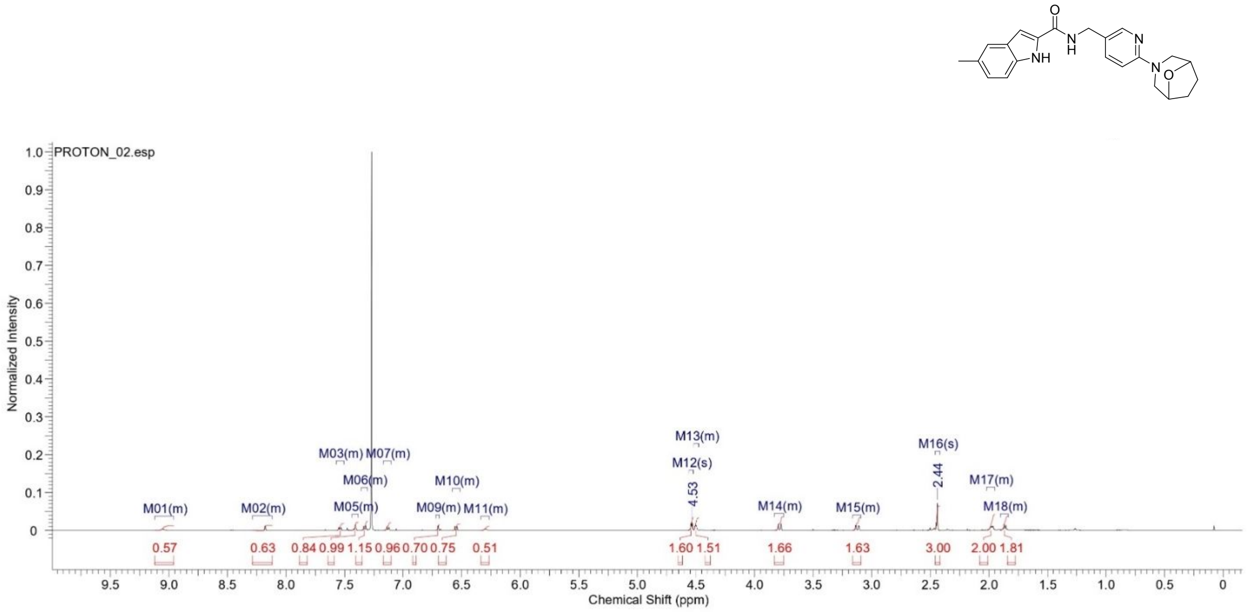

Figure S113: N-((6-(8-oxa-3-azabicyclo[3.2.1]octan-3-yl)pyridin-3-yl)methyl)-5-methyl-1H-indole-2-carboxamide (S6)

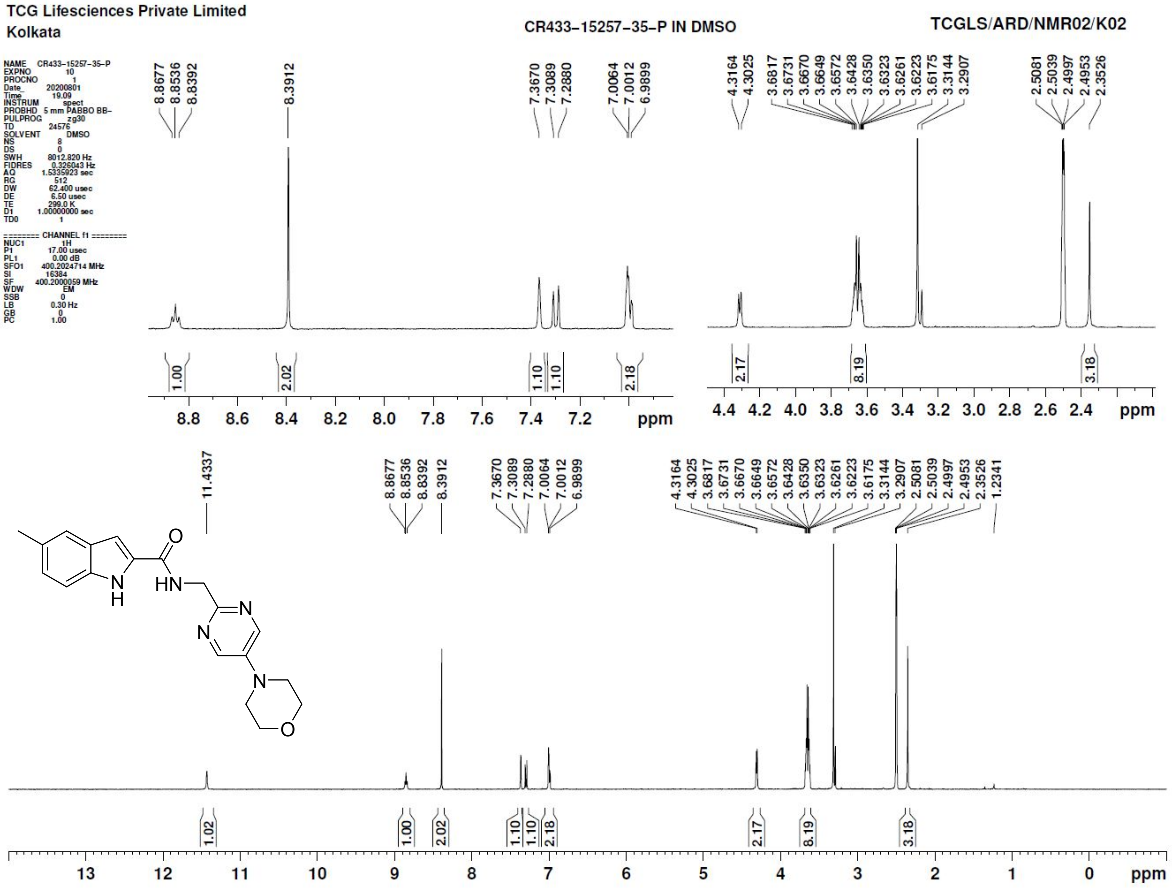

Figure S114: 5-methyl-N-((5-morpholinopyrimidin-2-yl)methyl)-1H-indole-2-carboxamide (S7)

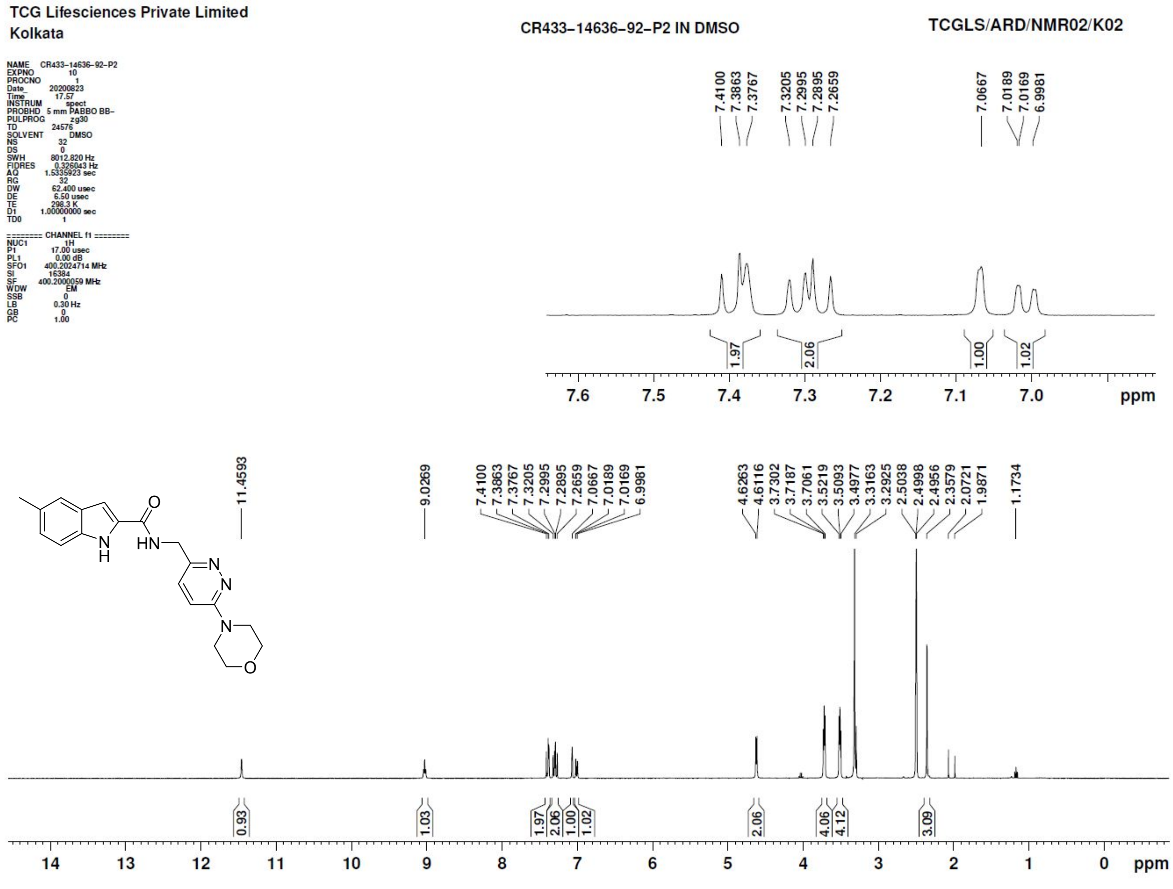

Figure S115: 5-methyl-N-((6-morpholinopyridazin-3-yl)methyl)-1H-indole-2-carboxamide (S8)

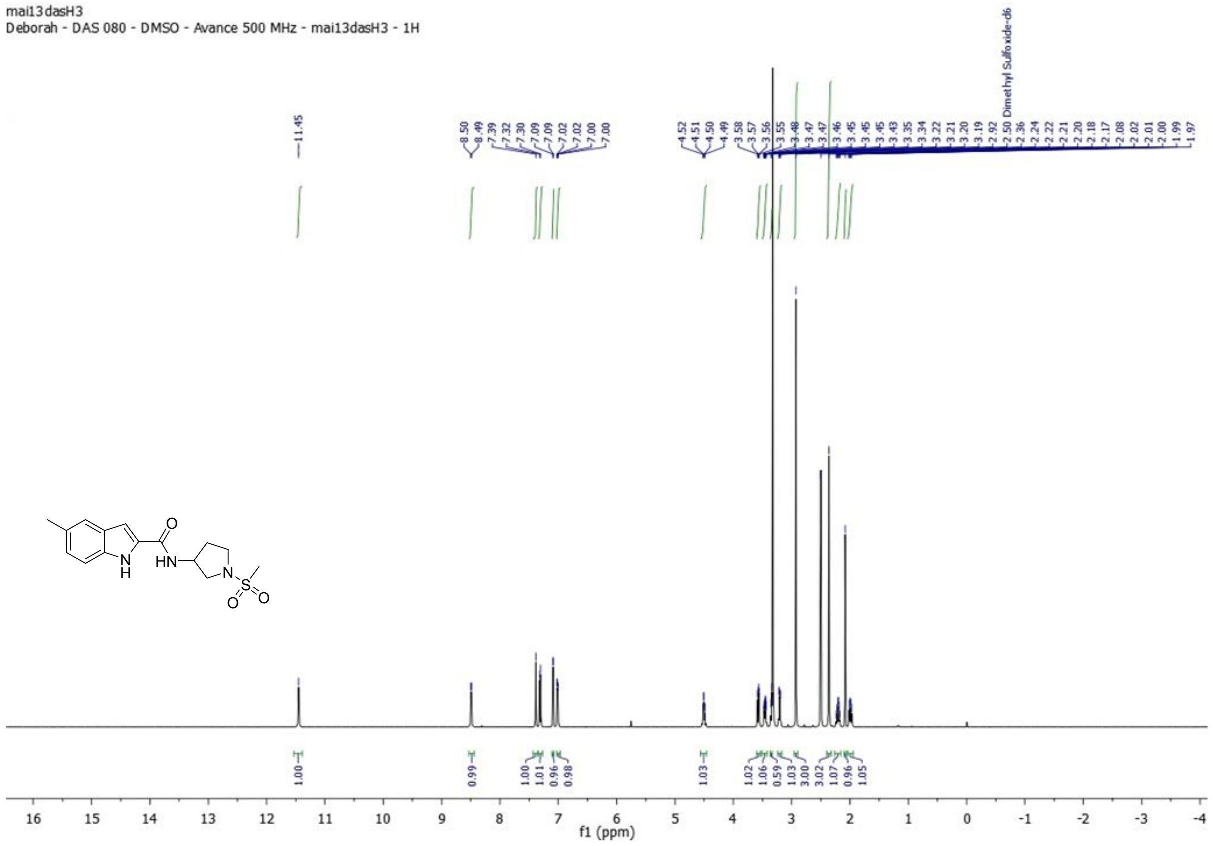

Figure S116: 5-methyl-N-((1-(methylsulfonyl)pyrrolidin-3-yl)methyl)-1H-indole-2-carboxamide (S9)

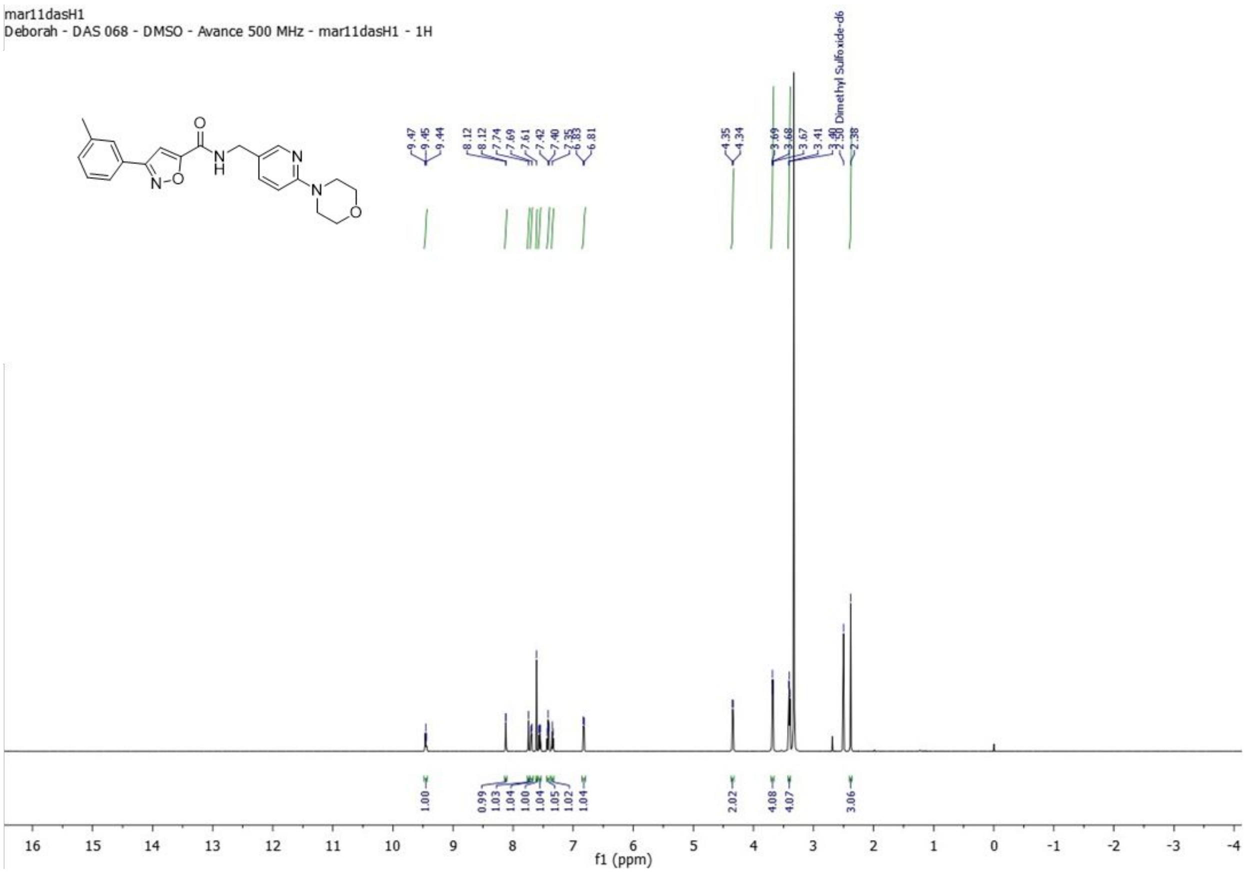

Figure S117: N-((6-morpholinopyridin-3-yl)methyl)-3-(m-tolyl)isoxazole-5-carboxamide (S10)

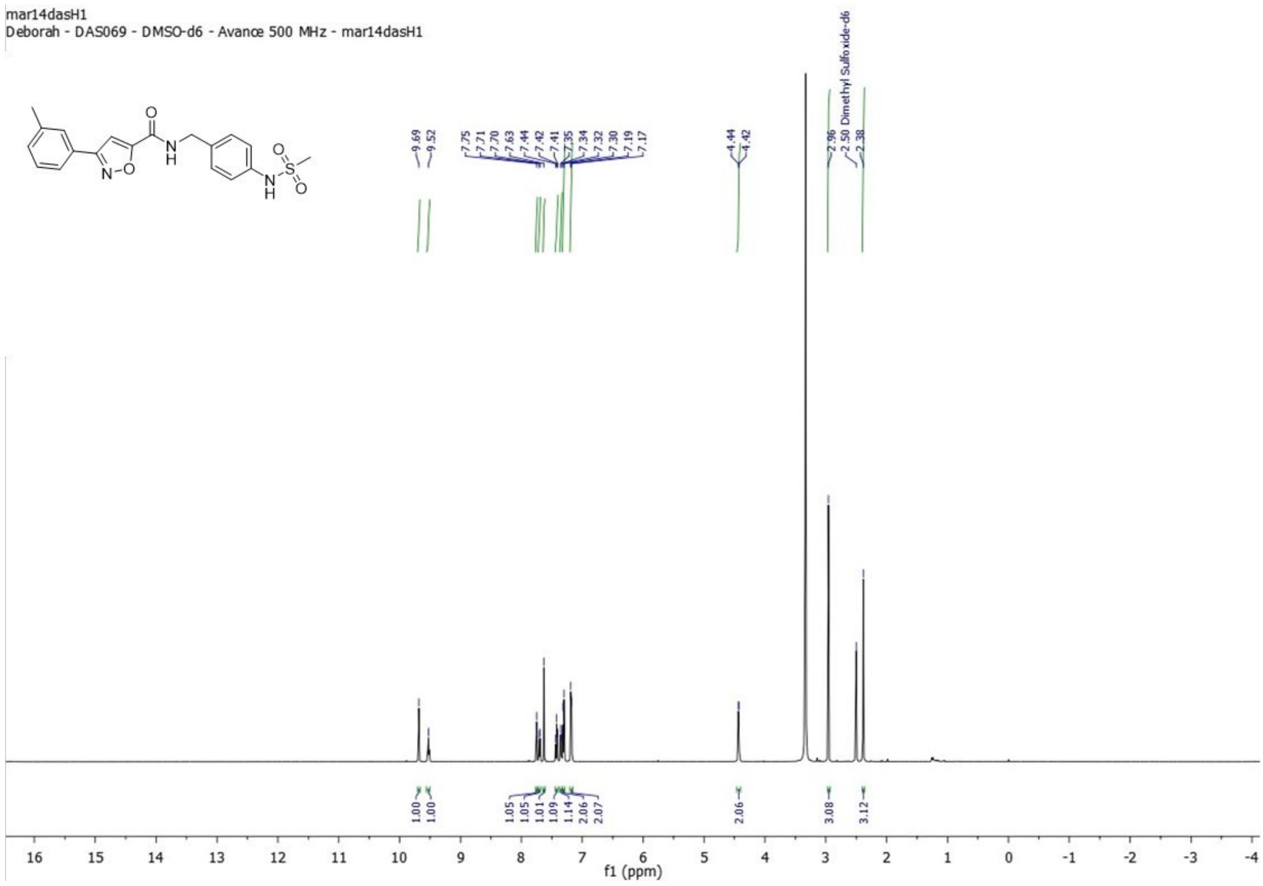

Figure S118: N-(4-(methylsulfonamido)benzyl)-3-(m-tolyl)isoxazole-5-carboxamide (S11)

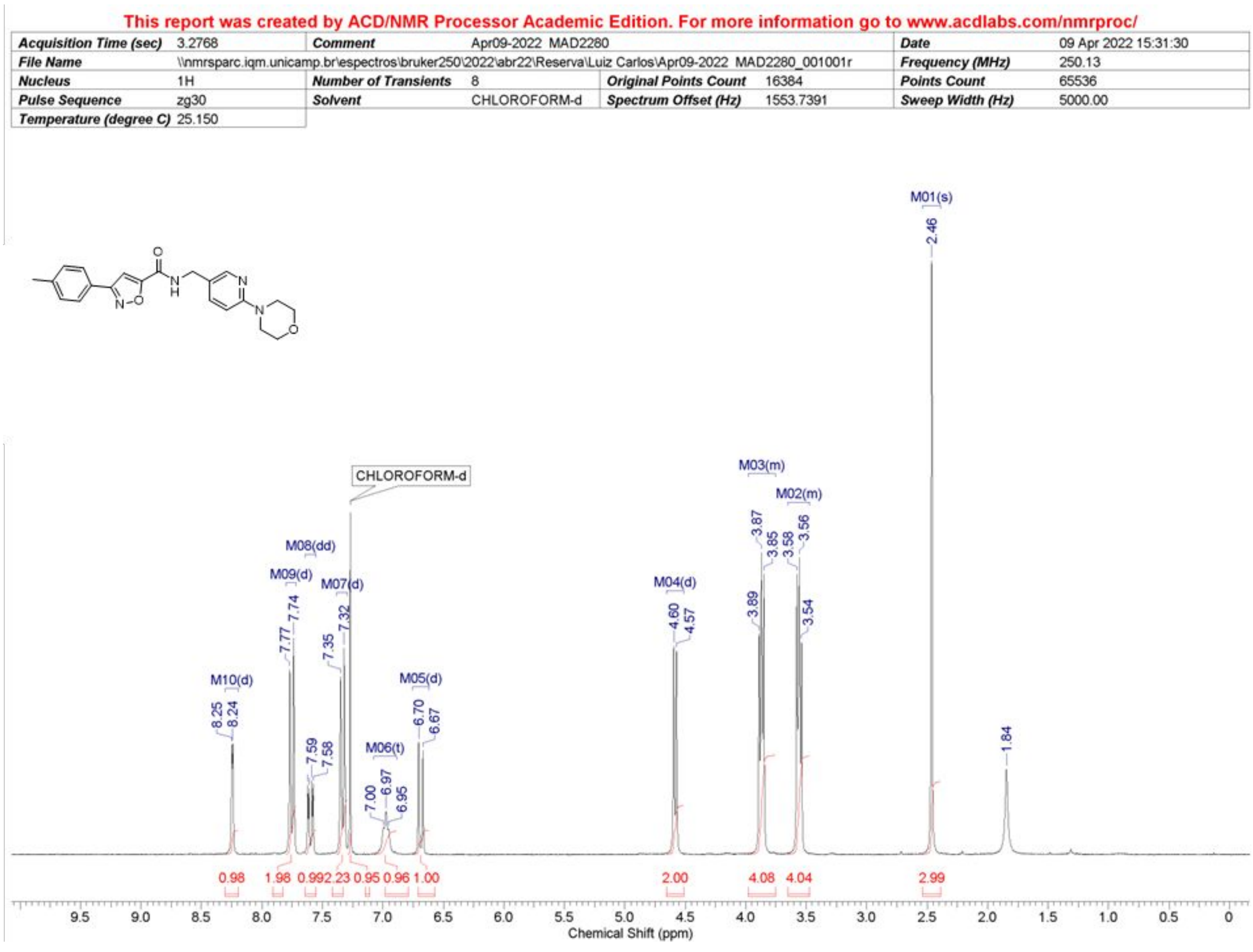

Figure S119: N-((6-morpholinopyridin-3-yl)methyl)-3-(p-tolyl)isoxazole-5-carboxamide (S12)

This report was created by ACD/NMR Processor Academic Edition. For more information go to www.acdlabs.com/nmrproc/

Acquisition Time (sec)	3.1807	Comment	mad2284-dmso	Date	25 Apr 2022 11:10:22		
File Name	\\nmrsparc.iqm.unicamp.br\espectros\avance500\2022\abr22\Reserva\Luiz Carlos\mad2284_001001r					Frequency (MHz)	499.87
Nucleus	1H	Number of Transients	16	Original Points Count	32768	Points Count	65536
Pulse Sequence	zg30	Solvent	DMSO-d6	Spectrum Offset (Hz)	3082.8799	Sweep Width (Hz)	10302.20
Temperature (degree C)	25.146						

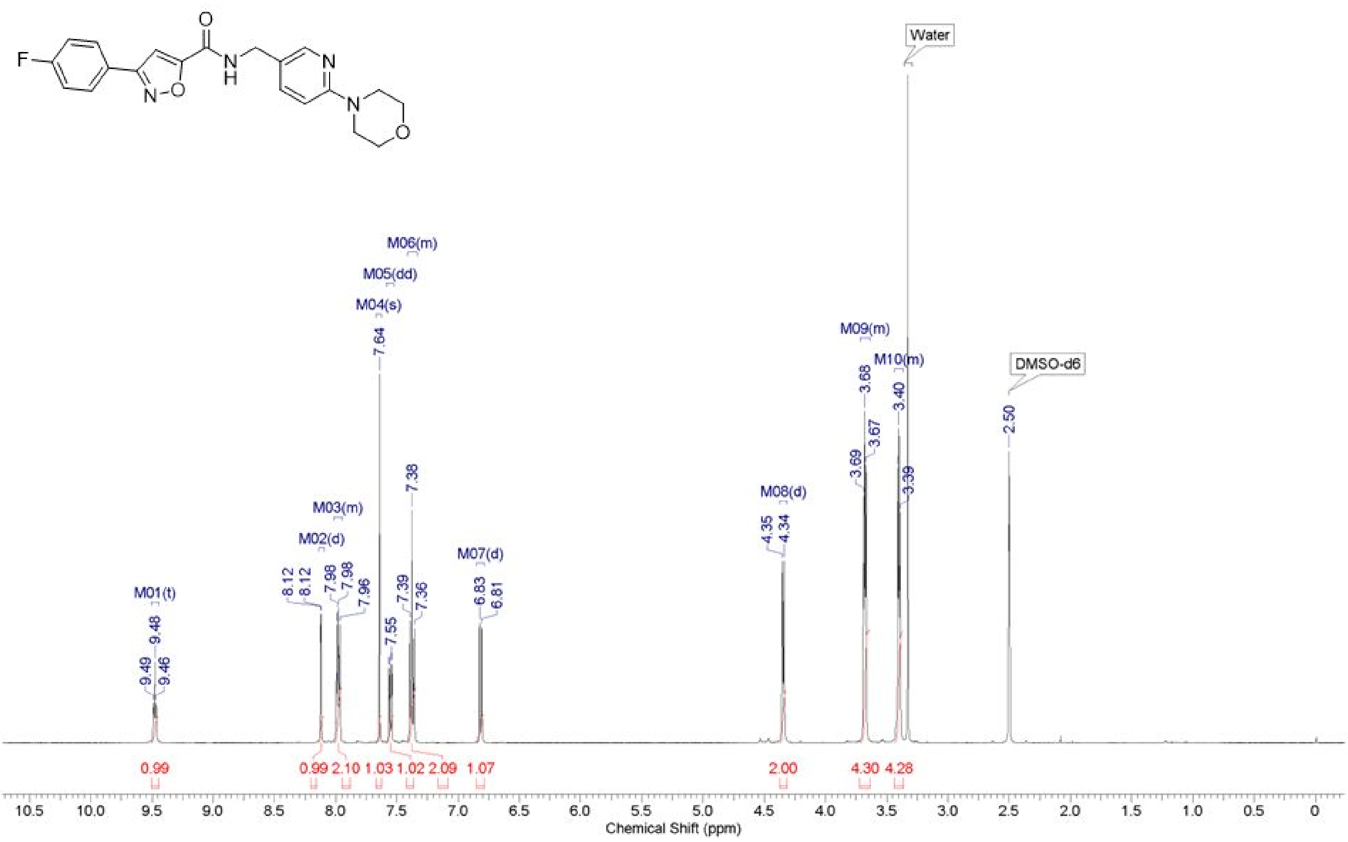

Figure S120: 3-(4-fluorophenyl)-N-((6-morpholinopyridin-3-yl)methyl)isoxazole-5-carboxamide (S13)

This report was created by ACD/NMR Processor Academic Edition. For more information go to www.acdlabs.com/nmrproc/

MDC-02-088

5/7/2022 1:06:25 PM

Acquisition Time (sec)	2.0494	Date	Apr 28 2022	Date Stamp	Apr 28 2022		
File Name	C:\Users\w540\Desktop\NMR_BOSTON\MDC-02-088_20220428_01\PROTON_01.fid\fid					Frequency (MHz)	499.66
Nucleus	1H	Number of Transients	16	Original Points Count	16384	Points Count	16384
Pulse Sequence	s2pul	Receiver Gain	54.00	Solvent	CHLOROFORM-d		
Spectrum Offset (Hz)	2496.2666	Spectrum Type	STANDARD	Sweep Width (Hz)	7994.40	Temperature (degree C)	25.000

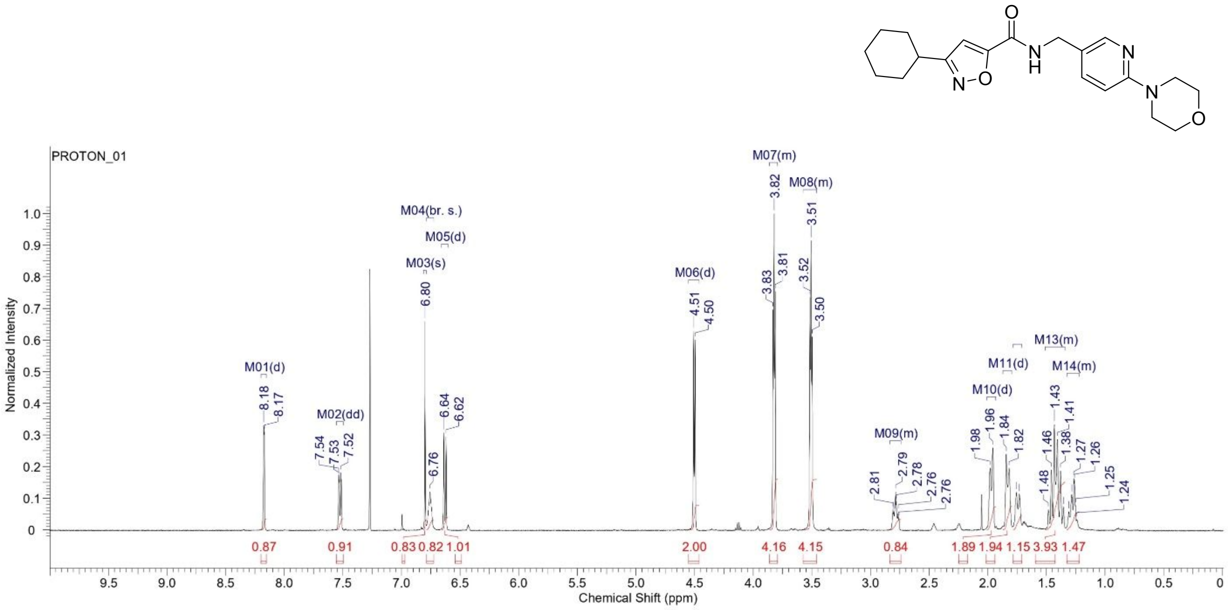

Figure S121: 3-cyclohexyl-N-((6-morpholinopyridin-3-yl)methyl)isoxazole-5-carboxamide (S14)

This report was created by ACD/NMR Processor Academic Edition. For more information go to www.acdlabs.com/nmrproc/

QJS-08-052

7/16/2022 10:25:51 AM

Acquisition Time (sec)	2.0494	Date	Jan 26 2022	Date Stamp	Jan 26 2022		
File Name	C:\Users\w540\Desktop\NMR_BOSTON\QJS-08-052_20220126_01\PROTON_01.fid\fid					Frequency (MHz)	499.66
Nucleus	1H	Number of Transients	8	Original Points Count	16384	Points Count	16384
Pulse Sequence	s2pul	Receiver Gain	54.00	Solvent	CHLOROFORM-d		
Spectrum Offset (Hz)	2493.8267	Spectrum Type	STANDARD	Sweep Width (Hz)	7994.40	Temperature (degree C)	25.000

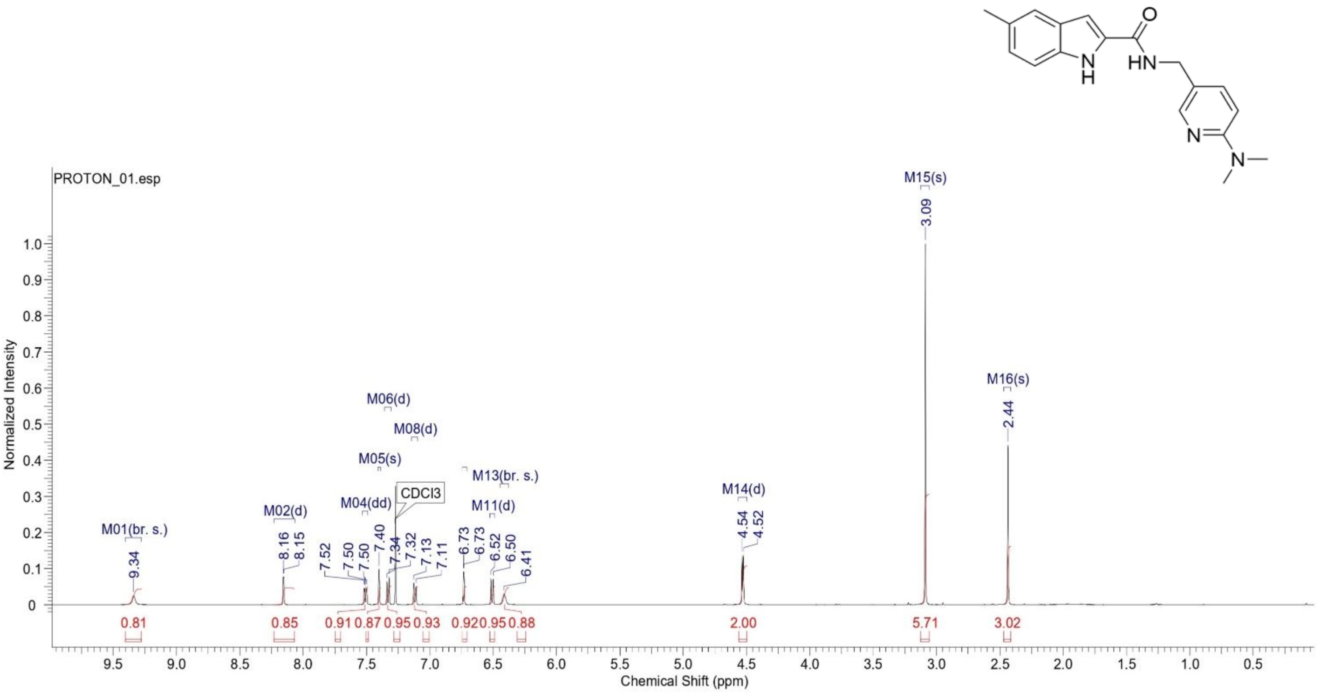

Figure S122: N-((6-(dimethylamino)pyridin-3-yl)methyl)-5-methyl-1H-indole-2-carboxamide (S15)

This report was created by ACD/NMR Processor Academic Edition. For more information go to www.acdlabs.com/nmrproc/

MDC-02-080

7/16/2022 3:25:26 PM

Acquisition Time (sec)	2.0494	Date	Apr 11 2022	Date Stamp	Apr 11 2022				
File Name	C:\Users\w540\Desktop\NMR_BOSTON\NEU-66_20220411_01\PROTON_02.fid\fid					Frequency (MHz)	499.66	Nucleus	1H
Number of Transients	64	Original Points Count	16384	Points Count	16384	Pulse Sequence	s2pul	Receiver Gain	60.00
Solvent	DMSO-d6	Spectrum Offset (Hz)	2500.5452	Spectrum Type	STANDARD	Sweep Width (Hz)	7994.40	Temperature (degree C)	25.000

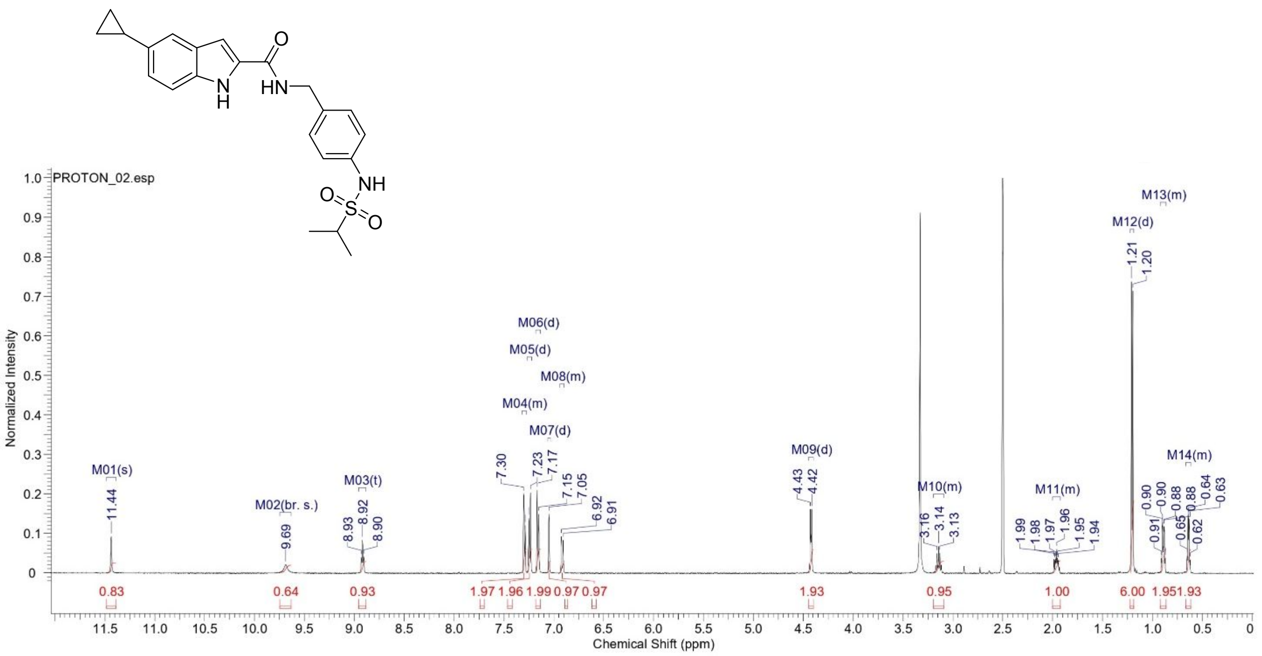

Figure S123: 5-cyclopropyl-N-(4-((1-methylethyl)sulfonamido)benzyl)-1H-indole-2-carboxamide (S16)

This report was created by ACD/NMR Processor Academic Edition. For more information go to www.acdlabs.com/nmrproc/

MDC-02-092

7/16/2022 2:10:48 PM

**Acquisition Time (sec)**	2.0494	**Date**	May 2 2022	**Date Stamp**	May 2 2022				
**File Name**	C:\Users\w540\Desktop\NMR_BOSTON\NEU-67_20220502_01\PROTON_03.fid\fid					**Frequency (MHz)**	499.66	**Nucleus**	1H
**Number of Transients**	128	**Original Points Count**	16384	**Points Count**	16384	**Pulse Sequence**	s2pul	**Receiver Gain**	60.00
**Solvent**	DMSO-d6	**Spectrum Offset (Hz)**	2500.5452	**Spectrum Type**	STANDARD	**Sweep Width (Hz)**	7994.40	**Temperature (degree C)**	25.000

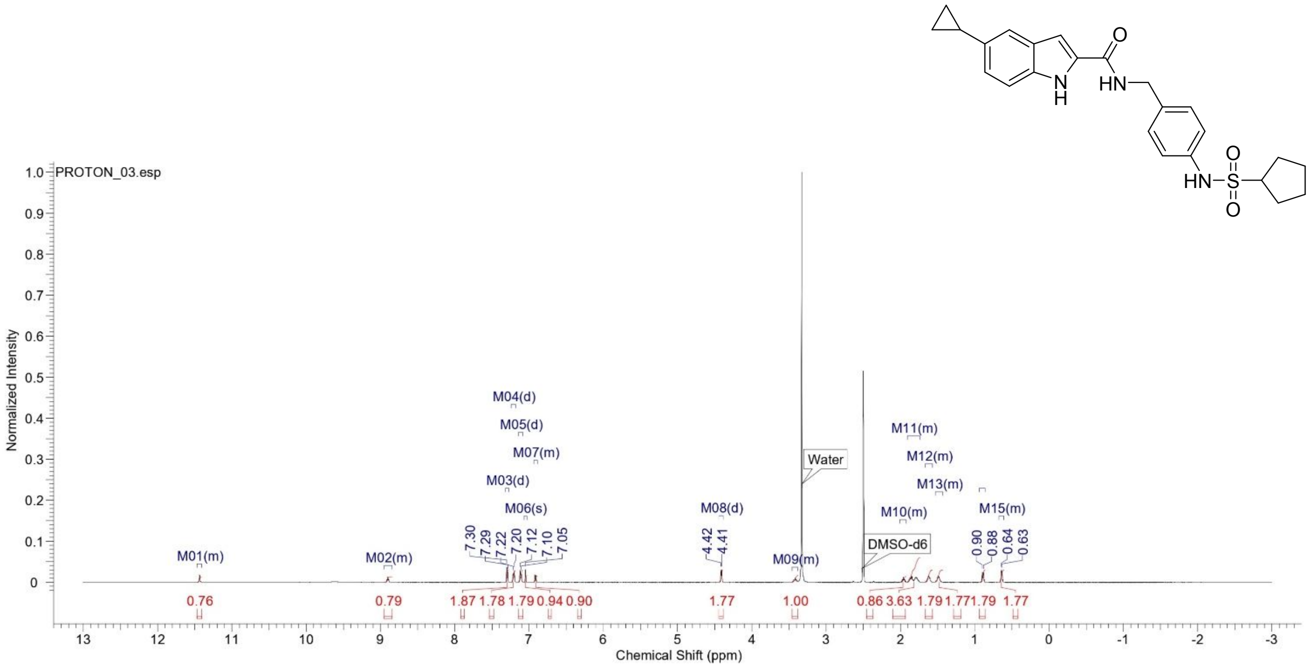

Figure S124: N-(4-(cyclopentanesulfonamido)benzyl)-5-cyclopropyl-1H-indole-2-carboxamide (S17)

This report was created by ACD/NMR Processor Academic Edition. For more information go to www.acdlabs.com/nmrproc/

MDC-02-114

7/18/2022 9:18:51 PM

**Acquisition Time (sec)**	2.0494	**Date**	Jun 30 2022	**Date Stamp**	Jun 30 2022		
**File Name**	C:\Users\w540\Desktop\NMR_BOSTON\MDC-02-114_20220630_01\PROTON_01.fid\fid					**Frequency (MHz)**	499.66
**Nucleus**	1H	**Number of Transients**	4	**Original Points Count**	16384	**Points Count**	16384
**Pulse Sequence**	s2pul	**Receiver Gain**	60.00	**Solvent**	METHANOL-d4		
**Spectrum Offset (Hz)**	2492.9333	**Spectrum Type**	STANDARD	**Sweep Width (Hz)**	7994.40	**Temperature (degree C)**	25.000

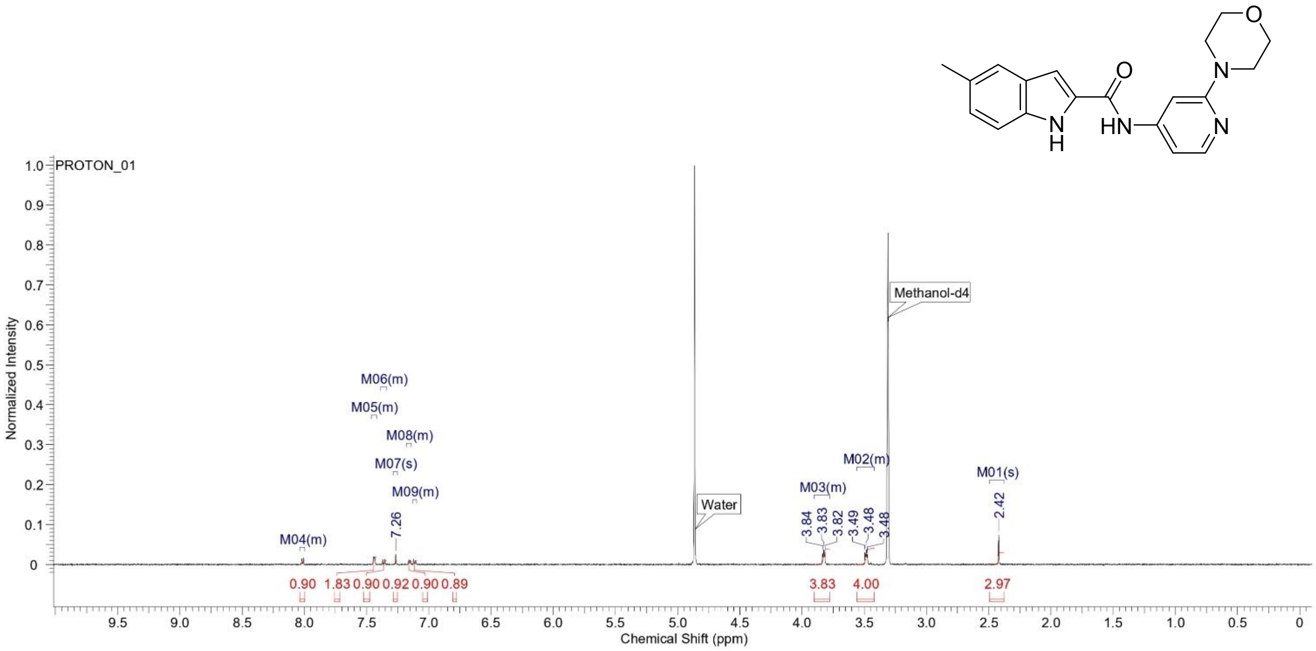

Figure S125: 5-methyl-N-(2-morpholinopyridin-4-yl)-1H-indole-2-carboxamide (S18)

This report was created by ACD/NMR Processor Academic Edition. For more information go to www.acdlabs.com/nmrproc/

MDC-02-098

7/16/2022 3:48:14 PM

Acquisition Time (sec)	2.0494	*Date*	Jun 2 2022	*Date Stamp*	Jun 2 2022		
File Name	C:\Users\w540\Desktop\NMR_BOSTON\MDC-02-098-03_20220602_01\PROTON_03.fid\fid					*Frequency (MHz)*	499.66
Nucleus	1H	*Number of Transients*	32	*Original Points Count*	16384	*Points Count*	16384
Pulse Sequence	s2pul	*Receiver Gain*	54.00	*Solvent*	acetone	*Spectrum Offset (Hz)*	2491.3813
Spectrum Type	STANDARD	*Sweep Width (Hz)*	7994.40	*Temperature (degree C)*	25.000		

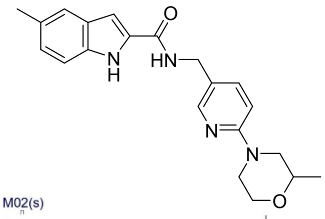

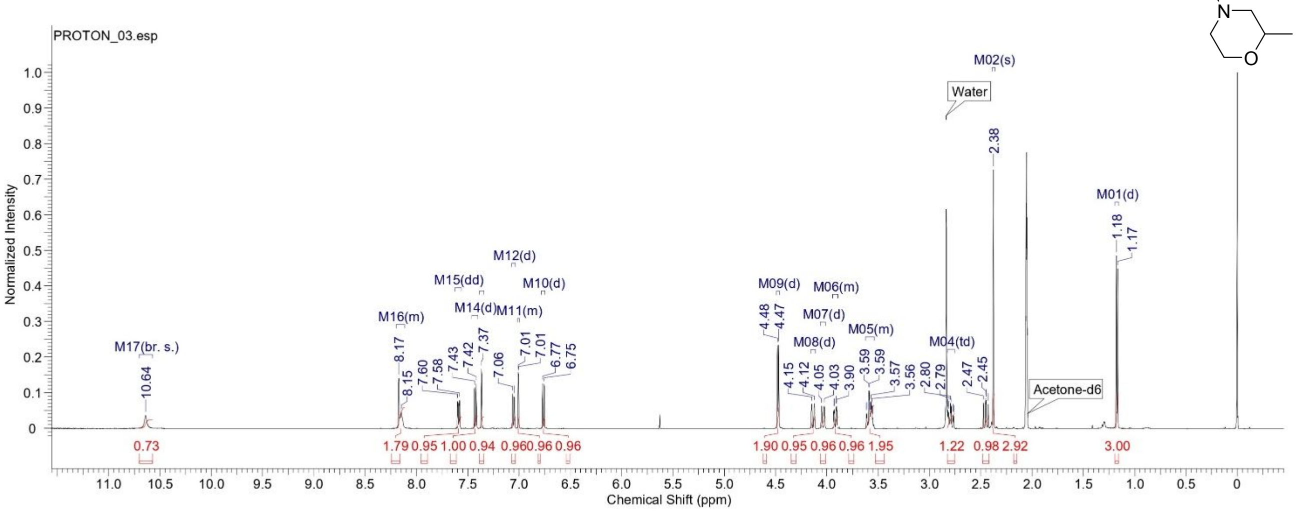

Figure S126: 5-methyl-N-((6-(2-methylmorpholino)pyridin-3-yl)methyl)-1H-indole-2-carboxamide (S19)

This report was created by ACD/NMR Processor Academic Edition. For more information go to www.acdlabs.com/nmrproc/

MDC-02-101FP

7/16/2022 10:00:09 PM

Acquisition Time (sec)	2.0494	*Date*	Jun 7 2022	*Date Stamp*	Jun 7 2022		
File Name	C:\Users\w540\Desktop\NMR_BOSTON\MDC-02-101-FP_20220607_01\PROTON_02.fid\fid					*Frequency (MHz)*	499.66
Nucleus	1H	*Number of Transients*	16	*Original Points Count*	16384	*Points Count*	16384
Pulse Sequence	s2pul	*Receiver Gain*	60.00	*Solvent*	acetone	*Spectrum Offset (Hz)*	2489.9177
Spectrum Type	STANDARD	*Sweep Width (Hz)*	7994.40	*Temperature (degree C)*	25.000		

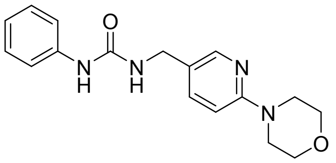

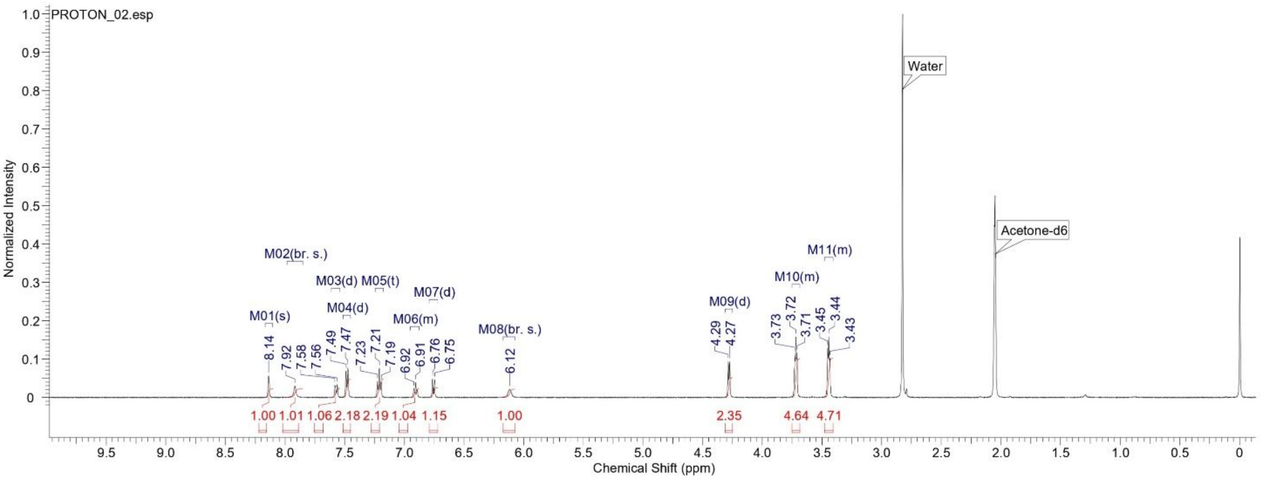

Figure S127: 1-((6-morpholinopyridin-3-yl)methyl)-3-phenylurea (S20)

This report was created by ACD/NMR Processor Academic Edition. For more information go to www.acdlabs.com/nmrproc/

MDC-02-118-02

7/21/2022 4:55:39 PM

**Acquisition Time (sec)**	2.0494	**Date**	Jul 20 2022	**Date Stamp**	Jul 20 2022		
**File Name**	C:\Users\w540\Desktop\NMR_BOSTON\MDC-02-118-02_20220720_01\PROTON_01.fid\fid					**Frequency (MHz)**	499.66
**Nucleus**	1H	**Number of Transients**	32	**Original Points Count**	16384	**Points Count**	16384
**Pulse Sequence**	s2pul	**Receiver Gain**	54.00	**Solvent**	METHANOL-d4		
**Spectrum Offset (Hz)**	2492.9333	**Spectrum Type**	STANDARD	**Sweep Width (Hz)**	7994.40	**Temperature (degree C)**	25.000

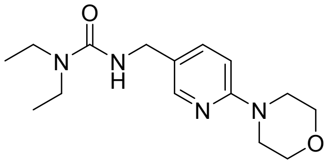

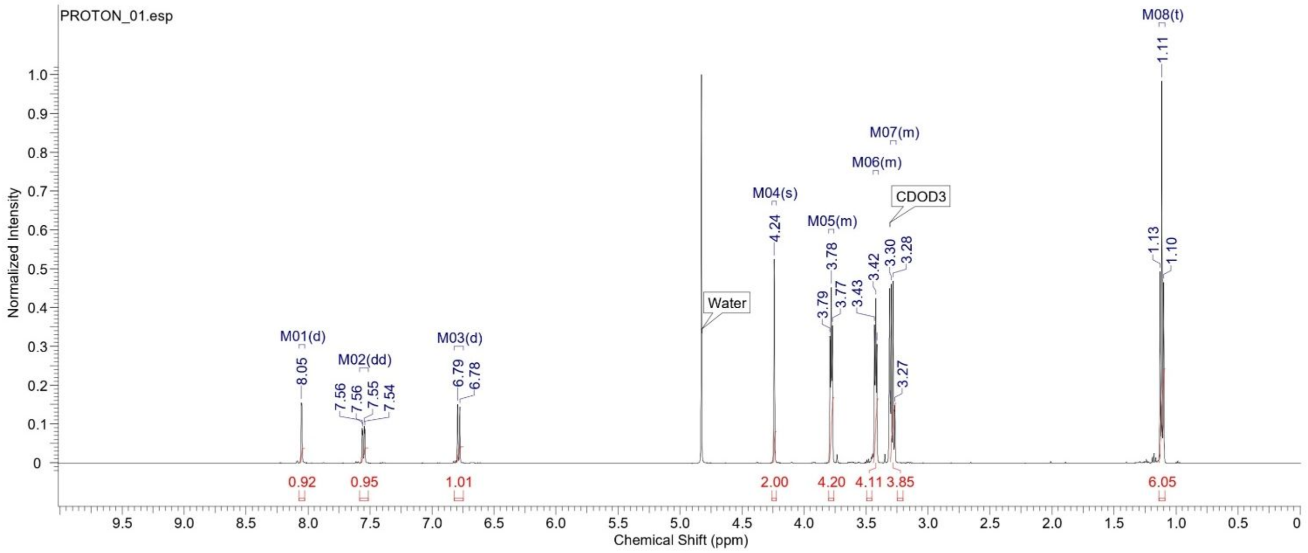

Figure S128: 1,1-diethyl-3-((6-morpholinopyridin-3-yl)methyl)urea (S21)

This report was created by ACD/NMR Processor Academic Edition. For more information go to www.acdlabs.com/nmrproc/

MDC-02-112-02

7/18/2022 9:08:48 PM

**Acquisition Time (sec)**	2.0494	**Date**	Jul 14 2022	**Date Stamp**	Jul 14 2022		
**File Name**	C:\Users\w540\Desktop\NMR_BOSTON\MDC-02-112-02_20220714_01\PROTON_01.fid\fid					**Frequency (MHz)**	499.66
**Nucleus**	1H	**Number of Transients**	32	**Original Points Count**	16384	**Points Count**	16384
**Pulse Sequence**	s2pul	**Receiver Gain**	60.00	**Solvent**	acetone	**Spectrum Offset (Hz)**	2489.4294
**Spectrum Type**	STANDARD	**Sweep Width (Hz)**	7994.40	**Temperature (degree C)**	25.000		

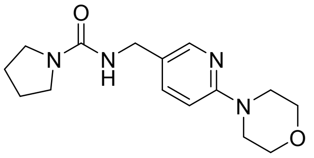

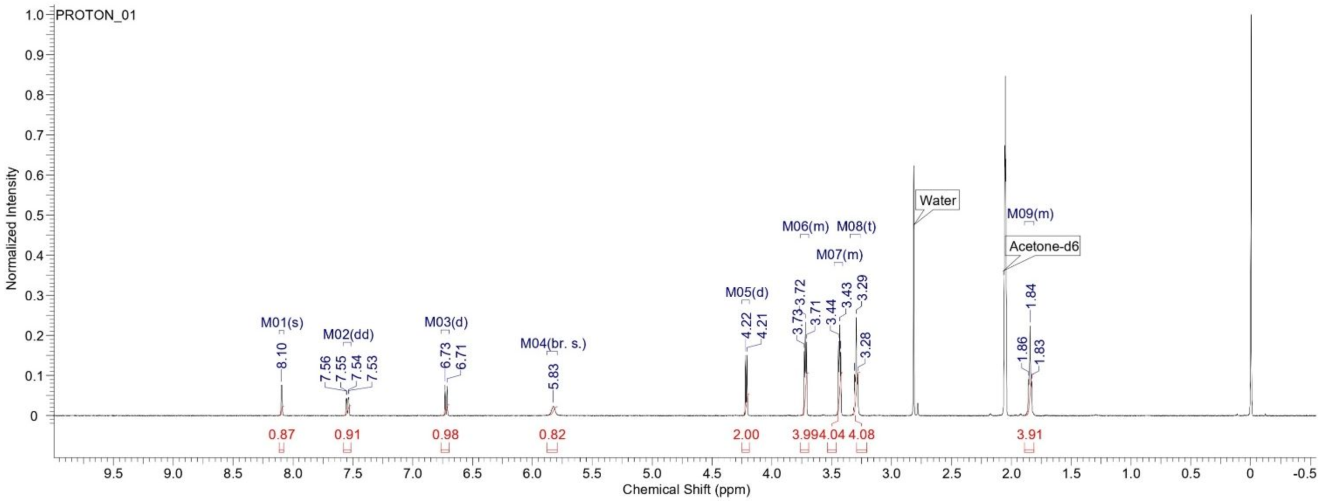

Figure S129: N-((6-morpholinopyridin-3-yl)methyl)pyrrolidine-1-carboxamide (S22)

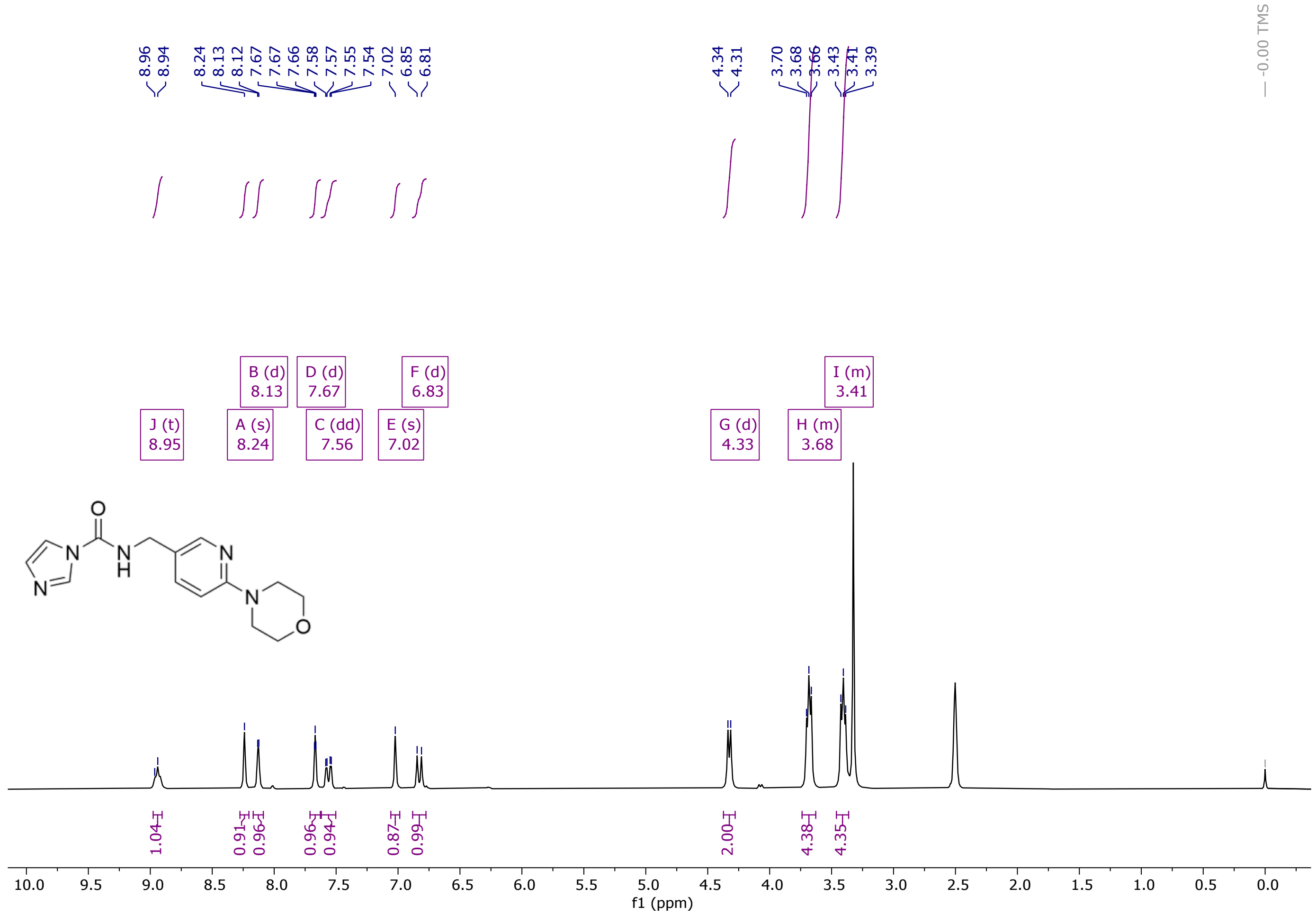

Figure S130: N-((6-morpholinopyridin-3-yl)methyl)-1H-imidazole-1-carboxamide (S23)

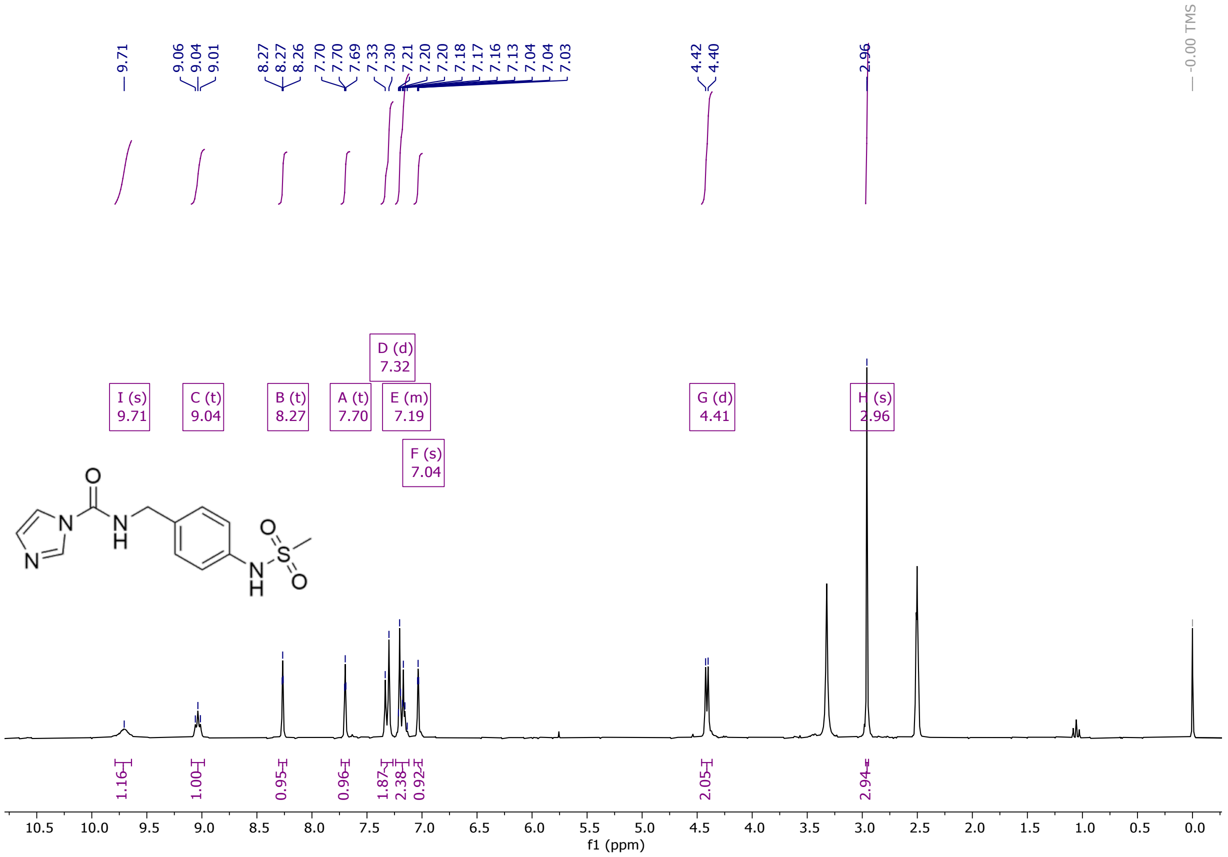

Figure S131: N-(4-(methylsulfonamido)benzyl)-1H-imidazole-1-carboxamide (S24)

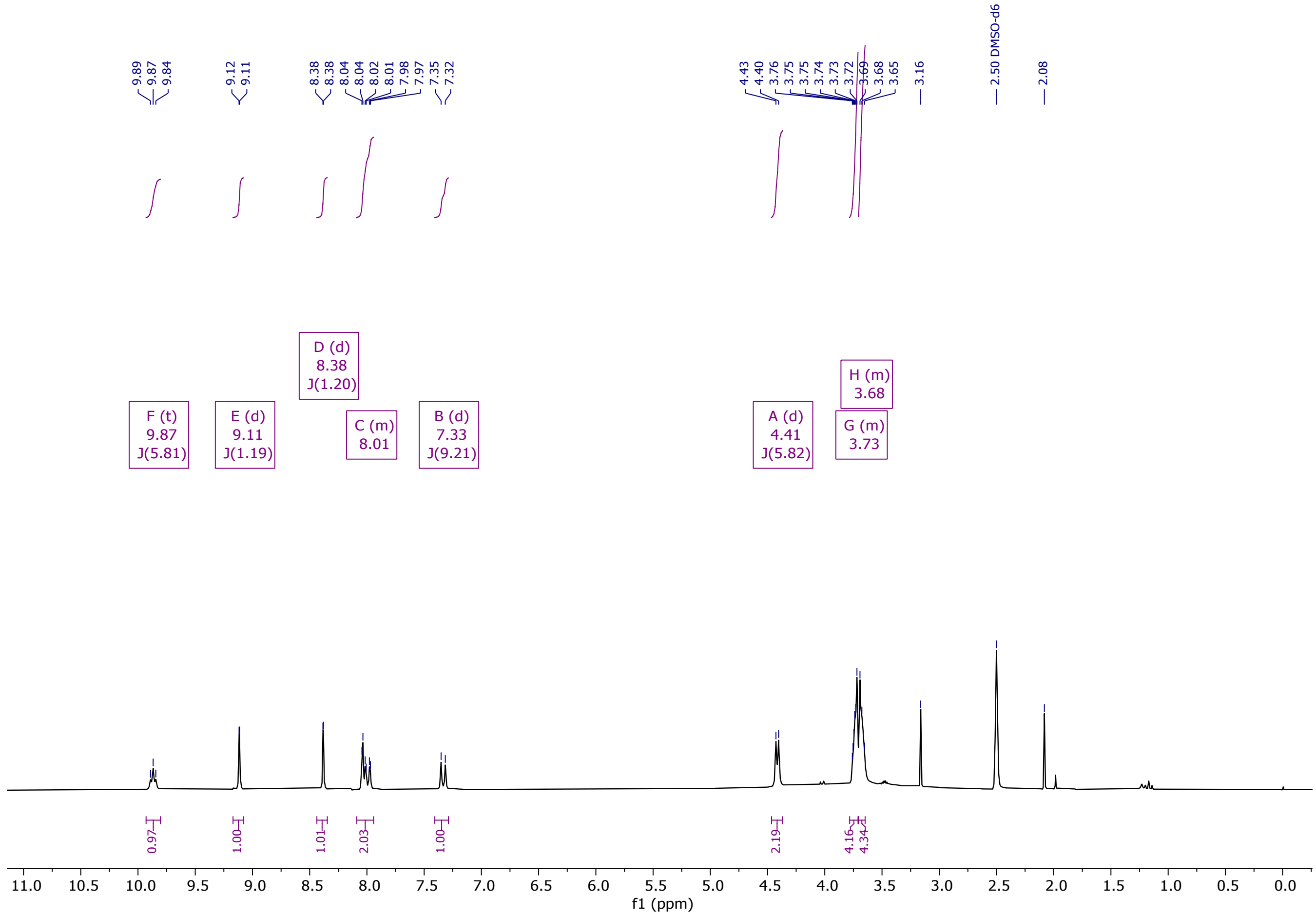

Figure S132: N-((6-morpholinopyridin-3-yl)methyl)-1H-imidazole-4-carboxamide (S25)

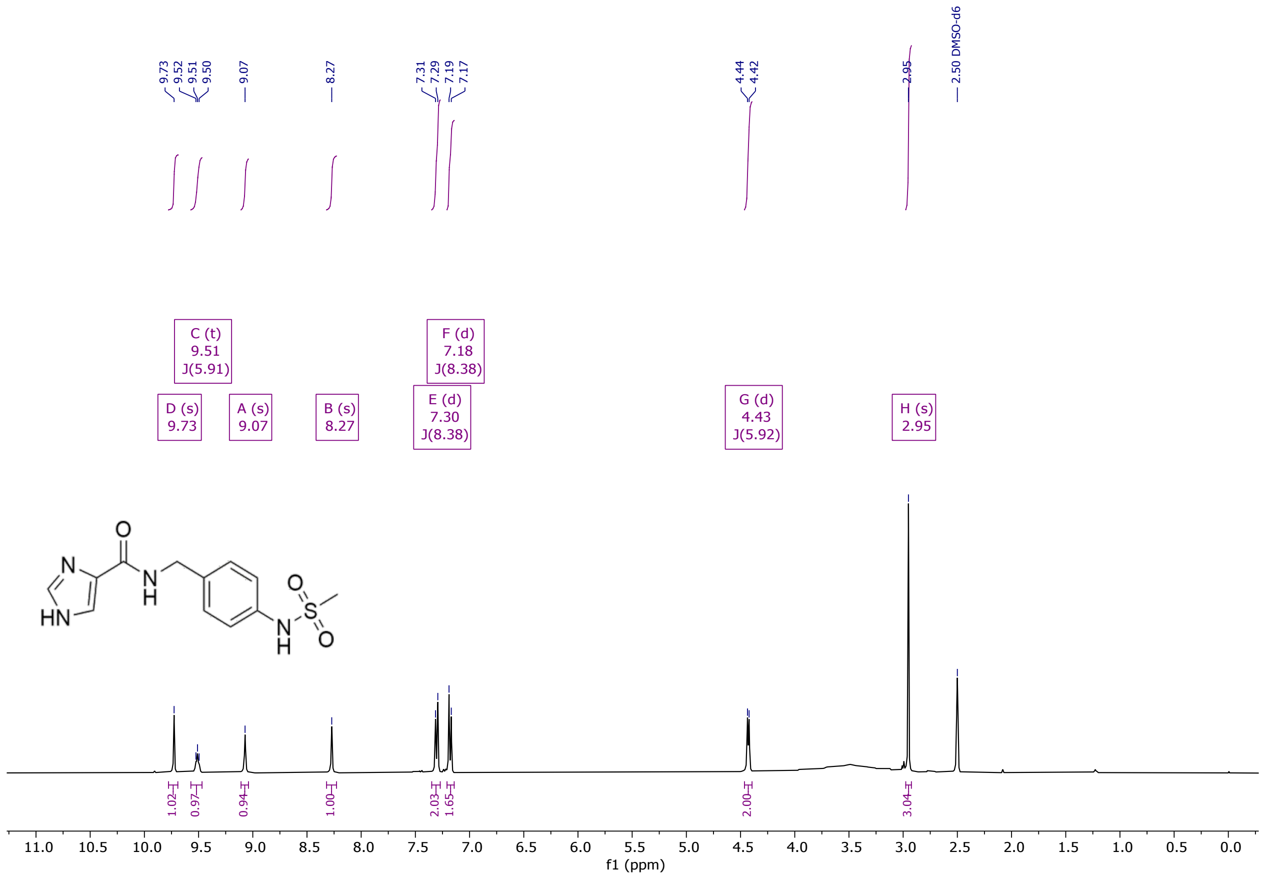

Figure S133: N-(4-(methylsulfonamido)benzyl)-1H-imidazole-4-carboxamide (S26)

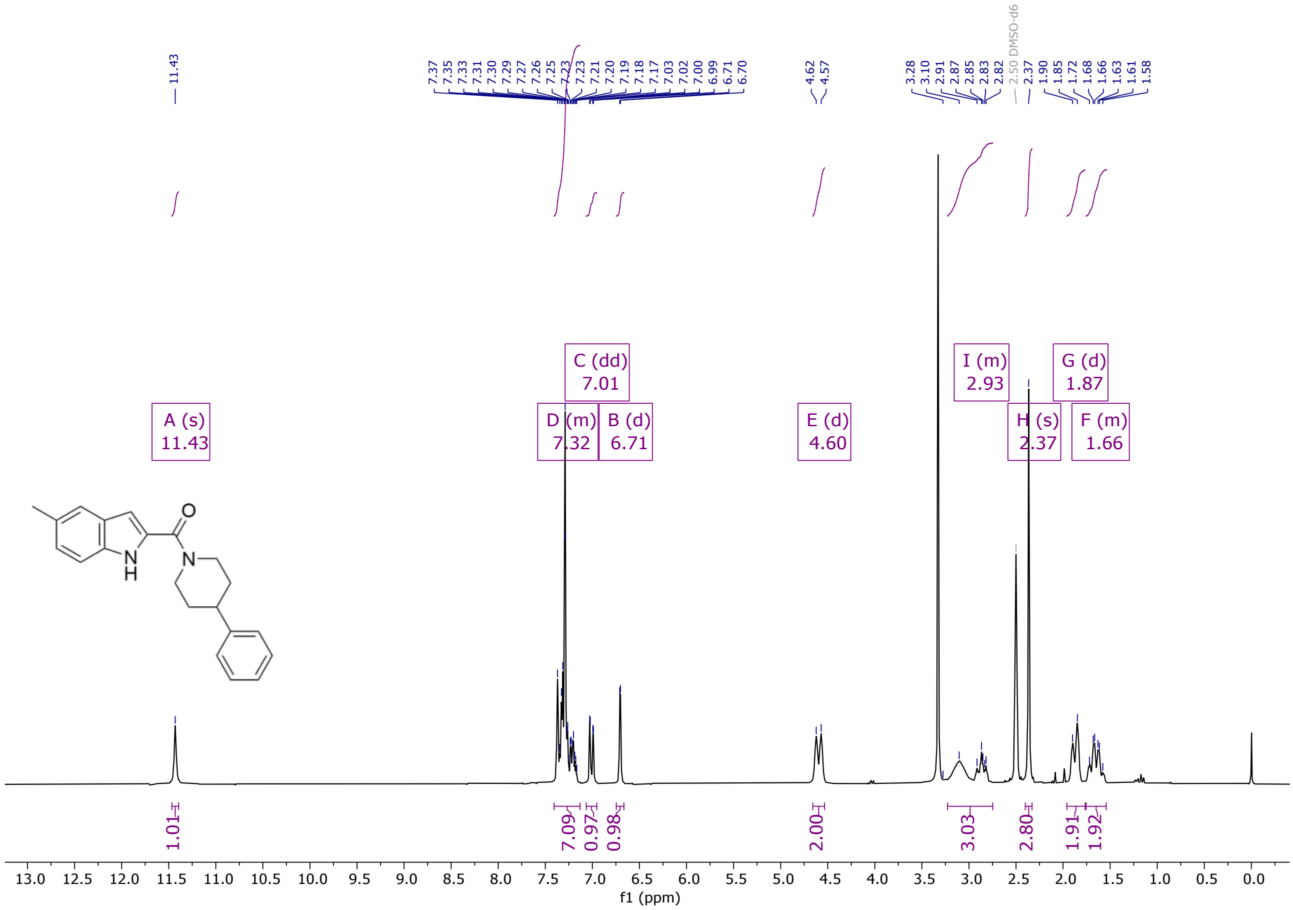

Figure S134: (5-methyl-1H-indol-2-yl)(4-phenylpiperidin-1-yl)methanone (S27)

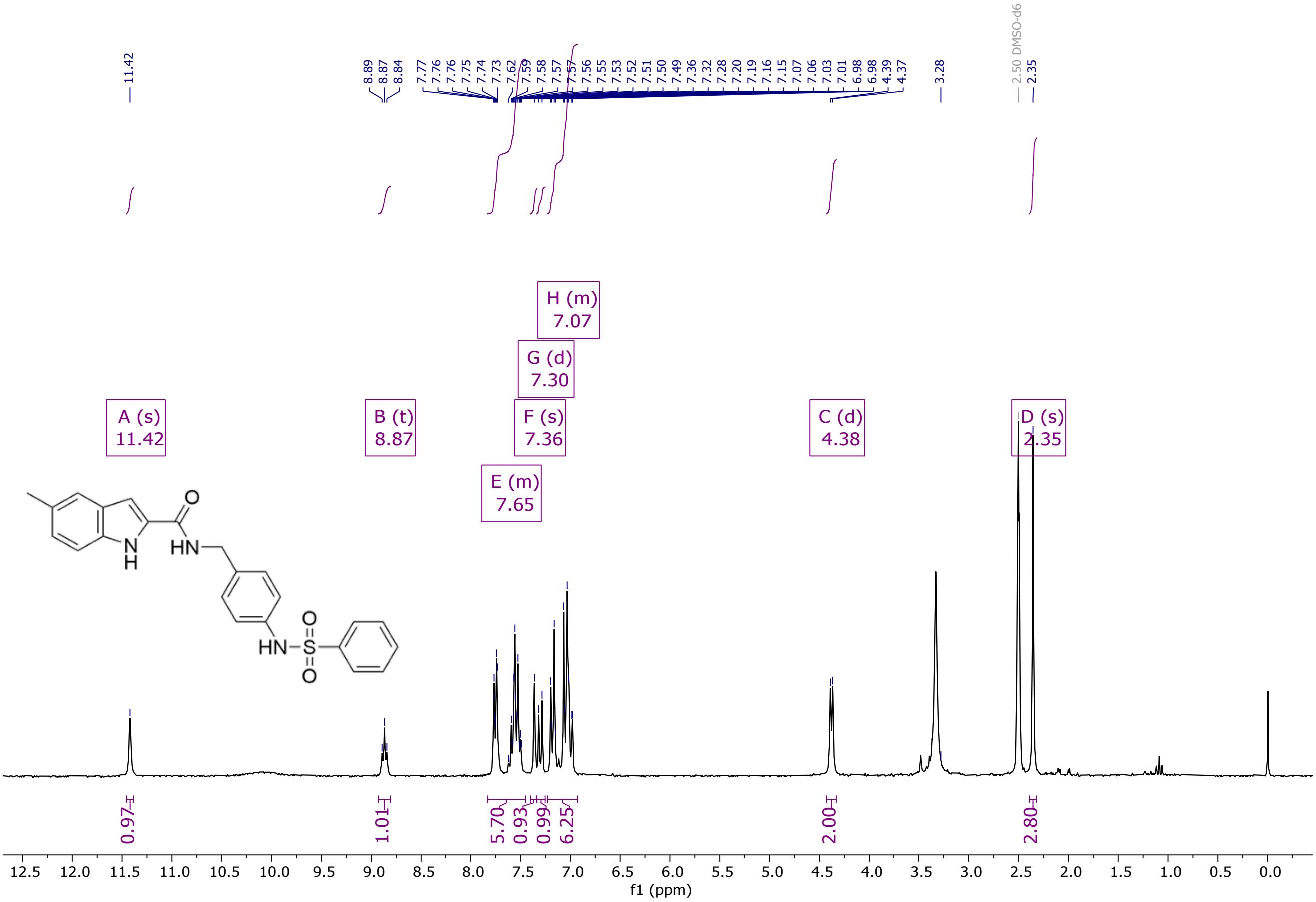

Figure S135: 5-methyl-N-(4-(phenylsulfonamido)benzyl)-1H-indole-2-carboxamide (S28)

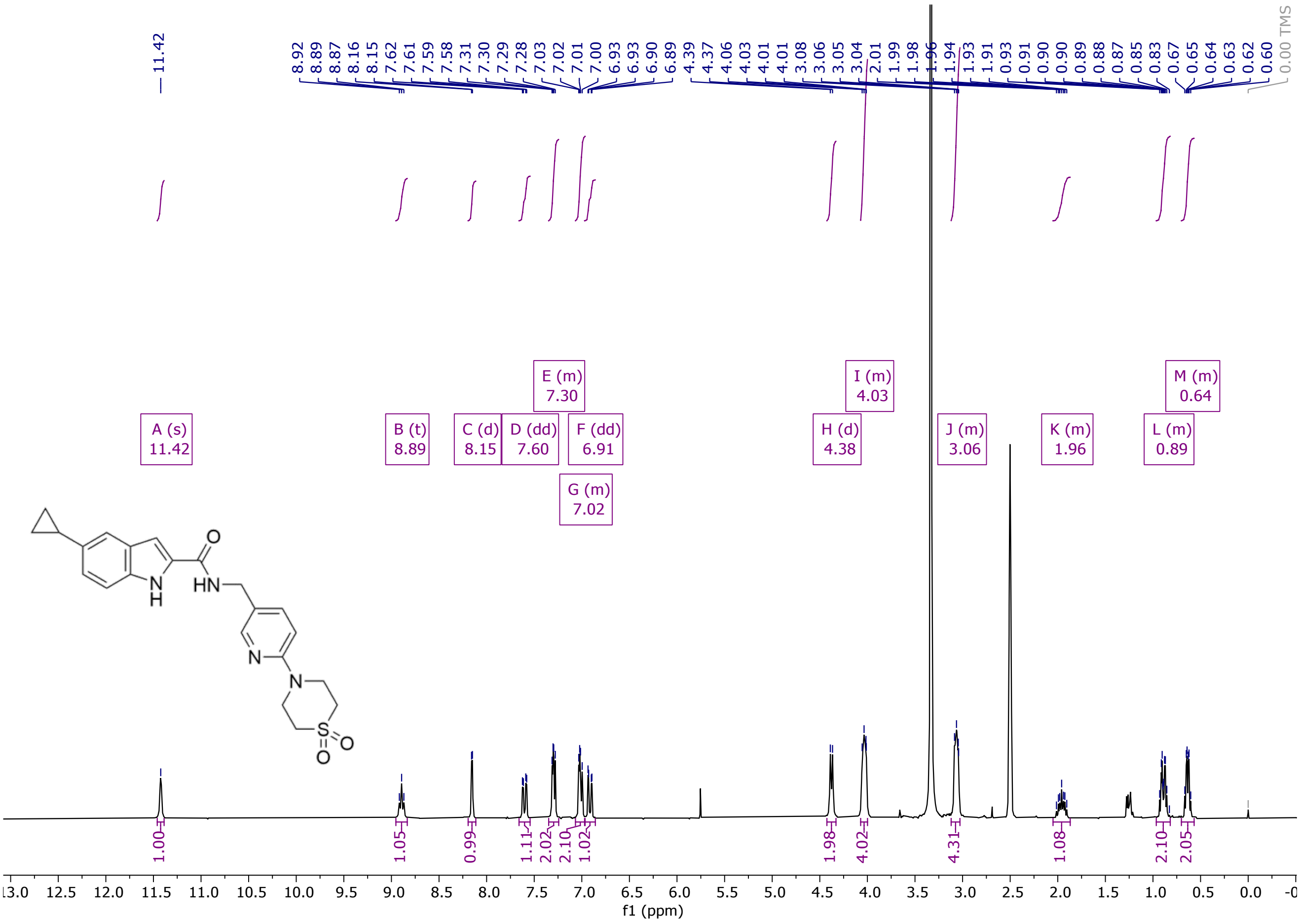

Figure S136: 5-cyclopropyl-N-((6-(1,1-dioxidothiomorpholino)pyridin-3-yl)methyl)-1H-indole-2-carboxamide (S29)

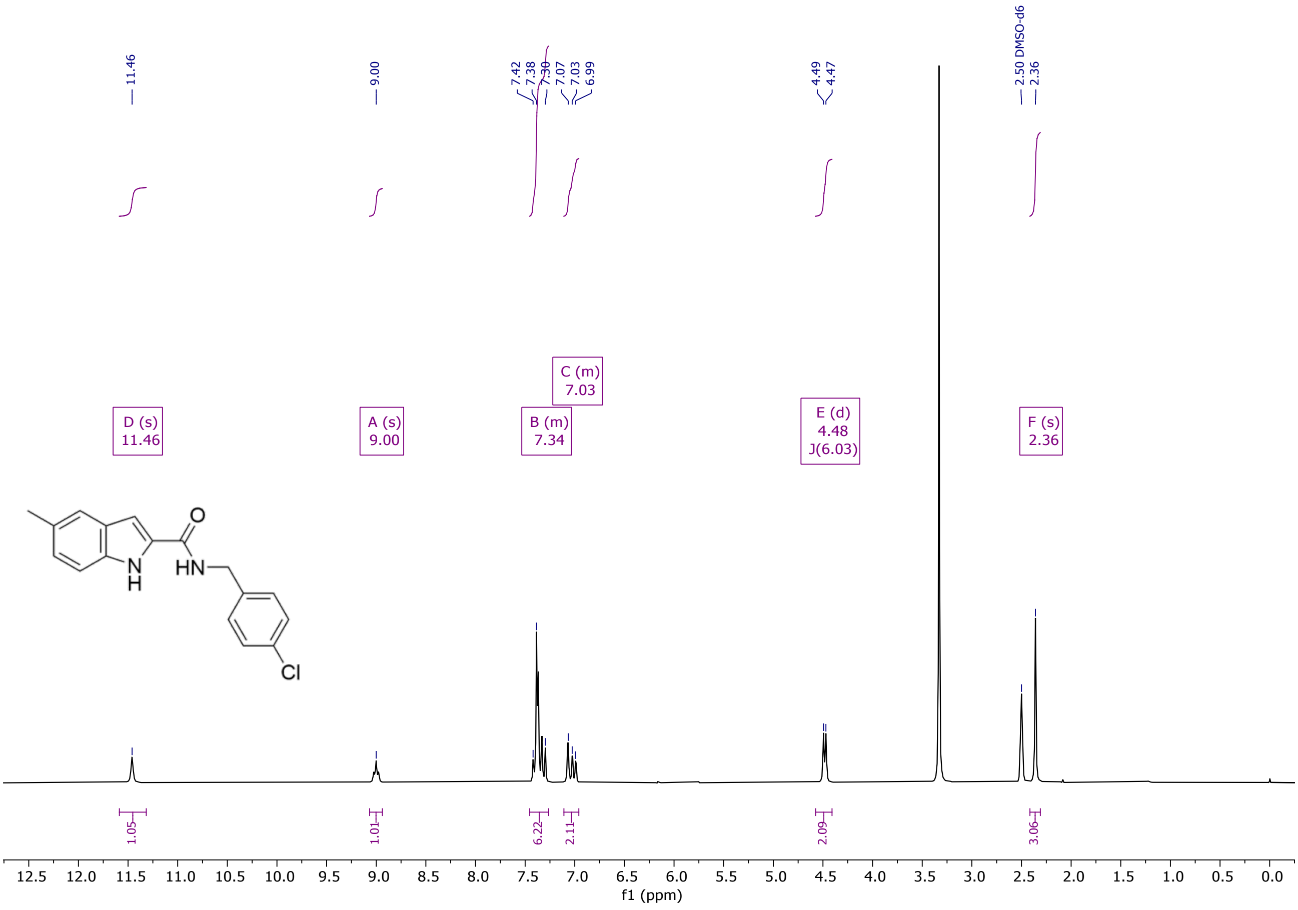

Figure S137: N-(4-chlorobenzyl)-5-methyl-1H-indole-2-carboxamide (S30)

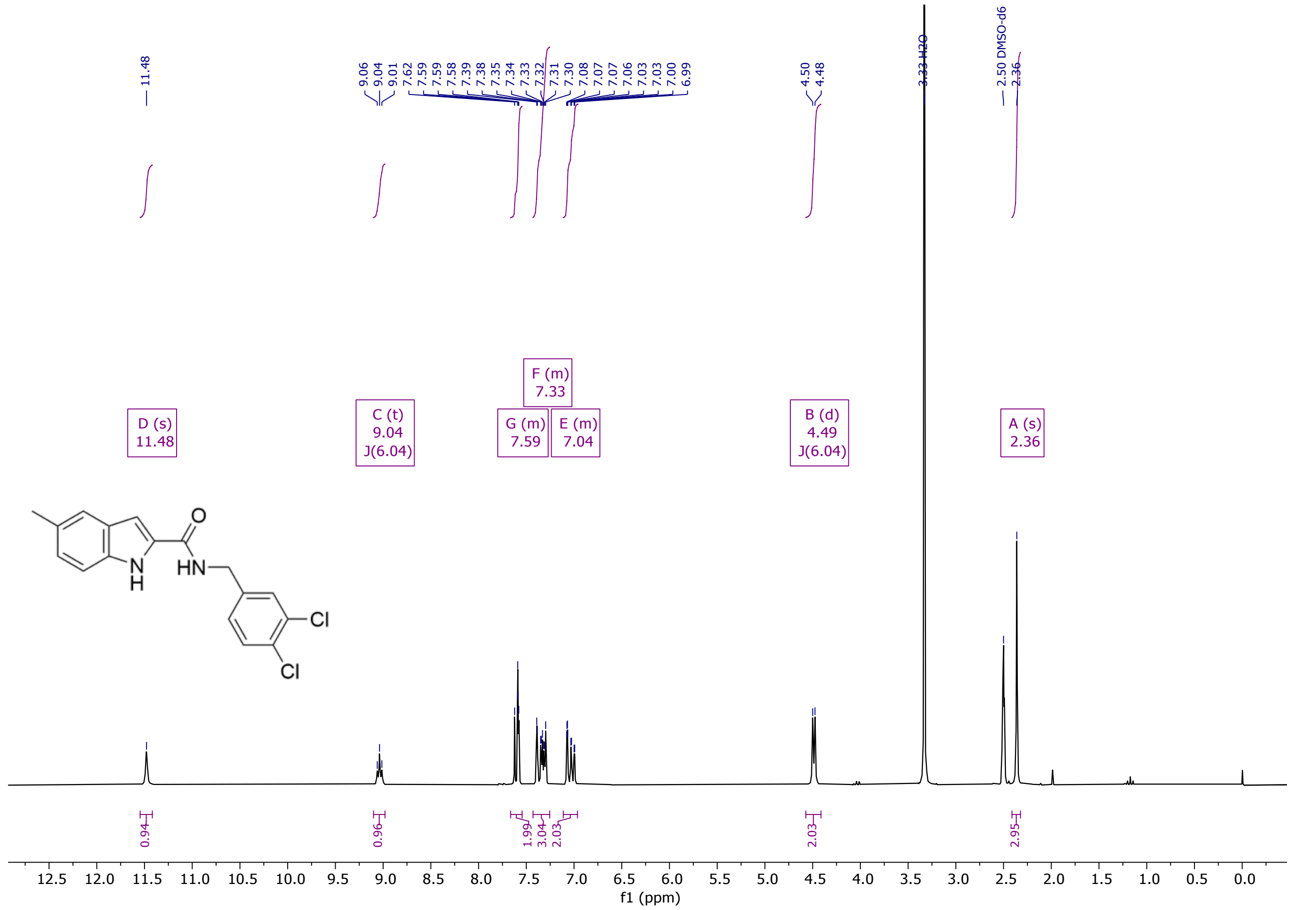

Figure S138: N-(3,4-dichlorobenzyl)-5-methyl-1H-indole-2-carboxamide (S31)

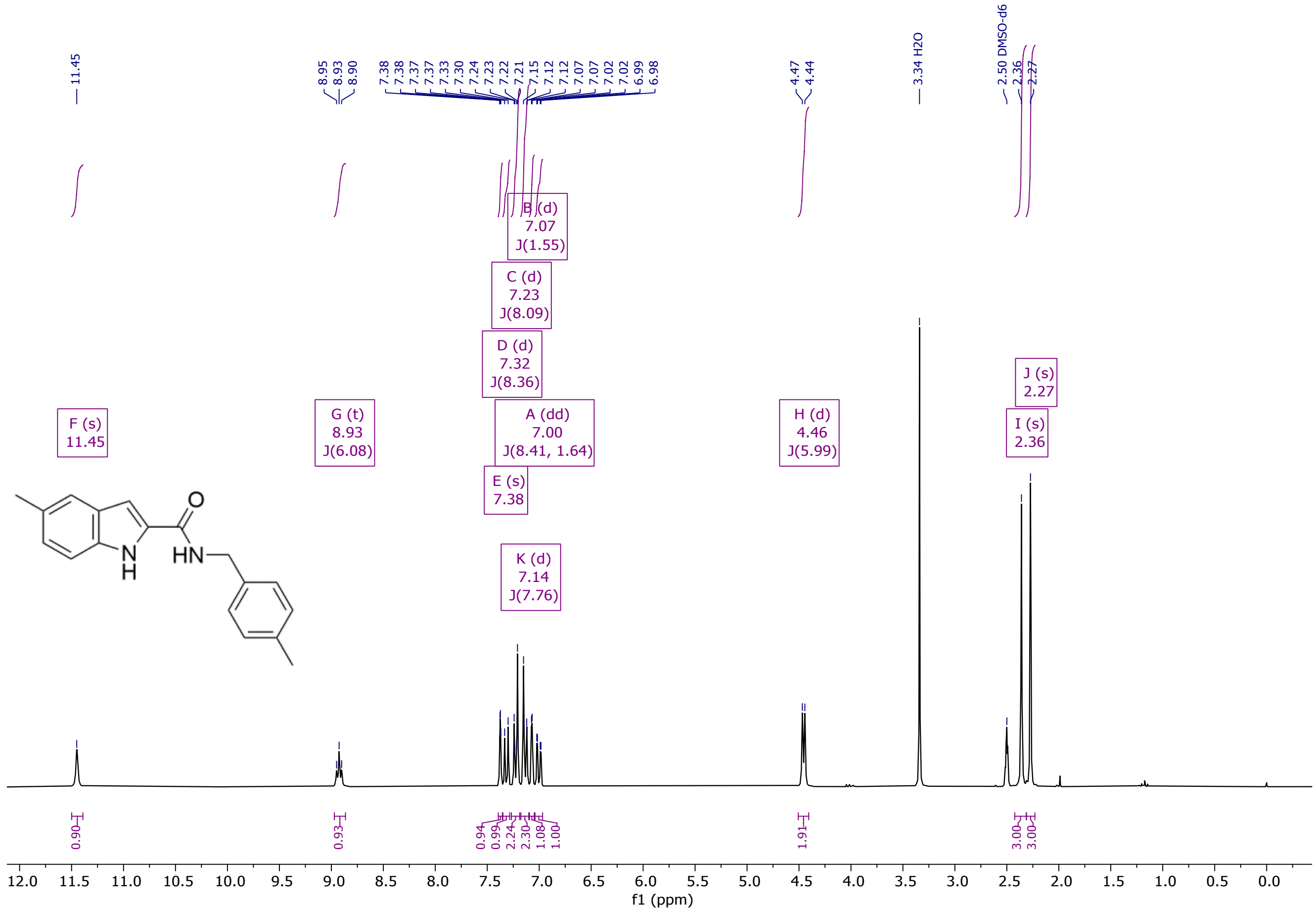

Figure S139: 5-methyl-N-(4-methylbenzyl)-1H-indole-2-carboxamide (S32)

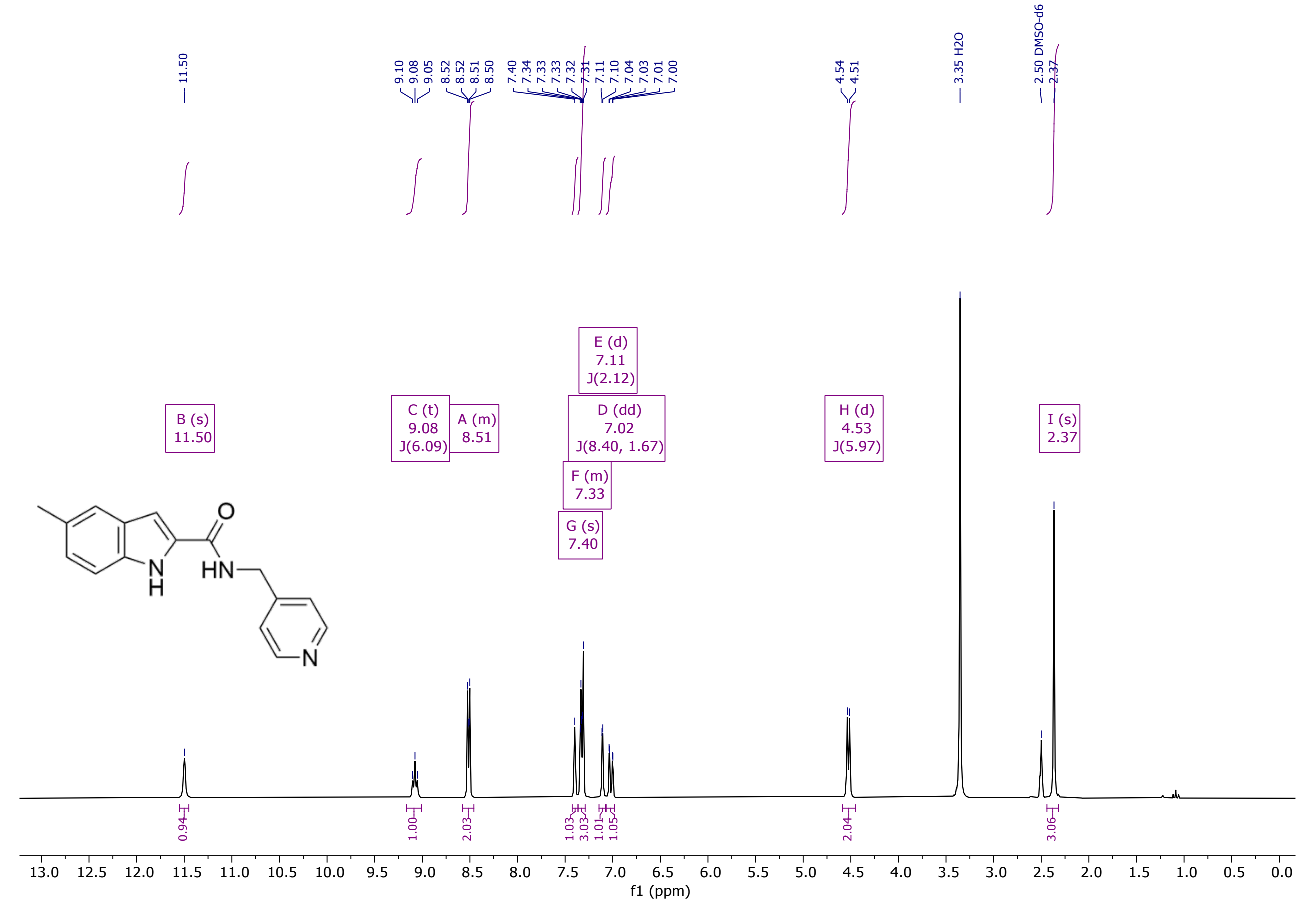

Figure S140: 5-methyl-N-(pyridin-4-ylmethyl)-1H-indole-2-carboxamide (S33)

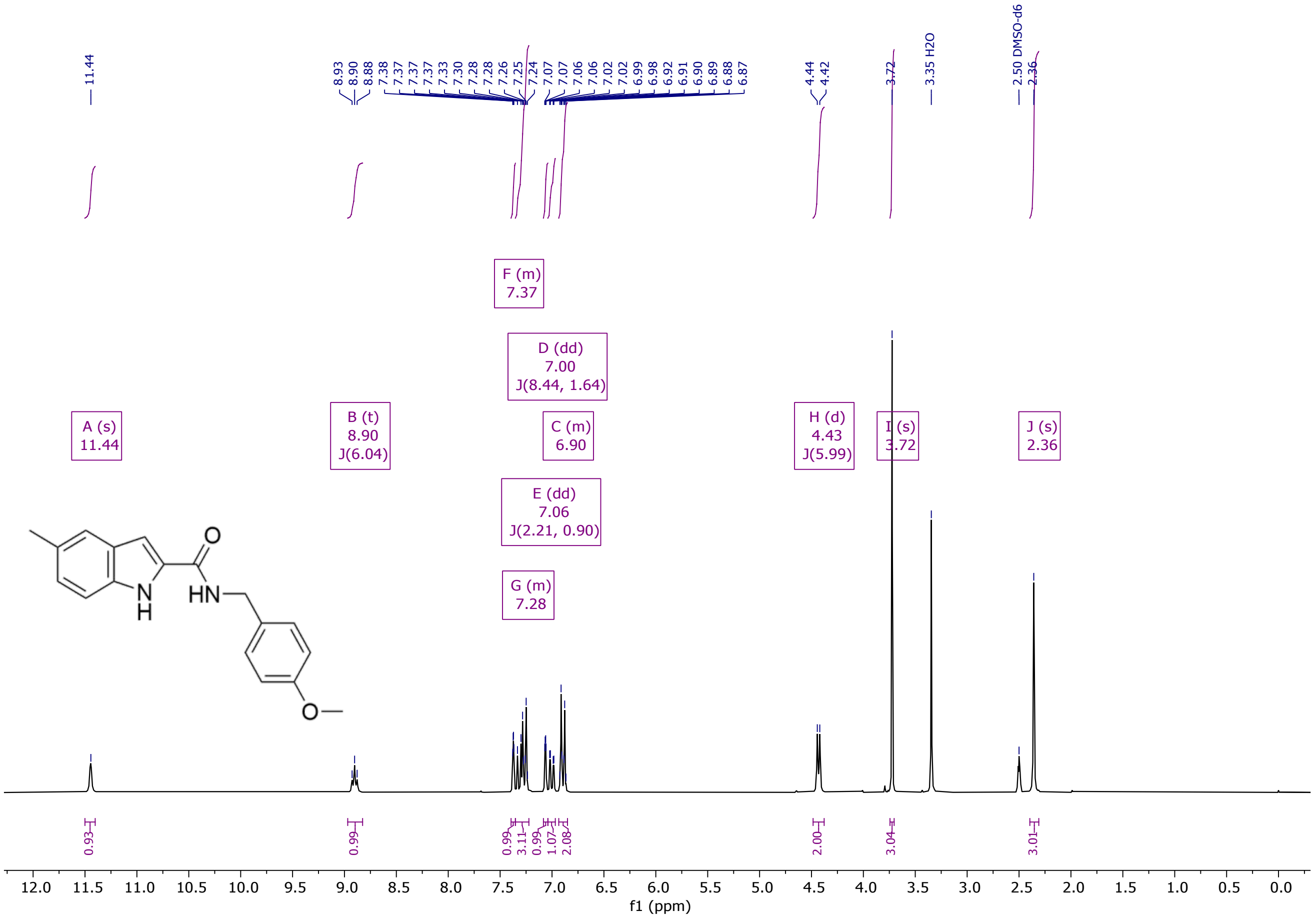

Figure S141: N-(4-methoxybenzyl)-5-methyl-1H-indole-2-carboxamide (S34)

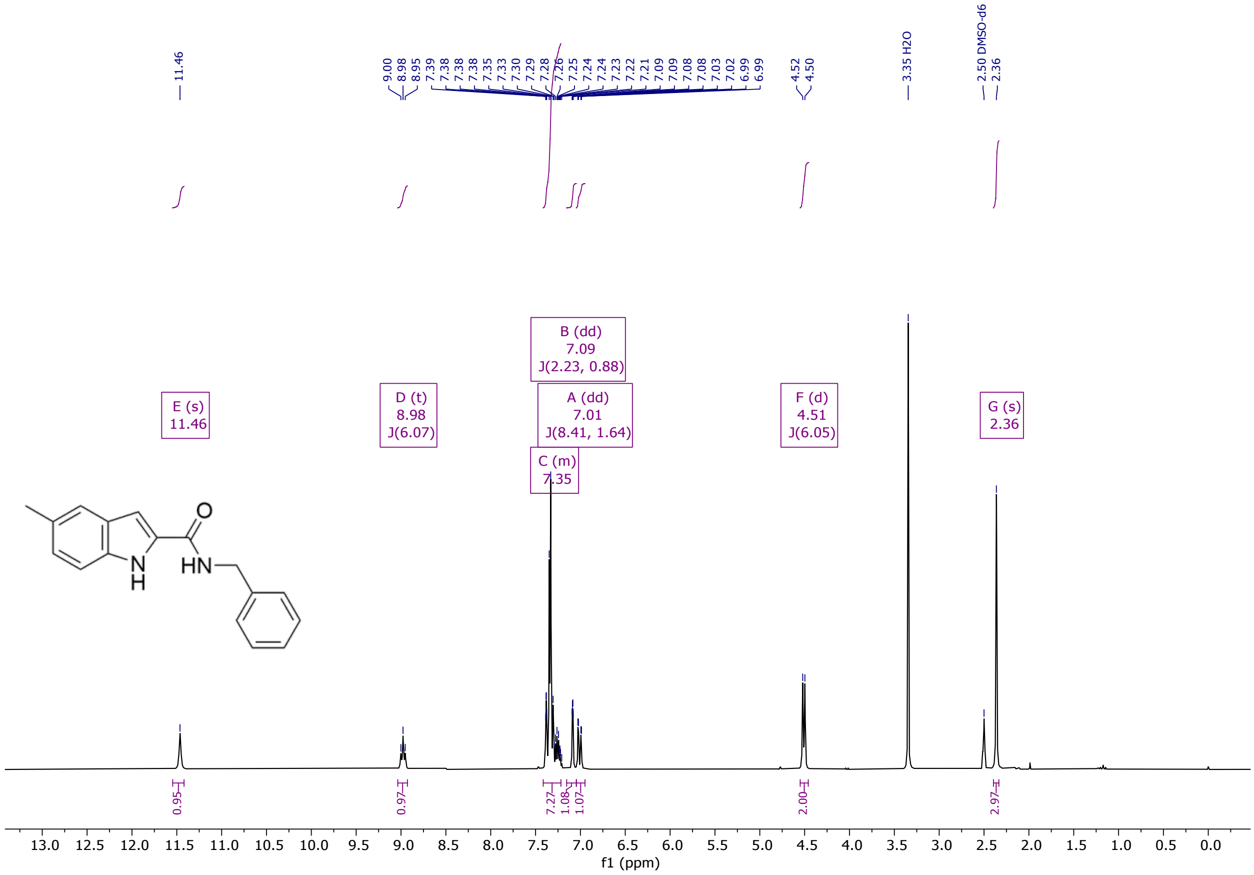

Figure S142: N-benzyl-5-methyl-1H-indole-2-carboxamide (S35)

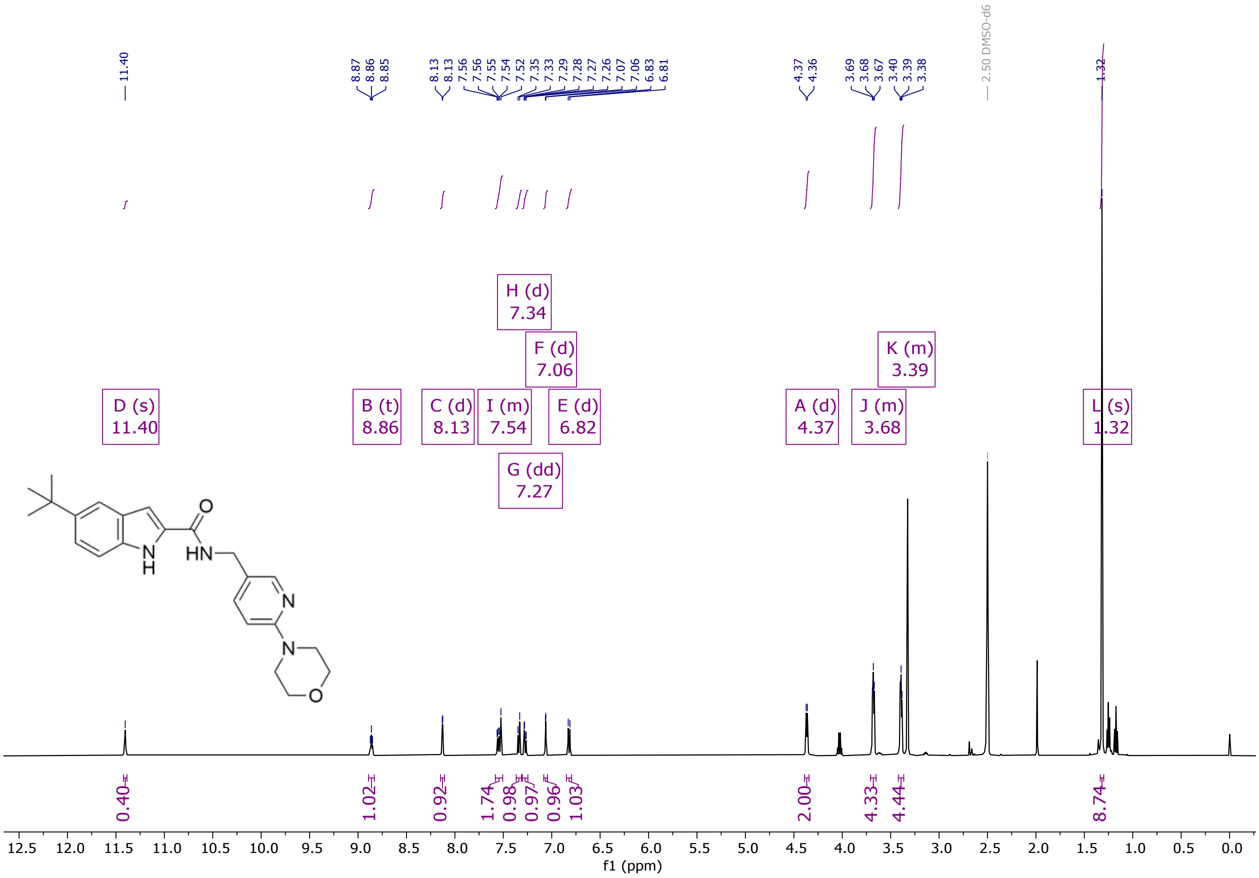

Figure S143: 5-(tert-butyl)-N-((6-morpholinopyridin-3-yl)methyl)-1H-indole-2-carboxamide (S36)

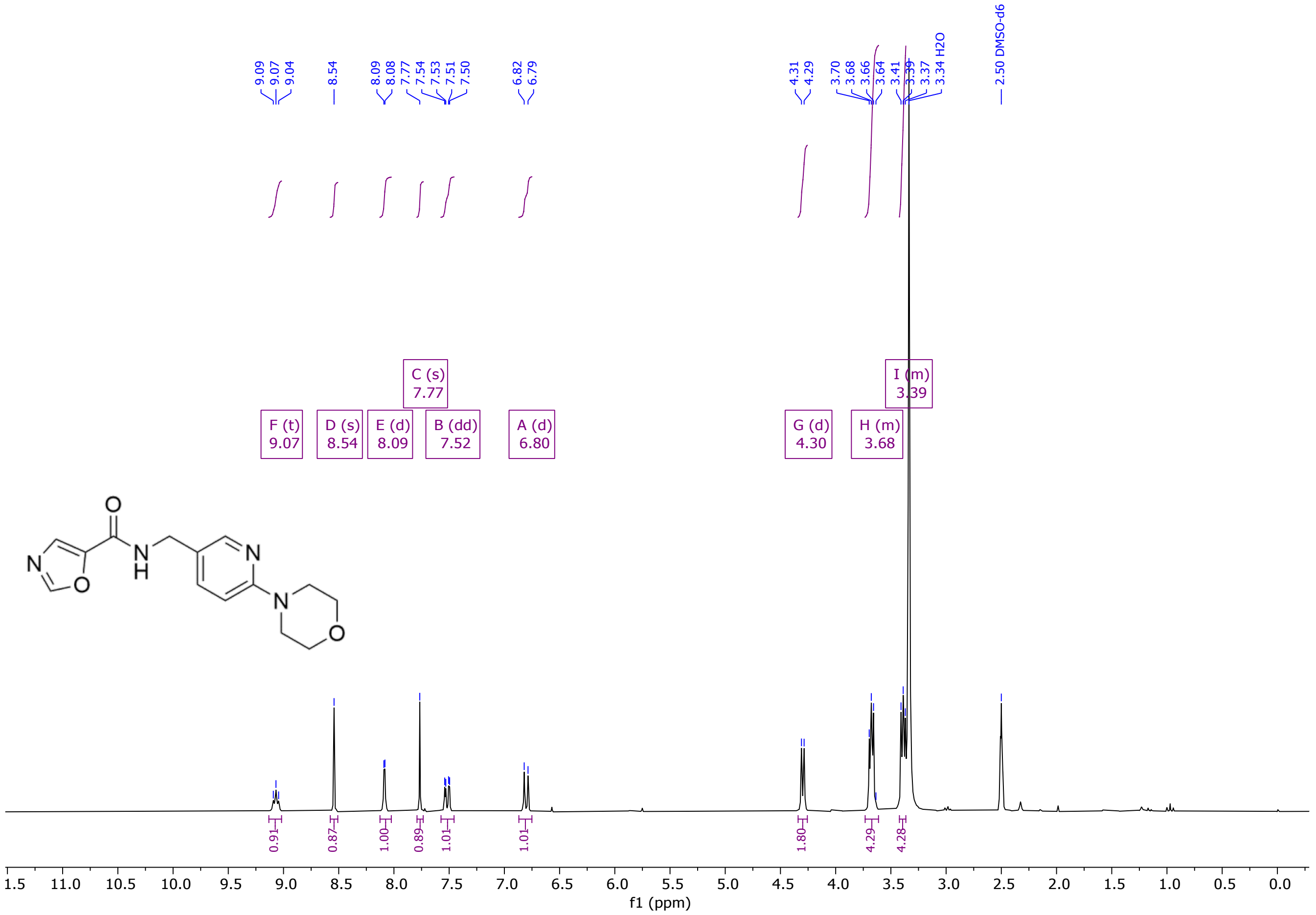

Figure S144: N-((6-morpholinopyridin-3-yl)methyl)oxazole-5-carboxamide (S37)

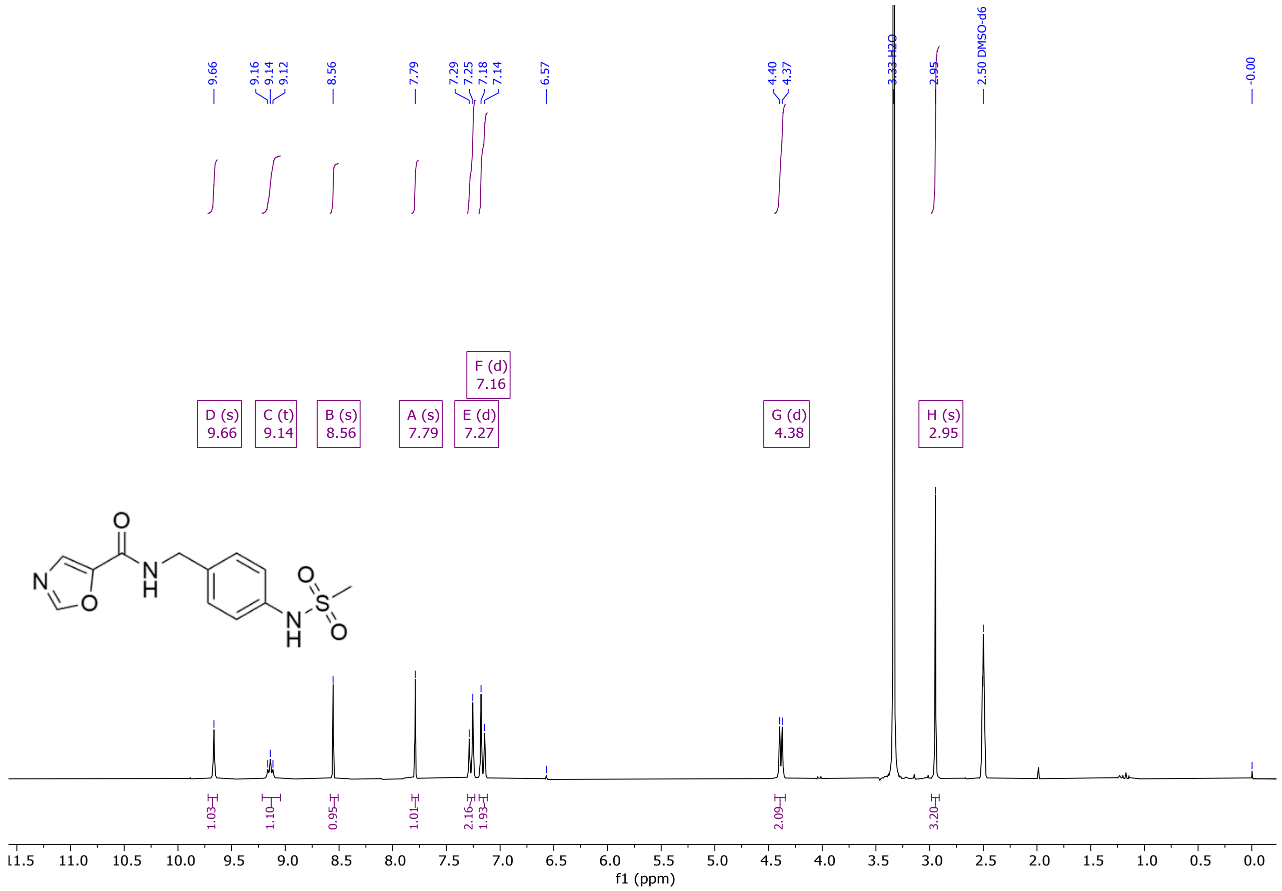

Figure S145: N-(4-(methylsulfonamido)benzyl)oxazole-5-carboxamide (S38)

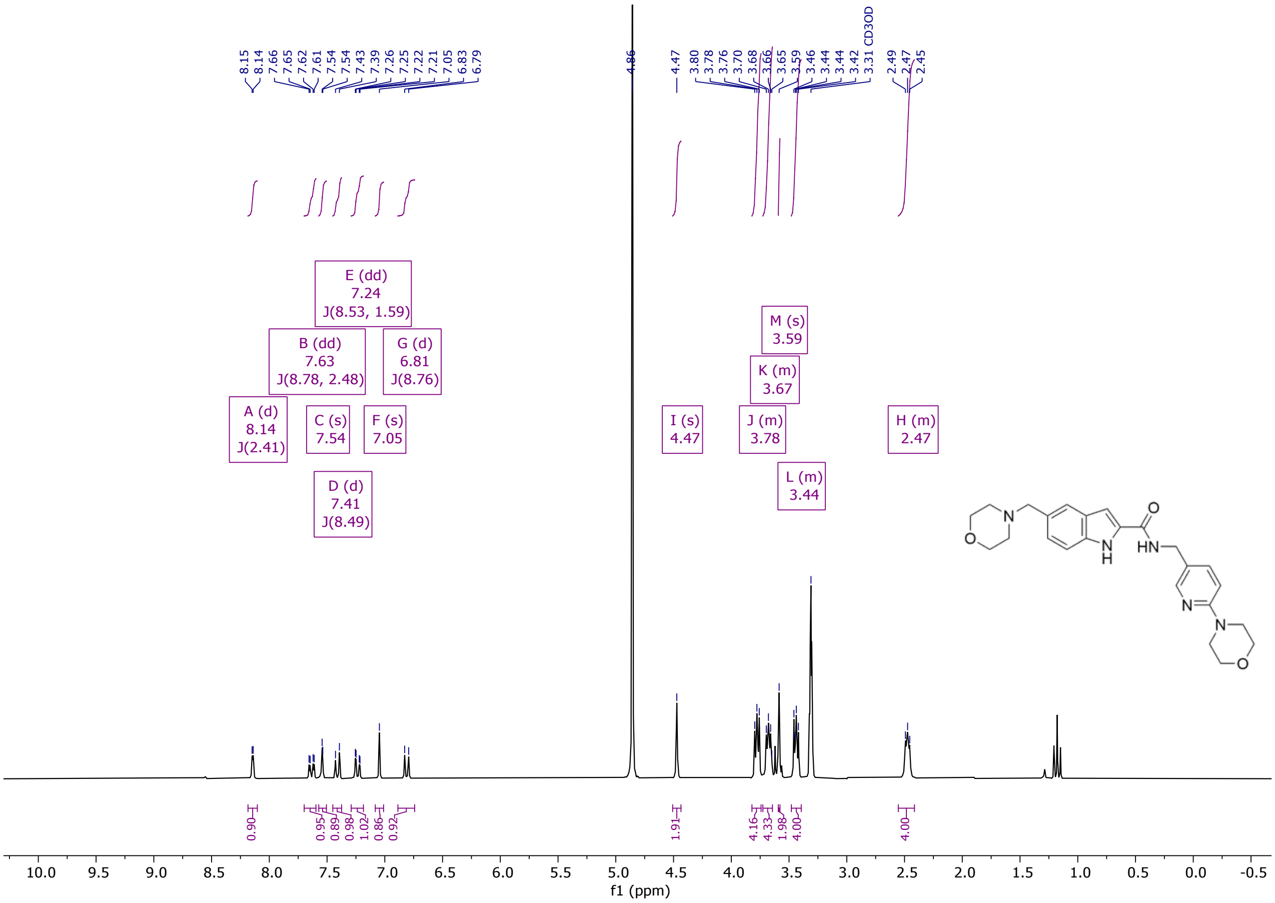

Figure S146: 5-(morpholinomethyl)-N-((6-morpholinopyridin-3-yl)methyl)-1H-indole-2-carboxamide (S39)

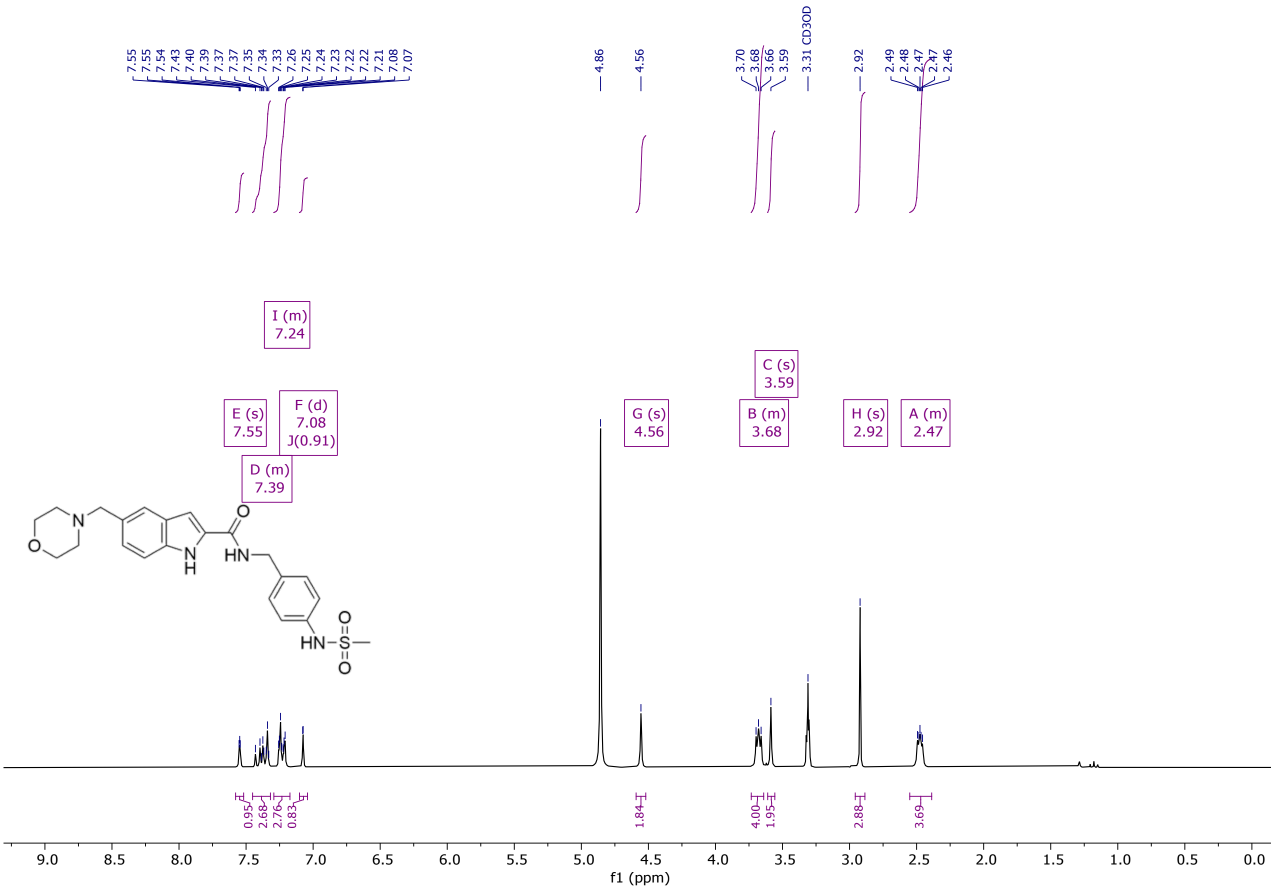

Figure S147: N-(4-(methylsulfonamido)benzyl)-5-(morpholinomethyl)-1H-indole-2-carboxamide (S40)

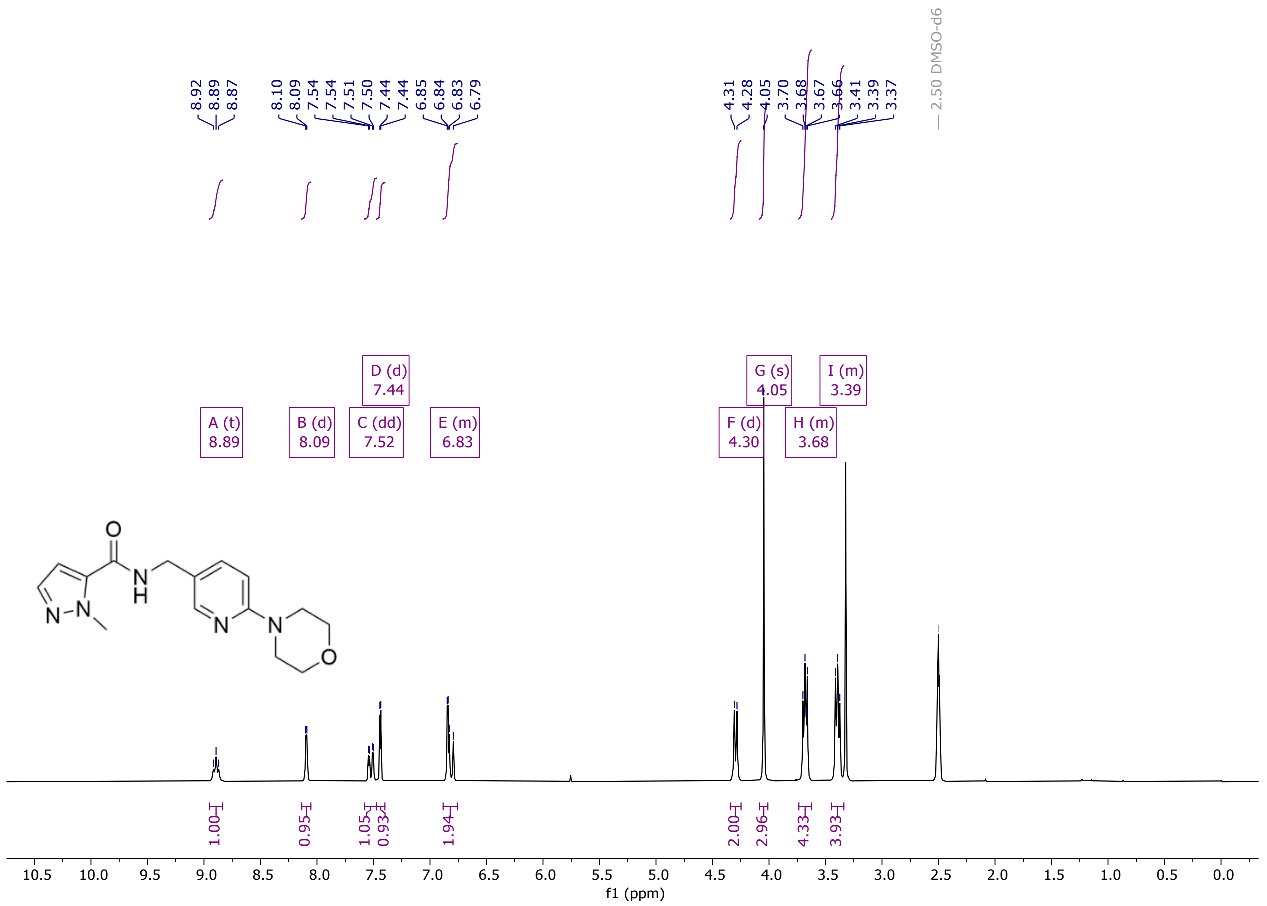

Figure S148: 1-methyl-N-((6-morpholinopyridin-3-yl)methyl)-1H-pyrazole-5-carboxamide (S41)

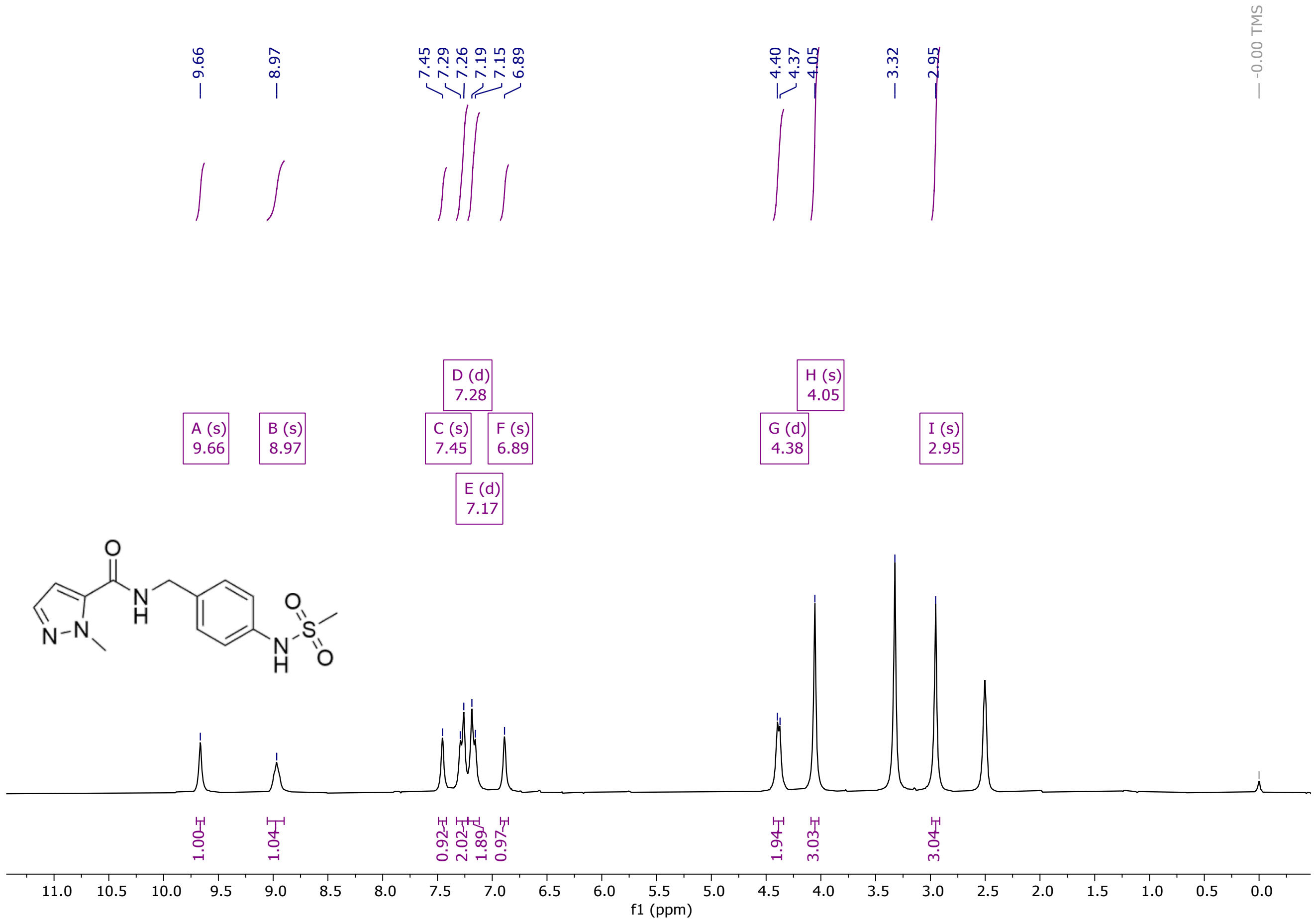

Figure S149: 1-methyl-N-(4-(methylsulfonamido)benzyl)-1H-pyrazole-5-carboxamide (S42)

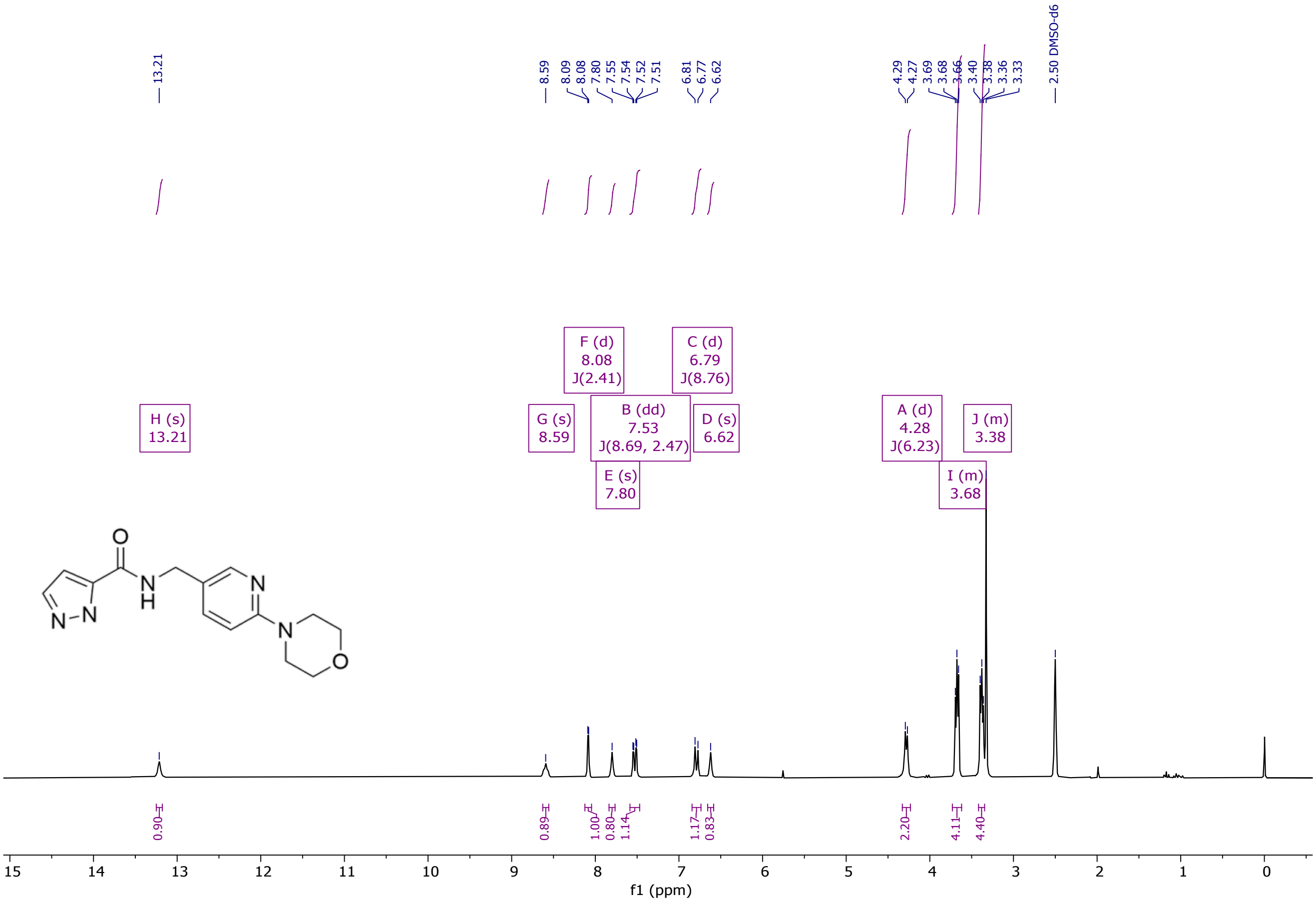

Figure S150: N-((6-morpholinopyridin-3-yl)methyl)-1H-pyrazole-3-carboxamide (S43)

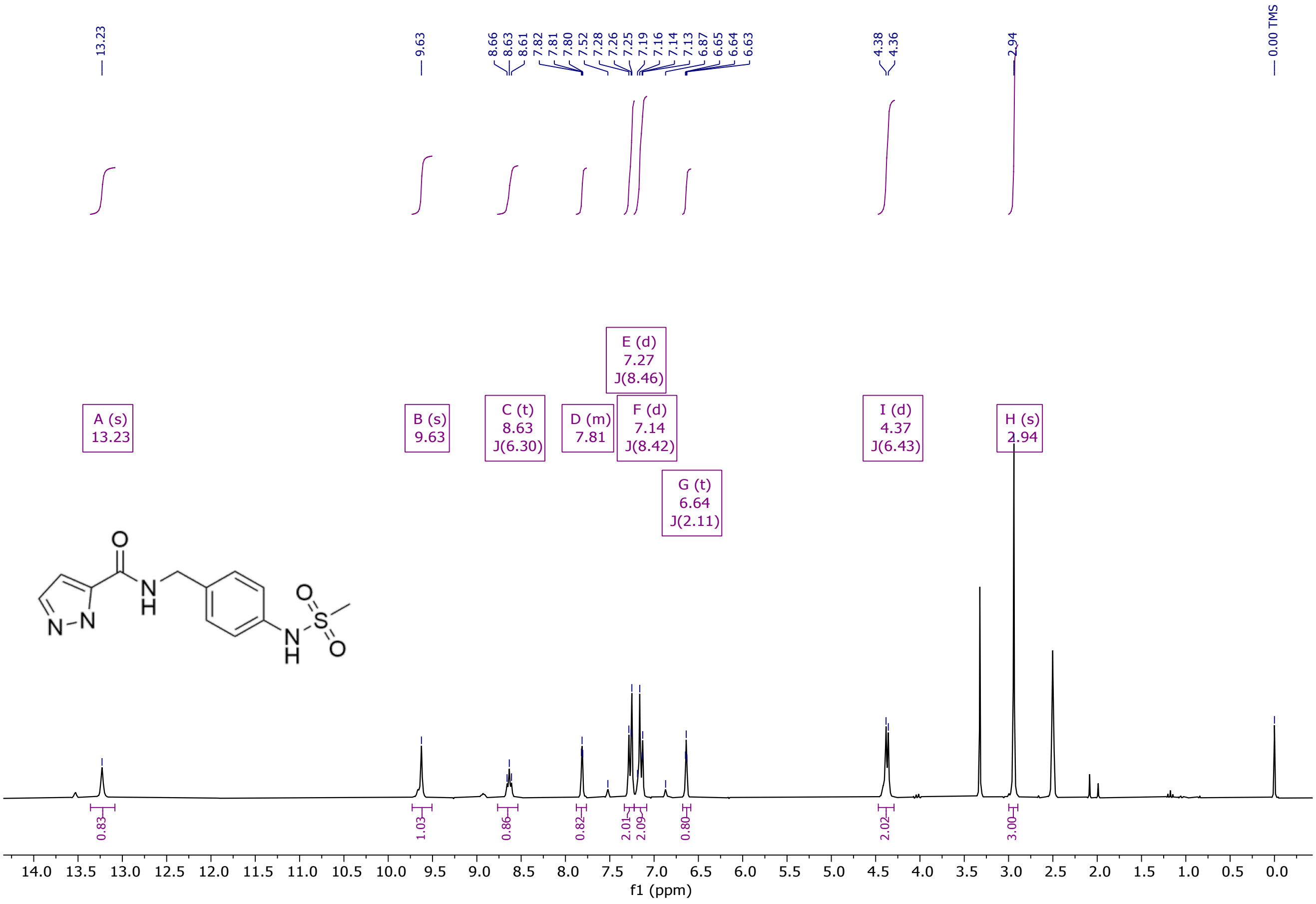

Figure S151: N-(4-(methylsulfonamido)benzyl)-1H-pyrazole-3-carboxamide (S44)

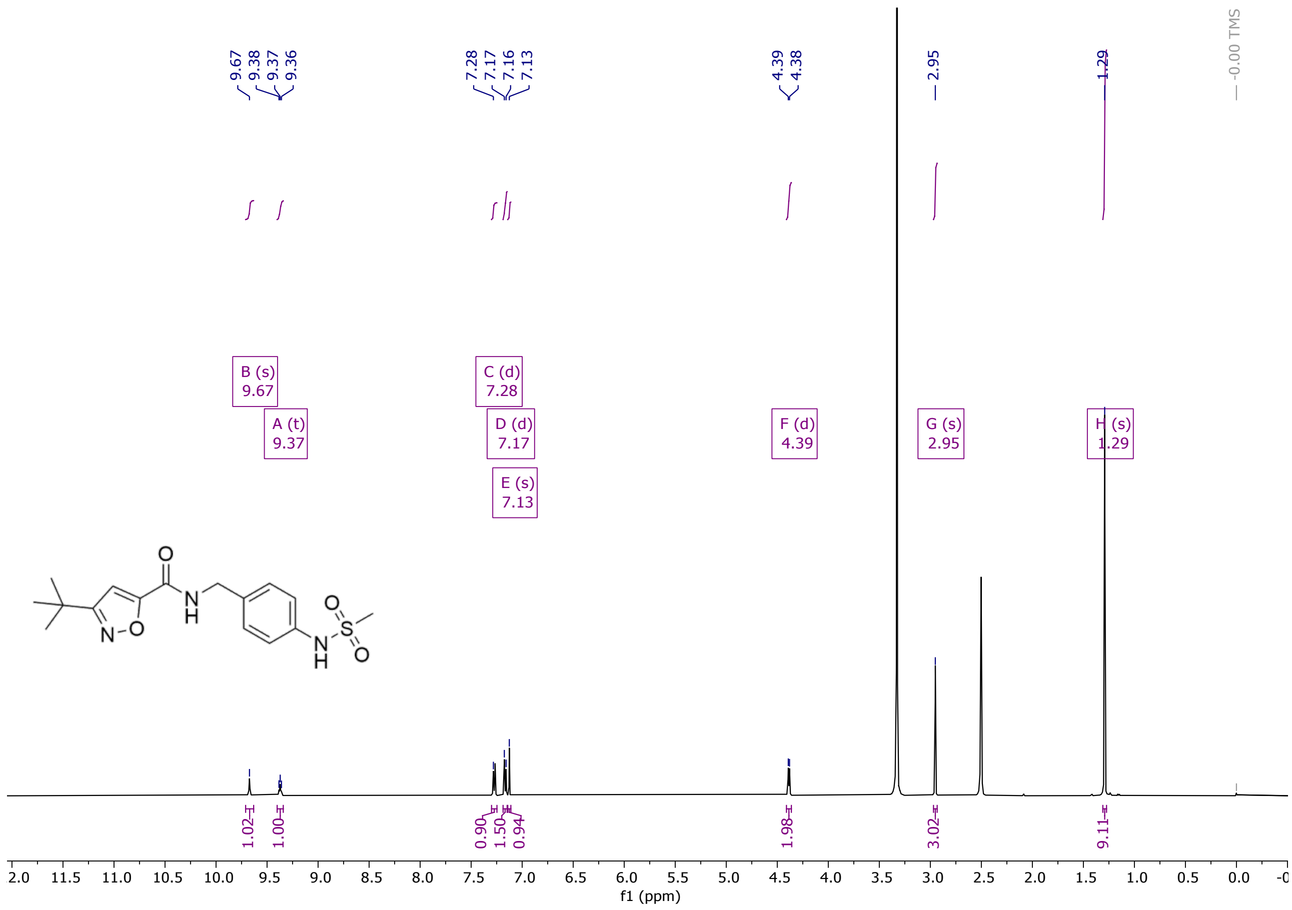

Figure S152: 3-(tert-butyl)-N-(4-(methylsulfonamido)benzyl)isoxazole-5-carboxamide (S45)

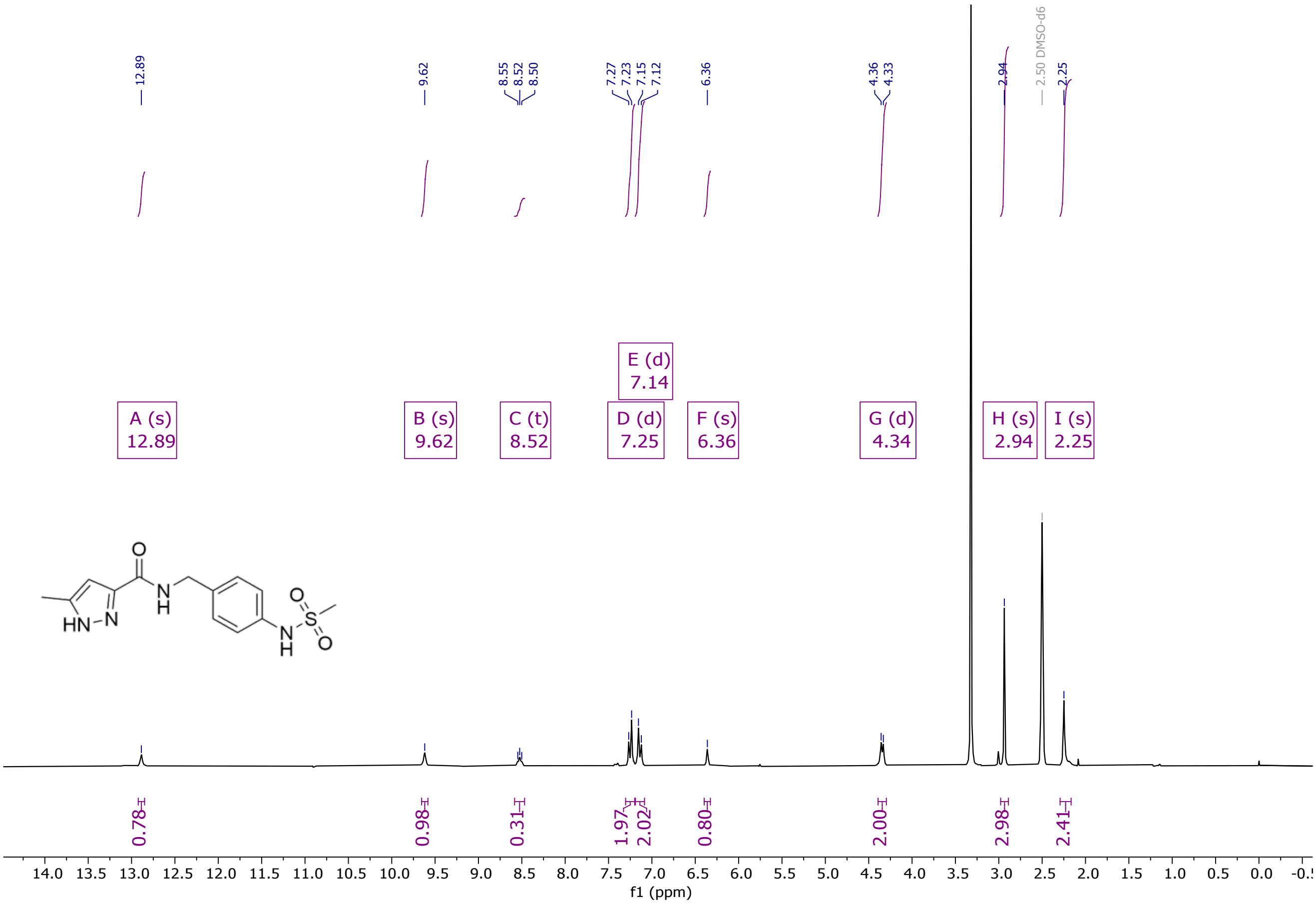

Figure S153: 5-methyl-N-(4-(methylsulfonamido)benzyl)-1H-pyrazole-3-carboxamide (S46)

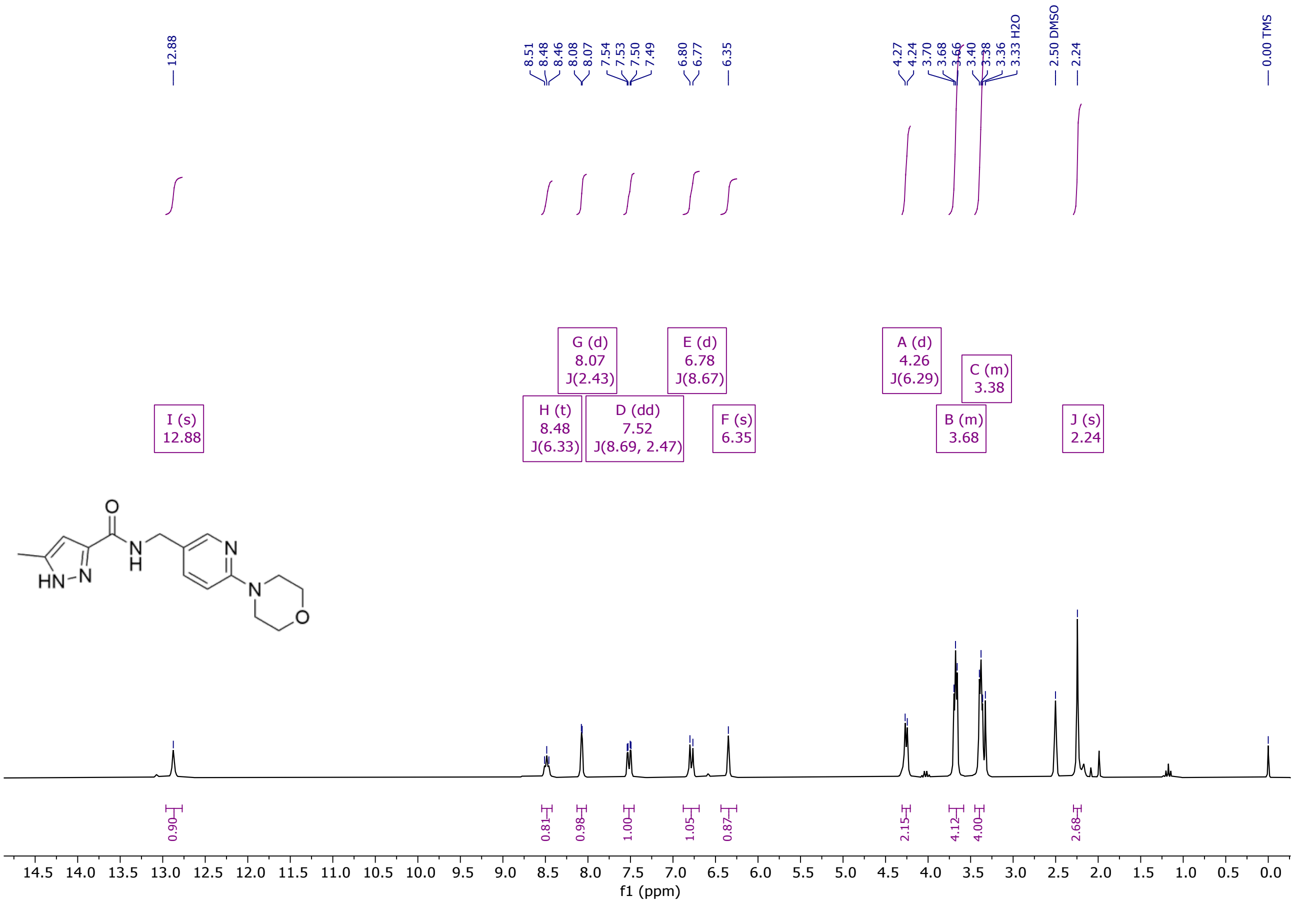

Figure S154: 5-methyl-N-((6-morpholinopyridin-3-yl)methyl)-1H-pyrazole-3-carboxamide (S47)

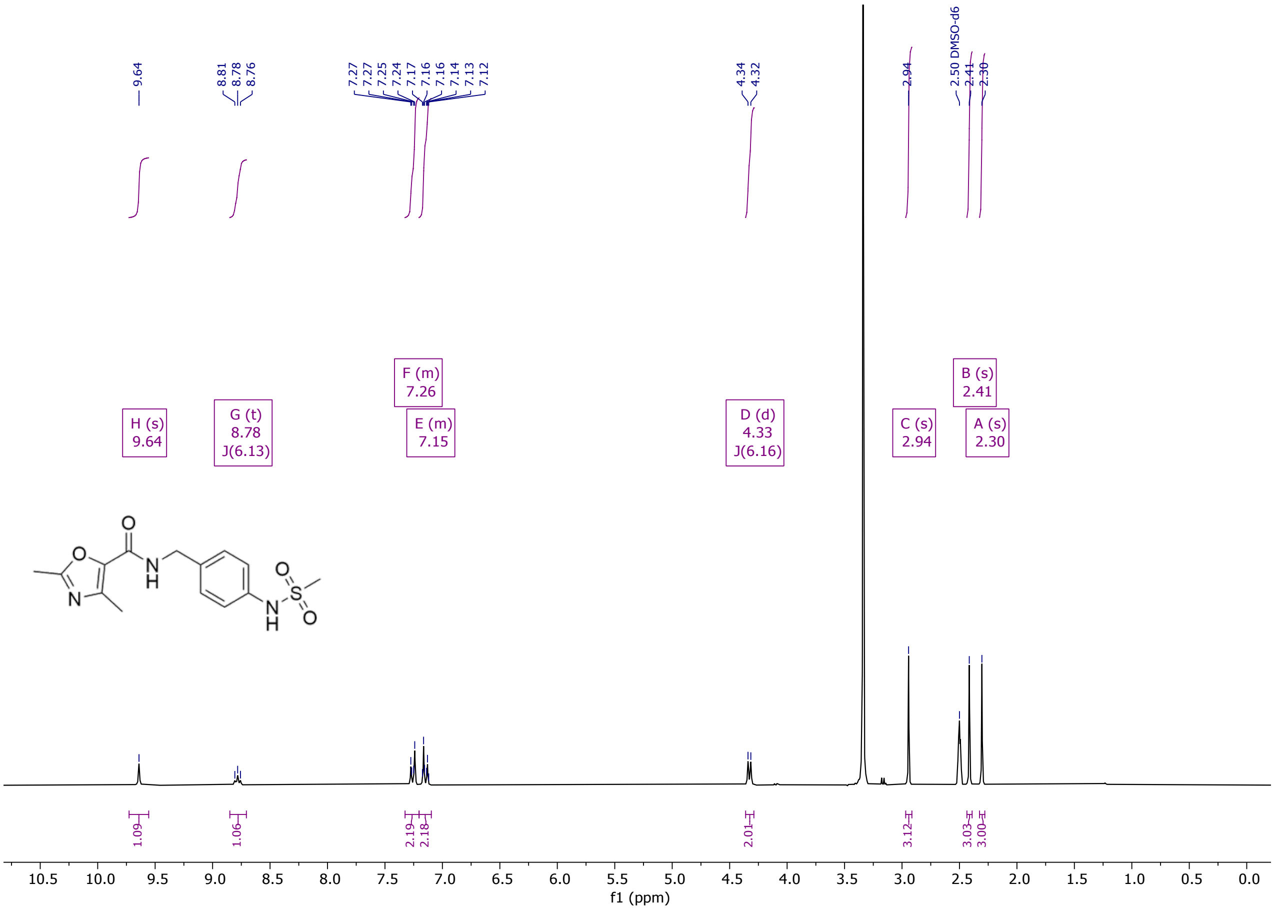

Figure S155: 2,4-dimethyl-N-(4-(methylsulfonamido)benzyl)oxazole-5-carboxamide (S48)

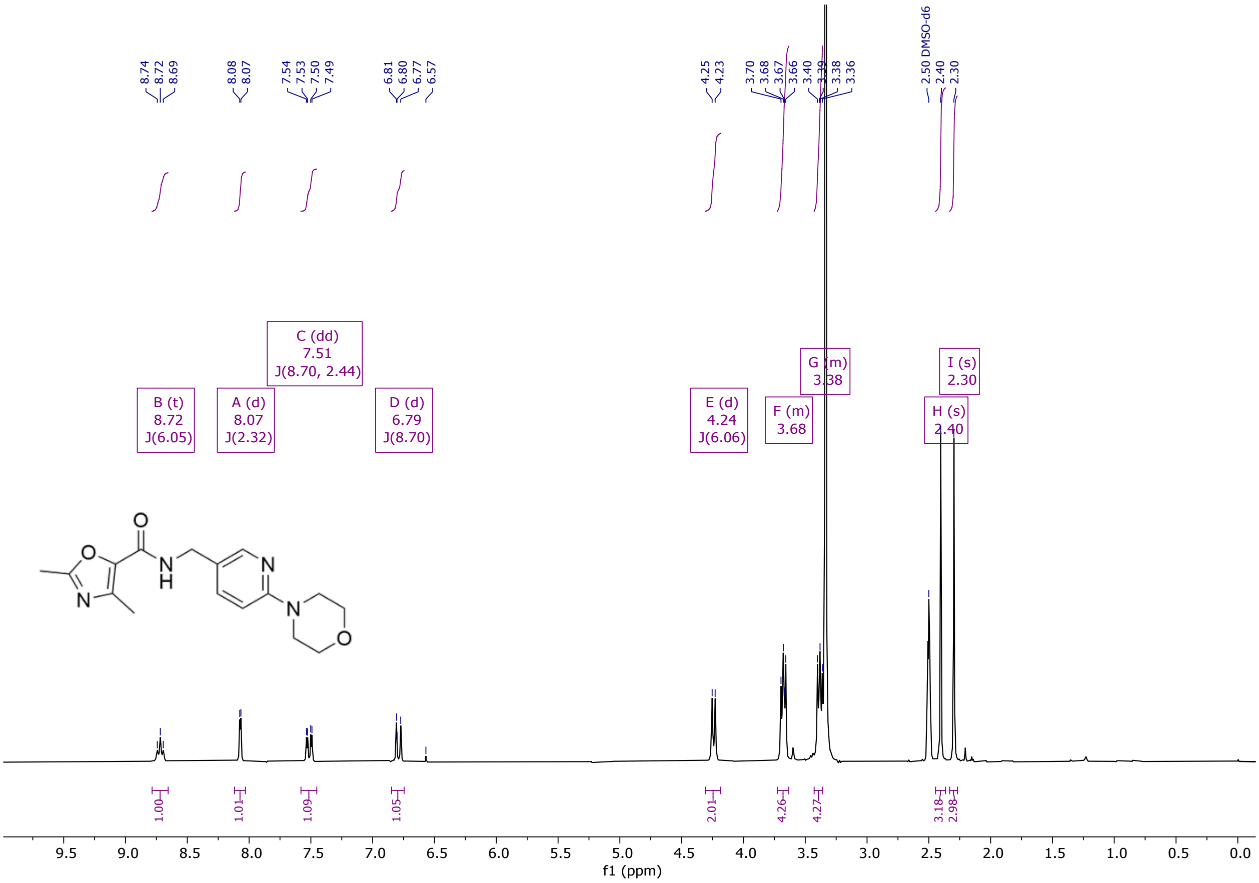

Figure S156: 2,4-dimethyl-N-((6-morpholinopyridin-3-yl)methyl)oxazole-5-carboxamide (S49)

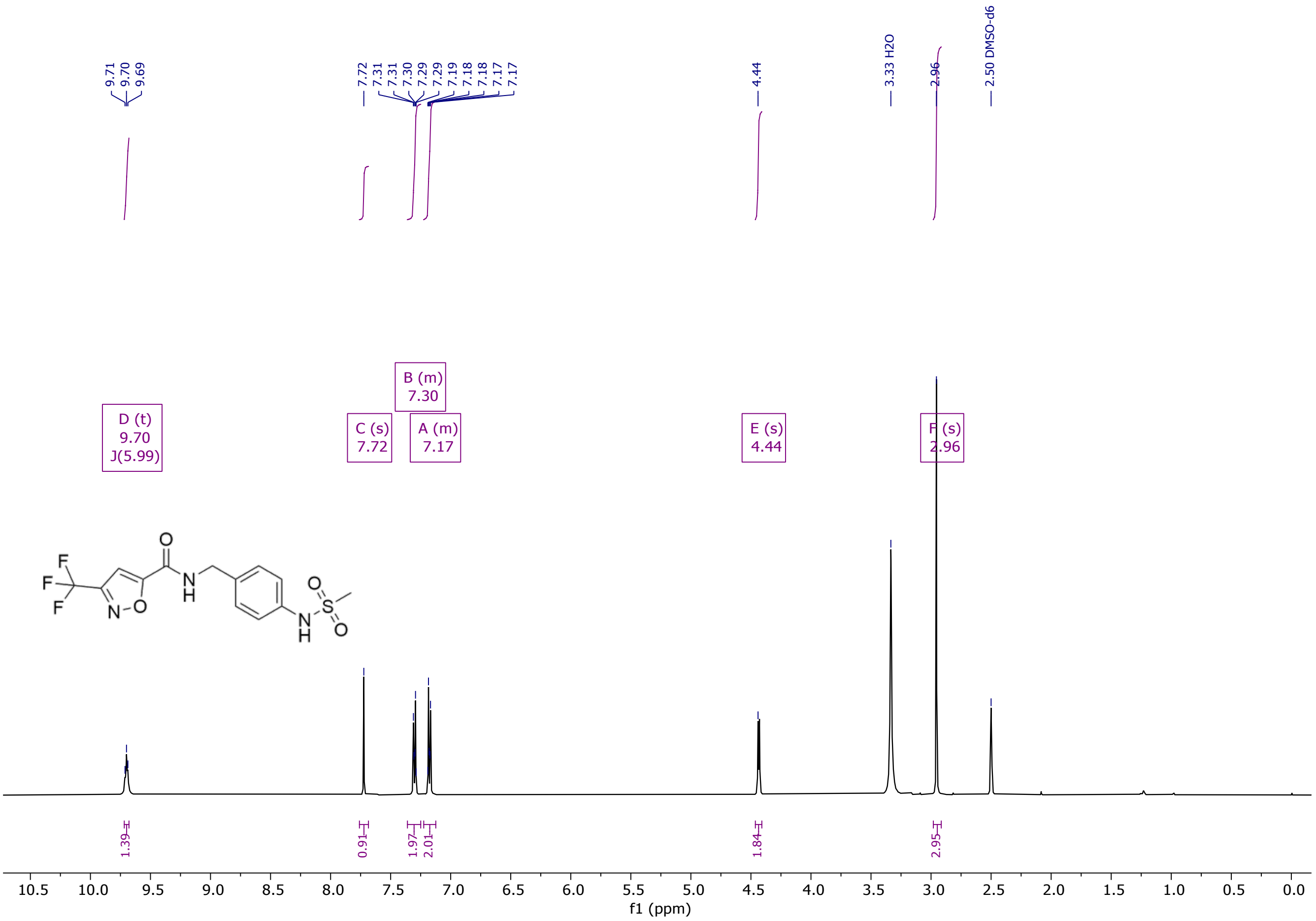

Figure S157: N-(4-(methylsulfonamido)benzyl)-3-(trifluoromethyl)isoxazole-5-carboxamide (S50)

This report was created by ACD/NMR Processor Academic Edition. For more information go to www.acdlabs.com/nmrproc/

Acquisition Time (sec)	2.0447	***Comment***	Dessoy - MAD 2159 - Acetona - Avance 400 MHz - ago23madH1 - 1H		
Date	23 Aug 2021 18:20:48	***File Name***	\\nmrsparc.iqm.unicamp.br\espectros\avance400\2021\ago21\Sala\Luiz Carlos\ago23madH1\ago23madH1_001000fid		
Frequency (MHz)	400.18	***Nucleus***	1H	***Number of Transients***	16
Original Points Count	16384	***Points Count***	16384	***Pulse Sequence***	zg30
Solvent	Acetone	***Spectrum Offset (Hz)***	2464.4622	***Sweep Width (Hz)***	8012.82
Temperature (degree C)	25.178				

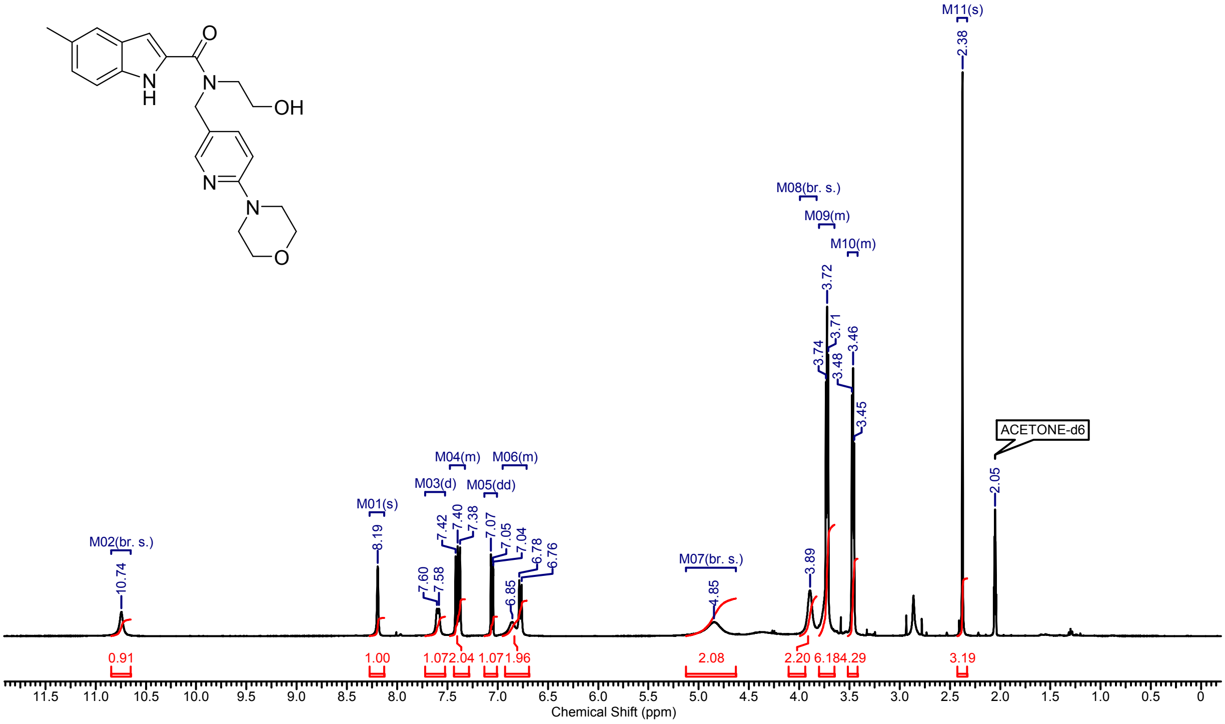

Figure S158: N-(2-hydroxyethyl)-5-methyl-N-((6-morpholinopyridin-3-yl)methyl)-1H-indole-2-carboxamide (S51)

This report was created by ACD/NMR Processor Academic Edition. For more information go to www.acdlabs.com/nmrproc/

Acquisition Time (sec)	2.0447	***Comment***	Dessoy - MAD 2188 - DMSO - Avance 400 MHz - nov18madH1 - 1H			***Date***	18 Nov 2021 15:08:10
File Name	\\nmrsparc.iqm.unicamp.br\espectros\avance400\2021\nov21\Sala\Luiz Carlos\nov18madH1_002001r					***Frequency (MHz)***	400.18
Nucleus	1H	***Number of Transients***	16	***Original Points Count***	16384	***Points Count***	65536
Pulse Sequence	zg30	***Solvent***	DMSO-d6	***Spectrum Offset (Hz)***	2468.0916	***Sweep Width (Hz)***	8012.82
Temperature (degree C)	80.237						

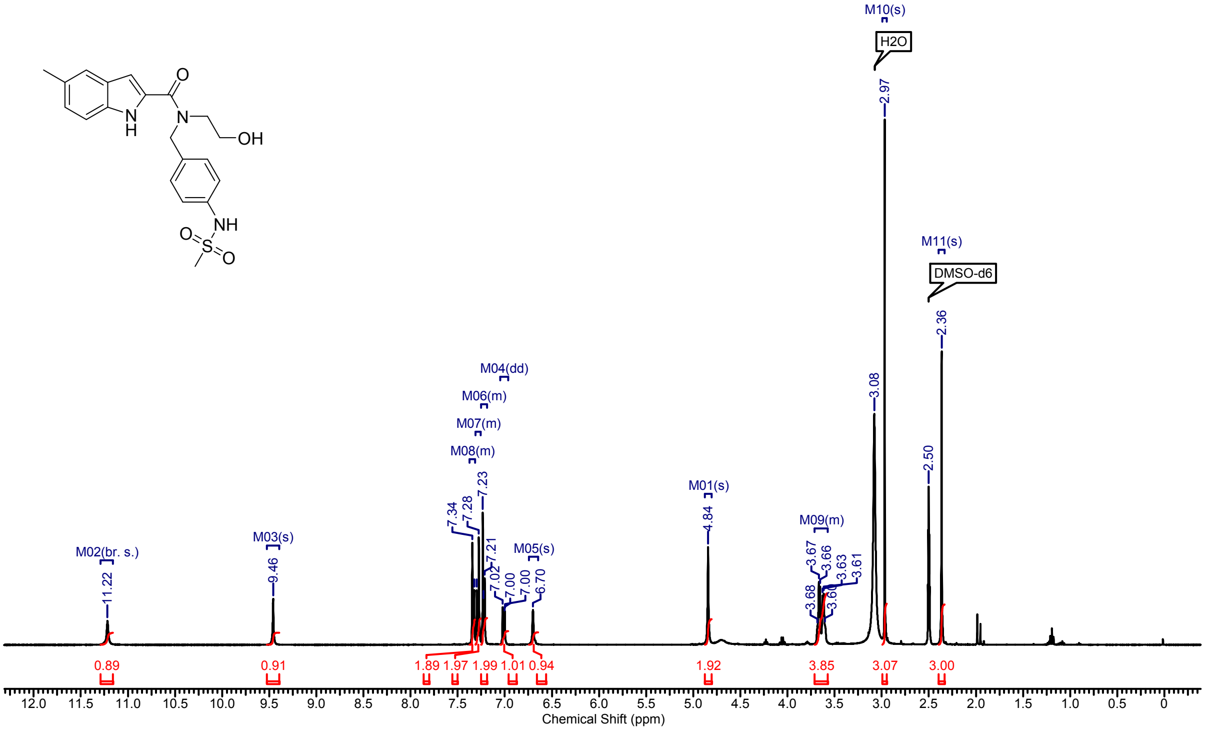

Figure S159: N-(2-hydroxyethyl)-5-methyl-N-(4-(methylsulfonamido)benzyl)-1H-indole-2-carboxamide (S52)

This report was created by ACD/NMR Processor Academic Edition. For more information go to www.acdlabs.com/nmrproc/

Acquisition Time (sec)	3.1807	*Comment*	Dessoy - MAD 2227 - DMSO-d6 - Avance 500 MHz - jan19madH2 - 1H			*Date*	19 Jan 2022 21:16:16
File Name	\\nmrsparc.iqm.unicamp.br\espectros\avance500\2022\jan22\Sala\Luiz Carlos\jan19madH2_001001r					*Frequency (MHz)*	499.87
Nucleus	1H	*Number of Transients*	32	*Original Points Count*	32768	*Points Count*	65536
Pulse Sequence	zg30	*Solvent*	DMSO-d6	*Spectrum Offset (Hz)*	3083.0371	*Sweep Width (Hz)*	10302.20
Temperature (degree C)	25.150						

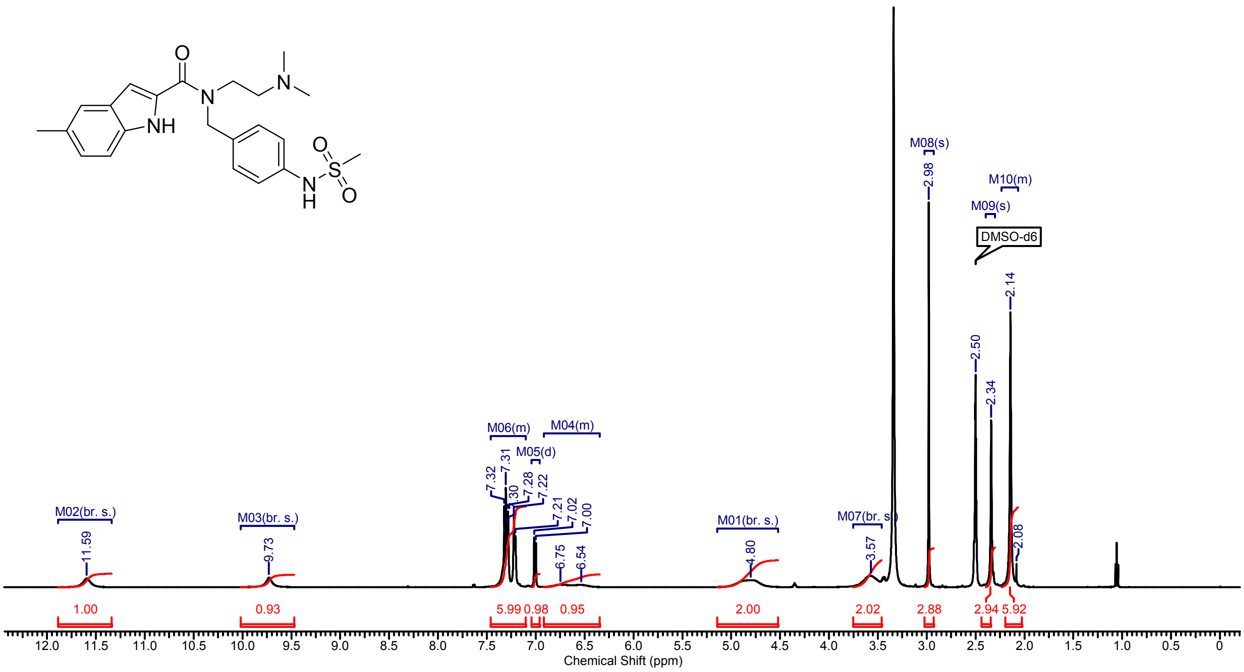

Figure S160: N-(2-(dimethylamino)ethyl)-5-methyl-N-(4-(methylsulfonamido)benzyl)-1H-indole-2-carboxamide (S53)

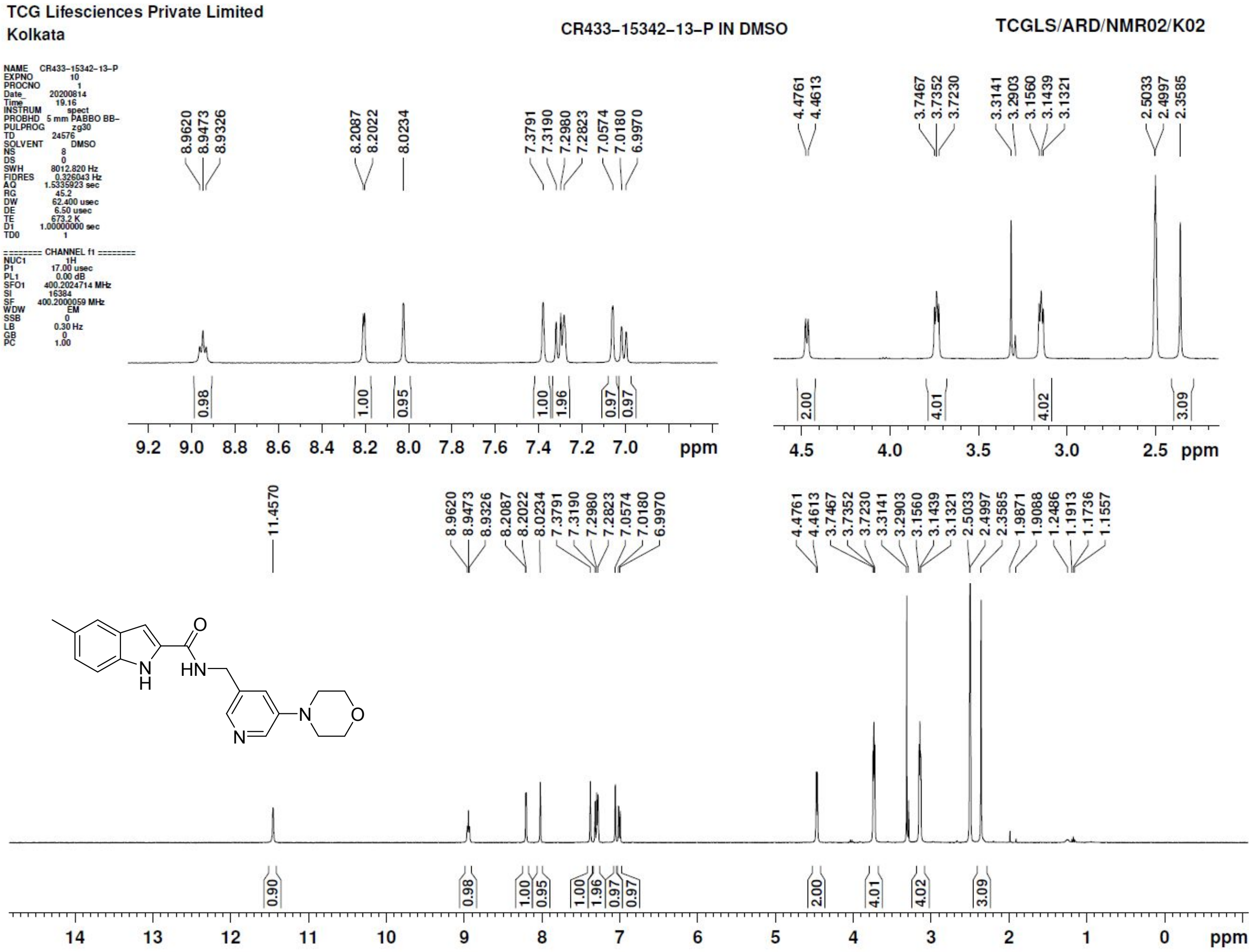

Figure S161: 5-methyl-N-((5-morpholinopyridin-3-yl)methyl)-1H-indole-2-carboxamide (S54)

HPLC purity profile of selected compounds

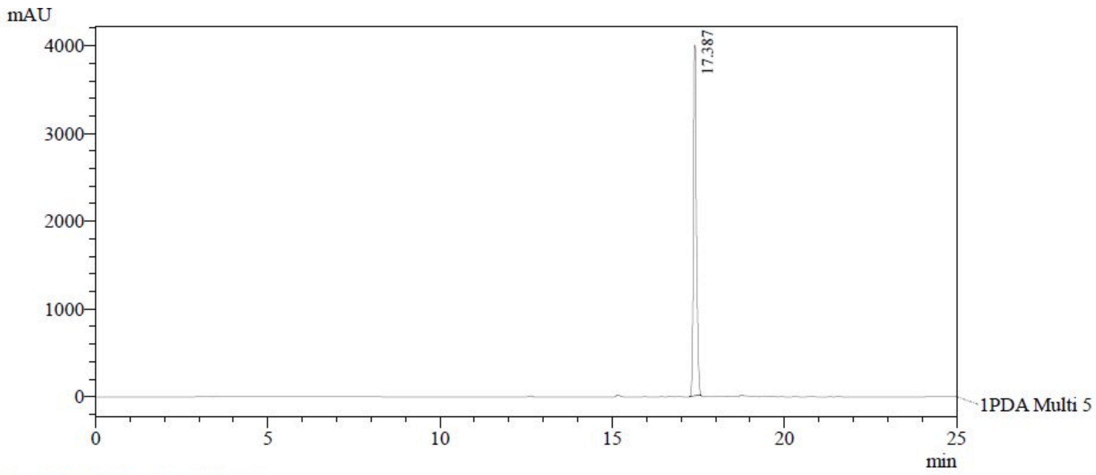

1 PDA Multi 5 / 292nm 4nm

PeakTable

PDA Ch5 292nm 4nm

Peak#	Ret. Time	Area	Height	Area %	Height %
1	17.387	24412767	3988603	100.000	100.000
Total		24412767	3988603	100.000	100.000

Figure S162:HPLC purity profile of 1

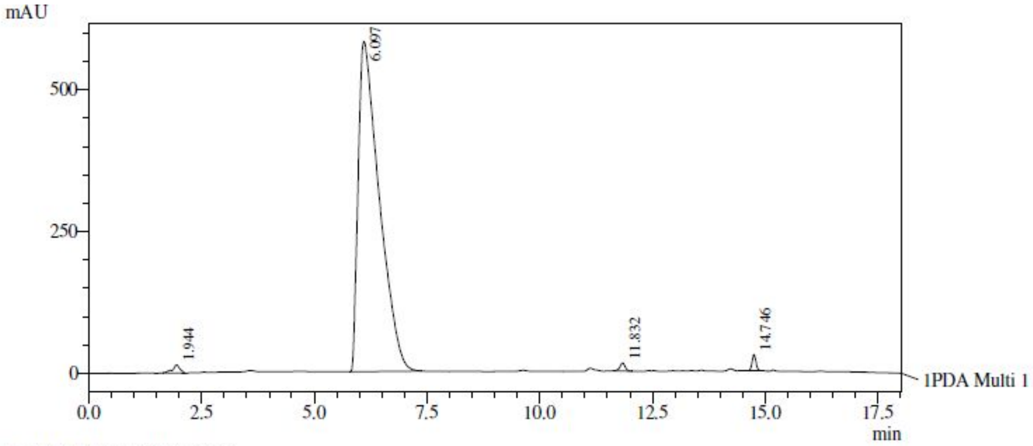

1 PDA Multi 1 / 298nm 4nm

PeakTable

PDA Ch1 298nm 4nm

Peak#	Ret. Time	Area	Height	Area %	Height %
1	1.944	162752	14524	0.841	2.272
2	6.097	18909567	582583	97.722	91.141
3	11.832	113171	14064	0.585	2.200
4	14.746	164946	28040	0.852	4.387
Total		19350436	639211	100.000	100.000

Figure S163: HPLC purity profile of ***2***

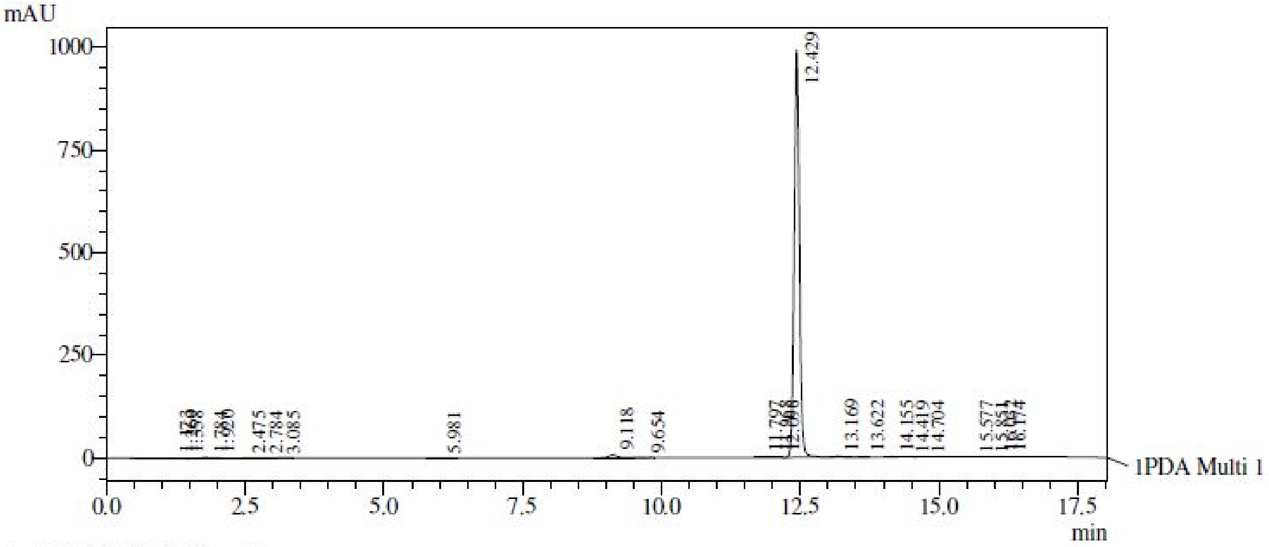

1 PDA Multi 1 / 296nm 4nm

PeakTable

PDA Ch1 296nm 4nm

Peak#	Ret. Time	Area	Height	Area %	Height %
1	1.173	1187	198	0.018	0.020
2	1.269	2191	407	0.033	0.040
3	1.358	3591	656	0.054	0.065
4	1.784	27644	2416	0.416	0.239
5	1.920	10093	1014	0.152	0.100
6	2.475	14846	545	0.223	0.054
7	2.784	3421	363	0.051	0.036
8	3.085	8754	641	0.132	0.063
9	5.981	3567	191	0.054	0.019
10	9.118	76776	6485	1.155	0.641
11	9.654	8313	831	0.125	0.082
12	11.797	1505	143	0.023	0.014
13	11.968	1769	259	0.027	0.026
14	12.096	1311	222	0.020	0.022
15	12.429	6435571	992318	96.848	98.081
16	13.169	18638	2086	0.280	0.206
17	13.622	1465	239	0.022	0.024
18	14.155	1238	143	0.019	0.014
19	14.419	8077	816	0.122	0.081
20	14.704	6018	860	0.091	0.085
21	15.577	1621	179	0.024	0.018
22	15.851	2676	247	0.040	0.024
23	16.032	1454	198	0.022	0.020
24	16.174	3330	279	0.050	0.028
Total		6645057	1011735	100.000	100.000

Figure S164: HPLC purity profile of ***3***

References

1. Bridal TR, Margulis M, Wang X, Donio M, Sorota S. Comparison of Human *Ether-à-go-go* Related Gene Screening Assays Based on IonWorks Quattro and Thallium Flux. *Assay Drug Dev Technol*. 2010;8(6):755-765. doi:10.1089/adt.2010.0267
2. Riley J, Brand S, Voice M, Caballero I, Calvo D, Read KD. Development of a Fluorescence-based Trypanosoma cruzi CYP51 Inhibition Assay for Effective Compound Triaging in Drug Discovery Programmes for Chagas Disease. *PLoS Negl Trop Dis*. 2015;9(9):e0004014. doi:10.1371/journal.pntd.0004014
3. Moraes CB, Giardini MA, Kim H, et al. Nitroheterocyclic compounds are more efficacious than CYP51 inhibitors against Trypanosoma cruzi: implications for Chagas disease drug discovery and development. *Sci Rep*. 2014;4(1):4703. doi:10.1038/srep04703
4. Franco CH, Alcântara LM, Chatelain E, Freitas-Junior L, Moraes CB. Drug Discovery for Chagas Disease: Impact of Different Host Cell Lines on Assay Performance and Hit Compound Selection. *Trop Med Infect Dis*. 2019;4(2):82. doi:10.3390/tropicalmed4020082
5. Lewis MD, Francisco AF, Taylor MC, Kelly JM. A New Experimental Model for Assessing Drug Efficacy against Trypanosoma cruzi Infection Based on Highly Sensitive In Vivo Imaging. *SLAS Discovery*. 2015;20(1):36-43. doi:10.1177/1087057114552623
6. Francisco AF, Lewis MD, Jayawardhana S, Taylor MC, Chatelain E, Kelly JM. Limited Ability of Posaconazole To Cure both Acute and Chronic Trypanosoma cruzi Infections Revealed by Highly Sensitive *In Vivo* Imaging. *Antimicrob Agents Chemother*. 2015;59(8):4653-4661. doi:10.1128/AAC.00520-15
7. da Silva AC, Kratz JM, Morgado PGM, Freitas-Junior LH, Moraes CB. Demystifying In Vivo Bioluminescence Imaging of a Chagas Disease Mouse Model for Drug Efficacy Studies. *Journal of Visualized Experiments*. 2024;(207). doi:10.3791/66740

8. Caddick S, Judd DB, Lewis AK de K, Reich MT, Williams MRV. A generic approach for the catalytic reduction of nitriles. *Tetrahedron*. 2003;59(29):5417-5423. doi:10.1016/S0040-4020(03)00858-5

9. Ren L, Nan G, Wang Y, Xiao Z. Carboxylic Acid-Promoted Single-Step Indole Construction from Simple Anilines and Ketones via Aerobic Cross-Dehydrogenative Coupling. *Journal of Organic Chemistry*. 2018;83(23):14472-14488. doi:10.1021/acs.joc.8b02180

10. Richard-Bildstein S, Aissaoui H, Pothier J, et al. Discovery of the Potent, Selective, Orally Available CXCR7 Antagonist ACT-1004-1239. *J Med Chem*. 2020;63(24):15864-15882. doi:10.1021/acs.jmedchem.0c01588

11. Dolšak A, Bratkovič T, Mlinarič L, et al. Novel Selective IDO1 Inhibitors with Isoxazolo[5,4-d]pyrimidin-4(5H)-one Scaffold. *Pharmaceuticals*. 2021;14(3):265. doi:10.3390/ph14030265

12. Xing C, Wu P, Skibo EB, Dorr RT. Design of Cancer-Specific Antitumor Agents Based on Aziridinylcyclopent[*b*]indoloquinones. *J Med Chem*. 2000;43(3):457-466. doi:10.1021/jm990466w

13. Heaner IV WL, Gelbaum CS, Gelbaum L, et al. Indoles via Knoevenagel–Hemetsberger reaction sequence. *RSC Adv*. 2013;3(32):13232. doi:10.1039/c3ra42296h

14. Henn L, Hickey DMB, Moody CJ, Rees CW. Formation of indoles, isoquinolines, and other fused pyridines from azidoacrylates. *J Chem Soc Perkin 1*. Published online 1984:2189. doi:10.1039/p19840002189
